# A Panel of miRNA Biomarkers Common to Serum and Brain-Derived Extracellular Vesicles Identified in Mouse Model of Amyotrophic Lateral Sclerosis

**DOI:** 10.1007/s12035-023-03857-z

**Published:** 2024-01-22

**Authors:** Natasha Vassileff, Jereme G. Spiers, John D. Lee, Trent M. Woodruff, Esmaeil Ebrahimie, Manijeh Mohammadi Dehcheshmeh, Andrew F. Hill, Lesley Cheng

**Affiliations:** 1 Department of Biochemistry and Chemistry, La Trobe Institute for Molecular Science, La Trobe University, Bundoora, Victoria Australia; 2 Clear Vision Research, Eccles Institute of Neuroscience, John Curtin School of Medical Research, College of Health and Medicine, The Australian National University, Acton, ACT Australia; 3 School of Medicine and Psychology, College of Health and Medicine, The Australian National University, Acton, ACT Australia; 4 School of Biomedical Sciences, The University of Queensland, St. Lucia, Australia; 5 Genomics Research Platform, School of Agriculture, Biomedicine and Environment, La Trobe University, Melbourne, VIC 3000 Australia; 6 School of Animal and Veterinary Sciences, The University of Adelaide, Adelaide, SA 5371 Australia; 7 School of BioSciences, The University of Melbourne, Melbourne, VIC 3010 Australia; 8 Institute for Health and Sport, Victoria University, Footscray, Victoria Australia

**Keywords:** Amyotrophic lateral sclerosis, ALS, miRNA, BDEV, Extracellular vesicles, TDP-43

## Abstract

Amyotrophic lateral sclerosis (ALS) is a progressive motor neuron disease characterised by the deposition of aggregated proteins including TAR DNA-binding protein 43 (TDP-43) in vulnerable motor neurons and the brain. Extracellular vesicles (EVs) facilitate the spread of neurodegenerative diseases and can be easily accessed in the bloodstream. This study aimed to identify a panel of EV miRNAs that can capture the pathology occurring in the brain and peripheral circulation. EVs were isolated from the cortex (BDEVs) and serum (serum EVs) of 3 month-old and 6-month-old TDP-43*Q331K and TDP-43*WT mice. Following characterisation and miRNA isolation, the EVs underwent next-generation sequencing where 24 differentially packaged miRNAs were identified in the TDP-43*Q331K BDEVs and 7 in the TDP-43*Q331K serum EVs. Several miRNAs, including miR-183-5p, were linked to ALS. Additionally, miR-122-5p and miR-486b-5p were identified in both panels, demonstrating the ability of the serum EVs to capture the dysregulation occurring in the brain. This is the first study to identify miRNAs common to both the serum EVs and BDEVs in a mouse model of ALS.

**Supplementary Information:**

The online version contains supplementary material available at 10.1007/s12035-023-03857-z.

## Introduction

Amyotrophic lateral sclerosis (ALS) is an incurable motor neuron disease characterised symptomatically by the progressive loss of motor function resulting from the deposition of aggregated proteins including TAR DNA-binding protein 43 (TDP-43) in motor neurons, spinal cord, and the motor cortex [1, 2]. Despite being the most common motor neuron disease, ALS is notoriously difficult to diagnose, relying on the elimination of other conditions that present overlapping symptoms to achieve a diagnosis [3, 4]. ALS is currently diagnosed based on the Revised El Escorial criteria and Awaji criteria which require upper and lower motor neuron degeneration, disease spread, and disease progression to be observed [5–8]. This stringent criterion results in a length of 10 to 16 months for an average patient to be diagnosed [9], and the requirement for disease progression leads to delayed treatment initiation in many patients [10]. Therefore, there is a need for a faster, more effective, and definitive diagnostic tool, which can be achieved through a blood based liquid biopsy. However, the blood contains many degradative enzymes, requiring specific biomarkers to be protected from cleavage and destruction through encapsulation in small extracellular vesicles (EVs) [11]. Small EVs are a heterogenous population of double lipid-membraned vesicles released from all cells which are involved in cell-cell communication [12, 13]. Their unique biogenesis process which allows them to represent the physiological state of their parental cells, coupled with their ability to cross the blood brain barrier (BBB), enables them to be a source of biomarkers [14, 15]. Furthermore, the BBB is known to deteriorate in response to neuroinflammation, a feature of ALS [16]. Additionally, the preferential CSF uptake and drainage via meningeal lymphatic vessels open a new avenue for brain-derived EVs to enter the bloodstream [16, 17].

Isolation of EVs from the bloodstream enables for the capture of changes being communicated between cells in a disease setting. Importantly, interception of these EVs can reveal pathways being deregulated during the progression of the disease. Plasma EVs from ALS patients exhibit altered size distribution and decreased levels of the heat shock protein HSP90 and miR-494-3p, a negative regulator of semaphorin 3A [18, 19]. CSF EVs have been found to contain downregulated levels of the proteosome like protein, bleomycin hydrolase, and other proteosome core proteins, in addition to enrichment of genes involved in oxidative stress, the unfolded protein response, and the ubiquitin-proteasome pathway [20, 21]. However, a caveat of these studies is the sampling of biofluids from patients at varying stages of their disease. Given EV protein and miRNA expression vary throughout disease progression, the detection of a particular group of markers may not be representative at a different stage in the disease. Therefore, to better diagnose patients at any stage in their disease, and to better understand the role of EVs throughout the disease, a panel of prognostic blood-based EV biomarkers is required, which have not been investigated thus far. Recently, a new application for prognostic EV biomarkers was implemented in Alzheimer’s disease (AD) and Parkinson’s disease (PD). By monitoring changes in their cargo, these EV biomarkers were able to determine the physiological effects selected treatments were having on patients [22–24]. Therefore, the need for a panel of prognostic EV biomarkers is imperative for understanding and managing ALS.

In this study, we isolated serum EVs and cortex brain-derived EVs (BDEVs) from TDP43*Q331K and TDP-43*WT mice, at 3-months-old and 6-months-old, which represented the early-symptomatic stage and the stage which corresponds with prominent motor neuron degeneration [25, 26]. The miRNA cargo of the EVs underwent next-generation deep sequencing (NGS), and panels of differentially packaged miRNAs found in the BDEVs and serum EVs were identified.

## Materials and Methods

### Animals

Transgenic TDP43*Q331K (Jackson Labs Line 103) and TDP-43*WT (Jackson Labs Line 96) mice were bred on a C57BL/6J background, in accordance with La Trobe animal ethics (AEC20014). These mice express either human TDP-43 with a lysine substituting a glutamine at position 331 or human wild-type TDP-43, both under the mouse prion protein promoter which ensured transgenic expression was restricted to the brain, spinal cord, and central nervous system [25–27]. Both female and male mice were used in this experiment to eliminate sex bias. Tissues were collected at 3-months (early-symptomatic stage), 6-months (symptomatic stage), and 10-months (advanced symptomatic stage) of age [25, 26, 28]. All mice were anesthetised with isoflurane for terminal blood collection via the inferior vena cava, and brain cortical tissues were isolated, immediately frozen on dry ice, and stored at −80 °C prior to analysis.

### Genotyping

Mouse ear clips were vortexed and incubated overnight at 55 °C in Extraction Solution (E7526 Sigma-Aldrich) containing proteinase K (PK) at a final concentration of 0.2 mg/ml. Samples were then vortexed and incubated at 95 °C for 3 min before centrifugation at 200 × g for 5 min. The supernatants were precipitated with isopropanol for 5 min at room temperature before being mixed and centrifuged at maximum speed for 5 min. The DNA pellets were washed with 80% (v/v) ethanol and centrifuged at maximum speed for 5 min, air-dried, and resuspended in nuclease-free dH_2_O. DNA samples were added to GoTaq Green Master Mix, 2× (Promega, M7122) with 10 µM of the upstream and downstream primers for the human TARDBP gene and underwent PCR using the following conditions: 95 °C for 30 s; 95 °C for 30 s; 51 °C for 1 min; 68 °C for 1 min; repeated for 30 cycles; 68 °C for 5 min. The samples were then run on a 1.5% (w/v) agarose gel containing SYBRsafe DNA stain (Invitrogen, S33102), and the results were imaged and analysed on the Syngene G:Box Instrument.

### Extracellular Vesicle Isolation from the Cortex Brain Region

A previously published EV isolation protocol was utilised, with minor amendments [29, 30]. Brains collected from 3- or 6-month-old mice were sectioned on ice into 2–3-mm slices. The sliced brain tissue was incubated at 37 °C in a shaking water bath for 10 min in a solution consisting of Collagenase Type III solution (50 U/ml of collagenase (Worthington) in DPBS). The collagenase solution was then deactivated through the addition of ice-cold 10× inhibition solution (5× PhosSTOP (Sigma-Aldrich), 1× cOmplete ULTRA protease inhibitor (Sigma-Aldrich), 2 mM EDTA in DPBS) at a final concentration of 1×. The tissue was then centrifuged at 300 × g for 5 min at 4 °C, and an aliquot of the pellet was saved to use as a total brain control sample. This total brain control sample was treated with 5× its weight of 1× inhibition solution, homogenised, sonicated for 20 min, and centrifuged at 10,000 × g for 5 min at 4 °C, from which the supernatant was saved and referred to as the brain homogenate (BH). The 300 × g supernatant was centrifuged at 2000 × g for 10 min at 4 °C and them at 10,000 × g for 30 min at 4 °C. The supernatant was overlaid on a sucrose gradient consisting of fraction 4 (F4); 1 ml of 2.5 M sucrose, fraction 3 (F3); 1.2 ml of 1.3 M sucrose, fraction 2 (F2); and 1.2 ml of 0.6 M sucrose, in an ultra-clear thin wall 13.2-ml tube (344059, Beckman Coulter). The gradient was centrifuged at 200,000 × g for 180 min at 4 °C in a SW41 rotor (15U12301, Beckman Coulter). The fractions were resuspended in ice cold DPBS and centrifuged at 128,000 × g for 80 min at 4 °C in 26.3-ml polycarbonate centrifuge bottles (355618, Beckman Coulter) in a Type 70 Ti rotor (15U6647, Beckman Coulter). Pellets containing BDEVs were resuspended in 80 µl of DPBS and stored at −80 °C.

### Extracellular Vesicle Isolation from Serum

EVs were isolated from serum samples collected from 3- or 6-month-old mice using the Norgen Plasma/Serum Exosome Purification Mini Kit (NOR-57400, Norgen) according to the manufacturer’s instructions. Briefly, the serum samples were made up to 1 ml prior to addition of ExoC buffer, nuclease free water, and Slurry E. Following incubation for 5 min at room temperature, the samples were centrifuged at 400 × g for 2 min. The pellet was resuspended in ExoR buffer, incubated at room temperature for 5 min and centrifuged at 100 × g for 2 min. The supernatant was added to a Mini Filter Spin column and spun at 1000 × g for 1 min, with the EVs eluting in the flowthrough.

### Size and Concentration Analysis

Size and concentration of the isolated vesicles were determined using nanoparticle tracking analysis (NTA). Following a dilution of 1 in 1000 in filtered and degassed DPBS, the samples were injected through a 1-ml syringe into the ZetaView© Quatt PMX-420 (Particle Metrix). Eleven positions within the instrument’s cell were scanned, each capturing 30 frames per position using the following parameters: maximum particle size, 1000; minimum particle size, 10; minimum brightness, 25; focus, autofocus; sensitivity, 80.0; shutter, 100; and cell temperature, 25 °C. These were then analysed using the in-built ZetaView Software 8.05.14-SP7 to determine the vesicles’ size and concentration.

### Transmission Electron Microscopy

Size and morphology of the isolated vesicles were observed using transmission electron microscopy. Briefly, a formvar-copper coated grid (ProSciTech) was glow discharged for 60 s, loaded with 5 μl of undiluted sample and incubated at room temperature for 30 s. Excess sample was blotted off and the grid was incubated in 5 μl of Uranyl acetate (Agar Scientific) for 10 s, twice. The grid was then imaged using the JEM-2100 Transmission Electron Microscope (Jeol).

### Western Blotting

Protein concentration of the samples was determined using a bicinchoninic acid (BCA) protein assay (Pierce, Thermo Fisher Scientific) according to the manufacturer’s protocol. Equivalent quantities of protein were then incubated in 1× lysis buffer (5 M NaCl, 1M Tris, Triton X-100, 1% (w/v) sodium deoxycholate, 1× cOmplete ULTRA protease inhibitor) at 4 °C for 20 min and subsequently centrifuged at 2500 × g for 5 min. The supernatant was mixed with Nupage 4× LDS sample buffer (Thermo Fisher Scientific, NP0007), containing 5% β-mercaptoethanol, and denatured at 70 °C for 10 min. The samples were then loaded into a 4–12% Bis-Tris Plus Gel (NuPAGE or Bolt, Invitrogen) with 1× MES SDS running buffer (NuPAGE, Invitrogen), transferred to a PVDF membrane, and probed with the desired antibody (Actin, Cell Signalling 8H10D10; Calnexin, Abcam ab22595; Flotillin-1, BD Bioscience 610821; Syntenin-1, Abcam ab133267; Tsg101, Abcam ab83; ApoB, Abcam ab139401; CD9, Abcam ab92726; GM130, BD Bioscience 610822; total TDP-43, Proteintech 10782-2-AP; human specific TDP-43 Proteintech 60019-2-IG-150UL; phosphorylated-TDP-43, Proteintech 800007-1-RR-100UL; C-terminal TDP-43, Proteintech 12892-1-AP) diluted in 2.5% skim milk in PBS-T or TBS-T (0.05%, Tween). Membranes were then washed, incubated in the desired secondary antibody (mouse IgG HRP or rabbit IgG HRP), and developed using Clarity ECL reagent (Bio-Rad) for imaging with the ChemiDoc Touch imaging system (Bio-Rad).

### miRNA Isolation

An Exosomal RNA Isolation Kit (Norgen, 58000) was used according to the manufacturer’s instructions to isolate miRNA from the samples. Briefly, lysis buffer A, lysis additive B, and ExoR buffer were added to the serum or cortex EVs and incubated at room temperature for 10 min. Ethanol was then added, and samples were centrifuged in a Mini Spin column at 3300 × g for 30 s. Wash solution A was then added, and the samples were centrifuged twice at 3300 × g for 30 s, centrifuged empty at 13,000 × g for 1 min, and subsequently transferred to a fresh Elution tube where Elution Solution A was added to the Mini Spin column. This was centrifuged at 400 × g for 1 min followed by two centrifugations at 5800 × g for 2 min. Upon elution of the RNA, the Savant DNA 120 SpeedVac concentrator was used to concentrate the samples to 5–10 µL (Thermo Fisher Scientific).

### Ion Torrent Small RNA Sequencing

The QIAseq miRNA library kit (Qiagen) was used to construct the small RNA libraries according to the manufacturers protocol. Briefly, the Agilent Small RNA Chip and the Agilent 2100 Bioanalyser (Agilent Technologies) were used to assess the small RNA concentration of the samples and 10 ng of small RNA was used for library construction. Library construction involved ligation of sequencing adapters and unique molecular indices for each sample. Libraries were measured using the Agilent DNA 1000 Chip and the Agilent 2100 Bioanalyser (Agilent Technologies) to determine the size distribution and concentration of the cDNA. The Ion Chef System (Ion Torrent, ThermoFisher Scientific) was used to load the indexed libraries onto an Ion 540 Chip (Ion Torrent, Thermo Fisher Scientific) which were then sequenced on the Ion GeneStudio S5 Series (Ion Torrent, Thermo Fisher Scientific).

### Small RNA Sequencing Data Analysis

Adaptor sequences were trimmed and removed from the unaligned sequences using CLC Genomics Workbench (Version 23.02, QIAGEN). The sequences underwent quality control (FASTQC) analysis where the number of sequence reads and size of the fastq files were measured, and per-sequence and per-base analysis were conducted to determine the quality of the sequenced reads. The reads were then aligned to the mouse genome and subsequently mapped to miRBase version 22 [31]. The maximum mismatches between reads and miRBase was set to 2, and length-based isomiRs were allowed with the following criteria: additional upstream bases = 2, additional downstream bases = 2, missing upstream bases = 2, and missing downstream bases = 2. Variability in the sequencing depth per sample generated required a per-sample library size normalisation to be performed using Trimmed Mean of M values (TMM) normalisation [32]. Principal component analysis (PCA) was then performed to measure the quality of expression data and remove outliers using CLC Genomics Workbench (Version 23.02, QIAGEN). The effect of ALS represented by the TDP-43*Q331K mice on control represented by the TDP-43*WT mice was performed using attribute weighting. Attribute weighting was performed using a seven-attribute weighting algorithm including Info Gain Ratio, Rule, Chi Squared, Gini Index, Uncertainty, Relief, and Info Gain, using RapidMiner version 9 (Rapid-I GmbH, Stochumer Str. 475, 44,227, Dortmund, Germany), as previously described [33–35]. Weights of each model were normalised to a range between 0 and 1, with 0 corresponding to non-important and 1 signifying high importance (responding to Q331K mutation). The miRNAs that received the highest weights (sum of the weights of all models) were selected as the representing ALS serum or ALS BDEV miRNA signature.

### Differential Expression Data Analysis

The mapped reads with at least a mean of 5 reads per million (RPM) across all samples underwent differential analysis and analysis of variance (ANOVA) resulting in the generation of Venn diagrams, volcano plots, and hierarchal clustering. The panel of miRNAs underwent pathway analysis using DIANA TOOLS, TargetScanMouse, and FunRich 3.1.3 [36–38]

## Results

### BDEVs Were Successfully Isolated and Classified as Small EVs

**Fig. 1:**
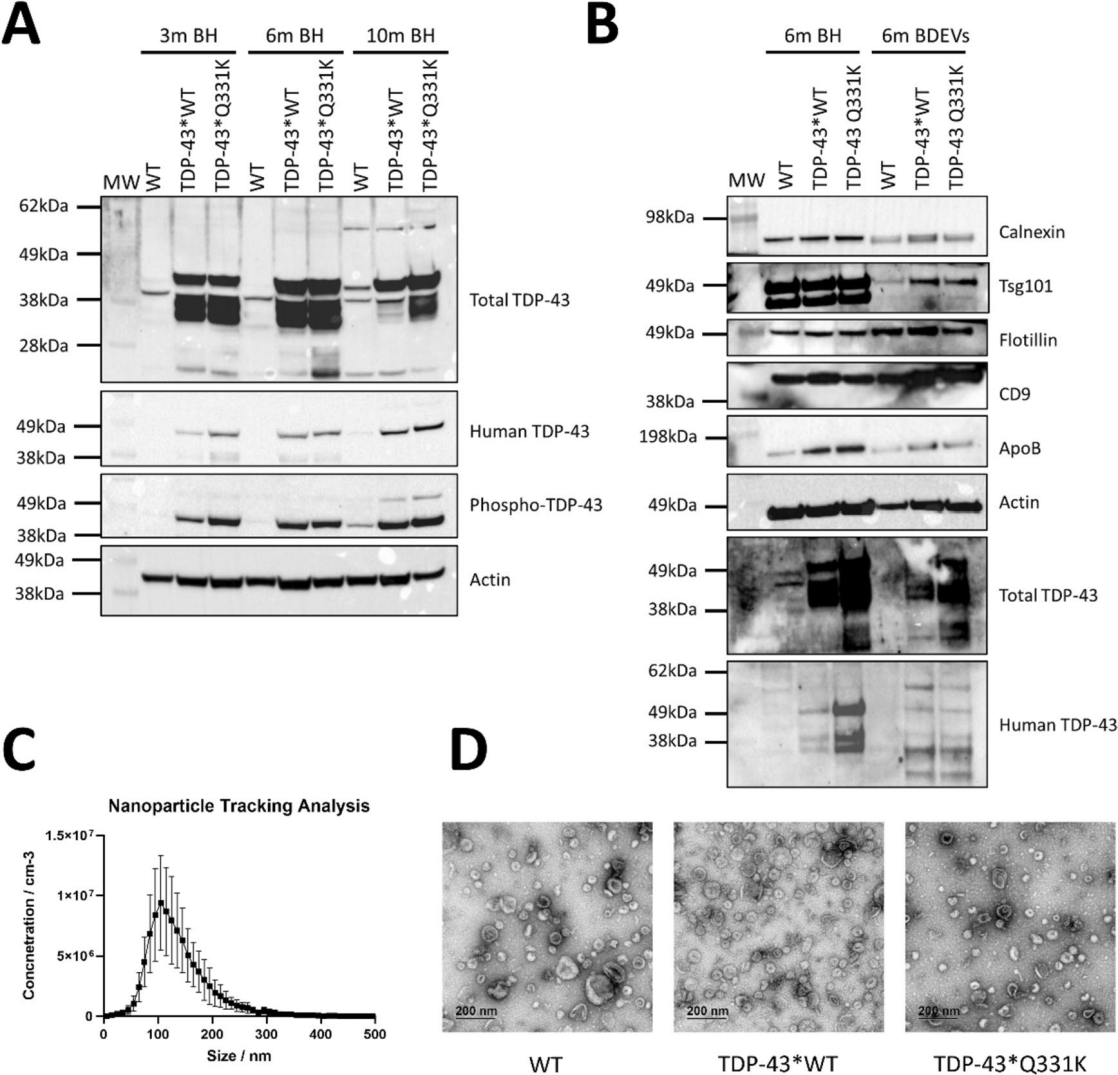
Characterisation of TDP-43 mouse brain homogenate (BH) and brain-derived extracellular vesicles (BDEVs). **A** BH isolated from 3-, 6-, and 10-month-old mice suggests an accumulation of human and phosphorylated TDP-43 with age in the TDP-43*Q331K expressing mice. BDEVs isolated from the brains of 6-month-old wildtype (WT), TDP-43*WT, and TDP-43*Q331K expressing mice appear to exhibit characteristics consistent with that of small EVs. **B** The isolated vesicles were positive for small EV enriched markers tsg101, flotillin, CD9 and actin, and negative for small EV non-enriched markers: calnexin and ApoB. The TDP-43*Q331K expressing mice were also found to contain more total TDP-43 but not human TDP-43 in their BDEVs. **C** Nanoparticle tracking analysis, performed on the ZetaView© Quatt PMX-420, demonstrates the vesicles appear to be between 80 and 150 nm in diameter, consistent with small EVs. This result is representative of *n* = 11. **D** Transmission electron microscopy images exhibit a population of vesicles 100 to 200 nm in diameter with depressed cup-like structures, consistent with that of small EVs

BH isolated from 3-, 6-, and 10-month-old TDP-43*WT, TDP-43*Q331K, and WT were probed with TDP-43 antibodies to assess TDP-43 expression as the mice aged (Fig. 1A). The TDP-43*WT and TDP-43*Q331K mice appeared to exhibit accumulation of human and phosphorylated TDP-43 with age, which appeared greater in the TDP-43*Q331K mice. As we are only focusing on potential biomarkers for an early diagnosis of the disease and investigating early disease changes facilitated through EV communication, BDEVs and serum EVs from 3- and 6-month-old mice were isolated from the TDP-43*WT (*n* = 12) and TDP-43*Q331K mice (*n* = 12), the demographics for which are detailed in Table 1. Quality control assessments were conducted on the BDEVs to ensure they met the Minimal Information for Studies of Extracellular Vesicles (MISEV)’s minimum criteria to be classified as small EVs [39]. The BDEVs isolated from the brains of 6-month-old male TDP-43*WT, TDP-43*Q331K, and WT mice were enriched in small EV markers with minimal expression of non-small EV markers (Fig. 1B). The BDEVs additionally underwent NTA analysis, performed on the ZetaView© Quatt PMX-420 (Particle Metrix), and exhibited a size consistent with that of small EVs (Fig. 1C) [40, 41]. Furthermore, TEM images demonstrated the BDEVs isolated from the TDP-43*WT, TDP-43*Q331K, and WT mice consisted of homogenous cup-shaped vesicle populations, ranging from 40 to 200 nm in diameter (Fig. 1D) [42]. Following successful characterisation, RNA was extracted, and the nucleotide lengths and concentration of the RNA were determined in preparation for NGS analysis (Supplementary Fig. 1A and Supplementary Fig. 1B).

**Table 1:** Sample demographics of TDP-43*Q331K and TDP-43*WT brain-derived extracellular vesicles (BDEVs) and serum extracellular vesicles (EVs)

Sample	Genotype	Timepoint and sex
BDEV 1	TARDBP*Q331K	3m female
BDEV 2	TARDBP*Q331K	3m female
BDEV 3	TARDBP*Q331K	3m female
BDEV 4	TARDBP*Q331K	3m male
BDEV 5	TARDBP*Q331K	3m male
BDEV 6	TARDBP*Q331K	3m male
BDEV 7	TARDBP*WT	3m female
BDEV 8	TARDBP*WT	3m female
BDEV 9	TARDBP*WT	3m female
BDEV 10	TARDBP*WT	3m male
BDEV 11	TARDBP*WT	3m male
BDEV 12	TARDBP*WT	3m male
BDEV 13	TARDBP*Q331K	6m female
BDEV 14	TARDBP*Q331K	6m female
BDEV 15	TARDBP*Q331K	6m female
BDEV 16	TARDBP*Q331K	6m male
BDEV 17	TARDBP*Q331K	6m male
BDEV 18	TARDBP*Q331K	6m male
BDEV 19	TARDBP*WT	6m female
BDEV 20	TARDBP*WT	6m female
BDEV 21	TARDBP*WT	6m female
BDEV 22	TARDBP*WT	6m male
BDEV 23	TARDBP*WT	6m male
BDEV 24	TARDBP*WT	6m male
Serum EV 1	TARDBP*Q331K	3m female
Serum EV 2	TARDBP*Q331K	3m female
Serum EV 3	TARDBP*Q331K	3m female
Serum EV 4	TARDBP*Q331K	3m male
Serum EV 5	TARDBP*Q331K	3m male
Serum EV 6	TARDBP*Q331K	3m male
Serum EV 7	TARDBP*WT	3m female
Serum EV 8	TARDBP*WT	3m female
Serum EV 9	TARDBP*WT	3m female
Serum EV 10	TARDBP*WT	3m male
Serum EV 11	TARDBP*WT	3m male
Serum EV 12	TARDBP*WT	3m male
Serum EV 13	TARDBP*Q331K	6m female
Serum EV 14	TARDBP*Q331K	6m female
Serum EV 15	TARDBP*Q331K	6m female
Serum EV 16	TARDBP*Q331K	6m male
Serum EV 17	TARDBP*Q331K	6m male
Serum EV 18	TARDBP*Q331K	6m male
Serum EV 19	TARDBP*WT	6m female
Serum EV 20	TARDBP*WT	6m female
Serum EV 21	TARDBP*WT	6m female
Serum EV 22	TARDBP*WT	6m male
Serum EV 23	TARDBP*WT	6m male
Serum EV 24	TARDBP*WT	6m male

### A Panel of Differentially Packaged miRNA was Identified in the BDEVs and Serum EVs When Comparing TDP-43*Q331K to TDP-43*WT

**Fig. 2:**
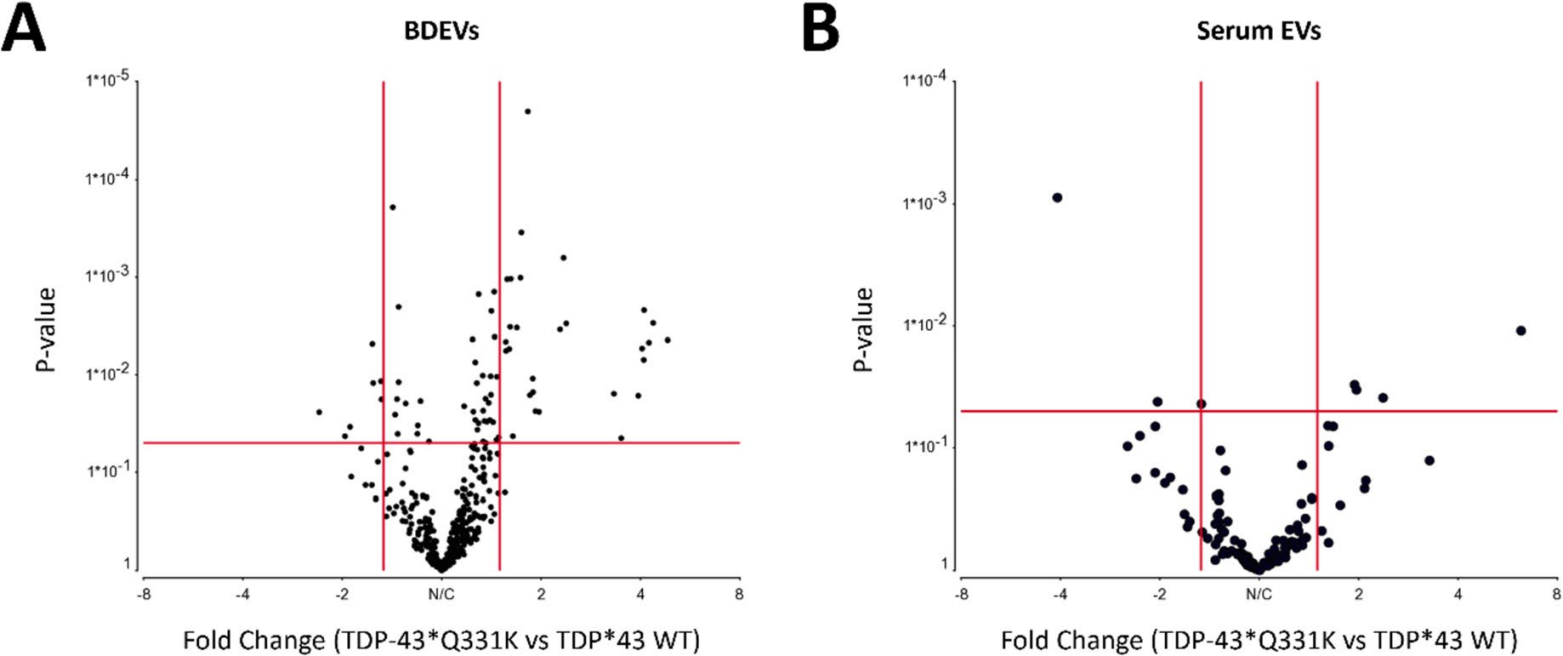
Volcano plot depicting the most abundant miRNAs in the brain-derived extracellular vesicles (BDEVs) and serum extracellular vesicles (EVs). **A** Volcano plot revealing the differential expression of the most abundant miRNAs in the TDP-43*Q331K BDEVs vs TDP-43*wildtype (WT) BDEVs. TDP-43*WT *n* = 12 (3 month-old and 6-month old combined), TDP-43*Q331K *n* = 11 (3-month-old and 6-month-old combined). **B** Volcano plot revealing the differential expression of the most abundant miRNAs in the TDP-43*Q331K serum EVs vs TDP-43*WT serum EVs. TDP-43*WT *n* = 12 (3 month-old and 6-month old combined), TDP-43*Q331K *n* = 11 (3 month-old and 6-month old combined). Differential expression is presented as normalised read counts based on counts per million (CPM), *p*-value = 0.05, fold change < −1.5 and > 1.5. Images created with Partek Genomic Suite

**Fig. 3:**
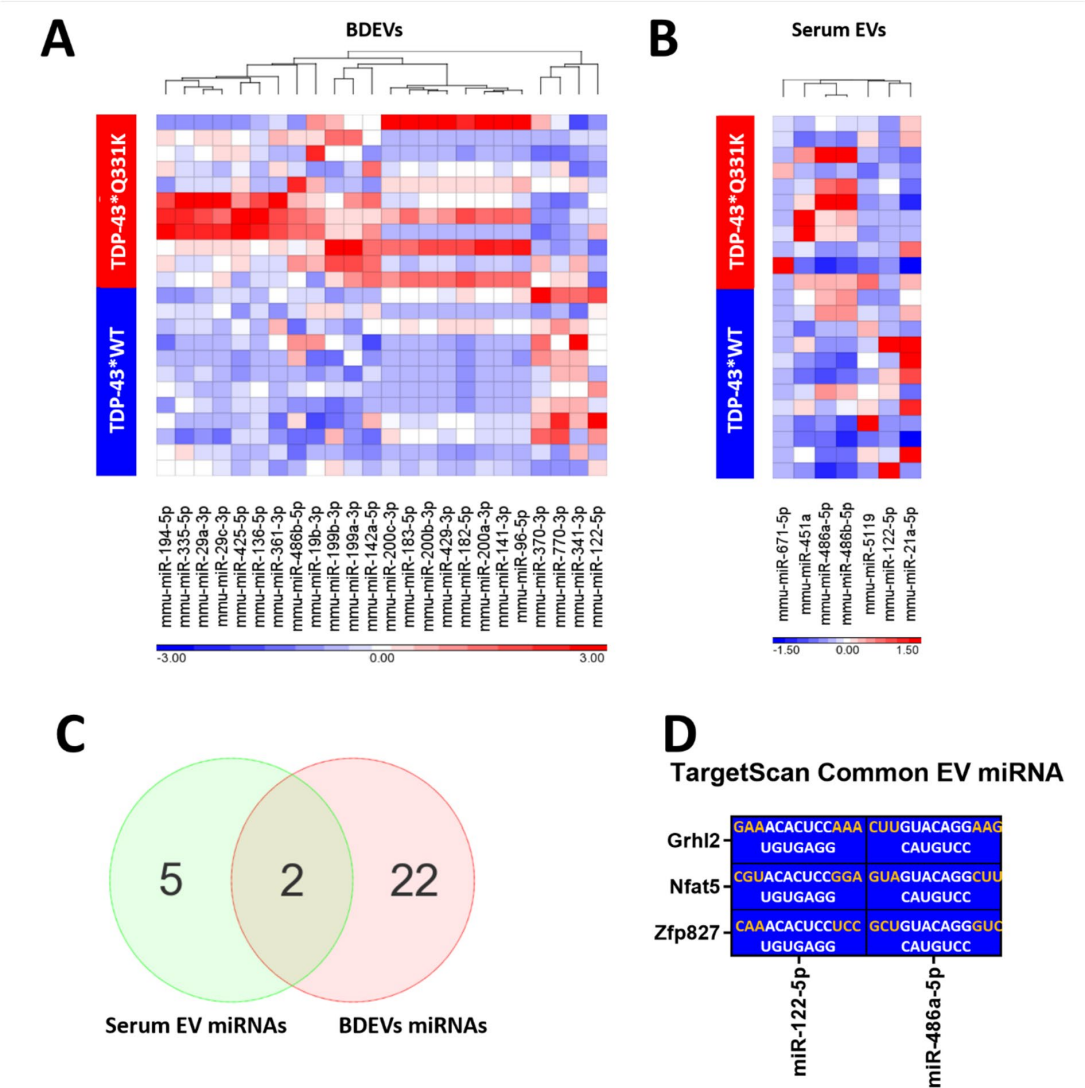
Heatmap depicting the statistically significantly differentially expressed miRNAs in the brain-derived extracellular vesicles (BDEVs) and serum extracellular vesicles (EVs). **A** Heatmap of the 24 statistically significantly differentially expressed miRNAs found in TDP-43*Q331K BDEVs compared to the TDP-43*wildtype (WT) BDEVs. TDP-43*WT *n* = 12, TDP-43*Q331K *n* = 11. **B** Heatmap of the seven statistically significantly differentially expressed miRNAs found in TDP-43*Q331K serum EVs compared to the TDP-43*WT serum EVs. TDP-43*WT *n* = 12, TDP-43*Q331K *n* = 11. Differential expression is presented as normalised read counts based on counts per million (CPM), p-value = 0.05, fold change < −1.5 and > 1.5. **C** Venn Diagram depicting the intersection between the BDEV miRNA panel and the serum EV miRNA panel. **D** The two miRNAs found to be common between the BDEVs and serum EVs appear to target three genes. Images created with Partek and GraphPad Prism

The attribute weighting model revealed that the timepoint and sex of the mice had no effect on the miRNAs differentially expressed between the TDP-43*Q331K EVs and TDP-43*WT EVs (Supplementary Table 1 and Supplementary Table 2). Therefore, for further downstream analysis, differential expression using only the miRNAs detected as features was carried out. It was revealed that several miRNAs appeared to be significantly differentially expressed in both the BDEVs (Fig. 2A; Supplementary Table 3) and serum EVs (Fig. 2B; Supplementary Table 4). Upon refinement of a cut-off *p*-value < 0.05 and a fold change < −1.5 or > 1.5, 24 significantly differentially expressed miRNAs were identified in the TDP-43*Q331K BDEVs compared to the TDP-43*WT BDEVs and 7 significantly differentially expressed miRNAs were identified in the TDP-43*Q331K serum EVs compared to the TDP-43*WT serum EVs (Tables 2 and 3). Interestingly, in both the serum EVs and BDEVs, the majority of these differentially packaged miRNAs appeared to be up-regulated in the TDP-43*Q331K samples (Fig. 3A and B). Specifically, in BDEVs, the vast majority of differentially expressed miRNAs were enriched in the TDP-43*Q331K samples compared to the TDP-43*WT samples, with the exception of mmu-miR-370-3p, mmu-miR-770-3p, mmu-miR-341-3p, and mmu-miR-122-5p. The two miRNA panels were then compared to identify common miRNAs.

**Table 2:** Significant miRNA in brain-derived extracellular vesicles (BDEVs)

miRNA name	Total counts	Maximum counts	Geometric mean	Arithmetic mean	*P*-value	FDR step up	Ratio	Log2(ratio)	Fold change
mmu-miR-199a-3p	3358.78	334.02	133.8	146.03	0.00002	0.0056	1.82	0.87	1.82
mmu-miR-29c-3p	85,509	9360.88	3354.67	3717.78	0.00035	0.032	1.74	0.8	1.74
mmu-miR-136-5p	39,359.5	7158.8	1288.53	1711.28	0.00063	0.041	2.34	1.23	2.34
mmu-miR-361-3p	3335.76	364.41	130.9	145.03	0.001	0.041	1.73	0.79	1.73
mmu-miR-425-5p	7718.57	751.23	313.45	335.59	0.001	0.041	1.58	0.66	1.58
mmu-miR-29a-3p	333,859	37810.8	13,325.8	14515.6	0.001	0.041	1.62	0.7	1.62
mmu-miR-96-5p	11,021.6	2016.63	195.07	479.2	0.0022	0.051	4.1	2.04	4.1
mmu-miR-200a-3p	69,763.5	13231.1	1065.51	3033.2	0.003	0.06	4.37	2.13	4.37
mmu-miR-19b-3p	2891.52	260.1	113.63	125.72	0.0032	0.06	1.61	0.69	1.61
mmu-miR-142a-5p	1503.92	111.34	58.02	65.39	0.0033	0.06	1.69	0.76	1.69
mmu-miR-141-3p	17470.2	3787.33	138.34	759.57	0.0044	0.063	4.83	2.27	4.83
mmu-miR-199b-3p	3131.87	316.44	124.42	136.17	0.0046	0.063	1.56	0.65	1.56
mmu-miR-200b-3p	29,489.1	7265.5	430.55	1282.14	0.0047	0.063	4.24	2.09	4.24
mmu-miR-770-3p	12,717.7	1120.97	496.45	552.94	0.0048	0.063	0.62	−0.7	−1.62
mmu-miR-335-5p	19,381.3	2118.16	757.65	842.67	0.0054	0.065	1.6	0.68	1.6
mmu-miR-429-3p	41,036.3	9106.46	628.55	1784.19	0.0054	0.065	4.04	2.02	4.04
mmu-miR-194-5p	2893.38	277.05	114.94	125.8	0.0057	0.065	1.57	0.65	1.57
mmu-miR-183-5p	29,065.3	7602.77	410.34	1263.71	0.007	0.077	4.09	2.03	4.09
mmu-miR-486b-5p	1028.09	116.61	31.51	44.7	0.011	0.098	1.89	0.92	1.89
mmu-miR-122-5p	1188.32	139.1	45.12	51.67	0.012	0.098	0.62	−0.69	−1.61
mmu-miR-341-3p	4525.72	447.32	177.82	196.77	0.012	0.098	0.65	−0.61	−1.53
mmu-miR-200c-3p	13,018.4	4089.48	104.13	566.02	0.016	0.12	3.94	1.98	3.94
mmu-miR-182-5p	28,750.4	4820.24	475.58	1250.02	0.016	0.12	3.32	1.73	3.32
mmu-miR-370-3p	10,016.9	981.2	391.28	435.52	0.018	0.12	0.66	−0.61	−1.52

**Table 3:** Significant miRNA in serum extracellular vesicles (EVs)

miRNA name	Total counts	Maximum counts	Geometric mean	Arithmetic mean	*P*-value	FDR step up	Ratio	Log2(ratio)	Fold change
mmu-miR-122-5p	796,858.00	264,294.00	3438.70	34,646.00	0.00088	0.10	0.24	−2.03	−4.08
mmu-miR-671-5p	128,563.00	98,039.20	17.02	5589.69	0.011	0.59	6.21	2.63	6.21
mmu-miR-486a-5p	1,073,030.00	175,619.00	29,390.70	46,653.50	0.030	0.67	1.94	0.96	1.94
mmu-miR-486b-5p	1,036,590.00	167,072.00	13,981.20	45,069.10	0.033	0.67	1.97	0.98	1.97
mmu-miR-451a	179,719.00	48,373.60	283.56	7813.86	0.039	0.67	2.37	1.25	2.37
mmu-miR-5119	742,395.00	230,104.00	4465.54	32,278.10	0.042	0.67	0.49	−1.02	−2.03
mmu-miR-21a-5p	762,960.00	72,664.80	5977.85	33,172.20	0.044	0.67	0.67	−0.58	−1.50

Finally, the significantly differentially expressed BDEV miRNA were compared to the differentially expressed serum EV miRNA. Only two miRNAs were found to be common between the BDEV miRNA and the serum EV miRNA (Fig. 3C; Table 4). These two miRNAs, mmu-miR-122-5p and mmu-miR-486a-5p, were found to both target zinc finger protein 827, nuclear factor of activated T cells 5, and grainyhead-like 2 protein (Fig. 3D; Supplementary Table 5). Interestingly, the UGUGAGG region of mmu-miR-122-5p targeted the ACACUCC sequence located on the 3′ end of all three genes. Likewise, the CAUGUCC region in mmu-486a-5p targeted the GUACAGG sequence located on the 3′ end of all three genes. Given these protein targets were not readily associated with neurodegeneration, the pathway targets of the two miRNA panels were investigated separately.

**Table 4:** Common significant miRNA in brain-derived extracellular vesicles (BDEVs) and serum extracellular vesicles (EVs)

	miRNA name	Total counts	Maximum counts	Geometric mean	Arithmetic mean	*P*-value	FDR step up	Ratio	Log2(ratio)	Fold change
Serum EVs	mmu-miR-122-5p	1188.32	139.10	45.12	51.67	0.012	0.098	0.62	−0.69	−1.61
	mmu-miR-486b-5p	1028.09	116.61	31.51	44.70	0.011	0.098	1.89	0.92	1.89
BDEVs	mmu-miR-122-5p	796,858.00	264,294.00	3438.70	34,646.00	0.00088	0.095	0.24	−2.03	−4.08
	mmu-miR-486b-5p	1,036,590.00	167,072.00	13,981.20	45,069.10	0.033	0.67	1.97	0.98	1.97

### BDEV miRNA Panel and Serum EV miRNA Panel Target Protein Clearance and Cell Death Pathways

Analysis of the Kyoto Encyclopedia of Genes and Genomes (KEGG) pathways targeted by the BDEV miRNAs revealed novel targets. The BDEV miRNAs were found to target axon guidance, in addition to sphingolipid metabolism and signalling, a lipid known to compose small EVs [43] (Supplementary Table 6). Protein processing in endoplasmic reticulum and ubiquitin mediated proteolysis were also targeted pathways implying early protein degradation disruption may be occurring. Finally, the panel of miRNAs was revealed to be involved in prion diseases, suggesting some of the miRNAs may be more generally associated with neurodegeneration. Gene Ontology analysis revealed the BDEV miRNAs were also involved in cell death and vesicle-mediated transport (Supplementary Table 7). The latter of which was observed as a target of the serum EV miRNAs (Supplementary Table 9). Interestingly, KEGG pathway analysis of the serum EV miRNAs revealed the miRNAs to be involved in lysine degradation and TGF-β signalling pathway, two pathways that overlap with the BDEV miRNA targets (Supplementary Table 8). Identification of the miRNA gene targets was then conducted to reveal whether these mRNAs encode proteins involved in neurodegeneration.

### The miRNA Gene Targets Are Involved in Regulation of Transcription

**Fig. 4:**
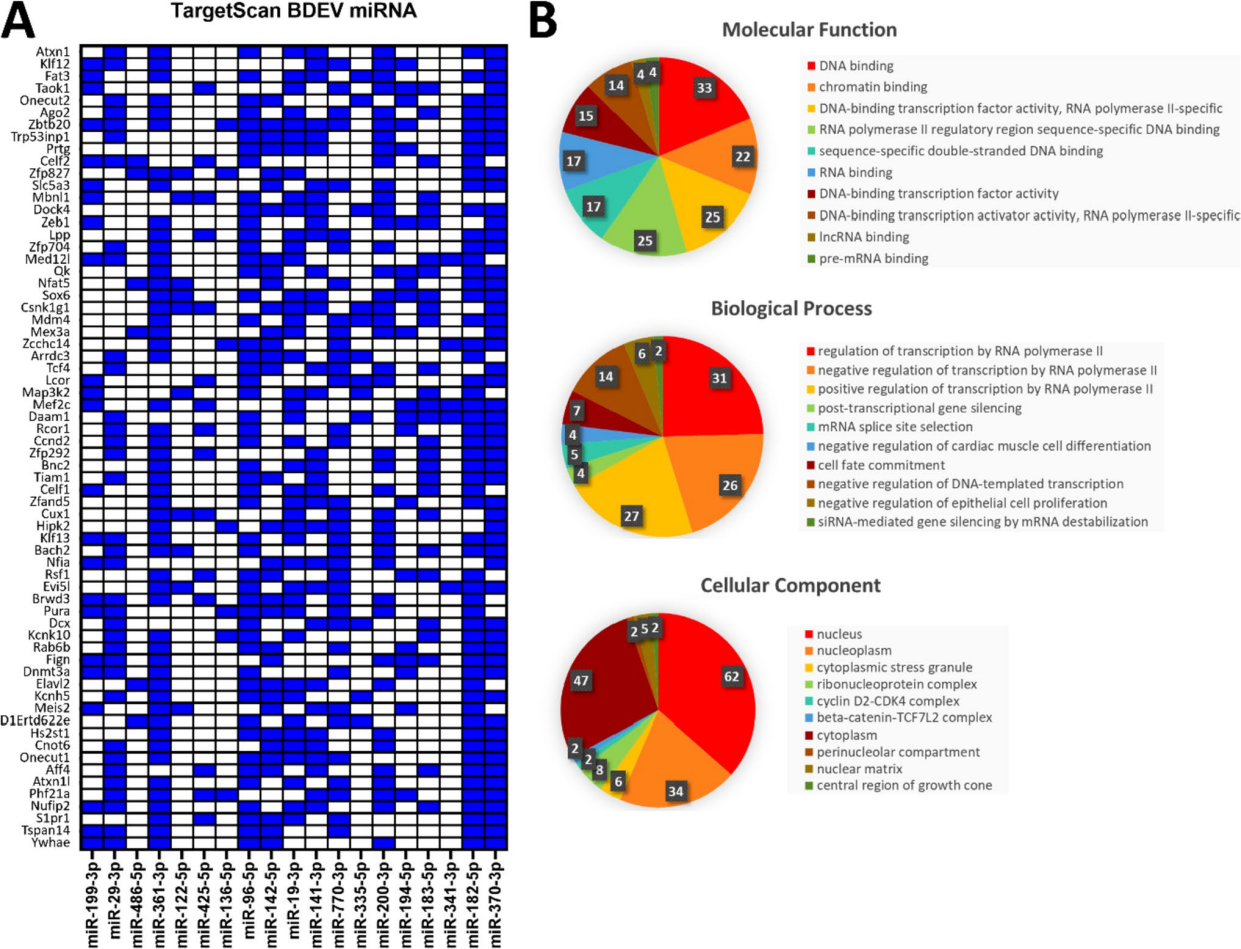
Genes targeted by the brain-derived extracellular vesicles (BDEV) miRNA panel appear to be involved in regulation of transcription by RNA polymerase II. **A** TargetScanMouse revealed the BDEV miRNA panel targeted 66 common genes. Cut-off >8 miRNA per gene. Blue squares represent a miRNA targeting the corresponding gene. **B** Gene Ontology analysis revealed the 66 targeted genes are involved in transcriptional and post-transcriptional regulation and are located in the ribonucleoprotein complex and cytoplasmic stress granules. Images created with GraphPad Prism and FunRich

**Fig. 5:**
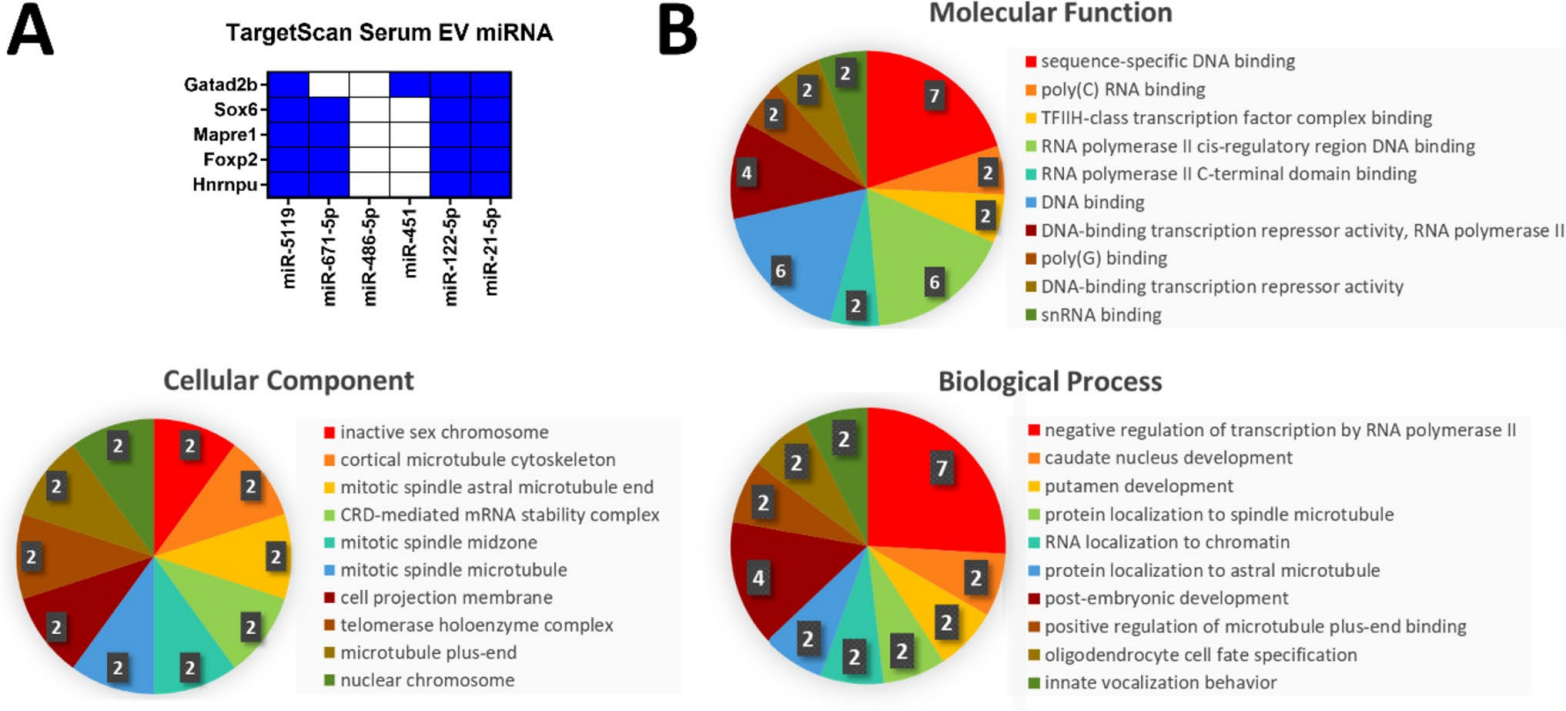
Genes targeted by the serum extracellular vesicles (EV) miRNA panel appear to be involved in negative regulation of transcription. **A** TargetScanMouse revealed the serum EV miRNA panel targeted five common genes. Cut off >4 miRNA per gene. Blue squares represent a miRNA targeting the corresponding gene. **B** Gene Ontology analysis revealed the five targeted genes are involved in DNA and RNA binding and activity. Images created with GraphPad Prism and FunRich

Initially, the targeted mRNA list generated through TargetScan was refined to only include those targeted by two-thirds or more of the miRNA in the BDEV miRNA panel or serum EV miRNA panel (Fig. 4A; Supplementary Table 10; Fig. 5A; Supplementary Table 11). Gene Ontology analysis using FunRich was then applied to the mRNA lists revealing that both the genes targeted by the BDEV miRNA panel, and the serum EV miRNA panel are involved in DNA and RNA binding activity, in addition to the negative regulation of transcription by RNA polymerase II (Figs. 4B and 5B). Interestingly, the gene targets of the BDEV miRNA panel were also found to be involved in the positive regulation of transcription by RNA polymerase II and miRNA regulation, suggesting a feedback mechanism may be facilitated through EVs (Fig. 5B).

## Discussion

The BDEVs and serum EVs were successfully isolated from 3-month and 6-month-old male and female TDP-43*Q331K and TDP-43*WT mice, representing the initial early-symptomatic stage and prominent symptomatic stage of the disease. Following EV isolation and characterisation, sequencing was performed. Through attribute weighting, the age and sex of the mice were not found to influence the mutation status nor influence the differential expression of miRNAs. Therefore, identification of the miRNA biomarker panels was achieved through differential expression analysis. The lack of an effect due to age suggests that the early-symptomatic TDP-43*Q331K animals have progressed far enough into the disease at a molecular level that their EV cargo is indistinguishable from that of the prominently symptomatic 6-month-old mice [25, 26, 28]. This may indicate these markers appear very early in the disease prior to symptomatic onset and the initiation of molecular changes associated with them [25].

Differential expression analysis revealed miRNA panels in both the BDEVs and serum EVs from TDP-43*Q331K and TDP-43*WT mice. In BDEVs, 24 miRNAs were differentially packaged in the TDP-43*Q331K BDEVs compared to the TDP-43*WT BDEVs. The enrichment of miR-96-5p in the BDEVs is consistent with the literature where miR-96-5p has been found to post-transcriptionally regulate Excitatory amino acid carrier 1 (EAAC1), a protein vital for neuronal glutathione production, which is known to be decreased in neurodegeneration [44]. Similarly, though the expression of miR-200a is up-regulated by FUS through a feed-forward regulatory loop, familial associated mutations in the 3′-end of the mRNA render it insensitive to miR-200a regulation [45]. This suggests the increase in miR-200a detected in the BDEVs may be an attempt to regulate an ALS disease-specific pathway dysregulated by ALS-associated proteins such as mutated FUS. miR-199a-3p and miR-183-5p have previously been found to be upregulated in circulating EVs and spinal cords of ALS patients [46, 47]. Although miR-183-5p, miR-451a, and miR-425-5p are found to be down regulated in the peripheral blood of sporadic ALS patients, this may be due to the selective packaging of these miRNAs into EVs or the retainment of these miRNAs in the parental cells [48, 49]. This may be the case for miR-451a, which is upregulated in the leukocytes of sporadic ALS patients where it was implicated in targeting MAPK signalling and apoptosis pathways [50, 51]. Both pathways were found to be targets of the BDEV and serum EV miRNA panels, suggesting an attempt to alleviate the pathogenesis and spread of the disease.

Interestingly, an overlap with other neurodegenerative diseases was observed in this panel of miRNAs with up-regulation of miR-136-5p being detected in the synaptosomes of mice exhibiting pre-clinical prion disease [52]. Prion diseases were identified as a pathway targeted by this miRNA panel through KEGG pathway analysis. The up-regulation of miR-136-5p in the BDEVs may therefore be a general marker for the initial stages of neurodegeneration. Conversely, miR-200b, miR-200c, miR-182-5p, miR-429-3p, and miR-141-3p were found to exhibit decreased expression in synaptosomes of mice in the late stages of prion disease [52]. Therefore, the enrichment of these miRNAs in the TDP-43*Q331K BDEVs may be an early sign of general neurodegenerative processes, with the miRNA expression changing as the disease progresses. In support of this, during the early stages of the disease, prion-infected mice display an up-regulation of miR-29a-3p in hippocampal neurons [53]. miR-29a-3p is known to target Actin-related protein 2/3 complex subunit 3 (ARPC3) which regulates the morphology of dendritic spines and attenuates synaptic overstimulation [53, 54]. Given excitotoxicity is a hallmark of ALS, the upregulation of this miRNA so early in the disease’s progression may be an attempt to prevent neuronal death [55]. Furthermore, miR-141-3p modulates protection of BBB integrity in intracerebral haemorrhage [56]. Additionally, miR-335-5p and miR-29a-3p are down-regulated in the peripheral blood of PD patients, with miR-335-5p also being a critical regulatory miRNA in AD [57–59]. This suggests that although these miRNAs may be more generally associated with neurodegenerative diseases, their selective packaging into EVs may be specific to ALS.

Specifically in ALS, the up-regulation of miR-183-5p has previously been detected in plasma EVs and spinal cords of ALS patients, where it was found to suppress p62 expression and lead to an increased expression of TDP-43 [47, 60]. miR-183-5p is a neuron-enriched miRNA whose overexpression is suggested to increase neuron survival under stress conditions by silencing apoptotic and necroptotic pathways [61]. Antagomirs of TDP-43 have been found to repress formation of stress granules and aggregated TDP-43 under cellular stress [47]. Interestingly, GO analysis of the gene targets of the BDEV miRNA panel revealed association with the ribonucleoprotein complex and cytoplasmic stress granules. These stress granules and aggregated forms of TDP-43 have previously been detected in ALS BDEVs suggesting stress granule formation may be initiated earlier in the disease than expected through miRNA modulation [30]. Likewise, the targeting of protein processing in endoplasmic reticulum and ubiquitin-mediated proteolysis by the BDEV miRNAs suggests protein degradation disruption which is associated with the late stages of ALS may be initiated earlier in the disease [62]. Interestingly both sets of genes targeted by the miRNA panels were determined to be involved in DNA and RNA binding activity, negative regulation of transcription by RNA polymerase II, and gene silencing, suggesting the EVs are potentially modulating a feedback loop. Furthermore, KEGG pathway analysis revealed lysine degradation and the TGF-β signalling pathway to be common targets between the BDEV and serum EV miRNA panels. This further suggests that the serum EV miRNAs are capable of capturing EV-mediated pathways dysregulated in ALS.

This is the first study to identify common miRNAs in both the serum EV and BDEVs in ALS, revealing two miRNAs with the potential to assist in the diagnosis of ALS. Despite the timepoint of sampling exhibiting no effect on the miRNA panels and a conservative sample size, 24 statistically significant differentially packaged miRNAs were identified in the BDEVs and 7 in serum EVs. Some miRNAs have previously been associated with ALS or other neurodegenerative diseases, with only miR-183-5p previously being detected in EVs of ALS patients. Furthermore, the detection of miR-122-5p and miR-486b-5p in both panels of miRNAs, isolated from the same animals, demonstrates the potential of serum EVs to recapitulate the dysregulation occurring in the motor cortex. In the future, a larger sample size and analysis in human samples should be performed to validate these miRNA panels and confirm their association with ALS disease pathogenesis.

### Supplementary Information

Below is the link to the electronic supplementary material.

Supplementary file1 (PDF 451 KB)

Supplementary file2 (PDF 419 KB)

Supplementary file3 (PDF 332 KB)

Supplementary file4 (PDF 246 KB)

Supplementary file5 (PDF 414 KB)

Supplementary file6 (PDF 438 KB)

Supplementary file7 (PDF 784 KB)

Supplementary file8 (PDF 713 KB)

## Data Availability

All data in this manuscript is included in the manuscript or supplemental figures.

## Abbreviations

ALS: Amyotrophic lateral sclerosis
TDP-43: TAR DNA-binding protein 43
EVs: Extracellular vesicles
BBB: Blood-brain barrier
AD: Alzheimer’s disease
PD: Parkinson’s disease
BDEVs: Brain-derived EVs
NGS: Next-generation deep sequencing
PK: Proteinase K
BH: Brain homogenate
NTA: Nanoparticle tracking analysis
BCA: Bicinchoninic acid
TMM: Trimmed mean of M values
PCA: Principal component analysis
RPM: Reads per million
ANOVA: Analysis of variance
EAAC1: Excitatory amino acid carrier 1
ARPC3: Actin-related protein 2/3 complex subunit 3
MISEV: Minimal Information for Studies of Extracellular Vesicles
KEGG: Kyoto Encyclopedia of Genes and Genomes
