# Supplementary material for: A Panel of miRNA Biomarkers Common to Serum and Brain-Derived Extracellular Vesicles Identified in Mouse Model of Amyotrophic Lateral Sclerosis
Source: Mol Neurobiol. 2024 Jan 22;61(8):5901–15. doi: 10.1007/s12035-023-03857-z (PMC11249427; doi:10.1007/s12035-023-03857-z)
Supplement: Supplementary file 1 — Supplementary file1 (PDF 451 KB) [file 12035_2023_3857_MOESM1_ESM.pdf]

Attribute	Weight_Info Gain Ratio	Weight_Rule	Weight_Chi Squared	Weight_Gini Index	Weight_Uncertainty	Weight_Relief	Weight_Info Gain	Sum_weight
mmu-miR-1843a-5p	1.000	1.000	1.000	1.000	1.000	1.000	1.000	7.000
mmu-miR-496a-3p	0.840	1.000	0.895	0.835	0.972	0.588	0.821	5.951
mmu-miR-425-5p	0.840	1.000	0.613	0.835	0.692	0.465	0.821	5.266
mmu-miR-30d-3p	0.713	0.727	0.694	0.701	0.670	0.923	0.697	5.126
mmu-miR-330-5p	0.617	1.000	0.721	0.582	0.680	0.565	0.582	4.746
mmu-miR-381-3p	0.453	1.000	0.962	0.516	0.963	0.269	0.443	4.608
mmu-miR-99b-5p	0.453	1.000	0.836	0.516	0.835	0.478	0.443	4.562
mmu-miR-490-3p	0.617	1.000	0.694	0.582	0.707	0.376	0.582	4.558
mmu-miR-540-3p	0.537	1.000	0.803	0.477	0.788	0.425	0.482	4.511
mmu-miR-6412	0.571	0.909	0.540	0.653	0.506	0.603	0.571	4.353
mmu-miR-29b-1-5p	0.453	0.909	0.688	0.516	0.701	0.543	0.443	4.255
mmu-miR-130a-3p	0.540	1.000	0.704	0.457	0.712	0.305	0.453	4.171
mmu-miR-130b-3p	0.622	0.636	0.606	0.568	0.613	0.531	0.558	4.134
mmu-miR-200a-5p	0.622	0.636	0.519	0.653	0.642	0.460	0.571	4.102
mmu-miR-1198-5p	0.537	1.000	0.540	0.477	0.583	0.482	0.482	4.100
mmu-miR-93-5p	0.469	0.455	0.852	0.516	0.823	0.500	0.443	4.058
mmu-miR-199a-3p	0.622	0.636	0.533	0.582	0.548	0.488	0.582	3.990
mmu-miR-122-5p	0.537	1.000	0.599	0.477	0.599	0.270	0.482	3.963
mmu-miR-29c-3p	0.622	0.818	0.458	0.568	0.513	0.423	0.558	3.959
mmu-miR-770-3p	0.537	1.000	0.546	0.477	0.512	0.357	0.482	3.910
mmu-miR-218-5p	0.455	1.000	0.622	0.524	0.623	0.217	0.455	3.895
mmu-miR-484	0.362	1.000	0.671	0.412	0.639	0.427	0.354	3.866
mmu-miR-154-5p	0.455	1.000	0.507	0.524	0.479	0.421	0.455	3.841
mmu-miR-148a-3p	0.537	0.545	0.589	0.477	0.586	0.564	0.482	3.780
mmu-miR-200a-3p	0.540	0.909	0.465	0.457	0.554	0.339	0.453	3.718
mmu-miR-99a-5p	0.286	1.000	0.852	0.316	0.808	0.149	0.270	3.680
mmu-miR-674-5p	0.469	1.000	0.638	0.384	0.662	0.132	0.393	3.678
mmu-miR-3093-3p	0.537	1.000	0.385	0.477	0.382	0.400	0.482	3.663
mmu-miR-101a-3p	0.409	0.818	0.826	0.302	0.806	0.171	0.313	3.644
mmu-miR-103-3p	0.358	1.000	0.706	0.399	0.728	0.110	0.338	3.640
mmu-miR-341-3p	0.469	1.000	0.589	0.384	0.591	0.205	0.393	3.631
mmu-miR-20b-5p	0.362	0.909	0.630	0.412	0.620	0.331	0.354	3.619
mmu-miR-29a-3p	0.540	0.909	0.456	0.457	0.492	0.277	0.453	3.584
mmu-miR-378d	0.470	0.909	0.688	0.360	0.685	0.102	0.359	3.572
mmu-miR-92b-3p	0.470	1.000	0.694	0.360	0.673	0.003	0.359	3.559
mmu-miR-212-5p	0.258	1.000	0.852	0.274	0.812	0.136	0.223	3.554
mmu-miR-5126	0.362	0.636	0.693	0.412	0.801	0.250	0.354	3.510
mmu-miR-708-5p	0.470	1.000	0.441	0.516	0.468	0.156	0.443	3.496
mmu-let-7d-5p	0.540	1.000	0.441	0.457	0.423	0.177	0.453	3.491
mmu-miR-140-3p	0.470	0.909	0.638	0.360	0.597	0.148	0.359	3.480
mmu-miR-672-5p	0.314	1.000	0.638	0.383	0.617	0.202	0.318	3.473
mmu-miR-6540-5p	0.409	1.000	0.688	0.302	0.664	0.085	0.313	3.460
mmu-miR-146a-5p	0.453	0.273	0.688	0.516	0.660	0.415	0.443	3.449
mmu-miR-22-3p	0.453	0.455	0.619	0.516	0.598	0.360	0.443	3.445
mmu-miR-377-5p	0.470	1.000	0.601	0.360	0.563	0.090	0.359	3.442
mmu-miR-222-3p	0.306	1.000	0.665	0.316	0.642	0.222	0.270	3.420
mmu-miR-101b-3p	0.617	0.636	0.362	0.582	0.405	0.232	0.582	3.416
mmu-miR-137-3p	0.350	1.000	0.796	0.233	0.768	0.064	0.197	3.408
mmu-miR-672-3p	0.356	0.727	0.901	0.228	0.887	0.040	0.240	3.379
mmu-miR-23b-5p	0.286	0.364	0.860	0.316	0.933	0.340	0.270	3.368
mmu-miR-872-3p	0.470	1.000	0.458	0.360	0.468	0.204	0.359	3.318
mmu-miR-330-3p	0.407	1.000	0.638	0.272	0.656	0.051	0.274	3.300
mmu-let-7b-5p	0.350	1.000	0.754	0.193	0.737	0.066	0.197	3.297
mmu-miR-98-3p	0.358	0.455	0.662	0.399	0.683	0.379	0.338	3.275
mmu-miR-26a-2-3p	0.314	0.818	0.622	0.383	0.620	0.191	0.318	3.267
mmu-miR-376b-5p	0.362	1.000	0.431	0.412	0.422	0.262	0.354	3.244
mmu-miR-455-5p	0.409	1.000	0.573	0.302	0.587	0.048	0.313	3.231
mmu-miR-1843b-5p	0.362	1.000	0.382	0.412	0.355	0.360	0.354	3.227
mmu-miR-344d-3-5p	0.356	0.727	0.540	0.379	0.582	0.318	0.313	3.214
mmu-miR-324-5p	0.540	0.545	0.468	0.457	0.465	0.276	0.453	3.204
mmu-let-7i-5p	0.350	1.000	0.556	0.298	0.533	0.206	0.250	3.193
mmu-miR-183-5p	0.540	1.000	0.268	0.457	0.370	0.105	0.453	3.193
mmu-miR-669a-3p	0.470	0.636	0.471	0.379	0.528	0.333	0.359	3.176
mmu-miR-409-3p	0.470	1.000	0.392	0.360	0.362	0.219	0.359	3.161
mmu-miR-96-5p	0.470	0.909	0.373	0.360	0.433	0.246	0.359	3.149
mmu-miR-542-3p	0.470	1.000	0.376	0.360	0.360	0.218	0.359	3.142
mmu-miR-151-3p	0.409	1.000	0.514	0.302	0.519	0.085	0.313	3.142
mmu-let-7c-5p	0.350	1.000	0.638	0.193	0.623	0.123	0.197	3.124
mmu-miR-223-3p	0.470	0.636	0.392	0.412	0.384	0.470	0.359	3.123
mmu-miR-421-3p	0.409	1.000	0.474	0.302	0.470	0.153	0.313	3.121
mmu-miR-30c-1-3p	0.407	1.000	0.573	0.272	0.560	0.016	0.274	3.102
mmu-miR-200b-3p	0.470	1.000	0.343	0.360	0.442	0.127	0.359	3.100
mmu-miR-99a-3p	0.294	0.818	0.638	0.298	0.613	0.186	0.250	3.097
mmu-miR-127-3p	0.314	1.000	0.546	0.383	0.516	0.011	0.318	3.089
mmu-miR-7231-5p	0.469	0.455	0.366	0.384	0.580	0.434	0.393	3.080
mmu-miR-335-5p	0.470	1.000	0.333	0.360	0.361	0.187	0.359	3.070
mmu-miR-340-5p	0.356	1.000	0.589	0.228	0.589	0.063	0.240	3.065
mmu-miR-298-5p	0.409	1.000	0.468	0.302	0.482	0.086	0.313	3.060
mmu-miR-132-5p	0.356	1.000	0.596	0.228	0.617	0.021	0.240	3.058
mmu-miR-370-3p	0.409	0.727	0.491	0.316	0.471	0.316	0.313	3.043

Attribute	Weight_Info Gain Ratio	Weight_Rule	Weight_Chi Squared	Weight_Gini Index	Weight_Uncertainty	Weight_Relief	Weight_Info Gain	Sum_weight
mmu-miR-181a-2-3p	0.409	1.000	0.432	0.302	0.426	0.147	0.313	3.029
mmu-miR-301b-3p	0.313	0.182	0.786	0.379	0.762	0.291	0.313	3.026
mmu-miR-338-3p	0.470	1.000	0.324	0.360	0.311	0.198	0.359	3.021
mmu-miR-141-3p	0.470	0.818	0.343	0.360	0.430	0.229	0.359	3.008
mmu-miR-192-5p	0.407	1.000	0.497	0.272	0.472	0.083	0.274	3.005
mmu-miR-490-5p	0.306	1.000	0.671	0.162	0.632	0.042	0.173	2.987
mmu-miR-3078-5p	0.469	0.636	0.441	0.384	0.471	0.192	0.393	2.986
mmu-miR-382-5p	0.470	0.909	0.376	0.360	0.389	0.113	0.359	2.975
mmu-miR-708-3p	0.350	1.000	0.579	0.193	0.604	0.050	0.197	2.973
mmu-miR-30b-5p	0.356	1.000	0.568	0.233	0.541	0.027	0.240	2.965
mmu-miR-135b-5p	0.350	0.909	0.507	0.274	0.481	0.208	0.223	2.951
mmu-miR-186-5p	0.470	1.000	0.359	0.360	0.378	0.019	0.359	2.944
mmu-miR-7a-5p	0.294	1.000	0.648	0.122	0.616	0.131	0.126	2.937
mmu-miR-124-5p	0.234	1.000	0.638	0.233	0.600	0.033	0.197	2.936
mmu-miR-190b-5p	0.258	1.000	0.694	0.162	0.673	0.008	0.135	2.931
mmu-miR-380-3p	0.350	1.000	0.573	0.193	0.529	0.085	0.197	2.926
mmu-miR-544-3p	0.350	1.000	0.532	0.193	0.570	0.064	0.197	2.906
mmu-miR-15b-5p	0.407	0.909	0.448	0.272	0.431	0.162	0.274	2.903
mmu-miR-574-3p	0.356	1.000	0.523	0.228	0.521	0.030	0.240	2.899
mmu-miR-362-3p	0.350	0.727	0.415	0.383	0.385	0.317	0.318	2.895
mmu-miR-20a-5p	0.455	0.273	0.390	0.524	0.404	0.394	0.455	2.894
mmu-miR-935	0.234	1.000	0.688	0.162	0.644	0.021	0.135	2.883
mmu-miR-127-5p	0.258	1.000	0.678	0.107	0.657	0.073	0.110	2.883
mmu-miR-145a-5p	0.470	0.909	0.307	0.360	0.300	0.177	0.359	2.881
mmu-miR-374b-5p	0.306	1.000	0.573	0.162	0.579	0.088	0.173	2.880
mmu-miR-361-5p	0.409	1.000	0.402	0.302	0.388	0.065	0.313	2.878
mmu-miR-217-5p	0.470	0.455	0.498	0.360	0.579	0.148	0.359	2.867
mmu-miR-6958-3p	0.220	0.909	0.593	0.233	0.697	0.015	0.197	2.865
mmu-miR-301a-3p	0.356	1.000	0.498	0.228	0.508	0.030	0.240	2.859
mmu-miR-22-5p	0.306	1.000	0.474	0.270	0.429	0.157	0.219	2.855
mmu-miR-142a-5p	0.409	0.364	0.589	0.316	0.585	0.271	0.313	2.848
mmu-miR-351-5p	0.294	1.000	0.629	0.131	0.611	0.058	0.126	2.847
mmu-miR-3547-3p	0.470	0.545	0.496	0.360	0.484	0.133	0.359	2.846
mmu-miR-93-3p	0.234	1.000	0.612	0.179	0.594	0.084	0.143	2.846
mmu-miR-15a-5p	0.258	1.000	0.500	0.233	0.473	0.177	0.197	2.839
mmu-miR-541-5p	0.407	1.000	0.395	0.272	0.420	0.070	0.274	2.838
mmu-miR-218-2-3p	0.356	1.000	0.491	0.228	0.486	0.036	0.240	2.836
mmu-miR-9-5p	0.206	1.000	0.671	0.101	0.648	0.126	0.083	2.835
mmu-miR-7010-5p	0.350	0.545	0.376	0.298	0.510	0.506	0.250	2.834
mmu-miR-378c	0.306	1.000	0.408	0.298	0.382	0.187	0.250	2.832
mmu-miR-29b-3p	0.470	0.909	0.261	0.360	0.309	0.151	0.359	2.819
mmu-miR-340-3p	0.306	0.909	0.533	0.233	0.523	0.110	0.197	2.812
mmu-miR-3106-5p	0.306	1.000	0.556	0.162	0.525	0.089	0.173	2.811
mmu-miR-1981-5p	0.350	1.000	0.474	0.193	0.546	0.049	0.197	2.809
mmu-miR-344b-3p	0.356	1.000	0.497	0.233	0.461	0.021	0.240	2.808
mmu-miR-700-5p	0.407	0.818	0.472	0.272	0.475	0.087	0.274	2.805
mmu-miR-3473a	0.294	1.000	0.606	0.162	0.603	0.003	0.135	2.803
mmu-miR-1983	0.409	1.000	0.349	0.302	0.346	0.082	0.313	2.801
mmu-miR-194-5p	0.470	0.727	0.303	0.360	0.306	0.274	0.359	2.800
mmu-miR-466f-3p	0.258	0.909	0.538	0.211	0.589	0.120	0.174	2.799
mmu-miR-412-3p	0.469	0.455	0.323	0.384	0.469	0.304	0.393	2.796
mmu-miR-1298-5p	0.294	1.000	0.408	0.298	0.467	0.074	0.250	2.791
mmu-miR-25-3p	0.232	1.000	0.491	0.283	0.467	0.083	0.232	2.787
mmu-miR-676-3p	0.206	1.000	0.655	0.072	0.620	0.174	0.058	2.785
mmu-miR-181b-5p	0.294	1.000	0.566	0.122	0.541	0.135	0.126	2.784
mmu-miR-541-3p	0.234	0.727	0.663	0.186	0.700	0.121	0.150	2.782
mmu-miR-429-3p	0.470	0.818	0.270	0.360	0.331	0.166	0.359	2.773
mmu-miR-28a-3p	0.258	0.909	0.606	0.183	0.578	0.093	0.147	2.773
mmu-miR-3535	0.206	1.000	0.678	0.072	0.630	0.129	0.058	2.772
mmu-miR-337-5p	0.350	1.000	0.507	0.193	0.483	0.027	0.197	2.756
mmu-miR-153-3p	0.306	1.000	0.568	0.162	0.541	0.005	0.173	2.756
mmu-miR-376c-3p	0.407	1.000	0.408	0.272	0.389	0.004	0.274	2.756
mmu-miR-379-3p	0.294	1.000	0.589	0.122	0.541	0.079	0.126	2.750
mmu-miR-1b-3p	0.306	0.545	0.487	0.283	0.612	0.283	0.232	2.748
mmu-miR-181b-1-3p	0.350	0.455	0.502	0.316	0.474	0.381	0.270	2.747
mmu-miR-129-2-3p	0.350	1.000	0.491	0.193	0.447	0.068	0.197	2.745
mmu-miR-101c	0.407	0.727	0.441	0.272	0.471	0.141	0.274	2.733
mmu-miR-667-3p	0.294	1.000	0.441	0.233	0.442	0.122	0.197	2.729
mmu-miR-139-5p	0.350	1.000	0.446	0.193	0.439	0.102	0.197	2.727
mmu-miR-532-5p	0.407	1.000	0.313	0.272	0.331	0.126	0.274	2.723
mmu-let-7a-5p	0.350	1.000	0.435	0.193	0.413	0.130	0.197	2.717
mmu-miR-1264-3p	0.258	1.000	0.556	0.183	0.543	0.030	0.147	2.717
mmu-miR-384-5p	0.350	0.909	0.504	0.193	0.526	0.037	0.197	2.717
mmu-miR-3077-3p	0.407	0.364	0.366	0.272	0.610	0.421	0.274	2.714
mmu-miR-423-3p	0.350	1.000	0.430	0.193	0.474	0.066	0.197	2.710
mmu-miR-3082-3p	0.358	0.000	0.454	0.399	0.616	0.536	0.338	2.703
mmu-miR-29b-2-5p	0.258	1.000	0.540	0.183	0.513	0.061	0.147	2.702
mmu-miR-369-3p	0.350	1.000	0.474	0.199	0.472	0.008	0.197	2.700
mmu-miR-448-3p	0.350	1.000	0.402	0.211	0.398	0.142	0.197	2.699
mmu-miR-652-3p	0.407	1.000	0.356	0.272	0.364	0.025	0.274	2.699

Attribute	Weight_Info Gain Ratio	Weight_Rule	Weight_Chi Squared	Weight_Gini Index	Weight_Uncertainty	Weight_Relief	Weight_Info Gain	Sum_weight
mmu-miR-431-3p	0.228	1.000	0.497	0.274	0.472	0.006	0.223	2.698
mmu-miR-376a-3p	0.350	1.000	0.455	0.193	0.444	0.056	0.197	2.695
mmu-miR-212-3p	0.258	1.000	0.599	0.102	0.607	0.014	0.110	2.691
mmu-miR-7b-5p	0.306	1.000	0.500	0.162	0.477	0.072	0.173	2.690
mmu-miR-150-5p	0.350	1.000	0.468	0.193	0.480	0.000	0.197	2.687
mmu-miR-101a-5p	0.350	0.455	0.424	0.383	0.498	0.259	0.318	2.687
mmu-miR-382-3p	0.407	0.455	0.514	0.283	0.519	0.232	0.274	2.684
mmu-miR-221-3p	0.350	1.000	0.458	0.211	0.442	0.011	0.197	2.667
mmu-miR-664-5p	0.409	1.000	0.310	0.302	0.290	0.040	0.313	2.663
mmu-miR-410-3p	0.306	1.000	0.498	0.162	0.515	0.006	0.173	2.659
mmu-miR-671-5p	0.294	1.000	0.466	0.211	0.479	0.028	0.174	2.652
mmu-miR-350-3p	0.407	0.818	0.448	0.272	0.407	0.021	0.274	2.647
mmu-miR-203-3p	0.306	1.000	0.507	0.162	0.481	0.014	0.173	2.643
mmu-miR-3083b-3p	0.470	0.455	0.343	0.360	0.442	0.207	0.359	2.635
mmu-miR-3059-5p	0.306	1.000	0.468	0.162	0.465	0.059	0.173	2.632
mmu-miR-466i-5p	0.356	1.000	0.379	0.228	0.381	0.048	0.240	2.632
mmu-miR-29a-5p	0.356	1.000	0.376	0.228	0.340	0.090	0.240	2.630
mmu-miR-6238	0.470	1.000	0.156	0.360	0.241	0.041	0.359	2.626
mmu-miR-377-3p	0.234	1.000	0.441	0.211	0.489	0.074	0.174	2.624
mmu-miR-125b-2-3p	0.350	0.909	0.458	0.211	0.433	0.065	0.197	2.622
mmu-miR-879-5p	0.234	1.000	0.596	0.114	0.573	0.014	0.091	2.621
mmu-miR-34c-3p	0.356	1.000	0.353	0.228	0.357	0.088	0.240	2.621
mmu-miR-673-5p	0.356	1.000	0.367	0.228	0.401	0.029	0.240	2.621
mmu-miR-9-3p	0.350	1.000	0.380	0.193	0.425	0.065	0.197	2.610
mmu-miR-1191a	0.286	1.000	0.340	0.316	0.328	0.069	0.270	2.609
mmu-miR-1843b-3p	0.350	1.000	0.408	0.193	0.386	0.074	0.197	2.609
mmu-miR-134-5p	0.407	0.455	0.458	0.298	0.446	0.267	0.274	2.605
mmu-miR-3085-3p	0.294	1.000	0.388	0.233	0.477	0.015	0.197	2.605
mmu-miR-125a-3p	0.234	1.000	0.573	0.135	0.529	0.020	0.110	2.601
mmu-miR-300-5p	0.258	0.818	0.556	0.199	0.514	0.093	0.162	2.600
mmu-miR-877-3p	0.306	0.818	0.573	0.162	0.541	0.021	0.173	2.594
mmu-miR-760-3p	0.356	0.909	0.376	0.233	0.343	0.134	0.240	2.590
mmu-miR-383-3p	0.306	0.636	0.614	0.183	0.611	0.066	0.173	2.590
mmu-miR-383-5p	0.350	0.273	0.517	0.383	0.503	0.246	0.318	2.590
mmu-miR-31-5p	0.234	1.000	0.392	0.283	0.396	0.052	0.232	2.589
mmu-miR-802-5p	0.470	0.000	0.343	0.360	0.589	0.467	0.359	2.587
mmu-miR-23a-3p	0.234	1.000	0.579	0.114	0.548	0.016	0.091	2.582
mmu-miR-3473b	0.294	1.000	0.474	0.122	0.468	0.095	0.126	2.579
mmu-let-7f-2-3p	0.234	1.000	0.458	0.211	0.462	0.039	0.174	2.577
mmu-miR-195a-5p	0.350	1.000	0.408	0.193	0.416	0.008	0.197	2.572
mmu-miR-5119	0.306	0.545	0.546	0.298	0.510	0.112	0.250	2.568
mmu-miR-1839-5p	0.286	1.000	0.326	0.316	0.336	0.033	0.270	2.566
mmu-miR-6239	0.258	1.000	0.515	0.115	0.524	0.035	0.110	2.557
mmu-miR-324-3p	0.350	1.000	0.369	0.193	0.351	0.092	0.197	2.551
mmu-miR-99b-3p	0.234	1.000	0.500	0.135	0.488	0.079	0.110	2.547
mmu-miR-34b-3p	0.407	0.727	0.353	0.272	0.346	0.167	0.274	2.546
mmu-miR-206-3p	0.306	1.000	0.425	0.162	0.448	0.030	0.173	2.545
mmu-miR-690	0.350	1.000	0.359	0.193	0.359	0.087	0.197	2.545
mmu-miR-421-5p	0.469	0.455	0.288	0.384	0.393	0.161	0.393	2.542
mmu-miR-1249-3p	0.350	1.000	0.383	0.193	0.399	0.019	0.197	2.540
mmu-miR-674-3p	0.206	1.000	0.481	0.199	0.456	0.033	0.162	2.537
mmu-let-7i-3p	0.407	1.000	0.251	0.272	0.274	0.056	0.274	2.534
mmu-miR-488-3p	0.356	1.000	0.336	0.228	0.343	0.029	0.240	2.532
mmu-miR-873a-3p	0.234	1.000	0.392	0.274	0.352	0.047	0.223	2.522
mmu-miR-3068-3p	0.234	1.000	0.540	0.057	0.509	0.119	0.060	2.518
mmu-miR-329-5p	0.234	1.000	0.360	0.233	0.394	0.101	0.197	2.518
mmu-miR-669b-5p	0.356	0.818	0.423	0.228	0.415	0.035	0.240	2.515
mmu-miR-3087-3p	0.294	0.364	0.480	0.298	0.502	0.327	0.250	2.514
mmu-miR-598-3p	0.407	0.545	0.399	0.272	0.413	0.197	0.274	2.508
mmu-miR-434-3p	0.234	1.000	0.441	0.211	0.418	0.020	0.174	2.498
mmu-miR-200c-5p	0.407	0.364	0.366	0.272	0.580	0.233	0.274	2.496
mmu-miR-411-3p	0.407	1.000	0.254	0.272	0.247	0.040	0.274	2.494
mmu-miR-505-3p	0.294	0.818	0.468	0.186	0.450	0.127	0.150	2.492
mmu-miR-17-3p	0.258	1.000	0.458	0.102	0.442	0.122	0.110	2.492
mmu-miR-152-3p	0.350	0.455	0.468	0.298	0.486	0.181	0.250	2.487
mmu-miR-30c-2-3p	0.350	1.000	0.333	0.211	0.293	0.100	0.197	2.483
mmu-miR-5121	0.313	0.545	0.422	0.379	0.409	0.098	0.313	2.479
mmu-miR-497a-5p	0.294	1.000	0.451	0.122	0.434	0.051	0.126	2.477
mmu-miR-337-3p	0.356	1.000	0.268	0.228	0.274	0.110	0.240	2.475
mmu-let-7f-5p	0.294	1.000	0.392	0.183	0.367	0.086	0.147	2.469
mmu-miR-107-3p	0.294	1.000	0.453	0.122	0.420	0.052	0.126	2.466
mmu-miR-32-5p	0.306	1.000	0.315	0.233	0.338	0.075	0.197	2.466
mmu-let-7g-3p	0.206	1.000	0.507	0.115	0.492	0.051	0.091	2.462
mmu-miR-23b-3p	0.350	1.000	0.343	0.193	0.345	0.032	0.197	2.460
mmu-miR-1843a-3p	0.350	1.000	0.353	0.193	0.341	0.025	0.197	2.458
mmu-miR-30a-5p	0.306	0.545	0.612	0.162	0.574	0.085	0.173	2.457
mmu-miR-140-5p	0.356	1.000	0.316	0.228	0.304	0.012	0.240	2.456
mmu-miR-106b-3p	0.258	1.000	0.441	0.102	0.442	0.102	0.110	2.456
mmu-miR-224-5p	0.356	0.545	0.474	0.228	0.494	0.116	0.240	2.453
mmu-let-7g-5p	0.258	0.364	0.721	0.233	0.666	0.011	0.197	2.450

Attribute	Weight_Info Gain Ratio	Weight_Rule	Weight_Chi Squared	Weight_Gini Index	Weight_Uncertainty	Weight_Relief	Weight_Info Gain	Sum_weight
mmu-miR-664-3p	0.234	1.000	0.491	0.115	0.457	0.058	0.091	2.446
mmu-miR-299a-5p	0.294	1.000	0.408	0.135	0.407	0.071	0.126	2.441
mmu-miR-431-5p	0.306	0.545	0.425	0.316	0.433	0.136	0.270	2.431
mmu-miR-669c-5p	0.356	1.000	0.228	0.228	0.201	0.176	0.240	2.429
mmu-miR-26a-1-3p	0.258	0.636	0.536	0.199	0.574	0.061	0.162	2.426
mmu-miR-497a-3p	0.362	0.545	0.317	0.412	0.355	0.078	0.354	2.425
mmu-miR-126a-3p	0.206	1.000	0.435	0.179	0.441	0.022	0.143	2.425
mmu-miR-376b-3p	0.234	1.000	0.376	0.211	0.333	0.094	0.174	2.421
mmu-miR-6986-5p	0.206	0.909	0.537	0.101	0.512	0.073	0.083	2.420
mmu-miR-676-5p	0.234	1.000	0.468	0.135	0.451	0.021	0.110	2.419
mmu-miR-666-5p	0.234	1.000	0.430	0.107	0.518	0.042	0.085	2.416
mmu-miR-129-5p	0.306	1.000	0.366	0.162	0.360	0.044	0.173	2.411
mmu-miR-669f-3p	0.206	0.545	0.595	0.199	0.624	0.079	0.162	2.410
mmu-let-7k	0.234	1.000	0.369	0.233	0.346	0.028	0.197	2.407
mmu-miR-124-3p	0.407	1.000	0.198	0.272	0.213	0.042	0.274	2.407
mmu-miR-3059-3p	0.258	1.000	0.376	0.199	0.352	0.055	0.162	2.402
mmu-miR-30e-3p	0.350	1.000	0.320	0.193	0.328	0.012	0.197	2.399
mmu-miR-361-3p	0.350	0.545	0.359	0.298	0.375	0.222	0.250	2.398
mmu-miR-216a-5p	0.220	0.909	0.399	0.233	0.406	0.027	0.197	2.391
mmu-miR-320-3p	0.234	1.000	0.441	0.131	0.411	0.067	0.105	2.389
mmu-miR-27b-3p	0.258	1.000	0.474	0.102	0.438	0.003	0.110	2.386
mmu-miR-24-3p	0.258	1.000	0.435	0.102	0.437	0.042	0.110	2.384
mmu-miR-190a-5p	0.350	1.000	0.295	0.193	0.327	0.019	0.197	2.381
mmu-miR-346-5p	0.350	0.636	0.408	0.211	0.398	0.181	0.197	2.381
mmu-miR-139-3p	0.306	0.455	0.589	0.183	0.576	0.094	0.173	2.376
mmu-miR-503-5p	0.306	1.000	0.349	0.162	0.338	0.045	0.173	2.374
mmu-miR-154-3p	0.234	0.909	0.491	0.072	0.475	0.132	0.060	2.373
mmu-miR-544-5p	0.234	1.000	0.261	0.274	0.236	0.143	0.223	2.370
mmu-miR-5124a	0.306	0.909	0.296	0.162	0.520	0.002	0.173	2.368
mmu-miR-30e-5p	0.306	1.000	0.293	0.233	0.275	0.059	0.197	2.364
mmu-miR-34c-5p	0.306	1.000	0.336	0.162	0.379	0.008	0.173	2.364
mmu-miR-138-1-3p	0.306	1.000	0.336	0.162	0.341	0.041	0.173	2.359
mmu-miR-138-5p	0.294	1.000	0.360	0.122	0.394	0.062	0.126	2.356
mmu-miR-7075-3p	0.350	0.455	0.307	0.298	0.373	0.323	0.250	2.355
mmu-miR-130b-5p	0.234	0.909	0.441	0.131	0.461	0.065	0.105	2.346
mmu-miR-199a-5p	0.350	1.000	0.300	0.193	0.298	0.005	0.197	2.342
mmu-miR-384-3p	0.350	1.000	0.258	0.193	0.291	0.052	0.197	2.341
mmu-miR-8103	0.258	0.455	0.455	0.283	0.519	0.140	0.232	2.340
mmu-miR-191-5p	0.306	1.000	0.359	0.162	0.338	0.001	0.173	2.339
mmu-miR-125a-5p	0.294	1.000	0.376	0.122	0.357	0.052	0.126	2.326
mmu-miR-26a-5p	0.356	1.000	0.261	0.228	0.239	0.003	0.240	2.326
mmu-miR-296-5p	0.258	1.000	0.326	0.233	0.306	0.002	0.197	2.323
mmu-miR-1a-3p	0.234	1.000	0.366	0.162	0.328	0.098	0.135	2.323
mmu-miR-495-3p	0.206	1.000	0.408	0.186	0.366	0.003	0.150	2.320
mmu-miR-6911-3p	0.234	0.636	0.388	0.274	0.505	0.051	0.223	2.311
mmu-miR-369-5p	0.206	1.000	0.408	0.107	0.393	0.111	0.085	2.311
mmu-miR-149-5p	0.258	1.000	0.376	0.102	0.338	0.125	0.110	2.309
mmu-miR-153-5p	0.294	1.000	0.376	0.122	0.357	0.035	0.126	2.308
mmu-miR-666-3p	0.234	1.000	0.385	0.072	0.377	0.180	0.060	2.308
mmu-miR-106b-5p	0.234	0.818	0.495	0.114	0.474	0.082	0.091	2.308
mmu-miR-467d-5p	0.234	0.818	0.369	0.274	0.359	0.031	0.223	2.307
mmu-miR-874-5p	0.220	0.727	0.444	0.233	0.476	0.007	0.197	2.305
mmu-miR-344d-3p	0.258	1.000	0.359	0.102	0.369	0.106	0.110	2.304
mmu-miR-7015-3p	0.206	0.636	0.629	0.052	0.675	0.053	0.052	2.304
mmu-miR-296-3p	0.258	1.000	0.360	0.107	0.388	0.077	0.110	2.300
mmu-miR-9b-3p	0.234	1.000	0.432	0.057	0.439	0.075	0.060	2.296
mmu-miR-136-5p	0.350	0.727	0.261	0.211	0.388	0.158	0.197	2.290
mmu-miR-7080-5p	0.258	1.000	0.365	0.102	0.389	0.066	0.110	2.289
mmu-miR-26b-3p	0.234	0.636	0.399	0.179	0.533	0.165	0.143	2.288
mmu-miR-668-3p	0.306	0.364	0.507	0.283	0.465	0.128	0.232	2.284
mmu-miR-412-5p	0.234	1.000	0.343	0.186	0.327	0.044	0.150	2.284
mmu-miR-331-3p	0.206	1.000	0.418	0.107	0.422	0.046	0.084	2.283
mmu-miR-3057-5p	0.294	0.455	0.355	0.274	0.432	0.251	0.223	2.283
mmu-miR-335-3p	0.234	1.000	0.330	0.211	0.326	0.001	0.174	2.276
mmu-miR-433-3p	0.234	1.000	0.359	0.135	0.326	0.110	0.110	2.275
mmu-miR-128-2-5p	0.306	0.636	0.496	0.183	0.473	0.006	0.173	2.274
mmu-miR-7055-5p	0.409	0.364	0.288	0.302	0.514	0.084	0.313	2.273
mmu-miR-322-3p	0.234	1.000	0.408	0.107	0.398	0.041	0.085	2.272
mmu-miR-3068-5p	0.234	1.000	0.336	0.135	0.335	0.121	0.110	2.272
mmu-miR-370-5p	0.356	0.909	0.277	0.228	0.258	0.004	0.240	2.272
mmu-miR-1195	0.234	0.909	0.333	0.115	0.370	0.217	0.091	2.269
mmu-miR-712-3p	0.234	0.455	0.392	0.233	0.625	0.130	0.197	2.267
mmu-miR-879-3p	0.294	1.000	0.303	0.122	0.289	0.132	0.126	2.266
mmu-miR-501-3p	0.258	1.000	0.343	0.107	0.351	0.095	0.110	2.264
mmu-miR-345-3p	0.258	1.000	0.353	0.102	0.346	0.092	0.110	2.261
mmu-miR-466h-5p	0.350	0.364	0.438	0.211	0.494	0.207	0.197	2.260
mmu-miR-200b-5p	0.362	0.545	0.240	0.412	0.283	0.062	0.354	2.259
mmu-miR-27a-3p	0.206	1.000	0.408	0.101	0.408	0.052	0.083	2.258
mmu-miR-1968-5p	0.306	0.545	0.426	0.162	0.552	0.092	0.173	2.257
mmu-miR-138-2-3p	0.306	1.000	0.227	0.162	0.253	0.136	0.173	2.256

Attribute	Weight_Info Gain Ratio	Weight_Rule	Weight_Chi Squared	Weight_Gini Index	Weight_Uncertainty	Weight_Relief	Weight_Info Gain	Sum_weight
mmu-miR-100-5p	0.234	1.000	0.343	0.186	0.312	0.028	0.150	2.253
mmu-miR-5099	0.350	1.000	0.201	0.193	0.181	0.129	0.197	2.251
mmu-miR-1194	0.356	0.364	0.393	0.233	0.503	0.159	0.240	2.247
mmu-miR-665-3p	0.258	1.000	0.343	0.131	0.320	0.081	0.110	2.242
mmu-miR-181c-3p	0.258	1.000	0.310	0.162	0.289	0.085	0.135	2.239
mmu-miR-378a-3p	0.258	1.000	0.369	0.102	0.339	0.061	0.110	2.239
mmu-miR-92b-5p	0.234	1.000	0.343	0.131	0.373	0.052	0.105	2.238
mmu-miR-1291	0.206	1.000	0.376	0.047	0.391	0.158	0.052	2.229
mmu-miR-709	0.306	1.000	0.269	0.179	0.273	0.026	0.173	2.226
mmu-miR-16-5p	0.294	1.000	0.336	0.122	0.333	0.008	0.126	2.218
mmu-miR-148a-5p	0.306	0.455	0.494	0.162	0.611	0.016	0.173	2.217
mmu-miR-503-3p	0.306	1.000	0.270	0.162	0.245	0.056	0.173	2.212
mmu-miR-181c-5p	0.206	1.000	0.402	0.062	0.377	0.112	0.052	2.211
mmu-let-7e-5p	0.407	0.364	0.425	0.272	0.404	0.064	0.274	2.210
mmu-miR-1298-3p	0.294	1.000	0.320	0.135	0.296	0.036	0.126	2.207
mmu-miR-300-3p	0.258	1.000	0.313	0.102	0.313	0.108	0.110	2.204
mmu-miR-129-1-3p	0.306	1.000	0.287	0.162	0.266	0.010	0.173	2.203
mmu-miR-31-3p	0.356	0.364	0.408	0.233	0.486	0.115	0.240	2.201
mmu-miR-135a-5p	0.409	0.364	0.343	0.302	0.358	0.113	0.313	2.201
mmu-miR-339-5p	0.258	1.000	0.284	0.186	0.276	0.045	0.150	2.200
mmu-miR-26b-5p	0.234	1.000	0.228	0.233	0.228	0.076	0.197	2.196
mmu-let-7f-1-3p	0.294	0.727	0.305	0.233	0.332	0.105	0.197	2.194
mmu-miR-344c-3p	0.258	0.818	0.418	0.102	0.400	0.087	0.110	2.194
mmu-miR-7066-5p	0.258	0.455	0.563	0.102	0.558	0.144	0.110	2.190
mmu-miR-532-3p	0.258	1.000	0.269	0.102	0.280	0.171	0.110	2.190
mmu-miR-574-5p	0.234	1.000	0.385	0.072	0.366	0.071	0.060	2.188
mmu-miR-182-5p	0.407	0.455	0.270	0.272	0.340	0.168	0.274	2.186
mmu-miR-29c-5p	0.294	0.455	0.468	0.233	0.461	0.078	0.197	2.186
mmu-miR-551b-3p	0.350	0.909	0.231	0.193	0.236	0.062	0.197	2.178
mmu-miR-485-3p	0.234	1.000	0.343	0.107	0.365	0.044	0.084	2.176
mmu-miR-1949	0.206	0.818	0.441	0.075	0.429	0.147	0.060	2.176
mmu-miR-204-5p	0.294	0.909	0.324	0.122	0.375	0.026	0.126	2.176
mmu-miR-362-5p	0.407	0.364	0.369	0.272	0.366	0.121	0.274	2.174
mmu-miR-187-5p	0.258	1.000	0.297	0.102	0.291	0.112	0.110	2.169
mmu-miR-6769b-3p	0.356	0.364	0.324	0.233	0.472	0.181	0.240	2.169
mmu-miR-98-5p	0.234	1.000	0.316	0.135	0.277	0.095	0.110	2.168
mmu-miR-449a-5p	0.234	0.727	0.441	0.131	0.445	0.082	0.105	2.166
mmu-miR-874-3p	0.294	1.000	0.303	0.122	0.281	0.039	0.126	2.165
mmu-miR-677-5p	0.294	0.818	0.343	0.186	0.360	0.011	0.150	2.162
mmu-miR-33-3p	0.356	0.545	0.365	0.228	0.389	0.036	0.240	2.159
mmu-miR-33-5p	0.258	0.364	0.648	0.131	0.626	0.021	0.110	2.159
mmu-miR-181d-3p	0.278	0.000	0.454	0.298	0.646	0.231	0.250	2.158
mmu-miR-485-5p	0.350	1.000	0.162	0.193	0.131	0.120	0.197	2.153
mmu-miR-540-5p	0.294	1.000	0.244	0.122	0.237	0.129	0.126	2.151
mmu-let-7a-1-3p	0.234	1.000	0.336	0.101	0.321	0.075	0.083	2.151
mmu-miR-434-5p	0.350	0.455	0.310	0.298	0.312	0.172	0.250	2.146
mmu-miR-411-5p	0.258	1.000	0.277	0.102	0.272	0.124	0.110	2.144
mmu-miR-5113	0.258	0.636	0.383	0.183	0.435	0.097	0.147	2.139
mmu-miR-344-3p	0.350	1.000	0.195	0.193	0.181	0.023	0.197	2.139
mmu-miR-1224-5p	0.306	0.636	0.284	0.283	0.349	0.048	0.232	2.138
mmu-miR-210-3p	0.306	0.818	0.320	0.162	0.301	0.058	0.173	2.138
mmu-miR-483-5p	0.294	1.000	0.277	0.135	0.298	0.007	0.126	2.137
mmu-miR-21a-5p	0.356	0.273	0.484	0.270	0.469	0.045	0.240	2.136
mmu-miR-329-3p	0.350	1.000	0.165	0.193	0.156	0.073	0.197	2.134
mmu-miR-34b-5p	0.350	0.545	0.332	0.199	0.359	0.150	0.197	2.131
mmu-miR-5615-3p	0.350	0.273	0.288	0.193	0.544	0.287	0.197	2.131
mmu-miR-152-5p	0.356	0.364	0.402	0.228	0.416	0.123	0.240	2.128
mmu-miR-376c-5p	0.258	1.000	0.302	0.102	0.326	0.027	0.110	2.125
mmu-miR-872-5p	0.294	1.000	0.261	0.122	0.256	0.055	0.126	2.113
mmu-miR-144-3p	0.350	0.545	0.211	0.283	0.273	0.219	0.232	2.113
mmu-miR-345-5p	0.294	0.818	0.343	0.186	0.313	0.008	0.150	2.111
mmu-miR-505-5p	0.306	0.636	0.333	0.162	0.326	0.174	0.173	2.110
mmu-miR-873a-5p	0.294	0.909	0.300	0.122	0.270	0.089	0.126	2.109
mmu-miR-10b-5p	0.306	0.818	0.294	0.162	0.318	0.035	0.173	2.106
mmu-let-7b-3p	0.258	1.000	0.302	0.107	0.318	0.008	0.110	2.103
mmu-miR-467a-3p	0.356	0.273	0.343	0.228	0.522	0.133	0.240	2.094
mmu-miR-143-3p	0.258	1.000	0.251	0.107	0.261	0.105	0.110	2.092
mmu-miR-128-1-5p	0.234	1.000	0.224	0.162	0.239	0.095	0.135	2.089
mmu-miR-142a-3p	0.409	0.364	0.310	0.302	0.283	0.108	0.313	2.089
mmu-miR-6994-3p	0.294	0.364	0.376	0.186	0.495	0.219	0.150	2.083
mmu-miR-339-3p	0.234	0.636	0.432	0.115	0.514	0.057	0.091	2.079
mmu-miR-425-3p	0.234	1.000	0.258	0.179	0.256	0.008	0.143	2.078
mmu-let-7j	0.350	0.273	0.510	0.193	0.498	0.056	0.197	2.077
mmu-miR-582-5p	0.306	0.545	0.415	0.162	0.420	0.055	0.173	2.076
mmu-miR-487b-3p	0.234	1.000	0.266	0.135	0.290	0.039	0.110	2.075
mmu-let-7c-1-3p	0.206	0.909	0.385	0.050	0.358	0.111	0.052	2.072
mmu-miR-365-3p	0.294	0.364	0.500	0.179	0.473	0.115	0.143	2.068
mmu-miR-409-5p	0.234	1.000	0.277	0.135	0.260	0.048	0.110	2.064
mmu-miR-181d-5p	0.234	1.000	0.303	0.058	0.313	0.096	0.060	2.064
mmu-miR-125b-5p	0.234	1.000	0.300	0.115	0.319	0.004	0.091	2.063

Attribute	Weight_Info Gain Ratio	Weight_Rule	Weight_Chi Squared	Weight_Gini Index	Weight_Uncertainty	Weight_Relief	Weight_Info Gain	Sum_weight
mmu-miR-193a-5p	0.206	0.818	0.400	0.075	0.399	0.096	0.060	2.054
mmu-miR-187-3p	0.234	1.000	0.303	0.115	0.293	0.018	0.091	2.054
mmu-miR-145a-3p	0.234	1.000	0.293	0.075	0.318	0.072	0.060	2.052
mmu-miR-199b-5p	0.234	0.364	0.504	0.183	0.565	0.054	0.147	2.050
mmu-miR-466h-3p	0.220	0.545	0.350	0.233	0.470	0.034	0.197	2.050
mmu-miR-323-5p	0.234	0.727	0.399	0.107	0.395	0.104	0.084	2.050
mmu-miR-1193-3p	0.234	0.727	0.392	0.131	0.398	0.058	0.105	2.045
mmu-miR-299a-3p	0.234	1.000	0.303	0.057	0.281	0.105	0.060	2.040
mmu-miR-148b-3p	0.234	1.000	0.264	0.115	0.278	0.058	0.091	2.039
mmu-miR-3970	0.234	1.000	0.238	0.115	0.229	0.133	0.091	2.038
mmu-miR-6964-3p	0.258	0.273	0.507	0.199	0.518	0.118	0.162	2.035
mmu-miR-34a-5p	0.294	1.000	0.218	0.122	0.214	0.058	0.126	2.032
mmu-miR-700-3p	0.234	1.000	0.277	0.115	0.254	0.061	0.091	2.032
mmu-miR-204-3p	0.294	1.000	0.202	0.122	0.240	0.045	0.126	2.029
mmu-miR-19a-3p	0.206	1.000	0.196	0.162	0.206	0.123	0.135	2.028
mmu-miR-205-5p	0.294	0.455	0.441	0.183	0.433	0.075	0.147	2.027
mmu-miR-155-5p	0.306	0.818	0.269	0.186	0.273	0.000	0.173	2.025
mmu-miR-136-3p	0.350	0.455	0.418	0.193	0.392	0.020	0.197	2.025
mmu-miR-30c-5p	0.258	1.000	0.221	0.102	0.212	0.119	0.110	2.023
mmu-miR-191-3p	0.306	0.182	0.606	0.162	0.561	0.031	0.173	2.020
mmu-miR-27a-5p	0.206	0.455	0.455	0.131	0.601	0.051	0.105	2.004
mmu-miR-466b-3p	0.206	0.909	0.376	0.052	0.367	0.040	0.052	2.001
mmu-miR-582-3p	0.234	1.000	0.270	0.075	0.252	0.082	0.060	1.974
mmu-miR-450a-5p	0.407	0.364	0.297	0.272	0.353	0.005	0.274	1.972
mmu-miR-19b-3p	0.356	0.273	0.280	0.316	0.285	0.191	0.270	1.971
mmu-miR-183-3p	0.407	0.364	0.189	0.272	0.344	0.118	0.274	1.969
mmu-miR-185-5p	0.234	1.000	0.198	0.162	0.189	0.050	0.135	1.969
mmu-miR-185-3p	0.294	1.000	0.146	0.122	0.133	0.148	0.126	1.968
mmu-miR-500-3p	0.294	0.818	0.251	0.135	0.254	0.087	0.126	1.965
mmu-miR-137-5p	0.306	0.818	0.211	0.162	0.215	0.078	0.173	1.963
mmu-miR-218-1-3p	0.356	0.273	0.255	0.228	0.451	0.160	0.240	1.962
mmu-miR-342-3p	0.234	1.000	0.244	0.057	0.253	0.110	0.060	1.958
mmu-miR-423-5p	0.234	1.000	0.185	0.186	0.158	0.045	0.150	1.958
mmu-miR-193b-5p	0.234	0.818	0.303	0.162	0.281	0.022	0.135	1.955
mmu-miR-8114	0.294	0.455	0.355	0.131	0.561	0.034	0.126	1.955
mmu-miR-1981-3p	0.234	0.818	0.310	0.131	0.300	0.052	0.105	1.951
mmu-miR-499-5p	0.234	0.909	0.310	0.072	0.283	0.082	0.060	1.950
mmu-miR-380-5p	0.234	1.000	0.228	0.114	0.251	0.030	0.091	1.948
mmu-miR-3095-3p	0.258	0.727	0.326	0.102	0.336	0.089	0.110	1.948
mmu-miR-30d-5p	0.234	1.000	0.244	0.057	0.231	0.117	0.060	1.943
mmu-miR-5101	0.306	0.273	0.320	0.162	0.523	0.183	0.173	1.939
mmu-miR-342-5p	0.306	0.545	0.303	0.233	0.306	0.046	0.197	1.938
mmu-miR-3064-5p	0.294	0.636	0.343	0.122	0.379	0.037	0.126	1.936
mmu-miR-744-5p	0.206	1.000	0.270	0.114	0.252	0.002	0.091	1.935
mmu-miR-7026-5p	0.306	0.273	0.320	0.162	0.504	0.195	0.173	1.932
mmu-miR-7073-5p	0.258	0.455	0.404	0.199	0.412	0.041	0.162	1.931
mmu-miR-696	0.234	0.364	0.458	0.101	0.581	0.110	0.083	1.930
mmu-miR-34a-3p	0.208	0.455	0.323	0.211	0.484	0.075	0.174	1.930
mmu-miR-6988-3p	0.350	0.273	0.393	0.193	0.508	0.009	0.197	1.923
mmu-miR-6946-5p	0.356	0.364	0.221	0.228	0.358	0.151	0.240	1.918
mmu-miR-211-5p	0.206	0.727	0.369	0.101	0.355	0.075	0.083	1.916
mmu-miR-219a-5p	0.350	0.727	0.210	0.193	0.234	0.003	0.197	1.914
mmu-miR-760-5p	0.258	0.364	0.474	0.107	0.580	0.014	0.110	1.907
mmu-miR-1912-3p	0.350	0.273	0.320	0.193	0.542	0.031	0.197	1.905
mmu-miR-378a-5p	0.234	1.000	0.244	0.072	0.248	0.044	0.060	1.901
mmu-miR-744-3p	0.220	0.455	0.324	0.233	0.438	0.032	0.197	1.899
mmu-miR-7689-3p	0.234	1.000	0.211	0.115	0.213	0.035	0.091	1.899
mmu-miR-671-3p	0.234	1.000	0.205	0.107	0.189	0.076	0.085	1.895
mmu-miR-338-5p	0.294	0.364	0.474	0.135	0.483	0.015	0.126	1.891
mmu-miR-3470a	0.350	0.273	0.435	0.193	0.431	0.008	0.197	1.886
mmu-miR-3572-3p	0.206	0.455	0.366	0.135	0.481	0.131	0.110	1.885
mmu-miR-3060-3p	0.258	0.455	0.264	0.283	0.295	0.097	0.232	1.883
mmu-miR-543-3p	0.234	1.000	0.231	0.058	0.231	0.068	0.060	1.881
mmu-miR-486a-3p	0.234	0.364	0.392	0.135	0.576	0.067	0.110	1.879
mmu-miR-25-5p	0.234	0.909	0.200	0.135	0.262	0.026	0.110	1.878
mmu-miR-151-5p	0.258	1.000	0.205	0.102	0.197	0.002	0.110	1.874
mmu-miR-7a-1-3p	0.234	1.000	0.205	0.057	0.185	0.134	0.060	1.873
mmu-miR-92a-3p	0.234	1.000	0.228	0.057	0.206	0.089	0.060	1.872
mmu-miR-667-5p	0.206	0.909	0.205	0.162	0.211	0.043	0.135	1.871
mmu-miR-141-5p	0.409	0.364	0.185	0.302	0.285	0.011	0.313	1.868
mmu-miR-200c-3p	0.409	0.364	0.185	0.302	0.285	0.010	0.313	1.867
mmu-miR-3069-3p	0.234	0.455	0.392	0.114	0.558	0.024	0.091	1.867
mmu-miR-7a-2-3p	0.258	0.545	0.325	0.102	0.376	0.145	0.110	1.862
mmu-miR-466d-3p	0.234	0.909	0.270	0.075	0.258	0.052	0.060	1.860
mmu-miR-1188-5p	0.206	0.818	0.310	0.062	0.302	0.107	0.052	1.857
mmu-miR-706	0.258	1.000	0.139	0.131	0.135	0.082	0.110	1.855
mmu-miR-10a-5p	0.350	0.364	0.291	0.199	0.282	0.172	0.197	1.854
mmu-miR-146b-5p	0.234	1.000	0.205	0.072	0.193	0.087	0.060	1.850
mmu-miR-7093-3p	0.350	0.273	0.324	0.193	0.438	0.075	0.197	1.849
mmu-miR-669d-5p	0.350	0.273	0.261	0.274	0.401	0.059	0.223	1.839

Attribute	Weight_Info Gain Ratio	Weight_Rule	Weight_Chi Squared	Weight_Gini Index	Weight_Uncertainty	Weight_Relief	Weight_Info Gain	Sum_weight
mmu-miR-133a-3p	0.294	0.455	0.359	0.183	0.352	0.046	0.147	1.836
mmu-miR-30f	0.206	0.364	0.515	0.075	0.522	0.093	0.060	1.835
mmu-miR-6540-3p	0.294	0.182	0.422	0.122	0.675	0.014	0.126	1.834
mmu-miR-3470b	0.206	1.000	0.205	0.047	0.189	0.134	0.052	1.833
mmu-miR-450b-3p	0.294	0.455	0.359	0.122	0.433	0.042	0.126	1.831
mmu-miR-2137	0.306	0.364	0.284	0.283	0.283	0.079	0.232	1.831
mmu-miR-379-5p	0.294	1.000	0.097	0.131	0.095	0.085	0.126	1.827
mmu-miR-3083b-5p	0.234	0.636	0.363	0.057	0.460	0.017	0.060	1.827
mmu-miR-6516-3p	0.356	0.364	0.297	0.228	0.319	0.022	0.240	1.825
mmu-miR-126a-5p	0.206	1.000	0.198	0.075	0.198	0.084	0.060	1.822
mmu-miR-486a-5p	0.234	1.000	0.178	0.057	0.168	0.124	0.060	1.820
mmu-miR-148b-5p	0.306	0.909	0.132	0.162	0.131	0.004	0.173	1.818
mmu-miR-363-3p	0.206	0.364	0.408	0.162	0.499	0.037	0.135	1.811
mmu-miR-491-5p	0.306	0.636	0.254	0.183	0.253	0.006	0.173	1.811
mmu-miR-107-5p	0.234	0.364	0.355	0.135	0.561	0.050	0.110	1.809
mmu-miR-323-3p	0.234	1.000	0.179	0.057	0.181	0.081	0.060	1.792
mmu-miR-450b-5p	0.206	0.364	0.425	0.135	0.513	0.033	0.110	1.786
mmu-miR-6899-3p	0.294	0.182	0.441	0.122	0.525	0.095	0.126	1.785
mmu-let-7e-3p	0.306	0.364	0.270	0.233	0.245	0.168	0.197	1.784
mmu-miR-1251-5p	0.234	0.818	0.231	0.131	0.261	0.003	0.105	1.783
mmu-miR-467d-3p	0.306	0.364	0.330	0.162	0.386	0.058	0.173	1.778
mmu-miR-375-3p	0.234	1.000	0.192	0.057	0.232	0.001	0.060	1.776
mmu-miR-3084-3p	0.234	0.455	0.357	0.131	0.484	0.010	0.105	1.775
mmu-miR-186-3p	0.234	1.000	0.162	0.114	0.140	0.029	0.091	1.770
mmu-miR-3099-3p	0.306	0.636	0.228	0.162	0.206	0.053	0.173	1.764
mmu-miR-376a-5p	0.258	0.455	0.376	0.131	0.398	0.033	0.110	1.760
mmu-miR-219a-2-3p	0.234	1.000	0.165	0.057	0.156	0.088	0.060	1.760
mmu-miR-301a-5p	0.206	0.727	0.293	0.075	0.347	0.051	0.060	1.760
mmu-miR-181a-5p	0.306	0.545	0.244	0.199	0.221	0.070	0.173	1.759
mmu-miR-6944-3p	0.258	0.636	0.282	0.102	0.331	0.037	0.110	1.758
mmu-miR-488-5p	0.206	0.545	0.364	0.055	0.415	0.120	0.052	1.757
mmu-miR-5106	0.306	0.545	0.124	0.283	0.230	0.037	0.232	1.756
mmu-miR-30a-3p	0.294	0.636	0.264	0.122	0.273	0.038	0.126	1.753
mmu-miR-16-1-3p	0.294	0.636	0.268	0.122	0.287	0.013	0.126	1.745
mmu-miR-467e-5p	0.206	1.000	0.162	0.072	0.137	0.102	0.058	1.737
mmu-miR-542-5p	0.294	0.727	0.221	0.122	0.212	0.031	0.126	1.733
mmu-miR-328-3p	0.234	1.000	0.146	0.062	0.145	0.083	0.060	1.730
mmu-miR-28a-5p	0.258	0.455	0.359	0.131	0.369	0.043	0.110	1.724
mmu-miR-6538	0.294	0.182	0.425	0.122	0.455	0.119	0.126	1.722
mmu-miR-547-3p	0.306	0.273	0.310	0.162	0.378	0.121	0.173	1.722
mmu-miR-6418-3p	0.234	0.545	0.303	0.131	0.380	0.009	0.105	1.708
mmu-miR-18a-3p	0.234	0.273	0.395	0.072	0.569	0.104	0.060	1.706
mmu-miR-326-3p	0.350	0.273	0.318	0.199	0.361	0.006	0.197	1.704
mmu-miR-669a-5p	0.234	0.364	0.426	0.058	0.531	0.025	0.060	1.697
mmu-miR-17-5p	0.234	0.636	0.231	0.135	0.236	0.112	0.110	1.695
mmu-miR-467c-5p	0.356	0.273	0.273	0.233	0.291	0.030	0.240	1.695
mmu-miR-15b-3p	0.234	0.636	0.293	0.115	0.301	0.024	0.091	1.694
mmu-miR-7688-5p	0.206	0.364	0.357	0.135	0.499	0.023	0.110	1.694
mmu-miR-466m-3p	0.258	0.364	0.310	0.102	0.424	0.125	0.110	1.692
mmu-miR-673-3p	0.350	0.364	0.268	0.193	0.292	0.026	0.197	1.689
mmu-miR-297c-5p	0.206	0.182	0.376	0.186	0.533	0.051	0.150	1.683
mmu-miR-181a-1-3p	0.294	0.818	0.136	0.135	0.136	0.038	0.126	1.683
mmu-miR-222-5p	0.234	0.818	0.198	0.058	0.189	0.115	0.060	1.672
mmu-miR-12194-3p	0.306	0.273	0.310	0.162	0.424	0.016	0.173	1.663
mmu-miR-770-5p	0.306	0.273	0.366	0.162	0.369	0.014	0.173	1.662
mmu-miR-325-5p	0.206	0.273	0.422	0.072	0.596	0.034	0.058	1.660
mmu-miR-501-5p	0.234	0.727	0.224	0.115	0.232	0.035	0.091	1.658
mmu-miR-125b-1-3p	0.258	0.273	0.376	0.199	0.354	0.035	0.162	1.657
mmu-miR-214-3p	0.294	0.364	0.324	0.122	0.425	0.001	0.126	1.655
mmu-miR-325-3p	0.356	0.273	0.156	0.228	0.286	0.115	0.240	1.654
mmu-miR-30b-3p	0.206	1.000	0.103	0.072	0.094	0.117	0.058	1.650
mmu-miR-1964-3p	0.306	0.182	0.296	0.162	0.495	0.032	0.173	1.645
mmu-miR-701-5p	0.294	0.364	0.290	0.122	0.405	0.045	0.126	1.645
mmu-miR-6240	0.350	0.455	0.195	0.193	0.181	0.069	0.197	1.639
mmu-miR-7019-3p	0.258	0.364	0.256	0.162	0.385	0.072	0.135	1.633
mmu-miR-195a-3p	0.234	0.273	0.432	0.072	0.466	0.095	0.060	1.632
mmu-miR-3083-5p	0.234	0.636	0.236	0.057	0.232	0.176	0.060	1.631
mmu-miR-3968	0.234	0.364	0.359	0.075	0.403	0.133	0.060	1.628
mmu-let-7d-3p	0.234	1.000	0.113	0.062	0.106	0.051	0.060	1.626
mmu-miR-344f-5p	0.206	0.727	0.236	0.062	0.259	0.078	0.052	1.621
mmu-miR-322-5p	0.306	0.455	0.254	0.162	0.231	0.033	0.173	1.613
mmu-miR-3078-3p	0.234	0.545	0.277	0.107	0.333	0.030	0.085	1.612
mmu-miR-132-3p	0.306	0.182	0.349	0.233	0.307	0.035	0.197	1.610
mmu-miR-221-5p	0.206	0.909	0.146	0.072	0.127	0.089	0.058	1.607
mmu-miR-192-3p	0.234	0.545	0.251	0.107	0.327	0.058	0.084	1.607
mmu-miR-543-5p	0.206	0.545	0.252	0.186	0.256	0.001	0.150	1.596
mmu-miR-27b-5p	0.356	0.364	0.178	0.228	0.185	0.039	0.240	1.589
mmu-miR-1969	0.350	0.273	0.268	0.193	0.298	0.007	0.197	1.586
mmu-miR-7083-5p	0.350	0.000	0.185	0.193	0.420	0.235	0.197	1.580
mmu-miR-493-5p	0.234	0.364	0.325	0.135	0.367	0.040	0.110	1.576

Attribute	Weight_Info Gain Ratio	Weight_Rule	Weight_Chi Squared	Weight_Gini Index	Weight_Uncertainty	Weight_Relief	Weight_Info Gain	Sum_weight
mmu-miR-1198-3p	0.208	0.000	0.366	0.211	0.580	0.035	0.174	1.573
mmu-miR-195b	0.206	0.273	0.343	0.101	0.522	0.045	0.083	1.572
mmu-miR-494-3p	0.258	0.727	0.123	0.102	0.109	0.142	0.110	1.571
mmu-miR-3474	0.356	0.273	0.156	0.228	0.286	0.029	0.240	1.568
mmu-miR-1224-3p	0.294	0.273	0.336	0.122	0.343	0.074	0.126	1.567
mmu-miR-210-5p	0.350	0.000	0.185	0.193	0.420	0.222	0.197	1.567
mmu-miR-297a-5p	0.258	0.273	0.264	0.102	0.378	0.178	0.110	1.563
mmu-miR-3072-3p	0.234	0.455	0.293	0.057	0.359	0.094	0.060	1.552
mmu-miR-6966-5p	0.350	0.000	0.185	0.193	0.420	0.206	0.197	1.551
mmu-miR-3061-3p	0.206	0.091	0.392	0.114	0.596	0.060	0.091	1.550
mmu-miR-3473d	0.306	0.182	0.155	0.162	0.350	0.220	0.173	1.548
mmu-miR-487b-5p	0.234	0.818	0.129	0.057	0.113	0.136	0.060	1.547
mmu-miR-3552	0.306	0.182	0.290	0.162	0.419	0.011	0.173	1.542
mmu-miR-106a-5p	0.294	0.182	0.293	0.122	0.427	0.097	0.126	1.540
mmu-miR-592-5p	0.294	0.182	0.369	0.122	0.358	0.089	0.126	1.539
mmu-miR-7027-3p	0.356	0.273	0.166	0.228	0.219	0.052	0.240	1.534
mmu-miR-1197-3p	0.306	0.182	0.197	0.162	0.413	0.090	0.173	1.524
mmu-miR-483-3p	0.350	0.273	0.210	0.193	0.234	0.064	0.197	1.522
mmu-miR-3475-3p	0.206	0.455	0.231	0.162	0.297	0.035	0.135	1.521
mmu-miR-3471	0.258	0.273	0.264	0.102	0.378	0.137	0.110	1.521
mmu-miR-511-3p	0.258	0.273	0.297	0.102	0.440	0.039	0.110	1.519
mmu-miR-7220-3p	0.306	0.182	0.155	0.162	0.369	0.171	0.173	1.517
mmu-miR-466k	0.234	0.364	0.256	0.072	0.385	0.140	0.060	1.512
mmu-miR-669f-5p	0.306	0.182	0.197	0.162	0.395	0.088	0.173	1.504
mmu-miR-188-5p	0.234	0.455	0.277	0.057	0.345	0.066	0.060	1.493
mmu-miR-3082-5p	0.258	0.273	0.255	0.102	0.451	0.040	0.110	1.489
mmu-miR-450a-2-3p	0.206	0.182	0.366	0.107	0.481	0.063	0.084	1.489
mmu-miR-1947-5p	0.258	0.273	0.255	0.102	0.451	0.038	0.110	1.487
mmu-miR-669k-5p	0.258	0.273	0.258	0.102	0.362	0.124	0.110	1.486
mmu-miR-466g	0.234	0.364	0.231	0.075	0.297	0.223	0.060	1.485
mmu-miR-135b-3p	0.206	0.364	0.323	0.072	0.454	0.008	0.058	1.484
mmu-miR-504-5p	0.234	0.636	0.165	0.062	0.212	0.112	0.060	1.481
mmu-miR-6236	0.258	0.727	0.121	0.102	0.126	0.033	0.110	1.477
mmu-miR-714	0.294	0.182	0.255	0.122	0.469	0.028	0.126	1.475
mmu-miR-877-5p	0.258	0.455	0.209	0.131	0.282	0.028	0.110	1.472
mmu-miR-6966-3p	0.294	0.182	0.255	0.122	0.469	0.019	0.126	1.466
mmu-miR-182-3p	0.294	0.364	0.224	0.135	0.312	0.011	0.126	1.466
mmu-miR-6900-3p	0.258	0.182	0.323	0.102	0.454	0.037	0.110	1.466
mmu-miR-3069-5p	0.306	0.273	0.156	0.162	0.286	0.107	0.173	1.463
mmu-miR-6989-3p	0.206	0.364	0.265	0.075	0.316	0.173	0.060	1.459
mmu-miR-491-3p	0.234	0.182	0.255	0.057	0.451	0.219	0.060	1.458
mmu-miR-135a-1-3p	0.234	0.091	0.363	0.057	0.473	0.176	0.060	1.454
mmu-miR-6946-3p	0.258	0.364	0.209	0.102	0.282	0.129	0.110	1.453
mmu-miR-223-5p	0.234	0.273	0.296	0.072	0.495	0.021	0.060	1.451
mmu-miR-3076-5p	0.258	0.273	0.256	0.102	0.399	0.052	0.110	1.450
mmu-miR-3066-3p	0.306	0.182	0.155	0.162	0.350	0.121	0.173	1.450
mmu-miR-670-5p	0.258	0.273	0.244	0.102	0.372	0.089	0.110	1.448
mmu-miR-7070-3p	0.306	0.182	0.197	0.162	0.395	0.031	0.173	1.447
mmu-miR-764-3p	0.294	0.455	0.202	0.122	0.209	0.037	0.126	1.444
mmu-miR-12189-3p	0.306	0.182	0.197	0.162	0.395	0.024	0.173	1.439
mmu-miR-410-5p	0.258	0.273	0.313	0.107	0.306	0.070	0.110	1.437
mmu-miR-128-3p	0.258	0.455	0.238	0.131	0.224	0.021	0.110	1.436
mmu-miR-7008-3p	0.234	0.273	0.255	0.101	0.469	0.017	0.083	1.432
mmu-miR-135a-2-3p	0.294	0.364	0.212	0.186	0.222	0.004	0.150	1.430
mmu-miR-7226-3p	0.206	0.455	0.261	0.047	0.287	0.122	0.052	1.429
mmu-miR-5114	0.206	0.000	0.288	0.135	0.494	0.189	0.110	1.422
mmu-miR-1912-5p	0.294	0.182	0.264	0.122	0.378	0.055	0.126	1.420
mmu-miR-466a-3p	0.350	0.364	0.141	0.211	0.151	0.006	0.197	1.419
mmu-miR-511-5p	0.234	0.091	0.288	0.135	0.494	0.063	0.110	1.416
mmu-miR-1970	0.306	0.182	0.155	0.162	0.350	0.075	0.173	1.403
mmu-miR-466i-3p	0.258	0.182	0.244	0.102	0.437	0.059	0.110	1.392
mmu-miR-670-3p	0.206	0.364	0.277	0.058	0.334	0.096	0.052	1.386
mmu-miR-3076-3p	0.258	0.364	0.198	0.102	0.285	0.059	0.110	1.376
mmu-miR-133b-3p	0.306	0.273	0.166	0.162	0.202	0.093	0.173	1.375
mmu-miR-1839-3p	0.206	0.364	0.244	0.050	0.325	0.117	0.052	1.358
mmu-miR-150-3p	0.206	0.364	0.224	0.107	0.357	0.016	0.084	1.357
mmu-miR-3473f	0.258	0.091	0.255	0.102	0.451	0.082	0.110	1.349
mmu-miR-9b-5p	0.306	0.273	0.149	0.162	0.202	0.079	0.173	1.344
mmu-miR-466q	0.258	0.455	0.150	0.102	0.179	0.076	0.110	1.330
mmu-miR-3100-5p	0.306	0.182	0.155	0.162	0.350	0.001	0.173	1.330
mmu-miR-712-5p	0.206	0.545	0.139	0.055	0.163	0.168	0.052	1.329
mmu-miR-344f-3p	0.234	0.273	0.256	0.072	0.399	0.034	0.060	1.328
mmu-miR-16-2-3p	0.350	0.000	0.185	0.193	0.400	0.003	0.197	1.327
mmu-miR-455-3p	0.294	0.182	0.249	0.122	0.284	0.057	0.126	1.313
mmu-miR-350-5p	0.234	0.273	0.264	0.057	0.378	0.047	0.060	1.311
mmu-miR-3062-5p	0.206	0.182	0.244	0.050	0.437	0.138	0.052	1.309
mmu-miR-365-1-5p	0.206	0.182	0.261	0.114	0.388	0.064	0.091	1.305
mmu-miR-21a-3p	0.258	0.455	0.099	0.102	0.185	0.095	0.110	1.304
mmu-miR-184-3p	0.294	0.182	0.261	0.122	0.241	0.071	0.126	1.295
mmu-miR-3472	0.206	0.273	0.247	0.072	0.361	0.078	0.058	1.294

Attribute	Weight_Info Gain Ratio	Weight_Rule	Weight_Chi Squared	Weight_Gini Index	Weight_Uncertainty	Weight_Relief	Weight_Info Gain	Sum_weight
mmu-miR-679-5p	0.306	0.273	0.162	0.162	0.193	0.021	0.173	1.290
mmu-miR-1934-5p	0.234	0.364	0.211	0.075	0.243	0.095	0.060	1.283
mmu-miR-5134-5p	0.258	0.182	0.197	0.102	0.395	0.038	0.110	1.282
mmu-miR-18a-5p	0.258	0.091	0.197	0.102	0.395	0.124	0.110	1.278
mmu-miR-1943-5p	0.206	0.455	0.160	0.050	0.198	0.155	0.052	1.275
mmu-miR-351-3p	0.258	0.182	0.197	0.102	0.395	0.029	0.110	1.274
mmu-miR-802-3p	0.258	0.182	0.197	0.102	0.395	0.026	0.110	1.271
mmu-miR-7224-3p	0.234	0.364	0.226	0.057	0.278	0.049	0.060	1.267
mmu-miR-1306-5p	0.206	0.273	0.256	0.047	0.399	0.031	0.052	1.265
mmu-miR-7065-3p	0.234	0.273	0.280	0.072	0.304	0.036	0.060	1.258
mmu-miR-297b-3p	0.206	0.455	0.177	0.101	0.177	0.058	0.083	1.256
mmu-miR-3099-5p	0.258	0.091	0.197	0.102	0.395	0.091	0.110	1.245
mmu-miR-1264-5p	0.206	0.091	0.320	0.058	0.504	0.012	0.052	1.243
mmu-miR-3094-5p	0.206	0.273	0.132	0.050	0.194	0.325	0.052	1.232
mmu-miR-5118	0.306	0.182	0.113	0.162	0.214	0.079	0.173	1.228
mmu-miR-451a	0.206	0.182	0.244	0.050	0.437	0.055	0.052	1.226
mmu-miR-3066-5p	0.350	0.000	0.117	0.193	0.299	0.068	0.197	1.224
mmu-miR-669a-3-3p	0.234	0.091	0.244	0.057	0.437	0.086	0.060	1.208
mmu-miR-432	0.306	0.182	0.099	0.162	0.207	0.080	0.173	1.208
mmu-miR-5108	0.206	0.273	0.198	0.050	0.330	0.092	0.052	1.201
mmu-miR-181b-2-3p	0.234	0.182	0.258	0.057	0.339	0.071	0.060	1.201
mmu-miR-12193-5p	0.258	0.091	0.197	0.102	0.395	0.040	0.110	1.194
mmu-miR-381-5p	0.306	0.182	0.099	0.162	0.263	0.009	0.173	1.193
mmu-miR-8104	0.258	0.091	0.197	0.102	0.395	0.034	0.110	1.188
mmu-miR-299b-5p	0.206	0.636	0.039	0.114	0.034	0.065	0.091	1.186
mmu-miR-6481	0.234	0.182	0.221	0.058	0.358	0.069	0.060	1.181
mmu-miR-1306-3p	0.258	0.364	0.113	0.102	0.155	0.079	0.110	1.181
mmu-miR-7213-5p	0.206	0.182	0.198	0.050	0.330	0.158	0.052	1.176
mmu-miR-433-5p	0.294	0.182	0.148	0.122	0.255	0.049	0.126	1.175
mmu-miR-539-3p	0.234	0.273	0.188	0.072	0.311	0.037	0.060	1.175
mmu-miR-5128	0.294	0.182	0.159	0.122	0.224	0.068	0.126	1.174
mmu-miR-6940-3p	0.234	0.091	0.218	0.072	0.425	0.069	0.060	1.170
mmu-miR-7080-3p	0.206	0.364	0.125	0.135	0.194	0.031	0.110	1.166
mmu-miR-144-5p	0.294	0.182	0.139	0.122	0.200	0.100	0.126	1.162
mmu-miR-3098-5p	0.234	0.091	0.218	0.072	0.425	0.061	0.060	1.161
mmu-miR-190a-3p	0.206	0.364	0.162	0.075	0.196	0.095	0.060	1.158
mmu-miR-147-3p	0.206	0.182	0.197	0.047	0.395	0.072	0.052	1.152
mmu-miR-702-3p	0.234	0.273	0.178	0.101	0.246	0.036	0.083	1.151
mmu-miR-7220-5p	0.206	0.182	0.256	0.050	0.399	0.005	0.052	1.150
mmu-miR-188-3p	0.206	0.273	0.198	0.047	0.330	0.043	0.052	1.149
mmu-miR-7667-5p	0.234	0.091	0.244	0.057	0.454	0.007	0.060	1.147
mmu-miR-467a-5p	0.234	0.091	0.218	0.072	0.425	0.045	0.060	1.145
mmu-miR-344h-3p	0.258	0.091	0.146	0.102	0.334	0.104	0.110	1.145
mmu-miR-133a-5p	0.234	0.091	0.218	0.072	0.425	0.036	0.060	1.136
mmu-miR-6929-5p	0.294	0.182	0.120	0.122	0.238	0.053	0.126	1.134
mmu-miR-6896-5p	0.234	0.091	0.218	0.072	0.425	0.031	0.060	1.131
mmu-miR-193b-3p	0.206	0.273	0.225	0.047	0.311	0.017	0.052	1.131
mmu-miR-3058-3p	0.294	0.000	0.117	0.122	0.318	0.152	0.126	1.128
mmu-miR-7063-3p	0.206	0.091	0.197	0.047	0.395	0.137	0.052	1.126
mmu-miR-32-3p	0.234	0.182	0.224	0.057	0.344	0.024	0.060	1.124
mmu-miR-5617-5p	0.258	0.091	0.146	0.102	0.334	0.081	0.110	1.122
mmu-miR-6954-3p	0.206	0.182	0.197	0.050	0.413	0.020	0.052	1.121
mmu-miR-12187-3p	0.234	0.091	0.197	0.057	0.395	0.086	0.060	1.120
mmu-miR-215-5p	0.206	0.091	0.197	0.047	0.395	0.129	0.052	1.118
mmu-miR-331-5p	0.234	0.182	0.188	0.058	0.311	0.082	0.060	1.114
mmu-miR-7b-3p	0.206	0.091	0.221	0.058	0.358	0.120	0.052	1.105
mmu-miR-10b-3p	0.234	0.091	0.218	0.072	0.425	0.003	0.060	1.103
mmu-miR-489-3p	0.206	0.182	0.195	0.114	0.286	0.027	0.091	1.101
mmu-miR-7235-3p	0.206	0.182	0.198	0.047	0.330	0.085	0.052	1.100
mmu-miR-1941-5p	0.258	0.182	0.146	0.102	0.271	0.029	0.110	1.098
mmu-miR-6984-3p	0.234	0.182	0.188	0.058	0.311	0.060	0.060	1.092
mmu-miR-363-5p	0.294	0.000	0.117	0.122	0.299	0.133	0.126	1.091
mmu-miR-3074-1-3p	0.206	0.182	0.198	0.047	0.330	0.072	0.052	1.087
mmu-miR-92a-1-5p	0.294	0.000	0.117	0.122	0.299	0.129	0.126	1.087
mmu-miR-8109	0.258	0.091	0.146	0.102	0.334	0.045	0.110	1.086
mmu-miR-6948-3p	0.234	0.091	0.197	0.057	0.395	0.052	0.060	1.086
mmu-miR-7674-3p	0.294	0.000	0.117	0.122	0.299	0.125	0.126	1.083
mmu-miR-219a-1-3p	0.234	0.091	0.244	0.057	0.372	0.025	0.060	1.082
mmu-miR-504-3p	0.258	0.091	0.146	0.102	0.351	0.024	0.110	1.082
mmu-miR-3070-3p	0.206	0.273	0.156	0.072	0.286	0.025	0.058	1.076
mmu-miR-7225-5p	0.258	0.091	0.146	0.102	0.334	0.033	0.110	1.073
mmu-miR-7056-5p	0.234	0.091	0.221	0.058	0.371	0.038	0.060	1.073
mmu-miR-149-3p	0.294	0.000	0.117	0.122	0.299	0.115	0.126	1.073
mmu-miR-3065-5p	0.234	0.091	0.155	0.057	0.350	0.124	0.060	1.071
mmu-miR-100-3p	0.206	0.182	0.198	0.050	0.330	0.052	0.052	1.070
mmu-miR-6999-3p	0.294	0.000	0.117	0.122	0.299	0.111	0.126	1.069
mmu-miR-6943-3p	0.294	0.000	0.117	0.122	0.299	0.109	0.126	1.067
mmu-miR-1187	0.234	0.182	0.156	0.058	0.286	0.090	0.060	1.066
mmu-miR-3064-3p	0.206	0.000	0.218	0.072	0.445	0.065	0.058	1.064
mmu-miR-3072-5p	0.258	0.091	0.146	0.102	0.351	0.005	0.110	1.063

Attribute	Weight_Info Gain Ratio	Weight_Rule	Weight_Chi Squared	Weight_Gini Index	Weight_Uncertainty	Weight_Relief	Weight_Info Gain	Sum_weight
mmu-miR-15a-3p	0.234	0.091	0.198	0.057	0.330	0.093	0.060	1.062
mmu-miR-378b	0.234	0.091	0.197	0.057	0.395	0.022	0.060	1.056
mmu-miR-134-3p	0.234	0.091	0.207	0.057	0.336	0.069	0.060	1.054
mmu-miR-297b-5p	0.234	0.273	0.159	0.057	0.254	0.017	0.060	1.053
mmu-miR-669m-3p	0.258	0.091	0.146	0.102	0.334	0.012	0.110	1.053
mmu-miR-3473g	0.258	0.091	0.146	0.102	0.334	0.012	0.110	1.053
mmu-miR-7015-5p	0.234	0.091	0.155	0.057	0.350	0.104	0.060	1.051
mmu-miR-466c-5p	0.294	0.000	0.117	0.122	0.299	0.092	0.126	1.050
mmu-miR-6915-5p	0.234	0.091	0.197	0.057	0.395	0.014	0.060	1.048
mmu-miR-143-5p	0.294	0.273	0.113	0.122	0.116	0.004	0.126	1.047
mmu-miR-5615-5p	0.234	0.091	0.197	0.057	0.395	0.012	0.060	1.046
mmu-miR-1970c-5p	0.206	0.273	0.124	0.072	0.230	0.082	0.058	1.044
mmu-miR-466f-5p	0.258	0.091	0.146	0.102	0.334	0.002	0.110	1.043
mmu-miR-7240-5p	0.294	0.000	0.117	0.122	0.299	0.084	0.126	1.042
mmu-miR-7219-5p	0.294	0.000	0.117	0.122	0.299	0.084	0.126	1.042
mmu-miR-8094	0.234	0.091	0.155	0.057	0.350	0.095	0.060	1.042
mmu-miR-7066-3p	0.258	0.091	0.146	0.102	0.334	0.001	0.110	1.042
mmu-miR-12184-3p	0.294	0.000	0.117	0.122	0.299	0.081	0.126	1.039
mmu-miR-5129-3p	0.234	0.091	0.146	0.057	0.334	0.112	0.060	1.033
mmu-miR-494-5p	0.206	0.091	0.197	0.047	0.395	0.043	0.052	1.032
mmu-miR-3088-3p	0.234	0.091	0.198	0.057	0.330	0.062	0.060	1.031
mmu-miR-7013-5p	0.294	0.182	0.099	0.122	0.207	0.001	0.126	1.029
mmu-miR-7036a-3p	0.294	0.000	0.117	0.122	0.299	0.069	0.126	1.027
mmu-miR-741-3p	0.294	0.000	0.117	0.122	0.299	0.065	0.126	1.023
mmu-miR-497b	0.294	0.000	0.117	0.122	0.299	0.065	0.126	1.023
mmu-miR-495-5p	0.258	0.182	0.099	0.102	0.171	0.100	0.110	1.022
mmu-miR-7668-3p	0.294	0.000	0.117	0.122	0.299	0.062	0.126	1.020
mmu-miR-202-3p	0.294	0.000	0.117	0.122	0.299	0.062	0.126	1.020
mmu-miR-7079-3p	0.294	0.000	0.117	0.122	0.299	0.062	0.126	1.020
mmu-miR-12200-3p	0.206	0.091	0.197	0.047	0.395	0.027	0.052	1.016
mmu-miR-3070-2-3p	0.206	0.091	0.221	0.058	0.358	0.021	0.052	1.006
mmu-miR-1982-5p	0.234	0.091	0.146	0.057	0.334	0.082	0.060	1.003
mmu-miR-7212-5p	0.206	0.091	0.146	0.047	0.334	0.123	0.052	0.999
mmu-miR-669c-3p	0.206	0.182	0.099	0.050	0.171	0.238	0.052	0.999
mmu-miR-6906-3p	0.206	0.000	0.218	0.072	0.425	0.011	0.058	0.990
mmu-miR-20b-3p	0.258	0.091	0.099	0.102	0.263	0.062	0.110	0.985
mmu-miR-6380	0.258	0.091	0.099	0.102	0.263	0.061	0.110	0.984
mmu-miR-20a-3p	0.234	0.091	0.146	0.057	0.271	0.123	0.060	0.981
mmu-miR-3572-5p	0.206	0.091	0.146	0.047	0.334	0.101	0.052	0.977
mmu-miR-7669-3p	0.234	0.091	0.099	0.057	0.263	0.174	0.060	0.977
mmu-miR-467b-5p	0.258	0.091	0.099	0.102	0.263	0.051	0.110	0.974
mmu-miR-7235-5p	0.258	0.091	0.099	0.102	0.263	0.049	0.110	0.972
mmu-miR-101b-5p	0.258	0.091	0.099	0.102	0.263	0.044	0.110	0.967
mmu-miR-448-5p	0.234	0.455	0.071	0.057	0.065	0.021	0.060	0.962
mmu-miR-3081-3p	0.206	0.091	0.146	0.047	0.334	0.086	0.052	0.962
mmu-miR-6953-3p	0.258	0.091	0.099	0.102	0.263	0.038	0.110	0.961
mmu-miR-1945	0.258	0.091	0.099	0.102	0.263	0.038	0.110	0.961
mmu-miR-1b-5p	0.234	0.091	0.155	0.057	0.350	0.012	0.060	0.959
mmu-miR-7219-3p	0.206	0.000	0.155	0.047	0.350	0.142	0.052	0.953
mmu-miR-6918-5p	0.258	0.091	0.099	0.102	0.263	0.027	0.110	0.950
mmu-miR-124b-3p	0.234	0.091	0.155	0.057	0.350	0.002	0.060	0.949
mmu-miR-7047-5p	0.234	0.091	0.155	0.057	0.350	0.001	0.060	0.948
mmu-miR-7047-3p	0.234	0.091	0.146	0.057	0.334	0.026	0.060	0.947
mmu-miR-219b-3p	0.258	0.091	0.099	0.102	0.263	0.022	0.110	0.945
mmu-miR-677-3p	0.294	0.182	0.057	0.122	0.132	0.031	0.126	0.943
mmu-miR-7078-5p	0.258	0.091	0.099	0.102	0.263	0.019	0.110	0.941
mmu-miR-1930-3p	0.258	0.091	0.099	0.102	0.263	0.017	0.110	0.940
mmu-miR-1966-3p	0.258	0.091	0.099	0.102	0.263	0.014	0.110	0.936
mmu-miR-190b-3p	0.258	0.091	0.099	0.102	0.263	0.012	0.110	0.934
mmu-miR-1191b-5p	0.258	0.091	0.099	0.102	0.263	0.011	0.110	0.934
mmu-miR-669e-3p	0.258	0.091	0.099	0.102	0.263	0.011	0.110	0.934
mmu-miR-665-5p	0.258	0.091	0.099	0.102	0.263	0.005	0.110	0.928
mmu-miR-1941-3p	0.258	0.091	0.099	0.102	0.263	0.005	0.110	0.927
mmu-miR-7117-3p	0.234	0.091	0.146	0.057	0.334	0.000	0.060	0.921
mmu-miR-5112	0.234	0.273	0.100	0.072	0.174	0.003	0.060	0.915
mmu-miR-214-5p	0.206	0.000	0.155	0.047	0.369	0.082	0.052	0.911
mmu-miR-344g-5p	0.234	0.091	0.099	0.057	0.263	0.108	0.060	0.911
mmu-miR-6516-5p	0.206	0.364	0.047	0.047	0.067	0.125	0.052	0.908
mmu-miR-3087-5p	0.206	0.091	0.146	0.047	0.334	0.031	0.052	0.907
mmu-miR-1951	0.206	0.091	0.146	0.047	0.334	0.030	0.052	0.906
mmu-miR-6913-5p	0.258	0.091	0.099	0.102	0.207	0.036	0.110	0.903
mmu-miR-1934-3p	0.234	0.091	0.124	0.057	0.230	0.108	0.060	0.902
mmu-miR-146a-3p	0.258	0.091	0.099	0.102	0.207	0.030	0.110	0.897
mmu-miR-6897-5p	0.206	0.000	0.155	0.047	0.350	0.085	0.052	0.896
mmu-miR-3104-3p	0.206	0.091	0.146	0.047	0.334	0.011	0.052	0.887
mmu-miR-3074-5p	0.206	0.182	0.099	0.050	0.185	0.112	0.052	0.887
mmu-miR-365-2-5p	0.234	0.091	0.113	0.057	0.214	0.116	0.060	0.884
mmu-miR-3061-5p	0.234	0.091	0.146	0.057	0.283	0.013	0.060	0.883
mmu-miR-1982-3p	0.234	0.091	0.120	0.072	0.238	0.066	0.060	0.881
mmu-miR-6952-3p	0.234	0.182	0.099	0.057	0.207	0.039	0.060	0.877

Attribute	Weight_Info Gain Ratio	Weight_Rule	Weight_Chi Squared	Weight_Gini Index	Weight_Uncertainty	Weight_Relief	Weight_Info Gain	Sum_weight
mmu-miR-216a-3p	0.206	0.000	0.155	0.047	0.350	0.062	0.052	0.874
mmu-miR-5710	0.206	0.000	0.155	0.047	0.350	0.057	0.052	0.868
mmu-miR-7044-3p	0.234	0.182	0.099	0.057	0.207	0.019	0.060	0.857
mmu-miR-3058-5p	0.206	0.000	0.155	0.047	0.350	0.034	0.052	0.845
mmu-miR-467f	0.234	0.091	0.099	0.057	0.263	0.042	0.060	0.845
mmu-miR-669n	0.206	0.182	0.113	0.050	0.214	0.026	0.052	0.843
mmu-miR-5627-3p	0.206	0.000	0.155	0.047	0.350	0.031	0.052	0.842
mmu-miR-193a-3p	0.234	0.182	0.099	0.057	0.207	0.004	0.060	0.842
mmu-miR-669o-5p	0.206	0.000	0.155	0.047	0.369	0.008	0.052	0.837
mmu-miR-6951-5p	0.234	0.091	0.120	0.072	0.238	0.022	0.060	0.836
mmu-miR-19b-1-5p	0.206	0.000	0.155	0.047	0.350	0.024	0.052	0.835
mmu-miR-3086-5p	0.206	0.182	0.067	0.107	0.103	0.084	0.085	0.834
mmu-miR-8111	0.234	0.091	0.099	0.057	0.207	0.083	0.060	0.830
mmu-miR-3964	0.206	0.000	0.155	0.047	0.350	0.014	0.052	0.825
mmu-miR-7656-5p	0.234	0.091	0.099	0.057	0.263	0.020	0.060	0.823
mmu-miR-669m-5p	0.206	0.000	0.155	0.047	0.350	0.010	0.052	0.821
mmu-miR-3103-3p	0.234	0.091	0.099	0.057	0.263	0.016	0.060	0.819
mmu-miR-6911-5p	0.234	0.091	0.099	0.057	0.263	0.015	0.060	0.818
mmu-miR-3083-3p	0.234	0.091	0.099	0.057	0.263	0.014	0.060	0.817
mmu-miR-466f	0.206	0.000	0.155	0.047	0.350	0.003	0.052	0.815
mmu-miR-6996-5p	0.234	0.091	0.099	0.057	0.263	0.012	0.060	0.814
mmu-miR-6995-5p	0.234	0.091	0.099	0.057	0.263	0.006	0.060	0.809
mmu-miR-103-2-5p	0.234	0.091	0.099	0.057	0.263	0.003	0.060	0.806
mmu-miR-146b-3p	0.206	0.091	0.099	0.047	0.207	0.090	0.052	0.792
mmu-miR-3093-5p	0.206	0.091	0.124	0.058	0.230	0.007	0.052	0.766
mmu-miR-7243-3p	0.206	0.000	0.099	0.047	0.263	0.086	0.052	0.753
mmu-miR-466d-5p	0.206	0.000	0.099	0.047	0.263	0.073	0.052	0.740
mmu-miR-7046-5p	0.206	0.000	0.099	0.047	0.263	0.068	0.052	0.735
mmu-miR-758-3p	0.206	0.091	0.099	0.047	0.207	0.033	0.052	0.735
mmu-miR-8117	0.206	0.000	0.099	0.047	0.263	0.039	0.052	0.706
mmu-let-7a-2-3p	0.234	0.091	0.067	0.057	0.096	0.099	0.060	0.703
mmu-miR-6964-5p	0.206	0.000	0.099	0.047	0.263	0.032	0.052	0.699
mmu-miR-12187-5p	0.206	0.000	0.099	0.047	0.263	0.030	0.052	0.698
mmu-miR-6997-5p	0.206	0.000	0.099	0.047	0.263	0.022	0.052	0.689
mmu-miR-669i	0.206	0.000	0.099	0.047	0.263	0.022	0.052	0.689
mmu-miR-6921-3p	0.206	0.000	0.099	0.047	0.263	0.022	0.052	0.689
mmu-miR-467b-3p	0.206	0.000	0.099	0.047	0.263	0.021	0.052	0.689
mmu-miR-1948-3p	0.206	0.000	0.099	0.047	0.263	0.017	0.052	0.684
mmu-miR-298-3p	0.206	0.000	0.099	0.047	0.263	0.016	0.052	0.683
mmu-miR-7041-3p	0.206	0.000	0.099	0.047	0.263	0.016	0.052	0.683
mmu-miR-6947-5p	0.234	0.091	0.057	0.057	0.132	0.052	0.060	0.682
mmu-miR-1247-3p	0.206	0.000	0.099	0.047	0.263	0.007	0.052	0.674
mmu-miR-6976-5p	0.206	0.000	0.099	0.047	0.263	0.003	0.052	0.670
mmu-miR-8100	0.234	0.000	0.056	0.057	0.174	0.090	0.060	0.670
mmu-miR-3103-5p	0.234	0.091	0.057	0.057	0.132	0.019	0.060	0.649
mmu-miR-7668-5p	0.234	0.091	0.057	0.057	0.132	0.019	0.060	0.648
mmu-miR-6945-3p	0.234	0.000	0.056	0.057	0.174	0.045	0.060	0.625
mmu-miR-96-3p	0.234	0.000	0.056	0.057	0.174	0.045	0.060	0.625
mmu-miR-3097-5p	0.234	0.000	0.056	0.057	0.174	0.045	0.060	0.625
mmu-miR-6935-5p	0.234	0.000	0.056	0.057	0.174	0.045	0.060	0.625
mmu-miR-7667-3p	0.206	0.000	0.047	0.047	0.153	0.090	0.052	0.595
mmu-miR-3085-5p	0.206	0.000	0.047	0.047	0.153	0.082	0.052	0.587
mmu-miR-299b-3p	0.206	0.000	0.047	0.047	0.153	0.068	0.052	0.573
mmu-miR-466n-5p	0.206	0.000	0.047	0.047	0.153	0.068	0.052	0.573
mmu-miR-8098	0.206	0.000	0.047	0.047	0.153	0.068	0.052	0.573
mmu-miR-7033-5p	0.206	0.000	0.047	0.047	0.153	0.068	0.052	0.573
mmu-miR-6983-3p	0.206	0.000	0.047	0.047	0.153	0.068	0.052	0.573
mmu-miR-6988-5p	0.206	0.000	0.047	0.047	0.153	0.068	0.052	0.573
mmu-miR-1929-5p	0.206	0.000	0.047	0.047	0.153	0.068	0.052	0.573
mmu-miR-12181-3p	0.206	0.000	0.047	0.047	0.153	0.068	0.052	0.573
mmu-miR-3097-3p	0.234	0.091	0.025	0.057	0.037	0.049	0.060	0.553
mmu-miR-6917-5p	0.206	0.000	0.047	0.047	0.153	0.022	0.052	0.528
mmu-miR-465a-3p	0.206	0.000	0.047	0.047	0.153	0.022	0.052	0.528
mmu-miR-6904-5p	0.206	0.000	0.047	0.047	0.153	0.004	0.052	0.510
mmu-miR-5129-5p	0.234	0.091	0.000	0.057	0.000	0.039	0.060	0.481
mmu-miR-450a-1-3p	0.234	0.091	0.000	0.057	0.000	0.037	0.060	0.478
mmu-miR-6994-5p	0.206	0.000	0.000	0.047	0.000	0.135	0.052	0.440
mmu-miR-7031-5p	0.206	0.000	0.000	0.047	0.000	0.067	0.052	0.373
Timepoint	0.000	0.000	0.002	0.000	0.003	0.248	0.000	0.253
Sex	0.000	0.000	0.002	0.000	0.003	0.226	0.000	0.231
