# Supplementary material for: A Panel of miRNA Biomarkers Common to Serum and Brain-Derived Extracellular Vesicles Identified in Mouse Model of Amyotrophic Lateral Sclerosis
Source: Mol Neurobiol. 2024 Jan 22;61(8):5901–15. doi: 10.1007/s12035-023-03857-z (PMC11249427; doi:10.1007/s12035-023-03857-z)
Supplement: Supplementary file 2 — Supplementary file2 (PDF 419 KB) [file 12035_2023_3857_MOESM2_ESM.pdf]

Attribute	Weight_Info Gain Ratio	Weight_Rule	Weight_Chi Squared	Weight_Gini Index	Weight_Uncertainty	Weight_Relief	Weight_Info Gain	Sum
mmu-let-7a-5p	1.000	0.818	0.772	0.713	0.838	0.720	0.842	5.702
mmu-miR-10b-5p	0.897	0.545	0.535	1.000	0.493	0.672	1.000	5.143
mmu-miR-16-5p	1.000	1.000	0.587	0.713	0.634	0.271	0.842	5.046
mmu-miR-674-5p	0.666	0.364	0.951	0.751	0.958	0.517	0.734	4.939
mmu-miR-19b-3p	1.000	0.909	0.481	0.713	0.556	0.330	0.842	4.830
mmu-miR-2137	0.867	0.818	0.514	0.751	0.468	0.657	0.734	4.808
mmu-miR-7a-5p	0.666	0.909	0.499	0.751	0.673	0.249	0.734	4.481
mmu-miR-98-5p	0.626	0.455	0.572	0.542	0.714	1.000	0.522	4.430
mmu-miR-671-5p	0.867	0.909	0.562	0.539	0.688	0.221	0.643	4.430
mmu-miR-451a	1.000	0.727	0.343	0.713	0.385	0.365	0.842	4.374
mmu-miR-375-3p	0.758	0.909	0.733	0.452	0.836	0.078	0.562	4.328
mmu-miR-107-3p	0.593	0.545	0.523	0.591	0.685	0.633	0.586	4.155
mmu-miR-101a-3p	0.744	0.909	0.610	0.383	0.762	0.262	0.462	4.133
mmu-miR-204-5p	0.867	0.364	0.377	0.539	0.688	0.654	0.643	4.132
mmu-let-7f-5p	0.867	0.909	0.419	0.539	0.422	0.310	0.643	4.109
mmu-miR-361-3p	0.867	0.455	0.557	0.591	0.686	0.240	0.643	4.037
mmu-miR-128-3p	0.626	0.909	0.604	0.591	0.603	0.082	0.586	4.000
mmu-miR-5108	0.498	0.727	1.000	0.354	1.000	0.045	0.334	3.959
mmu-miR-30e-5p	0.867	0.545	0.535	0.539	0.535	0.270	0.643	3.935
mmu-miR-328-3p	0.744	0.818	0.548	0.560	0.519	0.145	0.544	3.878
mmu-miR-143-3p	0.626	0.727	0.664	0.534	0.695	0.113	0.513	3.872
mmu-miR-122-5p	0.758	0.909	0.286	0.452	0.572	0.312	0.562	3.850
mmu-miR-203-3p	0.666	0.818	0.259	0.751	0.531	0.090	0.734	3.849
mmu-miR-27b-3p	0.593	0.909	0.412	0.591	0.419	0.308	0.586	3.818
mmu-miR-1843a-5p	0.867	0.364	0.465	0.539	0.721	0.197	0.643	3.796
mmu-miR-1a-3p	0.666	0.818	0.259	0.751	0.531	0.016	0.734	3.774
mmu-miR-200a-3p	0.549	0.727	0.664	0.542	0.680	0.074	0.522	3.759
mmu-miR-141-3p	0.626	0.636	0.804	0.363	0.851	0.132	0.344	3.755
mmu-let-7c-5p	0.626	0.909	0.772	0.241	0.733	0.143	0.295	3.719
mmu-miR-192-5p	0.758	0.909	0.492	0.452	0.465	0.059	0.562	3.698
mmu-miR-361-5p	0.626	0.727	0.793	0.241	0.867	0.126	0.295	3.676
mmu-miR-423-5p	0.652	0.909	0.656	0.321	0.642	0.091	0.405	3.675
mmu-miR-125b-5p	0.758	0.909	0.436	0.452	0.411	0.103	0.562	3.631
mmu-miR-22-3p	0.652	1.000	0.569	0.363	0.538	0.089	0.405	3.616
mmu-miR-186-5p	0.744	0.273	0.621	0.534	0.607	0.235	0.513	3.527
mmu-miR-25-3p	0.744	0.636	0.587	0.383	0.572	0.136	0.462	3.521
mmu-miR-103-3p	0.867	0.364	0.363	0.591	0.374	0.312	0.643	3.514
mmu-miR-127-3p	0.498	0.818	0.729	0.149	0.910	0.226	0.141	3.471
mmu-miR-125a-5p	0.498	0.909	0.699	0.320	0.675	0.046	0.318	3.465
mmu-miR-28a-3p	0.758	0.273	0.514	0.462	0.767	0.126	0.562	3.461
mmu-miR-148b-3p	0.867	0.636	0.376	0.539	0.358	0.031	0.643	3.450
mmu-miR-106b-3p	0.498	0.909	0.613	0.320	0.592	0.190	0.318	3.440
mmu-miR-30d-5p	0.498	0.909	0.750	0.259	0.708	0.066	0.247	3.438
mmu-miR-425-3p	0.652	0.455	0.597	0.321	0.830	0.166	0.405	3.425
mmu-miR-191-5p	0.498	0.909	0.470	0.534	0.412	0.084	0.513	3.421
mmu-miR-5119	0.744	1.000	0.372	0.383	0.382	0.022	0.462	3.364
mmu-miR-423-3p	0.652	0.909	0.384	0.321	0.353	0.298	0.405	3.322
mmu-let-7i-5p	0.626	0.636	0.386	0.591	0.445	0.041	0.586	3.310
mmu-miR-30a-3p	0.438	0.727	0.638	0.227	0.665	0.368	0.214	3.277
mmu-miR-17-3p	0.438	0.909	0.610	0.268	0.700	0.052	0.259	3.236
mmu-miR-484	0.626	0.818	0.578	0.241	0.529	0.142	0.295	3.229
mmu-miR-21a-5p	0.758	0.636	0.329	0.462	0.343	0.129	0.562	3.220
mmu-miR-486a-5p	0.744	0.727	0.379	0.462	0.402	0.021	0.463	3.198
mmu-miR-10a-5p	0.498	0.909	0.587	0.320	0.550	0.016	0.318	3.198
mmu-miR-8110	0.744	0.273	0.286	0.383	0.572	0.467	0.462	3.187
mmu-miR-30c-5p	0.549	0.727	0.674	0.227	0.721	0.024	0.259	3.181
mmu-miR-322-5p	0.758	0.273	0.476	0.452	0.547	0.088	0.562	3.156
mmu-miR-15a-5p	0.744	0.364	0.483	0.417	0.557	0.111	0.462	3.139
mmu-miR-194-5p	0.485	0.818	0.347	0.542	0.362	0.060	0.522	3.137
mmu-miR-144-3p	0.626	0.818	0.521	0.241	0.552	0.079	0.295	3.131
mmu-miR-23a-3p	0.593	0.455	0.384	0.591	0.352	0.161	0.586	3.121
mmu-miR-320-3p	0.498	1.000	0.600	0.114	0.555	0.208	0.140	3.115
mmu-miR-219a-5p	0.758	0.273	0.336	0.452	0.513	0.202	0.562	3.095
mmu-miR-378a-5p	0.758	0.273	0.334	0.452	0.580	0.111	0.562	3.070
mmu-miR-142a-5p	0.498	0.909	0.492	0.268	0.469	0.155	0.259	3.049
mmu-miR-26a-5p	0.549	1.000	0.492	0.227	0.487	0.034	0.259	3.048
mmu-miR-383-5p	0.744	0.273	0.242	0.383	0.541	0.403	0.462	3.048
mmu-miR-24-3p	0.438	0.727	0.600	0.354	0.571	0.015	0.334	3.039
mmu-miR-344-3p	0.744	0.273	0.242	0.383	0.514	0.418	0.462	3.036
mmu-let-7i-3p	0.744	0.273	0.242	0.383	0.514	0.357	0.462	2.975
mmu-miR-9-5p	0.498	0.909	0.259	0.369	0.508	0.073	0.351	2.966
mmu-miR-126a-5p	0.498	0.818	0.419	0.395	0.422	0.032	0.380	2.964
mmu-miR-363-3p	0.744	0.273	0.286	0.383	0.572	0.237	0.462	2.956
mmu-miR-99a-5p	0.549	0.545	0.288	0.395	0.403	0.378	0.380	2.939
mmu-miR-5112	0.652	0.545	0.363	0.369	0.366	0.222	0.405	2.923
mmu-miR-199a-3p	0.652	0.909	0.225	0.321	0.232	0.174	0.405	2.917
mmu-miR-152-3p	0.549	0.909	0.384	0.202	0.376	0.232	0.259	2.911
mmu-miR-29b-3p	0.438	0.818	0.575	0.226	0.534	0.105	0.213	2.910
mmu-miR-150-5p	0.626	0.727	0.415	0.241	0.422	0.184	0.295	2.910

Attribute	Weight_Info Gain Ratio	Weight_Rule	Weight_Chi Squared	Weight_Gini Index	Weight_Uncertainty	Weight_Relief	Weight_Info Gain	Sum
mmu-miR-187-3p	0.438	0.455	0.571	0.320	0.714	0.093	0.318	2.908
mmu-miR-181a-5p	0.652	0.364	0.406	0.369	0.379	0.325	0.405	2.898
mmu-miR-181c-5p	0.744	0.273	0.346	0.383	0.501	0.184	0.462	2.893
mmu-let-7e-5p	0.626	0.182	0.597	0.241	0.840	0.110	0.295	2.890
mmu-miR-221-3p	0.652	0.909	0.178	0.321	0.179	0.231	0.405	2.876
mmu-miR-205-5p	0.549	0.727	0.311	0.320	0.356	0.293	0.318	2.875
mmu-miR-1198-5p	0.438	0.545	0.515	0.320	0.646	0.064	0.318	2.847
mmu-miR-8114	0.744	0.273	0.242	0.383	0.514	0.202	0.462	2.820
mmu-miR-421-3p	0.744	0.273	0.242	0.383	0.514	0.187	0.462	2.805
mmu-miR-378c	0.652	0.636	0.346	0.321	0.375	0.065	0.405	2.800
mmu-miR-26b-5p	0.438	0.818	0.569	0.114	0.538	0.199	0.123	2.800
mmu-miR-27a-3p	0.498	0.818	0.467	0.213	0.510	0.093	0.199	2.798
mmu-miR-374b-5p	0.744	0.273	0.286	0.383	0.572	0.071	0.462	2.790
mmu-miR-30a-5p	0.626	1.000	0.290	0.241	0.279	0.053	0.295	2.783
mmu-miR-342-3p	0.438	0.909	0.483	0.226	0.459	0.052	0.213	2.781
mmu-miR-140-5p	0.744	0.273	0.286	0.383	0.547	0.045	0.462	2.739
mmu-miR-200c-3p	0.498	0.636	0.521	0.213	0.584	0.076	0.199	2.727
mmu-miR-339-3p	0.498	0.545	0.557	0.113	0.686	0.188	0.140	2.726
mmu-miR-378a-3p	0.626	0.818	0.371	0.241	0.351	0.004	0.295	2.707
mmu-miR-5106	0.549	0.364	0.524	0.369	0.525	0.003	0.351	2.685
mmu-miR-10b-3p	0.549	0.273	0.449	0.202	0.694	0.252	0.259	2.678
mmu-miR-434-3p	0.438	0.273	0.518	0.094	0.709	0.523	0.123	2.677
mmu-miR-615-3p	0.549	0.364	0.449	0.202	0.658	0.195	0.259	2.676
mmu-miR-126a-3p	0.498	0.909	0.406	0.113	0.404	0.198	0.140	2.668
mmu-miR-676-3p	0.498	0.727	0.438	0.143	0.428	0.289	0.140	2.664
mmu-miR-33-5p	0.438	0.364	0.518	0.268	0.764	0.023	0.259	2.634
mmu-miR-145a-3p	0.652	0.273	0.320	0.321	0.512	0.143	0.405	2.625
mmu-miR-425-5p	0.438	0.818	0.470	0.149	0.429	0.177	0.141	2.623
mmu-miR-92a-3p	0.498	0.909	0.397	0.113	0.361	0.188	0.140	2.606
mmu-miR-206-3p	0.549	0.545	0.216	0.202	0.362	0.467	0.259	2.601
mmu-miR-339-5p	0.549	0.727	0.277	0.354	0.300	0.046	0.334	2.588
mmu-miR-532-5p	0.438	0.455	0.347	0.259	0.397	0.445	0.247	2.587
mmu-miR-8117	0.498	0.636	0.475	0.113	0.535	0.174	0.140	2.570
mmu-miR-145a-5p	0.498	0.636	0.406	0.199	0.387	0.234	0.195	2.556
mmu-miR-146a-5p	0.438	0.909	0.320	0.109	0.309	0.344	0.123	2.552
mmu-miR-130a-3p	0.498	0.636	0.502	0.113	0.606	0.051	0.140	2.546
mmu-miR-338-3p	0.438	0.727	0.415	0.143	0.415	0.245	0.136	2.519
mmu-miR-15b-5p	0.438	0.636	0.404	0.094	0.455	0.362	0.123	2.512
mmu-miR-19a-3p	0.626	0.364	0.389	0.241	0.549	0.037	0.295	2.500
mmu-miR-139-3p	0.652	0.273	0.346	0.321	0.486	0.013	0.405	2.494
mmu-miR-93-5p	0.498	0.909	0.320	0.143	0.284	0.176	0.140	2.470
mmu-miR-23b-3p	0.498	0.636	0.403	0.213	0.448	0.072	0.199	2.469
mmu-miR-223-3p	0.498	0.909	0.354	0.123	0.353	0.088	0.140	2.465
mmu-miR-99b-5p	0.498	0.545	0.320	0.113	0.366	0.480	0.140	2.461
mmu-let-7b-5p	0.498	1.000	0.182	0.227	0.178	0.160	0.214	2.458
mmu-miR-29a-3p	0.498	1.000	0.223	0.123	0.230	0.241	0.140	2.455
mmu-miR-362-5p	0.652	0.182	0.204	0.321	0.451	0.241	0.405	2.455
mmu-miR-93-3p	0.498	0.545	0.435	0.123	0.479	0.231	0.140	2.452
mmu-let-7g-5p	0.626	0.455	0.354	0.268	0.331	0.122	0.295	2.451
mmu-miR-466i-5p	0.485	0.636	0.062	0.542	0.197	0.006	0.522	2.449
mmu-miR-185-5p	0.438	0.909	0.320	0.099	0.340	0.215	0.123	2.444
mmu-miR-181a-1-3p	0.652	0.273	0.303	0.321	0.393	0.079	0.405	2.426
mmu-miR-140-3p	0.498	0.818	0.257	0.199	0.278	0.178	0.195	2.424
mmu-miR-378d	0.626	0.364	0.394	0.241	0.410	0.078	0.295	2.407
mmu-miR-101b-3p	0.549	0.455	0.346	0.226	0.496	0.062	0.259	2.393
mmu-let-7d-3p	0.438	0.636	0.360	0.259	0.397	0.050	0.247	2.387
mmu-miR-215-5p	0.438	0.909	0.298	0.213	0.249	0.080	0.199	2.385
mmu-miR-382-5p	0.498	0.273	0.449	0.113	0.671	0.228	0.140	2.372
mmu-miR-222-3p	0.498	0.727	0.397	0.123	0.415	0.071	0.140	2.372
mmu-miR-744-5p	0.438	0.818	0.320	0.149	0.319	0.165	0.141	2.350
mmu-miR-669c-5p	0.626	0.182	0.204	0.241	0.451	0.345	0.295	2.343
mmu-miR-199a-5p	0.549	0.273	0.389	0.202	0.532	0.139	0.259	2.343
mmu-miR-351-5p	0.652	0.455	0.216	0.321	0.252	0.040	0.405	2.341
mmu-miR-138-5p	0.626	0.364	0.248	0.268	0.443	0.097	0.295	2.340
mmu-miR-1839-5p	0.498	0.545	0.427	0.114	0.518	0.087	0.140	2.330
mmu-miR-215-3p	0.549	0.455	0.363	0.202	0.442	0.060	0.259	2.330
mmu-miR-20a-5p	0.438	0.727	0.265	0.227	0.310	0.148	0.214	2.329
mmu-miR-200b-3p	0.438	0.545	0.320	0.227	0.418	0.161	0.214	2.323
mmu-miR-664-5p	0.626	0.182	0.320	0.241	0.584	0.073	0.295	2.321
mmu-miR-130b-3p	0.626	0.182	0.320	0.241	0.619	0.034	0.295	2.316
mmu-miR-709	0.498	0.545	0.338	0.227	0.449	0.044	0.214	2.315
mmu-miR-210-3p	0.549	0.364	0.389	0.202	0.545	0.002	0.259	2.310
mmu-miR-301a-3p	0.498	0.091	0.518	0.113	0.709	0.235	0.140	2.303
mmu-miR-455-5p	0.498	0.273	0.406	0.199	0.529	0.186	0.195	2.286
mmu-miR-100-5p	0.626	0.545	0.105	0.369	0.152	0.132	0.351	2.279
mmu-miR-29c-3p	0.652	0.273	0.256	0.369	0.265	0.059	0.405	2.278
mmu-miR-146b-5p	0.498	0.636	0.309	0.149	0.319	0.217	0.141	2.269
mmu-miR-127-5p	0.652	0.182	0.204	0.321	0.451	0.055	0.405	2.268
mmu-miR-5124a	0.438	0.909	0.234	0.104	0.316	0.143	0.123	2.267

Attribute	Weight_Info Gain Ratio	Weight_Rule	Weight_Chi Squared	Weight_Gini Index	Weight_Uncertainty	Weight_Relief	Weight_Info Gain	Sum
mmu-miR-345-3p	0.652	0.364	0.164	0.321	0.250	0.110	0.405	2.264
mmu-miR-96-5p	0.626	0.182	0.154	0.241	0.408	0.353	0.295	2.259
mmu-let-7d-5p	0.438	0.545	0.427	0.104	0.421	0.197	0.123	2.255
mmu-miR-150-3p	0.652	0.000	0.204	0.321	0.451	0.217	0.405	2.249
mmu-miR-133a-3p	0.438	0.818	0.213	0.094	0.231	0.329	0.123	2.246
mmu-miR-324-5p	0.438	0.364	0.248	0.268	0.443	0.222	0.259	2.242
mmu-miR-1981-5p	0.549	0.455	0.308	0.259	0.338	0.067	0.259	2.235
mmu-miR-429-3p	0.438	0.545	0.339	0.099	0.415	0.270	0.123	2.229
mmu-miR-1306-3p	0.652	0.000	0.204	0.321	0.474	0.162	0.405	2.217
mmu-miR-31-5p	0.549	0.273	0.389	0.202	0.532	0.012	0.259	2.216
mmu-miR-340-5p	0.438	0.545	0.335	0.212	0.435	0.052	0.198	2.215
mmu-miR-144-5p	0.498	0.636	0.341	0.113	0.422	0.051	0.140	2.201
mmu-miR-483-5p	0.549	0.364	0.289	0.202	0.504	0.024	0.259	2.191
mmu-miR-30c-1-3p	0.626	0.182	0.204	0.241	0.451	0.187	0.295	2.185
mmu-miR-15b-3p	0.498	0.273	0.388	0.143	0.636	0.095	0.140	2.173
mmu-miR-148a-3p	0.438	0.909	0.209	0.114	0.277	0.101	0.123	2.172
mmu-miR-708-5p	0.626	0.182	0.204	0.241	0.451	0.171	0.295	2.169
mmu-miR-6987-5p	0.626	0.182	0.154	0.241	0.385	0.285	0.295	2.168
mmu-miR-16-1-3p	0.626	0.182	0.204	0.241	0.474	0.143	0.295	2.164
mmu-miR-541-5p	0.626	0.182	0.259	0.241	0.508	0.050	0.295	2.160
mmu-miR-376b-5p	0.626	0.182	0.154	0.241	0.385	0.275	0.295	2.157
mmu-miR-1191a	0.626	0.182	0.129	0.241	0.266	0.411	0.295	2.150
mmu-miR-133b-3p	0.438	0.273	0.320	0.094	0.495	0.408	0.123	2.150
mmu-miR-497a-5p	0.652	0.000	0.204	0.321	0.451	0.112	0.405	2.144
mmu-miR-224-5p	0.626	0.182	0.154	0.241	0.385	0.256	0.295	2.138
mmu-miR-125b-1-3p	0.438	0.364	0.289	0.268	0.460	0.049	0.259	2.128
mmu-miR-1247-5p	0.438	0.273	0.334	0.143	0.580	0.212	0.136	2.116
mmu-miR-195a-5p	0.549	0.091	0.259	0.202	0.508	0.239	0.259	2.107
mmu-miR-877-3p	0.549	0.182	0.320	0.202	0.562	0.034	0.259	2.107
mmu-miR-151-3p	0.438	0.636	0.281	0.213	0.303	0.032	0.199	2.102
mmu-let-7j	0.438	0.545	0.260	0.212	0.281	0.167	0.198	2.101
mmu-miR-155-5p	0.438	0.455	0.320	0.099	0.384	0.276	0.123	2.095
mmu-miR-30d-3p	0.626	0.182	0.154	0.241	0.385	0.205	0.295	2.087
mmu-miR-5107-5p	0.652	0.000	0.204	0.321	0.474	0.030	0.405	2.086
mmu-miR-106b-5p	0.626	0.182	0.204	0.241	0.451	0.088	0.295	2.086
mmu-miR-125b-2-3p	0.498	0.273	0.334	0.143	0.603	0.093	0.140	2.084
mmu-miR-181d-5p	0.438	0.455	0.389	0.149	0.485	0.015	0.141	2.072
mmu-miR-149-5p	0.438	0.182	0.380	0.259	0.521	0.043	0.247	2.070
mmu-miR-181b-5p	0.549	0.455	0.265	0.213	0.294	0.034	0.259	2.068
mmu-miR-129-5p	0.626	0.182	0.154	0.241	0.385	0.180	0.295	2.062
mmu-miR-34a-5p	0.498	0.818	0.173	0.113	0.177	0.140	0.140	2.059
mmu-miR-218-5p	0.498	0.091	0.380	0.259	0.538	0.039	0.247	2.052
mmu-miR-136-3p	0.626	0.182	0.204	0.241	0.451	0.052	0.295	2.050
mmu-miR-185-3p	0.626	0.182	0.204	0.241	0.451	0.046	0.295	2.043
mmu-miR-214-3p	0.652	0.000	0.204	0.321	0.451	0.010	0.405	2.042
mmu-miR-99a-3p	0.626	0.182	0.154	0.241	0.385	0.155	0.295	2.038
mmu-miR-137-3p	0.498	0.091	0.334	0.199	0.639	0.076	0.195	2.032
mmu-miR-92a-1-5p	0.626	0.182	0.204	0.241	0.451	0.027	0.295	2.024
mmu-miR-532-3p	0.438	0.182	0.388	0.094	0.696	0.101	0.123	2.021
mmu-miR-142a-3p	0.549	0.364	0.277	0.259	0.247	0.067	0.259	2.021
mmu-miR-1964-3p	0.498	0.273	0.334	0.113	0.603	0.054	0.140	2.014
mmu-miR-326-3p	0.438	0.636	0.341	0.109	0.298	0.062	0.123	2.008
mmu-miR-22-5p	0.438	0.273	0.320	0.094	0.495	0.260	0.123	2.002
mmu-miR-30c-2-3p	0.626	0.182	0.154	0.241	0.385	0.109	0.295	1.991
mmu-miR-499-5p	0.626	0.182	0.154	0.241	0.385	0.093	0.295	1.976
mmu-miR-669a-3p	0.626	0.182	0.154	0.241	0.385	0.093	0.295	1.976
mmu-miR-376a-3p	0.438	0.091	0.259	0.099	0.508	0.448	0.123	1.966
mmu-miR-1954	0.626	0.182	0.154	0.241	0.385	0.077	0.295	1.959
mmu-let-7a-1-3p	0.498	0.091	0.259	0.113	0.508	0.339	0.140	1.947
mmu-miR-124-3p	0.438	0.273	0.205	0.094	0.368	0.442	0.123	1.942
mmu-miR-25-5p	0.438	0.273	0.334	0.143	0.580	0.036	0.136	1.940
mmu-miR-223-5p	0.498	0.182	0.320	0.113	0.584	0.099	0.140	1.936
mmu-miR-491-5p	0.626	0.182	0.154	0.241	0.385	0.047	0.295	1.929
mmu-miR-1306-5p	0.438	0.273	0.286	0.143	0.572	0.079	0.136	1.926
mmu-miR-8109	0.498	0.636	0.129	0.113	0.338	0.068	0.140	1.922
mmu-miR-574-3p	0.498	0.091	0.388	0.113	0.636	0.056	0.140	1.922
mmu-miR-18a-3p	0.438	0.182	0.259	0.094	0.508	0.317	0.123	1.920
mmu-miR-431-5p	0.549	0.000	0.129	0.202	0.338	0.438	0.259	1.916
mmu-miR-501-3p	0.498	0.455	0.218	0.113	0.259	0.223	0.140	1.906
mmu-miR-350-5p	0.438	0.182	0.259	0.094	0.531	0.278	0.123	1.904
mmu-miR-500-3p	0.498	0.182	0.320	0.113	0.584	0.058	0.140	1.895
mmu-miR-674-3p	0.549	0.000	0.129	0.202	0.338	0.410	0.259	1.887
mmu-miR-5100	0.438	0.182	0.259	0.094	0.508	0.276	0.123	1.879
mmu-miR-3473b	0.438	0.727	0.062	0.227	0.197	0.006	0.214	1.870
mmu-miR-30b-5p	0.438	0.727	0.176	0.104	0.189	0.108	0.123	1.865
mmu-miR-184-3p	0.438	0.636	0.139	0.094	0.163	0.265	0.123	1.858
mmu-miR-296-5p	0.438	0.364	0.155	0.268	0.262	0.111	0.259	1.856
mmu-miR-143-5p	0.438	0.455	0.205	0.226	0.310	0.000	0.213	1.847
mmu-miR-29a-5p	0.498	0.091	0.320	0.113	0.562	0.107	0.140	1.830

Attribute	Weight_Info Gain Ratio	Weight_Rule	Weight_Chi Squared	Weight_Gini Index	Weight_Uncertainty	Weight_Relief	Weight_Info Gain	Sum
mmu-miR-23a-5p	0.498	0.091	0.320	0.113	0.478	0.188	0.140	1.828
mmu-miR-434-5p	0.498	0.364	0.216	0.113	0.311	0.185	0.140	1.827
mmu-miR-540-3p	0.438	0.182	0.336	0.149	0.513	0.053	0.141	1.812
mmu-miR-341-3p	0.549	0.000	0.129	0.202	0.338	0.334	0.259	1.812
mmu-miR-219a-1-3p	0.549	0.000	0.129	0.202	0.338	0.334	0.259	1.812
mmu-miR-3968	0.438	0.182	0.259	0.094	0.508	0.205	0.123	1.809
mmu-miR-8101	0.438	0.182	0.320	0.099	0.562	0.065	0.123	1.789
mmu-miR-322-3p	0.549	0.091	0.191	0.202	0.452	0.026	0.259	1.770
mmu-miR-5114	0.498	0.091	0.191	0.113	0.430	0.300	0.140	1.762
mmu-miR-1249-3p	0.438	0.182	0.259	0.094	0.508	0.154	0.123	1.758
mmu-miR-6538	0.438	0.273	0.277	0.094	0.425	0.119	0.123	1.748
mmu-miR-183-5p	0.498	0.091	0.346	0.123	0.501	0.032	0.140	1.732
mmu-miR-181c-3p	0.438	0.182	0.129	0.094	0.266	0.479	0.123	1.711
mmu-miR-7b-5p	0.626	0.182	0.074	0.241	0.169	0.092	0.295	1.679
mmu-miR-186-3p	0.438	0.182	0.259	0.094	0.531	0.049	0.123	1.675
mmu-miR-667-3p	0.549	0.000	0.129	0.202	0.338	0.197	0.259	1.674
mmu-miR-99b-3p	0.549	0.000	0.129	0.202	0.359	0.176	0.259	1.674
mmu-miR-134-5p	0.626	0.182	0.086	0.241	0.177	0.057	0.295	1.664
mmu-miR-27a-5p	0.549	0.091	0.129	0.202	0.338	0.090	0.259	1.658
mmu-miR-29c-5p	0.438	0.273	0.205	0.094	0.383	0.141	0.123	1.656
mmu-miR-136-5p	0.498	0.091	0.191	0.113	0.430	0.188	0.140	1.650
mmu-miR-700-3p	0.549	0.000	0.129	0.202	0.338	0.165	0.259	1.642
mmu-miR-196a-5p	0.549	0.000	0.129	0.202	0.359	0.138	0.259	1.636
mmu-miR-503-3p	0.549	0.000	0.129	0.202	0.359	0.138	0.259	1.636
mmu-miR-7036b-5p	0.498	0.091	0.129	0.113	0.338	0.327	0.140	1.636
mmu-miR-30e-3p	0.498	0.182	0.204	0.113	0.451	0.023	0.140	1.609
mmu-miR-17-5p	0.498	0.182	0.204	0.113	0.451	0.011	0.140	1.597
mmu-miR-300-3p	0.438	0.182	0.204	0.094	0.451	0.106	0.123	1.596
mmu-miR-331-3p	0.549	0.000	0.129	0.202	0.338	0.116	0.259	1.593
mmu-miR-3960	0.438	0.273	0.216	0.099	0.362	0.072	0.123	1.583
mmu-miR-3535	0.549	0.000	0.129	0.202	0.338	0.104	0.259	1.581
mmu-miR-222-5p	0.549	0.000	0.129	0.202	0.338	0.104	0.259	1.581
mmu-miR-132-3p	0.498	0.182	0.148	0.113	0.275	0.224	0.140	1.580
mmu-miR-301b-3p	0.438	0.091	0.259	0.099	0.531	0.034	0.123	1.575
mmu-miR-505-5p	0.498	0.091	0.129	0.113	0.338	0.265	0.140	1.574
mmu-miR-23b-5p	0.549	0.000	0.129	0.202	0.338	0.088	0.259	1.565
mmu-miR-1971	0.549	0.000	0.129	0.202	0.338	0.081	0.259	1.558
mmu-miR-379-5p	0.549	0.091	0.129	0.202	0.266	0.062	0.259	1.558
mmu-miR-365-3p	0.549	0.000	0.129	0.202	0.338	0.080	0.259	1.558
mmu-miR-188-5p	0.549	0.000	0.129	0.202	0.338	0.080	0.259	1.558
mmu-miR-193b-3p	0.549	0.000	0.129	0.202	0.338	0.075	0.259	1.552
mmu-miR-501-5p	0.549	0.000	0.129	0.202	0.338	0.075	0.259	1.552
mmu-miR-450b-5p	0.498	0.091	0.129	0.113	0.338	0.240	0.140	1.549
mmu-miR-676-5p	0.498	0.091	0.191	0.113	0.452	0.056	0.140	1.541
mmu-miR-181a-2-3p	0.549	0.000	0.129	0.202	0.338	0.061	0.259	1.539
mmu-miR-872-3p	0.549	0.000	0.129	0.202	0.338	0.052	0.259	1.530
mmu-miR-92b-3p	0.549	0.000	0.129	0.202	0.338	0.049	0.259	1.527
mmu-miR-381-3p	0.498	0.091	0.191	0.113	0.430	0.061	0.140	1.523
mmu-miR-431-3p	0.549	0.000	0.129	0.202	0.338	0.040	0.259	1.517
mmu-miR-190b-5p	0.498	0.091	0.191	0.113	0.430	0.055	0.140	1.517
mmu-miR-335-5p	0.498	0.091	0.191	0.113	0.430	0.055	0.140	1.517
mmu-miR-139-5p	0.498	0.091	0.216	0.113	0.362	0.085	0.140	1.505
mmu-miR-376b-3p	0.498	0.091	0.129	0.113	0.338	0.184	0.140	1.492
mmu-miR-369-3p	0.498	0.091	0.129	0.113	0.338	0.169	0.140	1.478
mmu-miR-409-3p	0.438	0.091	0.191	0.094	0.430	0.104	0.123	1.470
mmu-miR-330-5p	0.438	0.091	0.191	0.094	0.430	0.086	0.123	1.452
mmu-miR-152-5p	0.438	0.091	0.191	0.094	0.430	0.076	0.123	1.442
mmu-miR-503-5p	0.498	0.091	0.129	0.113	0.338	0.130	0.140	1.439
mmu-miR-153-3p	0.438	0.182	0.129	0.094	0.266	0.193	0.123	1.424
mmu-miR-486a-3p	0.498	0.273	0.130	0.143	0.203	0.030	0.140	1.416
mmu-miR-671-3p	0.438	0.091	0.191	0.094	0.430	0.037	0.123	1.403
mmu-miR-298-5p	0.498	0.273	0.130	0.123	0.220	0.005	0.140	1.389
mmu-miR-3057-5p	0.438	0.000	0.062	0.094	0.197	0.467	0.123	1.380
mmu-miR-350-3p	0.498	0.091	0.129	0.113	0.338	0.070	0.140	1.379
mmu-miR-1943-5p	0.498	0.091	0.129	0.113	0.338	0.061	0.140	1.370
mmu-miR-101c	0.549	0.091	0.062	0.202	0.197	0.011	0.259	1.370
mmu-miR-542-5p	0.498	0.091	0.129	0.113	0.338	0.053	0.140	1.362
mmu-miR-125a-3p	0.498	0.091	0.129	0.113	0.338	0.042	0.140	1.351
mmu-miR-148b-5p	0.498	0.091	0.129	0.113	0.338	0.042	0.140	1.351
mmu-miR-19b-1-5p	0.498	0.091	0.129	0.113	0.338	0.027	0.140	1.336
mmu-miR-9-3p	0.498	0.182	0.129	0.113	0.266	0.007	0.140	1.335
mmu-miR-574-5p	0.498	0.091	0.129	0.113	0.338	0.024	0.140	1.333
mmu-miR-5113	0.498	0.091	0.129	0.113	0.338	0.022	0.140	1.331
mmu-miR-10a-3p	0.498	0.091	0.129	0.113	0.338	0.019	0.140	1.328
mmu-miR-760-3p	0.498	0.091	0.129	0.113	0.338	0.019	0.140	1.328
mmu-miR-3473a	0.498	0.091	0.129	0.113	0.338	0.018	0.140	1.327
mmu-miR-3074-5p	0.498	0.091	0.129	0.113	0.338	0.018	0.140	1.327
mmu-miR-1950	0.498	0.091	0.073	0.113	0.223	0.187	0.140	1.325
mmu-miR-1964-5p	0.498	0.091	0.073	0.113	0.223	0.187	0.140	1.325

Attribute	Weight_Info Gain Ratio	Weight_Rule	Weight_Chi Squared	Weight_Gini Index	Weight_Uncertainty	Weight_Relief	Weight_Info Gain	Sum
mmu-miR-18a-5p	0.498	0.091	0.129	0.113	0.338	0.012	0.140	1.321
mmu-miR-200b-5p	0.498	0.091	0.129	0.113	0.338	0.003	0.140	1.312
mmu-miR-3964	0.498	0.091	0.129	0.113	0.338	0.003	0.140	1.312
mmu-miR-664-3p	0.498	0.091	0.129	0.113	0.338	0.001	0.140	1.310
mmu-miR-672-5p	0.498	0.091	0.129	0.113	0.338	0.001	0.140	1.309
mmu-miR-195a-3p	0.438	0.091	0.129	0.094	0.338	0.073	0.123	1.286
mmu-miR-193a-5p	0.438	0.091	0.129	0.099	0.338	0.063	0.123	1.281
mmu-miR-5121	0.498	0.091	0.073	0.113	0.223	0.140	0.140	1.278
mmu-miR-879-3p	0.498	0.091	0.073	0.113	0.223	0.140	0.140	1.278
mmu-miR-376a-5p	0.498	0.091	0.073	0.113	0.223	0.140	0.140	1.278
mmu-miR-3099-3p	0.498	0.091	0.073	0.113	0.223	0.140	0.140	1.278
mmu-miR-592-5p	0.498	0.091	0.073	0.113	0.223	0.140	0.140	1.278
mmu-miR-1970	0.438	0.091	0.129	0.094	0.338	0.064	0.123	1.277
mmu-miR-214-5p	0.498	0.091	0.148	0.113	0.275	0.011	0.140	1.275
mmu-miR-873a-5p	0.438	0.091	0.129	0.094	0.338	0.051	0.123	1.264
mmu-miR-34c-5p	0.498	0.182	0.062	0.113	0.118	0.143	0.140	1.255
mmu-miR-511-5p	0.438	0.182	0.129	0.099	0.266	0.016	0.123	1.253
mmu-miR-872-5p	0.498	0.182	0.074	0.113	0.169	0.064	0.140	1.240
mmu-miR-582-3p	0.498	0.091	0.073	0.113	0.223	0.093	0.140	1.231
mmu-miR-146a-3p	0.498	0.091	0.073	0.113	0.223	0.093	0.140	1.231
mmu-miR-7115-5p	0.498	0.091	0.073	0.113	0.223	0.093	0.140	1.231
mmu-miR-212-5p	0.498	0.091	0.073	0.113	0.223	0.093	0.140	1.231
mmu-miR-154-5p	0.498	0.091	0.073	0.113	0.223	0.093	0.140	1.231
mmu-miR-488-3p	0.498	0.091	0.073	0.113	0.223	0.093	0.140	1.231
mmu-miR-335-3p	0.498	0.091	0.073	0.113	0.223	0.093	0.140	1.231
mmu-miR-382-3p	0.498	0.091	0.073	0.113	0.223	0.093	0.140	1.231
mmu-miR-384-5p	0.498	0.091	0.073	0.113	0.223	0.093	0.140	1.231
mmu-miR-199b-5p	0.498	0.091	0.073	0.113	0.223	0.093	0.140	1.231
mmu-miR-8112	0.498	0.091	0.073	0.113	0.223	0.093	0.140	1.231
mmu-miR-26a-2-3p	0.498	0.091	0.073	0.113	0.223	0.093	0.140	1.231
mmu-miR-1843b-5p	0.498	0.091	0.073	0.113	0.223	0.093	0.140	1.231
mmu-miR-32-5p	0.498	0.091	0.073	0.113	0.223	0.093	0.140	1.231
mmu-miR-345-5p	0.498	0.091	0.073	0.113	0.223	0.093	0.140	1.231
mmu-miR-487b-3p	0.498	0.091	0.073	0.113	0.223	0.093	0.140	1.231
mmu-miR-3082-5p	0.498	0.091	0.073	0.113	0.223	0.093	0.140	1.231
mmu-miR-6374	0.498	0.091	0.073	0.113	0.223	0.093	0.140	1.231
mmu-miR-551b-3p	0.498	0.091	0.073	0.113	0.223	0.093	0.140	1.231
mmu-miR-411-5p	0.498	0.091	0.073	0.113	0.223	0.093	0.140	1.231
mmu-miR-5126	0.498	0.091	0.073	0.113	0.223	0.085	0.140	1.223
mmu-miR-190a-5p	0.498	0.182	0.074	0.113	0.169	0.030	0.140	1.206
mmu-miR-369-5p	0.438	0.091	0.062	0.094	0.147	0.202	0.123	1.156
mmu-miR-3969	0.438	0.091	0.062	0.094	0.147	0.074	0.123	1.028
mmu-miR-219a-2-3p	0.438	0.091	0.062	0.094	0.147	0.027	0.123	0.982
mmu-miR-20b-5p	0.498	0.091	0.000	0.113	0.000	0.134	0.140	0.975
mmu-miR-7240-5p	0.438	0.000	0.062	0.094	0.197	0.047	0.123	0.959
mmu-miR-6966-5p	0.438	0.000	0.062	0.094	0.197	0.047	0.123	0.959
mmu-miR-3572-5p	0.438	0.000	0.062	0.094	0.197	0.047	0.123	0.959
mmu-miR-216a-5p	0.438	0.000	0.062	0.094	0.197	0.047	0.123	0.959
mmu-miR-216b-5p	0.438	0.000	0.062	0.094	0.197	0.047	0.123	0.959
mmu-miR-217-5p	0.438	0.000	0.062	0.094	0.197	0.047	0.123	0.959
mmu-miR-147-3p	0.498	0.091	0.000	0.113	0.000	0.087	0.140	0.928
mmu-miR-182-5p	0.498	0.091	0.000	0.113	0.000	0.080	0.140	0.922
mmu-miR-6239	0.498	0.091	0.000	0.113	0.000	0.078	0.140	0.919
mmu-miR-219c-5p	0.438	0.000	0.062	0.094	0.197	0.000	0.123	0.913
mmu-miR-505-3p	0.438	0.000	0.062	0.094	0.197	0.000	0.123	0.913
mmu-miR-5118	0.438	0.000	0.062	0.094	0.197	0.000	0.123	0.913
mmu-miR-210-5p	0.438	0.000	0.062	0.094	0.197	0.000	0.123	0.913
mmu-miR-466f-5p	0.438	0.000	0.062	0.094	0.197	0.000	0.123	0.913
mmu-miR-6914-3p	0.438	0.000	0.062	0.094	0.197	0.000	0.123	0.913
mmu-miR-130b-5p	0.438	0.000	0.062	0.094	0.197	0.000	0.123	0.913
mmu-miR-466f-3p	0.438	0.000	0.062	0.094	0.197	0.000	0.123	0.913
mmu-miR-5125	0.438	0.000	0.062	0.094	0.197	0.000	0.123	0.913
mmu-miR-706	0.438	0.000	0.062	0.094	0.197	0.000	0.123	0.913
mmu-miR-16-2-3p	0.438	0.000	0.062	0.094	0.197	0.000	0.123	0.913
mmu-miR-1968-5p	0.438	0.000	0.062	0.094	0.197	0.000	0.123	0.913
mmu-miR-3110-5p	0.438	0.000	0.062	0.094	0.197	0.000	0.123	0.913
mmu-miR-130a-5p	0.438	0.000	0.062	0.094	0.197	0.000	0.123	0.913
mmu-miR-6418-3p	0.438	0.000	0.062	0.094	0.197	0.000	0.123	0.913
mmu-miR-7667-3p	0.438	0.000	0.062	0.094	0.197	0.000	0.123	0.913
mmu-miR-1298-5p	0.438	0.000	0.062	0.094	0.197	0.000	0.123	0.913
mmu-miR-7118-5p	0.438	0.000	0.062	0.094	0.197	0.000	0.123	0.913
mmu-miR-6946-5p	0.438	0.000	0.062	0.094	0.197	0.000	0.123	0.913
Sex	0.000	0.000	0.003	0.000	0.004	0.420	0.000	0.427
Timepoint	0.000	0.000	0.003	0.000	0.004	0.374	0.000	0.380
