# Supplementary material for: A Panel of miRNA Biomarkers Common to Serum and Brain-Derived Extracellular Vesicles Identified in Mouse Model of Amyotrophic Lateral Sclerosis
Source: Mol Neurobiol. 2024 Jan 22;61(8):5901–15. doi: 10.1007/s12035-023-03857-z (PMC11249427; doi:10.1007/s12035-023-03857-z)
Supplement: Supplementary file 3 — Supplementary file3 (PDF 332 KB) [file 12035_2023_3857_MOESM3_ESM.pdf]

Sample na	Sample typ	Mutation	Sex	Timepoint	mmu-let-7	mmu-let-7	mmu-let-7	mmu-let-7	mmu-let-7	mmu-let-7	mmu-let-7	mmu-let-7	mmu-let-7	mmu-let-7	mmu-let-7	mmu-let-7	mmu-let-7	mmu-let-7	mmu-let-7	mmu-let-7	mmu-let-7	mmu-miR-	mmu-miR-	mmu-miR-	mmu-miR-
NV1 BDEV1	BDEVs	Q331K	Female	3m	50.7733	61957.8	18.1334	89313.6	21.76	25.3867	97970.4	105.173	15275.5	10.8801	31613.5	68304.4	43.5199	27533.6	65.2799	84323.3	47.1466	522.238	2698.23	39.8933	5160.73
NV2 BDEV2	BDEVs	Q331K	Female	3m	21.4645	60216.4	8.83837	72870.3	20.2019	40.4036	103082	59.3428	15393.8	15.1514	32678.9	89886.5	40.4036	31550.1	54.2924	90689.5	13.8888	465.903	2146.44	16.414	4707.01
NV4 BDEV4	BDEVs	Q331K	Male	3m	58.3055	61470.5	33.3175	83385.1	8.32944	49.9762	90723.2	141.599	13935	8.32944	34766.7	76205.1	74.9642	32059.6	91.6229	74914.1	24.9881	341.503	3415.03	49.9762	4572.81
NV5 BDEV5	BDEVs	Q331K	Male	3m	24.4187	62184.4	14.6513	87506.5	19.535	9.76754	95193.4	122.093	14231.2	34.1861	33424.2	80454.4	53.721	28823.7	58.6047	79560.7	29.3024	341.86	2349.07	9.76754	4522.32
NV6 BDEV6	BDEVs	Q331K	Male	3m	14.0533	68389.8	14.0533	97262.1	7.02669	28.1065	111308	126.479	15086.1	7.02669	33369.3	83075.4	28.1065	28282	35.1331	87994	14.0533	203.771	2297.7	28.1065	3140.89
NV7 BDEV7	BDEVs	WT	Female	3m	27.8167	61941.9	5.56341	100334	22.2534	22.2534	102276	105.703	15143.3	16.69	33324.2	80478.9	16.69	21724.7	55.6332	90309.3	38.9433	311.546	2153	11.1267	4968.04
NV8 BDEV8	BDEVs	WT	Female	3m	36.6657	57762.9	7.33321	105018	21.9994	0.0001	100090	168.662	14592.9	14.6663	30755.1	64252.8	14.6663	24221.3	21.9994	97295.8	0.0001	593.982	1202.63	14.6663	5844.49
NV17 BDEV	BDEVs	WT	Female	3m	12.5796	48368.1	12.5796	96409.2	0.0001	12.5796	92798.9	188.692	14629.9	12.5796	29649.8	59148.7	62.8975	18428.9	62.8975	96962.7	12.5796	264.169	3220.35	37.7386	5899.78
NV18 BDEV	BDEVs	WT	Male	3m	16.7481	55159.7	16.7481	84385	8.37412	25.1222	81127.5	192.602	17091.4	58.6182	29376.1	69906.3	75.3663	19352.4	108.862	81077.2	25.1222	393.579	3240.74	41.8702	5041.16
NV19 BDEV	BDEVs	WT	Male	3m	31.5042	60956.5	7.87613	74015	19.6902	43.3183	87136.5	90.5745	15929.3	35.4422	28814.5	79437.6	43.3183	39458.9	70.8844	91799.1	7.87613	311.103	3760.8	43.3183	5355.7
NV20 BDEV	BDEVs	WT	Male	3m	45.7313	63450.4	24.39	86364.8	33.5363	15.2438	103413	85.3649	15140.1	24.39	32850.2	80834.4	36.585	33197.8	73.1699	92788.5	18.2926	387.19	2536.55	18.2926	4353.61
NV21 BDEV	BDEVs	Q331K	Female	6m	49.2683	52383.1	16.4228	75270.9	27.3713	10.9486	83257.8	191.599	14380.8	5.47435	24469.9	69391.6	32.8456	25904.1	114.959	78955.1	10.9486	437.94	3875.77	21.8971	5342.87
NV22 BDEV	BDEVs	Q331K	Male	6m	17.9584	57466.6	53.8751	92099.2	44.8959	8.97926	96193.7	287.333	13971.6	8.97926	34138.8	70297.8	8.97926	26892.6	89.7917	75514.7	17.9584	287.333	3358.21	17.9584	4606.31
NV23 BDEV	BDEVs	Q331K	Male	6m	23.4399	56507.5	29.2998	84547.3	23.4399	11.72	96566.1	158.219	16038.7	17.5799	27934.4	75569.9	29.2998	24904.8	64.4595	77257.6	0.0001	591.855	3176.09	17.5799	5086.43
NV24 BDEV	BDEVs	Q331K	Male	6m	24.9036	47537.7	12.4518	58124.8	0.0001	28.0165	70090.9	87.1623	12096.9	21.7907	26740.1	69499.4	34.2424	23045.1	65.3718	60571.5	6.22597	675.507	5569.05	28.0165	7863.28
NV33 BDEV	BDEVs	Q331K	Male	6m	22.776	47920.4	34.1639	60581	14.235	37.0109	71234.4	176.513	12774.4	14.235	28526.8	68618	19.929	24911.1	173.666	56025.8	22.776	671.888	4942.36	45.5518	7575.82
NV34 BDEV	BDEVs	Q331K	Male	6m	32.1251	47794.5	9.8847	60165.1	7.41355	37.0674	69817.4	128.5	13220.7	14.827	26866.4	69558	12.3559	24076.4	59.3077	60553.1	19.7693	738.874	4097.17	24.7116	8236.35
NV35 BDEV	BDEVs	WT	Female	6m	13.336	62358.9	13.336	75837.1	35.5626	44.4532	87768.3	115.578	14513.9	22.2267	33682.1	83127.4	84.4611	34429	75.5704	82447.2	8.89073	431.196	2822.77	13.336	5912.27
NV36 BDEV	BDEVs	WT	Female	6m	16.7016	72617.9	33.403	110405	25.0523	16.7016	118939	175.365	17277.7	16.7016	40425.9	87407.1	8.35083	35649.3	41.7538	89912.3	8.35083	250.522	1912.32	8.35083	3123.17
NV37 BDEV	BDEVs	WT	Female	6m	19.139	65775.5	9.56953	86120.1	19.139	14.3543	95522.1	143.542	17593.4	28.7084	35607.9	84933.5	23.9237	31636.5	47.8473	73172.7	33.4931	531.104	2545.47	0.0001	5942.62
NV38 BDEV	BDEVs	WT	Male	6m	57.2757	62381.4	16.3646	82018.7	16.3646	32.729	90724.6	155.463	15718.1	16.3646	34038.1	74499.3	32.729	32303.5	65.458	79858.6	16.3646	327.289	2021.01	16.3646	5130.26
NV39 BDEV	BDEVs	WT	Male	6m	31.6732	59558.2	12.6694	75568.9	9.50204	34.8405	97683.1	63.3464	14664.7	6.33473	29456	82524.4	25.3386	30960.5	57.0117	84564.1	6.33473	582.786	2502.18	31.6732	5808.85
NV40 BDEV	BDEVs	WT	Male	6m	18.789	56549.3	18.789	68093.8	8.0525	24.1573	81589.6	77.84	14113.2	29.5256	31342.6	82818.9	45.6304	31425.8	34.8938	84539.5	10.7366	432.146	2555.3	16.1049	5341.43

Sample na	Sample typ	Mutation	Sex	Timepoint	mmu-miR-	mmu-miR-	mmu-miR-	mmu-miR-	mmu-miR-	mmu-miR-	mmu-miR-	mmu-miR-	mmu-miR-	mmu-miR-	mmu-miR-	mmu-miR-	mmu-miR-	mmu-miR-	mmu-miR-	mmu-miR-	mmu-miR-	mmu-miR-	mmu-miR-	mmu-miR-	mmu-miR-
NV1 BDEV1	BDEVs	Q331K	Female	3m	65.2799	1305.6	246.613	47.1466	0.0001	2078.07	72.5332	7.25341	54.3999	15170.3	319.146	366.292	21266.7	61.6532	18963.7	2441.46	28.2951	26336.8	279.252	90.6664	39.8933
NV2 BDEV2	BDEVs	Q331K	Female	3m	49.2419	1172.97	179.291	37.8784	2.52532	1878.76	98.4837	3.78793	75.7567	5677.96	348.481	417.924	10646.3	41.6663	17650	2217.93	25.7024	30108.2	311.865	51.7671	15.1514
NV4 BDEV4	BDEVs	Q331K	Male	3m	58.3055	899.569	283.198	41.6468	58.3055	1740.83	49.9762	0.0001	66.6348	19940.4	133.27	266.539	21573	49.9762	17241.7	1753.82	20.3267	25771	366.491	124.94	24.9881
NV5 BDEV5	BDEVs	Q331K	Male	3m	53.721	1010.93	219.767	29.3024	39.0699	996.279	34.1861	14.6513	14.6513	18323.7	273.488	346.744	22562.8	9.76754	24574.9	2627.18	88.1607	24604.2	200.233	87.907	39.0699
NV6 BDEV6	BDEVs	Q331K	Male	3m	77.2926	786.979	231.878	21.0799	7.02669	1004.8	63.2394	28.1065	84.3192	15043.9	309.17	295.117	17334.6	14.0533	16568.6	1583.71	18.3551	16224.4	140.532	56.2128	21.0799
NV7 BDEV7	BDEVs	WT	Female	3m	77.8865	1051.47	289.292	50.0699	22.2534	1218.37	44.5066	16.69	72.3232	16384	417.249	367.179	20072.4	44.5066	19655.2	2414.32	27.9813	27371.5	189.153	44.5066	61.1965
NV8 BDEV8	BDEVs	WT	Female	3m	161.329	967.971	212.66	51.3319	36.6657	1136.63	51.3319	36.6657	117.33	20408.1	337.323	293.325	26032.6	51.3319	14761.6	1602.05	18.5676	34509.6	263.992	102.664	29.3326
NV17 BDEV	BDEVs	WT	Female	3m	88.0565	1346.01	377.385	100.636	113.215	1899.5	113.215	25.1591	88.0565	18416.4	490.6	352.226	20718.4	0.0001	16177.2	1939.91	22.484	35335.8	314.487	213.851	50.318
NV18 BDEV	BDEVs	WT	Male	3m	41.8702	1163.99	284.717	50.2442	16.7481	1557.57	83.7403	33.4962	50.2442	18866.7	326.587	242.847	25850.6	75.3663	20809.4	1930.34	121.286	29409.5	343.335	100.488	50.2442
NV19 BDEV	BDEVs	WT	Male	3m	70.8844	1275.92	252.033	55.1323	11.8141	1969.01	55.1323	19.6902	43.3183	14425	220.529	330.793	20654.9	43.3183	18508.7	2461.76	121.582	21253.5	252.033	51.1943	7.87613
NV20 BDEV	BDEVs	WT	Male	3m	45.7313	1033.52	201.217	33.5363	24.39	1484.74	85.3649	6.09759	82.3162	16310.8	314.021	310.972	23005.8	36.585	20722.3	2137.9	96.8251	24575.9	231.705	91.4624	30.4875
NV21 BDEV	BDEVs	Q331K	Female	6m	60.2168	1187.91	268.238	32.8456	10.9486	1631.33	54.7426	54.7426	49.2683	20457.3	514.579	361.3	28882.1	87.5881	18836.9	3085.59	100.45	26112.2	246.341	71.1653	21.8971
NV22 BDEV	BDEVs	Q331K	Male	6m	35.9167	808.124	215.5	17.9584	53.8751	1822.77	35.9167	80.8125	80.8125	22456.9	296.312	242.437	25231.4	26.9376	17823.6	2265.12	132.321	19556.6	269.375	62.8542	35.9167
NV23 BDEV	BDEVs	Q331K	Male	6m	41.0197	1060.65	246.118	11.72	5.86005	1476.71	46.8797	0.0001	64.4595	12880.2	386.757	357.457	20222.7	117.199	16068	2306.61	95.9734	24242.6	240.258	41.0197	23.4399
NV24 BDEV	BDEVs	Q331K	Male	6m	115.179	1696.55	124.518	65.3718	3.11304	3053.79	68.4847	15.5648	115.179	17167.8	370.44	535.425	27453	46.6942	16464.3	4377.07	124.286	29121.5	270.826	62.2588	28.0165
NV33 BDEV	BDEVs	Q331K	Male	6m	74.0217	1876.16	105.338	51.2458	8.54105	3026.34	48.3988	25.6229	96.7975	23436.4	264.77	586.479	36153.8	62.6337	22710.3	3875.88	78.5725	27678.4	185.054	88.2566	22.776
NV34 BDEV	BDEVs	Q331K	Male	6m	116.144	2335.24	207.577	46.952	2.47125	4954.66	46.952	19.7693	123.558	18837.6	390.442	553.538	26285.6	46.952	20317.8	3776.39	189.731	33632.4	187.808	51.8943	24.7116
NV35 BDEV	BDEVs	WT	Female	6m	44.4532	1489.18	253.383	35.5626	4.44541	1022.42	102.242	8.89073	53.3439	12491.3	257.828	395.633	19426	75.5704	22395.5	3301.02	353.04	28076.6	208.93	102.242	44.4532
NV36 BDEV	BDEVs	WT	Female	6m	33.403	843.424	267.223	75.1567	50.1045	1235.91	33.403	16.7016	83.5074	16350.7	459.29	425.887	16142	0.0001	18580.4	1865.64	21.6226	17762	66.8059	58.4552	0.0001
NV37 BDEV	BDEVs	WT	Female	6m	90.9097	1669.87	291.868	47.8473	47.8473	2114.84	38.2778	23.9237	105.264	15292	406.701	454.548	22028.8	76.5556	18000.1	2173.77	194.66	30871	157.896	52.632	47.8473
NV38 BDEV	BDEVs	WT	Male	6m	73.6402	1251.88	261.832	139.098	16.3646	1939.19	57.2757	65.458	57.2757	20930.2	310.925	270.014	27263.2	16.3646	21511.1	2816.29	129.315	20226.5	130.916	90.0047	32.729
NV39 BDEV	BDEVs	WT	Male	6m	72.8483	1358.78	196.374	63.3464	6.33473	1827.54	85.5176	15.8367	57.0117	7721.91	389.58	386.412	15798.6	34.8405	20454.5	3447.84	77.4011	24762.1	164.7	69.681	15.8367
NV40 BDEV	BDEVs	WT	Male	6m	69.7876	1154.18	246.94	83.2082	16.1049	1589.01	72.4717	16.1049	77.84	10607.7	418.725	469.723	18117.9	42.9462	25416.1	5139.31	218.138	27308.4	217.415	104.681	42.9462

Sample na	Sample typ	Mutation	Sex	Timepoint	mmu-miR-	mmu-miR-	mmu-miR-	mmu-miR-	mmu-miR-	mmu-miR-	mmu-miR-	mmu-miR-	mmu-miR-	mmu-miR-	mmu-miR-	mmu-miR-	mmu-miR-	mmu-miR-	mmu-miR-	mmu-miR-	mmu-miR-	mmu-miR-	mmu-miR-	mmu-miR-	mmu-miR-
NV1 BDEV1	BDEVs	Q331K	Female	3m	35156.8	39.8933	570.454	1457.55	61.6532	181.333	101.546	274.557	337.279	25.3867	2110.71	417.065	21.76	932.05	413.439	246.613	2802.73	1218.47	12004.2	54.3999	58.0266
NV2 BDEV2	BDEVs	Q331K	Female	3m	28753.4	22.7271	195.965	1753.62	31.5654	234.846	155.301	109.587	202.018	26.5149	1611.09	534.084	21.4645	1065.64	434.338	185.604	3033.16	1392.59	17653.8	32.828	31.5654
NV4 BDEV4	BDEVs	Q331K	Male	3m	30785.2	49.9762	608.085	1574.25	66.6348	224.892	366.491	274.826	299.856	16.6588	1590.9	291.527	16.6588	724.653	366.491	158.258	2398.41	924.557	16092.3	58.3055	74.9642
NV5 BDEV5	BDEVs	Q331K	Male	3m	33170.2	48.8373	234.419	1864.38	43.9536	131.861	19.535	234.419	268.605	24.4187	1680	214.884	14.6513	986.511	317.442	117.209	2011.61	703.256	14934.4	73.2559	53.721
NV6 BDEV6	BDEVs	Q331K	Male	3m	24480.7	77.2926	140.719	1517.74	42.1597	112.426	126.479	400.329	316.197	0.0001	1489.64	168.638	28.1065	997.776	245.931	196.745	1348.84	639.42	8312.46	21.0799	35.1331
NV7 BDEV7	BDEVs	WT	Female	3m	33624.7	66.7599	372.833	2223.8	61.1965	166.899	228.096	155.682	289.292	5.56341	1841.46	506.262	22.2534	734.357	239.223	150.21	2124.54	728.794	8951.37	38.9433	38.9433
NV8 BDEV8	BDEVs	WT	Female	3m	37340.2	65.9981	519.462	2603.26	95.3306	249.326	571.983	228.516	205.327	7.33321	1796.61	461.986	14.6663	931.306	197.994	197.994	1297.96	916.639	9569.71	95.3306	95.3306
NV17 BDEV	BDEVs	WT	Female	3m	31373.2	62.8975	538.1	2059.39	25.1591	213.851	88.0565	128.613	314.487	25.1591	1295.69	402.544	25.1591	893.144	188.692	213.851	3043.4	792.508	8554.05	50.318	50.318
NV18 BDEV	BDEVs	WT	Male	3m	33688.7	83.7403	156.925	1932.88	8.37412	167.48	385.205	462.752	309.839	16.7481	1390.09	217.725	0.0001	711.792	318.213	133.984	2202	753.569	12912.7	33.4962	25.1222
NV19 BDEV	BDEVs	WT	Male	3m	32173.6	118.141	294.62	1400.89	11.8141	181.149	169.335	429.975	334.731	11.8141	1539.76	397.74	15.7522	700.967	480.438	252.033	2142.16	1327.11	10979.2	59.0703	51.1943
NV20 BDEV	BDEVs	WT	Male	3m	35953.8	57.9262	230.934	1947.8	45.7313	112.804	85.3649	336.132	185.973	18.2926	1615.83	314.021	30.4875	548.774	292.679	195.12	1886.83	877.975	16569.9	64.0237	67.0725
NV21 BDEV	BDEVs	Q331K	Female	6m	33475	76.6396	558.633	2216.36	38.3198	421.517	832.086	322.721	246.341	10.9486	1521.84	416.043	32.8456	914.199	394.146	93.0623	2960.93	1072.68	16504.9	60.2168	43.7941
NV22 BDEV	BDEVs	Q331K	Male	6m	32351.9	17.9584	300.802	1732.98	35.9167	215.5	44.8959	300.802	287.333	8.97926	2119.08	305.291	8.97926	709.354	305.291	143.667	1984.39	1266.06	14968.3	44.8959	62.8542
NV23 BDEV	BDEVs	Q331K	Male	6m	25109.9	46.8797	313.507	1740.4	58.5996	240.258	820.393	313.507	328.157	35.1598	1658.37	298.857	35.1598	914.152	275.418	275.418	2390.69	1517.73	11942.6	46.8797	17.5799
NV24 BDEV	BDEVs	Q331K	Male	6m	33376.9	80.9365	194.01	1739.68	34.2424	71.5977	68.4847	441.03	382.891	46.6942	1908.23	507.409	52.92	1083.3	494.957	305.068	3728.64	6135.48	9435.31	59.1459	77.8235
NV33 BDEV	BDEVs	Q331K	Male	6m	43476.3	48.3988	196.689	1285.73	17.082	54.0928	71.1747	321.461	418.507	54.0928	2351.61	412.813	31.3169	814.237	330.25	244.841	2812.4	4814.18	8757.32	59.7867	76.8686
NV34 BDEV	BDEVs	Q331K	Male	6m	35137.3	84.0192	418.86	1425.43	14.827	54.3654	71.6635	418.86	395.384	37.0674	1939.85	709.22	34.5962	827.836	642.499	326.192	3728.51	7158.8	9111.13	44.4808	76.6058
NV35 BDEV	BDEVs	WT	Female	6m	40514.6	17.7814	144.473	1755	31.1173	120.024	431.196	144.473	213.375	8.89073	1431.39	320.063	44.4532	702.36	213.375	137.805	2324.59	893.464	17061.1	44.4532	71.1251
NV36 BDEV	BDEVs	WT	Female	6m	26129.4	66.8059	204.593	1778.71	16.7016	66.8059	133.612	204.593	150.313	25.0523	1494.78	267.223	8.35083	584.551	150.313	108.56	1310.79	1027.14	11323.6	66.8059	41.7538
NV37 BDEV	BDEVs	WT	Female	6m	34646.1	66.9861	366.031	1569.39	33.4931	114.833	263.16	366.031	177.035	0.0001	1856.47	444.979	23.9237	674.645	277.514	157.896	1989.6	1253.6	20655.6	105.264	86.125
NV38 BDEV	BDEVs	WT	Male	6m	35290	40.9113	214.258	1358.25	65.458	40.9113	73.6402	530.325	163.645	0.0001	2119.2	270.014	40.9113	818.224	278.196	155.463	1414.54	998.166	18262.7	122.734	49.0935
NV39 BDEV	BDEVs	WT	Male	6m	32148.2	44.3425	88.6331	1384.12	19.004	193.206	193.206	212.262	338.903	19.004	1884.55	367.408	25.3386	497.268	383.245	199.541	2346.53	1162.39	17588.1	47.5098	53.8444
NV40 BDEV	BDEVs	WT	Male	6m	32325	48.3145	163.398	1826.81	48.3145	185.205	212.047	279.484	276.466	13.4208	1315.23	391.884	34.8938	735.453	241.572	150.312	2598.02	1784.95	14454.1	34.8938	50.9986

Sample na	Sample typ	Mutation	Sex	Timepoint	mmu-miR-	mmu-miR-	mmu-miR-	mmu-miR-	mmu-miR-	mmu-miR-	mmu-miR-	mmu-miR-	mmu-miR-	mmu-miR-	mmu-miR-	mmu-miR-	mmu-miR-	mmu-miR-	mmu-miR-	mmu-miR-	mmu-miR-	mmu-miR-	mmu-miR-	mmu-miR-	mmu-miR-
NV1 BDEV1	BDEVs	Q331K	Female	3m	101.546	2393.59	119.68	275.626	406.185	97.9198	217.599	710.824	65.2799	14655.3	141.44	29.0133	500.478	2749	116.053	641.918	768.851	21.76	1320.1	874.024	83.4131
NV2 BDEV2	BDEVs	Q331K	Female	3m	77.0194	3583.29	84.595	143.938	421.712	63.1306	37.8784	695.699	36.6158	10831.9	64.3932	10.101	247.472	2541.64	146.463	446.964	790.394	6.31315	654.033	297.976	18.9393
NV4 BDEV4	BDEVs	Q331K	Male	3m	74.9642	1874.1	283.198	174.916	458.114	66.6348	966.204	966.204	41.6468	7646.34	66.6348	0.0001	399.809	2398.85	66.6348	341.503	791.288	0.0001	1557.59	757.97	74.9642
NV5 BDEV5	BDEVs	Q331K	Male	3m	92.7908	1553.02	263.721	146.512	278.372	83.0233	576.279	688.604	107.442	11525.6	97.6745	24.4187	268.605	2886.28	156.279	444.418	630	19.535	1206.28	883.953	58.6047
NV6 BDEV6	BDEVs	Q331K	Male	3m	77.2926	1278.84	161.612	168.638	259.984	35.1331	3787.33	892.377	63.2394	8284.35	63.2394	7.02669	259.984	2705.24	84.3192	442.675	435.649	14.0533	976.697	611.314	35.1331
NV7 BDEV7	BDEVs	WT	Female	3m	77.8865	1874.84	133.52	189.153	350.489	55.6332	0.0001	356.052	33.38	10236.5	55.6332	16.69	216.969	2336.59	144.646	233.659	617.528	22.2534	1240.62	578.585	44.5066
NV8 BDEV8	BDEVs	WT	Female	3m	168.662	2661.92	344.656	256.659	315.324	80.6644	21.9994	513.318	14.6663	10178.4	29.3326	58.665	307.991	3028.58	183.328	175.995	777.31	14.6663	1598.62	1055.97	131.996
NV17 BDE\	BDEVs	WT	Female	3m	37.7386	3107.13	201.272	150.954	289.328	37.7386	666.713	515.759	50.318	9610.73	50.318	12.5796	427.703	1798.87	88.0565	289.328	641.554	25.1591	993.78	628.974	113.215
NV18 BDE\	BDEVs	WT	Male	3m	133.984	2110.25	267.969	184.228	435.449	41.8702	829.028	653.173	66.9922	7670.6	58.6182	16.7481	259.595	2755.05	125.61	343.335	686.669	0.0001	854.15	502.441	83.7403
NV19 BDE\	BDEVs	WT	Male	3m	66.9464	2732.98	129.955	279.599	370.174	78.7604	259.909	929.372	74.8224	14633.7	63.0084	23.6282	267.785	2244.67	122.079	417.43	858.487	15.7522	1350.74	681.277	31.5042
NV20 BDE\	BDEVs	WT	Male	3m	79.2674	1926.81	225.607	231.705	210.363	85.3649	30.4875	667.675	54.8775	6624.92	27.4388	18.2926	167.681	2048.76	115.852	530.481	707.309	6.09759	1134.13	628.041	48.78
NV21 BDE\	BDEVs	Q331K	Female	6m	125.908	2310.13	229.918	213.496	399.62	38.3198	312.032	717.126	109.485	11370	71.1653	16.4228	295.609	2490.78	65.6911	503.631	908.725	5.47435	1445.2	777.343	76.6396
NV22 BDE\	BDEVs	Q331K	Male	6m	125.708	1984.39	296.312	260.396	332.229	98.7708	2038.27	727.312	107.75	9410.16	26.9376	35.9167	215.5	2181.94	53.8751	395.083	754.25	17.9584	1427.69	942.812	62.8542
NV23 BDE\	BDEVs	Q331K	Male	6m	105.479	2021.68	158.219	134.779	550.835	64.4595	3041.31	943.452	111.339	10237.3	58.5996	46.8797	492.236	2824.49	152.359	644.594	603.575	17.5799	873.132	574.275	64.4595
NV24 BDE\	BDEVs	Q331K	Male	6m	93.3882	2698.92	140.082	361.101	647.491	59.1459	199.228	999.253	93.3882	19206.8	56.033	34.2424	401.569	3679.49	171.212	591.458	800.025	18.6777	1055.29	1484.87	21.7907
NV33 BDE\	BDEVs	Q331K	Male	6m	130.961	2269.05	190.748	492.528	629.183	68.3277	688.97	996.444	111.032	12219.3	42.7049	28.4699	455.517	3097.52	102.491	686.123	851.248	11.388	948.046	1816.38	37.0109
NV34 BDE\	BDEVs	Q331K	Male	6m	143.327	3200.14	195.221	444.807	578.249	103.788	2377.25	800.653	74.1346	24444.6	61.7789	56.8366	558.48	3437.37	187.808	454.692	657.326	7.41355	1196.04	1905.26	44.4808
NV35 BDE\	BDEVs	WT	Female	6m	128.914	2009.28	173.367	248.938	480.094	115.578	4.44541	733.477	31.1173	9548.53	62.2345	44.4532	231.156	2556.06	133.36	306.727	675.688	35.5626	680.133	729.032	26.672
NV36 BDE\	BDEVs	WT	Female	6m	141.963	1144.05	417.537	258.873	208.768	83.5074	517.745	835.073	25.0523	9762	25.0523	8.35083	308.977	2045.93	91.8581	417.537	551.148	16.7016	1185.8	1144.05	108.56
NV37 BDE\	BDEVs	WT	Female	6m	119.618	2200.97	387.562	373.208	478.472	57.4167	14.3543	540.673	47.8473	8378.04	43.0626	4.78482	272.729	2090.92	157.896	358.854	655.506	14.3543	1172.26	913.881	181.819
NV38 BDE\	BDEVs	WT	Male	6m	106.369	1849.19	425.476	310.925	237.285	57.2757	540.028	687.308	81.8225	10219.6	8.18234	0.0001	278.196	1881.91	98.1869	351.836	646.397	16.3646	1440.07	1243.7	57.2757
NV39 BDE\	BDEVs	WT	Male	6m	57.0117	2622.54	91.8522	186.872	421.253	91.8522	313.564	775.992	50.6771	13524.4	79.1829	19.004	319.899	2058.75	158.366	443.424	696.809	31.6732	715.813	462.428	9.50204
NV40 BDE\	BDEVs	WT	Male	6m	83.2082	2297.62	225.467	228.151	424.093	59.051	29.5256	735.453	50.9986	21701.2	85.8924	101.997	346.253	2455.98	158.364	340.885	550.247	16.1049	920.658	848.186	34.8938

Sample na	Sample typ	Mutation	Sex	Timepoint	mmu-miR-	mmu-miR-	mmu-miR-	mmu-miR-	mmu-miR-	mmu-miR-	mmu-miR-	mmu-miR-	mmu-miR-	mmu-miR-	mmu-miR-	mmu-miR-	mmu-miR-	mmu-miR-	mmu-miR-	mmu-miR-	mmu-miR-	mmu-miR-	mmu-miR-	mmu-miR-	mmu-miR-
NV1 BDEV1	BDEVs	Q331K	Female	3m	174.079	340.906	7.25341	1287.46	32.64	2284.79	54.3999	395.305	68.9065	11006.9	21.76	21.76	576.638	1900.37	649.171	119.68	137.813	174.079	366.292	217.599	489.598
NV2 BDEV2	BDEVs	Q331K	Female	3m	171.715	239.896	6.31315	1551.75	16.414	3114.86	35.3532	398.985	58.0802	10816.8	11.3636	12.6262	638.881	1919.17	906.555	188.129	104.797	237.371	51.7671	61.868	628.78
NV4 BDEV4	BDEVs	Q331K	Male	3m	116.611	241.551	0.0001	1882.43	0.0001	1849.11	16.6588	349.832	124.94	12077.5	16.6588	0.0001	308.186	2040.69	624.701	108.282	83.2935	208.234	1515.94	1549.26	341.503
NV5 BDEV5	BDEVs	Q331K	Male	3m	73.2559	244.186	19.535	1484.65	14.6513	1509.07	34.1861	332.093	34.1861	13034.6	9.76754	19.535	444.418	1640.93	571.395	117.209	58.6047	112.326	1421.16	1274.65	390.698
NV6 BDEV6	BDEVs	Q331K	Male	3m	126.479	154.585	7.02669	1545.85	0.0001	1208.57	35.1331	358.356	91.3458	13828.3	21.0799	14.0533	330.25	2157.16	864.271	147.559	70.266	231.878	4820.24	7602.77	386.463
NV7 BDEV7	BDEVs	WT	Female	3m	139.083	205.843	0.0001	929.073	16.69	1813.64	27.8167	255.912	72.3232	12133.6	16.69	0.0001	506.262	2175.26	795.554	139.083	111.266	233.659	38.9433	27.8167	422.812
NV8 BDEV8	BDEVs	WT	Female	3m	95.3306	278.658	36.6657	806.643	0.0001	2559.26	29.3326	256.659	58.665	8191.09	0.0001	14.6663	564.65	2148.6	601.316	161.329	36.6657	197.994	36.6657	65.9981	388.655
NV17 BDEV	BDEVs	WT	Female	3m	100.636	150.954	25.1591	1698.23	12.5796	3736.11	0.0001	327.067	25.1591	12063.7	25.1591	12.5796	478.021	2503.32	704.451	264.169	75.477	150.954	1622.75	1182.47	540.918
NV18 BDEV	BDEVs	WT	Male	3m	100.488	234.473	16.7481	1683.18	8.37412	2704.81	50.2442	326.587	66.9922	13398.4	33.4962	8.37412	443.823	2034.89	644.799	83.7403	66.9922	159.106	1649.68	1507.32	502.441
NV19 BDEV	BDEVs	WT	Male	3m	114.203	295.351	7.87613	2496.7	23.6282	2701.48	27.5662	523.756	82.6984	13889.4	7.87613	15.7522	366.236	2028.08	500.128	129.955	114.203	149.645	444.996	315.041	496.19
NV20 BDEV	BDEVs	WT	Male	3m	161.583	219.51	21.3413	1859.73	21.3413	2048.76	33.5363	341.459	76.2187	15002.9	3.04884	33.5363	390.239	1972.54	783.527	88.4137	67.0725	167.681	94.5111	118.901	411.58
NV21 BDEV	BDEVs	Q331K	Female	6m	109.485	312.032	5.47435	1959.78	16.4228	2271.81	38.3198	262.764	120.434	13723.9	27.3713	10.9486	454.363	2009.05	656.91	197.073	136.856	136.856	388.672	328.455	509.105
NV22 BDEV	BDEVs	Q331K	Male	6m	89.7917	368.146	17.9584	1759.92	26.9376	1409.73	35.9167	386.104	62.8542	13899.7	17.9584	8.97926	413.041	2137.04	610.583	98.7708	53.8751	89.7917	3896.96	3430.04	502.833
NV23 BDEV	BDEVs	Q331K	Male	6m	169.939	439.496	11.72	1418.11	29.2998	1875.18	35.1598	398.476	111.339	13196.6	0.0001	0.0001	562.555	2015.82	796.953	140.639	169.939	169.939	4037.5	3808.97	410.196
NV24 BDEV	BDEVs	Q331K	Male	6m	189.889	351.762	0.0001	2359.61	3.11304	1839.75	24.9036	619.475	130.743	22036.5	12.4518	3.11304	463.828	1431.95	507.409	208.567	80.9365	186.776	370.44	261.487	488.731
NV33 BDEV	BDEVs	Q331K	Male	6m	279.004	350.179	2.84708	2007.12	17.082	1722.42	42.7049	606.408	148.043	25184.4	2.84708	45.5518	663.347	1201.43	409.966	133.808	68.3277	145.196	1135.95	993.597	387.19
NV34 BDEV	BDEVs	Q331K	Male	6m	158.154	306.423	9.8847	2046.11	37.0674	2033.76	34.5962	506.586	84.0192	18696.7	22.2405	9.8847	625.201	2078.24	699.336	210.048	106.26	219.933	3627.65	3407.72	437.394
NV35 BDEV	BDEVs	WT	Female	6m	195.594	226.711	17.7814	1893.7	8.89073	2640.52	22.2267	417.86	84.4611	16936.6	0.0001	13.336	524.547	1200.23	511.211	124.469	53.3439	142.25	31.1173	13.336	395.633
NV36 BDEV	BDEVs	WT	Female	6m	158.664	141.963	0.0001	1160.75	16.7016	1219.21	25.0523	267.223	66.8059	14446.8	0.0001	16.7016	250.522	1736.95	759.917	83.5074	66.8059	150.313	1336.12	1386.22	292.276
NV37 BDEV	BDEVs	WT	Female	6m	157.896	330.146	9.56953	1531.11	23.9237	2526.33	38.2778	224.882	62.2014	12976.2	9.56953	14.3543	574.166	2110.06	846.895	138.757	95.6944	200.958	66.9861	43.0626	598.09
NV38 BDEV	BDEVs	WT	Male	6m	106.369	310.925	8.18234	1980.1	8.18234	1841	16.3646	376.383	57.2757	14539.8	8.18234	0.0001	368.201	2397.39	736.401	106.369	147.28	253.649	1194.61	965.504	351.836
NV39 BDEV	BDEVs	WT	Male	6m	126.693	319.899	12.6694	1713.52	22.1713	2831.58	28.5059	392.747	76.0156	14506.3	19.004	19.004	478.264	2327.98	855.175	139.362	110.856	177.37	528.941	465.595	668.303
NV40 BDEV	BDEVs	WT	Male	6m	128.839	209.363	18.789	1991.63	18.789	2198.31	37.578	305.991	72.4717	15865.9	8.0525	2.68423	523.406	1835.95	681.77	209.363	115.418	182.521	72.4717	37.578	493.881

Sample nai	Sample typ	Mutation	Sex	Timepoint	mmu-miR-	mmu-miR-	mmu-miR-	mmu-miR-	mmu-miR-	mmu-miR-	mmu-miR-	mmu-miR-	mmu-miR-	mmu-miR-	mmu-miR-	mmu-miR-	mmu-miR-	mmu-miR-	mmu-miR-	mmu-miR-	mmu-miR-	mmu-miR-	mmu-miR-	mmu-miR-	mmu-miR-
NV1 BDEV1	BDEVs	Q331K	Female	3m	14.5067	199.466	177.706	116.053	61.6532	1581.22	14.5067	435.199	1911.25	54.3999	1428.9	43.5199	3764.47	239.359	7.25341	10.8801	145.066	18.1334	721.704	14.5067	134.186
NV2 BDEV2	BDEVs	Q331K	Female	3m	32.828	193.18	282.825	204.543	13.8888	1872.45	7.57576	405.298	2489.87	25.2523	1820.68	21.4645	2440.63	295.451	8.83837	13.8888	128.786	5.05054	748.728	1.26271	88.3829
NV4 BDEV4	BDEVs	Q331K	Male	3m	33.3175	199.904	141.599	99.9522	33.3175	1665.87	16.6588	191.575	2298.9	74.9642	1532.6	33.3175	3331.74	191.575	8.32944	41.6468	66.6348	16.6588	732.982	41.6468	133.27
NV5 BDEV5	BDEVs	Q331K	Male	3m	14.6513	322.326	136.744	63.4884	19.535	1367.44	24.4187	224.651	1430.93	34.1861	1191.63	9.76754	3428.37	205.116	0.0001	9.76754	141.628	9.76754	732.558	19.535	126.977
NV6 BDEV6	BDEVs	Q331K	Male	3m	42.1597	217.824	133.505	133.505	63.2394	955.617	14.0533	175.665	1419.37	28.1065	920.484	7.02669	2417.15	175.665	7.02669	14.0533	77.2926	7.02669	681.58	0.0001	91.3458
NV7 BDEV7	BDEVs	WT	Female	3m	44.5066	272.602	239.223	144.646	61.1965	1335.2	11.1267	250.349	1535.47	22.2534	1095.97	11.1267	3137.71	267.039	5.56341	0.0001	100.14	22.2534	584.148	5.56341	77.8865
NV8 BDEV8	BDEVs	WT	Female	3m	43.9988	447.32	241.993	161.329	43.9988	1796.61	7.33321	315.324	2683.92	14.6663	1620.62	0.0001	2991.91	153.995	7.33321	14.6663	43.9988	7.33321	403.321	7.33321	190.661
NV17 BDEV	BDEVs	WT	Female	3m	37.7386	327.067	239.01	163.533	50.318	1358.58	12.5796	301.908	2654.27	50.318	981.2	37.7386	2868.12	188.692	25.1591	37.7386	75.477	0.0001	503.18	0.0001	163.533
NV18 BDEV	BDEVs	WT	Male	3m	33.4962	326.587	259.595	150.732	25.1222	1323.09	8.37412	217.725	2152.12	92.1143	1314.72	25.1222	3056.52	117.236	0.0001	33.4962	83.7403	16.7481	820.654	16.7481	200.977
NV19 BDEV	BDEVs	WT	Male	3m	15.7522	185.087	303.227	118.141	15.7522	2232.86	15.7522	271.723	2563.65	31.5042	1303.48	7.87613	2882.63	224.467	7.87613	0.0001	118.141	7.87613	937.248	7.87613	106.327
NV20 BDEV	BDEVs	WT	Male	3m	54.8775	234.753	231.705	173.778	33.5363	1423.76	24.39	195.12	1868.88	36.585	1140.23	12.1951	3582.27	231.705	15.2438	9.14633	109.755	3.04884	942.062	15.2438	109.755
NV21 BDEV	BDEVs	Q331K	Female	6m	5.47435	361.3	213.496	82.1138	54.7426	1406.88	10.9486	295.609	2249.92	98.5366	1510.89	10.9486	4182.32	262.764	21.8971	16.4228	147.805	5.47435	733.549	10.9486	175.176
NV22 BDEV	BDEVs	Q331K	Male	6m	26.9376	332.229	98.7708	116.729	35.9167	1499.52	0.0001	215.5	2011.33	62.8542	1571.35	17.9584	4139.39	278.354	8.97926	0.0001	107.75	0.0001	664.458	8.97926	278.354
NV23 BDEV	BDEVs	Q331K	Male	6m	58.5996	275.418	175.799	146.499	5.86005	1388.81	11.72	345.737	2009.96	76.1794	1207.15	17.5799	3305.01	234.398	17.5799	5.86005	128.919	29.2998	855.552	11.72	128.919
NV24 BDEV	BDEVs	Q331K	Male	6m	34.2424	130.743	236.583	102.727	6.22597	1752.58	34.2424	479.392	1780.6	40.4683	613.249	21.7907	3928.53	466.941	3.11304	9.33891	277.051	15.5648	1266.97	24.9036	133.856
NV33 BDEV	BDEVs	Q331K	Male	6m	48.3988	156.584	290.392	108.185	14.235	1824.92	37.0109	469.752	1249.83	45.5518	660.5	28.4699	5153.04	486.834	14.235	11.388	236.3	2.84708	1523.14	14.235	142.349
NV34 BDEV	BDEVs	Q331K	Male	6m	42.0097	163.096	269.356	123.558	24.7116	1791.58	29.6539	459.634	1974.45	46.952	766.057	7.41355	4275.09	415.153	17.2982	9.8847	244.644	2.47125	1294.88	14.827	133.442
NV35 BDEV	BDEVs	WT	Female	6m	31.1173	288.945	320.063	137.805	17.7814	1822.58	40.0079	395.633	1675.88	31.1173	1235.8	26.672	3938.55	315.617	13.336	22.2267	128.914	13.336	946.852	31.1173	177.813
NV36 BDEV	BDEVs	WT	Female	6m	58.4552	242.171	275.574	116.91	41.7538	1227.56	16.7016	242.171	1494.78	100.209	1169.1	25.0523	3198.33	250.522	0.0001	8.35083	83.5074	0.0001	784.969	0.0001	192.067
NV37 BDEV	BDEVs	WT	Female	6m	52.632	363.639	239.236	191.389	33.4931	1550.25	14.3543	368.423	1746.42	47.8473	1808.62	4.78482	5177.06	263.16	9.56953	9.56953	71.7709	9.56953	655.506	14.3543	215.312
NV38 BDEV	BDEVs	WT	Male	6m	49.0935	188.191	261.832	188.191	24.5468	1751	8.18234	302.743	2462.85	57.2757	1464.62	0.0001	3624.73	196.374	8.18234	24.5468	122.734	8.18234	891.864	16.3646	278.196
NV39 BDEV	BDEVs	WT	Male	6m	25.3386	221.712	342.07	221.712	9.50204	1501.31	15.8367	345.237	2020.75	31.6732	1320.77	15.8367	3031.12	281.891	15.8367	12.6694	129.86	6.33473	969.198	6.33473	79.1829
NV40 BDEV	BDEVs	WT	Male	6m	26.8414	220.099	322.096	193.258	24.1573	1728.58	2.68423	434.83	1900.37	45.6304	818.661	21.4732	2982.07	316.728	8.0525	8.0525	123.47	10.7366	866.975	5.36837	120.786

Sample na	Sample typ	Mutation	Sex	Timepoint	mmu-miR-	mmu-miR-	mmu-miR-	mmu-miR-	mmu-miR-	mmu-miR-	mmu-miR-	mmu-miR-	mmu-miR-	mmu-miR-	mmu-miR-	mmu-miR-	mmu-miR-	mmu-miR-	mmu-miR-	mmu-miR-	mmu-miR-	mmu-miR-	mmu-miR-	mmu-miR-	mmu-miR-
NV1 BDEV1	BDEVs	Q331K	Female	3m	29.0133	228.479	47.1466	206.719	21.76	134.186	47.1367	460.596	932.05	500.478	10.8801	94.2931	290.132	32.64	562.131	32.64	76.1598	203.093	32.64	224.853	301.012
NV2 BDEV2	BDEVs	Q331K	Female	3m	37.8784	141.412	21.4645	137.625	17.6767	260.098	125.841	678.441	119.948	60.6054	0.0001	17.6767	171.715	20.2019	319.441	23.9897	85.8576	171.715	23.9897	131.312	267.674
NV4 BDEV4	BDEVs	Q331K	Male	3m	49.9762	116.611	8.32944	108.282	66.6348	166.587	40.9834	400.472	3631.59	1957.4	41.6468	774.629	141.599	133.27	832.934	49.9762	83.2935	233.222	41.6468	274.868	158.258
NV5 BDEV5	BDEVs	Q331K	Male	3m	24.4187	117.209	9.76754	83.0233	14.6513	83.0233	56.2205	549.361	2607.91	1045.12	43.9536	429.767	244.186	34.1861	317.442	43.9536	87.907	151.395	9.76754	107.442	170.93
NV6 BDEV6	BDEVs	Q331K	Male	3m	21.0799	147.559	14.0533	168.638	28.1065	175.665	35.2256	344.21	13231.1	7265.5	231.878	4089.48	217.824	14.0533	470.782	21.0799	28.1065	175.665	21.0799	126.479	210.798
NV7 BDEV7	BDEVs	WT	Female	3m	33.38	139.083	16.69	55.6332	33.38	61.1965	37.1865	363.372	161.336	27.8167	0.0001	22.2534	161.336	27.8167	534.078	33.38	66.7599	122.393	11.1267	178.026	328.236
NV8 BDEV8	BDEVs	WT	Female	3m	51.3319	117.33	21.9994	131.996	7.33321	175.995	36.7623	359.226	161.329	29.3326	0.0001	14.6663	183.328	65.9981	1283.3	36.6657	95.3306	58.665	21.9994	161.329	197.994
NV17 BDEV	BDEVs	WT	Female	3m	25.1591	100.636	50.318	75.477	88.0565	113.215	26.8603	262.468	2842.96	1358.58	75.477	566.077	163.533	50.318	515.759	37.7386	88.0565	88.0565	25.1591	201.272	226.431
NV18 BDEV	BDEVs	WT	Male	3m	66.9922	92.1143	33.4962	108.862	8.37412	108.862	45.8676	448.199	3676.19	1155.61	33.4962	494.067	251.221	58.6182	695.043	41.8702	58.6182	159.106	25.1222	83.7403	108.862
NV19 BDEV	BDEVs	WT	Male	3m	35.4422	133.893	27.5662	149.645	35.4422	141.769	134.056	464.522	1051.45	441.058	7.87613	145.707	240.219	23.6282	594.64	11.8141	63.0084	200.839	35.4422	232.343	200.839
NV20 BDEV	BDEVs	WT	Male	3m	12.1951	67.0725	15.2438	91.4624	18.2926	67.0725	51.7963	506.125	185.973	112.804	0.0001	36.585	271.338	33.5363	350.606	12.1951	103.657	124.999	24.39	195.12	195.12
NV21 BDEV	BDEVs	Q331K	Female	6m	49.2683	268.238	60.2168	235.393	10.9486	164.228	42.1815	412.182	1330.24	541.951	43.7941	202.547	262.764	109.485	1182.44	87.5881	98.5366	208.022	43.7941	197.073	197.073
NV22 BDEV	BDEVs	Q331K	Male	6m	0.0001	215.5	26.9376	152.646	26.9376	80.8125	40.0128	390.987	8036.35	3645.54	152.646	1328.92	287.333	35.9167	493.854	53.8751	53.8751	116.729	26.9376	233.458	269.375
NV23 BDEV	BDEVs	Q331K	Male	6m	29.2998	334.017	29.2998	316.437	11.72	134.779	41.3454	404.01	12886	4547.32	99.6192	1851.74	193.378	87.8993	1506.01	82.0394	64.4595	181.658	29.2998	128.919	228.538
NV24 BDEV	BDEVs	Q331K	Male	6m	37.3553	164.986	46.6942	161.873	21.7907	205.454	89.0138	869.774	1064.62	264.6	12.4518	90.2753	270.826	43.5812	600.797	46.6942	62.2588	186.776	18.6777	130.743	273.939
NV33 BDEV	BDEVs	Q331K	Male	6m	56.9398	213.524	28.4699	136.655	25.6229	136.655	70.8359	692.157	2895.38	1101.78	14.235	304.627	219.218	48.3988	788.614	25.6229	82.5626	176.513	11.388	236.3	113.879
NV34 BDEV	BDEVs	Q331K	Male	6m	39.5385	168.038	32.1251	148.269	7.41355	175.452	67.9087	663.554	9083.95	2515.63	64.25	1000.82	244.644	37.0674	800.653	42.0097	59.3077	163.096	39.5385	214.99	331.134
NV35 BDEV	BDEVs	WT	Female	6m	17.7814	88.9064	26.672	102.242	8.89073	93.3517	79.6503	778.298	26.672	26.672	0.0001	0.0001	248.938	84.4611	795.711	22.2267	146.695	106.688	13.336	111.133	111.133
NV36 BDEV	BDEVs	WT	Female	6m	41.7538	83.5074	58.4552	167.015	8.35083	58.4552	37.9875	371.198	1979.12	1277.66	83.5074	843.424	167.015	108.56	609.603	25.0523	158.664	133.612	50.1045	175.365	150.313
NV37 BDEV	BDEVs	WT	Female	6m	19.139	86.125	33.4931	57.4167	19.139	71.7709	59.5231	581.63	110.049	38.2778	0.0001	14.3543	167.465	52.632	703.354	110.049	90.9097	62.2014	14.3543	215.312	229.667
NV38 BDEV	BDEVs	WT	Male	6m	16.3646	73.6402	32.729	65.458	24.5468	32.729	190.976	234.5	2078.29	924.593	24.5468	458.205	212.738	32.729	409.112	16.3646	73.6402	122.734	40.9113	310.925	212.738
NV39 BDEV	BDEVs	WT	Male	6m	44.3425	126.693	31.6732	158.366	3.16741	126.693	136.24	642.918	1593.16	623.961	9.50204	224.879	326.233	34.8405	437.089	50.6771	63.3464	183.704	15.8367	123.525	196.374
NV40 BDEV	BDEVs	WT	Male	6m	45.6304	134.207	21.4732	112.734	34.8938	123.47	73.014	713.439	77.84	26.8414	2.68423	13.4208	367.726	42.9462	518.038	80.5241	72.4717	96.6289	29.5256	136.891	139.575

Sample nai	Sample typ	Mutation	Sex	Timepoint	mmu-miR-	mmu-miR-	mmu-miR-	mmu-miR-	mmu-miR-	mmu-miR-	mmu-miR-	mmu-miR-	mmu-miR-	mmu-miR-	mmu-miR-	mmu-miR-	mmu-miR-	mmu-miR-	mmu-miR-	mmu-miR-	mmu-miR-	mmu-miR-	mmu-miR-	mmu-miR-	mmu-miR-
NV1 BDEV1	BDEVs	Q331K	Female	3m	29.0133	3953.05	5356.5	195.839	210.346	4279.45	1994.66	97.9198	4841.58	68.9065	971.943	348.159	7126.38	5186.11	1637.38	830.504	7996.77	710.824	681.811	4290.33	10.8801
NV2 BDEV2	BDEVs	Q331K	Female	3m	34.0906	5071.91	6615.88	132.574	151.513	4903.98	1854.78	117.423	5022.67	101.009	1079.53	188.129	6266.34	4292.88	1395.4	779.031	6510.02	527.771	584.589	4118.64	13.8888
NV4 BDEV4	BDEVs	Q331K	Male	3m	24.9881	3848.16	3806.51	174.916	108.282	3215.13	1849.11	108.282	2890.28	24.9881	716.324	491.431	4889.32	3573.29	1527.64	791.288	8321.01	608.042	691.336	3140.16	0.0001
NV5 BDEV5	BDEVs	Q331K	Male	3m	4.88382	2983.95	4219.53	73.2559	102.558	3731.16	1426.05	107.442	3179.3	39.0699	698.372	395.581	5123.02	3789.77	1247.55	761.86	7750.46	566.511	546.977	3345.35	9.76754
NV6 BDEV6	BDEVs	Q331K	Male	3m	35.1331	2002.58	3457.08	49.1863	56.2128	2620.92	1138.31	77.2926	2438.23	21.0799	660.5	323.223	6225.56	4419.73	1004.8	815.085	6274.75	379.436	337.277	3105.75	0.0001
NV7 BDEV7	BDEVs	WT	Female	3m	11.1267	3454.82	5301.84	111.266	111.266	2998.63	1268.44	61.1965	3694.04	66.7599	1168.3	283.729	5413.1	4089.04	1065.4	823.37	6091.83	411.685	617.528	3760.8	5.56341
NV8 BDEV8	BDEVs	WT	Female	3m	36.6657	4832.52	5389.51	102.664	51.3319	1767.28	2045.94	102.664	6218.48	43.9988	1165.97	234.66	5257.84	3959.88	1151.3	608.649	5983.82	432.654	366.656	2705.92	0.0001
NV17 BDEV	BDEVs	WT	Female	3m	62.8975	3585.15	4817.94	113.215	163.533	2603.95	1660.49	62.8975	4767.63	50.318	1509.54	264.169	5912.36	4868.26	1314.91	528.339	5522.39	402.544	540.918	3799.01	12.5796
NV18 BDEV	BDEVs	WT	Male	3m	33.4962	3190.5	5836.49	150.732	92.1143	2738.3	1658.06	58.6182	5007.66	8.37412	1515.7	226.099	8700.6	5392.87	979.76	628.051	7201.65	569.433	711.792	2930.91	8.37412
NV19 BDEV	BDEVs	WT	Male	3m	19.6902	4725.62	4111.21	114.203	126.017	3981.33	1606.71	110.265	3638.73	74.8224	1047.51	326.855	5627.42	3918.33	1435.48	547.384	7159.31	389.864	748.223	3552.09	11.8141
NV20 BDEV	BDEVs	WT	Male	3m	6.09759	4030.44	4795.67	121.95	88.4137	3085.33	1414.62	76.2187	3871.9	106.706	1262.18	213.412	5112.74	3798.73	1376.93	908.526	8768.19	484.75	487.799	3874.95	12.1951
NV21 BDEV	BDEVs	Q331K	Female	6m	43.7941	4138.53	5884.46	114.959	136.856	4587.42	1976.2	104.011	3963.36	32.8456	1007.26	426.991	8381.07	5649.42	1448.02	1045.58	8337.28	624.064	700.704	4702.38	10.9486
NV22 BDEV	BDEVs	Q331K	Male	6m	17.9584	3582.68	4310	89.7917	71.8334	3663.5	1535.44	80.8125	3439.02	35.9167	772.208	368.146	6007.06	4175.31	1688.08	1068.52	9589.74	709.354	619.562	3923.89	0.0001
NV23 BDEV	BDEVs	Q331K	Male	6m	29.2998	2847.93	5918.37	158.219	181.658	5098.15	1857.6	123.059	4564.9	105.479	943.452	316.437	9346.62	6135.36	1640.79	914.152	9457.95	878.992	920.012	4764.14	11.72
NV24 BDEV	BDEVs	Q331K	Male	6m	34.2424	4205.58	4270.88	68.4847	77.8235	7825.92	2337.82	96.5011	4632.05	99.6141	840.493	386.004	6026.65	4087.29	1483.31	631.926	11791.8	803.138	728.427	4762.79	24.9036
NV33 BDEV	BDEVs	Q331K	Male	6m	14.235	3632.75	3506.49	54.0928	45.5518	6522.44	2035.59	71.1747	5938.81	39.8579	973.668	338.791	5671.19	4637.74	1706.81	697.511	13676.9	1087.55	794.308	5246.99	8.54105
NV34 BDEV	BDEVs	Q331K	Male	6m	19.7693	3182.84	3249.19	71.6635	66.7212	7126.8	2920.9	155.683	8594.66	84.0192	1435.74	380.557	5930.76	3916.77	1426.14	533.769	11374.7	850.076	694.394	4517.26	24.7116
NV35 BDEV	BDEVs	WT	Female	6m	13.336	5045.43	4960.9	62.2345	128.914	5432.17	1462.51	57.7892	4187.49	40.0079	1075.77	146.695	5930.05	5361.05	1812.64	911.289	7828.2	577.891	786.821	4471.99	13.336
NV36 BDEV	BDEVs	WT	Female	6m	0.0001	2563.67	3097.57	41.7538	8.35083	2271.4	1002.09	41.7538	4008.35	16.7016	1286.01	267.223	6505.22	5503.13	1398.33	559.499	7640.92	634.656	509.395	4033.4	25.0523
NV37 BDEV	BDEVs	WT	Female	6m	9.56953	3693.8	4205.68	57.4167	110.049	4186.63	1712.93	76.5556	5009.6	86.125	1119.62	205.743	5191.42	4291.89	1291.87	712.923	10162.7	770.34	665.076	4244.04	19.139
NV38 BDEV	BDEVs	WT	Male	6m	8.18234	3960.2	3551.09	73.6402	114.551	3690.19	1407.34	130.916	5162.99	65.458	1440.07	319.107	6136.68	4835.7	1818.39	630.032	10350.5	810.041	638.214	3567.45	16.3646
NV39 BDEV	BDEVs	WT	Male	6m	38.0079	4532.43	4665.35	91.8522	76.0156	5352.76	1317.6	69.681	5387.6	98.1868	1441.13	323.066	6473.99	4852.32	1626.24	839.338	10182.9	814	874.179	4868.16	3.16741
NV40 BDEV	BDEVs	WT	Male	6m	40.2621	3908.1	3494.53	64.4193	56.3669	5615.21	1385.01	83.2082	5397.79	96.6289	1613.16	225.467	6941.17	4536.19	1359.63	477.776	8135.61	571.721	893.817	4920.02	8.0525

Sample na	Sample typ	Mutation	Sex	Timepoint	mmu-miR-	mmu-miR-	mmu-miR-	mmu-miR-	mmu-miR-	mmu-miR-	mmu-miR-	mmu-miR-	mmu-miR-	mmu-miR-	mmu-miR-	mmu-miR-	mmu-miR-	mmu-miR-	mmu-miR-	mmu-miR-	mmu-miR-	mmu-miR-	mmu-miR-	mmu-miR-	mmu-miR-
NV1 BDEV1	BDEVs	Q331K	Female	3m	76.1598	29.0133	250.239	68.9065	402.559	16214.8	123.306	32.64	2132.47	4156.15	326.399	2132.47	25.3867	525.865	21.76	10.8801	36.2666	50.7733	228.479	26.0547	7.25341
NV2 BDEV2	BDEVs	Q331K	Female	3m	20.2019	3.78793	131.312	83.3324	334.592	14064.2	160.352	31.5654	1699.47	4055.51	233.583	2398.96	13.8888	473.479	30.3028	11.3636	25.2523	22.7271	273.987	46.3472	5.05054
NV4 BDEV4	BDEVs	Q331K	Male	3m	24.9881	24.9881	241.551	58.3055	524.749	12394.1	116.611	0.0001	1690.86	3406.7	283.198	2398.85	8.32944	366.491	24.9881	16.6588	16.6588	24.9881	274.868	25.425	8.32944
NV5 BDEV5	BDEVs	Q331K	Male	3m	39.0699	43.9536	302.791	43.9536	395.581	11442.6	83.0233	14.6513	1465.12	2832.56	278.372	2114.65	14.6513	415.116	19.535	9.76754	24.4187	4.88382	195.349	24.8963	4.88382
NV6 BDEV6	BDEVs	Q331K	Male	3m	77.2926	28.1065	252.957	21.0799	281.064	7630.88	77.2926	14.0533	1159.39	1637.2	175.665	1510.72	0.0001	372.41	14.0533	14.0533	42.1597	0.0001	91.3458	7.29113	7.02669
NV7 BDEV7	BDEVs	WT	Female	3m	22.2534	33.38	378.305	77.8865	205.843	10931.9	66.7599	27.8167	1101.54	2642.57	194.716	2331.03	11.1267	561.895	16.69	22.2534	38.9433	11.1267	211.406	50.7185	22.2534
NV8 BDEV8	BDEVs	WT	Female	3m	29.3326	73.3312	293.325	80.6644	410.654	9621.05	168.662	58.665	1385.96	2273.27	329.99	1173.3	36.6657	322.657	0.0001	29.3326	7.33321	29.3326	249.326	0.0001	7.33321
NV17 BDEV	BDEVs	WT	Female	3m	100.636	12.5796	364.805	50.318	528.339	11258.6	62.8975	12.5796	1647.91	3144.87	301.908	3232.93	0.0001	616.395	0.0001	12.5796	62.8975	0.0001	239.01	51.1497	50.318
NV18 BDEV	BDEVs	WT	Male	3m	50.2442	25.1222	334.961	108.862	418.701	11690.1	66.9922	33.4962	1616.19	2612.69	259.595	2361.47	16.7481	527.563	25.1222	25.1222	16.7481	8.37412	184.228	8.73821	16.7481
NV19 BDEV	BDEVs	WT	Male	3m	19.6902	19.6902	192.963	114.203	346.545	12841.9	118.141	82.6984	2059.58	3654.48	315.041	2213.16	15.7522	653.711	27.5662	27.5662	47.2563	23.6282	173.273	7.99325	15.7522
NV20 BDEV	BDEVs	WT	Male	3m	18.2926	12.1951	146.34	67.0725	356.703	11472.4	79.2674	36.585	1899.37	2774.36	234.753	1878.03	18.2926	402.434	18.2926	6.09759	12.1951	39.6338	222.558	21.6878	15.2438
NV21 BDEV	BDEVs	Q331K	Female	6m	43.7941	60.2168	186.125	93.0623	596.693	13351.7	109.485	21.8971	1762.71	3700.59	459.837	2748.07	0.0001	541.951	16.4228	16.4228	16.4228	32.8456	186.125	38.9597	0.0001
NV22 BDEV	BDEVs	Q331K	Male	6m	62.8542	71.8334	179.583	116.729	242.437	14205	107.75	53.8751	2460.29	4310	377.125	2442.33	17.9584	502.833	8.97926	26.9376	26.9376	44.8959	224.479	0.0001	17.9584
NV23 BDEV	BDEVs	Q331K	Male	6m	17.5799	29.2998	210.958	58.5996	310.577	16566.1	105.479	17.5799	2320.54	4441.84	386.757	2009.96	35.1598	486.376	23.4399	5.86005	11.72	46.8797	222.678	35.3305	5.86005
NV24 BDEV	BDEVs	Q331K	Male	6m	24.9036	37.3553	264.6	93.3882	280.164	29252.3	214.793	52.92	3442.91	8202.59	382.891	4946.46	40.4683	644.378	31.1295	15.5648	31.1295	15.5648	221.019	38.0151	15.5648
NV33 BDEV	BDEVs	Q331K	Male	6m	17.082	22.776	219.218	76.8686	279.004	37810.8	162.278	37.0109	5622.79	9360.88	387.19	3615.67	22.776	572.244	28.4699	5.69407	34.1639	22.776	210.677	26.0409	11.388
NV34 BDEV	BDEVs	Q331K	Male	6m	54.3654	29.6539	244.644	66.7212	301.481	26564.9	247.115	22.2405	3706.73	6489.24	338.548	4040.33	29.6539	538.711	27.1828	29.6539	69.1923	19.7693	118.615	30.1092	9.8847
NV35 BDEV	BDEVs	WT	Female	6m	8.89073	17.7814	164.477	93.3517	408.969	12664.7	120.024	35.5626	2169.31	2991.7	342.289	2809.44	13.336	528.992	13.336	4.44541	8.89073	31.1173	262.274	26.978	13.336
NV36 BDEV	BDEVs	WT	Female	6m	58.4552	41.7538	250.522	50.1045	325.679	6822.55	83.5074	33.403	1494.78	1311.06	217.119	1344.47	16.7016	267.223	8.35083	33.403	0.0001	33.403	175.365	8.62373	8.35083
NV37 BDEV	BDEVs	WT	Female	6m	28.7084	33.4931	215.312	52.632	301.437	11904.4	148.326	33.4931	1851.69	2784.71	287.083	2478.48	9.56953	459.333	9.56953	4.78482	43.0626	14.3543	210.528	29.5486	9.56953
NV38 BDEV	BDEVs	WT	Male	6m	90.0047	8.18234	310.925	24.5468	392.747	10015.1	204.556	16.3646	1963.74	2741.05	278.196	1832.82	8.18234	630.032	8.18234	32.729	98.1869	16.3646	270.014	90.9937	16.3646
NV39 BDEV	BDEVs	WT	Male	6m	28.5059	6.33473	107.689	72.8483	275.556	14531.6	129.86	31.6732	1856.05	3461.87	190.039	2540.19	12.6694	427.587	15.8367	19.004	44.3425	38.0079	190.039	25.7862	15.8367
NV40 BDEV	BDEVs	WT	Male	6m	37.578	10.7366	228.151	45.6304	314.044	10607.7	136.891	26.8414	1360.86	2523.09	271.098	4541.55	5.36837	418.725	16.1049	16.1049	29.5256	34.8938	158.364	13.6451	13.4208

Sample na	Sample typ	Mutation	Sex	Timepoint	mmu-miR-	mmu-miR-	mmu-miR-	mmu-miR-	mmu-miR-	mmu-miR-	mmu-miR-	mmu-miR-	mmu-miR-	mmu-miR-	mmu-miR-	mmu-miR-	mmu-miR-	mmu-miR-	mmu-miR-	mmu-miR-	mmu-miR-	mmu-miR-	mmu-miR-	mmu-miR-	mmu-miR-
NV1 BDEV1	BDEVs	Q331K	Female	3m	333.652	504.105	11344.2	47.1466	1233.06	36.2666	232.106	1864.1	21.76	9527.22	412.629	6495.34	583.891	14.5067	97.9198	315.519	7.25341	344.532	39.8933	14.5067	464.212
NV2 BDEV2	BDEVs	Q331K	Female	3m	185.604	753.779	14165.2	39.141	1707.05	47.9793	228.533	1885.08	20.2019	6849.66	473.19	8532.72	719.688	11.3636	58.0802	142.675	6.31315	386.359	30.3028	7.57576	343.43
NV4 BDEV4	BDEVs	Q331K	Male	3m	233.222	807.946	11078	8.32944	1207.75	24.9881	99.9522	1632.55	8.32944	8962.37	366.491	8146.1	516.419	24.9881	133.27	358.162	24.9881	399.809	16.6588	0.0001	366.491
NV5 BDEV5	BDEVs	Q331K	Male	3m	410.232	659.302	13757.4	43.9536	1147.67	73.2559	219.767	1640.93	24.4187	11066.5	654.418	8009.3	561.628	14.6513	53.721	239.302	0.0001	288.139	43.9536	4.88382	532.325
NV6 BDEV6	BDEVs	Q331K	Male	3m	351.33	562.128	10441.5	7.02669	765.899	49.1863	210.798	1292.89	28.1065	8347.59	484.835	7926	400.516	7.02669	63.2394	330.25	14.0533	295.117	42.1597	0.0001	435.649
NV7 BDEV7	BDEVs	WT	Female	3m	406.122	751.047	11338	27.8167	990.27	27.8167	261.476	1385.27	11.1267	8817.85	534.078	6397.81	650.908	11.1267	27.8167	222.533	11.1267	205.843	55.6332	22.2534	595.275
NV8 BDEV8	BDEVs	WT	Female	3m	483.986	718.645	8367.08	43.9988	945.972	51.3319	241.993	1151.3	21.9994	7523.78	445.181	4091.88	733.312	14.6663	7.33321	381.322	7.33321	329.99	29.3326	0.0001	476.653
NV17 BDEV	BDEVs	WT	Female	3m	603.815	817.667	18303.2	25.1591	1270.53	50.318	150.954	2088.19	12.5796	12466.3	452.862	9635.89	540.918	0.0001	88.0565	389.964	25.1591	226.431	25.1591	0.0001	515.759
NV18 BDEV	BDEVs	WT	Male	3m	619.677	619.677	12234.4	25.1222	1080.25	16.7481	234.473	1917.65	25.1222	9203.05	468.945	7561.74	787.158	8.37412	92.1143	376.831	16.7481	460.571	25.1222	25.1222	611.303
NV19 BDEV	BDEVs	WT	Male	3m	334.731	590.702	12507.1	23.6282	2161.97	43.3183	228.405	2677.85	3.93812	10380.6	504.066	9600.88	665.525	15.7522	78.7604	137.831	15.7522	429.244	35.4422	19.6902	630.083
NV20 BDEV	BDEVs	WT	Male	3m	329.264	618.895	11100.5	9.14633	1146.33	30.4875	204.266	1634.13	6.09759	8435.87	536.579	7143.21	539.628	15.2438	57.9262	189.022	9.14633	280.485	45.7313	15.2438	448.165
NV21 BDEV	BDEVs	Q331K	Female	6m	290.135	684.281	16548.7	27.3713	1280.97	27.3713	268.238	2403.19	38.3198	12919.2	574.796	11151	673.333	32.8456	49.2683	312.032	0.0001	295.609	38.3198	21.8971	377.723
NV22 BDEV	BDEVs	Q331K	Male	6m	466.916	799.145	11592.1	26.9376	1275.04	53.8751	161.625	1831.75	8.97926	9365.26	448.958	7829.83	583.645	26.9376	80.8125	314.271	26.9376	395.083	26.9376	26.9376	386.104
NV23 BDEV	BDEVs	Q331K	Male	6m	275.418	755.933	13436.9	23.4399	1992.38	46.8797	199.238	2291.24	11.72	9293.88	644.594	8713.74	632.874	29.2998	87.8993	281.278	5.86005	269.558	17.5799	11.72	474.656
NV24 BDEV	BDEVs	Q331K	Male	6m	221.019	1027.27	21843.5	15.5648	2104.35	52.92	189.889	2642.88	31.1295	16616.9	1086.42	14730.4	694.185	21.7907	77.8235	295.729	12.4518	566.555	52.92	49.8071	417.134
NV33 BDEV	BDEVs	Q331K	Male	6m	148.043	1400.72	17648.5	22.776	1742.35	34.1639	159.431	2451.25	28.4699	14545.2	1303.92	12708.9	572.244	5.69407	96.7975	296.086	5.69407	387.19	116.726	25.6229	407.119
NV34 BDEV	BDEVs	Q331K	Male	6m	306.423	1220.75	17038.6	17.2982	1364.08	24.7116	185.336	2508.22	37.0674	15138.3	923.644	12289	548.596	9.8847	101.317	491.759	14.827	410.211	74.1346	17.2982	370.673
NV35 BDEV	BDEVs	WT	Female	6m	235.602	840.164	18910.4	13.336	1475.84	31.1173	182.258	2035.95	13.336	12580.2	680.133	11371.1	617.899	22.2267	31.1173	177.813	0.0001	373.406	71.1251	17.7814	488.985
NV36 BDEV	BDEVs	WT	Female	6m	425.887	643.006	7824.64	50.1045	718.163	58.4552	250.522	1119	16.7016	7966.6	300.626	5361.17	375.783	0.0001	58.4552	467.641	0.0001	242.171	8.35083	0.0001	375.783
NV37 BDEV	BDEVs	WT	Female	6m	468.902	794.263	9870.87	0.0001	1086.13	23.9237	277.514	1492.83	23.9237	8033.54	463.041	5961.76	612.444	14.3543	57.4167	449.764	14.3543	397.132	86.125	19.139	502.395
NV38 BDEV	BDEVs	WT	Male	6m	458.205	556.392	10424.2	16.3646	965.504	40.9113	171.827	1849.19	0.0001	9524.12	343.654	7380.38	572.757	24.5468	57.2757	368.201	8.18234	245.467	81.8225	8.18234	417.294
NV39 BDEV	BDEVs	WT	Male	6m	205.875	655.634	15880.9	6.33473	1558.32	38.0079	161.533	1811.7	6.33473	9080.69	471.93	10319.1	630.295	19.004	76.0156	186.872	6.33473	392.747	63.3464	19.004	449.759
NV40 BDEV	BDEVs	WT	Male	6m	222.783	928.71	23153.3	21.4732	1948.68	37.578	182.521	2399.62	21.4732	14021.9	673.054	13334.8	552.932	34.8938	91.2606	236.204	2.68423	295.255	32.2097	16.1049	394.568

Sample nai	Sample typ	Mutation	Sex	Timepoint	mmu-miR-	mmu-miR-	mmu-miR-	mmu-miR-	mmu-miR-	mmu-miR-	mmu-miR-	mmu-miR-	mmu-miR-	mmu-miR-	mmu-miR-	mmu-miR-	mmu-miR-	mmu-miR-	mmu-miR-	mmu-miR-	mmu-miR-	mmu-miR-	mmu-miR-	mmu-miR-	mmu-miR-
NV1 BDEV1	BDEVs	Q331K	Female	3m	895.784	863.144	32.64	456.958	105.173	14.5067	645.544	39.8933	76.1598	848.637	18.1334	181.333	4203.29	195.839	1762.55	174.079	1599.35	652.798	32.64	25.3867	10.8801
NV2 BDEV2	BDEVs	Q331K	Female	3m	493.681	122.473	41.6663	712.113	89.6455	22.7271	758.829	29.0401	143.938	712.113	15.1514	188.129	3565.61	185.604	2212.09	114.898	832.061	679.285	27.7775	6.31315	8.83837
NV4 BDEV4	BDEVs	Q331K	Male	3m	1057.83	1549.26	33.3175	366.491	99.9522	8.32944	674.677	108.282	116.611	674.677	8.32944	99.9522	3523.31	233.222	2248.92	216.563	1474.29	716.324	41.6468	33.3175	8.32944
NV5 BDEV5	BDEVs	Q331K	Male	3m	678.837	1152.56	43.9536	283.256	87.907	0.0001	727.674	53.721	200.233	713.023	14.6513	136.744	2559.07	200.233	1977.91	87.907	1601.86	488.372	68.3722	4.88382	19.535
NV6 BDEV6	BDEVs	Q331K	Male	3m	625.367	779.952	21.0799	295.117	98.3724	7.02669	386.463	63.2394	70.266	372.41	0.0001	56.2128	2459.19	98.3724	1862.05	56.2128	857.244	274.037	21.0799	0.0001	35.1331
NV7 BDEV7	BDEVs	WT	Female	3m	801.117	767.737	33.38	439.502	116.83	5.56341	1068.16	55.6332	94.5764	545.205	11.1267	116.83	2470.11	133.52	1574.42	255.912	1702.37	294.856	5.56341	16.69	11.1267
NV8 BDEV8	BDEVs	WT	Female	3m	1085.3	1495.96	65.9981	586.649	109.997	7.33321	1107.3	29.3326	87.9975	623.315	0.0001	124.663	3820.55	322.657	1591.29	447.32	1488.62	476.653	29.3326	7.33321	7.33321
NV17 BDEV	BDEVs	WT	Female	3m	2125.93	956.041	62.8975	566.077	201.272	12.5796	1220.21	75.477	125.795	415.123	25.1591	138.374	3346.14	176.113	1484.38	314.487	1320.85	553.497	12.5796	0.0001	0.0001
NV18 BDEV	BDEVs	WT	Male	3m	1030	460.571	66.9922	510.815	108.862	8.37412	954.638	133.984	83.7403	695.043	25.1222	184.228	2729.93	133.984	2101.88	234.473	1297.97	544.311	41.8702	41.8702	16.7481
NV19 BDEV	BDEVs	WT	Male	3m	1063.26	571.012	47.2563	444.996	114.203	19.6902	846.673	55.1323	90.5745	760.037	15.7522	102.389	3705.67	181.149	2725.11	86.6364	787.603	582.826	7.87613	27.5662	15.7522
NV20 BDEV	BDEVs	WT	Male	3m	743.894	762.186	21.3413	381.093	100.609	3.04884	911.574	60.975	155.486	612.798	36.585	164.632	2429.85	137.194	2195.1	240.851	1332.3	384.142	15.2438	30.4875	3.04884
NV21 BDEV	BDEVs	Q331K	Female	6m	1149.59	947.045	60.2168	481.734	136.856	32.8456	810.189	120.434	109.485	739.024	16.4228	120.434	3361.19	218.97	2063.79	240.867	1642.27	481.734	38.3198	16.4228	5.47435
NV22 BDEV	BDEVs	Q331K	Male	6m	754.25	1984.39	0.0001	368.146	152.646	8.97926	727.312	89.7917	107.75	879.958	26.9376	152.646	2873.33	206.521	1885.62	224.479	1670.12	439.979	26.9376	17.9584	8.97926
NV23 BDEV	BDEVs	Q331K	Male	6m	761.793	416.056	58.5996	375.037	87.8993	23.4399	498.096	123.059	52.7396	961.031	5.86005	152.359	2730.74	205.098	1711.1	146.499	1441.55	339.877	29.2998	11.72	5.86005
NV24 BDEV	BDEVs	Q331K	Male	6m	522.974	470.054	90.2753	432.698	71.5977	24.9036	719.089	49.8071	90.2753	1752.58	34.2424	118.292	5179.93	196.115	2870.13	140.082	2197.73	522.974	43.5812	18.6777	15.5648
NV33 BDEV	BDEVs	Q331K	Male	6m	404.272	515.304	51.2458	276.157	88.2566	25.6229	577.938	93.9506	85.4096	2118.16	22.776	224.912	5449.13	358.72	2482.57	122.42	2642	307.474	28.4699	5.69407	17.082
NV34 BDEV	BDEVs	Q331K	Male	6m	578.249	548.596	71.6635	333.605	103.788	24.7116	892.086	66.7212	168.038	1761.93	29.6539	155.683	4289.89	365.73	2335.24	172.981	2757.8	387.971	19.7693	9.8847	19.7693
NV35 BDEV	BDEVs	WT	Female	6m	666.797	484.539	26.672	373.406	84.4611	4.44541	675.688	57.7892	102.242	906.844	8.89073	213.375	2711.64	151.141	2813.88	262.274	1426.95	342.289	48.8986	17.7814	26.672
NV36 BDEV	BDEVs	WT	Female	6m	693.111	1620.04	16.7016	258.873	100.209	8.35083	684.76	50.1045	83.5074	308.977	0.0001	133.612	1603.34	192.067	1427.98	141.963	1436.33	384.134	0.0001	16.7016	33.403
NV37 BDEV	BDEVs	WT	Female	6m	942.589	1488.05	57.4167	349.284	43.0626	19.139	1081.35	76.5556	177.035	837.326	19.139	186.604	2578.96	210.528	1583.74	263.16	2019.15	545.458	57.4167	9.56953	9.56953
NV38 BDEV	BDEVs	WT	Male	6m	842.77	1832.82	8.18234	335.472	139.098	0.0001	760.948	81.8225	155.463	630.032	32.729	180.009	2593.77	278.196	2274.66	253.649	2070.11	474.57	73.6402	24.5468	57.2757
NV39 BDEV	BDEVs	WT	Male	6m	516.272	202.708	53.8444	585.953	139.362	12.6694	722.148	44.3425	152.031	839.338	6.33473	177.37	2774.57	209.043	2635.2	126.693	1206.75	551.113	38.0079	6.33473	12.6694
NV40 BDEV	BDEVs	WT	Male	6m	638.824	416.041	37.578	509.985	48.3145	10.7366	977.025	91.2606	112.734	673.718	2.68423	91.2606	2222.41	198.626	2496.24	201.31	1446.75	429.461	32.2097	10.7366	5.36837

Sample na	Sample typ	Mutation	Sex	Timepoint	mmu-miR-	mmu-miR-	mmu-miR-	mmu-miR-	mmu-miR-	mmu-miR-	mmu-miR-	mmu-miR-	mmu-miR-	mmu-miR-	mmu-miR-	mmu-miR-	mmu-miR-	mmu-miR-	mmu-miR-	mmu-miR-	mmu-miR-	mmu-miR-	mmu-miR-	mmu-miR-	mmu-miR-
NV1 BDEV1	BDEVs	Q331K	Female	3m	29.0133	50.7733	649.171	87.0398	94.2931	58.0266	598.398	188.586	39.8933	235.733	192.213	1751.67	54.3999	25.3867	322.772	3561.37	319.146	43.5199	14.5067	427.945	50.7733
NV2 BDEV2	BDEVs	Q331K	Female	3m	15.1514	39.141	946.958	27.7775	46.7167	30.3028	710.85	128.786	13.8888	174.24	111.11	1176.75	47.9793	12.6262	405.298	4132.52	126.261	49.2419	25.2523	396.46	92.1707
NV4 BDEV4	BDEVs	Q331K	Male	3m	24.9881	74.9642	691.336	116.611	91.6229	58.3055	724.653	183.246	83.2935	333.174	108.282	1465.96	33.3175	8.32944	366.491	3514.98	441.455	66.6348	8.32944	283.198	74.9642
NV5 BDEV5	BDEVs	Q331K	Male	3m	4.88382	68.3722	483.488	34.1861	29.3024	29.3024	463.953	78.1396	48.8373	244.186	166.047	1528.6	14.6513	9.76754	297.907	4820.23	361.395	92.7908	24.4187	205.116	83.0233
NV6 BDEV6	BDEVs	Q331K	Male	3m	28.1065	63.2394	337.277	49.1863	35.1331	35.1331	372.41	63.2394	70.266	168.638	133.505	1166.41	28.1065	21.0799	154.585	3646.8	534.021	77.2926	21.0799	210.798	35.1331
NV7 BDEV7	BDEVs	WT	Female	3m	22.2534	33.38	539.641	89.0131	38.9433	116.83	806.681	66.7599	50.0699	155.773	100.14	1424.21	27.8167	16.69	311.546	3666.22	667.598	50.0699	27.8167	189.153	94.5764
NV8 BDEV8	BDEVs	WT	Female	3m	14.6663	21.9994	733.312	73.3312	109.997	95.3306	696.646	131.996	43.9988	315.324	146.662	953.305	21.9994	0.0001	256.659	2434.59	689.313	58.665	7.33321	461.986	139.329
NV17 BDE	BDEVs	WT	Female	3m	37.7386	37.7386	628.974	0.0001	25.1591	12.5796	301.908	138.374	12.5796	452.862	150.954	1698.23	25.1591	0.0001	176.113	3497.1	981.2	25.1591	25.1591	503.18	88.0565
NV18 BDE	BDEVs	WT	Male	3m	41.8702	25.1222	636.425	41.8702	83.7403	83.7403	1138.87	125.61	92.1143	150.732	192.602	1674.8	25.1222	0.0001	167.48	4496.85	468.945	75.3663	16.7481	410.327	117.236
NV19 BDE	BDEVs	WT	Male	3m	15.7522	59.0703	842.735	59.0703	47.2563	31.5042	614.331	169.335	51.1943	228.405	86.6364	1220.78	23.6282	31.5042	318.979	3788.37	232.343	114.203	3.93812	346.545	106.327
NV20 BDE	BDEVs	WT	Male	3m	30.4875	54.8775	545.725	64.0237	30.4875	33.5363	393.288	115.852	33.5363	189.022	97.5599	1560.96	9.14633	15.2438	353.654	4539.58	402.434	97.5599	21.3413	274.387	57.9262
NV21 BDE	BDEVs	Q331K	Female	6m	16.4228	54.7426	525.528	109.485	136.856	164.228	1647.75	153.279	60.2168	197.073	114.959	1948.83	49.2683	38.3198	344.878	4850.18	531.002	65.6911	38.3198	328.455	76.6396
NV22 BDE	BDEVs	Q331K	Male	6m	0.0001	17.9584	547.729	107.75	80.8125	35.9167	700.375	134.687	44.8959	233.458	116.729	1562.37	0.0001	35.9167	251.417	3753.29	439.979	107.75	0.0001	287.333	35.9167
NV23 BDE	BDEVs	Q331K	Male	6m	11.72	82.0394	509.816	111.339	146.499	99.6192	1746.26	205.098	35.1598	234.398	175.799	1306.77	70.3195	17.5799	363.317	3392.91	328.157	93.7593	134.779	310.577	76.1794
NV24 BDE	BDEVs	Q331K	Male	6m	40.4683	49.8071	756.444	77.8235	62.2588	71.5977	787.573	233.47	15.5648	127.631	261.487	1326.11	90.2753	37.3553	908.978	3682.6	196.115	80.9365	18.6777	563.442	59.1459
NV33 BDE	BDEVs	Q331K	Male	6m	34.1639	19.929	902.494	65.4807	51.2458	62.6337	552.315	113.879	39.8579	122.42	364.414	1545.91	65.4807	37.0109	1153.03	3866.2	259.076	93.9506	45.5518	620.642	79.7156
NV34 BDE	BDEVs	Q331K	Male	6m	24.7116	42.0097	551.067	96.375	49.4231	64.25	504.115	111.202	49.4231	138.385	271.827	1759.46	79.0769	42.0097	1588.95	3286.63	232.288	69.1923	17.2982	743.817	93.9038
NV35 BDE	BDEVs	WT	Female	6m	13.336	35.5626	742.367	53.3439	44.4532	17.7814	591.227	137.805	26.672	177.813	133.36	1813.69	35.5626	8.89073	560.11	4191.93	382.297	106.688	44.4532	351.18	75.5704
NV36 BDE	BDEVs	WT	Female	6m	0.0001	25.0523	300.626	91.8581	25.0523	100.209	375.783	91.8581	41.7538	267.223	83.5074	1402.92	16.7016	8.35083	225.47	3615.87	751.566	75.1567	33.403	242.171	50.1045
NV37 BDE	BDEVs	WT	Female	6m	33.4931	57.4167	598.09	43.0626	52.632	33.4931	540.673	110.049	47.8473	229.667	105.264	1961.73	9.56953	28.7084	440.194	3258.39	492.826	62.2014	57.4167	330.146	86.125
NV38 BDE	BDEVs	WT	Male	6m	16.3646	24.5468	523.663	73.6402	32.729	49.0935	400.93	73.6402	32.729	163.645	49.0935	1890.1	8.18234	0.0001	572.757	4606.6	589.121	73.6402	57.2757	327.289	24.5468
NV39 BDE	BDEVs	WT	Male	6m	12.6694	38.0079	598.622	57.0117	79.1829	38.0079	839.338	133.027	19.004	183.704	82.3502	1849.71	44.3425	6.33473	668.303	3851.45	228.047	69.681	15.8367	310.397	63.3464
NV40 BDE	BDEVs	WT	Male	6m	24.1573	45.6304	544.879	42.9462	26.8414	45.6304	432.146	118.102	24.1573	174.469	91.2606	1484.33	64.4193	24.1573	566.352	3449.11	362.358	53.6828	8.0525	467.039	67.1034

Sample na	Sample typ	Mutation	Sex	Timepoint	mmu-miR-	mmu-miR-	mmu-miR-	mmu-miR-	mmu-miR-	mmu-miR-	mmu-miR-	mmu-miR-	mmu-miR-	mmu-miR-	mmu-miR-	mmu-miR-	mmu-miR-	mmu-miR-	mmu-miR-	mmu-miR-	mmu-miR-	mmu-miR-	mmu-miR-	mmu-miR-	mmu-miR-
NV1 BDEV1	BDEVs	Q331K	Female	3m	297.386	2139.73	152.32	29.0133	108.8	467.838	72.5332	21.76	1461.54	65.2799	174.079	859.517	3604.89	7.25341	9498.21	409.812	2183.25	710.824	1029.97	10.8801	10.8801
NV2 BDEV2	BDEVs	Q331K	Female	3m	348.481	2582.04	188.129	25.2523	98.4837	343.43	23.9897	42.9289	2304.26	88.3829	275.249	867.414	4074.44	6.31315	12238.5	467.166	1950.73	819.434	930.544	13.8888	26.5149
NV4 BDEV4	BDEVs	Q331K	Male	3m	183.246	1882.43	49.9762	16.6588	149.928	258.21	66.6348	24.9881	1590.9	33.3175	116.611	1016.18	2748.68	8.32944	9037.34	424.797	1840.78	441.455	674.677	0.0001	16.6588
NV5 BDEV5	BDEVs	Q331K	Male	3m	258.837	1929.07	78.1396	9.76754	34.1861	210	29.3024	34.1861	1767.91	92.7908	151.395	932.79	3477.21	4.88382	9459.76	410.232	2832.56	752.093	698.372	9.76754	29.3024
NV6 BDEV6	BDEVs	Q331K	Male	3m	224.851	1665.3	84.3192	21.0799	77.2926	175.665	28.1065	28.1065	1819.89	21.0799	112.426	583.207	3239.26	28.1065	10476.6	238.904	1707.46	548.074	674.553	21.0799	7.02669
NV7 BDEV7	BDEVs	WT	Female	3m	211.406	1885.96	55.6332	22.2534	61.1965	411.685	27.8167	27.8167	1758.01	22.2534	144.646	1379.7	2586.94	0.0001	13563.4	305.982	1880.4	823.37	817.807	16.69	11.1267
NV8 BDEV8	BDEVs	WT	Female	3m	271.325	1935.94	58.665	21.9994	87.9975	483.986	58.665	36.6657	1796.61	73.3312	234.66	1136.63	2947.91	36.6657	13067.6	432.654	1811.28	872.641	1026.64	7.33321	29.3326
NV17 BDEV	BDEVs	WT	Female	3m	352.226	2478.16	62.8975	12.5796	88.0565	490.6	37.7386	12.5796	1509.54	50.318	88.0565	2138.51	2427.84	12.5796	14177.1	239.01	1748.55	628.974	1044.1	25.1591	12.5796
NV18 BDEV	BDEVs	WT	Male	3m	510.815	1733.42	41.8702	66.9922	83.7403	343.335	41.8702	25.1222	2135.37	58.6182	200.977	1046.75	3106.76	16.7481	13222.6	351.709	1691.55	527.563	803.906	0.0001	8.37412
NV19 BDEV	BDEVs	WT	Male	3m	519.818	2756.61	149.645	19.6902	181.149	279.599	66.9464	39.3803	1850.87	106.327	165.397	1705.16	2213.16	11.8141	10742.9	551.322	2768.43	791.541	728.533	23.6282	47.2563
NV20 BDEV	BDEVs	WT	Male	3m	344.508	2563.99	128.047	9.14633	76.2187	277.436	24.39	45.7313	2268.27	45.7313	146.34	1048.77	2795.7	6.09759	10396.2	387.19	1874.98	676.821	798.771	6.09759	24.39
NV21 BDEV	BDEVs	Q331K	Female	6m	251.815	2080.21	104.011	10.9486	104.011	426.991	27.3713	16.4228	2080.21	27.3713	191.599	1105.8	3153.17	38.3198	11495.9	481.734	2693.33	629.539	1056.53	16.4228	0.0001
NV22 BDEV	BDEVs	Q331K	Male	6m	197.542	1831.75	62.8542	17.9584	89.7917	350.187	89.7917	17.9584	1993.37	8.97926	143.667	969.749	3681.46	0.0001	9751.37	520.791	2361.52	395.083	610.583	8.97926	8.97926
NV23 BDEV	BDEVs	Q331K	Male	6m	392.617	2226.78	199.238	11.72	99.6192	451.216	41.0197	17.5799	2015.82	70.3195	117.199	1002.05	3017.87	11.72	10653.4	416.056	2332.26	433.636	820.393	11.72	23.4399
NV24 BDEV	BDEVs	Q331K	Male	6m	560.329	1354.13	242.809	9.33891	180.55	407.795	52.92	84.0494	2020.3	96.5011	149.421	2823.43	2608.64	6.22597	7642.26	1101.98	5871	566.555	513.635	21.7907	65.3718
NV33 BDEV	BDEVs	Q331K	Male	6m	515.304	1790.75	259.076	14.235	65.4807	338.791	45.5518	34.1639	2308.9	68.3277	202.136	2385.77	3305.35	8.54105	4347.34	1013.53	5508.91	532.386	492.528	22.776	14.235
NV34 BDEV	BDEVs	Q331K	Male	6m	528.826	1608.72	331.134	17.2982	133.442	437.394	19.7693	64.25	1739.69	66.7212	121.087	2636.72	2397.02	14.827	5515.61	1650.73	5421.71	783.355	593.076	27.1828	24.7116
NV35 BDEV	BDEVs	WT	Female	6m	426.75	2667.19	213.375	17.7814	66.6798	257.828	48.8986	26.672	2013.73	80.0157	155.586	1693.66	3005.03	13.336	8486.1	422.305	2973.91	742.367	617.899	8.89073	26.672
NV36 BDEV	BDEVs	WT	Female	6m	217.119	2162.84	66.8059	16.7016	66.8059	208.768	25.0523	50.1045	1753.65	25.0523	100.209	1394.57	2630.48	25.0523	14471.8	459.29	1686.85	534.447	417.537	0.0001	16.7016
NV37 BDEV	BDEVs	WT	Female	6m	339.715	2191.4	162.68	4.78482	100.479	224.882	19.139	62.2014	1803.84	95.6944	186.604	1842.12	2937.82	4.78482	10134	569.381	2440.21	655.506	755.985	9.56953	28.7084
NV38 BDEV	BDEVs	WT	Male	6m	384.565	2209.2	122.734	8.18234	114.551	237.285	32.729	8.18234	2364.67	57.2757	130.916	1120.97	2986.52	0.0001	10964.2	613.668	2880.15	662.761	777.312	8.18234	32.729
NV39 BDEV	BDEVs	WT	Male	6m	475.097	2397.66	186.872	38.0079	41.1752	291.393	22.1713	47.5098	2049.25	76.0156	177.37	1270.09	2904.43	12.6694	9045.85	513.105	2511.68	639.797	680.972	28.5059	15.8367
NV40 BDEV	BDEVs	WT	Male	6m	480.46	2176.83	177.153	10.7366	85.8924	359.674	10.7366	32.2097	1744.69	50.9986	147.627	1736.63	2394.25	8.0525	12073.2	730.084	4069.15	740.821	724.716	32.2097	21.4732

Sample nai	Sample typ	Mutation	Sex	Timepoint	mmu-miR-	mmu-miR-	mmu-miR-	mmu-miR-	mmu-miR-	mmu-miR-	mmu-miR-	mmu-miR-	mmu-miR-	mmu-miR-	mmu-miR-	mmu-miR-	mmu-miR-	mmu-miR-	mmu-miR-	mmu-miR-	mmu-miR-	mmu-miR-	mmu-miR-	mmu-miR-	mmu-miR-
NV1 BDEV1	BDEVs	Q331K	Female	3m	1468.79	32.64	94.2931	68.9065	155.946	65.2799	333.652	591.145	14.5067	337.279	97.9198	8018.53	928.423	152.32	25.3867	39.8933	32.64	32.64	36.2666	264.746	0.235774
NV2 BDEV2	BDEVs	Q331K	Female	3m	1766.39	80.8072	114.898	22.7271	32.828	80.8072	362.369	68.1811	11.3636	385.096	106.059	5070.64	765.142	284.088	13.8888	13.8888	0.0001	16.414	7.57576	194.442	13.5261
NV4 BDEV4	BDEVs	Q331K	Male	3m	1415.99	133.27	66.6348	91.6229	324.844	41.6468	391.479	2507.13	0.0001	291.527	58.3055	7146.58	699.665	216.563	16.6588	16.6588	33.3175	83.2935	49.9762	266.539	83.2935
NV5 BDEV5	BDEVs	Q331K	Male	3m	2070.7	87.907	87.907	34.1861	97.6745	83.0233	317.442	1758.14	24.4187	278.372	78.1396	7135.11	654.418	146.512	14.6513	43.9536	24.4187	34.1861	53.721	332.093	97.6744
NV6 BDEV6	BDEVs	Q331K	Male	3m	1089.12	14.0533	84.3192	77.2926	175.665	42.1597	196.745	9106.47	21.0799	259.984	28.1065	4482.97	463.755	175.665	0.0001	7.02669	28.1065	42.1597	14.0533	224.851	56.2128
NV7 BDEV7	BDEVs	WT	Female	3m	1668.99	44.5066	77.8865	50.0699	139.083	33.38	189.153	61.1965	11.1267	317.109	72.3232	4595.3	556.331	244.786	0.0001	11.1267	11.1267	44.5066	38.9433	255.912	50.0699
NV8 BDEV8	BDEVs	WT	Female	3m	1173.3	58.665	58.665	58.665	285.992	43.9988	278.658	36.6657	29.3326	285.992	36.6657	6724.47	1048.64	425.321	7.33321	7.33321	0.0001	65.9981	29.3326	249.326	29.3318
NV17 BDE\	BDEVs	WT	Female	3m	1824.03	50.318	100.636	188.692	289.328	75.477	301.908	1484.38	12.5796	364.805	50.318	5937.52	754.769	239.01	25.1591	25.1591	25.1591	75.477	12.5796	150.954	97.2312
NV18 BDE\	BDEVs	WT	Male	3m	1850.66	41.8702	41.8702	58.6182	150.732	66.9922	318.213	2302.85	33.4962	376.831	92.1143	6347.51	586.181	401.953	33.4962	25.1222	66.9922	41.8702	25.1222	318.213	41.8463
NV19 BDE\	BDEVs	WT	Male	3m	1744.54	31.5042	59.0703	31.5042	126.017	35.4422	212.653	673.401	11.8141	252.033	86.6364	4418.45	602.516	299.289	27.5662	35.4422	3.93812	86.6364	19.6902	236.281	43.318
NV20 BDE\	BDEVs	WT	Male	3m	1963.39	24.39	79.2674	48.78	137.194	51.8287	207.315	192.071	30.4875	335.362	94.5111	6085.29	658.529	103.657	27.4388	30.4875	15.2438	33.5363	9.14633	249.997	0.339733
NV21 BDE\	BDEVs	Q331K	Female	6m	1932.41	49.2683	104.011	131.382	251.815	109.485	377.723	875.88	32.8456	344.878	109.485	8725.95	826.611	843.034	0.0001	21.8971	16.4228	21.8971	27.3713	208.022	38.3197
NV22 BDE\	BDEVs	Q331K	Male	6m	1822.77	89.7917	152.646	107.75	251.417	71.8334	224.479	5189.95	17.9584	323.25	62.8542	6940.89	781.187	44.8959	8.97926	8.97926	17.9584	35.9167	44.8959	359.166	35.9158
NV23 BDE\	BDEVs	Q331K	Male	6m	1511.87	41.0197	99.6192	52.7396	87.8993	52.7396	375.037	5994.73	11.72	222.678	58.5996	4770	914.152	791.093	5.86005	17.5799	29.2998	23.4399	29.2998	246.118	29.2831
NV24 BDE\	BDEVs	Q331K	Male	6m	1970.49	68.4847	149.421	21.7907	96.5011	80.9365	734.653	504.296	31.1295	373.553	102.727	8130.99	1338.56	133.856	15.5648	34.2424	9.33891	34.2424	21.7907	133.856	46.6942
NV33 BDE\	BDEVs	Q331K	Male	6m	2061.22	48.3988	167.972	37.0109	108.185	59.7867	464.058	1728.12	14.235	361.567	136.655	8754.47	1890.4	85.4096	45.5518	37.0109	2.84708	19.929	11.388	108.185	62.6337
NV34 BDE\	BDEVs	Q331K	Male	6m	1638.37	74.1346	195.221	51.8943	108.731	93.9038	751.23	3983.5	32.1251	385.5	111.202	11026.3	1658.14	98.8461	19.7693	7.41355	4.9424	54.3654	12.3559	143.327	79.0625
NV35 BDE\	BDEVs	WT	Female	6m	2164.87	40.0079	124.469	62.2345	97.797	84.4611	266.719	17.7814	13.336	235.602	80.0157	5756.68	764.594	502.321	8.89073	22.2267	8.89073	4.44541	0.0001	253.383	34.3594
NV36 BDE\	BDEVs	WT	Female	6m	1119	16.7016	33.403	66.8059	225.47	16.7016	292.276	1369.52	0.0001	409.186	116.91	6062.63	467.641	150.313	0.0001	0.0001	0.0001	41.7538	16.7016	258.873	0.46524
NV37 BDE\	BDEVs	WT	Female	6m	1775.13	52.632	172.25	71.7709	186.604	76.5556	287.083	71.7709	14.3543	416.27	124.403	9579	923.451	248.805	19.139	33.4931	9.56953	119.618	23.9237	234.451	0.399867
NV38 BDE\	BDEVs	WT	Male	6m	2053.74	98.1869	147.28	81.8225	196.374	49.0935	270.014	1685.54	24.5468	572.757	65.458	7298.55	670.943	106.369	8.18234	0.0001	16.3646	57.2757	49.0935	220.92	80.2114
NV39 BDE\	BDEVs	WT	Male	6m	1966.9	47.5098	98.1868	28.5059	47.5098	69.681	259.72	766.49	12.6694	487.766	79.1829	5517.46	684.14	247.051	9.50204	28.5059	6.33473	31.6732	19.004	114.023	31.6551
NV40 BDE\	BDEVs	WT	Male	6m	2308.36	40.2621	115.418	45.6304	120.786	50.9986	305.991	67.1034	18.789	370.411	75.1558	7204.21	767.662	378.463	16.1049	42.9462	18.789	13.4208	10.7366	193.258	27.6302

Sample na	Sample typ	Mutation	Sex	Timepoint	mmu-miR-	mmu-miR-	mmu-miR-	mmu-miR-	mmu-miR-	mmu-miR-	mmu-miR-	mmu-miR-	mmu-miR-	mmu-miR-	mmu-miR-	mmu-miR-	mmu-miR-	mmu-miR-	mmu-miR-	mmu-miR-	mmu-miR-	mmu-miR-	mmu-miR-	mmu-miR-	mmu-miR-
NV1 BDEV1	BDEVs	Q331K	Female	3m	50.7733	326.399	460.585	10.8801	7.25341	279.252	10.8801	7.25341	264.746	3.62675	10.8801	36.2666	36.2666	65.2799	3.62675	18.1334	14.5067	239.359	130.56	65.2799	3866.01
NV2 BDEV2	BDEVs	Q331K	Female	3m	28.5984	257.573	510.095	6.31315	42.9289	263.886	27.7775	10.101	248.734	8.83837	13.8888	18.9393	21.4645	41.6663	7.57576	22.7271	6.31315	313.128	108.585	70.7063	5231
NV4 BDEV4	BDEVs	Q331K	Male	3m	116.608	233.222	541.407	0.0001	33.3175	249.88	8.32944	8.32944	199.904	8.32944	0.0001	16.6588	16.6588	91.6229	0.0001	33.3175	8.32944	191.575	66.6348	91.6229	2665.39
NV5 BDEV5	BDEVs	Q331K	Male	3m	34.1861	297.907	395.581	9.76754	19.535	244.186	14.6513	4.88382	136.744	0.0001	14.6513	39.0699	19.535	53.721	0.0001	34.1861	9.76754	273.488	73.2559	19.535	3286.74
NV6 BDEV6	BDEVs	Q331K	Male	3m	21.0799	203.771	337.277	7.02669	7.02669	203.771	21.0799	7.02669	133.505	0.0001	21.0799	7.02669	14.0533	42.1597	21.0799	14.0533	14.0533	224.851	84.3192	14.0533	2318.78
NV7 BDEV7	BDEVs	WT	Female	3m	66.7289	233.659	261.476	11.1267	33.38	272.602	11.1267	11.1267	222.533	5.56341	5.56341	22.2534	27.8167	38.9433	11.1267	38.9433	11.1267	250.349	194.716	50.0699	3721.86
NV8 BDEV8	BDEVs	WT	Female	3m	78.2883	322.657	337.323	36.6657	36.6657	285.992	14.6663	29.3326	234.66	7.33321	7.33321	73.3312	7.33321	21.9994	131.996	29.3326	7.33321	219.994	197.994	43.9988	3857.22
NV17 BDEV	BDEVs	WT	Female	3m	37.7386	276.749	440.282	12.5796	12.5796	503.18	0.0001	0.0001	150.954	12.5796	0.0001	12.5796	37.7386	25.1591	25.1591	25.1591	25.1591	264.169	201.272	75.477	4302.18
NV18 BDEV	BDEVs	WT	Male	3m	2.33235	334.961	368.457	33.4962	33.4962	368.457	8.37412	0.0001	267.969	8.37412	33.4962	25.1222	8.37412	66.9922	33.4962	8.37412	25.1222	209.351	125.61	58.6182	4053.02
NV19 BDEV	BDEVs	WT	Male	3m	35.0729	315.041	385.926	7.87613	3.93812	252.033	39.3803	27.5662	275.661	7.87613	7.87613	11.8141	39.3803	70.8844	15.7522	3.93812	15.7522	189.025	114.203	66.9464	3292.18
NV20 BDEV	BDEVs	WT	Male	3m	27.4384	225.607	454.263	6.09759	27.4388	210.363	6.09759	15.2438	213.412	3.04884	21.3413	15.2438	15.2438	48.78	12.1951	15.2438	3.04884	173.778	91.4624	33.5363	3753
NV21 BDEV	BDEVs	Q331K	Female	6m	76.6396	317.506	481.734	16.4228	38.3198	339.403	5.47435	5.47435	262.764	10.9486	10.9486	27.3713	27.3713	21.8971	16.4228	0.0001	10.9486	208.022	164.228	54.7426	3552.79
NV22 BDEV	BDEVs	Q331K	Male	6m	44.8959	332.229	377.125	26.9376	44.8959	260.396	44.8959	62.8542	233.458	17.9584	8.97926	35.9167	17.9584	53.8751	17.9584	8.97926	8.97926	242.437	62.8542	35.9167	3403.1
NV23 BDEV	BDEVs	Q331K	Male	6m	41.0197	292.997	363.317	11.72	29.2998	193.378	29.2998	17.5799	199.238	5.86005	5.86005	52.7396	35.1598	46.8797	11.72	23.4399	17.5799	275.418	169.939	29.2998	4060.94
NV24 BDEV	BDEVs	Q331K	Male	6m	90.2716	354.875	712.863	12.4518	24.9036	205.454	31.1295	3.11304	280.164	6.22597	37.3553	37.3553	18.6777	49.8071	9.33891	12.4518	31.1295	554.103	112.066	56.033	4040.59
NV33 BDEV	BDEVs	Q331K	Male	6m	58.3697	392.884	492.528	14.235	25.6229	185.054	11.388	11.388	347.332	8.54105	19.929	51.2458	17.082	39.8579	5.69407	5.69407	34.1639	520.998	85.4096	48.3988	2832.75
NV34 BDEV	BDEVs	Q331K	Male	6m	86.485	682.038	805.595	14.827	42.0097	257	14.827	0.0001	242.173	12.3559	54.3654	51.8943	7.41355	24.7116	19.7693	14.827	39.5385	518.942	108.731	46.952	3939.01
NV35 BDEV	BDEVs	WT	Female	6m	2.47623	284.5	284.5	4.44541	26.672	124.469	17.7814	13.336	222.266	8.89073	17.7814	13.336	17.7814	40.0079	8.89073	8.89073	8.89073	253.383	115.578	75.5704	3391.77
NV36 BDEV	BDEVs	WT	Female	6m	5.42688	225.47	300.626	0.0001	41.7538	233.821	8.35083	25.0523	175.365	16.7016	8.35083	58.4552	33.403	16.7016	0.0001	33.403	25.0523	91.8581	208.768	16.7016	4225.47
NV37 BDEV	BDEVs	WT	Female	6m	14.3543	421.055	459.333	9.56953	9.56953	349.284	14.3543	14.3543	210.528	9.56953	19.139	33.4931	9.56953	19.139	4.78482	14.3543	9.56953	330.146	172.25	33.4931	4617.25
NV38 BDEV	BDEVs	WT	Male	6m	49.0707	327.289	433.659	8.18234	16.3646	220.92	8.18234	16.3646	155.463	0.0001	0.0001	24.5468	0.0001	49.0935	0.0001	24.5468	49.0935	188.191	212.738	24.5468	3575.64
NV39 BDEV	BDEVs	WT	Male	6m	25.3385	335.735	468.762	3.16741	25.3386	310.397	19.004	19.004	256.553	6.33473	15.8367	38.0079	12.6694	28.5059	25.3386	28.5059	12.6694	297.728	91.8522	95.0195	3940.14
NV40 BDEV	BDEVs	WT	Male	6m	34.8938	407.988	587.825	8.0525	24.1573	212.047	21.4732	18.789	214.731	13.4208	8.0525	34.8938	26.8414	42.9462	24.1573	37.578	8.0525	327.464	118.102	37.578	3655.79

Sample na	Sample typ	Mutation	Sex	Timepoint	mmu-miR-	mmu-miR-	mmu-miR-	mmu-miR-	mmu-miR-	mmu-miR-	mmu-miR-	mmu-miR-	mmu-miR-	mmu-miR-	mmu-miR-	mmu-miR-	mmu-miR-	mmu-miR-	mmu-miR-	mmu-miR-	mmu-miR-	mmu-miR-	mmu-miR-	mmu-miR-	mmu-miR-
NV1 BDEV1	BDEVs	Q331K	Female	3m	36.2666	14.5067	18.1334	148.693	43.5199	36.2666	72.5332	1283.84	27464.6	72.5332	61.6532	10.8801	18.1334	32.64	36.2666	18.1334	50.7733	21.76	61.6532	105.173	29.0133
NV2 BDEV2	BDEVs	Q331K	Female	3m	25.2523	6.31315	40.4036	196.967	50.5045	13.8888	82.0698	1350.99	31675.1	94.6959	15.1514	12.6262	20.2019	26.5149	83.3324	11.3636	21.4645	15.1514	66.9185	116.16	13.8888
NV4 BDEV4	BDEVs	Q331K	Male	3m	33.3175	8.32944	16.6588	49.9762	41.6468	33.3175	83.2935	1266.06	25687.7	41.6468	83.2935	8.32944	16.6588	16.6588	74.9642	24.9881	66.6348	33.3175	133.27	124.94	49.9762
NV5 BDEV5	BDEVs	Q331K	Male	3m	14.6513	9.76754	34.1861	58.6047	19.535	4.88382	39.0699	1245.35	25522.3	34.1861	180.698	14.6513	24.4187	39.0699	48.8373	4.88382	48.8373	19.535	63.4884	131.861	4.88382
NV6 BDEV6	BDEVs	Q331K	Male	3m	42.1597	0.0001	49.1863	14.0533	7.02669	35.1331	42.1597	808.058	23258	49.1863	196.745	14.0533	7.02669	28.1065	14.0533	7.02669	42.1597	7.02669	133.505	98.3724	7.02669
NV7 BDEV7	BDEVs	WT	Female	3m	0.0001	5.56341	16.69	72.3232	22.2534	0.0001	55.6332	1424.21	30570.4	72.3232	122.393	5.56341	16.69	50.0699	89.0131	27.8167	83.4498	33.38	122.393	116.83	50.0699
NV8 BDEV8	BDEVs	WT	Female	3m	14.6663	7.33321	43.9988	80.6644	65.9981	43.9988	36.6657	791.976	30058.4	73.3312	80.6644	14.6663	0.0001	131.996	65.9981	21.9994	95.3306	29.3326	146.662	65.9981	0.0001
NV17 BDEV	BDEVs	WT	Female	3m	25.1591	0.0001	25.1591	62.8975	50.318	25.1591	50.318	1320.85	24391.6	100.636	226.431	0.0001	75.477	37.7386	100.636	0.0001	138.374	12.5796	150.954	100.636	62.8975
NV18 BDEV	BDEVs	WT	Male	3m	8.37412	25.1222	33.4962	33.4962	16.7481	8.37412	58.6182	1373.34	21663.6	92.1143	33.4962	33.4962	25.1222	58.6182	58.6182	25.1222	75.3663	33.4962	75.3663	83.7403	8.37412
NV19 BDEV	BDEVs	WT	Male	3m	11.8141	3.93812	27.5662	59.0703	98.4505	35.4422	90.5745	886.054	16004.1	70.8844	39.3803	7.87613	47.2563	15.7522	23.6282	19.6902	47.2563	23.6282	78.7604	137.831	35.4422
NV20 BDEV	BDEVs	WT	Male	3m	6.09759	18.2926	30.4875	51.8287	21.3413	30.4875	60.975	1192.06	17877.8	48.78	39.6338	12.1951	48.78	42.6825	51.8287	21.3413	39.6338	21.3413	137.194	128.047	24.39
NV21 BDEV	BDEVs	Q331K	Female	6m	60.2168	5.47435	27.3713	114.959	82.1138	38.3198	49.2683	1527.32	21141.5	65.6911	65.6911	5.47435	5.47435	43.7941	71.1653	5.47435	65.6911	10.9486	87.5881	131.382	38.3198
NV22 BDEV	BDEVs	Q331K	Male	6m	17.9584	35.9167	53.8751	53.8751	53.8751	26.9376	26.9376	1275.04	13396.9	62.8542	134.687	8.97926	35.9167	53.8751	26.9376	44.8959	161.625	0.0001	134.687	53.8751	53.8751
NV23 BDEV	BDEVs	Q331K	Male	6m	23.4399	23.4399	46.8797	128.919	29.2998	41.0197	76.1794	1242.31	16243.8	82.0394	58.5996	5.86005	17.5799	17.5799	52.7396	11.72	29.2998	23.4399	52.7396	105.479	35.1598
NV24 BDEV	BDEVs	Q331K	Male	6m	40.4683	0.0001	37.3553	133.856	56.033	28.0165	49.8071	1824.18	13622.2	121.405	6.22597	3.11304	62.2588	24.9036	71.5977	12.4518	21.7907	15.5648	127.631	90.2753	12.4518
NV33 BDEV	BDEVs	Q331K	Male	6m	5.69407	0.0001	31.3169	113.879	113.879	31.3169	54.0928	1739.51	9782.23	179.36	17.082	2.84708	59.7867	34.1639	71.1747	11.388	45.5518	5.69407	119.573	85.4096	19.929
NV34 BDEV	BDEVs	Q331K	Male	6m	14.827	4.9424	42.0097	175.452	93.9038	34.5962	54.3654	1986.81	8228.93	239.702	17.2982	12.3559	88.9615	54.3654	106.26	46.952	61.7789	17.2982	113.673	74.1346	17.2982
NV35 BDEV	BDEVs	WT	Female	6m	13.336	22.2267	44.4532	66.6798	53.3439	13.336	62.2345	1226.91	16123.2	93.3517	17.7814	4.44541	8.89073	44.4532	111.133	4.44541	26.672	13.336	115.578	102.242	8.89073
NV36 BDEV	BDEVs	WT	Female	6m	0.0001	8.35083	0.0001	33.403	0.0001	41.7538	58.4552	668.059	16709.8	108.56	175.365	0.0001	50.1045	83.5074	66.8059	83.5074	83.5074	16.7016	116.91	125.261	50.1045
NV37 BDEV	BDEVs	WT	Female	6m	4.78482	9.56953	19.139	71.7709	81.3403	52.632	71.7709	1363.64	15047.9	129.187	133.972	23.9237	28.7084	28.7084	66.9861	23.9237	100.479	9.56953	172.25	157.896	33.4931
NV38 BDEV	BDEVs	WT	Male	6m	16.3646	16.3646	32.729	65.458	32.729	0.0001	40.9113	842.77	13091.6	73.6402	98.1869	0.0001	16.3646	40.9113	90.0047	0.0001	81.8225	16.3646	49.0935	147.28	57.2757
NV39 BDEV	BDEVs	WT	Male	6m	28.5059	12.6694	44.3425	133.027	66.5137	22.1713	82.3502	1260.59	20749.1	98.1868	31.6732	12.6694	38.0079	31.6732	38.0079	12.6694	44.3425	15.8367	72.8483	117.191	15.8367
NV40 BDEV	BDEVs	WT	Male	6m	10.7366	10.7366	24.1573	147.627	37.578	21.4732	29.5256	1092.44	19070.8	72.4717	96.6289	10.7366	48.3145	53.6828	72.4717	21.4732	53.6828	16.1049	128.839	77.84	16.1049

Sample na	Sample typ	Mutation	Sex	Timepoint	mmu-miR-	mmu-miR-	mmu-miR-	mmu-miR-	mmu-miR-	mmu-miR-	mmu-miR-	mmu-miR-	mmu-miR-	mmu-miR-	mmu-miR-	mmu-miR-	mmu-miR-	mmu-miR-	mmu-miR-	mmu-miR-	mmu-miR-	mmu-miR-	mmu-miR-	mmu-miR-	mmu-miR-
NV1 BDEV1	BDEVs	Q331K	Female	3m	417.065	32.64	282.879	36.2666	413.439	344.532	141.44	76.1598	126.933	3481.59	7.25341	420.692	76.1598	43.5199	355.412	79.7865	14600.9	9073.89	47.1466	2110.71	65.2799
NV2 BDEV2	BDEVs	Q331K	Female	3m	540.397	21.4645	443.176	7.57576	257.573	357.319	65.6559	46.7167	128.786	4373.68	7.57576	185.604	18.9393	27.7775	214.644	74.4941	17290.2	10379.9	89.6455	3424.2	61.868
NV4 BDEV4	BDEVs	Q331K	Male	3m	374.82	24.9881	266.539	49.9762	324.844	491.431	24.9881	83.2935	149.928	2673.72	33.3175	1499.28	83.2935	8.32944	541.407	108.282	22814.1	14243.2	24.9881	1815.8	58.3055
NV5 BDEV5	BDEVs	Q331K	Male	3m	293.023	48.8373	307.674	9.76754	307.674	527.442	73.2559	39.0699	107.442	2510.23	14.6513	727.674	83.0233	34.1861	634.884	117.209	17928.1	11618.4	39.0699	1948.6	122.093
NV6 BDEV6	BDEVs	Q331K	Male	3m	217.824	7.02669	442.675	35.1331	231.878	576.181	28.1065	21.0799	84.3192	2150.14	14.0533	1257.76	70.266	28.1065	519.968	56.2128	18177.8	13041.4	56.2128	2733.34	49.1863
NV7 BDEV7	BDEVs	WT	Female	3m	261.476	33.38	305.982	55.6332	255.912	472.882	50.0699	50.0699	127.956	2637.01	38.9433	689.851	83.4498	27.8167	734.357	105.703	20612.1	14531.4	38.9433	2030.61	44.5066
NV8 BDEV8	BDEVs	WT	Female	3m	549.984	7.33321	307.991	95.3306	373.989	623.315	87.9975	65.9981	124.663	2742.59	58.665	1437.29	95.3306	36.6657	571.983	65.9981	14922.9	9929.04	58.665	1620.62	95.3306
NV17 BDEV	BDEVs	WT	Female	3m	490.6	37.7386	415.123	75.477	478.021	440.282	37.7386	100.636	176.113	3446.78	12.5796	1660.49	113.215	12.5796	956.041	50.318	18114.5	11359.3	37.7386	2289.47	125.795
NV18 BDEV	BDEVs	WT	Male	3m	410.327	8.37412	267.969	16.7481	66.9922	477.319	117.236	41.8702	100.488	3157	50.2442	1750.17	117.236	8.37412	770.41	100.488	30431.2	19754.3	33.4962	2001.39	41.8702
NV19 BDEV	BDEVs	WT	Male	3m	374.112	35.4422	212.653	11.8141	409.554	232.343	35.4422	55.1323	157.521	3051.96	15.7522	429.244	27.5662	19.6902	370.174	129.955	25140.3	15055	86.6364	1921.75	86.6364
NV20 BDEV	BDEVs	WT	Male	3m	356.703	9.14633	338.411	30.4875	429.873	399.385	51.8287	51.8287	100.609	3341.42	9.14633	603.651	67.0725	33.5363	679.87	85.3649	13246.8	9624.88	48.78	2192.05	70.1212
NV21 BDEV	BDEVs	Q331K	Female	6m	509.105	32.8456	306.558	16.4228	268.238	487.208	82.1138	71.1653	251.815	2988.94	43.7941	1051.06	54.7426	27.3713	498.157	169.702	15366.2	9169.36	49.2683	2622.16	49.2683
NV22 BDEV	BDEVs	Q331K	Male	6m	359.166	71.8334	260.396	17.9584	260.396	413.041	80.8125	71.8334	170.604	2756.6	44.8959	1661.14	44.8959	26.9376	628.541	125.708	12956.9	7695.14	44.8959	1957.46	80.8125
NV23 BDEV	BDEVs	Q331K	Male	6m	427.776	29.2998	263.698	11.72	334.017	404.336	58.5996	82.0394	140.639	3070.61	17.5799	445.356	41.0197	23.4399	345.737	58.5996	16724.3	10348.7	123.059	2818.63	76.1794
NV24 BDEV	BDEVs	Q331K	Male	6m	345.536	77.8235	298.842	12.4518	211.68	351.762	71.5977	99.6141	168.099	2988.42	9.33891	591.458	59.1459	37.3553	311.294	121.405	21933.8	12112.4	115.179	2213.3	127.631
NV33 BDEV	BDEVs	Q331K	Male	6m	207.83	48.3988	307.474	8.54105	370.108	620.642	48.3988	65.4807	230.606	1833.46	17.082	395.731	65.4807	37.0109	222.065	122.42	7470.48	5013.54	102.491	1597.16	170.819
NV34 BDEV	BDEVs	Q331K	Male	6m	274.298	54.3654	239.702	24.7116	266.884	719.105	103.788	101.317	205.106	3212.5	4.9424	308.894	46.952	24.7116	328.663	177.923	7532.07	4606.23	168.038	2238.86	170.51
NV35 BDEV	BDEVs	WT	Female	6m	515.657	31.1173	426.75	26.672	471.203	400.078	53.3439	88.9064	164.477	2738.31	8.89073	502.321	35.5626	26.672	306.727	168.922	10886.6	8232.72	66.6798	2044.84	133.36
NV36 BDEV	BDEVs	WT	Female	6m	334.029	8.35083	409.186	16.7016	392.484	551.148	8.35083	91.8581	91.8581	3064.72	83.5074	1327.77	66.8059	25.0523	1068.89	75.1567	10238	7908.14	33.403	2455.11	167.015
NV37 BDEV	BDEVs	WT	Female	6m	382.777	43.0626	406.701	62.2014	296.653	521.534	76.5556	76.5556	191.389	3784.71	57.4167	1277.52	129.187	52.632	736.847	129.187	9899.58	7090.95	43.0626	2373.22	124.403
NV38 BDEV	BDEVs	WT	Male	6m	450.023	40.9113	343.654	24.5468	270.014	343.654	40.9113	32.729	122.734	3076.52	8.18234	1358.25	81.8225	40.9113	1120.97	171.827	17722.7	12559.7	57.2757	2176.47	139.098
NV39 BDEV	BDEVs	WT	Male	6m	468.762	50.6771	307.229	9.50204	259.72	323.066	66.5137	76.0156	145.697	3556.89	0.0001	212.21	28.5059	25.3386	269.222	107.689	17895.3	11272.5	60.1791	2926.6	72.8483
NV40 BDEV	BDEVs	WT	Male	6m	327.464	37.578	370.411	10.7366	249.625	488.512	56.3669	34.8938	169.101	3282.7	10.7366	375.779	48.3145	18.789	531.459	136.891	16896.6	12051.8	72.4717	2729.76	155.68

Sample na	Sample typ	Mutation	Sex	Timepoint	mmu-miR-	mmu-miR-	mmu-miR-	mmu-miR-	mmu-miR-	mmu-miR-	mmu-miR-	mmu-miR-	mmu-miR-	mmu-miR-	mmu-miR-	mmu-miR-	mmu-miR-	mmu-miR-	mmu-miR-	mmu-miR-	mmu-miR-	mmu-miR-99b-5p
NV1 BDEV1	BDEVs	Q331K	Female	3m	61.6532	58.0266	0.0001	134.186	21.76	5929.58	54624.7	29.0133	377.172	61.6532	54.3999	1233.06	65.2799	166.826	2136.1	1265.7	61.6532	678.184
NV2 BDEV2	BDEVs	Q331K	Female	3m	70.7063	27.7775	5.05054	227.27	31.5654	5659.02	52549.9	10.101	208.331	12.6262	41.6663	1135.09	16.414	18.9393	2228.51	1284.08	31.5654	325.754
NV4 BDEV4	BDEVs	Q331K	Male	3m	99.9522	108.282	0.0001	91.6229	8.32944	6163.71	67459.3	8.32944	932.886	33.3175	66.6348	1157.78	49.9762	816.276	2332.22	849.593	0.0001	699.665
NV5 BDEV5	BDEVs	Q331K	Male	3m	58.6047	68.3722	19.535	131.861	14.6513	6578.37	57642.5	4.88382	600.697	4.88382	43.9536	1103.72	48.8373	468.837	1904.65	854.651	19.535	688.604
NV6 BDEV6	BDEVs	Q331K	Male	3m	63.2394	56.2128	14.0533	126.479	7.02669	5789.91	52587	14.0533	541.048	42.1597	21.0799	1011.83	28.1065	2016.63	2115	498.888	14.0533	330.25
NV7 BDEV7	BDEVs	WT	Female	3m	44.5066	66.7599	16.69	172.463	33.38	5329.65	55015.6	5.56341	578.585	61.1965	55.6332	1173.86	44.5066	11.1267	1730.19	561.895	38.9433	394.995
NV8 BDEV8	BDEVs	WT	Female	3m	65.9981	102.664	7.33321	190.661	7.33321	4443.87	60894.2	14.6663	667.314	14.6663	80.6644	777.31	21.9994	43.9988	1517.95	1187.96	29.3326	843.308
NV17 BDEV	BDEVs	WT	Female	3m	88.0565	62.8975	12.5796	75.477	25.1591	5723.67	64243.4	0.0001	377.385	62.8975	37.7386	1270.53	12.5796	616.395	1106.99	528.339	25.1591	339.646
NV18 BDEV	BDEVs	WT	Male	3m	66.9922	75.3663	8.37412	100.488	8.37412	7076.04	65769.5	8.37412	468.945	100.488	50.2442	1331.47	50.2442	427.075	1934.4	577.807	25.1222	577.807
NV19 BDEV	BDEVs	WT	Male	3m	110.265	47.2563	19.6902	86.6364	35.4422	6560.73	65213.5	15.7522	366.236	19.6902	55.1323	1177.47	19.6902	133.893	2087.15	744.285	43.3183	342.607
NV20 BDEV	BDEVs	WT	Male	3m	57.9262	42.6825	6.09759	124.999	42.6825	6374.92	59990.1	9.14633	536.579	21.3413	21.3413	887.184	42.6825	30.4875	2088.39	713.406	36.585	411.58
NV21 BDEV	BDEVs	Q331K	Female	6m	131.382	82.1138	5.47435	191.599	21.8971	6952.29	69446.3	16.4228	728.075	98.5366	43.7941	1538.26	38.3198	147.805	1702.49	1062	65.6911	662.384
NV22 BDEV	BDEVs	Q331K	Male	6m	98.7708	125.708	17.9584	161.625	0.0001	6105.83	64937.3	0.0001	844.041	98.7708	44.8959	1562.37	62.8542	1203.21	1679.1	835.062	44.8959	556.708
NV23 BDEV	BDEVs	Q331K	Male	6m	46.8797	29.2998	0.0001	134.779	17.5799	6322.88	58224.4	5.86005	322.297	41.0197	35.1598	1406.39	58.5996	1986.52	1910.34	1388.81	35.1598	597.715
NV24 BDEV	BDEVs	Q331K	Male	6m	174.325	37.3553	9.33891	93.3882	31.1295	10580.9	60758.3	31.1295	357.988	24.9036	71.5977	1621.84	31.1295	161.873	1581.37	1634.29	46.6942	728.427
NV33 BDEV	BDEVs	Q331K	Male	6m	139.502	56.9398	2.84708	56.9398	5.69407	11960.2	55632.9	5.69407	284.698	22.776	34.1639	1668.33	88.2566	358.72	1742.35	1594.31	45.5518	1101.78
NV34 BDEV	BDEVs	Q331K	Male	6m	155.683	51.8943	7.41355	84.0192	19.7693	10954.6	53404	4.9424	610.374	22.2405	106.26	1611.19	34.5962	1344.31	1769.34	2016.46	61.7789	669.682
NV35 BDEV	BDEVs	WT	Female	6m	106.688	53.3439	4.44541	75.5704	17.7814	9157.35	55859.8	8.89073	342.289	22.2267	57.7892	1400.27	44.4532	22.2267	2151.53	822.383	40.0079	480.094
NV36 BDEV	BDEVs	WT	Female	6m	33.403	133.612	0.0001	125.261	16.7016	6396.66	43615.9	16.7016	885.178	75.1567	16.7016	835.073	33.403	350.731	2246.35	350.731	66.8059	467.641
NV37 BDEV	BDEVs	WT	Female	6m	95.6944	90.9097	28.7084	167.465	14.3543	8038.32	56646.3	23.9237	1114.84	90.9097	43.0626	1066.99	71.7709	47.8473	2263.17	1344.51	38.2778	564.597
NV38 BDEV	BDEVs	WT	Male	6m	49.0935	122.734	16.3646	81.8225	24.5468	6905.81	59386.7	32.729	859.135	65.458	40.9113	916.41	73.6402	392.747	2061.92	654.579	24.5468	540.028
NV39 BDEV	BDEVs	WT	Male	6m	50.6771	31.6732	6.33473	152.031	9.50204	6974.42	60242.3	6.33473	383.245	9.50204	19.004	1184.58	22.1713	212.21	1799.03	1498.14	41.1752	304.062
NV40 BDEV	BDEVs	WT	Male	6m	91.2606	56.3669	10.7366	174.469	18.789	8629.49	54995.2	16.1049	394.568	13.4208	34.8938	985.077	26.8414	42.9462	1827.89	1121.97	34.8938	303.307
