# Supplementary material for: A Panel of miRNA Biomarkers Common to Serum and Brain-Derived Extracellular Vesicles Identified in Mouse Model of Amyotrophic Lateral Sclerosis
Source: Mol Neurobiol. 2024 Jan 22;61(8):5901–15. doi: 10.1007/s12035-023-03857-z (PMC11249427; doi:10.1007/s12035-023-03857-z)
Supplement: Supplementary file 4 — Supplementary file4 (PDF 246 KB) [file 12035_2023_3857_MOESM4_ESM.pdf]

Sample na	Sample ty	Mutation	Sex	Timepoint	mmu-let-7	mmu-let-7	mmu-let-7	mmu-let-7	mmu-let-7	mmu-let-7	mmu-let-7	mmu-miR-	mmu-miR-	mmu-miR-	mmu-miR-	mmu-miR-	mmu-miR-	mmu-miR-	mmu-miR-	mmu-miR-	mmu-miR-	mmu-miR-	mmu-miR-	mmu-miR-	mmu-miR-
NV9 Serum	Serum EVs	Q331K	Female	3m	3120.13	34321.4	4680.19	3120.13	3120.13	1040.04	24961	520.021	0.0001	6760.27	1040.04	1040.04	4160.17	1040.04	11960.5	5200.21	3120.13	7280.29	1542.19	17.874	1560.06
NV11 Seru	Serum EVs	Q331K	Female	3m	4237.29	23728.8	4237.29	847.458	1694.92	2542.37	27966.1	0.0001	1694.92	847.458	2542.37	3389.83	5084.75	847.458	6779.66	2542.37	3389.83	5084.75	4146.44	90.8483	7627.12
NV12 Seru	Serum EVs	Q331K	Male	3m	12867.6	67095.6	5514.71	0.0001	10110.3	3676.47	17463.2	0.0001	0.0001	1838.24	3676.47	919.118	2757.35	919.118	2757.35	1838.24	4595.59	5514.71	809.689	109.429	11029.4
NV13 Seru	Serum EVs	Q331K	Male	3m	6940.26	21424.3	4828	4526.25	3922.75	3621	52806.3	7543.76	0.0001	2715.75	4828	905.251	5431.51	1207	8750.76	3319.25	2715.75	3017.5	298.293	3.4573	603.501
NV14 Seru	Serum EVs	Q331K	Male	3m	15228.4	30456.9	25380.7	2538.07	16074.5	7614.21	22842.6	1692.05	1692.05	0.0001	3384.09	0.0001	0.0001	0.0001	20304.6	7614.21	4230.12	15228.4	745.298	100.726	3384.09
NV15 Seru	Serum EVs	WT	Female	3m	6003.43	17438.5	4288.16	3144.65	2001.14	2287.02	24013.7	0.0001	571.755	2858.78	3716.41	1143.51	6289.31	2572.9	8576.33	12864.5	7146.94	21154.9	4521.64	52.4057	1143.51
NV16 Seru	Serum EVs	WT	Female	3m	4187.6	23729.8	8654.38	1395.87	3629.26	1395.87	35175.9	837.521	1116.69	2791.74	4187.6	558.347	10608.6	4466.78	12562.8	13400.3	6141.82	9491.9	1655.85	19.1913	2791.74
NV25 Seru	Serum EVs	WT	Female	3m	8879.02	37735.8	9988.9	1109.88	8879.02	4439.51	24417.3	0.0001	0.0001	1109.88	0.0001	4439.51	4439.51	2219.76	13318.5	15538.3	4439.51	8879.02	0.0001	0.0001	5549.39
NV26 Seru	Serum EVs	WT	Male	3m	3807.29	41445	12183.3	3589.73	4242.41	3154.61	12074.5	1414.14	2610.71	1849.25	4677.52	870.237	5982.88	5547.76	207225	3045.83	2284.37	8049.69	1613	18.6947	652.678
NV27 Seru	Serum EVs	WT	Male	3m	5291.01	55555.6	5952.38	0.0001	6613.76	0.0001	17195.8	661.376	3968.25	2645.5	661.376	4629.63	3306.88	3306.88	12566.1	6613.76	2645.5	7936.51	3268.99	37.8875	661.376
NV28 Seru	Serum EVs	WT	Male	3m	6459.95	28854.4	8182.6	1722.65	4521.96	1722.65	12489.2	1722.65	2368.65	1291.99	215.332	430.663	3229.97	4952.63	48664.9	4091.3	3229.97	4521.96	3405.83	39.4736	6459.95
NV29 Seru	Serum EVs	Q331K	Female	6m	8943.09	27642.3	13008.1	2439.02	1626.02	1626.02	23577.2	3252.03	813.008	2439.02	2439.02	813.008	13821.1	6504.07	37398.4	8943.09	5691.06	10569.1	1607.39	18.6296	0.0001
NV30 Seru	Serum EVs	Q331K	Female	6m	3994.67	25965.4	23302.3	1331.56	3994.67	5326.23	14647.1	0.0001	1997.34	1997.34	2663.12	1331.56	1331.56	1331.56	9986.68	5992.01	4660.45	6657.79	652.721	13.0576	4660.45
NV31 Seru	Serum EVs	Q331K	Female	6m	9116.13	32501	5152.6	396.354	8323.42	3963.54	36068.2	792.707	7530.72	5152.6	4756.24	792.707	4756.24	3170.83	14268.7	9512.49	2378.12	8323.42	1959.06	22.7055	792.707
NV32 Seru	Serum EVs	Q331K	Male	6m	12927.4	39199.3	6672.23	3336.11	12927.4	2919.1	30442	1668.06	8757.3	7089.24	2919.1	2919.1	4587.16	2085.07	17097.6	5421.18	3336.11	11259.4	2885.65	33.4447	2085.07
NV41 Seru	Serum EVs	Q331K	Male	6m	4749.47	14248.4	9023.99	1899.79	3799.57	1424.84	25172.2	474.947	1424.84	1424.84	949.893	949.893	6174.31	6174.31	19472.8	16148.2	3324.63	25172.2	7512.08	87.0648	1424.84
NV42 Seru	Serum EVs	Q331K	Male	6m	78431.4	0.0001	39215.7	19607.8	39215.7	0.0001	0.0001	0.0001	0.0001	19607.8	0.0001	19607.8	0.0001	0.0001	0.0001	0.0001	0.0001	0.0001	0.0001	0.0001	0.0001
NV43 Seru	Serum EVs	WT	Female	6m	8163.27	26122.5	17959.2	11428.6	6530.61	3265.31	26122.5	1632.65	1632.65	4897.96	3265.31	1632.65	4897.96	6530.61	42449	8163.27	8163.27	4897.96	0.0001	0.0001	3265.31
NV44 Seru	Serum EVs	WT	Female	6m	5148.01	16731	6435.01	1287	9009.01	5148.01	25740	0.0001	5148.01	2574	0.0001	2574	10296	5148.01	10296	2574	5148.01	11583	3816.77	44.2362	2574
NV45 Seru	Serum EVs	WT	Female	6m	6920.42	20761.2	1730.1	3460.21	1730.1	1730.1	20761.2	1730.1	1730.1	3460.21	1730.1	3460.21	1730.1	0.0001	6920.42	10380.6	3460.21	12110.7	1524.12	205.983	1730.1
NV46 Seru	Serum EVs	WT	Male	6m	0.0001	0.0001	0.0001	0.0001	0.0001	0.0001	0.0001	0.0001	0.0001	0.0001	0.0001	0.0001	0.0001	333333	0.0001	0.0001	0.0001	0.0001	0.0001	0.0001	0.0001
NV47 Seru	Serum EVs	WT	Male	6m	4078.3	21207.2	10603.6	1631.32	3262.64	1631.32	19575.9	1631.32	2446.98	815.661	2446.98	815.661	10603.6	7340.95	21207.2	10603.6	5709.63	17128.9	4031.58	46.7259	0.0001
NV48 Seru	Serum EVs	WT	Male	6m	4507.71	23962	6642.94	711.744	3084.22	2846.97	7591.93	1897.98	474.496	1897.98	2135.23	1186.24	6168.45	7829.18	264294	8066.43	4744.96	7591.93	1876.24	21.7456	2846.97

Sample nai	Sample typ	Mutation	Sex	Timepoint	mmu-miR-	mmu-miR-	mmu-miR-	mmu-miR-	mmu-miR-	mmu-miR-	mmu-miR-	mmu-miR-	mmu-miR-	mmu-miR-	mmu-miR-	mmu-miR-	mmu-miR-	mmu-miR-	mmu-miR-	mmu-miR-	mmu-miR-	mmu-miR-	mmu-miR-	mmu-miR-	mmu-miR-
NV9 Serum	Serum EVs	Q331K	Female	3m	520.021	2080.08	7800.31	3640.15	6760.27	2600.1	4160.17	1040.04	28081.1	1040.04	3640.15	10400.4	0.0001	1040.04	2600.1	1560.06	39001.6	520.021	2080.08	520.021	2600.1
NV11 Seru	Serum EVs	Q331K	Female	3m	4237.29	3389.83	11016.9	847.458	11016.9	847.458	847.458	5084.75	31355.9	4237.29	3389.83	7627.12	0.0001	2542.37	0.0001	0.0001	26271.2	0.0001	1694.92	0.0001	847.458
NV12 Seru	Serum EVs	Q331K	Male	3m	0.0001	0.0001	3676.47	2757.35	4595.59	1838.24	0.0001	2757.35	15625	10110.3	919.118	0.0001	1838.24	2757.35	0.0001	1838.24	39522.1	0.0001	1838.24	1838.24	1838.24
NV13 Seru	Serum EVs	Q331K	Male	3m	1810.5	301.75	8449.01	301.75	14484	603.501	2112.25	2112.25	19613.8	5733.26	905.251	23234.8	1508.75	603.501	1207	1508.75	47374.8	1508.75	905.251	1810.5	603.501
NV14 Seru	Serum EVs	Q331K	Male	3m	7614.21	0.0001	5922.17	0.0001	12690.4	0.0001	16074.5	0.0001	16074.5	3384.09	0.0001	846.024	2538.07	846.024	0.0001	0.0001	5076.14	5076.14	3384.09	0.0001	846.024
NV15 Seru	Serum EVs	WT	Female	3m	3144.65	1715.27	14293.9	1143.51	4574.04	2001.14	5145.8	1429.39	35734.7	5431.68	1429.39	22012.6	2287.02	2858.78	1143.51	571.755	38021.7	1715.27	2001.14	2858.78	2001.14
NV16 Seru	Serum EVs	WT	Female	3m	1675.04	279.174	18146.3	1675.04	6141.82	1116.69	7258.51	2512.56	25963.1	3629.26	2791.74	13679.5	3908.43	279.174	837.521	1395.87	30429.9	0.0001	837.521	1675.04	1116.69
NV25 Seru	Serum EVs	WT	Female	3m	3329.63	1109.88	14428.4	1109.88	17758	2219.76	1109.88	5549.39	9988.9	0.0001	0.0001	21087.7	0.0001	1109.88	0.0001	0.0001	54384	1109.88	0.0001	5549.39	4439.51
NV26 Seru	Serum EVs	WT	Male	3m	1849.25	217.559	2066.81	0.0001	5438.98	761.457	2719.49	2719.49	18166.2	19797.9	2610.71	7614.57	1196.58	3154.61	435.119	2937.05	32851.4	217.559	1849.25	326.339	1849.25
NV27 Seru	Serum EVs	WT	Male	3m	2645.5	1984.13	12566.1	1322.75	9259.26	3306.88	1984.13	661.376	15211.6	3968.25	2645.5	7275.13	0.0001	661.376	1322.75	1984.13	40343.9	661.376	1322.75	661.376	661.376
NV28 Seru	Serum EVs	WT	Male	3m	3014.64	215.332	9259.26	1937.98	11197.2	430.663	3660.64	6890.61	6459.95	5813.95	1291.99	5598.62	0.0001	645.995	215.332	2153.32	41989.7	2368.65	645.995	861.327	7536.61
NV29 Seru	Serum EVs	Q331K	Female	6m	3252.03	813.008	13008.1	0.0001	7317.07	813.008	3252.03	7317.07	21138.2	4878.05	2439.02	26016.3	1626.02	1626.02	813.008	2439.02	35772.4	0.0001	1626.02	3252.03	3252.03
NV30 Seru	Serum EVs	Q331K	Female	6m	6657.79	665.779	25299.6	0.0001	4660.45	1331.56	665.779	3994.67	5326.23	3994.67	4660.45	6657.79	0.0001	665.779	1331.56	1997.34	85885.5	1331.56	1331.56	0.0001	4660.45
NV31 Seru	Serum EVs	Q331K	Female	6m	3170.83	1981.77	9512.49	396.354	13872.4	1585.41	1981.77	2774.47	29330.2	9512.49	3963.54	17439.6	2774.47	396.354	1981.77	1585.41	105430	1189.06	2774.47	1189.06	3170.83
NV32 Seru	Serum EVs	Q331K	Male	6m	0.0001	417.014	10008.3	417.014	9591.33	2085.07	2085.07	4170.14	31276.1	5004.17	4170.14	10008.3	2502.09	1251.04	3336.11	4587.16	156797	1668.06	834.028	834.028	2919.1
NV41 Seru	Serum EVs	Q331K	Male	6m	949.893	949.893	7124.2	949.893	11398.7	474.947	8549.04	5224.41	58418.4	3324.63	2374.73	18997.9	1899.79	949.893	1424.84	949.893	55568.8	949.893	2374.73	1424.84	2849.68
NV42 Seru	Serum EVs	Q331K	Male	6m	0.0001	0.0001	0.0001	0.0001	0.0001	0.0001	39215.7	0.0001	0.0001	0.0001	0.0001	0.0001	0.0001	0.0001	0.0001	0.0001	39215.7	0.0001	0.0001	0.0001	0.0001
NV43 Seru	Serum EVs	WT	Female	6m	4897.96	0.0001	9795.92	0.0001	24489.8	1632.65	9795.92	3265.31	19591.8	6530.61	0.0001	21224.5	0.0001	0.0001	1632.65	0.0001	31020.4	9795.92	3265.31	0.0001	1632.65
NV44 Seru	Serum EVs	WT	Female	6m	6435.01	1287	9009.01	0.0001	24453	0.0001	3861	1287	25740	3861	2574	11583	2574	1287	0.0001	2574	60489.1	1287	3861	0.0001	2574
NV45 Seru	Serum EVs	WT	Female	6m	0.0001	0.0001	13840.8	0.0001	17301	0.0001	8650.52	1730.1	24221.5	5190.31	3460.21	10380.6	0.0001	0.0001	1730.1	1730.1	20761.2	1730.1	0.0001	0.0001	6920.42
NV46 Seru	Serum EVs	WT	Male	6m	0.0001	0.0001	0.0001	0.0001	0.0001	0.0001	0.0001	0.0001	0.0001	0.0001	0.0001	0.0001	0.0001	0.0001	0.0001	0.0001	0.0001	0.0001	0.0001	0.0001	0.0001
NV47 Seru	Serum EVs	WT	Male	6m	3262.64	815.661	8972.27	1631.32	22022.8	815.661	5709.63	7340.95	42414.4	1631.32	3262.64	21207.2	815.661	815.661	2446.98	1631.32	38336.1	815.661	2446.98	4078.3	0.0001
NV48 Seru	Serum EVs	WT	Male	6m	1660.74	237.248	2846.97	237.248	6405.69	711.744	4033.21	3795.97	44602.6	2609.73	711.744	5456.7	237.248	2135.23	711.744	237.248	13997.6	237.248	1423.49	474.496	711.744

Sample nai	Sample typ	Mutation	Sex	Timepoint	mmu-miR-	mmu-miR-	mmu-miR-	mmu-miR-	mmu-miR-	mmu-miR-	mmu-miR-	mmu-miR-	mmu-miR-	mmu-miR-	mmu-miR-	mmu-miR-	mmu-miR-	mmu-miR-	mmu-miR-	mmu-miR-	mmu-miR-	mmu-miR-	mmu-miR-	mmu-miR-	mmu-miR-
NV9 Serum	Serum EVs	Q331K	Female	3m	0.0001	9360.38	21840.9	2600.1	1040.04	2600.1	289.662	2830.46	3120.13	1560.06	1040.04	8840.35	113885	520.021	3120.13	40041.6	6760.27	28601.1	520.021	34321.4	48361.9
NV11 Seru	Serum EVs	Q331K	Female	3m	3389.83	8474.58	19491.5	5932.2	3389.83	847.458	0.0001	0.0001	847.458	0.0001	847.458	1694.92	116102	1694.92	847.458	37288.1	11016.9	72033.9	0.0001	22881.4	56779.7
NV12 Seru	Serum EVs	Q331K	Male	3m	1838.24	5514.71	3676.47	0.0001	0.0001	0.0001	242.976	676.142	919.118	919.118	2757.35	134191	148897	919.118	0.0001	39522.1	6433.82	72610.3	919.118	13786.8	31250
NV13 Seru	Serum EVs	Q331K	Male	3m	3621	5733.26	15691	905.251	905.251	603.501	168.081	1642.42	2414	301.75	301.75	1508.75	19915.5	603.501	1207	19915.5	6940.26	35304.8	1207	20820.8	17199.8
NV14 Seru	Serum EVs	Q331K	Male	3m	846.024	846.024	20304.6	846.024	0.0001	2538.07	0.0001	0.0001	2538.07	0.0001	0.0001	1692.05	104907	0.0001	0.0001	23688.7	15228.4	77834.2	846.024	25380.7	42301.2
NV15 Seru	Serum EVs	WT	Female	3m	571.755	15437.4	36020.6	2572.9	1715.27	857.633	2547.84	24896.4	1715.27	1143.51	285.878	4288.16	31732.4	285.878	1143.51	32590	8004.57	56603.8	1429.39	60034.3	46026.3
NV16 Seru	Serum EVs	WT	Female	3m	837.521	11725.3	23729.8	2233.39	4745.95	837.521	155.505	1519.54	5025.13	1116.69	1116.69	7258.51	87381.4	0.0001	558.347	39084.3	6141.82	34896.7	1675.04	48297	54159.7
NV25 Seru	Serum EVs	WT	Female	3m	3329.63	9988.9	14428.4	0.0001	0.0001	0.0001	721.262	7047.89	0.0001	1109.88	0.0001	4439.51	87680.4	1109.88	0.0001	24417.3	7769.15	83240.8	0.0001	17758	49944.5
NV26 Seru	Serum EVs	WT	Male	3m	326.339	12618.4	40466	5438.98	4677.52	217.559	111.086	1085.49	1196.58	4024.85	3045.83	6309.22	34809.5	2937.05	1087.8	72664.8	3154.61	33939.2	0.0001	23931.5	68313.6
NV27 Seru	Serum EVs	WT	Male	3m	0.0001	12566.1	21164	0.0001	661.376	661.376	307	2999.88	1984.13	1322.75	1984.13	29761.9	111111	661.376	0.0001	61507.9	7275.13	90608.5	661.376	23809.5	72751.3
NV28 Seru	Serum EVs	WT	Male	3m	1937.98	13350.6	12489.2	1722.65	4306.63	645.995	299.86	2930.11	1722.65	430.663	645.995	18733.8	152885	0.0001	645.995	51033.6	12919.9	101637	0.0001	26485.8	67614.1
NV29 Seru	Serum EVs	Q331K	Female	6m	813.008	8943.09	25203.3	4065.04	1626.02	0.0001	4000.29	39089.1	6504.07	813.008	0.0001	3252.03	27642.3	3252.03	813.008	21138.2	5691.06	38211.4	1626.02	26829.3	73170.7
NV30 Seru	Serum EVs	Q331K	Female	6m	1331.56	17976	7323.57	665.779	0.0001	665.779	158.675	1172.88	2663.12	665.779	665.779	2663.12	23968	1997.34	1331.56	12649.8	11984	59254.3	1331.56	7323.57	30625.8
NV31 Seru	Serum EVs	Q331K	Female	6m	1585.41	15061.4	27348.4	1585.41	792.707	2378.12	110.388	1078.67	2774.47	0.0001	0.0001	3170.83	29330.2	1585.41	1189.06	27348.4	13476	53507.7	1585.41	54300.4	40824.4
NV32 Seru	Serum EVs	Q331K	Male	6m	1251.04	7089.24	14178.5	834.028	1668.06	1251.04	154.857	1513.2	3336.11	0.0001	0.0001	1251.04	35029.2	417.014	1251.04	27940	9174.31	56713.9	0.0001	24186.8	23769.8
NV41 Seru	Serum EVs	Q331K	Male	6m	1899.79	14723.3	27072	2849.68	3324.63	1899.79	132.277	1292.56	3799.57	3799.57	0.0001	6649.25	32296.4	474.947	3799.57	44645	10448.8	64592.7	474.947	64592.7	54143.9
NV42 Seru	Serum EVs	Q331K	Male	6m	0.0001	0.0001	0.0001	0.0001	0.0001	0.0001	0.0001	0.0001	0.0001	0.0001	0.0001	0.0001	0.0001	0.0001	0.0001	0.0001	0.0001	19607.8	0.0001	0.0001	0.0001
NV43 Seru	Serum EVs	WT	Female	6m	0.0001	8163.27	16326.5	1632.65	3265.31	0.0001	389.111	2876.2	3265.31	0.0001	1632.65	9795.92	34285.7	1632.65	1632.65	19591.8	13061.2	53877.6	1632.65	29387.8	40816.3
NV44 Seru	Serum EVs	WT	Female	6m	0.0001	20592	34749	3861	0.0001	1287	358.443	3502.56	0.0001	0.0001	1287	2574	23166	0.0001	2574	55341.1	7722.01	75933.1	0.0001	38610	46332
NV45 Seru	Serum EVs	WT	Female	6m	0.0001	10380.6	5190.31	1730.1	0.0001	0.0001	0.0001	0.0001	0.0001	0.0001	1730.1	8650.52	41522.5	0.0001	1730.1	22491.3	3460.21	83045	1730.1	20761.2	58823.5
NV46 Seru	Serum EVs	WT	Male	6m	0.0001	0.0001	0.0001	0.0001	0.0001	0.0001	0.0001	0.0001	0.0001	0.0001	0.0001	0.0001	0.0001	0.0001	0.0001	0.0001	166667	0.0001	0.0001	0.0001	0.0001
NV47 Seru	Serum EVs	WT	Male	6m	0.0001	8156.61	30179.4	815.661	2446.98	815.661	302.893	2959.75	2446.98	2446.98	815.661	4893.96	80750.4	0.0001	1631.32	64437.2	12234.9	77487.8	1631.32	48939.6	42414.4
NV48 Seru	Serum EVs	WT	Male	6m	0.0001	3321.47	21352.3	1660.74	4033.21	0.0001	484.557	4734.9	5456.7	948.992	237.248	11150.7	122894	1660.74	0.0001	25622.8	9252.67	74970.3	237.248	18030.8	37959.7

Sample na	Sample typ	Mutation	Sex	Timepoint	mmu-miR-	mmu-miR-	mmu-miR-	mmu-miR-	mmu-miR-	mmu-miR-	mmu-miR-	mmu-miR-	mmu-miR-	mmu-miR-	mmu-miR-	mmu-miR-	mmu-miR-	mmu-miR-	mmu-miR-	mmu-miR-	mmu-miR-	mmu-miR-	mmu-miR-	mmu-miR-	mmu-miR-
NV9 Serum	Serum EVs	Q331K	Female	3m	19760.8	520.021	36401.5	3640.15	1040.04	1040.04	5720.23	2080.08	21320.9	1040.04	0.0001	1040.04	1040.04	0.0001	4680.19	1040.04	22880.9	1560.06	13520.5	0.0001	0.0001
NV11 Seru	Serum EVs	Q331K	Female	3m	20339	1694.92	24576.3	5932.2	1694.92	1694.92	2542.37	1694.92	23728.8	847.458	1694.92	11016.9	0.0001	1694.92	4237.29	1694.92	44067.8	3389.83	20339	847.458	3389.83
NV12 Seru	Serum EVs	Q331K	Male	3m	15625	1838.24	1838.24	9191.18	5514.71	2757.35	10110.3	919.118	2757.35	0.0001	919.118	0.0001	0.0001	0.0001	919.118	0.0001	43198.5	0.0001	11029.4	0.0001	1838.24
NV13 Seru	Serum EVs	Q331K	Male	3m	6940.26	0.0001	25950.5	3621	6940.26	603.501	905.251	1207	6638.51	0.0001	603.501	2112.25	301.75	301.75	1810.5	1508.75	18708.5	1508.75	21424.3	0.0001	301.75
NV14 Seru	Serum EVs	Q331K	Male	3m	16920.5	3384.09	4230.12	3384.09	0.0001	0.0001	846.024	1692.05	17766.5	0.0001	0.0001	8460.24	0.0001	0.0001	4230.12	0.0001	58375.6	4230.12	5922.17	2538.07	0.0001
NV15 Seru	Serum EVs	WT	Female	3m	6289.31	2287.02	25157.2	1429.39	4288.16	571.755	6575.19	1143.51	18296.2	2287.02	1143.51	1429.39	2572.9	571.755	3430.53	2001.14	19725.6	2572.9	14293.9	571.755	571.755
NV16 Seru	Serum EVs	WT	Female	3m	13400.3	1675.04	24567.3	1675.04	3070.91	837.521	3629.26	279.174	19542.2	1675.04	0.0001	1954.22	1116.69	1395.87	5862.65	837.521	17867.1	1116.69	15075.4	279.174	0.0001
NV25 Seru	Serum EVs	WT	Female	3m	15538.3	554.939	7769.15	7769.15	6659.27	0.0001	1109.88	1109.88	4439.51	0.0001	0.0001	0.0001	0.0001	0.0001	0.0001	1109.88	49944.5	2219.76	7769.15	1109.88	0.0001
NV26 Seru	Serum EVs	WT	Male	3m	4024.85	2066.81	12183.3	2719.49	5874.1	543.898	4024.85	217.559	8267.25	1305.36	1196.58	217.559	217.559	217.559	1305.36	108.78	13923.8	979.017	8158.47	1087.8	435.119
NV27 Seru	Serum EVs	WT	Male	3m	27116.4	330.688	7275.13	3306.88	0.0001	0.0001	3306.88	661.376	7936.51	3968.25	661.376	1984.13	1322.75	661.376	7936.51	1984.13	48280.4	3968.25	8597.88	1322.75	1984.13
NV28 Seru	Serum EVs	WT	Male	3m	9689.92	3014.63	7105.94	3229.97	1291.99	2153.32	3875.97	861.327	6244.62	861.327	1076.66	1291.99	1076.66	215.332	2153.32	1291.99	37898.4	2153.32	16795.9	1507.32	430.663
NV29 Seru	Serum EVs	Q331K	Female	6m	6504.07	1219.51	19512.2	4878.05	4065.04	0.0001	3252.03	0.0001	7317.07	813.008	813.008	813.008	1626.02	813.008	4065.04	2439.02	16260.2	3252.03	13821.1	2439.02	813.008
NV30 Seru	Serum EVs	Q331K	Female	6m	7323.57	1331.56	23968	3994.67	2663.12	1331.56	1331.56	0.0001	7989.35	665.779	1331.56	3328.89	665.779	0.0001	2663.12	2663.12	28628.5	665.779	11984	1331.56	665.779
NV31 Seru	Serum EVs	Q331K	Female	6m	3963.54	1585.41	14665.1	5152.6	3567.18	792.707	2378.12	396.354	16646.8	1585.41	396.354	1585.41	1981.77	2378.12	2774.47	1189.06	9116.13	1981.77	19025	396.354	0.0001
NV32 Seru	Serum EVs	Q331K	Male	6m	2502.09	1251.04	14595.5	5004.17	7506.26	1251.04	417.014	417.014	11676.4	1668.06	834.028	1251.04	1251.04	417.014	3336.11	834.028	14595.5	2085.07	15429.5	1668.06	1251.04
NV41 Seru	Serum EVs	Q331K	Male	6m	5224.41	0.000101	13773.5	2849.68	3799.57	474.947	4274.52	474.947	22322.5	1899.79	474.947	2374.73	474.947	1424.84	2374.73	2374.73	16623.1	5224.41	15673.2	1424.84	474.947
NV42 Seru	Serum EVs	Q331K	Male	6m	0.0001	0.0001	0.0001	0.0001	0.0001	0.0001	0.0001	0.0001	39215.7	0.0001	0.0001	0.0001	0.0001	0.0001	0.0001	0.0001	0.0001	0.0001	0.0001	0.0001	0.0001
NV43 Seru	Serum EVs	WT	Female	6m	6530.61	3265.31	19591.8	0.0001	3265.31	0.0001	6530.61	0.0001	24489.8	1632.65	0.0001	3265.31	0.0001	1632.65	8163.27	3265.31	24489.8	3265.31	17959.2	1632.65	0.0001
NV44 Seru	Serum EVs	WT	Female	6m	7722.01	2574	19305	3861	1287	0.0001	3861	0.0001	29601	1287	2574	3861	1287	1287	9009.01	0.0001	16731	3861	21879	0.0001	0.0001
NV45 Seru	Serum EVs	WT	Female	6m	12110.7	1730.1	20761.2	0.0001	1730.1	1730.1	1730.1	0.0001	12110.7	6920.42	0.0001	6920.42	5190.31	3460.21	1730.1	0.0001	22491.3	6920.42	24221.5	0.0001	1730.1
NV46 Seru	Serum EVs	WT	Male	6m	0.0001	0.0001	0.0001	0.0001	0.0001	0.0001	0.0001	0.0001	0.0001	0.0001	0.0001	0.0001	0.0001	0.0001	0.0001	0.0001	0.0001	0.0001	0.0001	0.0001	0.0001
NV47 Seru	Serum EVs	WT	Male	6m	9787.93	2446.98	15497.6	2446.98	1631.32	815.661	3262.64	0.0001	22838.5	1631.32	0.0001	4078.3	815.661	815.661	815.661	815.661	20391.5	0.0001	8156.61	1631.32	815.661
NV48 Seru	Serum EVs	WT	Male	6m	7117.44	1423.49	3321.47	2609.73	948.992	1423.49	3321.47	237.248	12574.1	1897.98	1423.49	1897.98	237.248	237.248	2846.97	474.496	22301.3	1897.98	6168.45	948.992	948.992

Sample nai	Sample typ	Mutation	Sex	Timepoint	mmu-miR-	mmu-miR-	mmu-miR-	mmu-miR-	mmu-miR-	mmu-miR-	mmu-miR-	mmu-miR-	mmu-miR-	mmu-miR-	mmu-miR-	mmu-miR-	mmu-miR-	mmu-miR-	mmu-miR-	mmu-miR-	mmu-miR-	mmu-miR-	mmu-miR-	mmu-miR-	mmu-miR-
NV9 Serum	Serum EVs	Q331K	Female	3m	5720.23	0.0001	5720.23	2600.1	1560.06	520.021	1560.06	9880.4	6760.27	11960.5	7280.29	10400.4	520.021	62402.5	56682.3	520.021	66042.6	0.0001	520.021	520.021	4160.17
NV11 Seru	Serum EVs	Q331K	Female	3m	2542.37	0.0001	4237.29	1694.92	847.458	0.0001	847.458	14406.8	11016.9	21186.4	14406.8	4237.29	1694.92	48305.1	38135.6	847.458	23728.8	0.0001	3389.83	1694.92	5932.2
NV12 Seru	Serum EVs	Q331K	Male	3m	919.118	919.118	3676.47	919.118	919.118	919.118	1838.24	22058.8	9191.18	11948.5	5514.71	0.0001	0.0001	22977.8	20968.9	919.118	39522.1	4595.59	2757.35	0.0001	919.118
NV13 Seru	Serum EVs	Q331K	Male	3m	905.251	301.75	7242.01	301.75	1207	2715.75	1508.75	10863	6336.76	13277	5431.51	12070	905.251	175619	167072	301.75	21122.5	301.75	301.75	905.251	3621
NV14 Seru	Serum EVs	Q331K	Male	3m	10998.3	4230.12	0.0001	846.024	1692.05	0.0001	2538.07	40609.1	17766.5	15228.4	15228.4	4230.12	0.0001	29610.8	29610.5	0.0001	3384.09	8460.24	12690.4	1692.05	1692.05
NV15 Seru	Serum EVs	WT	Female	3m	1429.39	0.0001	19153.8	2001.14	1715.27	285.878	5431.68	6003.43	4002.29	9433.96	10005.7	7718.7	1715.27	61749.6	68610.6	285.878	12578.6	0.0001	0.0001	1143.51	5431.68
NV16 Seru	Serum EVs	WT	Female	3m	1954.22	558.347	10887.8	1675.04	3350.08	837.521	5025.13	7537.69	5583.47	10329.4	5304.3	6141.82	837.521	66443.3	63093.2	1395.87	26242.3	0.0001	279.174	279.174	3908.43
NV25 Seru	Serum EVs	WT	Female	3m	2219.76	1109.88	4439.51	1109.88	1109.88	2219.76	13318.5	23307.4	17758	21087.7	17758	0.0001	1109.88	31076.6	35516.1	3329.63	48834.6	1109.88	1109.88	1109.88	6659.27
NV26 Seru	Serum EVs	WT	Male	3m	1305.36	108.78	7179.45	1414.14	435.119	1958.03	8919.93	2719.49	5221.42	8484.81	4133.63	8811.15	435.119	26977.3	27521.2	0.0001	22408.6	217.559	217.559	761.457	3154.61
NV27 Seru	Serum EVs	WT	Male	3m	0.0001	661.376	1984.13	3306.88	3968.25	661.376	1322.75	14550.3	2645.5	25132.3	9920.64	2645.5	0.0001	19391.8	11243.4	1984.13	30423.3	1984.13	1984.13	0.0001	0.0001
NV28 Seru	Serum EVs	WT	Male	3m	2583.98	1076.66	3229.97	430.663	1507.32	1291.99	1937.98	12058.6	15288.5	17657.2	4521.96	1507.32	1291.99	11627.9	14211.9	1076.66	14642.5	430.663	215.332	215.332	5167.96
NV29 Seru	Serum EVs	Q331K	Female	6m	813.008	0.0001	12195.1	1626.02	0.0001	813.008	4065.04	11382.1	13008.1	14634.1	5691.06	5691.06	1626.02	73170.7	79674.8	0.0001	16260.2	0.0001	0.0001	3252.03	4065.04
NV30 Seru	Serum EVs	Q331K	Female	6m	665.779	665.779	0.0001	665.779	0.0001	1331.56	1331.56	9986.68	9986.68	15978.7	4660.45	8655.13	0.0001	145140	146019	1331.56	13315.6	1331.56	0.0001	1331.56	4660.45
NV31 Seru	Serum EVs	Q331K	Female	6m	2378.12	1981.77	7530.72	1585.41	1585.41	792.707	2774.47	4359.89	6341.66	11890.6	10701.5	25366.6	396.354	50733.3	59056.7	792.707	15457.8	1189.06	396.354	396.354	792.707
NV32 Seru	Serum EVs	Q331K	Male	6m	1668.06	834.028	3336.11	834.028	0.0001	834.028	834.028	9591.33	6672.23	12510.4	6255.21	48373.6	417.014	50041.7	49242.1	834.028	11259.4	417.014	2085.07	1251.04	5421.18
NV41 Seru	Serum EVs	Q331K	Male	6m	7124.2	949.893	11398.7	4274.52	949.893	2849.68	3799.57	11873.7	12348.6	11398.7	8074.09	7124.2	474.947	29446.6	24697.2	1424.84	22322.5	0.0001	949.893	1424.84	4274.52
NV42 Seru	Serum EVs	Q331K	Male	6m	0.0001	0.0001	0.0001	0.0001	0.0001	0.0001	0.0001	0.0001	0.0001	0.0001	0.0001	0.0001	0.0001	364.056	9101.64	58823.5	0.0001	39215.7	98039.2	0.0001	0.0001
NV43 Seru	Serum EVs	WT	Female	6m	3265.31	0.0001	3265.31	1632.65	1632.65	0.0001	3265.31	13061.2	14693.9	9795.92	8163.27	6530.61	1632.65	65305.8	60347.5	0.0001	31020.4	0.0001	0.0001	0.0001	8163.27
NV44 Seru	Serum EVs	WT	Female	6m	3861	0.0001	9009.01	1287	0.0001	2574	1287	5148.01	11583	14157	6435.01	9009.01	2574	34749	42471	0.0001	41184	0.0001	2574	0.0001	3861
NV45 Seru	Serum EVs	WT	Female	6m	17301	0.0001	8650.52	0.0001	3460.21	0.0001	0.0001	13840.8	8650.52	5190.31	5190.31	1730.1	0.0001	25951.5	8650.52	1730.1	230104	0.0001	0.0001	0.0001	12110.7
NV46 Seru	Serum EVs	WT	Male	6m	0.0001	166667	0.0001	0.0001	0.0001	0.0001	0.0001	0.0001	0.0001	0.0001	0.0001	0.0001	0.0001	18234.3	0.0001	0.0001	0.0001	0.0001	0.0001	0.0001	0.0001
NV47 Seru	Serum EVs	WT	Male	6m	815.661	0.0001	6525.29	1631.32	815.661	0.0001	3262.64	8972.27	9787.93	17128.9	7340.95	7340.95	815.661	20391.5	20391.5	1631.32	39967.4	815.661	815.661	815.661	1631.32
NV48 Seru	Serum EVs	WT	Male	6m	3558.72	237.248	4270.46	948.992	474.496	237.248	1186.24	8778.17	12811.4	7354.69	3558.72	2135.23	474.496	3321.46	4270.46	948.992	12574.1	711.744	237.248	1186.24	3321.47

Sample nar	Sample typ	Mutation	Sex	Timepoint	mmu-miR-	mmu-miR-	mmu-miR-	mmu-miR-	mmu-miR-99b-5p
NV9 Serum	Serum EVs	Q331K	Female	3m	520.021	1040.04	8840.35	520.021	520.021
NV11 Seru	Serum EVs	Q331K	Female	3m	847.458	0.0001	8474.58	847.458	0.0001
NV12 Seru	Serum EVs	Q331K	Male	3m	919.118	1838.24	1838.24	919.118	0.0001
NV13 Seru	Serum EVs	Q331K	Male	3m	603.501	1508.75	4224.5	1810.5	1207
NV14 Seru	Serum EVs	Q331K	Male	3m	0.0001	0.0001	0.0001	3384.09	846.024
NV15 Seru	Serum EVs	WT	Female	3m	857.633	1715.27	9433.96	1715.27	285.878
NV16 Seru	Serum EVs	WT	Female	3m	279.174	1395.87	10329.4	2791.74	2512.56
NV25 Seru	Serum EVs	WT	Female	3m	2219.76	0.0001	3329.63	2219.76	0.0001
NV26 Seru	Serum EVs	WT	Male	3m	870.237	979.017	5003.86	2828.27	870.237
NV27 Seru	Serum EVs	WT	Male	3m	0.0001	1984.13	3306.88	2645.5	0.0001
NV28 Seru	Serum EVs	WT	Male	3m	861.327	1076.66	4306.63	4737.3	645.995
NV29 Seru	Serum EVs	Q331K	Female	6m	1626.02	1626.02	6504.07	2439.02	0.0001
NV30 Seru	Serum EVs	Q331K	Female	6m	1331.56	2663.12	13981.4	0.0001	1997.34
NV31 Seru	Serum EVs	Q331K	Female	6m	1585.41	396.354	7134.36	2378.12	792.707
NV32 Seru	Serum EVs	Q331K	Male	6m	2085.07	0.0001	8340.28	3336.11	834.028
NV41 Seru	Serum EVs	Q331K	Male	6m	0.0001	1424.84	4749.47	4274.52	1424.84
NV42 Seru	Serum EVs	Q331K	Male	6m	0.0001	0.0001	0.0001	0.0001	0.0001
NV43 Seru	Serum EVs	WT	Female	6m	1632.65	3265.31	13061.2	0.0001	3265.31
NV44 Seru	Serum EVs	WT	Female	6m	1287	0.0001	15444	1287	0.0001
NV45 Seru	Serum EVs	WT	Female	6m	1730.1	0.0001	5190.31	0.0001	0.0001
NV46 Seru	Serum EVs	WT	Male	6m	0.0001	0.0001	0.0001	0.0001	0.0001
NV47 Seru	Serum EVs	WT	Male	6m	815.661	0.0001	5709.63	3262.64	815.661
NV48 Seru	Serum EVs	WT	Male	6m	1423.49	711.744	2372.48	14946.6	2135.23
