# Supplementary material for: A Panel of miRNA Biomarkers Common to Serum and Brain-Derived Extracellular Vesicles Identified in Mouse Model of Amyotrophic Lateral Sclerosis
Source: Mol Neurobiol. 2024 Jan 22;61(8):5901–15. doi: 10.1007/s12035-023-03857-z (PMC11249427; doi:10.1007/s12035-023-03857-z)
Supplement: Supplementary file 5 — Supplementary file5 (PDF 414 KB) [file 12035_2023_3857_MOESM5_ESM.pdf]

| **miR-122-5p** | **miR-486a-5p** | **miR-122-5p** | **miR-486a-5p** | **miR-122-5p** | **miR-486a-5p** | **miR-122-5p** | **miR-486a-5p** | **miR-122-5p** | **miR-486a-5p** |
|---|---|---|---|---|---|---|---|---|---|
| 5730596B20Rik | 1810022K09Rik | Dand5 | Dynll1 | Idh3a | Pdpk1 | P4ha1 | Tsc22d2 | Sox11 | |
| Acap2 | 3222401L13Rik | Dennd2c | Elavl2 | Igf1r | Pdzrn3 | Papola | Unc5c | Sox6 | |
| Acvr1 | 4930533K18Rik | Dhfr | Elfn2 | Ihh | Phc3 | Pcdh7 | Wipf3 | Sp2 | |
| Adam10 | 8430419L09Rik | Dicer1 | Epha3 | Inhba | Phf20l1 | Pdpr | Zc3hav1 | Spef1 | |
| Adamts2 | A230050P20Rik | Dlat | Fam83g | Iqgap1 | Piga | Phf19 | Zfp281 | Spry2 | |
| Adss | Aak1 | Dlg2 | Fgf13 | Kcnq5 | Pik3r1 | Pigv | Zfp507 | Stim2 | |
| Ago1 | Abhd17b | Dlg4 | Fgf9 | Khdrbs1 | Pim1 | Pitx2 | Zfp644 | Stk24 | |
| Akap10 | Aff3 | Emx2 | Gab2 | Kif2a | Pitx3 | Pkm | Zfp827 | Stx6 | |
| Aldh18a1 | Agbl5 | Epo | Gabra1 | Kif5b | Plagl2 | Plcb3 | | Syt7 | |
| Aldoa | Aldh1a1 | Evi5l | Gabrb3 | Large | Ppp3cb | Plch1 | | Tbc1d10a | |
| Amigo1 | Ankrd13a | Exoc6b | Glis1 | Lnpep | Prrc2c | Pmepa1 | | Tbc1d10b | |
| Ank1 | Ar | Fam117b | Gm12355 | Lrch3 | Psmb5 | Ppip5k1 | | Tbr1 | |
| Ankrd13c | Arhgap44 | Fam219a | Golga3 | Lrp10 | Pten | Ppm1e | | Tfdp2 | |
| Arfip2 | Arhgap5 | Fkbp5 | Gpr153 | Maf1 | Ptpn12 | Prickle2 | | Tgif1 | |
| Atp11a | Arl1 | Foxk2 | Grhl2 | Map3k12 | Rassf3 | Ptbp2 | | Tmcc3 | |
| Atp1b1 | Armc8 | Foxo3 | Gucy1a2 | Map3k2 | Rhoq | Ptchd4 | | Tmem126a | |
| Atp6v1h | Asb4 | Foxp2 | Gxylt1 | Mapre1 | Rnf34 | Ptprb | | Tmem150c | |
| Bach2 | Atxn7l3 | Fundc2 | Gzf1 | Mars2 | Rpl37a | Rabl6 | | Tmem28 | |
| Bai2 | B3gat2 | G3bp2 | H3f3b | Mbnl1 | Rufy2 | Rad21 | | Tmem87a | |
| Bdnf | Bahcc1 | G6pc3 | Hnrnpa2b1 | Mef2d | Scn3b | Ralgapa1 | | Tns4 | |
| Bhlhe41 | Bcorl1 | Gabrb1 | Hpse2 | Meis2 | Siah1a | Ralgapa2 | | Trps1 | |
| Braf | Bivm | Galc | Idh2 | Micu3 | Skiv2l2 | Rbm47 | | Usp46 | |
| Brpf1 | Btaf1 | Galnt1 | Ipo7 | Mipol1 | Slain2 | Ric3 | | Vamp3 | |
| Brwd1 | Cabp2 | Gas2l2 | Irx5 | Mllt1 | Slc10a7 | Ror1 | | Vsig10l | |
| Cadm2 | Cadm1 | Gata4 | Itgb3bp | Mmgt1 | Slc12a5 | Senp1 | | Zbtb41 | |
| Calm3 | Ccdc117 | Gatad2b | Kctd1 | Morc4 | Slc8a1 | Serp1 | | Zfp282 | |
| Cbl | Ccdc47 | Git1 | Kdm5b | Mrrf | Smad2 | Sesn2 | | Zfp46 | |
| Ccdc6 | Ccdc66 | Gm20489 | Lmo3 | Ms4a6b | Smoc1 | Sgsh | | Zfp689 | |
| Ccdc97 | Ccnt2 | Gm608 | Mamdc2 | Ms4a6c | Snrpd1 | Sh2d1a | | Zfp827 | |
| Ccng1 | Cdh7 | Gm614 | Maml2 | Msn | Sox12 | Shisa4 | | Zscan29 | |
| Ccrn4l | Celf2 | Gm6169 | Maml3 | Mtf2 | Sp5 | Slc12a6 | | Zyx | |
| Cdc42bpb | Chd1 | Gnpda2 | Map3k7 | Nacc1 | Srsf1 | Slc15a1 | | | |
| Cdh6 | Chst3 | Gpm6b | Map7d1 | Negr1 | Srsf3 | Slc1a5 | | | |
| Cept1 | Cnksr2 | Gramd3 | Map9 | Nfat5 | Stau1 | Slc25a34 | | | |
| Ciita | Col6a6 | Grem2 | March4 | Nhsl2 | Stk35 | Slc25a35 | | | |
| Clic4 | Copg2 | Grhl2 | Mark1 | Nkx1-2 | Stk4 | Slc25a51 | | | |
| Clic5 | Cops7b | Gtf3c2 | Mettl1 | Nlgn3 | Surf2 | Slc2a3 | | | |
| Cmtr1 | Crebrf | Gys1 | Mex3a | Nod2 | Sypl | Slc39a8 | | | |
| Cpeb1 | Ctu2 | H1f0 | Mpv17l2 | Nol4l | Tanc1 | Slc41a1 | | | |
| Cs | D1Ertd622e | Haao | Naa15 | Npas3 | Tbx2 | Slc4a3 | | | |
| Csk | Dcc | Hdhd2 | Nalcn | Npepps | Tenm2 | Slc52a2 | | | |
| Csnk1g1 | Dkk2 | Hhip | Nfat5 | Nt5c1a | Tet3 | Slc7a1 | | | |
| Ctdnep1 | Dll4 | Hif3a | Nkx2-3 | Obfc1 | Timm23 | Slc9a1 | | | |
| Cux1 | Dlx3 | Hist1h3h | Nr2c2 | Ocln | Tmem115 | Slco5a1 | | | |
| Cxcr3 | Dnajc21 | Hnrnpu | Nr3c1 | Olfml1 | Tmub2 | Snn | | | |
| Daglb | Dock3 | Hp1bp3 | Pawr | Oprd1 | Tob1 | Sort1 | | | |
