# Supplementary material for: A Panel of miRNA Biomarkers Common to Serum and Brain-Derived Extracellular Vesicles Identified in Mouse Model of Amyotrophic Lateral Sclerosis
Source: Mol Neurobiol. 2024 Jan 22;61(8):5901–15. doi: 10.1007/s12035-023-03857-z (PMC11249427; doi:10.1007/s12035-023-03857-z)
Supplement: Supplementary file 6 — Supplementary file6 (PDF 438 KB) [file 12035_2023_3857_MOESM6_ESM.pdf]

Supplementary data:

Supplementary Table 1: Attribute Weighted Brain Derived Extracellular Vesicles (BDEVs) miRNA

BDEV 3 was removed as it was an outliner as determined by PCA.

Supplementary Table 2: Attribute Weighted serum EV miRNA

Serum EV 2 was removed as it was an outliner as determined by PCA.

Supplementary Table 3: Reads RPM for miRNA in Brain Derived Extracellular Vesicles (BDEVs)

Supplementary Table 4: Reads RPM for miRNA in Serum EVs

Supplementary Table 5: TargetScan of Common Significant miRNA in Brain Derived Extracellular Vesicles (BDEVs) and Serum EVs

Supplementary Table 6: Kyoto Encyclopedia of Genes and Genomes (KEGG) pathway of Significant miRNA in Brain Derived extracellular vesicles (BDEVs)

KEGG pathway	p-value	#genes	#miRNAs
Prion diseases	9.21E-15	13	7
Fatty acid biosynthesis	3.25E-08	3	4
Proteoglycans in cancer	7.54E-08	76	13
Thyroid hormone signaling pathway	2.35E-07	51	10
Renal cell carcinoma	3.29E-06	33	13
FoxO signaling pathway	4.18E-06	55	14
Glioma	6.03E-05	27	12
Hippo signaling pathway	9.52E-05	55	14
AMPK signaling pathway	0.000107	54	10
Lysine degradation	0.000152	19	6
ErbB signaling pathway	0.000152	33	9
Acute myeloid leukemia	0.000655	24	10
Insulin signaling pathway	0.000655	54	13
Hepatitis B	0.000655	50	13
Pathways in cancer	0.001541	121	13
mTOR signaling pathway	0.001681	27	11
Endocytosis	0.001814	74	11
Prostate cancer	0.001814	37	12
GnRH signaling pathway	0.002285	35	9
Ubiquitin mediated proteolysis	0.002475	55	10
Cell cycle	0.002684	43	11
MAPK signaling pathway	0.002684	79	13
Oocyte meiosis	0.003935	40	10
Long-term potentiation	0.003993	28	8
Steroid biosynthesis	0.004857	7	6
Protein processing in endoplasmic reticulum	0.004966	55	10
Colorectal cancer	0.005463	24	10

**Sphingolipid metabolism**	0.005657	19	9
**Non-small cell lung cancer**	0.007676	23	11
**Primary bile acid biosynthesis**	0.008328	5	4
**Adrenergic signaling in cardiomyocytes**	0.008328	45	9
**N-Glycan biosynthesis**	0.008861	16	6
**TGF-beta signaling pathway**	0.008861	27	13
**Chronic myeloid leukemia**	0.008861	28	13
**Dorso-ventral axis formation**	0.00992	13	10
**Regulation of actin cytoskeleton**	0.009932	67	12
**Neurotrophin signaling pathway**	0.01053	41	9
**HIF-1 signaling pathway**	0.010565	40	10
**Starch and sucrose metabolism**	0.011223	20	7
**Glycosaminoglycan biosynthesis - keratan sulfate**	0.013589	5	4
**PI3K-Akt signaling pathway**	0.016303	100	14
**Focal adhesion**	0.016408	65	10
**Axon guidance**	0.020772	43	11
**Viral carcinogenesis**	0.023323	59	14
**Estrogen signaling pathway**	0.027082	33	10
**Adherens junction**	0.03038	28	8
**Other glycan degradation**	0.032139	4	4
**p53 signaling pathway**	0.032915	23	9
**Sphingolipid signaling pathway**	0.032915	40	10
**Glycosaminoglycan biosynthesis - chondroitin sulfate / dermatan sulfate**	0.033118	7	6
**Small cell lung cancer**	0.03416	29	10
**Progesterone-mediated oocyte maturation**	0.043474	31	10
**2-Oxocarboxylic acid metabolism**	0.045544	7	4

Supplementary Table 7: Gene Ontology analysis of Significant miRNA in Brain Derived extracellular vesicles (BDEVs)

GO Category	p-value	#genes	#miRNAs
**Cell**	0	3067	20
**Intracellular**	0	2801	20
**Organelle**	1.52E-162	2405	19
**anatomical structure development**	3.43E-153	958	19
**biological_process**	7.96E-152	3911	20
**ion binding**	2.68E-82	1475	19
**molecular_function**	1.21E-77	3810	20
**cell differentiation**	1.58E-77	700	18
**embryo development**	7.54E-60	295	19
**cellular protein modification process**	2.17E-53	642	19
**Cytoplasm**	7.76E-48	2297	20
**chromosome organization**	9.11E-35	187	17
**cellular nitrogen compound metabolic process**	1.45E-34	1013	20
**biosynthetic process**	7.85E-32	897	20

**cellular_component**	4.53E-30	3708	20
**anatomical structure formation involved in morphogenesis**	2.13E-26	216	16
**cell morphogenesis**	7.73E-22	187	14
**homeostatic process**	8.65E-19	239	14
**protein complex**	2.73E-16	841	20
**cell death**	7.82E-16	241	17
**cell division**	6.72E-15	148	12
**Cytoskeleton**	3.51E-14	378	14
**cell cycle**	4.03E-13	263	16
**nuclear chromosome**	7.38E-13	77	12
**cytoskeleton organization**	1.88E-12	189	16
**catabolic process**	5.37E-12	418	16
**cell motility**	6.45E-12	159	15
**cytoplasmic membrane-bounded vesicle**	3.69E-11	144	15
**Growth**	1.73E-10	119	17
**Chromosome**	1.85E-10	174	15
**cytoskeletal protein binding**	1.41E-09	187	16
**cellular component assembly**	4.34E-07	273	17
**Endosome**	6.89E-07	172	14
**protein binding transcription factor activity**	1.70E-06	110	14
**in utero embryonic development**	4.29E-06	103	13
**nucleic acid binding transcription factor activity**	5.21E-06	207	16
**developmental maturation**	8.68E-06	42	10
**Nucleoplasm**	1.01E-05	248	17
**response to stress**	1.73E-05	453	16
**enzyme binding**	0.000121478	257	12
**endoplasmic reticulum**	0.000299717	375	15
**Vacuole**	0.000311825	77	11
**ligase activity**	0.0005797	150	14
**cofactor metabolic process**	0.005444081	57	9
**circulatory system process**	0.005610098	39	9
**small molecule metabolic process**	0.007069404	414	17
**peptidyl-threonine phosphorylation**	0.012510811	25	7
**enzyme regulator activity**	0.016359561	163	10
**Golgi apparatus**	0.016359561	332	15
**ribonucleoprotein complex assembly**	0.025437908	36	11
**nuclear envelope**	0.03794573	64	10
**vesicle-mediated transport**	0.040989097	211	13

Supplementary Table 8: Kyoto Encyclopedia of Genes and Genomes (KEGG) pathway analysis of Significant miRNA in serum extracellular vesicles (EVs)

KEGG pathway	p-value	#genes	#miRNAs
**Lysine degradation**	2.17E-05	9	2
**Porphyrin and chlorophyll metabolism**	7.64E-05	13	2

**Hippo signaling pathway**	0.000159	23	2
**Steroid biosynthesis**	0.000339	2	1
**TGF-beta signaling pathway**	0.006718	10	1
**Drug metabolism - other enzymes**	0.013213	10	2
**FoxO signaling pathway**	0.013213	18	2
**Thyroid hormone signaling pathway**	0.018594	20	2
**Drug metabolism - cytochrome P450**	0.023957	8	2
**Glycosaminoglycan degradation**	0.029001	4	1
**Sulfur relay system**	0.035561	2	2
**Pathways in cancer**	0.046221	40	2

Supplementary Table 9: Gene Ontology analysis of Significant miRNA in Serum extracellular vesicles (EVs)

GO Category	p-value	#genes	#miRNAs
**Cell**	1.88E-242	872	2
**Intracellular**	3.43E-143	812	2
**Organelle**	2.70E-43	698	2
**anatomical structure development**	4.42E-42	282	2
**biological_process**	1.57E-41	1137	2
**ion binding**	6.10E-27	446	2
**cell differentiation**	4.83E-22	208	2
**embryo development**	1.19E-19	94	2
**molecular_function**	8.34E-19	1102	2
**biosynthetic process**	1.74E-16	295	2
**Cytoplasm**	2.35E-13	671	2
**cellular protein modification process**	6.03E-13	183	2
**cellular nitrogen compound metabolic process**	1.06E-12	311	2
**chromosome organization**	3.99E-10	57	2
**cofactor metabolic process**	7.15E-09	36	2
**anatomical structure formation involved in morphogenesis**	1.53E-07	65	2
**homeostatic process**	9.34E-07	76	2
**cellular amino acid metabolic process**	9.68E-05	43	2
**Growth**	0.000122	41	2
**protoporphyrinogen IX biosynthetic process**	0.00045	7	1
**cell morphogenesis**	0.0005	50	2
**response to stress**	0.000557	151	2
**in utero embryonic development**	0.000957	38	2
**sulfur compound metabolic process**	0.001244	28	2
**cell death**	0.00184	68	2
**cytoskeleton organization**	0.004526	55	2
**cellular_component**	0.00891	1050	2
**protein complex**	0.01226	233	2
**catabolic process**	0.014422	119	2
**cytoplasmic membrane-bounded vesicle**	0.017902	41	2
**small molecule metabolic process**	0.025585	136	2

**cellular component assembly**	0.02668	83	2
**protein binding transcription factor activity**	0.0268	35	2
**cell division**	0.029894	38	2
**positive regulation of fibroblast proliferation**	0.046663	12	2
**Cytoskeleton**	0.046663	101	2

Supplementary Table 10: TargetScan of Significant miRNA in Brain Derived Extracellular Vesicles (BDEVs)

Supplementary Table 11: TargetScan of Significant miRNA in serum EVs

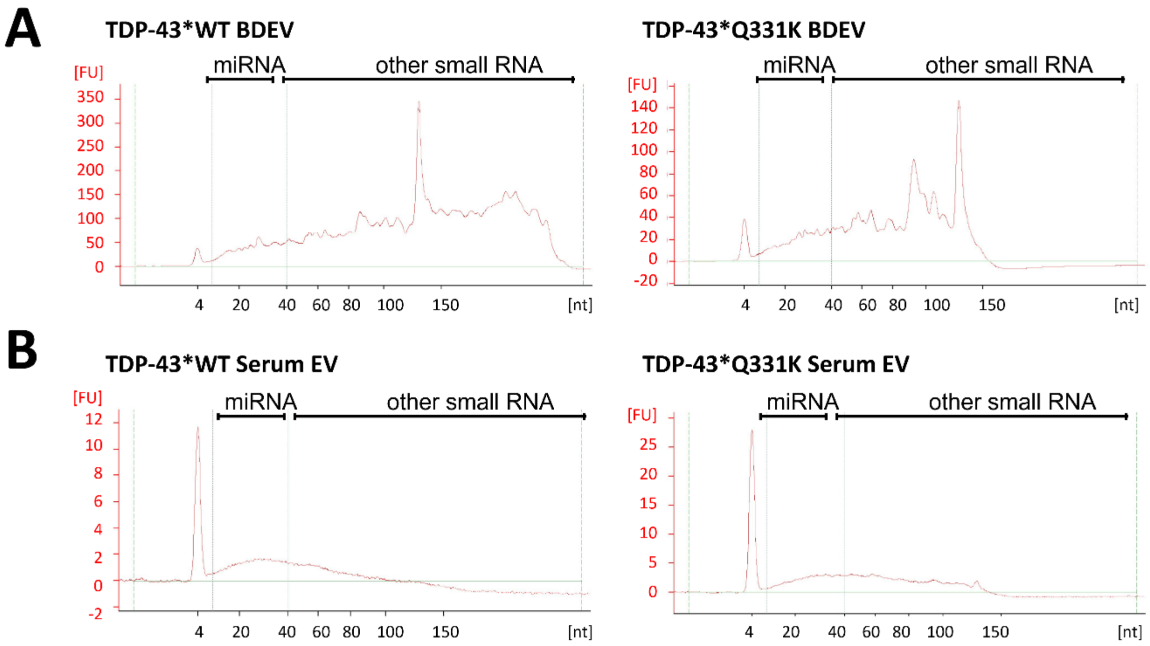

Supplementary Fig. 1: Small RNA is present in Brain Derived Extracellular Vesicles (BDEVs) and serum EVs. (A). A small RNA assay was run using the Agilent Bioanalyser on the small RNA isolated from the BDEVs and serum EVs. The initial peaks at 4 nt represent the marker. The BDEVs isolated from the TDP-43*WT and TDP-43*Q331K mice appear to contain miRNA indicated by peaks in the 10-40 nt region, in addition to other small non-coding RNAs due to peaks in the ≥40 nt region. **(B).** The serum EVs isolated from the TDP-43*WT and TDP-43*Q331K mice appear to contain miRNA indicated by peaks in the 10-40 nt region. These results are from TDP-43*WT and TDP-43*Q331K mice of n=12 (from the BDEVs), and n=12 (from the serum EVs).
