# Supplementary material for: A Panel of miRNA Biomarkers Common to Serum and Brain-Derived Extracellular Vesicles Identified in Mouse Model of Amyotrophic Lateral Sclerosis
Source: Mol Neurobiol. 2024 Jan 22;61(8):5901–15. doi: 10.1007/s12035-023-03857-z (PMC11249427; doi:10.1007/s12035-023-03857-z)
Supplement: Supplementary file 7 — Supplementary file7 (PDF 784 KB) [file 12035_2023_3857_MOESM7_ESM.pdf]

miR-199-3p	miR-29-3p	miR-486-5p	miR-361-3p	miR-122-5p	miR-425-5p	miR-136-5p	miR-96-5p	miR-142-5p	miR-19-3p	miR-141-3p	miR-770-3p	miR-335-5p	miR-200-3p	miR-194-5p	miR-183-5p	miR-341-3p	miR-182-5p	miR-370-3p
Zbtb20	Tet3	Zc3hav1	Fbxl19	Cux1	Grin2b	Cpeb2	Phf20l1	Tmem245	Atxn1	Klf12	Grin2b	Prkaa2	Zeb2	Dgkk	Cep97	Lrriq1	Htr4	Aak1
Fam199x	Adamts17	Map9	Chaf1a	Nol4l	Cenpl	Braf	Snx30	Sytl5	Rora	Fat3	Zbtb46	Gm608	Gpm6a	Fzd6	L3mbtl3	M5C1000I18Rik	Frs2	Bsn
Tmem62	Col4a5	Gucy1a2	Ppp1r3c	Grhl2	Amph	Zbtb20	Cacnb4	Zbtb20	Taok1	Peg3	Nhsl2	C77370	Uba6	Onecut2	Ago2	Kcnip4	Adcy6	Dnah17
Celsr2	Tet2	Gabrb3	Neil2	Slc25a34	Zfp449	6330408A02Rik	Sox6	Zfp616	Tnrc6b	Gmps	Gna13	Fam120c	Arhgap20	Hectd2	Lhfpl2	Mfrp	Rgs17	Tmem245
Erbb4	Col1a1	Srsf3	Kmt2d	Slco5a1	Dek	Psd3	Slc39a1	Kcnh7	Zbtb20	Fnip2	Muc5b	Psd3	Myo9a	Ppp2r3a	Mtmr6	Arpc2	Rnf152	Trp53inp1
Itga3	Sestd1	Gm12355	Zbtb16	Aldoa	Cdc5l	Gdi2	Slc1a1	Zfpm2	March2	Ikzf2	Lhx4	Trim59	Msn	Prtg	Celf2	Mblac1	Lims1	Tnrc6b
Ak4	Eln	Mex3a	St6galnac6	Vamp3	Ap3m1	Gm6377	Brpf3	Tbc1d8b	Dock10	Zyg11b	Rgs8	Hoxd12	Hipk3	Hook3	Mal2	Ptpn14	Kcnq3	Grin2b
Ptpn4	Atad2b	Nalcn	Ldoc1l	Slc12a6	Syncrip	Prdm16	Gad2	Rgs17	Zfp827	Tnrc6b	Gm2007	A330008L17Rik	Cers6	Slk	Psen2	En1	Slc5a3	Cmah
Ankrd44	Ifi30	Col6a6	Shank1	Slc7a1	Kcnb2	Ptchd4	D3Bwg0562e	POU2F1	Foxp2	Kbtbd4	Gm4724	Rbmx	Ptpn14	Tmem161b	Gjd2	Galnt16	Snx30	Syne2
Fktn	Col3a1	Zfp281	Lrcol1	Nlgn3	Bcor	Gm6871	Rev1	Skp2	Tub	Zbtb34	Gm14434	Glyr1	Zfp711	Slc10a7	Unc13b	Zfp937	Cacnb4	Gpr26
D030056L22Rik	Adamts12	Ccdc117	Vamp1	Sh2d1a	Larp4b	Ppp2r2a	Sh3pxd2b	Necab1	Kcnj2	Zbtb20	Gm11007	Gzf1	Tmem245	Rap2b	Cacna1e	Acap2	Palld	Gm6710
St6gal2	Clock	Nr2c2	Rab11fip4	P4ha1	Brwd3	Hipk2	Nova2	Ptpn2	Prickle2	Zeb2	Gm6710	Sp1	Rab11fip2	Snap91	Mbnl1	Dlec1	Rapgef5	Gm4724
Rfx3	Has3	Prrc2c	Tmem28	Gpm6b	Rufy2	Hs6st3	Frs2	Uba3	Rnf152	Ppm1e	Gm14419	Dock4	Esrrg	Cbln4	Klhl28	Tbx1	Fat3	Gm11007
Gna12	Per3	Pik3r1	Mks1	Lnpep	Ndnf	Frrs1	Tbr1	Fam199x	Trak2	Plag1	Ascc2	Ubl4	Zeb1	Sv2c	Foxn2	S1pr3	Gad2	Gm14434
Cd2ap	Grin2b	Wipf3	Trim56	Grem2	Pomt2	Smg1	C77370	Brwd3	Dtna	Zeb1	Plekhh1	Klhl15	Dlc1	Rab11fip2	Epdr1	Fat1	Foxn3	Gm2007
Klhl3	Adamts2	Pdpk1	Agtr2	Gm608	Lpp	Cntn5	Rab3c	Cacna1e	Crebl2	Sytl5	Slc7a1	Zfp286	Hook3	Tmed5	Zeb1	Nebl	Cacna2d1	Hdac4
Ankrd13c	Zdhhc21	Cdh7	Gltpd1	Slc41a1	Gpr26	Antxr2	Celf2	Samd12	Lpp	Atad2b	Zfp704	Rbm7	C030046E11Rik	Ntng1	Rora	Csrnp3	Prrg3	Lpp
Abca13	Gpr156	Ar	9330159N05Rik	Slc25a51	Pde3a	Ar	Chst10	Nfat5	Spire1	Kcng3	Klhdc7a	Hand1	Phc3	Zbtb10	Akap7	Med12l	Arf4	Gm14419
Cd151	Zbtb20	Gabra1	Mex3a	Ralgapa2	Il1rapl1	Tmx4	Klhl7	Ptpn4	Itgb8	Sass6	Lrrc75b	Tmem184c	Qk	Bicd2	Ttc14	Ache	Satb2	Ep400
Scd4	Cep97	Armc8	Fam195b	Ralgapa1	Hipk3	Gdf7	Hook3	Cadm2	Zmynd11	Zfp281	Bhlha15	Clock	Ago2	Nfat5	Atp13a3	Dennd3	Cttn	Gm14305
Alx4	Icos	Arhgap5	Ly6d	Ciita	Pdcd10	Plcxd3	Sox5	Slc5a3	Rab14	Slc16a7	Pgbd5	9030624G23Rik	Slc25a30	Trim23	Vangl1	Pvrl1	Nfat5	Mlec
Itgb8	Cd46	Asb4	Atp5e	Gm20489	Tmem170b	Lnpep	Tmem170b	Ppip5k2	Sox6	Slc5a3	Tacr3	Onecut2	Zbtb20	Setd8	Atf2	Nova2	Clock	Csmd2
Cpeb4	Col6a3	Celf2	Ppp2r4	Ppip5k1	Serp1	Abcb1a	Gng12	Prkaa2	Cep350	Lysmd3	AI118078	Csnk1g1	Mmd	Sgpp2	Arhgap6	Zcchc5	Parm1	Dpp10
Purg	Col11a1	Lmo3	Nkx3-1	Zfp282	Wapal	Irak2	Prdm16	Robo1	Slc24a3	Ptprg	Cabp7	Secisbp2l	Mdm4	Zbtb20	Ptpn4	Ube2g2	Lhx1	Hook3
Lrp2	Col2a1	Dock3	Serpinb3b	Spef1	Dr1	Rap2c	Etv3	Slitrk4	Klhl42	Slc30a1	Ptprt	Pygo1	Tenm1	Fam107b	Rbms1	Phf12	Igsf3	Fam114a2
Cnot7	Wisp1	Gab2	Ltbr	Pdpr	Dnajc27	Nfib	Lmtk3	Hipk1	Rapgef2	Wdfy3	Apbb2	Shisa6	Atp11c	Ptplb	Erbb2ip	Prdm11	Nid1	Sik2
Gja3	Ammecr1l	Grhl2	Sdr9c7	Phf19	Ank3	Trpc4ap	Slc6a9	Zc3h12c	Dsel	Sestd1	Zbtb20	Fat3	Lrp1b	Mex3a	Enah	Pax2	Pdk4	Syne1
Taok1	Nfia	8430419L09Rik	Mvd	Bach2	Rgs8	Rbm33	Tcf7l2	Acadsb	Ppara	Strn	Gm14305	Kcnj4	Nek10	Rasgrf2	Slc22a23	Usp17ld	Camsap2	Slc24a2
Lin28b	Nsd1	Dlx3	Tmem132b	Zfp827	Zfp148	Srl	Adcy6	Pura	Kbtbd8	Arl5b	Mpv17l	Whsc1l1	Sec23a	Osbpl8	Irs1	Apold1	Bcl2l13	Ino80
Adamtsl3	Amer1	Tob1	Gm53	Slc2a3	Arl5b	Sept3	Cnnm3	Lmx1a	Elmod2	Etnk1	Prx	Dnah3	Zcchc24	Ssh2	Dmxl1	D17Wsu92e	Cadm2	Rnft2
Arel1	Tet1	Ankrd13a	Igf2	Kcnq5	Chp2	Tanc2	Med1	Unc80	Mpped2	Tmem56	Gipc3	Numa1	Styx	Ube2d3	Nr3c1	Tapt1	Olfr976	Zbtb20
Vldlr	Nav3	Piga	Slamf9	Foxo3	Adra1a	Bmpr2	Sort1	Kcnq3	Ralgps1	Cds2	Map2	Chfr	Kdr	Eif4ebp2	Rcn2	Zcchc14	Usp13	Rgp1
Itga6	Brwd3	Agbl5	Gm10684	Arfip2	Neto1	Sema4c	Vamp3	Tapt1	Lonrf1	Lmln	Smoc1	2510009E07Rik	A1cf	Lingo2	Frmd6	Whsc1l1	Nptx1	Tacr1
Plekhh1	Nrep	Crebrf	F830016B08Rik	Gm6169	Dip2c	BC005537	Specc1	Med14	Arrdc3	Atxn1	Gdf7	Rprm	Lhfp	Hbegf	Hectd2	Fzd1	Lmtk2	Nfat5
Kdm5a	Zbtb34	Bivm	Olfr550	Zscan29	Foxi1	Tmem178b	Sowahc	Tdo2	Rapgef6	Akap2	Plekhg1	Rnf141	Wasf3	Brms1l	Tcf4	Fam196b	Rasa1	Klf12
Wdr76	Tdg	Tsc22d2	C1qa	Nfat5	Herc2	Pdc	Cdh7	Igf2bp3	Acsl4	Palm2Akap2	Mecp2	Prss46	Marcks	Gpr101	Lck	A3galt2	Gxylt1	Kmt2d
Acvr1c	Smtnl2	Aff3	Spesp1	Adamts2	Gm20521	Kctd16	Tes	Epas1	Kcnb2	Prtg	Etv3	Gm17590	Zfyve20	Igf1r	Bnip3l	Slc14a1	Dab2ip	Tfcp2l1
Ppp2r5e	Zfp36l1	Pdzrn3	Fbxl18	Micu3	Pabpn1	Galnt1	Pnpla8	Bai3	Ivns1abp	Abl2	Ksr2	Cln8	Lcor	Fbxw7	Cep170b	Tex2	Zbtb20	Tcf23
Dio2	Col4a3	Scn3b	Ccl22	Slc1a5	Zfp423	Plekha8	Ppm1f	Chd9	Slc8a1	Bclaf1	Zfp11	Cep350	Crtap	Xpnpep3	Btg1	Ficd	Atad2	Magi3
Reps2	Kctd20	Epha3	Myo19	Ror1	Ptp4a2	Bptf	Zeb1	Nck2	9430020K01Rik	Atxn7	Nt5c1a	Prdm11	Slc5a3	Setd5	Samd4	Fxyd6	Aak1	Tchh
Nova1	Col7a1	Phf20l1	Pcx	Csnk1g1	Zfp831	Yes1	Prtg	Phip	Clock	Rgag1	Cacna1e	Nckap5	Rapgef2	Qk	Ccdc50	Gm9913	Sox5	Tmem151b
Tppp	Col9a1	Copg2	Fam171a2	Ankrd13c	Fam174a	Zbtb33	Map2k1	Dgke	Map3k2	Plcxd3	4930402H24Rik	Il7	Zfp281	Ache	Mef2c	Atl1	Gnaq	Taok1
Ccnt1	Bach2	Pten	Wdr54	Acap2	Neurod6	Pcdh19	Lrrc3	Dio2	AW549877	Pcdh9	Fibcd1	Daam1	Dennd1b	Rcor1	Ppp2ca	Mier2	Mtss1	Syn3
Rapgef5	Olfr701	Tenm2	Shisa5	Ptprb	Lcor	Cnot7	St6galnac3	Qk	Tmem65	Pappa	Syne2	Kdm4c	Adamts3	Dmd	Mapk8ip1	Htr5a	Mitf	Map3k13
G3bp2	Klhl28	Ccdc66	Cyp2g1	Khdrbs1	Serpine1	Abhd16b	Fut9	Pou2f1	Med12l	Kmt2a	Surf6	Map3k2	Aak1	Dstyk	Mfsd6	Mcu	Aldh6a1	Fbxl18
Atad1	Glis2	Gxylt1	C1ql4	Nhsl2	Crebzf	Cacul1	Frem1	Cnot6	Neurod1	Dstyk	Sorcs2	Rab3c	Spryd7	Nbr1	Baz1b	Rabif	Pcnx	1110002E22Rik
Lpar4	Adamts9	Sp5	Hrk	Atp11a	Rcor1	Klhl15	Hbegf	Vmp1	Chic1	Zdbf2	Mcc	Ehd1	Cdh20	Pvrl4	Plekha5	Paqr3	Zcchc14	Cep250
Mdga2	C77370	Slc10a7	Zfp772	Ptchd4	Cpeb2	Extl3	Mitf	F2rl1	Lin9	Myt1l	Znf512b	Gpr63	Zfpm2	Stox2	Srek1ip1	Prrc1	Sp3	Synm
Kctd16	P2ry4	Tet3	A530016L24Rik	Cdh6	Arhgap21	Stx17	Ccdc50	Hltf	Skil	Dock10	Brsk2	Sephs1	Synj1	Ubr3	Hnrnpa1	Gm10545	Ago1	Ddi2
Rb1	Trim71	4930533K18Rik	Scn4b	Ago1	Polh	Gprin3	Opcml	Ncapg2	Adrb1	Mbnl1	Cplx2	Lancl3	Nfib	Ubn2	Prkci	Abhd8	Ppm1l	Pdpr
Pnrc1	Nav2	Mamdc2	Dpf1	Gys1	Vezf1	Creb3l2	Xkr4	Reps2	Csmd1	Trhde	Zfp764	Sorcs1	Egln1	Tacr3	Lnpep	Olfml3	Ptprg	Glis3
Nlk	Eif4e2	Mark1	Kcnab3	Galc	S1pr1	Prlr	Zfp704	Prkg1	Acadsb	Lpar1	Foxi1	Alpk3	Calcrl	Kmt2c	Arhgef37	Nxnl1	Gmps	Atf7
Bcar3	Amot	Aak1	Crhr1	Meis2	Zfhx4	Cdv3	Zfp449	Slc23a2	Pmepa1	Lpp	Bsn	Hoxd8	Clasp1	Arid2	Crk	Zfp239	Asxl3	Kcnc1
Fgf6	Sh3pxd2a	Rhoq	H2afb1	5730596B20Rik	Fgf10	Zfp827	Unc13c	Kif5c	Tnks	Tmed5	Tbc1d24	Mpc1	Ncoa2	8030462N17Rik	Tpm3-rs7	Prss32	Nrn1	Atxn1
Kdm6a	3110057O12Rik	Snrpd1	Fis1	Tbc1d10a	Pou2f2	Gpr26	Gpc3	Nhsl2	Pak6	Slc7a11	Tmco4	Acot11	Ywhag	Slc12a6	Chd2	Edar	L1cam	Rapgef1
Celf1	Rora	Pitx3	Clec14a	Zyx	Dyrk1a	Fam173b	Pgap1	Trip11	Nbeal2	Sept11	Tshz2	Adamts12	Pgap1	Ube2v2	Arhgap12	Hyal1	Slc7a14	Smcr8
Nid2	Cd276	Map3k7	Gm5382	Nt5c1a	Arih1	Mtpn	Tns3	Gdnf	Mycl	Dock4	Hip1	Asph	Ube3a	Rabac1	Npc2	Tie1	Slc1a2	Rnf150
Ccdc85c	4931406P16Rik	Skiv2l2	6330403A02Rik	Npas3	Phip	Map3k1	Epha3	Fgf4	Adcy1	Qk	Sh3pxd2b	Rab11b	Strn	Myrf	Tmpo	Slc10a4	Braf	Smurf1
Tab3	Adam12	Slain2	Gm14484	Pkm	Rnf145	Mdm1	Ntn4	Fbxl3	Tfcp2l1	Rgs17	Emp2	Srek1ip1	Fhl1	Tor1b	Fcho2	Tnr	Tns3	Fam73b
Ppp2r2a	Slc19a3	Arl1	Gm14483	Akap10	Fst	Pura	Ankrd27	Slc24a2	Lrp2	Cacna1e	Rnf169	Sec14l5	Slc24a4	Itpkb	Ttc7b	6330408A02Rik	Ddi2	Zbtb42
Lox	Nav1	Slc12a5	Serpinb3a	Tmem28	Cpeb1	Rpusd4	Htr1b	Lrp1b	Phtf2	Dusp3	Kcna2	Fmn1	Slc6a1	Lrrfip1	Tmed7	Hadhb	Fut9	Kcnq2
Mysm1	Hrk	Phc3	Rnd1	Obfc1	Pak2	Tgfb2	Foxo1	Pank3	Psd3	P2ry1	Arhgef37	Tgoln1	Fbxo33	Acbd3	Lmo3	Tmem50b	Tacc1	Ank3
Cep85l	Ccnt2	Nfat5	Gm14474	Tmcc3	Ybx3	Zfp472	2410016O06Rik	Ube2d1	Ppp2r5e	Dr1	Nt5dc3	Stard7	Sesn3	Tcf24	Pdcd4	Gna14	Fnbp1l	Peg3
Golga4	Col4a1	Abhd17b	Gm14478	Sox11	Rhot1	Zfp449	Gpr124	Hoxd8	Zfp831	Osbpl6	Asic2	Ppara	Pkd1	3830406C13Rik	Lrp6	Slc30a9	Sept9	Ntrk3
Sema3a	Klf12	Plagl2	Gm14476	Ctdnep1	Mbnl1	Ncs1	Tmem198	Cpeb2	Car8	Bend3	Pla2r1	Fasn	Npnt	Mid1ip1	Erich5	Sox11	Htr1a	Ppfibp1
Slc39a10	Tll1	Hpse2	Gm14482	Evi5l	Rsf1	Glipr2	Dock1	Chm	Impdh1	Gna13	Dnaja4	Vangl2	Slc6a11	Dach1	Grem2	Mycl	Med1	Sox6
Dtna	St8sia6	Chst3	Lrrc15	Pmepa1	2510009E07Rik	Gdf6	Slitrk2	Zfp275	Usp32	Kcnh7	Stac3	FMN1	Twistnb	Rab2b	Plag1	Tpm3-rs7	Mak	Cacna1e
Fgf7	Col19a1	Unc5c	Synpo	Atp1b1	Dis3l2	Dis3	Nhlrc3	Adamts1	Tab3	Cers6	Plec	Kat7	Mblac2	Bckdha	Dgcr2	Smim14	Slmo2	Hist2h4
Scd2	Smim17	Arhgap44	Pla2g12b	Zbtb41	Mbd5	Ppargc1a	Sh3kbp1	Rasef	Coq10b	Nr3c1	Zzef1	Arhgap18	Immp2l	Serp1	Adcy6	Pitx2	Mob1b	Xpo4
Fut9	Dot1l	Nr3c1	Stau2	Brpf1	Grm1	Pcdh1	Rapgef4	Prpf4b	Dpysl5	Kcnc2	Zfp672	Crim1	Msl2	Nrp1	Ppapdc1b	Hist2h2bb	Kmt2a	Gpr124
Acvr2a	Ddx3x	Bahcc1	Dapk3	Tbc1d10b	Maml1	Zfp92	B4galnt1	Abca1	Gpr137c	Foxn3	Sept3	Fam107b	Apoo	Chd6	Hoxd3	Parp10	Exoc4	Rasal2
Slc7a5	Fbn1	Elavl2	E2f2	Cpeb1	Ptbp2	Mrps16	Slc12a6	Map3k13	Igf2r	Gbp7	Rspo4	Il17rd	Vkorc1l1	Rsbn1l	Ctdspl	A930004D18Rik	Fam171a1	Gpatch2
Map3k5	Dusp2	B3gat2	Suv420h2	Foxp2	Sh3rf1	Man1c1	Pard3b	Kif13a	Erbb4	Dmwd	Glt6d1	Zfp113	Gjc1	Pnpla8	Clcn3	Mgat5	Ube2h	Cdnf
Enox2	Gpr165	Timm23	Bcl11b	Exoc6b	Sdk2	Gm15737	Ankib1	Neto2	Fras1	Cdk19	Cacng4	Sirt4	Dennd5a	Cks1brt	Epha4	Tsr2	Samd12	Slc7a1
Katnbl1	Stard8	Cops7b	Gjb3	Snn	Hnrnpab	Fzd5	Zcchc3	Vps54	Ccnd2	Ralgps1	Triobp	Lrrc8c	Zbtb38	Wapal	Rnf138	4732440D04Rik	Rac1	Fmn1
Arhgap20	Eml6	Smad2	Gstm2	Map3k2	Rab2b	Cntrob	Cacnb1	Lrp2	Ubl3	Tanc2	Gm7120	Lysmd4	Zfp292	Hrh1	Ccl20	Camk2b	Sv2c	Ints2
Adamts3	Pi15	Cnksr2	E130208F15Rik	Gnpda2	Efnb2	Samhd1	Ralgps1	Slc4a4	Smurf1	Hook1	AA474408	Slc45a4	Zfp217	Cdk14	Lrp1b	Egln1	Bnc2	Gab2
Plcb1	Otud4	Cabp2	Myd88	Fundc2	Ubr5	Sgpl1	Prkce	Pcgf3	Kcna4	Tiam1	Kcnj10	F13a1	Celf1	Clcn5	Zfyve26	Dnajc16	Vamp3	Tet3

miR-199-3p	miR-29-3p	miR-486-5p	miR-361-3p	miR-122-5p	miR-425-5p	miR-136-5p	miR-96-5p	miR-142-5p	miR-19-3p	miR-141-3p	miR-770-3p	miR-335-5p	miR-200-3p	miR-194-5p	miR-183-5p	miR-341-3p	miR-182-5p	miR-370-3p
Abca1	Otub2	Btaf1	Ybey	Ocln	Acvr1c	Exph5	Plekhm1	Rbm24	Braf	Slk	Hivep3	Zmpste24	Zfp131	Sned1	Map3k13	Ldlrap1	Gpr158	Scube3
Plag1	Lysmd1	Dcc	Clic4	Inhba	Tmtc2	Cbx4	Gan	Kras	Gnaq	Pgrmc2	Lmf2	Zfp654	Cnep1r1	Cxcl1	Map3k2	Fam172a	Ralgps1	Arid1b
Cela1	Znf512b	Glis1	1700052N19Rik	Gm614	Ppp2r2a	Uggt1	Zfp36l1	Dtl	Tnfaip3	Irs2	Fam53b	Ube2g1	Cecr2	Zfhx4	Mxd4	Gabpb1	Mkl2	Foxk1
Rapgef4	Ptp4a1	Maml2	Tmem52b	Aldh18a1	Zbtb34	Esrrg	Klhl8	Lrat	Gatsl2	Tmtc1	Ncapg2	Pcnx	Gucy1a3	Taf4a	Ajap1	Zfp106	Msn	Eif4ebp2
Znrf2	Nlgn3	Kdm5b	Pfn2	Clic4	Ube2g1	Wasf3	Dgkk	Vangl1	Rap2c	Zfand5	Ttc9c	Nrxn1	Vash2	Thsd4	Srsf2	Dcp1b	Zfp697	Nt5c1a
Nedd4	Rpl5	Slc8a1	Abcc10	Fam117b	Cacna1d	Ankrd11	St8sia3	Pip5k1b	Ubn2	Ulk2	Morc4	Rasa1	Rusc2	Tead1	Podxl	Ifitm10	Epas1	Cux1
Ikzf4	Gm9726	Gpr153	Gm5132	Mllt1	Rhno1	Tet3	Cd164	Rsf1	Vamp3	Dip2b	Ksr1	Snip1	Foxp1	Sumo2	Atrn	Mef2c	Wisp1	Hipk2
Lcor	Ankrd52	Ppp3cb	Prrt2	Zfp46	Dach1	St8sia2	Epb4.1l3	Zfr	Zbtb10	Zbtb16	Megf6	Usp6nl	Crkl	Krit1	Tub	Sbds	Acvr1c	2010111I01Rik
Eml6	Grip1	Cadm1	Ptcd1	Rabl6	Slc16a1	2510003E04Rik	Pura	Otud4	Suz12	Brms1l	Rimkla	Zdhhc8	Peak1	Ctdspl2	L2hgdh	Xkr9	Zfand4	Slc5a3
Nxph1	Pxdn	Srsf1	Tmem167b	Igf1r	Clcn1	Tmem233	Atxn1	Sox5	Mllt4	Fam189a1	Kremen1	Hnrnpr	Cfl2	Epc2	Sacs	Abhd13	Col25a1	Klf13
Wdr47	Islr2	Dll4	Chmp7	Dennd2c	Dhx40	Socs4	Gria1	Pds5b	Slc4a7	Fgfr1op	Gja5	Etf1	Rspry1	Klhl11	Bach2	Fbxo7	Zfp36l1	Pnpla3
Pura	Slc16a14	Gzf1	Ajuba	Hif3a	Arhgap9	Cntn1	Ccdc79	Luzp2	8030462N17Rik	Arrb1	Podxl	Nabp1	Syncrip	Daam1	Zmym2	Gins2	Dyrk2	Rab43
Tmtc3	Col15a1	Naa15	Kcnj4	Cxcr3	Gria3	Lzts3	Zhx2	C77370	Synpo2	Adipor2	Gpr35	Ccnt2	Nfia	Malt1	Osbpl8	Ncbp1	Dnajc30	Sp4
Met	Trib2	Stk4	Vgll4	Kif2a	Clrn1	Purb	Zcchc11	Spopl	Wdfy3	Crlf3	Phc3	Phf20l1	Rps6kb1	Tmem109	Phf6	Stk24	Wdr76	Cyth1
Tubgcp3	Spns1	Sypl	Cebpa	Cadm2	Spats2l	Slc7a3	Msn	Trp53inp1	Atf2	Gpm6b	Syn3	Efnb1	Chsy1	Lgi1	Pam	Armc4	Tenm4	Reps2
Mvb12b	Pan2	Itgb3bp	1700012L04Rik	Lrch3	Mef2c	Srsf1	Grhl2	Synj1	Elovl5	Map3k2	Plxna4	Sept11	Clic4	Ddx6	Gng5	Zfp799	Cyb5b	Plxna1
Mecp2	Tfeb	Stk35	Spdyb	Ppm1e	Cygb	Rpgrip1l	Celsr1	Med28	Zfp654	Slc25a36	Iqsec1	Mtdh	Inhba	Dbf4	Taok1	Arsi	Stox2	Acox3
Map3k4	Dpysl5	Pawr	Ccpg1os	Prickle2	Cab39	Ino80	Cog5	Dmd	Ptchd4	Gabrg3	Gm15800	Lcor	Phf21b	Grhl2	Trim2	Tmem179	Mgat4c	Glg1
Dcbld2	Dio2	Ccdc47	Gm14501	Cs	Zbtb43	Tnpo1	Parm1	Kitl	G3bp2	Astn1	Slc36a1	Ppp6c	BC030336	Enah	Nrg1	1700084C01Rik	Smcr8	Hspa12a
Ano5	Unc13b	Siah1a	Mknk2	Acvr1	Tfap2b	Ptpn2	Chst1	Ap3m1	Epgn	Rora	Paqr4	1700052N19Rik	Osbpl11	Nrn1	Tmcc3	Psmb5	Prkacb	Ptprt
Pex7	Asxl3	Ipo7	Anp32a	Bhlhe41	Zfp292	Zfp710	Cep97	Slitrk2	Dmxl2	Epha7	Foxo3	Calu	Zc3h12c	Atf7	Plagl2	Ddx49	Brd4	Mvb12b
Nacc1	Zfp651	Zfp827	Gpr85	Spry2	Tm9sf3	Hydin	Fam49b	Npat	Arap2	Npas3	Gm1818	Pggt1b	Kcnb2	Itga9	Bmp2k	Tmem5	Mbnl2	Gfod1
Psd2	Mbtd1	Pim1	Tomm20	Tmem150c	9930021J03Rik	Nrk	Jam2	Caprin2	Ing5	Cpeb3	Tenm1	Synrg	Elmod2	Tbl2	Daam1	Rab4a	Rarg	Rora
Net1	Ndst1	Maml3	Ilk	Maf1	Srsf11	Peg10	Kmt2a	Mfn1	Pptc7	3830406C13Rik	Abl2	Ndfip1	Lhx5	Sltm	Foxp1	Lyl1	Add3	Sec14l5
Rreb1	Gpr37	Golga3	Abhd14a	Sort1	Ptprn2	Slc2a3	Pcdh11x	Btg3	Prkar2a	Mbnl3	Tfb2m	Stim2	Slco5a1	Rsf1	Xkr7	Lsm11	Prtg	Zhx3
Ptpn3	Rnf150	Mettl1	Sp6	G6pc3	Rgmb	Mkln1	Btbd3	Als2	Klhl20	Mybl1	Alk	Rlim	Trp53inp1	Atl3	Ss18	Plekho1	Nudt10	Pde1c
Med12l	Ireb2	March4	Itpripl1	Ccng1	Ryr2	Ikzf4	Tktl1	Arrdc3	Tspan2	Myrip	Phyhip	Max	Mecp2	Tm4sf19	Nrp2	Flywch2	Rapgef6	Pcnx
Lzts3	Mex3b	Atxn7l3	Pet117	Mbnl1	Creb1	Atp5s	Ankrd52	Acer2	Syt1	Zfp607	4921522P10Rik	Plekha8	Map2	Cttnbp2nl	Slain1	Daam1	Cbfa2t3	Peak1
Sema3e	Dnmt3a	Dnajc21	Gm20708	Ccrn4l	Dnajc6	Zcchc14	Mtor	Capn7	Card10	Nr2c2	Tmem127	Rock1	Xpr1	Got2	Jade2	Tbc1d15	Fam126b	Man2a2
Slc5a3	Morf4l1	Rassf3	Gdf7	Usp46	Armcx3	Bcl7b	Phip	Ccng2	Fam83d	4933411K20Rik	Prss35	Lphn2	Irs4	Scai	Aak1	Mfsd7c	Zfp398	Mtr
Id4	Traf3	Tanc1	Itpkc	Idh3a	Pkp4	Tm9sf3	Zcchc16	Plxdc2	Wdr1	Slc6a9	Kcnq2	Zranb1	Klf12	Rsbn1	Kcnj14	Spin1	Fzd3	Kmt2a
Fxr1	Tcf4	Fgf9	Lgi2	Npepps	Adat1	Aph1b	Pou2f2	Mastl	Taf4a	Xrn1	Srcrb4d	Wwp1	Prkaa2	Nsd1	Eme2	Tmem135	Ahcyl1	Zcchc14
Dnajc3	Rlf	Zfp507	Kctd15	Tns4	Senp6	Ociad1	Farp1	Sgip1	Edaradd	Dlc1	Cecr6	Ar	Cops8	Nudc	Map3k4	Ctps2	Lphn2	Slc39a9
Rnf152	Lims1	Smoc1	Dhrs4	Hnrnpu	Hdlbp	Fam63b	Slco4c1	Soat1	St8sia4	E2f3	Ntsr1	Sh3pxd2a	Edem3	Pygo1	Dap	Hoga1	Inpp5a	Adcy1
Pde4b	Adamts18	Kctd1	Rnf181	Mars2	Agap1	Npas3	Tsku	Mysm1	Cdk19	Slc14a1	Gjb2	Uprt	Plcg1	Rfx6	Sox6	Mamdc2	Tmem108	Cntn2
Sumo3	Hdac4	H3f3b	C130026L21Rik	Nkx1-2	Impad1	Smarcd1	Stmnd1	Lims1	Klf10	Myh10	Rd3	Adcy3	Pard3b	Grhl3	Rab11fip4	Evi5l	Igf1r	Zfp827
Rab11fip2	Ago3	Zfp644	Pde1c	Slc52a2	Ulk1	Tstd2	Galr1	Rnf169	Mastl	Ino80d	Rdh8	Ndst1	Tmeff2	Klf12	Sel1l	Glrx2	Unc5d	Ccdc93
Ncoa1	0610030E20Rik	Irx5	Midn	Bai2	Man1a2	Fzd4	Cdc42bpg	Rhobtb3	Tgif1	Hmg20a	Gm14446	Sgcd	Esyt2	Amd2	Fam57a	Usf2	Dennd5b	Lpgat1
Etnk1	Adam19	Map7d1	Pla2g2f	Mmgt1	Kcnc3	Stt3b	Sh3bp5	Zfp148	Stox2	Tcf12	Rasal2	Pcdh9	Scd1	Prrc2b	Itgb1	Ptgr1	Qk	Trim66
Gnptab	Gan	D1Ertd622e	Src	Trps1	Prdm8	Map2k4	Oxsr1	Dock4	Nfib	Itga2	4930544L04Rik	Setd8	Tsc22d2	Gyg	Pfn2	Zfp474	Tectb	Zfp174
Edn1	Atxn1l	Fgf13	Radil	Adss	Foxj3	Ccdc66	Zak	Eif3j1	Skida1	Acadsb	Zfp467	Adam19	Evi5	Amd1	Tnrc6b	Ier2	Chic1	Hivep3
Upf2	Col8a1	Bcorl1	Ephx1	Cdc42bpb	Hsph1	Arhgap32	Rnf152	Fcho2	Ddx3y	Myadm	Fgfr3	Nr4a3	Slc38a2	Erbb4	Slc30a9	Psmd7	Bach2	Klhdc10
Pvrl3	Cdkl2	Ccnt2	Prelp	Slc4a3	Cbx6	Rif1	Map2k3	Pphln1	Nav3	Hic2	Hpca	Acvr1c	Foxg1	Sez6	Nudt4	C1ql2	Trabd2b	Cep350
POU2F1	Ntrk3	Fam83g	Cxcl13	Brwd1	Iffo2	Dcaf7	Camk2n1	D15Ertd621e	Ccm2	Grin3a	Gmps	Med6	Map3k1	Runx3	Dip2a	Pink1	Zak	Xirp1
Ktn1	Lep	Sox12	Btg1	Rbm47	Phf21a	Zfp281	Ezr	Lrp12	Zc3h12b	Mme	2010002M12Rik	Kdsr	Prkg1	Peak1	Kcnk2	Nub1	Tagln3	Gm15737
Sesn3	Npas3	Stau1	Cpne6	Nacc1	Dgkg	Apold1	Fam160a2	Btbd7	Brwd3	Shroom4	A930016O22Rik	Wapal	Nuak1	Hnf1b	Tmem150a	Fbp1	Tmem50b	Dpp8
Glt8d2	Col24a1	Dkk2	Zfp697	Negr1	Hnrnpd	Mob1b	Nova1	Atp13a3	Rnf11	Phlpp2	Apbb1	Arhgef33	Rps6ka3	Ergic2	Abca1		Dcun1d3	Abl2
Dctn4	Slco5a1	Hnrnpa2b1	Rcor2	Nod2	Fam63b	Hoxc10	Zbtb20	Ube2d3	Slc24a4	Trp53inp1	Amt	Arhgef37	Mgat2	Abca1	Ankrd13c		Mast4	Usp49
Itpk1	Ankrd13b	1810022K09Rik	Olfr701	Slc25a35	Elk3	Zic3	Zbtb37	Nrg1	Zc2hc1a	Thrb	Syt9	Dennd6a	Gm9804	Arhgap21	Gatad2b		Ccbe1	Fam160a2
Palb2	Rnd3	Nkx2-3	P2rx6	Cept1	Mtf2	Dixdc1	Fhl1	Atp7a	Kdm2a	Glcci1	Rgs13	Rps9	Zc3hav1	Fam63b	Tsen54		Fam118a	Pla2g2c
Sec16b	Akap5	Tmem115	Phactr2	Sgsh	Igf1	Sgip1	Itga3	Hecw2	Fam102a	Tgfb2	Rasl12	Tpst1	Col4a3	Cep170	Insr		Foxo3	Tenm4
Mbnl1	Col25a1	A230050P20Rik	Gatsl2	Dlg2	Rlim	C77080	Gulp1	Senp5	Htr1b	Yaf2	Slc39a7	Kdm2b	Flt1	Glcci1	Zdhhc6		Nf1	Rnf144b
Klf12	Mark3	Ptpn12	Vps37d	Foxk2	Grin2a	Zfp462	Prrx1	Iqsec1	Rc3h2	Tmem135	Tppp	Ppp1r3a	Ipo8	Naa50	Ap3m1		Zfc3h1	Adarb1
Ankrd50	Col5a1	Psmb5	Gbx1	Hhip	Hnrnpa3	Col6a6	Slc16a9	Slc18a2	Prr5l	Sobp	Pcdh1	Evi5	Adamts6	Tspan7	Mtf1		Pura	Nek10
Runx1	Kif26b	Ctu2	Zfp740	Sox6	Hnrnpul2	Wfdc5	Abcd1	Pp2d1	Nbeal1	Tmem170b	Rnf150	Cers5	Lca5	Lurap1l	Ash2l		Vps26b	Wnk3
Phlpp2	Dpysl2	Tbx2	Dgcr2	Tgif1	Git1	Crebzf	Foxq1	Camta1	Scn1b	Zfp644	Samd12	Elf4	N4bp2	Tbc1d30	Qk		POU2F1	Smg7
Iffo2	Sparc	Dynll1	Pdcd1	Olfml1	Dcun1d1	Cpd	Rufy1	Fam171b	Klf13	S100pbp	Lcor	Aptx	Ctdspl2	Trim9	Mrvi1		Arhgap29	Igf2bp1
Brwd3	Hif3a	3222401L13Rik	Zfp251	Stim2	Zfhx3	Cpeb4	Socs6	Slc16a9	Cacna1c	Mnx1	1700019D03Rik	Trim26	Cdc73	Dusp9	Lphn1		Cntn1	Gabpb2
Sh3glb1	Adamts6	Mpv17l2	A930011G23Rik	Sp2	Ppp1cc	Rmdn2	Gap43	Tmtc1	Igsf3	3110057O12Rik	Cst10	Nxph1	Slc1a2	Neto1	Skil		Nuak1	Med22
Olfr482	Cecr2	Elfn2	Lrsam1	Pcdh7	Ptprq	Ace3	Tbc1d24	Cbln4	Ccser2	Arid5b	Mcmdc2	Dcx	Ppm1f	Fkbp6	Ppm1e		Nup155	Exoc4
St8sia4	Ky	Rnf34	Vamp2	Tbr1	Mapk6	2410127L17Rik	Atp2b4	Tnfsf8	Jade1	Snx27	Mtmr3	Dcaf10	Rap1b	Asap1	Gpr85		Dscam	Fam163a
Rap2a	Cav2	Idh2	Grasp	Git1	Nov	Ubtd2	Zhx1	Spire1	Appl1	Ccne2	Aldh1a3	Prpf38b	Slc7a11	Cnr1	Rab8b		Evi5	Gpatch2l
Kdm3a	Tmem236	Rufy2	Iffo2	Hp1bp3	Satb2	Mapk9	Tbl1x	Tenm4	Fam46a	Chp1	Best3	Mdm4	Sytl5	Trps1	Tmsb4x		Pgap1	Arhgap11a
Zhx1	Plag1	Tmub2	Ssbp3	Sesn2	Gabrg2	Pfkfb2	Zbtb41	Stox2	Gskip	Nanos1	Pacs1	Tctex1d1	Arid4b	Atp8a2	Eea1		Trim37	Slit3
Cdk5r1	Thsd4	Surf2	Spsb2	Serp1	Fam135b	Rnf139	Ebf3	Dnajc25	Aff1	4930503L19Rik	Elavl3	Stag2	Erg	Fryl	Spry2		Nckap1	Ptpn14
Sbno2	Ap1g1	Chd1	Xpnpep1	Adam10	Slc39a10	Syde1	Ahr	Cep170	Pde5a	Mbtps2	Tspyl4	Plxdc2	Dennd5b	Fmr1	AI314180		Slco3a1	Nek9
Ppp1r9a	Camk4	Aldh1a1	Trim21	Cmtr1	Aff4	Prom2	Zkscan17	Cdon	Sybu	Ak4	Ldoc1l	Nrp1	Otud4	Rev3l	Styx		Mturn	Ppp1r3f
Casp9	Col5a3	Rpl37a	Maml2	Gramd3	Thrb	Trpm6	Rgs2	Krtap20-2	D10Bwg1379e	Spag9	Npbwr1	Arrdc3	Kcna2	Crk	Caps2		Plekha7	Slc7a14
Edem3	Cbx6		Zfp503	Pitx2	Bfar	Myo3b	Onecut3	Hipk2	Fat3	Paqr9	Adrbk2	Slc9a9	Wipf1	Nrk	Mfap3		Ktn1	Kcng1
Mrgbp	Mycn		P2rx3	Stx6	Csnk1g1	Msl2	Sun2	Atp1b1	Zfp518a	Mib1	Rbfox1	Fam84a	Sox5	Zfp516	Yod1		Sox6	Trim56
Adam10	Prdm11		Pgam1	Papola	Suv420h1	Frem2	Ppm1l	Elavl4	Med13	Rapgef5	Aicda	Crkl	Scn3b	Dlat	Rfx3		Brms1l	Plec
Ctsq	Eps15		Umodl1	Gata4	Dync1i2	Mtmr7	Sec14l1	Asxl2	Fmr1	Tfrc	Igf1r	Hgf	Dach1	Ttc7b	Serp1		Lsm14a	Peg10
Fam60a	Dgkd		Epha1	Ccdc97	Msl2	Wnk1	Dnajc1	Nckap1	Magi2	March1	9430015G10Rik	Isl1	Ikzf2	Foxa1	Kcnk10		Pdia4	Rgs8
Mapk8	Ppic		Hoxd9	Calm3	Soga1	Nbl1	Tmem145	Grin2b	Atp10a	Fam126b	Specc1l	Fam168a	Kcnq3	Elmsan1	Csmd1		Fbxo33	Pcnt
Nufip2	Tmc5		Mgl2	Slc15a1	C77370	Sorcs1	Deptor	Pten	Ebf2	Pds5b	Srrm3	Cbx8	Gli3	Farp1	Zfp451		Rab6b	Zzef1
Fn1	Eml4		Sp9	Dand5	Celf2	Cttnbp2	Slc24a4	Iltifb	Wnk3	Kpna4	Chat	Fam134c	Wapal	Tmem263	Cnot6l		Wasf2	Abr
9130401M01Rik	Gid8		Tfe3	Syt7	Socs7	Rab30	Nkap	Ccdc88a	Mtmr10	Slc23a2	Atl3	Elfn2	Cep350	Sephs1	Sidt2		Slc44a2	Fam168b
Map3k2	Cyth3		Syt2	Braf	Acan	Usp37	E2f5	Dynlt1b	Mycn	Dnajc13	Syn1	Cables2	Fignl2	Cerk	Slitrk1		Thbs2	Klhdc7a

miR-199-3p	miR-29-3p	miR-486-5p	miR-361-3p	miR-122-5p	miR-425-5p	miR-136-5p	miR-96-5p	miR-142-5p	miR-19-3p	miR-141-3p	miR-770-3p	miR-335-5p	miR-200-3p	miR-194-5p	miR-183-5p	miR-341-3p	miR-182-5p	miR-370-3p
Mtor	Gm17296		Ahrr	Mipol1	Taok1	Pwwp2a	Myrip	Dynlt1f	Rgs17	C78339	Cbfa2t3	Arf3	Nbr1	Pmepa1	Tcf7l2		Zbtb37	Icmt
Ino80d	Fem1b		Hpse2	Plcb3	Cux1	Phex	Slc16a7	Fbxo30	Crebrf	Ythdf2	Ganc	Vav2	Ptpn21	Rif1	Bzw1		Fam134b	Sh2d1b2
Vamp3	Xkr6		Traf1	Mtf2	Ppp2cb	Gcnt3	Lcp1	Ankrd28	Mbnl2	Rere	Bmp8a	Tmem260	Scrt2	Ppfibp1	Scyl3		Limch1	Ccnj
Pde1a	Foxn2		Zcchc13	Map3k12	Uxs1	Mtmr4	5730559C18Rik	Tbc1d9	Rgl1	Fam46c	Dock3	Atp1b1	Asap1	Pak2	Tceanc2		Med12l	Kcnk3
Col12a1	Cldn1		Spdef	Mapre1	Lif	Alpk3	Bach2	Cpeb3	Cbx7	Zfr	Tub	Naa25	Adamts8	Tln2	Mapk4		Phf21b	Grik3
Agl	Fam126b		Gm10097	Pigv	Smek1	4921524J17Rik	Kcnc2	Nsun3	Kpna3	Sp4	5830462I19Rik	Cdk13	Rasa2	Taok1	Snx30		Plekha8	Unc5d
Hnf1b	Pdgfc		Kctd5	Hist1h3h	Sall3	Zfhx2	Plagl1	Onecut2	Smarca2	Aco1	Slc6a9	Tfdp2	Rap2c	Adam17	Slc44a1		Mme	Mical3
Chka	Kif26a		Rdm1	Amigo1	Pgk1	Atmin	Nacc1	Kit	Otud7b	Ttbk2	Egfem1	Npas4	St6galnac5	Krr1	Muc15		Hoxa9	Gm10676
Snn	Pcsk5		Hoxa10	Rad21	Hoxc5	Ammecr1l	3110021A11Rik	Plxna2	Mapre2	Abi2	Syt7	Arih1	Foxn2	Cask	Zeb2		Slitrk4	Gpd1l
Serpine2	AF529169		Cflar	Ank1	Arel1	Zbtb5	Samd12	Stk38l	Sbno1	Ptch1	Dpysl5	Sh3bp5	Gprin3	Cks1b	Pex19		Rsc1a1	Eda
Cd44	Ppp1r3d		Fam168b	Morc4	Atp8a2	Patl1	Gpm6b	Six4	Gulp1	Tet3	6330416G13Rik	Lonrf1	Nr3c1	Trim32	Pkp4		Abcd1	Brcc3
Txndc15	Hmgcs1		Man2a2	Msn	Nrarp	Fbxl14	Pdk2	Rassf2	Zdhhc18	Gnaq	Flt1	Rsrc2	Taok1	Trp53inp2	Smco4		Arhgef3	Prkcb
Pdgfra	Col1a2		Ubap1	Vsig10l	Mast4	Ensa	Pde8b	Zfp770	Etl4	Evx2	A430105I19Rik	Naa50	Tbc1d12	Nwd2	Npas3		Zzef1	Cmklr1
Appbp2	Arf2		1600002H07Rik	Fam219a	Arfip1	Psme4	Slc25a25	Usp9x	Adcy9	Sept8	Ar	Pgf	Usp27x	Ltbp1	Celf1		Plxdc2	9830107B12Rik
Hipk3	Eva1b		Gnpda1	Tfdp2	B3gat2	Nsun3	Gm608	Rhoq	Igfbp3	Celf1	Zfp366	Lbh	Cdk12	Zfyve9	Cx3cl1		Fndc3b	Samd12
Pip5k1b	Stmn2		4932438H23Rik	G3bp2	Rnf219	Grid1	Lpp	Pum2	Rin2	Chd2	Cd300lf	Srgap1	Fez2	Pafah1b1	Plcb4		Ankrd34b	Rgag4
Ndrg1	Kdm5a		Kif26a	Slc9a1	Nras	Tsc22d2	Sesn3	Med12l	Pura	Kcnj15	Slc37a1	Sgms2	Mtfr1	Sox6	Sin3a		Tmem145	Mtf1
Gpm6a	Dcx		Ap3s2	Iqgap1	Nbea	Htatip2	Chic1	Mier3	Rnf167	Kctd20	Col5a1	Nus1	Kcnd2	Fign	Foxo1		Ago3	Kndc1
Pik3cb	Shroom2		4933411G11Rik	Tmem87a	Meis1	Slc7a2	Trib3	Appbp2	Mecp2	Tfap2c	Tmc8	Ptpra	Ap1s2	Dnmt3a	Rps6ka3		Cnot6l	Dsg2
Zhx2	Gk5		Cdx4	Kif5b	Agfg1	Kcnh7	Mtss1	Dlg1	Jazf1	Scd2	Sertm1	Fkbp1b	Med13	Ralgapb	Pde4d		Lpp	Nfrkb
Nox4	D630045J12Rik		Apol7e	Galnt1	Tmem260	Creb1	Usp45	Spag9	Chl1	Cd47	Hipk2	Sptssa	Wnk1	Lin28b	Slc35a1		Ntng1	Prkar2a
Aplp2	Gtf3c4		Mfge8	Fkbp5	Ctnnd1	Cyb5rl	Ash1l	Gm20388	Dock3	Kcnk5	Gpr17	Trim67	AI593442	Slc30a1	Ltn1		Pcx	Hip1r
Nemf	Slc6a2		Gm20489	Lrp10	CTNND1	Ppp1r18	Soga1	Galnt2	Clip4	Tmem229b	Chtf8	Crnkl1	Elavl2	Prkd3	Ammecr1l		Magi1	Lhfpl4
Cblb	Pgap1		Gga2	Ms4a6c	Atp1b1	Fam120a	Cep170b	Dynlt1a	Acbd5	Fbxl20	Dusp18	Proser1	Nab1	Pan3	Rreb1		Ppfibp1	Klhl3
Luc7l3	Mcl1		H2-D1	Dlat	Ano8	Tmem185b	Sv2c	Dynlt1c	Zbtb4	Stag1	Ptch1	Gm10000	Palm2	Kdm5a	Gramd1b		Sh3bp4	Cox17
4933434E20Rik	Elovl4		Osbpl5	Senp1	Pex5l	Mllt11	Ptpn4	Myo9a	Phf12	Usp9x	Klf7	Mboat2	2700081O15Rik	Ube2w	Dcx		Eif3j1	Bmp10
Pon2	Pigm		E2f8	Large	Ankhd1	Tubgcp6	Ankrd45	Wwp1	Chmp1a	Fcho2	Fyco1	Thada	Trpv3	Atp6v1h	Morc3		St6galnac3	Kremen1
Ankrd17	Adamts16		Tbc1d16	Gtf3c2	Baz2b	Arhgef15	Sema3a	Galnt3	Bmpr2	Arf2	Epha8	Nucks1	Pcsk2	Mfsd4	Ern1		Ncoa4	D10Bwg1379e
Ddr2	Rgs8		Sfrp5	Epo	Cenpo	Zbtbd6	Abca2	Fbxo45	Rimkla	Hcn1	Thbs1	Tmod2	Ypel2	Zic1	Dusp10		Gm6768	Slc45a4
Ippk	Gas7		Gm10676	Slc39a8	Fam60a	Paqr9	Ajap1	Fign	Prkaa1	Gm5423	Wnt9b	Tceal8	Agfg1	Tmem41b	Ppp2r2a		Sesn3	Ttc28
Lonrf3	Rel		Med22	Ccdc6	Erlec1	Rgs4	Slc18a3	Gabrg3	Ntrk3	Prkce	Zfp316	Fam78a	Spag9	Ube3a	Gmfb		Ldlr	Pogk
Gja1	Zdhhc5		Ykt6	Clic5	Bclaf1	Heph	Tmem169	Rc3h2	Fam83g	Tcerg1	Xpo4	Btaf1	Trim33	BC030336	Pctp		Vldlr	D130043K22Rik
Golim4	Prkab2		Cidec	Atp6v1h	Snx12	Radil	Cacng5	Gm4847	Zbtb46	Pten	Mkl2	Zmym4	Dtna	Tmem167b	Ypel5		Cnnm3	Nod1
Cdc14a	Hmcn1		Adcy9	Dicer1	Rnf186	Arl10	Vat1l	Chsy3	Sh3rf3	Csnk1e	Dusp8	Eef2k	Hs3st1	Mef2c	Med1		Whsc1l1	C2cd2
Ank2	Zfp174		Rbbp9	Mrrf	Scamp1	Pdk3	Prrg3	Ubl3	Adcy7	Rcor3	Zfp651	Fem1c	Katnal1	Lpin2	Smpd3		Spin1	Ccdc82
Fubp1	Bmf		Xkr7	Bdnf	Tmem127	Rbmx	Tnrc6b	Nfia	Stk35	Txnip	Rhoa	Zeb2	Pikfyve	Ptpn12	Faxc		Maf	Lgi2
Phf6	Eml5		Pin1rt1	Hdhd2	Atp5g3	F13b	Erlin1	Trhde	Zfp367	Greb1l	Gabrg3	Usp49	Rps6ka6	Socs2	Adck1		Gab2	Rprd1b
Ptpru	Fam167a		Cpeb1	Ihh	Ephb3	Kcnd1	Arl10	Ube2a	Macf1	Tbx4	Tas2r143	Psmb2	4932418E24Rik	Bclaf1	Ppp2cb		Syndig1	Slc29a3
Tmsb4x	Col4a6		Sebox	Mef2d	Hoxb9	Ap3m1	Ube2l3	Slc25a40	Pten	C230062I16Rik	Tmem201	Usp28	Cux1	Nrip1	Gbp7		Jazf1	Gnaq
Zfp710	Tmem169		Dpysl4	Ptbp2	Gtdc1	Hspa13	Mab21l2	S1pr1	Snx25	BC007180	Anks1b	Zfp592	Sema6d	Mgat4a	Amd1		Casp2	Heg1
Vangl1	Slc30a3		Carhsp1	Csk	Ppp3ca	Gse1	Gm20388	Zfp827	Palb2	Crebrf	Stx1b	Ahsg	Klf3	Trip12	Plxna4		Tmem170b	Peg12
Nfia	Bak1		Dync1li2	H1f0	C230052I12Rik	Arhgap5	Galnt2	Tmf1	Ccdc88a	Dmxl1	Sohlh2	Kcnh5	Tbc1d2b	Sufu	Spopl		Coro1c	Dcaf17
Dnmt3a	Zbtb5		Gdf11	Plch1	Kcna4	Arid5b	Slain2	Mal2	Dgkk	Gata6	Vav2	Bcl2l2	Igsf10	Rc3h2	Amd2		Sh3pxd2b	Nf1
Zeb1	Snx24		Ankrd52	Stk24	Sema6a	Ccdc162	Spen	Gsk3b	Evi5l	Mcoln3	Apln	Senp2	Rbfox3	Fnbp1	Syt1		Ptprn2	Map2
Emc7	Foxo3		Shisa2	Oprd1	Mrc2	Acsf2	Alk	Fgf13	Rbms3	Slc39a9	G3bp1	Kdelr1	Med1	Pcmt1	Brms1l		9330159F19Rik	Dnmt3a
Arhgef12	Tmem178b		Tmem235	Dlg4	Txnl1	Hspa2	Pet117	Itga8	Ndfip2	Ube3a	Arhgap31	Fmr1	Phtf2	Sdc4	Plekha3		Lonrf2	Arhgap19
Add3	Trp53inp1		Nr4a1	Cbl	E130309D14Rik	Sumo2	Mcf2	Actn4	St3gal5	Lrrtm2	Aadacl3	Rassf2	Slc30a4	Tcf7l2	Idh2		Gnaz	Flywch1
Patz1	Slc7a1		Cacng7	Dhfr	Chpf2	Tmem161b	Cdh20	Rad21l	Pcdh1	Cdk13	Mthfr	Gorasp1	Creb5	Pspc1	Fam81a		Phf20l1	Usp37
Cbll1	Megf6		Nkx2-4	Ms4a6b	Cnnm2	Phf21a	Gnai3	Btbd10	Prune2	Dyrk2	Pogk	Slit3	Greb1l	Rab6b	Robo2		Ebf3	Slc36a4
Aebp2	Slc24a4		Aoah	Gatad2b	Matr3	Entpd1	Celsr3	Ldb2	Tshz3	Lgalsl	Camk1d	Kdelr2	Six1	Meis2	Kif13a		Tro	Slc7a11
Fat3	Abcb6		Pdgfrb	Gas2l2	Orc4	Dld	Bai3	Cic	Rab18	Lpin2	Slit1	Eif4a2	Tfap2a	Zfp395	Pdgfd		Xpr1	Zfp704
Fbxw11	Mfap3		Ecel1	Daglb	Ndufaf7	Pmp22	Mbnl1	Arhgef6	Ppp1r12a	Sost	Golga1	Eme2	Zmym4	Mrpl47	Slc25a20		Ptpn4	Ncoa1
Dzip1l	Palm2		Sgsh	Ric3	Sdhaf2	Prpf4	Lrch2	Cask	Cnot6l	Memo1	Rnf216	Adcy5	Crip2	Cep350	Cdk5r1		Aebp2	Sh3pxd2b
Wapal	Erlin2		Tmprss11e	Gabrb1	Col14a1	Lmtk3	Camta1	Rc3h1	Sesn3	Hipk1	Mvb12b	Slc26a9	Arih1	Ccr10	Ppp2r5c		Kctd16	Gm6760
Kl	Pmp22		Tnfaip8l2	Tmem126a	Habp2	Fam122a	Gxylt1	Stx3	Unc93b1	Rbm12	Slc9a7	Scrt1	Scd2	Mtss1	Hdhd2		Fam168a	Runx1t1
Uxs1	Atp2b1		Apol7b	Haao	Cep57	Mat2a	Hspa13	Ddx6	2510039O18Rik	Stx2	Zfp345	Nipa2	Slc14a1	Lrrc4c	Mecom		Slc39a9	Fcho1
Cited2	Kdm2a		Crcp	Zfp689	Ppp2r3a	Dlg1	Ube2g1	Car8	Unc13a	Srp72	Kcng3	Rbfox2	Negr1	Uba2	Rab9b		Cacna1b	Nkd2
Ywhae	Zfp346		Rap2b	Shisa4	Sla2	Cmtr1	Fam178a	Gopc	Ago3	Ctnnd2	Prrc2c	Gpr75	Kdm7a	Btbd7	4931406P16Rik		Celf1	Atf6
Cdk17	Suv420h1		Calml3	Emx2	Tlcd1	Gm10742	Snx29	Rap1a	Mecom	Slc30a7	Wdfy3	Dclre1b	Ccnj	Slc30a10	Atl2		Prdm1	BC034090
Depdc1b	Foxj2		Popdc2		Stk39	Mgrn1	Cpeb1	Tsc22d2	Ankrd50	Fam96b	Son	Fam131b	Tbx5	B630005N14Rik	Ccdc43		Ppp2r3a	Rassf2
Crim1	Col5a2		L3mbtl1		Olfml1	Naa11	Fosl2	Rffl	Shank1	Sorbs2	Smyd1	Myo1d	Tcf4	Sdad1	Pdcd6		Celf2	Capn8
Slc7a1	Mybl2		Cdk10		Arl3	Rad51	Map3k3	Fgf15	Sema4c	Tgfbr1	Magi2	Dmrt2	Cul5	Dtna	Hs3st1		Gpr155	Zfp568
Gpbp1l1	Sp1		Il17rd		Tgfb1	E2f1	Slc22a5	Zfp503	Rad18	Stxbp5	Ndor1	Zfp326	Slit2	Adamts6	Arhgef18		Dcun1d1	Zfp275
Flrt3	Narf		Afmid		Pln	Cd99l2	Nhlh2	Rhoa	Fam114a1	Sptan1	Fpgt	Sept7	Nptx1	Trp63	Dtna		Ept1	Ube2h
Tgif2	C1qtnf6		Cnot3		Id1	Rab21	Irs1	Efna5	Ccdc142	Hecw1	Ino80c	Hnrnpa2b1	Mmgt1	Sec31b	Csnk2a1		Kcna2	Rbms3
Pou2f1	Fbxl18		Nr2c2ap			Pdzrn3	Kpnb1	Tenm3	Lrrtm2	Ranbp9	Scn3b	Agmo	Pik3cb	Map2	Usp47		Fgf9	Ccdc15
Arl15	Kctd3		Map3k10			Cir1	Serpinb2	AI467606	Hic1	Clcn5	Mfsd12	Acbd4	Klf9	Zc3h12d	Rpia		Muc19	Bahcc1
Sh3rf1	Samd4		Foxk1			Gng3	Tmx4	Twistnb	Mylip	Jazf1	D130040H23Rik	Igf2bp2	Tardbp	Hs3st2	Dpp8		Scrt1	Olfr1133
Mtf2	Nek10		Adap1			Zbtb6	Rnf183	Cul4a	E130309F12Rik	Spry4	Asb1	Emc6	Abat	Rell1	Thsd7b		Onecut3	Mical2
Ube2h	Efna5		Ebf1			Cecr6	Slc10a3	Setd2	C87436	Foxp1	Ccdc50	Tgm2	Fosb	Tnpo1	Spcs2		Pdzd8	Shisa2
Ube2w	Senp1		Lypd6			Myoz1	Tgfbr1	Tiam1	Bnc2	Jag1	Cpsf2	Asah2	Ube2w	Klf7	Sik3		Ralgps2	Mill2
Bend7	Ibsp		Npas4			Acvr1b	Col25a1	Scn4b	Sv2a	Dcp2	Gfod1	Cnn1	Fhod1	Sla2	Cpeb1		Clptm1l	Sh3pxd2a
Gfod1	1810013L24Rik		Ets1			Rbm15b	Ttyh3	Sgcd	Klf7	Uba6	Jade2	Spidr	Arl5b	Enox1	Pde3a		Ostm1	Gm608
Rfx7	Lrp6		Zfp930			Hs3st1	Itpr2	Trpm7	Lsm12	Mtf2	Tanc2	Prkaca	Ranbp9	Myl4	Kpna4		Bcl2l12	Fam199x
Slco4c1	Erp29		Rnf222			Gosr1	Gid4	Sgrap1	Sgip1	Ralgps2	Pou2f2	Cnih4	Gabpa	Chd4	Col25a1		Gpr85	St6gal1
Mtf1	Ccnd2		Ccdc162			Xpo7	Dhcr24	Selt	Prpf18	C1galt1	Ppard	Nop16	Ctnnd2	Tra2b	Runx1t1		Fam43a	Pard3b
Gpr171	Nos1ap		Pskh1			Ipo7	Mob1b	Fam19a1	Hnrnpul2	Camsap2	1700009J07Rik	Med9	Ndst1	Sox11	Rala		Asun	Knop1
Slitrk6	Fbxw7		Zfp346			Samd8	Ginm1	Mbd2	Cnksr2	Rasa2	Prok1	Gstz1	Nova1	Rheb	Gse1		Fign	Fam102a

miR-199-3p	miR-29-3p	miR-486-5p	miR-361-3p	miR-122-5p	miR-425-5p	miR-136-5p	miR-96-5p	miR-142-5p	miR-19-3p	miR-141-3p	miR-770-3p	miR-335-5p	miR-200-3p	miR-194-5p	miR-183-5p	miR-341-3p	miR-182-5p	miR-370-3p
Scube3	Col27a1		Cxcr5			Prorsd1	Nus1	Yod1	Chsy1	Sox5	Cspg4	Hdhd2	Nog	D430041D05Rik	Zbtb34		Dcbld1	Accs
Extl2	Col6a2		Bahcc1			Ankrd63	Dchs1	Tgfbr2	Fam162a	Prkacb	Hist2h2bb	Eif4e2	Vat1l	Itgbl1	Mapkapk2		Tmem115	Samd4
Itga8	Smek2		Pgpep1l			Tcf12	Rundc3b	Clcn5	Sgtb	Peli1	Cd209c	Glipr2	Cdh11	Trib2	Sp2		Nus1	Ceacam2
Llgl2	Stag2		Defb20			Ahcy	Edem1	Atxn7l2	Mxd4	Ncoa2	Tmem184a	Hrct1	Lats2	Mib1	Celsr3		Hook3	Ttll4
Pawr	Lox		Hoxb1			Ncoa4	Bmp2k	Lphn3	Klhl28	Leng8	Lrrc59	Frk	Cdk17	Slc7a5	Dagla		Rimkla	Tppp
Zfp217	Taf5		Tmprss5			Tspan3	Pik3c2a	Cisd2	Wnk1	Dcx	Tvp23a	D1Ertd622e	Zc3h6	Adam12	Pcdhga12		Kcnc3	Disp2
Abhd4	Nup160		Slc48a1			Pole	Dgkh	4932411E22Rik	Dlx1	Brd4	Edaradd	Gabrb1	Plcl1	Tmed2	Picalm		Shc4	Rnf185
AU040320	Slc43a2		Znrf1			Kcna4	Tcf4	Yy2	Purg	Klf6	Golga7b	Ebf4	A830080D01Rik	Pip4k2b	Dock4		Pi4k2a	Smarcd1
Zfp281	Nudt11		Duoxa2			Cd24a	Dpy19l3	Slc39a8	Ankrd12	Zcchc24	Scrt1	Cul4b	Nrg1	Hspa4l	Msantd3		Ss18l1	FMN1
Prdx6	Mafg		Nr3c2			Nova2	Pcdh17	Rab6b	Hipk2	Apbb2	Rs1	Gspt1	Slc23a2	Pitpnm2	Scn3a		Pde3a	Tnfrsf19
Slc23a2	Ypel2		Ncf1			Hhip	Gm16039	Etv1	Fam179b	Gigyf1	4933411G11Rik	Mms19	Ythdf3	Opcml	Cdyl2		Fam49b	Xrcc3
Ywhag	Fbn2		Il7			Elk1	Zfhx4	Cdh12	Abhd10	Camsap1	Cdc42bpb	Itih4	Ppp1r9a	Lsamp	Arhgap18		Ubxn10	Fgd6
Mmp24	Apcdd1		Doc2b			Golim4	Ppp3r1	Set	Ddx3x	Dync1li2	Lzts2	Anapc15	Hspa13	Itsn1	1810013L24Rik		Dusp4	Arid3b
Tanc1	Plp1		Tssk4			Kcns3	Cpsf7	Rnf146	Plag1	Ccdc6	Camkk1	Ahsa2	Zfp532	Evx2	Plcxd3		Kdm5a	Zfp607
Iqgap1	Vegfa		Klhl5			Pbx3	Cpeb4	Mdga2	Gigyf1	Mbtd1	Rab11fip4	Stx8	Fam217b	Gm10033	Arid4b		Ank3	Sec24b
Ankrd61	Col22a1		Fbxl8			Msantd2	Insig2	Grsf1	Yipf6	Calcr	Dcx	Eif2b3	Fmnl2	Exoc5	Ncs1		Kcnj3	Mcc
Rbm47	Wdr26		Slc6a9			B230219D22Rik	Slc2a13	Pappa	Btf3l4	St3gal3	Klhl29	Comtd1	Apaf1	Ncl	March4		Ube2q2	Fras1
Phyhipl	Larp4b		Vipr1			Rbms2	Casp2	Man1a	Ano1	Fam168b	Lhx6	Acadsb	Phactr2	Bmpr1b	Phf3		Creb3l1	Cacna1c
Tead1	Enho		S1pr1			Rybp	Lims1	Meis1	Snx17	Cadm2	Add3	Txnl4a	Golga7	Rfx3	Rnf157		Trp53inp1	Als2
Meis2	Usp34		4632428N05Rik			Ret	Bcr	Gas7	Nrk	Hdac4	Hsdl1	Gtf2b	Zc3h4	Akt2	Zfp592		Ednrb	Palm2Akap2
1700025G04Rik	Ptbp3		Gm4951			Nepn	Morf4l1	Pcmtd1	Mybl1	Iqsec1	Usp31		Srp54a	Efnb2	Zfpm2		Tmem260	Baiap3
Fam53b	Atp5g1		Gm5577			Kcnk3	Gltscr1l	Tfap2b	2810403A07Rik	Snap91	Sdc3		Aff3	Acsl3	Epb4.1l5		Ebf1	Chd2
Asap2	Hecw1		Siglecg			Ptbp2	Abca1	Macf1	Tnpo2	Msantd4	Tbc1d8b		Ankrd44	Tjap1	Tram1		Sept7	Ar
Nacc2	Iffo1		Loxl2			Ntn3	Prima1	Zfx	Mier3	Peak1	Gm9821		Tbc1d22b	Slc40a1	Zfp609		Elmo1	Onecut2
Wnk1	Zbtb40		Adra2c			Stx12	Kras	Runx1t1	Sdc1	Klhdc10	Eda		Fat3	Elf4	Lrrc58		Atp8a1	Plxna2
Ptprf	Gxylt2		Erg			Tnpo2	Abat	Klf11	Mob1b	Csnk1g1	Trhr		Scn2a1	Jmjd1c	Med12l		C2cd2	Wnt2b
Naa25	Elf2		Rxra			Ptprr	Cnot6l	Slc16a12	Slc35f1	Lifr	Sema6b		Rab8b	Depdc1b	Sema3e		Slc39a1	Kif1b
Ddhd1	5730508B09Rik		Ankrd45			Suox	Avil	Zcchc14	Kcnk10	Socs7	Nyap1		Fndc3b	Chd9	Cux1		Cdv3	2900026A02Rik
Sypl	Tnrc6b		Fam102b			Etf1	Dcaf10	Sec23a	Heg1	Pdzd8	Pbx1		Xrn1	Pgm2l1	Klhl3		Phip	Snx29
1110002E22Rik	Pgap2		Ccdc120			Lin52	Clvs2	Map3k1	Lonrf3	Cyp26b1	Gpr137c		Vcpip1	Paip2	Rbmx		Greb1	5730409E04Rik
Tra2b	Frat2		Med10			Mink1	Slc12a5	Zfp651	Cgn	Hs2st1	Hlcs		Gm6104	Cnksr3	Tsc1		Slc1a1	1110002L01Rik
Bbx	Gpr101		Zfp703			Thap11	Hoxa5	Dlgap1	Sipa1l1	Rheb	Hic2		Hic2	Phyhipl	Ptdss1		Fam120c	Prrc2c
Atl1	Syncrip		Ttl			Slc39a5	Gna11	Rnf165	Slc9a6	Upk3b	Txlna		Pls3	Chd8	Fndc3b		Pdzd2	Yars
Kpnb1	Traf4		Atcay			A930016O22Rik	2810459M11Rik	Prpf40a	Plxnc1	Urgcp	Clstn1		Rnf169	Tsc22d2	Ick		Ahr	Gabbr2
Iqgap2	Sptan1		Olfr288			Tmem9	Rictor	Ankib1	Mapk14	Gcnt2	Fbxl7		Nr2c2	Smc3	Fam118a		Tapt1	Kif26b
Rorb	Arrdc3		Dkc1			Kcnk10	Fzd3	Bnip2	Tgoln1	Ppp1r15b	Nfam1		Dnajc5	Snx1	Nrbf2		Gdf6	Trim44
Atf6	Clcn5		Espnl			Pde6h	Nlgn2	Msrb3	Slc30a7	Ppef1	Prss33		Lpar1	Bicd1	Auts2		Rnf144a	Akap2
Ccdc88a	Pten		Mypop			Fam58b	Jmjd1c	Wdr26	Mycs	Reck	Arhgap32		Pdik1l	Csnk1d	Arhgap21		Kdelr2	Gatsl2
B3gat2	Insig1		Otulin			C8b	Pik3ca	Sass6	Poglut1	Aktip	Asb13		Mapre1	Rnf11	Slitrk3		Npy4r	Ceacam1
Hhip	Strn		Flnc			Ncaph2	4930402H24Rik	Klhl14	Nr3c2	Map7d1	Klk4		Ptprz1	Rap1b	Fam78b		Astn1	Zmiz1
Myo5a	Gm608		Gabarap			Tmem241	Slc25a1	E130311K13Rik	Napb	Sim2	Cnr1		Sulf1	Ctage5	Hoxd8		Dmxl1	Xpnpep3
Rgs4	Akap17b		Rxfp3			Thoc6	2610528J11Rik	Sumo2	Tnrc6c	Map2k4	Ago1		Eps8	Tmed9	Zfp292		Etl4	Sugp2
Fbxo28	Cps1		Krt9			Rfwd2	Tc2n	Herpud1	Siva1	Pou3f3	Pomt1		Mprip	Atf2	Flrt3		Sort1	A630007B06Rik
Fndc3a	Plcxd2		Nbl1			Prss12	Slc16a13	Atf1	Syncrip	Atp8a2	Cux1		Cbx4	Ppargc1a	Tle4		Ell	Klhl24
Fgf10	Ybx3		Ash2l			Nanos1	Gprc5b	Cdh9	Sgk1	Erc2	Rilpl1		Ubn2	Lats1	Uchl3		Ppp1r9a	Nhlrc3
Pcmtd1	Shroom4		Agpat4			Rdh7	Dpp8	Cylc1	Id2	Maml2	Zfp457		Cnot6	Cadm1	Gria1		Arhgap31	Onecut1
Fign	Dnmt3b		Kif12			Elfn2	Map3k2	Zdhhc20	Slc9a1	Nsun3	Pex26		Ppp1r9b	Lphn2	Ing3		Celf6	Prob1
Mef2a	Antxr2		Il11ra2			Tfam	St7	Spcs2	Esr1	Rnf145	Syt2		Frem1	Gmfb	Tcf12		Foxn2	Il6st
Vps41	Hcfc2		B230104I21Rik			Mtch2	Lhx1	Musk	Rictor	Nrxn1	Soga1		Dock4	Adam23	Mdm4		Cd2ap	Flt3
Tacc2	Nckap5		Btd			Traf6	Bicd2	Tns1	Grb10	Pdzd2	Phxr4		Smg8	Rbm39	Bcl11b		Dgkk	Ttyh3
Nutf2	Rnf152		Pim2			Nr1h4	Tmcc3	Adamts5	Cnot6	Lhx1	Napg		Mtf2	Elf2	Hsph1		D3Bwg0562e	Itga2
Slc17a9	Atrn		Pdss2			Tbx6	Myo19	Mak	Kalrn	Schip1	Ccdc88b		Iqsec1	Atad2b	Uchl4		Gmfb	Primpol
Xpo7	Zbtb46		Iigp1			Sri	Rgs17	Fam134b	Rab8b	Cdon	Susd5		Cacna2d1	K230010J24Rik	Mef2a		Stk17b	Mgll
Dlx2	Hip1		Gnb5			Commd1	Rhpn2	Cntn1	1110002E22Rik	Epn2	Maml3		Nova2	Birc6	Slc8a2		Wfdc9	Rab3gap1
Foxm1	Zfp282		Tmem161a			Lyl1	Igsf11	Ttpa	Vsig10	Sdad1	Shisa9		Ccnyl1	Zfp503	Mta1		Onecut2	Camk4
Ppm1a	Rfx7		Rbbp4			Mospd1	Rdh10	Mars2	Ell2	Cdk17	Chd6		March8	Atp5g1	Aff4		Pirt	E330009J07Rik
Cfl2	Eomes		Lefty2			Brk1	Grid1	Zfp191	Pcdha10	Grb2	Kmt2d		Psip1	Tfrc	Nubp1		Grhl2	Zfp276
Cpd	Bace1		Folh1			Lmo4	Tmem178b	Ubr3	Rfx1	4930452B06Rik	Snx29		Acaca	Pfkfb2	Usp2		Anxa11	Nsun4
Plxdc2	Jazf1		Fam107b			Rpp21	Asxl3	Gstcd	Rap1b	Map3k7	Hira		Asxl3	1700025G04Rik	Sept7		Cep83	Edc4
Tspan14	Ldlrap1		Hpca			Mtx2	C1galt1	Cnep1r1	Atg16l1	1700025G04Rik	Hspg2		Heatr5a	Spcs2	Ezr		Rprd1b	Stard9
Chad	Gjd2		Magel2				Cdc73	Kcnj3	Kif13b	Elmsan1	Mllt6		Tmppe	Pdhb	Apoo		Tead1	Fyco1
Zkscan1	AI593442		Cdk5r2				Lrig1	Prdm11	Fbxo36	Rab30	Veph1		Nedd1	Rad51d	Myo1b		Taf15	Myt1
Raph1	Fer		Erh				Trpm3	Prox1	Nrbp1	Rph3al	Gjc3		Hs2st1	Eif4g2	Rock1		Snx29	Decr2
Stim2	Chsy1		Slc12a1				Sdc2	Apln	Cltc	Bend4	Nsd1		Clip1	Nacc2	Thsd7a		Ankrd52	AW011738
Pde7b	Zfp91		Atxn7l3				Cep170	Fam73b	Atxn1l	Foxn2	Atf6		Slc4a4	Tgif1	Pvrl3		Lancl3	Micall1
Rnf216	Dennd1b		Tmem35				Tbx1	Ubr1	Lrtm1	Tada1	Pcdh19		Klhl29	Eya4	Prune2		Mef2c	Ahr
Mef2c	Col4a2		Apln				Smek1	Prtg	Arih2	Acvr2a	Hmgcll1		Npc1	Col4a3bp	Sppl2a		Wipi2	Hmga2
Fgl2	Slc16a7		Actr5				Rab8b	Prrg4	Med13l	Fem1c	Stard9		Tob1	Matr3	Agap3		Igdcc3	Nwd1
Arhgef3	Oxtr		Sox4				Coro2b	Zbtb37	Dnaic1	Rnf44	Olfr1349		Jun	Prss53	Myt1l		Vwa3b	Fbf1
Pde7a	Adamts10		Gpnmb				Kcnc3	Elavl2	Atxn7	Ywhag	Stac2		Nanos1	Syvn1	Nr4a2		Prlr	Mgat4a
Cdc42	Pcdh17		Gm10369				Rab23	Foxa1	Sh3d19	Atp6v1a	Gm14137		Sfxn1	Rab11fip3	Socs6		Glb1l	Ptrf
Csrp2	Hcn1		Ifng				Atg16l1	Armc8	Eea1	Slain2	Dgkd		Tceb1	Tox	Ctdsp1		Wdr78	Mettl16
Slc30a1	Cep68		Ddit4				Satb2	Zcchc11	Cdc42bpb	Slit3	Aak1		Efna1	Dennd4b	Pja2		Fnbp1	Susd4
Lrrc1	Ror1		2010001E11Rik				Frmpd4	Gk5	Fam168a	Nckap5	Ngp		Lysmd3	Kif13a	Lsm11		Ppil1	Mfsd6
Mcfd2	Crispld1		Gphn				Myo16	Cep97	Lmln	Ptpn21	Gm9804		Prdm16	Ralgps1	Sgms1		Clic5	Senp1
Sik2	1810041L15Rik		Aph1a				Pou2f3	Stk35	Spty2d1	Ctbp2	Lrrc41		Tmem41b	Ptpn13	Zic3		Prrx1	Ppp1r9a
Vtcn1	Hmgcr		Fam20b				Npnt	Nfe2l2	Rab33b	Lrrc8a	Stom		Esco1	Rbbp4	Tet1		Dos	Ank2
Crebrf	Ahr		Gm9875				Eea1	Snx16	Asxl2	Arhgef18	Igfbp4		Brwd3	Mtx2	Dync1i1		Fmr1	Gdf7

miR-199-3p	miR-29-3p	miR-486-5p	miR-361-3p	miR-122-5p	miR-425-5p	miR-136-5p	miR-96-5p	miR-142-5p	miR-19-3p	miR-141-3p	miR-770-3p	miR-335-5p	miR-200-3p	miR-194-5p	miR-183-5p	miR-341-3p	miR-182-5p	miR-370-3p
Arid4b	Akt3		Chtf8				Arhgap29	Nrp1	Mrpl17	Hipk2	Gm6205		Syvn1	Sall1	Gm10295		Whsc1	Rab22a
Cabyr	Arl5b		Rhbdf2				Fat3	Kat6b	Cux1	Ppt2	Gm6367		Fhl4	Nfe2l2	Med13		Atad2b	Srd5a2
Ankrd45	Zkscan7		Qrfp				Evi5l	Spock2	Vangl1	Syt4	Fam168a		Chmp6	Stat5b	Scyl2		Ssh2	2210018M11Rik
Kmt2a	Adamts20		Cs				Zic2	Lmbrd1	Nbea	Kmt2c	Sgpp2		Avl9	Phf21a	Rsf1		Prkcz	Iqsec2
Adcy1	Lgi2		5430435G22Rik				Brwd3	Fmn1	Arhgap21	Phlpp1	Ndnf		Abcb7	Mboat2	Sobp		Tnk2	Nxf1
Nde1	Mapkbp1		Bdkrb2				Ptp4a1	Ptgfrn	Arc	Kat6b	Otud7b		Pkia	Ppp2r2c	Prdm5		Pex5	Atxn7l3
Fam49b	Ccdc88a		Bbc3				St3gal6	Dstyk	Sox12	Frmd6	Lpp		Mex3b	Id4	Arfgap2		Zc3h15	Snx27
Pik3r1	Kbtbd8		Lrrc38				Syne2	Ddx5	Enc1	Slc25a3	Fam160a2		Mga	Glis3	Nr4a3		N4bp1	Unc13b
Gga3	Zhx3		Hoxc13				Jazf1	FMN1	Socs3	Khdrbs1	Smtnl2		Sox6	Fxr1	Csnk1g1		Rasef	Plekhm1
Mllt6	Xkr7		Hnrnpf				Smcr8	Arhgap5	Syt11	Slc35d1	Cep170b		Syde1	Nsun3	D630044L22Rik		Zbtb41	Fzd3
Celf2	Zfp36		6030445D17Rik				Eif4ebp2	Tspan14	Nfia	9330159F19Rik	Thrb		Fign	Zeb1	Rimbp2		Rnf24	Sirpa
Smoc1	Frem1		Atg12				Shox2	Slain1	Ankrd29	Ptp4a1	Snx27		Fli1	Dhx15	Mark2		Pik3ap1	Fam83g
Cisd2	Ccdc85a		Ace3				Grm5	Myt1l	Btbd7	Proser2	Galnt6		Gpatch8	Arhgap5	Ccdc14		Dock9	Anks1
Cxxc5	Bdh1		Gkn3				Ryk	Cul2	Tnfrsf12a	Elmod1	Tmx2		Nfya	Luc7l3	Gm28048		Brpf3	Kif21b
Ccser2	Kcnc3		Jade3				Yipf4	Maml1	Tgm3	Akap6	AU040320		Xiap	Sec24b	Fign		Mfap3	Rcor1
Rhog	Dennd6a		Ccrn4l				Cacna1c	Homer1	Ptprd	Tmed9	Brwd3		Stk10	Slitrk5	Ptch1		Zfp704	Ssh1
Pde11a	Stx16		Acox1				Prrt3	Rps6ka5	Ankib1	Elavl2	Tmub1		Tln2	Satb1	Nufip2		St8sia1	Gtf2ird1
Ddit4	Igf1		Cers4				Rnf121	Degs1	Zfyve9	Atl1	Rap2a		Fscn1	Stx16	9330151L19Rik		Map3k2	Slc30a4
Tspan3	Pcgf3		Pdap1				Morf4l2	Csmd3	Adipor2	Pcgf3	Crocc		Cyp1b1	Dcun1d5	Xpr1		Grip1	Vash1
Ddx42	Hnf4g		Spta1				Tm9sf4	Vps13a	Syt6	Tnpo1	Sepsecs		Trim44	Cdh2	Egr1		Gan	Plekha5
Eml4	Plekha8		Hif1an				Col13a1	Ep300	Zfp661	Bnc2	Plcxd2		Gnai3	Zbtb7a	Ythdf3		Stk19	Gprin3
Fgf16	Atxn1		Pou4f1				Slc44a2	Tle4	Grk6	Rdx	Smg6		Eif5a2	Fzd5	Bnc2		Chmp2b	Dhx38
AI597479	Nap1l3		Tmem127				Kctd16	Fam155a	Mphosph9	Sox6	Lrrc20		Ammecr1l	Slc25a51	Eml4		Deptor	Steap2
Ebf1	Ccnyl1		Nudcd3				Fyn	Paip1	Bambi	Esrp1	Ttl		Fubp1	Lhx6	Lrrc4c		Abhd13	E130309D14Rik
Ociad1	Btg2		Dopey2				Slc22a23	Igf1	Mtcl1	Ttr	Trim30a		Ppp4r2	Gpm6b	Acvr2a		Eif3j2	Alkbh8
Chsy3	Ppm1d		Podxl				Pak1	Ssh2	Adamtsl3	Osbpl11	Glis2		Pura	Isca2	Ccnd2		Eea1	Adamts12
Socs7	Pde3a		Arhgef19				Igsf3	Kbtbd12	Fkbp1b	Ank2	Ccser2		Tmod3	Zfand5	Dgkh		Mllt1	Pappa2
Zfp740	Isl1		Smim9				Luzp1	H3f3a	Pou4f1	Mal2	Serpina11		Xpnpep3	Arhgap12	Nckap1		Abhd2	Slc22a23
Bmp5	March1		Slc37a3				Creb3l2	Nhlh2	Chst1	Tmem18	Slx4		Tmem170b	Smurf2	Unc5c		Mmd	AI118078
Pnoc	Fam136b-ps		Fam92a				Adam23	Acap2	Fibin	Zfp516	Ndst1		Myt1	Mtfr1l	Naa30		Kif19a	Chdh
Srr	Htr1b		Cacna1i				Foxp2	Ccdc171	Casz1	4921524J17Rik	Jade1		Pcmtd1	Gca	Rap2c		Slc23a2	Pex5l
Ins2	Prr14l		Crb3				Chmp2b	Pcdha10	Hprt	Tmem168	Akap13		Pum2	Hnrnpa0	Cpeb2		Bcl2	Phc2
Ireb2	Rnf39		Fam126b				Braf	Soga1	Snx29	Pcnxl4	Mdk		Wdfy3	Msi2	Pgrmc2		Samd4	Cdc42ep4
Stc2	Fras1		Kcna7				Zfp609	Ahr	Slc52a2	Src	Tmem132b		Ap4e1	Gatad1	Sncb		Phf13	Hif1an
Wnt9a	Itga6		Mrps18c				Zmynd19	Magi2	Rnf111	Evi5l	Man1b1		Dek	Ptprd	Nck2		Pld1	Tspyl4
Esrp1	Vps13a		Mtmr1				Homer1	Clcn3	Hip1	Kcnj2	Ranbp10		Gatsl2	Kdm2b	Abhd13		Etv3	Slc9a1
Api5	Tpk1		Wnk2				Trim71	Ankrd17	Ubxn2b	Sema6a	Zfp866		Clasp2	Ppp3r1	Zfp207		Tlr4	F2rl3
Igf1	Brwd1		Mill1				Atg9a	Nhlrc2	Phf20	Ankrd44	Erc1		AI597479	Kcmf1	B3gnt2		Slc25a51	Creb3l2
Dgki	Bcl2l2		Arl5c				Zfand5	Acot12	Arfip1	Stat5b	Rab40b		Mknk1	Limk2	Eny2		Flot1	Ninl
Ggnbp2	Slc16a2		Nr2f2				Grin2b	Otx2	Dcbld2	Dcun1d3	Ubxn10		Polk	Six5	Ctsb		Paip2	Palm
1700052N19Rik	2210018M11Rik		Arfrp1				Fam81a	Mlh3	Emx2	Fbxl2	Igf2		Bap1	Slc44a1	Rhob		Pou2f1	Sprn
Suz12	Gtpbp2		Elk1				Magi3	Med13	Otud1	Gpr137c	Sema4g		Ptpn13	Snx29	Scn2a1		Amph	Gm4767
Acbd3	Appbp2		Gm5814				Wdr82	Opn1sw	Csnk1g1	Egr2	Gm14326		Hdhd2	Gjb1	Prr13		Pcmtd1	Gm10778
Usp47	Kcnc2		Nnat				Brinp2	Ubxn7	Rbms1	Med13l	Kcnc3		Nrbp1	Pfn2	Bmi1		Cd164	Kcnb1
Pex13	Naa40		Nf2				Fbxo41	Hnrnph3	Fam160a1	Tns3	Nfat5		Ppp1r18	Dusp10	Khdrbs2		Prkce	Cdc42bpb
Pcgf5	Col4a4		E330021D16Rik				Rab35	Hs2st1	Trim33	Bhlhe40	Ntrk3		Gdi2	Fam78b	4932411E22Rik		Rnf208	4930427A07Rik
Pcdh17	Fam134c		Cnot4				Igdcc3	Ipmk	Ikzf1	Sox17	1700008I05Rik		Srsf1	Set	Pnoc		Gpr124	Pag1
Atp6v1a	Klhl9		Arl14epl				Nat8l	Plekha3	Gda	Ifrd1	Wdr82		Ank1	Rffl	Kank1		Rnf144b	Col23a1
Adarb1	Sypl2		Sdr42e1				Dmxl1	Tcf12	Kctd16	Tshz3	Tmem42		Spata2	Fbrs	Pknox2		Nova2	Pcgf3
Bag4	Dynlt1c		Mrfap1				Dennd2c	Arid1b	Nfic	Arhgap24	Gstz1		Ganc	Eri1	Alyref		Chl1	D10Jhu81e
Klf13	Lif		Tnfrsf8				Smad7	Golim4	E2f8	Wapal	Clic5		B3gnt2	Arhgap24	Heatr1		Egr3	C1qtnf1
Zbtb10	Angptl4		Lasp1				Ago2	Capn10	Adnp	Igf2	Bach2		Gm28048	Gigyf1	Hlf		C77370	Rab6b
Egr1	Kctd5		Scn2b				Reck	Eif5b	Armc8	Esrrg	Fam120c		Myb	Vapa	Cand1		Rsu1	Cdk12
Itga1	Proser1		Dnm2				Phactr4	Pibf1	Ino80	Esr2	Ensa		Kcnb1	Ubr5	Bcl6		Rabgap1	Klhl32
Vgll4	Slc5a8		Mipol1				Lmtk2	Cog3	Fignl2	Gm28048	Tcf24		Efnb2	Cep57	Rab21		Ddx6	Mylk2
Trim17	Mlf1		Adra1b				Cdc42bpb	Klf10	Nmt2	B3gnt2	Fndc7		Nek9	Sobp	Strbp		Plet1	Grk1
Gfpt2	Luzp1		Dnajb2				Ldb2	Fam126a	Col19a1	Mapre2	Ccdc85c		Supt20	Lhx1	Gm16039		Pard3b	Cyb5b
Rps6ka6	Entpd7		Tex24				Otud6b	Pvrl3	Rbm46	Egfr	Lima1		Elmod1	Mfsd2a	Sdpr		Ppp4r2	Gbp11
Pgrmc2	Sh3rf3		Clcn2				Hn1l	Bbx	Lnpep	Klhl18	Exoc6b		Hmgb3	Camk2g	Tox4		Bmp2k	Rfx7
Csnk1a1	Cspg4		Kif19a				Fam171a1	Nck1	Ptpn14	Cdc25a	Mei1		St3gal6	Prickle1	Mkl2		Clcn5	Sft2d2
Zbtb43	Nckap5l		Rgs9bp				Coro1c	Arhgef40	D630044L22Rik	Vcan	Rab3b		Ywhab	Tbpl1	Ankrd28		B630005N14Rik	Ppm1f
Mapre1	Gpam		Tnfrsf19				Rapgef5	Med17	Mb21d2	Srek1	Kcna1		Plxna2	Arid4a	Celf6		Slc35d1	Megf8
2010111I01Rik	Mxd4		Pdx1				Arid5b	Sort1	B3galnt2	AW554918	Ick		Cdyl	Fam160b1	Snx1		Ubn2	Cdk5rap2
Atad2b	Jarid2		Aatk				CTNND1	Hdlbp	Dnajb1	Slc17a6	Tenm4		Ddx26b	Gm3646	Fem1c		Evi5l	Arrb1
Myo5b	Gabpb2		Pim1				Ctnnd1	Sh3rf3	Sbf2	Lrrc57	Kcns1		Ncs1	Prr7	Srsf11		Tns1	Nynrin
Tmem170	Dbt		March9				Slc35a1	Bmp2	Fbxo8	Faxc	Pnma3		Pan3	Otp	Ntng1		Epm2a	Fbxl20
Kpna6	Fscn1		Gfpt1				Hspa2	Alkbh5	Hipk1	Fam160a2	Akr1d1		Napb	Eif4e3	Ccdc176		Dixdc1	Gm10032
Nap1l1	Mief1		D17Wsu92e				Foxo4	Glra2	Arid5b	Cdk8	Spred3		Ccny	Samd4	Ocln		Smim13	Tsnax
Zbtb18	Lamc1		Pcdh1				Zfp697	Magi3	B230219D22Rik	Brd3	Kcnd1		Nalcn	Gm9887	Fgf9		Gins3	Endod1
Kcns2	Ccnj		Sema7a				Nckap1	Ikzf2	F3	Syncrip	Snx21		Ago3	Rab3b	Eif4a2		Eif5	Cntn5
Hoxa9	Rapgefl1		Icam1				Kif5a	Casp9	Wdr45b	Msantd2	Gucy2e		Dpy19l1	Scaf11	Safb		Tox3	Itga9
Cys1	Ctdspl2		Parn				Grid2	Shc4	Dnal1	Fam84b	Btbd9		Dyrk2	Kmt2e	Msi2		Fam78a	Baz1b
Milr1	Smurf2		Sfxn3				Erc2	Npas4	Cacul1	Sipa1l2	Glt8d2		Vldlr	Arf4	Map4k5		Rev1	Irs1
Trim11	Slc30a10		C330021F23Rik				Slc38a4	Slk	Socs1	Adpgk	Ablim1		Frmd4a	Smurf1	Tiam1		Zfp36	Nav2
Phax	Mga		Stard4				Fmr1	Eya4	Pnrc1	Dek	Slc25a27		Ube2d1	Sox17	Erp44		Mapk1	Kdm4b
Calu	Cbx2		Ctsd				Gnaq	Avpr1a	Wbp1l	Rtn4rl1	Spidr		C78339	Wars2	Gnb1		Prdm16	Trp53i11
Gnas	Prrc2c		Tnk2				Prosc	Ccdc85a	Hecw2	Fam168a	Kif3c		Mmgt2	Sema6a	Snx13		Cd47	Sec14l1
Ncf2	Rab15		Mgst2				Mief2	St8sia2	Ldoc1	G2e3	Cyp1b1		Zic3	Fgf10	Prkca		Ywhag	Grap2

miR-199-3p	miR-29-3p	miR-486-5p	miR-361-3p	miR-122-5p	miR-425-5p	miR-136-5p	miR-96-5p	miR-142-5p	miR-19-3p	miR-141-3p	miR-770-3p	miR-335-5p	miR-200-3p	miR-194-5p	miR-183-5p	miR-341-3p	miR-182-5p	miR-370-3p
Mkrn1	Camk2g		C2cd2				Brms1l	Btaf1	Patl1	Bap1	Prex2		Dusp1	Phax	Vps37a		Stard13	Rreb1
Nol7	Ppm1e		Rnase10				Foxk2	Fam219b	Aftph	Wdr82	Apc		Glcci1	Gm16039	Pcif1		Gm9938	Larp4b
Prdm16	Arrdc4		Abcg3				Foxf2	Becn1	Rsbn1l	Wdr26	AI661453		Sox1	Bmp1	Ppp1r14b		B4galt6	Neu4
Ppp2r3c	Ap4e1		Spop				Bsdc1	Pou3f1	Zhx3	Nrp1	Src		Ell2	Psme3	Hn1		Slit3	Trabd2b
Kcmf1	Phactr2		RP23-133G16.3				Vangl2	Sacs	Stam2	Epn1	Ctdspl		Arl14epl	Clasp1	Nefl		Camkk2	Axin1
Eno4	Hinfp		Dffb				Filip1	Tcf4	Ablim1	Plekha8	Tlr9		Lpin1	Fgfr1	Kctd15		Xkr4	Mafg
Sall3	Mtss1l		Cdc42se1				Anxa11	Bche	Map3k1	Nfia	Pla2g2e		Map4k3	Dnajc1	Rufy2		Scn2a1	Adarb2
Csnk1d	Pitpnm2		Apold1				Gabrb1	Enpp5	Memo1	Lsamp	Art4		BC005537	Tmem5	Tmem170		Wasl	Fam189a1
Sgcg	Ago1		Atxn2l				Ppp3ca	Itpripl2	Rnf38	St3gal5	Hnf4a		Fam76b	Exoc6b	Arpp19		Sp8	Prdm4
Anxa13	Morc2a		Trim67				Kdelr2	Egr3	Atg14	Sesn2	Lrp6		Nup153	Mrpl35	Irx2		Opn3	Dcp1a
Timm22	Emid1		4933421I07Rik				Vldlr	Rps6ka3	Mtus1	Tnik	Dnhd1		Ets1	Foxf1	Htr2a		Gyk	Cbfa2t2
Mmp16	Kpna4		Npy1r				Olfr78	Myf5	Gtf2a1	Serpinh1	Nacc1		Bicd2	Prss12	Fbxl2		Ncald	Slc9a8
Khdrbs3	Cnr1		Hsd17b11				Unc5d	Stt3b	Atp11a	Kif3a	Nfkbib		Fzd3	Nucks1	Sar1a		Kpna3	Ago2
Twist1	Rap1gds1		Gpr161				Slc22a21	Clock	Sulf1	Trim2	Htr5b		Klhl3	Atp5j	Cetn2		Tctn3	Zfp366
Otud7a	Med12l		Fblim1				Ext1	Rps6ka4	Fam124a	Samd8	Prdm11		Ccser1	Tmed10	Palm2		Hoxa10	A530064D06Rik
Tax1bp1	Emp1		Fam219b				Mapk9	Cast	Trim67	Has2	Cdc27		Ptchd4	Tmem242	Caln1		Tex2	Pla2g2e
Slamf7	Adamts15		Styxl1				Slc1a2	Pdgfra	Dlg1	Khdrbs3	Zfp65		Gnaq	Swap70	Grin2a		Kif5a	Adrbk2
Ndufb9	Gsk3b		Diap2				Phf21b	Bhlhe41	Mmgt1	Kdm4a	Tlr4		6430573F11Rik	Car5b	Kif2a		Wwc2	Aplnr
	Hook3		Bcl2l13				Pou3f2	Srebf1	Kpna6	Yy1	Xiap		Rqcd1	Exoc7	Cenpm		Gabrb1	Itga4
	Gm16515		Sfrp1				Ctdsp1	Ebf3	Inhba	Tnks2	Wscd2		Eif5b	Pgm3	Smim14		Rab9b	Mx1
	Prkg1		Mapk8ip1				Amer2	Zfp462	Pex5l	Foxa1	Wdr54		Gm9925	Cul4b	Vmac		Lcor	Rnf169
	Kdelc1		Foxi1				Unkl	Itga6	Zfp516	Clasp2	Cyp7a1		Piga	Gdf11	Slc39a5		Tspan4	Amotl1
	Rmnd5a		Lhfpl4				Cobl	Gng13	Kcna2	Ppp4r2	Adap1		Thap1	F11	Faf2		Pcmt1	Fbxo41
	Bsnd		Lman1l				Tacc1	Ptprb	Rab2b	Pou4f1	St3gal3		St13	Lyrm5	Ttc19		Gm28048	Kcnk6
	Phlpp2		Telo2				Lamc1	Lbr	Ago1	Spire1	4933427D14Rik		Jazf1	Capzb	Pgm3		Taf4b	Mthfr
	Ddi2		D10Wsu102e				Mtmr12	Dram2	Pcnxl2	Rag1	Rxra		Reck	Tusc3	Zfp142		Myrip	Sec14l3
	Armc8		Eif4ebp2				Ogt	Syt7	Npas2	Qser1	Shc3		Slitrk1	Kcnj11	Aktip		Myo19	Nmnat2
	Phc1		Psme4				Rnf130	Csnk1g1	Ddhd2	Gli2	Spn		Bmi1	Snx10	Hoxb4		Hdac9	Mafk
	Gm20721		Fam212b				F13a1	Etfdh	Tmbim6	Hoxc13	Tom1l2		Prrc2c	Gipc1	Paip2		Evx2	Smad5
	Gpcpd1		Rgn				Qk	Scyl3	Chd2	Kpna3	Adipor2		Yipf5	Aven	Leap2		Fbxw7	Rgs3
	Serinc5		Cbx6				Foxn3	Ginm1	Enpp5	Nipal3	Zswim4		Foxf1	Txndc5	Pigx		Arel1	Pgm5
	Dpp3		Gpr157				Mdm4	Wnt2b	Nek9	Gab1	Tchp		Xkr6	Dll4	Tmem53		Map1lc3b	N4bp1
	Tfec		Adad2				Ptpn9	Rb1cc1	Cntfr	Bcap29	Wdr52		Phf6	Adck2	Pink1		Klhl7	Ago1
	Ammecr1		Col9a2				Plcb4	Cbx3	Arrdc4	Ogt	Tbc1d22a		Ppara	Rnf130			Pygo2	Sik3
	Tmem132d		Tuft1				Rab27a	Fam168a	Jph3	Rc3h2	Tcf23		Tmx4	Nell2			Grem1	Soga1
	Slc35b2		Armc9				Plod2	Yy1	Tmem139	Naa60	Snx30		Fubp3	Prorsd1			Tnrc6a	Mgme1
	Lasp1		Dnajb12				Fibcd1	Ccnt2	Smad5	Ptprz1	Map3k12		Tnfrsf11b	Otud7a			Kcnj2	Urb2
	Zbtb10		Dda1				Gdnf	Sun1	Fbxo48	Kif21b	Armcx3		Inpp4a	Ndufb9			Zhx2	Zfp825
	Tubb2a		Yap1				Cables1	Pou3f3	Cs	Lmo3	Gm4631		Chd2	Cdk5rap3			D15Ertd621e	Sstr5
	Srcrb4d		Josd1				Dcun1d3	Zbtb46	Mib1	Cdc14a	Cldn2		Ppp6r3	Amigo1			Yes1	Stpg1
	Trim37		Ptp4a2				Tll1	9430038I01Rik	Llgl2	Sft2d2	Rcbtb2		Pcdh19				Fam149b	Trim33
	Ing4		Socs3				Rassf8	Toe1	Pik3cb	Spred2	BC003331		Acyp1				Syne2	Camk1d
	Syt14		Zfhx2				Cbx6	Hiat1	Ythdf2	Six1	Slc6a6		Kmt2c				Fbxo42	Plxna4
	Aplnr		Cdkn1a				Myb	Nek4	Tsc1	Sox11	Tc2n		Xkr8				Rsf1	Plch1
	Erc1		2610507B11Rik				Fam118a	Ric8b	Zfp711	Rims1	Lrrc15		Nr5a2				Rtn4	Dot1l
	Spast		Stx16				Cacna2d1	Ptprd	Mical3	Arhgap23	Gm14308		Usp31				Dcx	Tom1l2
	Atg9a		Efcab5				Sept11	Osbpl6	S1pr1	Ccdc80	Slc24a2		Ptpn12				Atxn1	Dlg5
	Gng12		Arsi				Wdr26	Gucy1b3	Bhlhe23	Pphln1	2700081O15Rik		Zdhhc17				Tgfbi	Nsg2
	Cemip		Klhl22				Trim46	Ccne2	Kcnq2	Cyb5a	H2-Q4		Trim2				Has2	Trim16
	Homer1		1700016D06Rik				Celf1	Trim36	Stam	D16Ertd472e	Acly		Sptssa				Arhgdia	Map4k4
	Dcaf10		Hoxc8				Trim37	Dnajc7	Hipk3	Wibg	Irgq		Mex3c				Aatk	Slc15a4
	G6pc		Zfp428				Paqr8	Nr3c1	Lrp11	Tm4sf1	Peg12		Sh3pxd2a				Unc13c	Lrch3
	Ago2		Sipa1l3				Cbx5	Camk2a	Ube2d2a	Zfp618	Xpnpep3		4931406P16Rik				Hspa13	Mxd4
	Upk1b		Wdr63				Neurod4	Cars2	Phlda3	Sept7	Pum1		Wdr82				Brwd3	Prdm15
	Rab30		Sema3f				Nptx1	Asphd1	Bcl7a	Fry	Arpc4		Sik2				Ppm1f	Zfp516
	Fam184b		Atp1b1				Prlr	Stau1	Dcun1d2	Mtpn	Sypl2		Rab37				Soga1	Cdon
	Fzd5		Gm5089				Cadm2	Tbc1d8	Cyb561d1	Gfod1	Vmac		Usp25				Prune2	Antxr1
	Atp2b4		Prkd2				Mycbp2	Zfand3	Ddx6	G3bp2	Zfp334		Git2				Zdhhc15	Gucd1
	Serpinh1		Ak4				Ago1	Kcnh5	Slc26a7	Vezt	Tenm3		Mboat2				Nrcam	Ston2
	Csrnp2		Hic1				Phf10	Setd1a	Wdr44	Itpripl2	Kcnj14		Ncoa7				Larp4	Casz1
	Kdm5c		Eef2k				Zfc3h1	Stx11	Cdc42bpa	Wdr52	Slc2a8		Taf4a				Tbr1	Rap1gap2
	Runx1t1		Trim9				Sp3	Strbp	Snx27	Vsig8	Dennd1a		Fermt2				Tnfaip8	1810041L15Rik
	Nfic		Dyrk4				Tmbim6	Qser1	Smarcd2	Ppp2r2a	Nav2		Pbx3				Celsr1	Mn1
	Orai2		Fan1				Ppfibp1	Ccr1	Vcpip1	Eif5	4933402D24Rik		Zbtb33				Zdhhc20	Iqsec3
	Map2k6		Bax				Reep2	Slc6a5	Tspyl4	Gpc2	Fkrp		Snap25				Pnpla2	Apba1
	Zfhx3		Icos				Cbfa2t3	Dnal1	Rhebl1	Kdm2b	B4galt5		Eri1				Slc35b4	Ssh2
	Pik3r1		Tollip				Pappa	Tom1l1	Tgfbr2	Agfg2	Pank3		Map3k13				Tnpo1	Kcns2
	Rps6ka3		Ubtf				Lnx2	Krit1	Vgll4	Lmbr1	Nxph1		Naa50				Reck	Arhgap26
	Nfib		Bace1				Sp7	Rhot1	Ar	Plxna4	Pomgnt1		Rhot1				Jmjd1c	Amfr
	Tmem136		Kcnj1				Celsr2	Alcam	Foxp1	Lrp1b	Btf3l4		Chd9				B3gnt2	Nceh1
	Cpsf7		Cyp2d37-ps				Dnajc30	Pfkp	Zfp91	Lypla1	Sema4b		Acap2				Akap17b	Loxl2
	Palm		Psg16				Snx16	9430020K01Rik	Mybpc1	Exoc5	Picalm		Tbk1				Map2k6	Grin3a
	Kdm6b		H2-Q10				Bnc2	Arid2	Timp2	Zfp608	Chd5		Slc35a2				Wwox	Trim71
	Il1rapl1		Foxm1				Rbm25	Syt2	Bmf	Pcdh8	Gm6484		Fbxo42				Gucy1a2	Rbm33
	Dgkh		Ern2				Xiap	Mapt	Soga1	Frmd4a	Trim11		Hipk2				Add2	Hdac5
	AU019823		Muc1				Rac1	Fam45a	Nhsl2	Unkl	Zfp109		Ap3s1				Sav1	Znfx1
	Trafd1		Bhlha15				Rqcd1	Fbxo3	Zfp521	Cul3	Gas7		Nrbf2				Pde7a	Slc16a7
	Usp42		Klf10				Fem1b	Bod1l	Tnrc6a	Ankrd50	Muc20		Vbp1				Rab19	Ncam1

miR-199-3p	miR-29-3p	miR-486-5p	miR-361-3p	miR-122-5p	miR-425-5p	miR-136-5p	miR-96-5p	miR-142-5p	miR-19-3p	miR-141-3p	miR-770-3p	miR-335-5p	miR-200-3p	miR-194-5p	miR-183-5p	miR-341-3p	miR-182-5p	miR-370-3p
	Tectb		Orai2				Zbtb1	Son	Itsn1	Arpc5	C87414		Plxna4				Kdm2b	Fam131b
	Rnf169		Scamp2				Lcor	Npc1	Csf2rb2	Cdv3	Xkr7		Adcy9				Gja3	Skp2
	Mfap5		Galr2				Csad	Sh3kbp1	Daam1	Adrb1	Galc		Fbxw7				Aadat	Rab40c
	Ankrd49		Unc45a				Tiam1	Kcnb1	Mprip	Hipk3	Luzp1		Khdrbs1				Uevld	Sec31b
	Cdc7		Ccr9				Ppp1r9b	Ube2e3	Cul5	Zcchc3	1700034I23Rik		Gata4				Creb1	Kif3b
	Lpl		Gm5423				Fndc3b	Myo1d	Ttyh3	Chd9	Rab1b		Suv420h1				Slc43a2	A430105I19Rik
	Uhrf1bp1l		Slc6a8				Atp6v1c1	Atp2b4	Vps4b	Enoph1	Stxbp5l		Golim4				Sox2	Rorb
	Cmpk1		Slc47a1				Rab21	Ptpre	Zeb2	Cckar	Slc17a9		Cep41				Ppp3r1	Extl3
	Lemd2		Sephs1				Slc7a8	Col4a3	Map3k12	Bahd1	Ppara		Fam107b				Cyb561	Lzts1
	Akap13		Tfap4				Rps6ka6	Shc3	Smoc1	Irf2bpl	Rrad		Pcnx				Trp53inp2	Hs3st3b1
	Eif3j1		Nyap1				Zdhhc17	Cdk17	Lphn2	Rnf165	Olfml2a		Fam179b				Htr1b	Veph1
	Hapln1		Parm1				Fut4	Btbd1	Asap2	Dixdc1	Cpne3		Dact1				Cacna2d2	Gm14137
	Lepre1		Ppp1r12c				Tln1	Adam12	Arfgef1	Harbi1	Fam53c		Mief1				Dyrk1a	Hcfc1
	Kcna2		Tmem120b				Eif5	Fam134a	Asxl3	Mrrf	Plin3		Ppp1r12b				Adarb2	Psd3
	Sh3bp5l		Vsx1				Bcl2l13	Bbs9	Camta1	Rbm7	Keap1		Plxnc1				Rab3c	Eif3j1
	Shprh		Sp7				Clock	Cps1	Nacc2	Akirin1	Usp20		Ranbp10				Pik3ca	Pttg1ip
	Ubn2		Ly9				Ktn1	Bzw1	Cds1	Sv2a	Unc13a		Foxo3				Bicd1	Uvssa
	Rlim		Ppp2r5b				Maf	Id2	C530008M17Rik	Ppp3r1	Wnk2		Errfi1				1810011O10Rik	Zfp871
	Psme4		Hs3st3b1				Arsj	Cwc25	L3mbtl3	Cfl2	9130011E15Rik		Fam60a				Cdsn	Gabbr1
	Prpf40a		Rgs16				Bcl2l11	Mat2a	Tacc1	Taf12	Col6a2		Arl5a				Actr2	Fam171a1
	C030046E11Rik		Tmem231				Igf1r	Cycs	Mlec	Supt6	Hltf		Golga1				Ank2	Sipa1l2
	N4bp2		Meis2				Dgcr2	Ccdc71l	Wnt1	Itga6	Tcf20		Rnd3				Tmem189	A3galt2
	Nat8l		Hcn3				Stk17b	Flt1	Csf2rb	Bace1	Cdc42ep1		Aim1				Slc4a1	Mtor
	Ina		Cd164l2				Ralgps2	Csnk2a1	Pik3r3	Mtap7d3	Cry2		Mtmr11				Sesn2	Ntng1
	Vash1		Tlx3				Vat1	Pum1	Pdpr	March7	Clp1		Pak3				Mycbp2	Zkscan1
	Tspan14		Ube2s				Uck2	Mob1a	Gramd1b	Npnt	Frmd7		Bcl11b				Slc12a6	Fgf11
	Mog		Raf1				Mtdh	BC030336	Apaf1	Zfp207	Rcor1		Frem2				Snx4	Smarcc1
	Nras		Mast3				Mfsd5	Abhd2	Kcne4	Canx	Nkx1-2		Ubxn7				Hmgcll1	N28178
	Vcl		Rasgrp2				Wasf2	Unkl	Mab21l2	Kctd2	Notum		Sec61a2				Tob1	Lrrc15
	Mlxip		Arl14				Klf3	Clasp1	Gpcpd1	Xpr1	Eme2		E130309F12Rik				Itpr1	Cdh7
	Cdc42bpa		Abca7				Gja3	Gm9966	Ccdc126	Nudt12	Kif3b		Ckap4				Ago2	5430435G22Rik
	Pdgfrb		Wnk3				Csrnp3	Socs1	Vps37a	Cep120	Pnma2		Nxph1				Mpp1	Tmcc3
	Mest		Mterfd2				Nid1	1500011K16Rik	Sec22b	Tcf4	Il12rb1		Mkl2				Tsnax	Zc3h7b
	Trim56		Dusp2				Slco3a1	Pbx1	Dlx3	Ampd2	9530068E07Rik		Nrip1				Fhl1	Bdkrb2
	Zc4h2		Clec4n				Baz1b	Xkr8	Cpd	Son	Mark2		Snx30				Ankrd28	Col19a1
	Fbxo42		4933414I15Rik				Inpp5a	Rock2	Hnrnpul1	Slc1a1	Necab2		Fam178a				Adh6-ps1	Atp10b
	Emp2		Lhx3				Mras	Ulk1	Ubl4	Aff4	Pde4dip		Arih2				B3galt1	Arhgap17
	Klhl42		Tnfrsf18				Ppp1r12c	Tnrc6c	Thsd7a	Cbx5	Rgp1		Dnajc1				Epha3	Bap1
	Sms		Syndig1				Ddah1	Stag1	Bend3	Luc7l3	Olfr1167		Map4k4				Dync1li2	Usp46
	Ldlrad3		Hes7				Nuak1	Lzic	Epn2	Dlg3	Fam160b2		Mdfic				Elavl4	Clstn1
	Ak3		Angptl7				Egr3	Rnf150	Tor1b	Map3k3	Tbc1d14		Prkar2a				Tcf7l2	Golga2
	Reps2		Snn				Rnf217	Sirt7	St8sia5	Plagl2	Plch2		Crybg3				Lhx3	Otud3
	Spata2l		Yipf1				Dscam	Slc17a7	Ccnt2	Atf5	Agpat2		Rtf1				Nufip2	Rnd3
	Tbc1d13		Pvrl4				Usp13	Ipo7	Cyld	Tra2b	Nov		Cbx5				Insig1	Fam126a
	Zfp704		Gm5422				Tenm4	Slc25a27	Thbs1	Stk35	Ankrd11		Sdk2				Zfp280b	Sbf2
	Trim63		Fbln5				Aifm3	Bicc1	Ccnl1	Snx9	Atp2b2		Zfp148				Bod1l	Ddx39
	9530068E07Rik		Rassf3				Gpr39	Irf9	Bcas3	Plxnc1	Wnt9a		Nudt4				Hand1	Nlgn3
	Wdfy1		Gnb2				Shc4	Brd1	Med26	Krt222	Apba1		Pak7				Dock1	Anks6
	Slk		Ogt				D10Bwg1379e	Ythdf3	Fam46c	Fus	Sept12		Ppp1r10				Luzp1	Cradd
	Celf2		Gtpbp8				Trabd2b	Stk3	Acbd3	Synpo2	Efr3a		Csrnp3				Vat1l	Gne
	Mmp2		Acads				Cacna1b	Mycn	Slc22a23	Rap2c	Fam227a		Cetn4				Daam1	Ppm1l
	B3galt1		Rab3d				Med12l	Lypla1	Dnaja2	Rpgr	Pappa2		Hspa9				Mecom	Ubap2
	Daam1		Mrpl50				Shc1	Egln3	Dgcr8	Mdm1	Cep41		Ppp4r1				Hecw2	Ppp1r3e
	Mast4		Hoxc6				Rap1gap2	Igsf10	Sphk2	Megf9	Cdh5		Bag5				Snap23	Sptb
	Cops7b		Kremen1				Rsf1	Rnf217	Prc1	Zfp292	Wdr11		Ripk2				Cbx7	Dtymk
	Kirrel		Prps2				Sp8	Fzd7	4930402H24Rik	Mapk6	Cmah		Atf2				Dazap2	Tmem28
	Syt2		Mtch1				Plekha8	Has3	Itga6	Elk3	Fbxl18		Tmcc1				Grm5	Cul5
	Golga7		Kcnj11				Naa50	Dido1	Gm9979	Gja1	Rasd2		Etv5				Kdelr1	Snx30
	Nfatc3		Wnt10b				Hdac7	Diap3	Rnf165	Myo9a	Fermt1		Gigyf1				Denr	Bmp2k
	Rere		Cplx2				Plch1	Tnfsf9	Rap2b	Slc10a7	Pitpna		Dcaf7				Gorasp1	Scube1
	Ddx3y		Pcbd1				Nptx2	Flrt2	Wnt3	Pxk	B3galt1		Hnrnpk				Gatsl2	Pmm2
	Plxna1		Rab3a				Scyl3	Mtus1	Sel1l3	Ypel5	Fndc8		Fam120c				Cep170b	Ezh1
	Agtr2		Wasf2				Gm28049	Slc22a23	Zfp217	D15Ertd621e	Wfdc15b		Gpr27				Reps2	Nt5e
	Aqp4		Adcy2				Xkr7	Fam189a1	Lif	Cntn1	Fktn		Rhoa				Prepl	Ehf
	Hpcal4		Rtn4rl2				Wipi2	Celsr1	Tm6sf1	Ifngr2	9930111J21Rik2		Myzap				Fyttd1	Adcy7
	Osbp		Mob3b				Cnn3	Sgce	Slc25a20	Apba2	Foxk1		Kctd15				Cdk12	Erv3
	Raph1		Gm4477				Iqsec1	Emc4	Mapk1	Pcf11	Crat		Rdh10				Nsd1	Nhsl2
	Usp31		Cdh23				Thbs2	Krt34	Rhob	Tenm3	Myrf		Smarcad1				Cers2	Prex2
	Chfr		Edn3				Rims4	Usp15	Kcnk2	Prickle1	Syngap1		Cpd				Stag1	Kirrel
	N4bp2l1		Abo				Mctp2	Txn2	Cast	Rnf11	Cr2		Papd5				Myadm	Ralgps2
	Fam168b		Emd				Fam53c	Rhoc	Cecr2	Asxl1	Rnf165		Gabarapl2				Rcor1	Dusp8
	Tmem183a		BC017643				Rufy2	Spag1	Runx1t1	Acot7	Scnn1g		Dnajb5				Ccnj	Gmeb2
	Mtx3		Tmem80				Med14	Ppl	Rai2	Fkbp5	Foxa1		Fxr2				Tiam1	Spib
	Arvcf		Tmem165				Mkl2	Cacng7	Gatad2b	Rnf13	Brsk1		Phf21a				Wdr47	Tmem263
	Mblac2		Syde1				Slc25a42	Mapk6	Akap7	Lgi2	Galnt1		Rsrc2				Atxn1l	Fam124a
	Elmo2		Rab5c				Strbp	Pbx3	Wdr26	Phyhipl	N4bp1		Cnot4				Myo1b	Dnajc27
	Wbp1l		Agpat1				Nanos1	Fam129b	Ano3	Stxbp1	Plac9b		Kank1				Spen	Bptf
	Strn4		E030010N08Rik				Pcnx	March6	B630005N14Rik	Cnot6	Plac9a		Klf10				Sowahb	Gucy2e

miR-199-3p	miR-29-3p	miR-486-5p	miR-361-3p	miR-122-5p	miR-425-5p	miR-136-5p	miR-96-5p	miR-142-5p	miR-19-3p	miR-141-3p	miR-770-3p	miR-335-5p	miR-200-3p	miR-194-5p	miR-183-5p	miR-341-3p	miR-182-5p	miR-370-3p
	Wac		Cacna2d2				Mfap3	Cblb	Tmem159	Rbm33	Gm14391		Sash1				Zbtb44	Atp2b2
	Gab1		Wtap				Csnk1d	Eif4e3	Fam69a	Cacna1b	Slc6a7		Pten				Grik3	Nlrc5
	Stx17		Rmdn3				Fam168a	Sept7	Dhx40	Cacna2d1	Gm12184		Akt3				Cebpa	Chrm3
	Hmgn3		Ngfr				Arrdc3	Taf4a	Mbnl3	Lhx6	Ptges2		Shroom1				Acer2	Pgbd5
	5430427O19Rik		Slc35e4				Rasa1	Nr4a3	Bend4	Rhobtb1	Rnf141		Secisbp2l				Cul5	Pvrl1
	Zbtb41		Prss56				Gprasp2	Il18	Kpna4	Hnrnpf	Slc52a2		Hsph1				Sh3kbp1	Tet2
	Xkr4		Pirt				Atxn1l	Ing3	Dyrk2	Celf6	Ttpal		Mgat3				Gm10032	Lars2
	Snx4		Arfip2				Sppl2a	Ube3c	Lrch1	Vwc2l	Igsf3		Fmr1				Csrnp3	Dnajc16
	Sppl2b		Prkcz				Ago3	Aph1a	Ppp6r2	Atp7b	Vldlr		Slc10a7				Ankib1	Srp72
	FMN1		Mep1a				Bod1l	Onecut1	Ccdc66	Jade1	Chrna2		Spred1				Atoh8	Trrap
	Ccdc117		Sez6l				Camsap2	Msh6	Mbd6	Spock2	Tspan4		Cdr2				Prpf4b	H6pd
	Syn3		Mgll				Fgf9	Scoc	Uhmk1	Pitpnm3	Mmab		Snrpb2				Farp1	Rc3h2
	Mtmr4		Atp6v0e2				Rabgap1	4933406J08Rik	Shank2	Foxp2	Anks3		Ssh2				0610030E20Rik	Prdm11
	Arnt		Tfcp2l1				Bicd1	Ssbp2	Rnf19a	Gpr12	Cdk2ap1		Ulk2				Ndrg1	Ubn2
	Ubfd1		Nfkbib				Stard7	Kcna4	Ereg	Tns1	Gpr146		Rev1				Ccnd2	Slc4a4
	Zmiz1		Fam71f1				Pggt1b	Hspa4l	Mbnl1	Xkr7	Prrg3		Btrc				Kcmf1	Foxo1
	Fmn1		Olfr536				Pdzd8	Aff4	Atrx	Itsn1	Carhsp1		Nav3				Phldb1	Cit
	Kcnh7		L3mbtl3				Tbx15	Myo1c	Rad51d	Zbtb46	Fam107b		Syt1				Klf7	Zc3h15
	Fam83f		Cacng6				Foxo3	Usp46	Pla2g10	Wipf1	Wdr83os		Nipbl				Kctd7	Epb4.1l4b
	Prrt4		Epn1				Gpm6a	Rnh1	Slc6a5	Nab1	Actr1a		Slc35e2				Lrch2	Megf9
	Pik3r3		Lrrc1				Creb1	Jmjd1c	Atp2c1	Mynn	Hspa13		Rab21				Gcnt4	Phldb1
	Xylt1		Hcn4				Gyk	Gm527	4930579G24Rik	Nudcd1	Hif1an		Ppap2b				Nkx2-2	Gm19684
	Hspg2		Lpgat1				Abl1	Epn2	Arhgap12	C87436	Fam126b		BC037034				Rere	Sec16a
	Snx22		Ier3				Taf4b	Mnat1	Zbtb18	Psmd11	Gm14418		Mex3a				Col5a1	Gna13
	D430041D05Rik		Clcn1				Dcx	Lrp8	Desi2	Ccdc117	Gm14322		Taf4b				Trio	Glyctk
	Nkrf		March4				Etl4	Kpna4	Spg20	Zfp36	1110002E22Rik		Arhgap6				Zadh2	Gpatch8
	Adcyap1r1		Eif4a3				Tnik	Ctnnb1	Tmem151b	Asxl3	Tmcc1		Ngef				Adamts18	Exoc6b
	Sgk1		Cmbl				Lphn2	Syncrip	Mctp1	Mob3b	Pou4f1		Snph				Wipf1	Leng8
	Cpeb3		Tssk2				Dbx1	Mfsd9	Spryd3	H2afz	Tmem178b		Plekhm3				Efnb2	Oas1a
	Hnrnpf		Vwa7				Mak	Rad9b	Nufip2	Atp1b1	Nf2		Grem2				Adam10	Zer1
	Zfyve26		Slc13a3				Zcchc14	Tnik	Mkl2	Tet1	Arnt2		Arhgef4				Oxsr1	Rpp14
	Mob1a		Snx21				Slc33a1	Mgll	Grsf1	Ywhaz	Gabbr2		Gls				Taf4a	Ttll6
	Vamp3		Tmem178b				Csrnp1	Crk	Bcl2l11	Parm1	Gm14410		Fstl4				Cdc42se1	Gmps
	4921524J17Rik		Leprel4				Brwd1	Surf4	Mapk6	Ak2	Gm14295		Atp6v0e				Ppp1r2	Ppp1r12b
	Peak1		Klf3				Has2	Kcns3	Fam46b	Svil	6330403A02Rik		Fbxw11				Pafah1b1	Prrc2b
	Srgap2		Prkcq				Spsb1	Jarid2	Plcb1	Sirt1	Nod2		Calu				Tbc1d22a	Zfp92
	Kcnj12		Stra8				Slc35a2	Hmgcll1	Arid4b	Sidt2	Gbp7		Sox2				Unkl	Nktr
	Ccnl2		Rnf214				Trp53inp1	Slc36a1	Cc2d1a	Rabgap1	D430019H16Rik		Ppp2r2c				Asap1	Plch2
	Fermt2		Ddx5				Cpm	Hs3st3a1	Hgf	Rarb	Tnfrsf19		Dag1				Mctp2	Ablim2
	Rsc1a1		Map1a				Clic5	Ppm1g	Kcnc4	Suv39h2	Gan		Rfx3				Syncrip	Papln
	Camk1d		Hspb7				Mllt1	Mospd2	Btg1	Rab38	Taf2		Creb1				Bicd2	Hsdl1
	Ube2h		Hspb2				Mecom	Zcchc2	Mdfic	Serf2	Cd34		Snai2				Scn9a	Kcnmb1
	Onecut2		Fam117b				Clvs1	Trip10	Tia1	Pkn2	Cerk		Cdc40				Atxn7	Foxn3
	Pdgfa		Xpo7				Acvr2a	Edc3	Cnot7	Cep170	Slc25a42		Pcsk5				Adra2c	Tecpr1
	Tiam1		Bmper				Fyttd1	Cdc37l1	Gucd1	Hoxa11	Col4a4		Slc6a6				Chst1	Sobp
	Ralgps1		Diras1				Tmem127	Marveld2	Sowahb	Elf2	Stx5a		Pcdh8				C530008M17Rik	Dlgap1
	Diap2		Prkce				Pdcd6	Rab3gap2	Fgf10	Anp32e	Tmem184b		Mbnl1				Itga3	Kcnab2
	Lpgat1		Cadm4				Stk35	Cit	Snph	Cadm1	Gm12887		Ier5				Cdh20	Dnah11
	Dip2b		Ccdc85c				Arid3a	Sp2	Socs5	Zmat4	1700066M21Rik		Prr11				Gramd1b	Tmppe
	Rnf13		Mrpl1				Basp1	Dlg2	Sdr16c6	Nampt	Aqp1		Hnrnpd				Crtc1	Rnf41
	Uhrf1bp1		Rarg				Cttn	Cntnap2	Ncald	Ptprq	Cmtm3		Ddx1				Igsf10	Ankfy1
	Scamp5		Aup1				Mturn	Lhx9	Lpin2	Wwtr1	Kbtbd4		Atxn1l				Foxp2	D17Wsu92e
	Prickle2		Gm9821				Ccdc88c	Cmtm6	Il1rapl1	Zfp239	Znrf3		Phldb1				Scn3a	Arnt2
	Endou		POU2F1				Fbxo33	Setd3	D230025D16Rik	Rffl	Uhrf2		Bbx				Gria1	Xirp2
	Arhgap28		Syt12				Ppp2r3a	Acsl6	BC030336	Ttpal	Gpc1		Lmo7				L3mbtl1	Arhgef18
	Pitpnm3		Mbtd1				Pcif1	Sgms1	Brwd1	Rhpn2	Sart3		Brsk2				Wnt2b	Sorbs2
	Klf4		Prnp				Azin1	Zbtb34	Tet3	Olig3	Pald1		Trim66				Stx5a	Ncor2
	Abce1		Cd80				Fzd9	St6galnac6	Syn1	Steap2	Shank2		E2f3				Lphn1	Asb1
	Strn3		Nelfb				Igf2bp1	Cmip	Asna1	Pex5	Plekhm3		Arid2				Trim71	Faf2
	Prelp		Cml3				Eif3j1	Pcbp1	Homer1	Cir1	Rabep1		Coro1c				Fosl2	Bzrap1
	Ppp1r12b		Bcas1				Jade2	P2rx4	Parm1	Vps26b	Sned1		Ick				Champ1	Ino80d
	Dcun1d4		Wfdc16				Kcnj14	Cyp26b1	Cntnap5a	Wdr44	Pard3b		Mpdz				Cdc42bpa	Alpl
	Kpna1		Cd82				Ddi2	Actc1	Dcp2	Sugt1	Ttll7		Anln				Triqk	Rassf5
	Zfp652		Sema3g				Dock9	Nfatc3	Vstm2b	Hoxb5	Kctd12		Mospd2				2610018G03Rik	Sin3a
	Slc7a11		Pfkfb3				Aebp2	4931406P16Rik	Map6d1	Rnf38	Ap1m2		S100pbp				Stxbp5	Mmp15
	Epb4.1l4b		Syt7				Epb4.1l4b	Cers6	Rfx4	Nme1	Sh3gl1		Asf1a				Plekhm1	Fbxo32
	Pgp		Sp4				Camkk2	Cir1	Ppp6r1	Kif1b	Cd84		Bnip3l				Pcdh18	Galnt6
	Kctd15		Tspyl2				Cdk12	Sertad4	Rab21	Tram1	Gnao1		Nufip2				Xbp1	Ppp1r12a
	Hs3st3b1		Fstl1				Mbnl2	Appl2	Pon2	Ube2z	Sp1		Dcp2				Fam46d	Tmem229b
	Rcor1		Saa1				Daam1	Bag4	Dcun1d3	Fam188a	Zfyve28		Hnf1b				H6pd	Dclre1a
	Ank3		Runx1				Secisbp2l	Dusp2	Prdm15	Trim32	Cd300lb		Dock9				Fbxo41	Kcnd3
	Rfx5		Camk2a				Plekha7	March7	Jam2	Pex13	Cabin1		Mmd2				Rimbp2	Flrt1
	Fbrs		Ctps				Epas1	Arid1a	Grm4	Ptprd	C330007P06Rik		Spesp1				Mmp16	Map1a
	U2surp		Rbp3				Tspan4	Rcn2	Lrrc16a	Gpr6	Ppp2r4		Gpr158				Wnt4	Rimbp3
	Commd2		Celf5				Greb1	Arhgap42	Abca1	Numbl	Lphn1		Synj2bp				Lmtk3	Brpf3
	Csgalnact2		Mbp				Stmn2	Zfp36	Ubap2l	Rybp	Esrp2		Slc16a2				Far2	Ybey
	Arhgap36		Rnase6				Plxdc2	D16Ertd472e	Flnc	Apold1	Defb42		Phactr3				Rictor	Hhipl1
	Cox10		Gripap1				Slmo2	Fbxo21	Raf1	Csnk1a1	Xrcc2		Basp1				Slc16a9	Nsl1

miR-199-3p	miR-29-3p	miR-486-5p	miR-361-3p	miR-122-5p	miR-425-5p	miR-136-5p	miR-96-5p	miR-142-5p	miR-19-3p	miR-141-3p	miR-770-3p	miR-335-5p	miR-200-3p	miR-194-5p	miR-183-5p	miR-341-3p	miR-182-5p	miR-370-3p
	Thoc2		Zfp773				Gpr137	Ammecr1	Nol4	Nufip2	Adarb1		Kdm3b				Pde7b	Kdm7a
	Pura		Klf13				Zfand4	Mmp24	Cbfb	Mphosph9	Arl5b		Mybl1				Myb	Gse1
	Hiatl1		Rab43				Ppil1	Lhx8	Ucp3	Trim36	Kif21b		Tubb3				Atad5	Tjp1
	Bcorl1		Wnt9a				Mospd2	Cep135	Slc1a2	Satb2	Col23a1		Mtss1l				Wdr5	Zfp609
	Brd4		Arl2bp				Itpr1	Kcnk1	Cnr1	Runx1	E2f2		Tmem170				Csnk1e	Trdmt1
	Setd8		Gm10300				Cbx2	Cpsf6	Srsf6	Sfpq	Nvl		Matr3				Cdo1	Rad54l2
	Dynlt1a		Jmjd8				Prdm1	Acin1	Abhd17c	Foxj1	Dnm3		Fam63b				Ky	Add1
	Dynlt1b		Fam117a				Zfp398	Slc25a37	Zfp800	Zmpste24	Rgs19		Aebp2				Bend4	Slc43a2
	Josd1		RP23-293C19.2				Rad23b	Gdf11	2310035C23Rik	Srcap	Dfna5		Zfr				Lrp6	Kank4
	Bmpr1a		Sox13				Cd248	Rsrc2	Cpeb3	Magi2	Fam196b		Pvrl4				Gm5819	Gm2832
	Ppp1r15b		Stat5a				Fam134b	Mex3a	Slc31a2	Sema3c	Zscan25		Hlf				Vps33a	Rufy1
	Rev3l		Sema6b				Spin1	Glce	Mier1	Phf21a	Stau1		Mxd4				Chmp1a	Phf8
	Zfp366		Gpx1				Celf6	Zfp664	Rap1a	Bmpr1b	E330009J07Rik		Pik3ca				Tbl1x	Card6
	Tmem65		Lrrc7				Stxbp5	Brpf3	Dgke	Garem	2900026A02Rik		Arhgap19				1700026D08Rik	Sftpd
	Ltbr		Klrg1				Rwdd4a	Ankrd33b	Mtmr9	Mapk1	Gabrr1		Acvr2a				Smad7	Homer2
	Pex5		Lrba				Dixdc1	H1f0	Clip1	Rbms1	C7		Csnk1g1				Rapgef4	Kif24
	Calm3		Arfgef1				Zfp322a	Meis2	Rab5b	Rapgef2	Gtf3c1		Vegfa				Tln1	Rgma
	Dynlt1f		Gucd1				Crkl	Mcl1	Ptk2b	Dolpp1	4930571K23Rik		Pip4k2b				Ptp4a1	Rbmx
	Gfod1		Tesc				Arhgef12	Mbip	Fkbp15	Hmgb1	Hist1h1a		Foxk1				Krt84	Mlxip
	Jmy		Spsb1				Nipbl	Nog	Rasgrp1	Nxph1	Tusc5		Brwd1				Sez6l2	Tbc1d16
	Dolpp1		Steap4				Mpp1	Hoxa1	Bmp2k	D230025D16Rik	Ppp2r5b		Amfr				Rab7	Slc7a2
	Adamts7		Foxo3				Taf15	Fam217b	Fut9	Stau2	Scarb2		Cbln2				Ankrd27	Atad2
	Fign		Plin5				Mast4	Rab8b	Mfsd6	Grin2d	Lrrc56		Xkr4				Spry4	Inpp4b
	Ttc14		Smyd1				Cabp1	Fam107b	Ptprg	Bbx	5330438I03Rik		Trak2				Chst10	Vhl
	Hip1r		Lmbr1l				Grb2	1190002N15Rik	Fosl2	Gphn	Ralgapa2		Akap2				Gpr21	Slfn5
	Dennd4a		Gm17334				Nsg1	Wnt7a	Tnip1	Stx16	Dlx4		Ankrd52				Mapk9	Elovl6
	Cilp2		Esrra				Spast	Rab11b	Sema6d	Spryd3	Errfi1		Atrx				Nup62cl	Irs4
	Ywhae		Tysnd1				Rab7	Sel1l3	Mpzl3	Chst4	Gm10010		Olfr558				Pdcd6	Tulp4
	Fam57b		C1qtnf6				Dyrk2	Aadat	Zmat3	Gjc1	Ankrd54		Reln				Myo1c	1700066B19Rik
	Slc31a1		Klhl3				Pmepa1	Tifab	Sgcd	Mttp	Fat2		Palm2Akap2				Pnpla8	Rab44
	Birc6		Defb14				Rarg	Smad4	Tbc1d8	Siah1a	Gpr141		Sesn1				Slc4a7	A930033H14Rik
	Sidt1		Dpf3				Rap2b	Osr1	Cobll1	Cpd	Pnpla1		Reep1				Skil	Arhgef26
	Abcc3		Rgp1				Kpna3	Abcg4	Dkk3	Fbxl19	Bcl2l1		Rfx7				Numb	Tmem44
	Ercc6		Fgf12				Bcl2	Pfn2	Maml1	Thoc2	Syce1		1810013L24Rik				Bag4	Tef
	1700019D03Rik		Nudt1				Ahcyl1	Atf6	Rnf145	Jam3	Ell		Pim2				Itsn2	Ikzf2
	Rnf122		Srsf5				Ept1	Pcbp2	Orai3	Ddx5	Arhgap35		Gad2				Phf21a	Sfxn5
	Sh3glb1		Ccl4				Ptpn1	C2cd2	Abcb7	Bin1	Ercc4		Pi4kb				Prr15l	Cenpq
	Slc25a22		Pak2				Gpr85	Bmi1	Trappc10	Nptx1	Slc16a14		Gosr2				Tmem68	Zfyve20
	Fam193b		Rnf121				Wnt2	Tanc1	Pou3f2	Slc39a10	Foxn3		Jade3				Cep250	Ss18l1
	Vash2		Lrch4				Add3	Nrip3	Itpripl2	Arcn1	Tmem253		Pds5b				Slc39a14	Mtmr9
	Vps37c		Dhx33				Usp5	Ccdc68	Smoc2	Epha5	Klf12		Atxn1				Arhgef12	BC051076
	Lmtk2		Bzrap1				Sec62	Snx6	Fastk	Rgs7bp	Cxcr5		Sik1				Lrrk1	Slc35e1
	Tmod3		A430078G23Rik				Nf1	Hivep2	Btaf1	Cpsf7	Tax1bp3		Dgka				Tspan9	Pot1b
	Ino80d		6330416G13Rik				Clptm1l	Brd3	Mllt6	Zfp609	Gm13285		Eif3j1				Pdgfra	Shc3
	Lsm11		Hmg20a				Nup155	Gata3	Ago2	March6	Slc48a1		Zbtb12				Bnip3	Epg5
	Cdk6		A830073O21Rik				Pan3	Fos	Npepl1	Farp1	Dzip1		Aff1				Spats2l	Plekhb2
	Pdik1l		Pif1				Coa4	Fbxo38	Ireb2	Zbtb18	Gpatch2l		Klf4				Dennd6a	Sh3d19
	Prr5l		Tcf21				Jmjd6	Spsb1	Camsap1	Siah1b	Mrm1		Casz1				Dcun1d4	Zbtb39
	Creb5		Fignl2				Magi1	Gda	Arhgef12	Trim23	Rnf20		Spast				Slc33a1	Tmem127
	Rhobtb1		Gkn1				Capns1	Tcf21	Ugt8a	Tnr	Cers3		Npm1				Qser1	Cgnl1
	Zfp191		Serpina7				Myo1c	Ranbp1	Asb7	Khdrbs2	Lnpep		Fnbp4				Grb2	Sorcs2
	Npffr1		Ppp1r2				Slc39a10	Zfpl1	Slc9a2	Pdcd4	Igf2r		Lepr				Tpd52	Calml3
	Lmln		Smg6				Evi5	Plagl2	Tbx20	Mpped2	Rfwd2		G6pc				Pan3	Lphn3
	Ccser2		Col27a1				Vps26a	Mb21d2	Nek10	Zbtb21	Grap2		Surf4				Usp24	Fzd6
	R3hdm4		Adcy5				Cog3	Znhit6	Rasgrp4	Srm	Midn		Kif21b				Ppp1r13b	Sox11
	Ephb3		E130309D14Rik				Fam110b	Ackr3	Tada2b	Ldlrad4	Syne3		Gpr173				Cobl	Gif
	Dcaf7		Chfr				Acer2	Usp10	Fam104a	Sdf2	Sv2c		Bnc2				Pbx2	Swap70
	Acvr2a		Oas1a				Fbxo42	Plxnd1	Pknox1	Ythdc1	Srebf1		Frmd4b				Srpx2	Ddx17
	Frmd4a		Lrrc4				Ssh2	Abcc5	Rapgef4	Matr3	Tmem235		Fam168b				Nfic	Gan
	Slc1a2		Iglon5				D230025D16Rik	Cd69	Pcgf5	Mtss1	Pfkfb3		Ambra1				Ep300	Aar2
	Itgb1		Adh6-ps1				Gabpb2	Armcx4	Cep170	Ubap1	Tnfaip2		Ocln				Gria3	Gnb3
	Tnfrsf1a		Gm4858				Sept9	Rbms1	Epc2	Nfatc2	Efcc1		Fam118b				Arrdc3	Dusp3
	Dtx4		Nhlrc4				Slc35b4	U2surp	Limch1	Calu	Ctdsp1		Socs6				Ier5l	Dixdc1
	Ctnnd1		Macrod2				Slc35e4	Chmp7	Garem	Cdc14b	Nup153		Rac1				Foxf2	Znrf1
	Ythdf3		Dydc1				Scaf11	Plekha7	Tbk1	Nck1	Clcn4-2		Ankrd28				Trib3	Fam83f
	CTNND1		H2-DMb1				Hhip	Tmem120b	Psd	Tspan4	Traf3		Lrp4				Nhlrc3	N4bp2l1
	Clec2l		Pag1				Tmem163	Proser1	Jakmip1	3632451O06Rik	Ttc21a		Bach2				Uhrf1bp1	Clic5
	E2f7		Slc8b1				Scn2a1	Atg16l1	Lclat1	8030462N17Rik	Slc12a2		Dot1l				Ntn4	Paqr3
	Nfix		Ctdsp1				Amph	Hoxa10	Mef2a	Wsb2	Gabrq		Prtg				Vav2	Spn
	Kdm5b		Tmem125				L1cam	Ifrd2	Anxa7	Tfap2a	Tlr5		Tbl1xr1				Zmpste24	Trpv2
	Insrr		Ptprf				Arid4a	Ier5l	Pgm2l1	Dact3	Adcy1		Ube2i				Setd7	Ushbp1
	Atp6v1a		Rbms3				Furin	Rnf128	Dbn1	Pitx2	Srgap3		Igsf3				Otud6b	St8sia3
	Nol4l		Pde6a				Ppp1r12a	Hdac4	Hhip	Ccnt2	Dstyk		Smim13				Map2k1	Etnk1
	Bcl11b		Vcp				Nr4a3	Bcor	Cbln2	Onecut1	Tmem38a		Prkca				Smad1	Dzank1
	Cbx5		Slc36a1				Gga2	Ppp3ca	Synm	Csnk2a1	Ammecr1l		Kcnq4				Fxr1	Cpd
	Map4k4		Mme				Kat7	Ddhd1	Dnajb9	Nsmaf	Sstr3		Ormdl3				Lgi1	Phyhip
	1110002E22Rik		Elfn2				Nufip2	Map4k3	Klhl17	Top1	Dusp28		Btf3l4				Cbx2	Bmpr2
	Itga11		Cacna1e				Cav1	Metap1	Dock4	Pitpnb	Tnfrsf1b		Serinc1				Ccny	Myo1e

miR-199-3p	miR-29-3p	miR-486-5p	miR-361-3p	miR-122-5p	miR-425-5p	miR-136-5p	miR-96-5p	miR-142-5p	miR-19-3p	miR-141-3p	miR-770-3p	miR-335-5p	miR-200-3p	miR-194-5p	miR-183-5p	miR-341-3p	miR-182-5p	miR-370-3p
	Nktr		Dkk3				Tnrc6a	Rhobtb1	Hadha	Ppp2ca	Slc29a3		Glis2				Rgs2	Casr
	Mapk8		Kif5c				Tbc1d22b	Otud3	D1Ertd622e	Me3	4632404H12Rik		Tmem164				Dgkh	Mtfr1
	Nanp		Pnpla1				Denr	Nup35	Pitx1	Ccdc85c	Idua		Ddx3x				Cited2	Uvrag
	Bicd1		Ren1				Dock4	Dcaf5	Wipf3	Cdk6	Nle1		Gabbr2				Ralgapb	Arhgef37
	B230219D22Rik		Fam101b				Gcnt4	Mmp16	Fbxo32	Csnk1g3	Cntnap5c		Actr3				Cxadr	Cmtm4
	Pdp2		Capn1				B3galt2	Dhx40	Prrt3	Ttyh2	Gm10644		Zfhx4				Slain2	Igfbp5
	Cnnm2		Ldb1				Spats2l	Nsd1	Elk3	Tbc1d15	Atp11c		Baz2b				Rdx	Col6a6
	Dennd5b		Scd3				Arhgef3	Spen	Osbpl6	Tial1	Pcsk4		Astn1				Pcgf3	Mtmr3
	H2afx		Mtmr6				Zfp532	Slc30a10	Usp13	Cfdp1	Crhr2		Casr				Vat1	Tmem132b
	Schip1		Tmem44				Dnmt3a	Trim3	Acer2	Nap1l1	Igfbp5		Wasf1				D630045J12Rik	Zcchc6
	Parg		Asap2				Myo1b	Tppp	Stk38	Lrrc39	Gm14139		Trim71				Dmrt3	BC067074
	Ehd2		Dcun1d3				Dgkg	Mea1	Hrk	Meis2	Has3		Elf2				Gabarapl2	Lrtm1
	Sox12		Pofut1				Fem1c	Rgmb	Sox4	Kif1c	Fmnl3		Col4a3bp				2810403A07Rik	Gm6483
	AI314180		Cdhr1				Ipo9	Rprd1b	Fxr1	C530008M17Rik	Pla2g2f		B4galt6				Ccdc88a	Chrm2
	Scn3b		Slc2a9				Nrn1	Erap1	Lgalsl	Atp6v1d	Gpr39		Mmp16				Mark3	1810011O10Rik
	Htr6		Nr6a1				G6pc2	Chst3	Dennd6a	Fbrs	Grhl2		Lrp1				Ksr2	Kbtbd8
	Tmem127		Ecscr				Tpd52	F13a1	Pik3ca	Bbs10	Opa3		Timm21				Golph3l	Pxk
	Slc25a44		1700017B05Rik				Fam149b	Lhx1	Fam73b	Glrx	Cxx1b		Scn5a				Rab35	Ccbe1
	Oxct1		Slc4a2				Rab40b	Mat2b	Hspa4l	Pde8b	Hydin		Bcl2				Ttbk2	Ikzf4
	Gopc		Khsrp				Kcnh5	Rtn1	Cbx6	Sdc2	C130074G19Rik		Dmd				Zcchc11	Igfn1
	Dtwd2		Rnf141				Onecut1	Trpc4ap	Actn1	Smc3	Jam2		Cdon				B3galt2	Maml3
	Kdm4a		Zdhhc12				Rftn1	Caln1	Aebp2	Bcl11b	Srgap1		Rbm12b2				Slc22a5	Tbc1d2
	Abi1		Cacul1				Dmrt3	Sp9	Tmem63b	Jmjd1c	Tead3		Zfp704				Esyt1	Grasp
	Rasal2		Cd226				St3gal2	Mrps14	Ndnf	E130308A19Rik	Grin3a		Eri3				P2rx6	Zbtb37
	Zfp609		Kpnb1				L3mbtl1	Ppp2r2a	Sept7	Csf3	Suv39h1		Prdm1				Rdh10	Fam102b
	Impdh1		Pdp1				Pcgf5	Exoc5	B4galt5	Sik1	Crkl		Tjp1				Camk2n1	Bnc2
	Pcdha10		Mob3a				Lphn1	Tmem54	Sgcb	Peli2	A530016L24Rik		Bag6				Taf12	Ccnyl1
	Klf13		Pycr2				Cntn1	Snx3	1810011O10Rik	Hnrnpab	Pkp1		Srf				Gprasp2	Atp2b3
	Nufip2		Zfp595				Klhl2	Strn3	Tmem64	March5	Knstrn		Celf2				Trim52	Dcaf10
	Def8		Fam131a				Gatsl2	Rybp	Spen	A130010J15Rik	Lmx1b		Cacna1c				Cald1	Rps6ka2
	Kctd21		Ifitm7				Pdia4	Smad2	Runx3	Nub1	Slc9a1		Mob4				Fam53c	Gm5878
	Iffo2		Gm11128				Slitrk4	Zbtb7a	Eps15	Dennd2d	Faxc		Mpp5				Rell1	Jazf1
	Mier3		Nudt15				Scn9a	Fam131b	Cse1l	Preb	Mrps11		Desi1				Cobll1	Zfp148
	Fam124a		Fam83c				Cdv3	Jak1	Jarid2	CSNK2A1	Pea15a		Glra2				Paip1	Anks4b
	Epha1		Upf2				Hoxa9	Cnot11	Kdm6a	Agk	Cyp4f40		Csmd3				Kcnk10	Fam53b
	Zer1		Klf6				Sgk3	Blcap	Glis2	Arfgef2	AA986860		Wnt16				Eif4ebp2	Dcaf7
	Abhd4		Tbc1d2b				Kcnj2	Ptbp2	Gja1	Ntn1	Tnfsf13b		Jmy				Phyhipl	Tead1
	Mapre2		Nrp1				Yes1	Hhip	Vapb	Arntl	St3gal4		March6				Phf6	Hapln4
	Ubtd2		Lep				Pdgfra	Nedd4l	Papd4	Drd2	Rnf183		Adcy2				Map3k3	Atxn7l3b
	Phf21a		Gdf6				Ddhd1	Itga9	Glcci1	Fgd4	Kpna1		Desi2				Ppm1a	Galnt10
	D3Bwg0562e		Wnt8a				Rell1	Selk	Kif3a	Tox3	Tpmt		Pard6b				Sc5d	Napg
	Usp37		Celf3				Srcrb4d	Rims1	Zfp62	Rnf122	Zkscan8		Nrg3				Fam110b	Surf4
	Btbd7		Mb21d2				Tead1	Abhd5	Slmap	Snx18	Adat3		Ep300				D230025D16Rik	Mknk2
	Tspan4		Tmem170b				Anxa3	Snx8	Gm10797	Hlf	Smim7		Sox12				Wdr82	Als2cl
	Bsdc1		Pou2f2				N4bp1	Irf2bp2	Inhbb	Gabarapl2	Uprt		Rasgef1b				Adam19	Ccdc117
	Pthlh		Tnni3k				Ephb2	Cadm3	Frk	Lmod3	Ube2o		Zfand6				Zfp532	Braf
	Pdhx		Ephb3				Cdk5r2	Fzd9	Smim12	Tm9sf4	Otud5		Plk2				Sgms2	Gltscr1l
	Nkiras2		Kdm5c				Pdzd2	Arhgap35	Lrp12	Arhgap1	Fgf14		Foxf2				Mgat2	Rabl2
	Ythdf1		Slc25a17				Rsc1a1	Gemin5	Slc48a1	Bmp1	Pappa		Scamp1				Grb10	Irx6
	Aff4		Hmgb1				Atp8a1	Gnpda2	Adamts18	1110057K04Rik	Fgf22		Adipor2				Yaf2	Rsc1a1
	Dpp4		Otop3				Reps2	Taf3	Adam12	Mctp1	1810043G02Rik		Prkar2b				Morf4l1	Fmn2
	Brd3		Zdhhc14				Sesn2	Rerg	Bace1	Chic2	Tmem11		Usp6nl				Fam60a	Gtf3c1
	Nlrx1		Dtl				Myo5b	1810022K09Rik	Zfand5	Ptpn6	Tmie		Srgap1				Fyco1	Fam173b
	Apc		Gins2				Pcsk5	Tceanc2	Dlg5	Ssr1	Cstf2t		Nyap1				Gcnt1	L3mbtl1
	Gpr85		Fgf1				Adam19	Rbbp6	Ppfia2	Fundc2	Kcnq4		Ppp2ca				Scn1a	Nt5m
	Rnf138		Pecr				Mtf1	Neurl1a	Foxf2	Epha6	Tra2b		Ntf3				Gm20388	Srsf6
	Scml2		Nps				Gm17296	Sorbs1	Slc6a8	Foxa2	Nr5a1		Siah1a				Galnt2	Tvp23a
	Bahd1		Btk				Mon2	Fgf16	Cluh	Mdga1	Clmn		Fyn				Cpeb1	Hrh3
	Setd5		Ccpg1				Prpf19	Ube2z	Rnf216	Igf2bp2	Rai1		Cdyl2				4930402H24Rik	Psg16
	Elmsan1		Cabp4				Pcx	Btg1	Rtn1	Cep72	Nek6		Lrrc8a				Slc30a10	Ptpn13
	Rnf19a		Ctcfl				Elavl4	Trpm6	Kit	Fbrsl1	H6pd		Sema3f				1700123L14Rik	Iws1
	Nova1		Angel1				Rab6b	Ism2	Npas3	Atg7	Arid1b		Hivep3				Kcnh5	D430041D05Rik
	Pcyt1b		Cldn5				Socs7	Hoxb2	Cpeb4	Dcun1d5	A530064D06Rik		Dnajb14				Arid3a	Tango6
	Grm4		Pip4k2c				Aatk	Guf1	Scube3	Cul4b	Naa15		Ednra				Arcn1	Sdk1
	Fam76b		Zmym3				Arel1	1110037F02Rik	Plekhg5	Daglb	Pcnx		Tsc22d1				Fem1c	Rictor
	Etv4		Hrh1				Mtmr3	Fgg	Etnk1	Gm5084	Fads2		Tbp				Sypl	Xkr4
	AI118078		RP23-408P8.1				Dync1li2	Hn1	Usp8	Nelfa	6430573F11Rik		Atl2				Amotl2	Fibcd1
	Ubxn7		Fam109a				2610018G03Rik	Cdk5	Vkorc1l1	Dpf3	Syt14		St3gal2				Ttyh3	Zfp507
	Fam219b		Zfp941				Cobll1	Ppp2cb	2210018M11Rik	Hint3	Cnst		Slc6a17				Pcdh8	Tbl1xr1
	Tnfaip1		Plcd1				Arfgap2	Rab11fip5	Entpd7	Ing2	Scn4b		Efcab2				CTNND1	Il22ra1
	Spsb4		Ptk7				Prepl	Gmcl1	Fem1c	Tmem57	Clic4		Rbm26				Ctnnd1	Sort1
	Fstl1		RP23-113N18.1				Dos	Kctd6	Plxna4	Scaf8	Parvb		Cnn3				Mospd2	Cbfa2t3
	Chmp6		Rpgrip1l				Pde7b	Get4	Nlgn3	Sec11a	Lrtm1		Trio				Jade2	Neurl1b
	Camkk2		Brd2				Ncoa4	Cyp4b1	Foxred2	Hnrnpr	Ubxn2b		Trip4				Pla2g12a	Traf3
	Sidt2		Gpr27				Tsc22d3	Tmem108	Gpr137b	Rpa1	Npy4r		Arhgef17				Pcsk5	Sesn3
	Slitrk4		B430203G13Rik				Elmo1	Wnt11	Itch	Slc6a20b	Jdp2		Csnk1g3				Cog3	Tanc2
	Ncor2		Sbp				Fbxw11	Cdh24	Mef2c	Eif4ebp1	Gcm2		Ppp2r5c				Dock4	Mef2c
	En1		Limd1				Ncald	Iffo2	Phip	Phb2	Tmem178		Ccdc68				Klhl13	Fzd5

miR-199-3p	miR-29-3p	miR-486-5p	miR-361-3p	miR-122-5p	miR-425-5p	miR-136-5p	miR-96-5p	miR-142-5p	miR-19-3p	miR-141-3p	miR-770-3p	miR-335-5p	miR-200-3p	miR-194-5p	miR-183-5p	miR-341-3p	miR-182-5p	miR-370-3p
	Mat2a		Spic				Efnb2	Slit1	Onecut1	Alkbh3	Rab43		Gatad2b				Shank1	Rapgef3
	Faxc		Marf1				Zadh2	Slc6a6	Pxdn	Nop9	Abhd15		Pfn2				Slc35a2	Prdm16
	Stx1a		Arrdc2				Arhgap24	Rnf138	Arl8b	Tacc2	Msi1		Rnf2				Smim14	Itga11
	Suv420h2		Gucy2e				Lsm14a	Mgat5	Mid1ip1	Cnot2	Sorcs1		Gpatch2l				Acvr1	Bid
	Kcnip2		Fosl2				Scn3a	Gpr6	Tmem47	Cnp	Pigu		Uhrf1bp1				Sos1	Tdg
	Wasf3		Pou3f1				Tmem115	Arfgap2	Dicer1	Prkag1	Irak1		Bhlhe41				Neurl1b	Rnf24
	Dennd2c		Amer1				Tapt1	Slc25a46	Usp33	B2m	Cyp2u1		Ppp2r5e				Znrf1	Gm6086
	Ss18l1		Ywhag				Hcn4	Gtf3c2	Map3k14	Ndufb9	Sord		Tead1				Kcnv2	Hspg2
	Zfp641		Slc7a5				Cyb561d1	Irf2bp1	Zfp704	Maf	Hs1bp3		Rps6ka2				Dnmt3a	Dab2ip
	Tmem229b		Wdr13				Onecut2	Bves	Celf1		Ppp6r1		Tmem120b				Fbxw11	Btnl9
	Ubtf		Ftsj2				Ubxn10	Ralbp1	Zic5		Pprc1		Hipk1				Prrxl1	Ankrd46
	Fos		Erf				Astn1	Fgf12	Igf1		Atp6v1b2		Lrch1				Map2k3	Plbd2
	Rab12		AI462493				Ugt8a	Baz2a	Robo2		Slc25a30		Cited2				Zbtb7a	Brsk2
	Pou4f3		Shisa7				Prkar1a	Asb14	Plaa		Prob1		Cntn4				Frmd4a	Wasf2
	Wdfy3		Actl6a				Fnbp1	Pik3cd	Col4a3bp		Arl6ip5		Smarcd1				Rad23b	Nr2c2
	Phldb3		Asb7				Tnfaip8	Alas1	Strn3		Pard6b		Nrp2				Gpr22	Ptprg
	Irs1		Prmt1				Cep85l	Pou3f4	Zdhhc7		Hoxb5		Kctd10				Nt5dc3	Mcemp1
	Gmeb1		Lipe				Pik3r1	Mpped2	Fam196a		Dhps		Dcun1d1				Pfn1	Draxin
	Unk		Gng12				Ywhag	Loxl3	Mex3c		Zfp641		Rgl1				Dnajb9	Ago3
	Bcl7a		Kcnmb4				Slc25a51	Mbl1	Nrbf2		Spock2		Dcun1d4				Slc35c1	Ndc1
	Clk2		Trh				Kif19a	Phospho2	Prkcz		Casp9		Zfp395				D17Wsu92e	Nav1
	Rnpepl1		Rdh16				Stx5a	Shank1	Hs3st5		Tmem221		Scn1a				Kpnb1	Rprm
	Tgfb2		St6gal1				Tmem189	Zfp516	Fbxo28		Ctdsp2		Fam219a				Mphosph9	Ccdc157
	Adam23		Egr1				Arcn1	Rtn3	Plekhm3		Spint1		Efna5				Gm16039	Chst11
	Ttc9		Wdr25				Dcun1d1	Dlx3	Spata2		Kcnj12		Sbf1				Cadm1	Lrat
	Dgkk		Fgf16				Prrxl1	Rgma	Kcnt2		AA415398		Lemd3				Ocrl	Arhgef12
	Fbxw9		Rictor				Scamp3	Cyp7b1	Lemd3		Itga3		Fgd1				Gm17296	Erbb4
	Tspan9		Tacc1				St3gal3	Slc33a1	2510009E07Rik		Cxxc4		Hnrnpu				Arid4a	Lpar2
	Rbfox2		Cdyl2				Kcnk10	Ephx1	Fndc3a		Gldn		Elavl4				St8sia3	Smoc1
	Rarb		S100pbp				Fam78a	Otp	Zic4		Gm9990		Pthlh				Cdc42bpg	Spns2
	Htr7		Kifc3				Hs3st1	Adal	4930506M07Rik		Faah		Lfng				Dpy19l3	Inpp5f
	Btla		Dvl2				Arhgdia	Ywhae	Rps6ka5		Lancl3		Rab7				Ndnl2	Grid2
	Enah		Ldlr				Phyhipl	Lsm6	Lrig1		Kcnb2		Schip1				Amica1	5330438I03Rik
	Ipo9		Msrb3				Kdelr1	Ppt1	Efna5		Chuk		Maf				Cdc73	Pde4dip
	Setdb1		Il11				Fam60a	2310033P09Rik	L3mbtl1		Rab39		Prkacb				Hsph1	Kcna5
	Zkscan4		Ttc4				Pbx2	Tbx1	Id4		Wdhd1		Pam				Hey2	Rasgrf2
	Fkbp4		Wnt5a				Fam43a	Ncoa4	Dlc1		U2af2		Ptbp1				Rnf44	Arhgef17
	Mafb		Chmp4c				Nampt	Fip1l1	Sox5		Ccr1		Arl8b				Med14	Rhox9
	Adc		Nupr1				Phf6	Mroh7	Socs6		Gm2a		Mapk7				Marcks	Rab11fip3
	Tmtc3		BC018242				Trio	Srf	Gtf2h1		Vegfc		Slc39a14				Bhlhe22	Pde6a
	Zmym2		Rhbdd1				Dlat	Tspan3	Dnajc16		Olfr288		Socs5				Ogt	Ikbkb
	Nrsn1		Ubn2				Cdc42bpa	Gm6133	Zfyve26		1700101E01Rik		Suz12				Bre	Golga4
	Dicer1		Psmd2				Scn1a	3830406C13Rik	Zfp804a		Ccl28		Mycn				Ube3c	Hrk
	Mfap2		Pate4				Srpk1	Zfand1	Irx4		Fam109a		Gpr146				Trim8	Slc2a12
	Dab2ip		Prss38				Tmem50b	Slc43a1	Pcdh7		Csf3r		Mex3d				Eml5	Samd7
	Urm1		BC005764				Cdc37	Pmm2	Satb1		Mnt		U2af1				Ret	Dnhd1
	Tanc1		Pomk				Gpr22	Lyl1	Kcns2		Msrb3		Sephs1				Slc22a23	Pxmp2
	Gm10300		Dag1				Cbln4	Zfp282	Kcnf1		Cad		Hmgcr				Nkain3	4930544L04Rik
	Plekha1		Crim1				Vwa3b	Serbp1	Mon2		Vash1		Aff4				Ezr	Trak2
	Vps13d		Peli3				Sos1	Nucks1	Myt1l		Clcn6		Stx12				Cep85l	Pacsin1
	Cacng4		Gab2				Ebf1	Fam47e	Fndc3b		Prkar1b		Arhgdia				March3	Apc2
	Me3		Proser1				Eif3j2	Atg7	Wwp1		Gm16515		Depdc1b				Slc30a1	Fam216b
	Ric8		Grin2c				Gphn	Pam16	Nacc1		Map2k6		Sec24a				Snai2	Star
	Rnf165		Klhl11				Anxa13	Gzf1	Zfp644		Tmem164		Amotl2				Fpgt	Adamts15
	Dctn1		Psmd4				Nt5dc3	Htr2a	4931406P16Rik		Wdfy2		Zc3h15				Ephb1	Map3k14
	Elavl4		Snw1				Ell	Gm17349	Furin		Zfr2		Zbtb21				Rnf183	Arhgap22
	Slc39a9		RP24-392D11.2				Mterfd3	Tmem206	March1		Gm21379		Cask				Chst11	Cbx2
	Glis3		Dpysl2				Cul5	Cxcl14	Ephb3		Rbm31y		Agap1				Clvs1	Iffo2
	Rnd1		Muc4				Prkcz	Arpp19	Atf7ip		Tnfaip1		Cntfr				Ppp3ca	Usp24
	Hnrnpul1		Champ1				Lrrc28	Prss8	Raph1		Axl		Frs2				Onecut1	Gm26992
	Mospd2		St8sia1				Gorasp1	Snf8	Smurf2		Chdh		Smurf2				Dgkg	AI846148
	Klhl25		Lmna				Nox4	Chid1	Akap1		Trmt1l		Arl2bp				Isl1	Zfp964
	Spry1		Mchr1				Fam222b	Polr1e	Fam43a		Jag2		Tmem200c				Tox	Snx22
	Svil		Gabbr2				D1Ertd622e	Fam84a	Zfpm2		Adam11		Tln1				F13a1	Pdgfra
	Rest		Axin2				Ndrg1	Nploc4	Snip1		Vsx2		Fgd4				Zfp397	Tnik
	Hdgf		Klf7				Esrrg	Cinp	Add3		Fam102a		Kif13a				Zfp292	Myo3a
	Larp4		Ngb				Cers5	Arl4a	Rapgefl1		Ddi2		Arhgef1				Prkd1	Sybu
	Zfp362		Zfp558				Lgi1	Rars2	Slc30a5		Foxp3		Selk				Vps26a	Wbscr17
	Pggt1b		Twist2				Slc43a2	Tuba4a	Pde7b		Fbxl16		Spats2l				Ostf1	Clpb
	Tbx21		Klf16				Pak3	Epha6	Gsc		Rab37		Actr1a				Rhobtb1	Lamc1
	Ppargc1a		Stk16				Lrit1	Plin4	Sptssa		Lipn		Slc39a10				Ccdc92	C77080
	Loxl4		Tmem159				Golph3l	Hfe2	Mex3a		Uevld		Eif2b5				Zhx1	Prtg
	Plod1		Gjc2				Eml5	Pdcd2	Mn1		Slco3a1		Stard13				Cnn3	Brf1
	Dcp2		Stac3				Ovol1	Xrcc6	Slitrk2		Lhfpl4		Lphn2				Fam222b	Uggt1
	Myo18a		Fam84a				Pcmt1	Tmem120a	Tbcel		Trim5		Osr1				Plekhh2	Mapt
	Rnf214		Sun2				Pcmtd1	Gadd45gip1	Agap1		Cmklr1		Ipo5				Rftn1	Sv2c
	Pik3cb		Kctd7				Reep1	Mterfd2	Stard4		Nlrc5		Scn3a				Cdc37	1190005I06Rik
	Ncoa3		Mvb12b				Obfc1	Csf1r	Mtf2		Ifnar1		Glg1				Foxo1	Syndig1l

miR-199-3p	miR-29-3p	miR-486-5p	miR-361-3p	miR-122-5p	miR-425-5p	miR-136-5p	miR-96-5p	miR-142-5p	miR-19-3p	miR-141-3p	miR-770-3p	miR-335-5p	miR-200-3p	miR-194-5p	miR-183-5p	miR-341-3p	miR-182-5p	miR-370-3p
	Rab6b		2010003K11Rik				Paip2	Rpn1	Kcnh5		Eif3j1		Sgip1				Lrit1	Vdr
	Lypla1		A4galt				Mgat2	2510002D24Rik	Dag1		Slk		Dmrt2				Map1b	Spock2
	Camta1		Gigyf1				Arpc5l	Galntl6	Cdk13		Gskip		Crebbp				Prr11	Adat3
	Mstn		Cldn1				Ep300	Hpd	Bsn		Flnc		Eif2s1				Tmem127	Nfia
	Coro6		Tbl1x				Tfdp2	Aven	Nhs		Dusp3		Pou6f1				Ss18	Ablim1
	Fbxo7		Cdcp1				Vps26b	Cops6	Wee1		Hspa12b		Slc16a12				Lmod1	Impad1
	Fam78b		Ddx51				Etf1	Gss	Fam195a		Rel		Snx13				Meis2	K230010J24Rik
	Npas4		Glo1				Atf7ip	Tmem184b	Mdm4		Cdh23		Fam57a				Adam22	Baz2a
	Zic5		Marveld1				Gramd2	Rab35	Spred2		Amer3		Dcaf5				Plag1	Gm27021
	Ddx6		Rdh9				Cep83	Il21	Pdcd6		Gsk3a		Lpl				Klf13	Pdcd1
	Xirp2		Cryzl1				Atoh8		Pdik1l		Slc38a3		Vps33a				Kcnj14	Pde11a
	Slc16a1		Gm28046				Sez6l2		Efnb2		Pdgfd		Mettl16				Alg9	Hic2
	Tmem151b		Fut4				Ctdp1		Hoxa5		Arf2		Hccs				Tmem198	Pold4
	Syt7		Lclat1				Kctd7		Zfp281		Zfp599		Kdm4a				Ube2l3	Slc22a4
	Pou2f2		Mpdu1				Ttbk2		Slc11a2		Iqsec3		Sdc2				Surf2	Mks1
	Ccdc80		Mturn				Hecw2		Ube2d3		Impg2		A830073O21Rik				Ikzf1	Chtf8
	Cbx3		Arf3				Xdh		Smad4		Pecam1		Akap7				Fem1b	Frmd8
	Gmfb		Atp6ap2				Sbds		Barhl2		Pars2		Lrig1				Antxr2	Stac
	Fam131b		Spock2				Adra2c		Hif1a		Rasgef1a		Lrrtm3				Rab21	Lrrc20
	Fam136a		Maz				Ids		Sfmbt1		Vwa5b1		Tex2				Rab40b	Lmx1b
	Tenm3		Ppp1r3b				Ppp4r2		Kif1b		Rab6b		Fgf10				Tnfsf13b	Ddx6
	4931428F04Rik		C230062I16Rik				Snap23		Slc17a6		E2f1		Grap2				Nampt	Def6
	Amfr		Eya1				Phf13		Pea15a		Gga1		Cnot8				Kdm6a	Fbln5
	Chic2		Ltbp2				Cadm1		Ptpn21		Fut4		Kras				Nr4a3	Ucp3
	Chtf8		Srsf1				Tns1		Fosb		L3mbtl3		Ank3				Fchsd2	6330416G13Rik
	Sbf2		A730049H05Rik				Fnta		Pogz		Hs3st3b1		Appl1				Tspan14	Terf2ip
	Mllt11		Scg5				Lmnb2		Ctgf		Golga4		Ankrd40				Pak3	Pkhd1
	Dlgap1		Cnnm2				Sema6a		Plcl1		Smndc1		Ndn				Rab23	Raver2
	Ski		Grpel2				Rnf208		Luzp1		Adal		Fam3c				Nat8l	Sh2b3
	Ubiad1		Ankrd1				Ccny		Cadm4		Cdkn1c		Gab1				Hes1	Sdk2
	Prpf3		2610021A01Rik				Gcnt1		Zfp607		Arid3b		Tra2b				Cbln4	Sh2d7
	Agpat4		S100a4				Ccnd2		Mink1		Phf20l1		Chst2				Pik3r1	Srpk1
	Rab14		Gpbp1				Cdyl2		Rcor3		Psmd8		Crk				Srpk1	2510009E07Rik
	Asic1		Nfkbil1				Phldb1		Bzrap1		Panx3		Rlf				Akap7	Klhl36
	Ube2e3		Col8a2				Tmx1		Trp53inp1		Ckap4		Slc6a15				Gdnf	Htr6
	Ptprd		Fbrsl1				Pfn1		Grin2a		Asb16		Grip1				Zfp202	Ly6h
	Clmn		Cxcr4				Rcor1		Vps37b		Ccdc93		Rims2				Klhl2	Gm21092
	Emc3		Ufd1l				Grb10		Tesk2		Ypel2		Sgms2				Zic2	Pde7a
	Tmem255a		Chst15				Gm28048		Celsr1		Slc31a2		Pin1				Gm6878	Gpr173
	Ppard		Fbxo48				Ccnj		Rab34		BC018242		Hcn1				Rps6ka6	Fcamr
	Ppip5k2		Gng7				Pafah1b1		Pitpnm2		Fbxo47		Mxd3				Tmod2	Strn
	Ankrd13c		Uts2b				Hdac9		Lzic		Nek10		Lamc1				Ero1lb	Tnrc6c
	Cep85l		Ankrd63				Adamts18		Sec63		Narf		Wdr20				Slc25a1	Anxa13
	Socs7		Rgs12				Myadm		Tenm3		Rab27a		Krt80				Npm1	Carhsp1
	Gid4		Slc2a3				Arhgap17		Hdac4		Amotl1		Ctbp2				Mcmbp	Usp3
	F11r		Mnx1				Slc35c1		Arid1a		Gatsl2		Pigh				Dok4	Lmbr1
	4833424O15Rik		Hspa2				Tspan14		Vapa		B3gat2		Brms1l				Rab2a	Pde5a
	Mxd1		BC030336				Esyt1		Znrf1		Fam173b		Itpr1				Bhlhe41	Lrrc8e
	Dcaf12		Gapdh				Fyco1		Eogt		Il17d		Rnf38				Mdm4	Ttc21a
	Tmed9		Pacsin1				B3gnt2		Zfp609		Mro		Cebpa				Dbx1	Slco2a1
	Klhdc3		Gbp9				Ccdc92		Henmt1		Sos1		Trim62				Sppl2a	Tmtc1
	Sgms2		Susd5				Cers2		Ptp4a1		Arhgef17		Hbs1l				Rnf130	Ralgps1
	Zfp292		Rsph4a				Tox3		Lbh		Kif13b		Rif1				Ccdc117	Clec16a
	Tfap2c		Opa3				Numb		Phf13		Map9		Lmtk2				Rock1	Vipr1
	Ttyh2		Dnajc16				Bcat2		Nus1		Cmtm4		Atp11a				Plod2	Pcm1
	Prkra		Ppp1r9a				Lmod1		Snx18		Alx4		Neo1				Git2	Ets2
	Kif18b		Gm5434				Trp53inp2		Nckap5		Il22ra1		Egr3				Nrf1	Arhgap6
	Zfp384		Adck5				Rnf139		Acsl1		9330171B17Rik		Stx1a				Sgms1	Pkp1
	Tubb2b		Pianp				Asun		Ccdc47		Ptbp1		Robo2				Zic3	Flt1
	Capn7		A630081J09Rik				Flot1		Cblb		Tmem63c		Caskin1				Ccdc134	Papd5
	Rab40c		Kera				Mfhas1		Hoxa9		Ccdc157		Trp73				Zfand5	4933426M11Rik
	L3mbtl4		Srsf2				Ret		Tpm2		Flrt1		Srpk2				Ptpn1	Man1a
	Tbc1d7		Elavl3				Appl1		Vmp1		Ocel1		Nr2f2				Akap1	Ep300
	Pdgfb		Igf2r				Ccng2		Nek8		Sdr16c6		Ppp2r3a				Sbds	Atp5sl
	Med26		Ctif				Pcdh8		Nptn		Smok3c		Cblb				Rfxap	Hsf3
	Spty2d1		Msl3				Antxr2		Fnbp1l		Zfp93		Slc38a4				D1Ertd622e	Asb6
	Ank1		Gpr56				Stag1		Zcchc3		Ifitm10		Senp5				Slc22a21	Trim72
	Kifc2		Zfp616				Prr11		Adss		Elovl6		Ptpn11				Bcmo1	Egfr
	Rnf217		Casp9				Fam135b		Eif4g2		Sgcd		Rprd1a				Ppp1r12a	Zbtb6
	Lsm14b		Asprv1				Rbm15		Znrf3		Otof		Setd7				Dpysl5	Pdpk1
	Acer3		Mark4				2310022B05Rik		Eif4a2		Zfhx3		Prdx2				Usp36	Strip2
	Wscd2		Man2b2				Ccdc88a		Npr3		Mypn		Ip6k1				U2surf	Sipa1l3
	Arf5		Tmem119				Rhob		2700081O15Rik		Bcl7c		Aplp2				Klhl9	Mfn2
	Vps25		Ttc38				Tsc22d2		Scn2a1		Ttc9		R3hdm2				Rnf145	A730018C14Rik
	Ctnnbip1		Gcnt3				Smad1		Tm9sf3		Bmp1		Rnf5				Aph1a	Hnrnpa3
	A330050F15Rik		Ddx39				Pacs1		Sf3a1		Rbpms		Fbxl16				Txlnb	Tub
	H2bfm		Nt5dc3				Snai2		Blcap		Ip6k1		Trim23				Naa50	Astn1
	Tmem86a		Wfs1				Rab2a		Atp2b2		P2rx7		Gnb4				Fnta	Tmem131

miR-199-3p	miR-29-3p	miR-486-5p	miR-361-3p	miR-122-5p	miR-425-5p	miR-136-5p	miR-96-5p	miR-142-5p	miR-19-3p	miR-141-3p	miR-770-3p	miR-335-5p	miR-200-3p	miR-194-5p	miR-183-5p	miR-341-3p	miR-182-5p	miR-370-3p
	Bcl9l		Negr1				Shank1		Rasgef1a		Celsr1		Kctd6				Edem3	Hmg20a
	Nfatc4		Bcl2				Wdr47		Rab1		Prickle2		Myo1b				Bdnf	Ksr2
	Rbmx		Scamp5				Tox		Lin28b		Hs6st3		Gem				Skap2	Abhd2
	Dlg2		Tmem115				Zbtb7a		Eps8l1		Zfp410		Bptf				Usp5	Git2
	Ide		Atp1a2				Zic3		Caprin2		Ptges		Grin2a				Lrrc8c	Phlpp2
	Mmp16		Cry2				Ldb1		Etv1		Fbxo10		Ptbp2				Ppp1r9b	Zmiz2
	B630005N14Rik		Cacng2				Plekhh2		Fam20b		Cbln3		1810049J17Rik				Gnai3	C7
	Dlgap2		Golga2				Zdhhc5		Mef2d		Mau2		Cggbp1				Slc38a4	Atl3
	Zfp385c		Ccnd2				Rhobtb1		Sema6b		Arrb1		Acp1				Col13a1	Cacna1s
	Ncoa4		Hopx				Tbc1d22a		Rnf44		Lmod3		Vti1a				Brwd1	6430548M08Rik
	Onecut1		Bin1				Tspan9		Dtnb		Glipr2		Bcl9				Bace2	Cpne5
	Ndrg4		Lmx1b				Klf13		Vti1a		Smarca4		Mtus1				Pde11a	Phactr2
	Cuedc1		Tcta				Ywhae		Hnrnpf		Ywhag		Sh3gl1				Yipf4	Map3k7
	Car3		Kcnc3				Irf6		Dennd1a		Zfp768		Wbp1l				St3gal2	Lamc3
	Cnot6		Rab37				Rbfox1		Bcl3		Kbtbd12		Rad9b				Txnl1	Twsg1
	Ddah2		Psd				Frmd4a		Mat2a		Rrp1b		Nedd4l				Pax6	Fat3
	Mlkl		Myog				Emc7		Mark2		Sharpin		Zranb2				Sdc2	Plekha6
	Snx11		Ccer1				Bnip3		Spry4		Hs3st6		Kcnk12				Elavl2	Tnfrsf23
	Sdk1		A4gnt				Fgf14		Caprin1		E430018J23Rik		Blcap				Grid1	Angpt4
	Helz		Alpl				Amotl2		Git2		Anks6		Jag2				Pias1	Sp9
	Kcnh5		Cenpo				Polr3g		Ric3		F2rl3		Mafg				Pkn2	Ankrd40
	Ube2k		Rc3h2				Znrf1		Fbxo30		Gng12		Mras				Foxk2	Cbln3
	Ptprk		Zdhhc3				Dpysl5		Hoxd4		H2-Ob		Arcn1				Vps36	Gm9925
	Vps26b		Teddm1				Tmem212		Cdc73		Herpud1		Hecw2				Dcaf10	Lonrf2
	Foxp1		Lrtm2				Bend4		Tom1l2		Gm14403		Tubb5				2310022B05Rik	Bace1
	Ostc		Micall2				Rita1		R3hdm2		Mast4		Rab13				Plekha1	Pdzd2
	Sfpq		Sumo3				Lekr1		Mob3b		Ankrd63		Akt2				Nox4	Kcnn3
	Tmem167		Zfp36l2				Acvr1		Tbc1d12		Aen		Nfic				Reep1	Mprip
	Vps36		Lrp12				Pax6		Hlf		Cdc26		Papola				Nptx2	Lmln
	Smpd3		Gm10273				Sik1		Tmem9b		Ddr1		Prkar1a				Rundc3b	Mcat
	Bbc3		Hs6st3				Cebpa		Kazn		Zfp189		Reep3				Zdhhc5	Fnbp4
	Per1		Lyst				Pou4f1		Ficd		Smg5		Plaa				Slc39a13	Edc3
	Emb		Ppp1r14b				Taf12		Usp14		Utp14b		Rbfox2				Prrt3	Rabep1
	Enpp2		Limd2				Myo1d		Zxdc		Phlda3		Ubqln1				Myo1d	Hs6st3
	Tmem33		Ehd1				Arpp19		Prtg		Tbc1d5		Shc1				Gid4	Dlg3
	Atp1b1		Pfn1				Slc30a9		Csnk2a1		Hnrnpa3		Atp6v0a2				Orc5	Myh11
	Hlf		Nab2				March3		Zfand6		Ppp1r10		Soat1				Nipbl	Chrm1
	Trim24		Efna3				Smim14		Nlk		Vps8		Cd38				Dr1	Lzts3
	Plekhf2		Kat7				Ero1lb		Spock1		Ucp3		Tial1				Pdzd4	Glcci1
	Lsamp		Asap3				Wipf1		Arhgap5		Mrps18c		Pde7b				Pou3f3	Lcor
	Sntb2		Nlgn2				Lphn3		Rab23		Vwa3a		Msmo1				Snx18	Med25
	Spry4		Nubp1				Bcmo1		Cab39		Tmem165		Rims3				Igf2bp1	Sdc3
	Gcc2		Hlcs				Taf4a		Itpkb		Erlin2		Pcnp				Slc30a9	Tmem132d
	Orai3		Ssbp4				D17Wsu92e		Ebf1		Otog		Rnf11				Kctd2	3110062M04Rik
	Dedd		Mllt6				Cxadr		Zfp113		Zfp879		Irs1				Ammecr1l	Akt2
	Hepacam		Nicn1				Edem3		Psap		C1qtnf1		Ube2b				Mpv17l	Rtn3
	Trove2		Sbpl				Arpc1b		Klf6		Rgs9bp		Fgf18				Sik1	Rel
	Nasp		Ahsg				Paip1		Rest		Pip5k1c		Otud7a				Ctdp1	Gm5464
	Etv6		Trpv3				Atg7		Sorl1		Camsap1		Trp53inp2				Fzd9	Zfp143
	Nebl		Epm2a				Tmem203		Zer1		Rnase6		Ccdc47				Egln1	Angel2
	Drp2		Grem1				Pkn2		Scarf1		Fbxo32		Adrb2				Oprl1	Pitpnm3
	D16Ertd472e		Ptpn7				Bzrap1		Txndc12		Lrrk1		Gata2				Rab5b	Mgat5
	Fbxl20		Pramef12				Mettl11b		Olfm1		Parva		Ythdf2				Skp1a	Stap1
	Nrg3		Cd93				Gm6878		Zranb2		Anks1		Tril				Dgcr2	Ccdc36
	Rybp		Prss45				Adam22		Ccrn4l		Trim62		Atp6v1d				Mfsd5	Samd14
	Cenpb		Oaf				Rdx		Srgap3		Grm2		Sltm				Ablim1	Pitx1
	Ccdc108		Hes6				Nedd4l		Reep3		Ddx39		Rad21				Arhgap17	Rad50
	Tbck		Vps33b				Dennd6a		Ttc9		B4galt7		Senp6				Ube2r2	Gm1968
	Src		B430306N03Rik				Mgat5		Gmfb		Sprn		Cadm1				Fam73b	Myo1h
	Zfyve9		Rasl10b				S1pr1		Ero1lb		Klhl34		Tomm70a				Atg7	Fstl4
	Cx3cl1		Ubash3b				Tmem57		Yy1		Prcc		Dnajb9				Nlgn2	Gpr179
	Ppp2ca		Sik1				Cacna2d4		Lrrk1		Efr3b		Cab39				Zbtb1	Arl6ip5
	Arfgef2		Sdc1				Fxr1		Wbp2		Wasf2		Cdk16				Insig2	4931406B18Rik
	Pitpna		Asic1				Rims3		Chmp4b		Taok1		Slc16a4				Vangl2	Grid1
	Hspa4l		Sectm1a				Orc5		Mapk10		Iqcb1		Cops2				Mtch2	D430019H16Rik
	Sike1		Tgfbr3				Kdm6a		Elavl2		Zdhhc7		Mocs1				Nap1l1	Fam19a3
	Metap2		Pcgf2				Pias1		Sptlc2		Zkscan7		Klf13				Rbm15	Etv5
	Naa60		Celsr3				Prps2		Calm1		Treml4		Lmo4				Asb14	Mettl4
	Ppp1r13b		Trnp1				Ldlrad4		Nrarp		Slc6a17		Arl3				Esrrg	B4galnt3
	Mxi1		Prdm11				Kctd2		Abr		Trp73		Nme2				Cask	Top3a
	Dclre1b		Dcun1d1				Champ1		Lrch2		B4galnt3		Gm20390				Tsc22d2	Hephl1
	Diablo		Egr3				Cygb		Kl		Scml4		Phospho2				Ddah1	Hyal1
	Gpr161		Ier5				Tmem86a		Zbtb7b		4930505A04Rik		Tmem126a				Mgat5	3830408C21Rik
	Adamts5		Tgfbrap1				Crebrf		Bptf		Gm12888		Emc4				Rgma	Zfp282
	Slitrk3		Apobec2				Aff4		Sema3a		Oas1a		Ywhae				Tmem47	Hnrnpa1
	Dip2c		Cdkl3				Slc39a13		Nova1		Mgll		Mrps25				Lphn3	Tbc1d13
	March9		Sdc4				Elavl1		Sept6		Tal2		Kif26b				Efna5	Marf1
	Kif3c		Slc24a3				Fam73b		Morc4		2310067B10Rik		Etf1				Slc35a1	Pnma3

miR-199-3p	miR-29-3p	miR-486-5p	miR-361-3p	miR-122-5p	miR-425-5p	miR-136-5p	miR-96-5p	miR-142-5p	miR-19-3p	miR-141-3p	miR-770-3p	miR-335-5p	miR-200-3p	miR-194-5p	miR-183-5p	miR-341-3p	miR-182-5p	miR-370-3p
	Cse1l		Calm1				Ddb2		Ccnd1		Tifab		Tmem258				Prkar1a	Pik3cd
	Erp44		Mbd6				Isl1		Macc1		Ppfibp1		Slc39a8				Ptrf	Sez6l
	Thoc1		Ndufab1				Git2		Cand1		Myo1c		Serpinc1				Dhcr24	Tmem216
	Adam22		Hdac8				Elavl2		Lsmem1		Emp1		Ndufb2				Parpbp	Chst15
	Sap30l		Cst6				Cnih4		Pdcd10		Pttg1ip		Csrp2				Irf2bpl	Usp13
	Mapk10		Taf13				Eif4e2		Polr3d		Zhx1		Prmt7				Slc7a8	AI314180
	Fgd4		Ampd3				Mapre1		Tcea1		Fzd1		Pmpcb				Bzrap1	Dhx37
	Ext1		Sema4c				Cpsf6		Rnf41		Ap2a2		Gpr161				Polr3g	Nol4l
	Clpx		Trps1				Plscr2		Rufy3		Usp9x		Mrpl24				Rims3	Mecp2
	Rras2		Cav1				Ap1s3		Ube2a		Por		Acot13				Colec12	Amotl2
	Med28		Syne3				Faf2		Dnajb14		Fbxo36						Bcat2	Irf4
	Rcc2		Zfp691				Ehd1		Klhl11		Smarcc2						Fam122a	Rassf8
	Tmem259		Hip1r				Mettl10		Tlr12		Trim12c						Epha7	Aff4
	Luc7l3		Tfap2e				Rbm26		Tln2		Gatad2a						Fgf14	Ap3b1
	Kcnk10		Uck2				Rcc2		Ormdl1		Maml1						Pnisr	Zeb1
	Lyl1		Pdrg1				Rab34		Elavl1		Atp2b4						St8sia5	BC003331
	Fyn		Zfp273				Tmem74		Lsm11		9130023H24Rik						Prps2	Bach2
	Pole3		Cds2				Ube2k		Gabra4		BC035947						Gphn	6330408A02Rik
	Arpp19		Ubp1				Pyroxd2		Hs2st1		Ttc7						Cnot6	Zfp879
	Paip2		Nkx2-3				Dclre1b		Kdm1b		Syngr3						Lzic	Onecut3
	Ctc1		D130052B06Rik				Rps12		Sec11a		Muc19						Mettl11b	BC021891
	Gng2		Brk1				Atg13		Rbbp8		Dot1l						Pou4f1	Desi2
	Ing2		A530084C06Rik				Egln1		Igsf10		Gm15319						Elavl1	Arhgap32
	Actn2		Otop1				Slc8a1		Arhgap1		4930438A08Rik						Ap1s3	Cbs
	Dcun1d5		Aoc1				Epha5		C230052I12Rik		Leng1						Stam	Smg9
	Exoc7		1810011O10Rik				0610007P14Rik		Fbxo3		Mkl1						Ddb2	Rdh16
	Arl4a		Trem1				Ell2		Arpp19		Fam178b						Ywhae	Rere
	Fbxw2		Smpd3				Mios		Mapk8		Krt84						Mras	Fbn2
	Fam149a		Aff3				Dlgap2		Ccna2		Mtg1						Ube2f	Pou2f2
	Mtif3		Nsl1				Ube2w		Usp24		Arfip2						Dcaf12	Zbtb43
	Ldlrad4		Lrguk				Sap30l		Rbm15		Tspan14						Smg5	Fcrls
	Oxnad1		Slfn5				Mdp1		Fzd8		Gm7461						Wnt2	Dtna
	Fga		Zbtb40				Hs2st1		Exoc5		Tnk2						Arpc1b	Kpna4
	Tyw5		2310067B10Rik				Snf8		Hoxb4		Cyp1a1						Tomt	Ccdc37
	Acadl		Ttc28				Prox2		Stk39		Morc2a						Fap	Rab15
	Denr		Slc1a2				Echdc1		Tlcd1		Rhox9						Zbtb24	Ipo8
			Cdca5				Atp9a		Tmeff2		Edn3						Etf1	Ggt5
			Acss1				Gpr126		Stc2		Ephb2						Vmac	Stard13
			Pim3				Miox		Limd2		Nwd1						9130213A22Rik	Kank2
			Mfap2				Mef2d		Rbfox2		BC023829						Whamm	Omp
			Morn4				Tmem126a		Mbd2		Tef						Prss12	Hs1bp3
			Zfp518b				Eif2s1		Tax1bp1		Vsig10						Klf15	Atg16l1
			Pi4kb				Fhl3		Gm15217		Ctsb						Nnmt	Rin1
			Rbms1				Tpm2		C1ql1		Arl4c						Ube2k	Slit1
			Pdgfb				Ppp1r11		Chst7		5730596B20Rik						Smc6	Zfp398
			Abcg4				Nop9		Hnrnpd		Kansl1						S1pr1	Zfp251
			Gm9881				Nudt2		Olfml3		Slc12a6						Ttc21b	Mfap3
			Ddr1						Cryz		Spata18						Rab10	Brap
			Pgap1						Hdlbp		Glra2						Enkur	Hinfp
			Gm5549						Ncf2		Serpina1f						Tmem86a	Ttc39b
			Rab3gap1						Lrrc40		Fkbp10						Aff4	Surf6
			Fcamr						Ttc32		Cdkn2aipnl						Guf1	Setd7
			Grik3						Sri		Lrsam1						Lmnb2	Lmtk2
			Ptgir						Hnrnpu		Als2						Slc8a1	Zbtb40
			Gfod2						Fezf2		Ssh1						Cyld	Ptprc
			Dgat2l6						Abi3		Syne1						Casd1	Ankrd23
			Ppfia2						Hpd		Mr1						Hs2st1	Igf2bp2
			Lurap1						Eif3e		Psd3						Rab34	Vgll4
			Gcc1						Dusp7		Thsd4						Gpr126	Ptpn7
			Asb6						Tmem165		D930048N14Rik						Mapre1	Spire1
			Cmtm2a						Rnf208		Pitpnm2						Tmem203	Ip6k1
			A830018L16Rik						Comtd1		Capn6						Fbxo28	9930022D16Rik
			Cntd1						Acot13		Tmprss3						Ube2w	Odf2
			Dusp8						Adipor1		Bbx						Ell2	Dyrk1b
			Zwint								Sept1						Pacs1	Iqsec1
			Lat2								Prdm6						Kif2a	Rmnd5a
			Dusp4								Peg10						Atp9a	Zfp954
			Neurod6								Otud3						Dusp1	Nkx6-3
			Rcn3								Glb1l2						Sec11a	Kcng4
			Mcur1								Trim7						Hadh	Eya1
			Rprd2								4933406P04Rik						Ajuba	Srp54a
			Dlx1								9830107B12Rik						Pyroxd2	Hmgcs2
			Ppfibp1								Ccdc134						Snf8	Cep170b
			Mtpn								Lzts3						Caln1	Gm4861
			Ntm								Fcamr						Rnf139	B3gnt3
			Cecr6								Tubgcp6						1200014J11Rik	Sox10
			Gm614								Pak4						Armc1	Cds1
			Mafk								Ccdc120						Mef2d	Gpr61

miR-199-3p	miR-29-3p	miR-486-5p	miR-361-3p	miR-122-5p	miR-425-5p	miR-136-5p	miR-96-5p	miR-142-5p	miR-19-3p	miR-141-3p	miR-770-3p	miR-335-5p	miR-200-3p	miR-194-5p	miR-183-5p	miR-341-3p	miR-182-5p	miR-370-3p
			Cirbp								Stk35						Tmem74	Pofut1
			Lmnb2								2810428I15Rik						Stau2	Gpr152
			Fuk								Mxd4						Dlgap2	Ndst1
			Abhd2								Gm5423						Dlat	Ctif
			Taf8								Kcns3						Hmox2	Mex3d
			Ubox5								Nat2						Rcc2	4930415F15Rik
			Mlx								Fam129b						Rbm26	Ncoa5
			Col6a6								Zmiz1						Sap30l	Slc39a14
			Cgnl1								K230010J24Rik						Prox2	Trim67
			Bak1								Cacng2						Mrto4	Hpgds
			Gm21967								Pramef12						Dclre1b	Grem1
			Zmynd12								1110057K04Rik						Cnih4	Fbxo47
			Xkrx								Clrn1						Zfp827	Sgsh
			Cdk2								Cdyl2						Slc22a1	Pum2
			H2-T23								Grid1						Rd3	Mis12
			Pawr								Ubtf						Jade1	Cds2
			Clptm1l								Slc9a9						Fdx1	Eya3
			2610018G03Rik								Thoc3						Mios	Nuak1
			Kctd18								Cnn1						Cdc23	Vezf1
			Cplx4								Kdm4b						Tmem126a	Xrn1
			Pax2								Prelp						Traf4	Enpp5
			Tex19.2								Sirpa						Ptch2	Slc25a15
			Cldn2								Zfp446						Nup50	Ptp4a2
			Erbb2ip								Tmem245						Oaf	Mapkbp1
			Adarb1								2010109I03Rik						Dnajc15	Tardbp
			Gatad2b								Map4						Mapre3	4930595D18Rik
			Sfxn2								Mrgprx2						Eif2s1	Urad
			Map7								Dcun1d3						Ap5b1	Sec62
			Amica1								Opcml						Ppp1r11	Kcnj1
			Prph2								Tulp4						Echdc1	Chmp3
			Enox1								Tomm5						Nop9	St6galnac6
			Rnf112								D630044L22Rik						H2-Bl	Ncald
			Adamts10								Rhcg						Hilpda	St8sia6
			Pln								Gltpd2						Fhl3	Osbpl5
			Tspyl1								Zscan20						Nudt2	Foxp4
			Ppargc1b								Gad2						Fxn	Med19
			Idh3g								Tmco1						Vbp1	Ptgdr
			Rnf215								Mark4						Srsf5	Ankrd11
			Ap1s1								Pdk4							Tns1
			Hspb8								Kcnb1							Meis1
			Raver2								Dcps							Acaca
			Cmas								Nabp1							Herc1
			Peg12								B230217C12Rik							Nr2c2ap
			Olig2								Rffl							Ntsr1
			Fads6								Lgals12							Slc30a7
			Usp37								Rnf44							Slc12a7
			Sparc								Myt1							Slc6a17
			Hist1h3d								Inhbb							Oas1g
			Tmem9								Il2ra							2410004P03Rik
			Mxra8								Trim56							Add3
			Bpnt1								Iqce							Ackr2
			Ppp2r2a								Mapt							Rgs16
			Vat1								Zfp775							Rxra
			Kcns2								Tab1							Ceacam9
			Sstr3								Tmbim7							Ccdc134
			Phlda3								C230062I16Rik							Cep112
			Rps4x								Txnip							Mlxipl
			Tmem164								Armc9							Nr4a3
			Timm23								Fnbp1							Cacna1d
			Iqcj								Pphln1							Zbtb46
			Ywhae								Neurl1b							Aff1
			Pgk1								Gria1							Slc27a1
			Hspa12a								Plxnc1							Fgf6
			Mrpl22								Epb4.1l1							Wdr91
			Slc35c1								Prrt1							E030010N08Rik
			Nf1								Abcd1							Ahrr
			Krt84								Ppp2r1b							Spsb1
			Traf2								Gtf2f1							Crtc3
			Dpysl5								Cd200r3							Npc1
			A1cf								Lpin3							Sit1
			Psip1								Sez6l							Bcl2l13
			Nfatc4								Gm17067							Htr4
			Btbd7								Lgi1							Kctd12
			Snx22								Gm20708							Kat6b
			Galnt5								Afmid							Ppp3r2
			Oas1e								P4ha2							Mapk11
			Mogat2								Man1a2							Sec16b
			Ctbp1								Vmn1r58							Gpr101

miR-199-3p	miR-29-3p	miR-486-5p	miR-361-3p	miR-122-5p	miR-425-5p	miR-136-5p	miR-96-5p	miR-142-5p	miR-19-3p	miR-141-3p	miR-770-3p	miR-335-5p	miR-200-3p	miR-194-5p	miR-183-5p	miR-341-3p	miR-182-5p	miR-370-3p
			Tmem184b								Mapkapk2							Cog8
			Rab1b								Fgf10							Cog3
			Hadh								Cacnb4							Nolc1
			Mapk4								Sdk1							Slc4a8
			Plch1								Atp6v0e2							Sec61a1
			Il13								Zbtb26							Gm12353
			Scg2								Wnt4							Golga3
			Gm9979								Rab3c							Rag1
			Hes3								Dvl3							Trim14
			Galnt16								Sorbs3							Med12
			Cd81								Slc7a8							Abl1
			Abl2								Paqr7							Cd8a
			Cldn18								Nphs2							Adamts14
			B4galt5								Tacc1							Scn7a
			Nup98								Tmem181a							Glb1l2
			Sv2a								Eps15							Tbck
			Aqp3								Rtl1							Fam212b
			Sox18								Jph3							Snhg11
			Prop1								Cenpo							Traf2
			Bsg								Haghl							Cyth4
			Ecm1								Taf8							Ttll12
			Clec2d								Prkar2a							Fam101b
			Krt73								Gif							Inhba
			Atp1b4								Top3a							Zfp882
			Mettl1								Arf1							Adcy3
			Mcf2								Nrl							Dok5
			Gtf2ird1								Ddx21							Kcnj16
			Mpeg1								Bean1							Gnl3l
			Lphn1								Plcd3							Ptpn1
			Etnk1								Cacna2d4							Slc2a10
			Kcna2								Hsd3b3							Cadm2
			Ldb3								Zc3h4							Cdr2l
			Olfr519								Rho							Zyg11b
			Mocs2								Glcci1							Elmsan1
			Pick1								Tbcel							Kif16b
			Chrm3								Ier3							Ankrd6
			RP23-133G16.2								Xlr5a							Pdc
			Palld								Tmem214							AF366264
			Fam160a2								Sbk1							Sec22c
			Emp1								Mrvi1							Tmcc2
			Rnf182								Hcn4							Map2k7
			Mafa								Fam83g							Gid4
			Arhgap30								Cep85l							Tcf4
			Limk1								Gabpb2							Nptx1
			Fam57b								Cpa5							Mylk4
			ARHGAP30								8430419L09Rik							Cdc42bpa
			Tlr12								Slc35d1							Sh3glb1
			Zfp319								Arl15							Adck3
			Arvcf								Plxna2							Ern1
			Cdc27								Myo18a							Megf11
			Npepps								Pydc3							Tstd2
			Kctd17								Xylt2							Crat
			Il1b								Btbd19							Rab14
			Hoxd12								2510003E04Rik							Stat3
			Grap								Fev							Slc10a5
			1700029F12Rik								Hibch							Kat7
			Nfic								4930562C15Rik							Aff2
			Bmp5								Dlg5							Sned1
			Camk2n1								Snn							Fam193a
			Klhdc3								6330408A02Rik							Slc35d2
			Hsf5								Cd33							Arfip2
			Gm853								Amer2							Deptor
			Gfod1								2610021A01Rik							Trim37
			En1								Hrk							Depdc5
			Endou								Larp1							Klhl18
			D630003M21Rik								Bcr							Zfp334
			Galnt18								Frrs1							Frrs1
			Ern1								Clec4a2							Dmxl1
			Cad								Hnf1a							Fam195b
			Arl16								Csmd2							Trmt13
			Wdr91								Med20							Gm10935
			Cbln2								Supt16							Laptm5
			Agtr1b								Epx							Kcnv2
			Mettl7a3								Mllt1							Zmat3
			Tmem63b								Ciao1							Tmem235
			Gli1								Calu							Atp9b
			Usp18								Tor4a							Nfam1
			Pigg								Prrt4							Ncs1

miR-199-3p	miR-29-3p	miR-486-5p	miR-361-3p	miR-122-5p	miR-425-5p	miR-136-5p	miR-96-5p	miR-142-5p	miR-19-3p	miR-141-3p	miR-770-3p	miR-335-5p	miR-200-3p	miR-194-5p	miR-183-5p	miR-341-3p	miR-182-5p	miR-370-3p
			Tnfaip1								Snapc5							Pik3ap1
			Ncbp1								Tmem186							Raf1
			Hapln3								Tnfsf18							Colq
			Ipo5								Rnd1							Pde3a
			Gdnf								Padi2							Chd5
			Cmklr1								Slc13a5							Lin28a
			Ppp2r5d								Pdzd4							Pfkfb4
			2810403A07Rik								Slc29a4							Nfatc3
			Cdkl1								Napb							Nfic
			Gm5286								Cd44							Igtp
			Agpat3								Hsd17b7							BC021785
			Slc16a12								Rbm4							Gm20489
			Entpd5								Chad							Brat1
			Zbtb1								Foxd2							Nacc2
			Cd47								Gpc6							Prr14l
			Wnt7b								POU2F1							Herc2
			Prx								Padi1							Cflar
			Olah								Hist1h3h							Dffa
			Slc16a5								Kdm4a							Sh3rf2
			Cdc23								Ms4a6c							Stox2
			Wnt10a								Meis1							Rapgef6
			Mfn2								Ncald							4930539E08Rik
			Rrh								Dgkg							Tcerg1l
			Btbd9								Pydc4							Alkbh1
			Cdo1								Clec9a							Gimap6
			Usp20								Sall2							Ddx3x
			Gan								Slc4a1ap							Ttc23
			Crb2								Ccr5							Dnajc3
			Adap2								Garem							Atxn1l
			3100002H09Rik								Ngef							Adam11
			Micu2								Ttc28							She
			Gm13288								Fam213a							Atp5c1
			Map3k4								Gm14085							Man1b1
			Nipal4								Coro2a							Hmgxb4
			Triap1								Arhgap33							Gab1
			Lypla2								Brat1							Ipmk
			Rbfox3								Rsf1							Lsm14b
			Fzd5								Ubfd1							Cend1
			Six4								Fhdc1							Cdkn1c
			Dagla								Gm12258							Dok4
			Piwil1								Tspyl2							Mpp5
			Hs3st2								Dnajb2							Stxbp4
			Acsl5								Nipal4							Btn1a1
			Dok7								Tusc2							Cacna1i
			Brap								Kif5a							Birc2
			Gm9966								Hpse2							Pde4a
			Gas2l1								Rapgef3							Parm1
			Zbtb12								D6Ertd527e							Ing5
			Naa30								Rgs1							Zfp236
			Sdk2								Cyp4f39							Plcg1
			Large								Ifi203							Katnal1
			Dazap1								Scarf1							Stxbp5l
			Adamtsl1								Il2rb							Xdh
			Akna								Rptor							Wdr76
			Cluh								Opn1sw							BC055111
			Idh3b								St8sia2							Homer1
			Olfr912								Gprin3							Mocs1
			Lrrtm4								Gm21992							AU021092
			Comp								Mtss1l							Cdk19
			Gm26566								Gpr165							Art2b
			Mobp								Ipo8							Wtap
			4932429P05Rik								Trabd							Slc16a12
			Exd2								Cdh15							Slc2a4
			5031439G07Rik								Ankdd1a							Myh14
			Shroom3								Tmem72							Efcab2
			9030612E09Rik								Abcf2							Elavl3
			Slc2a5								Slc26a10							Rhof
			Eda								Ebf4							Ncapg2
			Pak4								Fhl5							Ubxn2b
			Plk1								Rnf186							Synrg
			Hyou1								Efcab6							Clip4
			Nfix								Lrrn4cl							Dpysl4
			Gm28049								Dll4							Dock4
			Wdr41								Tcf4							Insrr
			Dmwd								Ncoa3							Dcaf5
			Tap2								Obfc1							Wdr54
			Cadm3								Slc24a3							Ccdc171
			E430018J23Rik								Ctns							Kctd3

miR-199-3p	miR-29-3p	miR-486-5p	miR-361-3p	miR-122-5p	miR-425-5p	miR-136-5p	miR-96-5p	miR-142-5p	miR-19-3p	miR-141-3p	miR-770-3p	miR-335-5p	miR-200-3p	miR-194-5p	miR-183-5p	miR-341-3p	miR-182-5p	miR-370-3p
			Pced1a								Cep164							Tm9sf4
			Senp6								Heatr6							Arcn1
			Gm1968								Endov							Synj1
			Gdap1								Rhof							Kcnj2
			Mtap								Rsbn1l							Fzd4
			P4ha2								Gsg1l							Pqlc2
			P2ry10								Smad4							Plekhm3
			Csf3								Trim44							Popdc2
			Drd4								Tmem229b							Neo1
			Stk32c								Tmem19							Sema3a
			Sit1								Evi5l							Zfp551
			Slc17a5								Ppp1r16b							Adcy6
			Lrat								Sqstm1							Huwe1
			Npc2								A730018C14Rik							Slc5a2
			Hoxa7								Zfp39							Cpsf4l
			Supt16								Ptcd1							Sez6
			Asb13								Uvssa							Zfp746
			Ankrd34b								Slamf7							Lpin2
			Lum								Idh3b							Gemin5
			Cnr2								Gm21885							Rrp1b
			Pisd								Ppt2							Mtx3
			Zfp445								Zcchc3							Dcdc2b
			Tmprss11g								Gdnf							Ggta1
			Clca5								Kcnab2							Reep6
			Cdca7l								Med22							Specc1
			Prox1								Rgag4							Crx
			Nans								Pigv							Jmy
			Tspan33								Draxin							Sgsm2
			Sepsecs								Rap1gap2							Ppp6r2
			Uba1y								Zfp831							Col20a1
			Prrg3								Daglb							Gulp1
			Wisp2								Lrrc52							Havcr2
			Nsg2								Camk2a							Skil
			Tmem59l								Cyth1							Angel1
			Rhbdl3								Aoah							Fam20c
			Mvk								Nrsn1							Atp11a
			Ncs1								Abcf3							Chd6
			Gnaz								Nat8l							Map3k4
			Kcnb1								Sec22b							Serinc4
			Zfp953								Zfp120							Gipc3
			Khdc1b								Lzts1							Ralgapa1
			Angptl4								Dzank1							Fbrs
			Pogk								Pi4k2a							Sdr9c7
			Nrbp1								Mfrp							Etv3
			Cops7b								Kdm2a							Rab11fip4
			Spns2								Gck							Opalin
			Slc38a1								Kif24							Casq2
			BC089597								Gjd4							Vash2
			Acot11								Slc25a19							Rdh1
			Pitpnm3								Nol10							Shank2
			Abcg8								Gm28049							Ascc2
			Syt13								Lrrc49							Mfap3l
			Heg1								Homer2							Syt2
			Prrg2								Cltc							Ppp1r16b
			Sec22c								Gm21092							Tmem47
			Rab30								Trim30b							Gramd1b
			Zmat2								Ube2q1							Vav2
			Mrap2								Pgpep1							Xpr1
			Kcnk2								Mtap7d3							Pom121l2
			Bfsp2								Polr3a							S100a7a
			Zfp957								Myt1l							Sema4f
			Trim17								Ddhd2							Rnf216
			Arhgap44								Mrgbp							Itih5
			App								Ldb3							Agfg1
			Serinc4								Syt12							Fer
			Galnt10								Mthfd1							Hnf4a
			Pigr								Ccdc177							Fosl2
			Pou2f1								Efnb3							Adamts2
			Rpap1								1700028P14Rik							Zdbf2
			Isoc2a								P4ha1							Zfand5
			Dlst								Gm10742							Gtf2a1
			Foxb1								Rreb1							Dph1
			Gm10302								Mdp1							Pdxdc1
			Rdh8								Bcl9l							Mcf2l
			Rhoj								Tmem150b							Prx
			Vmn2r34								Kctd7							Ccdc13
			C1qtnf1								Ermap							D930048N14Rik
			Pou3f3								Lrrc32							Parva

miR-199-3p	miR-29-3p	miR-486-5p	miR-361-3p	miR-122-5p	miR-425-5p	miR-136-5p	miR-96-5p	miR-142-5p	miR-19-3p	miR-141-3p	miR-770-3p	miR-335-5p	miR-200-3p	miR-194-5p	miR-183-5p	miR-341-3p	miR-182-5p	miR-370-3p
			Papln								Ptgdr2							Lpl
			Impad1								Hoxa6							Ptgir
			Proser2								Gm21119							Tpm4
			Prom2								Ush1g							Bcl2l1
			Vps26b								Paox							Bcl7c
			Lamp3								Nuf2							Zfp618
			Tgfb1								Gucd1							Bicd2
			Zik1								Otp							4930556J24Rik
			Atxn1l								Armc7							Ambra1
			Stx6								Rasgef1c							Mettl14
			Nlgn3								Pitpnc1							E2f5
			Micall1								Gpr45							Adora1
			Pcyt1b								Tfcp2l1							BC068281
			Mtmr12								Gnal							Tcea3
			Rhox9								Pvrl1							Sema6a
			Hist1h2bg								Gprc5b							Csrp2bp
			Vmn2r45								Mylk3							Rsg1
			C78339								1700020N01Rik							Mob1a
			Lin7c								Heg1							Spc25
			Nsun6								Ube2j1							Nrk
			1700026D08Rik								Grik3							Best3
			Unc5b								Ctnna3							Il17re
			Guk1								Whamm							Amigo3
			Ccnk								Htr5a							Gm13083
			Vmn1r45								Lingo1							Lin28b
			Prss53								F2r							Atad1
			Gpr152								Zfp935							Vangl1
			Pard6b								Rnf144b							Ankrd63
			Lrrc20								Lrp3							Dnm1
			Otog								Mroh3							Pard3
			Crip3								Xlr5b							Srrm4
			Stc2								Xlr5c							Ptch1
			Atl2								Lrp4							Nufip2
			Pygo1								Fem1b							Notch2
			Plod1								Jmy							Dusp26
			Snx27								Ints1							Cyp2d37-ps
			Bahd1								Lrtm2							Prkab2
			Gjd4								Gtf3a							Iqgap2
			Kcns1								Srf							Zfp408
			Ace								Inpp5d							Pnpla1
			Nipal3								Gpr61							Lrrc59
			Mnt								Ncoa1							Atg14
			Srd5a2								Il17rd							Ctdspl2
			Kcnj16								Lins							Nrcam
			Brcc3								Cp							Ppp1r26
			Tmem123								Fam118a							Pum1
			Stag1								Otop3							Hoga1
			Tmem150b								Mia3							RP23-180L12.5
			Dmtf1								Gpr176							Fosl1
			Adig								Bcap29							Nrg1
			2010109I03Rik								Atp8a2							Foxn4
			Fam196b								Wnk1							Fam84a
			Dbpht2								Mgea5							Marveld2
			Alpk3								Pla2g4c							Podxl
			Vps54								Pcnt							Ccdc109b
			Hemgn								Htr1a							Aktip
			Erlin2								Ptplb							Frs2
			Agfg2								Ube2m							Wrb
			Colgalt2								Slc28a2							Atxn7
			Fgf23								Efna4							Spopl
			Gm6086								Tecrl							Gpr116
			Chst11								Stard8							Ccpg1
			Wnt3								Map3k6							Zfp12
			Zfp619								Trp53							Szrd1
			Fgf3								Tmem185b							Camsap1
			Ndst1								Slc38a1							Mmp17
			Nacc2								Fam171b							Olfml3
			Zcchc4								Hmga1							Thrb
			Mgat3								Wdr4							Pacs2
			Tssc4								Cybrd1							Trim29
			Parp16								Ly6g5b							Cdk15
			F12								Krtap5-3							Musk
			Rgs3								Klhdc4							Galnt2
			Rab44								Snap29							Apol8
			Gosr2								Trim25							Ctnnal1
			K230010J24Rik								Gm2042							Hectd1
			Thbd								4732440D04Rik							Eme2
			Msantd4								Pacsin1							Camkv

miR-199-3p	miR-29-3p	miR-486-5p	miR-361-3p	miR-122-5p	miR-425-5p	miR-136-5p	miR-96-5p	miR-142-5p	miR-19-3p	miR-141-3p	miR-770-3p	miR-335-5p	miR-200-3p	miR-194-5p	miR-183-5p	miR-341-3p	miR-182-5p	miR-370-3p
			Lsm14b								Tmub2							Taf1
			Mat2a								Cyp4f15							Ccdc64
			Cebpg								Reep6							Smug1
			Themis3								Rab3il1							Nfxl1
			Smok3a								Pcdh11x							Snx21
			Piezo1								Slc46a2							Ttbk1
			Farp1								Flt3							Gm9754
			Btnl2								Eif4e1b							Itga3
			Sox2								Hmga1-rs1							Eea1
			Cic								Smad5							Pde10a
			Bcl2l2								Zbtb4							Pafah2
			Hmga1-rs1								Rcan3							Lemd2
			Clstn2								Klhl3							Mrps10
			Zkscan16								Elfn2							Aadacl3
			Spred2								Pou2af1							Stard8
			Ajap1								Itpk1							Daam1
			Zmat1								Ace							Dnajc18
			Abi3bp								Lrrc4b							Otud6b
			Dlg4								Zfp418							Slc37a2
			Kctd20								Col5a2							H2-M9
			Dennd6b								Uox							Edn2
			Cd59a								Fcho2							Purg
			Ick								Scamp5							Pigr
			Zbtb26								Slc35f6							Spata2l
			Pcyox1								Rab44							Ears2
			Rgs8								Nova2							Cntnap1
			Ivl								Tecpr2							Pxdn
			Cspg5								Gprasp2							Gm5423
			Mfsd12								Ghrhr							Pdx1
			Szrd1								Evx2							Ttpal
			9130011E15Rik								Xpo6							Srebf2
			Rbm43								Clec2l							Kcng3
			Itgb8								Slc11a1							D630039A03Rik
			Zfp58								Zbtb7a							Cul1
			Rasal1								Fam189a1							2310011J03Rik
			Ei24								Oxr1							Nudcd3
			Cdh4								Dnm2							Sfrp1
			Rbfox2								Fzd3							Pikfyve
			Ppp1r26								Cadm2							Tnrc18
			Vstm2b								Tfam							Slc4a1ap
			Fgd1								Atg9b							Shisa9
			Nutf2								Bbs1							Nudt6
			Glis2								Cep170							Crim1
			Pcgf3								Tymp							Fam132b
			Lypd1								4931409K22Rik							Itpk1
			E2f1								Tbx18							Gm20388
			Acot2								Map2k7							Crispld2
			Padi3								Osbpl5							Tmem164
			Clstn3								Cdh4							Vps33a
			Nuak1								Dag1							Sesn2
			Rasal3								Yy1							Slc2a1
			R3hdm1								Ttc39b							Rabif
			Entpd3								S1pr1							Wnt6
			Psca								Ces2e							Camkk1
			Pcdhb12								Dhcr24							Csrnp3
			Zfp266								Phf21b							Luzp1
			Peak1								Dtx4							Trim55
			AI314180								Clec4a3							Sec1
			Slc7a11								9330151L19Rik							Plxnb3
			Dctn2								Tgm3							Rfx5
			Arhgdia								Asic4							Mtif3
			Gm17067								Fancc							Tctn1
			Ankrd46								Mtfp1							Mc1r
			Arhgef10l								Ap2s1							Fam46a
			Gpalpp1								Rnf182							Snph
			Wwc1								Asb6							Bloc1s5
			Ripk1								Hoxc5							Sgta
			Kcnd3								Htra3							Nlrp12
			Epb4.1l4b								Numbl							Gm10097
			2010315B03Rik								Usp2							Stk38l
			Diras2								Gnpat							Tprkb
			Ensa								Gm9970							Mat1a
			1600014C10Rik								Cenpb							Iqgap1
			Gm10083								Ky							Myo6
			Ak2								Acap3							Rgs7bp
			Ss18l1								Glyctk							Coq9
			Picalm								Nos1							Smad1
			Nrep								Tln2							Sox12

miR-199-3p	miR-29-3p	miR-486-5p	miR-361-3p	miR-122-5p	miR-425-5p	miR-136-5p	miR-96-5p	miR-142-5p	miR-19-3p	miR-141-3p	miR-770-3p	miR-335-5p	miR-200-3p	miR-194-5p	miR-183-5p	miR-341-3p	miR-182-5p	miR-370-3p
			Ccdc88c								6720489N17Rik							S100z
			Chmp3								Mllt10							Dusp22
			Acadsb								Sh3glb1							Ncdn
			Aldh1b1								Irf4							Drd5
			Rps9								Spata2l							P2rx3
			Hrh3								Iscu							Hdgfrp3
			Pkib								Vstm2l							Edn1
			Agpat9								Zfp319							Gm20826
			Serpinh1								Jmjd6							Aen
			Trak1								Abhd11							Ptger1
			Cmah								Dnm1							Ap5z1
			Ccdc22								Grk1							Clgn
			Arsa								Rnft2							Cstf2t
			Arfgap2								Cntn2							Sele
			Mef2c								Phf21a							Capn13
			Sdccag3								Ralgps2							Cxcr5
			Snx20								Jmjd8							Ptprn
			Irx6								Eif6							Tfap2c
			Gyk								Gid4							Gga1
			D630044L22Rik								Ctsd							5031439G07Rik
			Gpr3								Map6d1							Alkbh5
			Tnpo1								Gnaz							Amer2
			Adra2b								Pole							Adra1a
			Polr1a								Ppp1r13b							Slk
			Npr3								Limd2							Tgds
			Rims1								Angel1							Ankrd45
			Adamtsl4								Slc12a7							Cd300lb
			Slc35a2								Cachd1							Plcd3
			Poll								Vimp							Pknox1
			Bnipl								Ccdc178							Syt15
			Rnf43								Tmem30b							Mob1b
			Pik3r1								Lyplal1							Hdlbp
			Cask								Add1							Zfp69
			Asb16								Pggt1b							Wnt10b
			Pax3								Fam149a							Traip
			Bcr								Tep1							Tmem132c
			Gpr116								Mfge8							Zdhhc15
			Dtwd2								Elk1							Jak2
			Kcnj10								Srpk2							Klc2
			Phlpp2								Cd244							Unc13d
			Mcoln3								Kat2b							Sertad2
			Dync1i1								Prlr							Myzap
			Kcna1								Ids							Pbxip1
			Cntnap5c								Agtr2							Det1
			Pvr								Klk13							Afap1
			Arhgap28								Brap							Fnbp1
			Onecut2								Mast3							Psen1
			Cela1								Lpgat1							Them7
			Stard10								Crb3							S1pr5
			Eif6								Zfp128							Nacc1
			Dolpp1								Usp5							Ccr9
			Nova2								Nxf3							Asxl1
			Tnfrsf14								Ccnd2							Aldh5a1
			Gpr17								Sh3bp5l							Tgif2
			Exoc3l4								Fam217b							Snapc1
			4930486L24Rik								Dsg2							Pskh1
			Mgat4c								Wdr78							Sh3gl1
			Egr2								Fbxl20							Gpr39
			Fam83a								Gm14124							Pdcd11
			Skint6								Myocd							Trp53bp2
			D430019H16Rik								Hmg20a							Gfra1
			Cdc42bpb								Auh							Wwtr1
			Dscr3								Srpk3							Lrrc38
			Gramd1c								Pdyn							Wnt7a
			Dmpk								Slc8a1							Grb10
			Ptpn21								Chst8							Pml
			Coprs								Gm5464							Abi2
			Slc29a2								Grap							Ulk1
			Zfp810								Pik3c2g							Rap1gap
			Dhrs1								Mrgpre							Thsd4
			Pdlim3								Bdkrb2							Artn
			Map2k6								Cbx6							Kdm1b
			Cdca4								Mast2							Alg11
			Rdh1								Rpn1							Mrgprx2
			Hdac7								Batf							Pitpnm2
			Hoxd3								Gucy1b3							Chrnb2
			Slc38a4								Pdk2							Kdm5b
			Tusc5								Lman2l							Paip2b

miR-199-3p	miR-29-3p	miR-486-5p	miR-361-3p	miR-122-5p	miR-425-5p	miR-136-5p	miR-96-5p	miR-142-5p	miR-19-3p	miR-141-3p	miR-770-3p	miR-335-5p	miR-200-3p	miR-194-5p	miR-183-5p	miR-341-3p	miR-182-5p	miR-370-3p
			Thy1								6430571L13Rik							Mphosph9
			Fubp1								Zcchc14							Efcc1
			Gpr61								Abl1							Msl3l2
			Cnnm1								Gm15409							Scn3b
			Inhbb								Med11							Tbkbp1
			Tsen54								Rassf2							Cntln
			Ppp1r10								Samd14							Pip4k2c
			Psrc1								Txnl4b							Palm2
			Zswim8								Mpped1							Cyb561
			Stil								Zfyve27							Ciita
			Pign								Camk2b							Tktl2
			Arid3a								Wac							LHB
			Tmc3								Galnt7							Dand5
			Gm21885								Mok							Sult1b1
			Siae								Scube1							Fam117a
			Trim25								Lamp5							Kpna7
			Calm3								Ftsj1							Jph3
			Tmem104								St6gal1							Gm53
			Gm5878								Gm14288							Rbm15b
			Astn2								Sox1							Orc5
			Mef2b								Gm53							Gpn1
			Otud1								2900011O08Rik							Usp39
			S100z								Ccl22							Ap3m1
			Ccdc134								Col4a3bp							Ptov1
			4930426L09Rik								Incenp							Krt75
			Tmprss6								Lactbl1							Atg9b
			Lss								Slc12a1							Slc23a2
			Cr2								Gm4477							Dctn1
			Fkbp14								Cml3							Ldlrap1
			Tead1								Fstl3							Rcor3
			Uroc1								Kcnj5							Klrg2
			Lsamp								Neu3							Oxsr1
			Liph								Zxdc							Rnf165
			Rnf125								Tnik							Pbx1
			Pigv								Fmod							9330171B17Rik
			Etv4								Clmp							Trpm5
			Gstm3								Drp2							Rasa3
			C330007P06Rik								Fignl2							Fgfr3
			A930009A15Rik								Wnt5a							Naa25
			Atg4c								Pmm1							Ppp2r5e
			Yod1								Hapln2							Setd1a
			Lonrf1								Spib							Thoc3
			Mapre3								Aoc3							Atp1b4
			Slc30a1								Pgpep1l							Samd11
			Gm13083								Rtn4ip1							Gtf2e2
			Prss35								Vat1l							Klrk1
			Creb1								Otud4							Mcfd2
			Slc45a3								Tpcn2							Ahcyl2
			Chrm4								Nkain3							Csnk1g1
			Slc6a6								Gfap							Thrap3
			Txnip								Osbp2							1700037C18Rik
			Tmem50b								Tma16							Cobl
			Atxn7l3b								Ubr3							Ces2e
			Vav2								Alpk3							Mmp14
			Atp11a								Zfp787							Rimbp2
			AI606181								Clec11a							Hdac11
			Msl3l2								Onecut1							4833424O15Rik
			Gm10282								Wdr6							Gm14391
			Atp8b2								Dennd2d							Adamtsl1
			Per2								Ppp1ca							Fam76b
			Rptor								Ubap2							Ankrd17
			Oas1g								Gm11128							Gm15440
			Ptch1								Ftcd							Rorc
			Rs1								Gm13083							Wdr73
			Dio3								Ddn							Ldlrad2
			Gramd4								6430548M08Rik							Gm10083
			Klrb1c								Cd99l2							Lrrc3
			Foxk2								Rbbp6							Phactr4
			Efnb1								Lurap1							Zc3hav1
			Camk1d								Col12a1							Arhgef3
			Cotl1								Btn2a2							Cdc6
			Pde3a								Msantd1							Sap18
			Zdhhc9								Ddx31							Atp6v1e1
			Gpr123								Usp22							Endov
			Ermp1								Kmt2a							Pnma2
			Spi1								Sufu							Pcdh15
			Mief2								Tmem2							Chst3
			Porcn								Arid3a							Bmp6

miR-199-3p	miR-29-3p	miR-486-5p	miR-361-3p	miR-122-5p	miR-425-5p	miR-136-5p	miR-96-5p	miR-142-5p	miR-19-3p	miR-141-3p	miR-770-3p	miR-335-5p	miR-200-3p	miR-194-5p	miR-183-5p	miR-341-3p	miR-182-5p	miR-370-3p
			Arl4c								Casr							Nos1
			Nudt18								Scn5a							Myh6
			Hoxb6								Enpp7							Ctla2a
			Islr2								Clcf1							Gss
			Syde2								Oprm1							Gm7903
			Vkorc1l1								Stk40							Gm5128
			Sesn2								Sec31b							Clvs1
			Pnpla8								Ccdc163							Ddhd2
			Pak6								Rnf24							Fpgs
			Tnfrsf13c								Osbpl6							Plp1
			Ighg2c								Foxp1							Vps37c
			Igfbp5								Fgf1							Ric3
			Axl								Sephs2							Tmed8
			4930563D23Rik								Nhlrc4							Pdap1
			Slc14a1								Tnip2							Ipcef1
			Lmx1a								Cdc14a							Zfp553
			Setbp1								Sun1							Poglut1
			Snta1								Zfyve19							A530084C06Rik
			Synj2bp								Sae1							Six1
			Elavl2								Fgf3							P2ry2
			Tifa								Carf							BC035044
			Dusp18								Rab2a							Osbpl6
			Nfx1								Fbxo3							Pald1
			D17H6S53E								H2bfm							Psd2
			Rhob								Tmem104							Ralgds
			Napa								Pou3f2							Syt7
			Nkain2								Nid1							0610030E20Rik
			Casp2								Robo2							Tfap2b
			Rwdd3								Prrt2							P2ry13
			Pnmal1								Xxylt1							Wbp2
			Aadacl3								Fer							Pramel3
			Pgm2								Asxl2							Gm15023
			Pqlc2								Serpina9							AV320801
			Zbtb18								Lmtk3							Grpel2
			Pde6h								Pcx							Mb21d1
			Gm10644								Brca2							Tnfaip1
			Slc35a3								Trpm3							Itpr3
			Yipf2								4930538K18Rik							Scrn1
			Tcf20								Trim58							Ddn
			Pmaip1								Fam65c							Rest
			Dqx1								Mest							Gm3495
			Lrrc16b								Caml							Tnfrsf22
			Wnk1								6330419J24Rik							Rspry1
			Pmp2								Klhl23							A830018L16Rik
			Ccdc42b								Sntb1							Podxl2
			Pla2g2d								Tbx20							Chmp4c
			Slc22a7								Stau2							Zfp945
			Onecut1								4732456N10Rik							Plod1
			E2f5								Serf2							Cdh6
			Furin								A330021E22Rik							Med13
			BC007180								Synj2bp							Gcm1
			Cd34								Ube3b							Nipal3
			Sh3bp2								Gna12							Rcsd1
			H2-T3								Zfp324							Hyal6
			Hoxb3								Kcne3							Spty2d1
			Gm7534								Rprm							Mbd5
			Ppfia3								Gm9949							Scd2
			Nmt2								Trib3							D5Ertd579e
			Btn1a1								Mycl							Fam198a
			Nfat5								Osbpl10							Cntnap5a
			Vti1a								Krtap5-5							Kbtbd12
			BC106179								Prc1							C2cd2l
			Tbc1d23								Sbno1							Spryd3
			Wdfy1								Ppfia4							Tmem41b
			Arhgap17								Pcdh12							Mcur1
			Pnpla6								Metrnl							Lbr
			Gpr173								Mul1							Senp2
			Slc4a5								Apod							Bard1
			2210418O10Rik								Tmem200b							Snx9
			Mrgpre								Fzd5							Bend4
			Mettl7a2								BC021891							Mtmr1
			Myh11								Pou2f1							Siglec15
			Gm14296								Nav1							Tbc1d4
			Rara								Snapc1							Cecr6
			Kdelr2								Gtpbp2							Map3k1
			Als2								Dtl							Nvl
			Klhdc7a								Prox1							Spr
			Vps18								Asic1							Qrfp

miR-199-3p	miR-29-3p	miR-486-5p	miR-361-3p	miR-122-5p	miR-425-5p	miR-136-5p	miR-96-5p	miR-142-5p	miR-19-3p	miR-141-3p	miR-770-3p	miR-335-5p	miR-200-3p	miR-194-5p	miR-183-5p	miR-341-3p	miR-182-5p	miR-370-3p
			Klhdc8a								Slc46a3							Hs6st1
			Tgtp1								Dennd2a							Hoxb3
			Rqcd1								2510009E07Rik							Ush1g
			Camk2b								Acvr1b							Evi5l
			Ier5l								Ctcf							Slc25a45
			Tm9sf4								Mafk							Stk10
			Cd3eap								Isoc1							Pknox2
			Tm4sf19								Med15							Fads1
			Spry4								Clpp							Clic6
			Tmem207								Oas1g							Prdm10
			Enam								Catsper2							Sema3c
			Zbtb22								Lif							Grik4
			Dlx3								Fam219a							2810021J22Rik
			Syvn1								Map1lc3b							Narg2
			Mettl7a1								Vat1							Eaf1
			Wdr72								Def8							Stx17
			Urad								Tgfa							Lman2
			E330009J07Rik								Mex3a							Kdm6b
			Tldc1								Cyp2c40							Zfp599
			Rassf4								Ackr2							Gm10384
			Gramd1b								Arl14ep							Htra3
			Plce1								Adra2b							9230104L09Rik
			Lrrtm2								Dnajb14							Cd40
			Dnaaf3								Tctn3							Tmem130
			Atf5								Rsrc1							Plcl2
			Hpn								Slc41a2							Steap4
			B3galt1								Nfatc2							Lhx6
			Srf								Col8a2							Nek6
			Kdelr1								Caly							Mapk1ip1l
			Kcnk10								Ppp1r7							Tnfsf15
			Zmiz2								Rab22a							Fzd7
			Arhgap6								Abca8b							A830010M20Rik
			Slc30a2								Hjurp							Gm16253
			Gm10032								Dennd3							Ngef
			Plxnb2								Mbd5							Ffar4
			I830077J02Rik								2510049J12Rik							Adck4
			Tmem200b								Wrb							Kcnk5
			Skida1								Arhgap44							BC023829
			Dcp1b								Tmem43							Tlr5
			Gla								Megf8							Erlin2
			Slc6a11								Cidea							Lbh
			Il2rb								Drd2							Ano5
			Rorb								Enpp6							Osbp2
			Creg2								Lbx1							Slc25a42
			Gm11711								Tyro3							9230104M06Rik
			Kcnip2								Cacng5							Lats2
			Ptpn14								Nlrp6							Marcks
			Spred3								Stim1							Sstr4
			H2-Q4								Dnajb12							Rell2
			Evi5l								Gimap1							Asb7
			Cntfr								Igfals							Thbd
			Gm10134								Arhgap17							Acvr1b
			Elmod2								Myl9							Def8
			Dhx34								Gbx1							Ctnnd1
			Kdm2a								Fam131b							Nedd4
			Sdad1								Pop1							Pan3
			Nrp2								Brk1							Dgkz
			Urm1								Gimap6							Pdk4
			Ror2								Ubox5							Ubn1
			Gpr132								Cc2d1b							Spry4
			Itga5								Ccdc82							Bcl2l2
			Oas1c								Prox2							Tmem72
			Piwil2								Tspear							Carns1
			Zfp831								Nudt7							Tbx20
			H6pd								Oog1							Cep162
			Cd274								Pofut1							Sgsm1
			Pdk2								Nlrp12							Kctd16
			Ppp3ca								Gm996							Phf21a
			Mycl								Dyrk1b							D7Ertd443e
			Hnrnpul2								Dkk3							Map2k5
			Kcnd1								Gm10436							Gpd1
			Cdx1								Spns1							Kctd21
			Cd46								Naaladl1							Ermn
			Slc7a12								Dctd							Arhgap35
			Tbc1d10b								Rtn4rl2							Irgq
			Qsox2								Loxl2							Zfp831
			Tm9sf3								Hoxd4							Atp2b4
			Gnal								Arfip1							Stx1b

miR-199-3p	miR-29-3p	miR-486-5p	miR-361-3p	miR-122-5p	miR-425-5p	miR-136-5p	miR-96-5p	miR-142-5p	miR-19-3p	miR-141-3p	miR-770-3p	miR-335-5p	miR-200-3p	miR-194-5p	miR-183-5p	miR-341-3p	miR-182-5p	miR-370-3p
			Lca5								Crk							Fam53c
			Col8a1								Tob1							Avl9
			Rbm5								Ergic2							Arf3
			Fer1l6								1300002K09Rik							Gm4631
			Ccdc89								Nfatc4							Rfx1
			Adra1a								Mamstr							Lrsam1
			Jag1								Pex5l							Ilf2
			Tmem43								Rpl21							Zfp616
			Klrb1b								Rnf43							Kcnq1
			B4galt1								Klf10							Ctnnbip1
			Sgpp1								Lipt2							Cphx1
			Ext1								Impdh1							Cphx3
			Sppl3								Mavs							Cphx2
			Lmo3								Slc22a23							Rnf215
			Rabif								Slc16a7							Rab31
			Btbd2								Vps13d							Brms1l
			Tcf7								Serpinb6e							Rhobtb2
			Six3								Dgcr6							Zpld1
			Lpin3								Emx1							Mylk3
			Lrrc23								2610044O15Rik8							Rbbp8nl
			Ptprs								Gabra3							Rgs12
			Arhgef2								Map6							Znf512b
			Zfp207								Acp2							Cep131
			Oprd1								Gm10837							Arid2
			Vstm2a								Synj2							P2ry4
			Hmga1								Acox1							Ilf3
			Atf6b								Il4ra							Tmem97
			Gemin7								Lin28a							Oraov1
			Mfsd6								Gm17174							Gm12830
			Mc1r								Zmynd15							Il11
			Eif5a								Cenpm							Ctdsp2
			Atxn1								Ccno							Rps6kc1
			Larp1								Lypd3							Gdpd5
			Fnbp4								Tnks							Jrk
			Crtc1								Pms2							Zfp780b
			Hrasls								Pyroxd2							Il13ra2
			Fam101a								Thap2							Ms4a4d
			Hmgcs1								Ndufaf4							Htr1d
			Tspear								Slc16a13							Cep85l
			Aldh7a1								Get4							Trim58
			Sema4d								Epm2a							Rxfp1
			Trim71								Osbp							Ppp6r3
			Brwd3								Dbnl							Rsbn1l
			Trabd								Scx							Fign
			Pa2g4								Gfm1							Swt1
			Ptk6								Fzd7							Ccdc115
			Bai2								Lingo3							Sertad4
			Erbb4								Twistnb							Gas7
			Fibcd1								Mdga1							Amer3
			Mob3c								Cacna1c							Tmem184a
			B430305J03Rik								Krba1							Tspan7
			Phf19								Brd9							Apc
			Eif3j2								Ccdc55							Yeats2
			Tmem30a								Pdhb							Timm9
			Bcl2l11								Ap2b1							Mettl6
			Eng								Dctpp1							Mansc1
			Intu								Cish							Ccdc38
			C2cd4c								Hepacam							Pigh
			Tbx6								Stac							Tnk2
			Ctf2								Fchsd1							Muc6
			Rexo1								Dctn1							Tmem8c
			Npnt								Trim14							Polg
			Cdc37								Gm16485							Cyb5r2
			Glyctk								Rimbp3							Hvcn1
			Bmx								Ncln							Naa15
			Pomgnt1								D130043K22Rik							Akap1
			Prune2								Arhgap4							Ubr2
			Ak1								A230065H16Rik							Pax7
			Ears2								Pxt1							Haao
			Carf								Pwp2							Speer4c
			Agk								Wipi2							Adam10
			Eif4h								Nfix							Efnb3
			Tgtp2								Prnd							Slc16a5
			Cntn4								Gm28045							Atg2b
			Plekhb1								Klhl33							Sestd1
			Gm7030								Naa25							AI464131
			Rwdd4a								Cdan1							4930572O03Rik
			Tlr6								Rps19							Nmt2

miR-199-3p	miR-29-3p	miR-486-5p	miR-361-3p	miR-122-5p	miR-425-5p	miR-136-5p	miR-96-5p	miR-142-5p	miR-19-3p	miR-141-3p	miR-770-3p	miR-335-5p	miR-200-3p	miR-194-5p	miR-183-5p	miR-341-3p	miR-182-5p	miR-370-3p
			Bmp10								Ap1ar							Poldip3
			Rasef								Qsox1							Hira
			Dapp1								Cdh3							Camk1g
			Sh3bp5								Chmp6							Edar
			Atf7ip								Rsc1a1							CTNND1
			Inpp4a								Fcho1							C4b
			Brf1								Rdh12							Ublcp1
			A530064D06Rik								Tcp11							Fbxl21
			Cdca7								Usp36							Grip2
			Khdrbs1								Atrn							Serinc5
			Hist2h2bb								Lsm11							Shroom4
			Rfx5								Snx32							Knstrn
			Slfn9								Apex2							Pdf
			Golga7b								Cyp2d22							Trim17
			Nfam1								4930578C19Rik							Ccr5
			Scaf11								Lhfpl2							Adrb3
			Atmin								Fam13a							B3galnt2
			Fam105a								Peli3							Cs
			Tmem86a								Lztfl1							Pde7b
			Uprt								Fam214b							Zfp397
			Tacr3								Dnlz							Adam19
			Cd300lh								Sept8							Csmd1
			Crtc2								Etv6							Trip4
			Nxnl1								Exosc10							Hydin
			Htr3a								Gm13023							Zpr1
			Inpp5j								2410141K09Rik							Gm10521
			Klhl24								Mief1							Rasl10b
			Cyth1								Atxn1l							Tns4
			Pdzd8								Itpr2							Pcmtd1
			Cryz								Ogfod1							Mex3a
			Ddx6								Rhbdf2							Dcun1d3
			Map3k13								Gypc							Thbs2
			Snap91								Sstr4							Tollip
			Fam210b								Atg13							Ube2l3
			Fgfr3								C1qtnf6							Rap1gds1
			Clcnkb								Slc35b4							Lrrc16b
			Cyth4								Rims4							Birc5
			Plk3								Polr3f							Scd4
			Gm11710								Rrp12							Phf20l1
			Efr3b								Dpysl4							Gpld1
			Bean1								Slc39a9							Gna11
			Zfp605								Cygb							Mfap1a
			Osbpl10								Tmod3							Fxyd5
			Pgrmc2								D630002J18Rik							Eml4
			Snap29								Dnmt3a							Tgfbr2
			Fam115c								Nek9							Wdr26
			Rab11fip1								Kcne1l							Cir1
			Rasl12								C87977							Jmjd6
			Gm5784								R3hdm2							Amer1
			Spen								Zfp747							Dsc3
			Pcdh15								Vwc2							Speer4d
			Efhd2								Ltbp1							C1rl
			Grip2								Hes7							Yipf6
			Nkx6-3								Vps4b							Ifnlr1
			Ms4a4b								Nptx1							Efnb2
			Mapk1ip1l								Zbtb1							Ahnak2
			Iba57								Gca							Kif1a
			Aacs								Sesn3							Klf3
			E2f3								Ptpn1							Slc22a5
			Gbp4								Gm11733							1810011H11Rik
			Brox								Slco2a1							Tfdp2
			Angpt2								Casz1							Zfp111
			Baz1b								Osgin1							Erbb3
			Usp51								Lyl1							Jmjd8
			Rnf114								Cabp2							C130026L21Rik
			Isg20l2								Gm9799							Cep164
			Men1								Rpl18a							Mtss1
			Gpd1								Gimap3							Wwc2
			Akap6								Ttll4							Igfbp6
			Glt1d1								Phactr3							Cog4
			Atg2a								Gcsam							Ntm
			Treml2								Ccdc87							Etv6
			Pip5k1a								Traf3ip1							Srgap1
			Dennd5a								Harbi1							Tmem181a
			Man1c1								Snapc4							Tnfsf10
			Fadd								Srsf1							Zfp870
			6330408A02Rik								Bcl7a							Rapgefl1
			Ube2l6								Wbp1l							Atp8b3

miR-199-3p	miR-29-3p	miR-486-5p	miR-361-3p	miR-122-5p	miR-425-5p	miR-136-5p	miR-96-5p	miR-142-5p	miR-19-3p	miR-141-3p	miR-770-3p	miR-335-5p	miR-200-3p	miR-194-5p	miR-183-5p	miR-341-3p	miR-182-5p	miR-370-3p
			Tspyl4								Nipal3							Serpinf2
			Dnajc5								Herc2							Bmf
			Tcf24								Klk14							Bmp8a
			Fam173b								Ptgdr							Supt6
			Myo1c								D430041D05Rik							Ccdc141
			Caprin1								1700020L24Rik							Aqp6
			Hcfc2								Serpine1							Sumf1
			Atoh8								Dcaf7							Irak3
			Mip								Liph							Stx4a
			Usp44								Astl							Ypel2
			1810041L15Rik								Prss12							Cdk5r1
			Faim2								Ift57							Fam177a
			Stx5a								Trub2							Errfi1
			Tnrc6b								Slc39a13							Clec1a
			Dlx2								Ppp2r2c							Ap2a2
			Foxl1								Phospho2							Map2k3
			Glce								Sftpb							Eif2s3x
			Fnbp1								Tgm2							Patl1
			Dsc3								Tmem150a							Pwwp2a
			Unc5a								Fut11							Plxnb1
			Ntmt1								2300003K06Rik							Tpcn1
			Apba1								Tex9							4833427G06Rik
			Cd2bp2								Brms1							Lrrc27
			Hp1bp3								Eri3							Myf6
			Csnk1g1								Tprgl							Upk3b
			Stx1b								Meis2							Gm7168
			Duox2								Npffr1							Rnf166
			Fbxl7								Paqr9							Gm5415
			Ntn1								Agpat3							Dpcr1
			Med13l								Thnsl1							Msx3
			Cdv3								Skida1							Dzip1l
			Adamts15								Dctn5							Clcn5
			Vil1								Tnfrsf1a							4930571K23Rik
			Nptxr								Mbtps1							Fbln2
			Antxr1								Asb18							Atf7ip
			Tyk2								Uggt2							Ntn1
			Ccdc14								Aldh1b1							Rabgap1
			Unc13a								6030419C18Rik							Abca8b
			Grb10								Sap30l							Yipf1
			Dsg2								Kcnc1							Gdf5
			Syngap1								Dync1li2							Rps19bp1
			Lig4								Foxm1							Aldh1a2
			4632415L05Rik								Hecw2							Herc3
			Tgfb3								Ccdc42b							Mlkl
			Gabra1								Lin52							Mthfsd
			Slc16a10								Mlkl							Chrm5
			Lrrc2								Lrrc8a							Dpysl3
			Nos3								Kcnn1							Spata2
			Rspo4								Gpr107							Cic
			Diap1								Abcc6							Usp20
			Vps37c								Magi1							Polr3a
			Stk10								Gdf11							Barhl2
			Arel1								Cds2							Lurap1
			Hip1								Edc3							Il2ra
			Arhgap29								4930453N24Rik							Klhl29
			Pi16								Ldhc							B230217C12Rik
			Prdm10								Itk							Kdsr
			Ssx2ip								Mylip							Socs7
			Cntn5								Zfp709							Kcna2
			Kcnab2								4930523C07Rik							Zdhhc5
			Plod2								Pik3cb							Ing3
			Zfp618								Snrnp25							Asap2
			Zdhhc2								4930481A15Rik							Glp1r
			Clpb								Ccdc124							Clcn7
			Arid5b								Cdk20							Klhl33
			Mtfr1								Pianp							Itpkb
			Cbfa2t3								Zfp697							Cdx4
			Siglec1								Tctn1							Foxo4
			Malt1								Nrbp1							Tpd52l1
			Ap1g1								Carm1							Exoc3l2
			Exoc8								Sfn							Gpr114
			Cpne1								Fam207a							Fam219a
			Chd3								Pgm2l1							Gm1070
			Prr18								Elf4							Zfp292
			Rfx2								Jmjd4							AI606181
			Foxh1								Zfp526							Aqp11
			Polr3g								Ago2							Ube2q2
			1110057K04Rik								Pus1							Gm9821

miR-199-3p	miR-29-3p	miR-486-5p	miR-361-3p	miR-122-5p	miR-425-5p	miR-136-5p	miR-96-5p	miR-142-5p	miR-19-3p	miR-141-3p	miR-770-3p	miR-335-5p	miR-200-3p	miR-194-5p	miR-183-5p	miR-341-3p	miR-182-5p	miR-370-3p
			Smek1								Dcaf11							Ky
			Arhgap11a								Kcnq1							AB041806
			Ttc7								Il6st							Ube3b
			Stt3b								Kdm5b							Fgd3
			Slc35f3								Slc35a2							Sipa1l1
			Olfr606								Abt1							Cnnm3
			Tmem229b								Rxfp1							Zdhhc2
			Hnrnpa0								Znrf1							Gapvd1
			Gm26596								Iffo2							Ppcdc
			Gm4980								Il6ra							Bckdk
			Cabp2								Mrps26							Gsap
			Tmem51								Pygm							Zdhhc3
			1110025L11Rik								Syt3							Chrna7
			Whsc1l1								Gm2163							Dffb
			Dyrk1a								Plekhf1							Ganc
			Enc1								Cngb3							Mecom
			Nat8l								4933426M11Rik							Shisa6
			Cdh13								Cbx4							Adap2
			Nupl1								Tada2b							Celf4
			Emp2								B3galt5							Cluh
			Vegfa								Dnajc28							Secisbp2l
			Pfas								Pax2							A930016O22Rik
			Gsg1l								Zgrf1							Gpr62
			Mavs								Colq							Srsf1
			Zc3h12a								Pax5							Smarcd2
			Pvrl1								Hpgds							B3gntl1
			Sh2b2								5330417C22Rik							Gid8
			Ptprt								Tmem25							Gpkow
			Col26a1								Slc5a3							Diras2
			Srcrb4d								Pabpn1l							Cdkl4
			Rnf144a								Elfn1							Sept8
			Jph2								Tomt							Slc4a9
			Mepce								Bgn							Ran
			Ush1g								Cdh24							Mpdz
			Mapt								1110032F04Rik							4933430I17Rik
			Bicd2								B3gat1							Gm10644
			Chmp6								3110001I22Rik							Tmc7
			Api5								Dmgdh							Tmem80
			Tmem184a								Ceacam19							Cd33
			Zbtb7a								E030019B06Rik							2310022B05Rik
			Abi1								Rgs3							Gpr89
			Wdtc1								Lrrc66							Pcyt1b
			Oxsm								Glul							Fam78a
			Gpr107								Mapkapk3							Nptxr
			Gpr182								Sh2d4b							Ddx3y
			Rad23b								Igsf23							Atoh8
			Dcstamp								Slc25a26							Fbxl7
			Ptrf								Cap1							Snx18
			Wnt3a								Cpd							Atp10a
			Chrm1								Atf6b							Gm10699
			Evx2								Vps37d							Mtfmt
			Ttf1								Ogfod2							Pex12
			Zfp946								Ncam1							Gm10654
			Gjd3								Abhd2							Stk32a
			Dpp10								Mapk1ip1l							Tmem186
			Arhgap19								Slc1a2							Bmp7
			Tmem143								Mroh2a							Klhl28
			Erbb3								Rita1							Pip5k1a
			Znhit6								Tmem135							2810459M11Rik
			Tfap2b								Tango6							Paics
			Edem1								Nt5e							Prrx1
			Fam120c								Irs3							Prkrip1
			Rhbdd2								Kctd20							1500002C15Rik
			Stat1								Trim16							Pxn
			Grin2b								Acvr2a							Nefh
			Dennd1a								Klhl30							Armc8
			Unc5c								Igf1							Frmd5
			Tarsl2								Gm11938							Krba1
			Ndufa4l2								Mcpt4							Polr1a
			Efna2								Cherp							Kcnj15
			Zfp667								Fam134c							Snf8
			Traf3								Adig							Inhbb
			Add2								Prdm14							Dhx35
			Igf1								Crtc1							Fkrp
			Fam222b								Rtn4rl1							Hes2
			Fbxo32								Atf7							Klf16
			Lpar1								Dusp15							Arhgef2
			Nhs								Bend3							Opa1

miR-199-3p	miR-29-3p	miR-486-5p	miR-361-3p	miR-122-5p	miR-425-5p	miR-136-5p	miR-96-5p	miR-142-5p	miR-19-3p	miR-141-3p	miR-770-3p	miR-335-5p	miR-200-3p	miR-194-5p	miR-183-5p	miR-341-3p	miR-182-5p	miR-370-3p
			Wtip								Fbxw17							Psmb11
			Zfp867								Smarcc1							4931406P16Rik
			Gabra3								Necab3							Zkscan8
			Rorc								Enah							Smarcc2
			Zfp386								Gm10608							Dock9
			Sectm1b								Gdap2							Gm13030
			Abi2								Zbtb42							Ccdc58
			Manbal								Tapt1							Pou4f3
			Cldn25								Wdr86							Bex4
			Kctd16								Plbd2							Cpt1b
			Fbxo4								Gba2							Ogdhl
			Iars								Gstm6							Ifit2
			Samd7								Ccdc127							Rtl1
			Cmip								Sft2d2							Vezt
			Ube2g2								Mmachc							Celf3
			Syt4								Rassf8							Col4a3
			Dna2								Plekhg2							Klhl42
			Mylk3								Ap5b1							Mapk6
			Prpf18								Erich5							Drp2
			Smg1								Rrp36							Nrxn1
			Sox5								Pde4d							Naa30
			BC068281								Sh2b3							Cplx4
			Gm3629								Kdm5c							Abhd12
			Nlrx1								Aff3							Atp6v0e2
			Nim1k								Tceb3							Rab3a
			Syt1								Vps53							Gm10134
			Efna4								Mrgprf							Hmga1-rs1
			Glyr1								Copa							Rab5b
			Chrna2								Ptp4a3							Kcna1
			Unc119b								Zfp14							Adra2b
			Fras1								Dnaaf3							Tgm2
			Ncoa5								Mapre1							Grin2a
			Hoxa6								Rasa3							Lama3
			Tet3								Grb2							Tirap
			Gpr146								1700049G17Rik							Lypla1
			Zdhhc18								Plcd4							Fanca
			Slx1b								Slamf8							Il1f8
			Atf6								Ndufaf3							Srbd1
			Fzd9								Stoml3							Gen1
			Srsf12								Slc30a2							Limk1
			Lgi3								Lonrf2							Gipr
			Fgf4								Xdh							Nol6
			Rassf8								Ccdc71							Midn
			Evi5								Cdc37							Tm9sf3
			Hpgds								Toe1							Tenm2
			Hist1h2bj								Tsen54							2610018G03Rik
			Dtx4								Ak2							Pmepa1
			Ncoa1								Pramel1							Ttc22
			Gnai2								Morf4l1							Rbl2
			Grap2								Flot2							Agap1
			Nosip								Rnf157							Lrrk1
			Tmed4								Plin4							Mbp
			4930427A07Rik								Fndc1							Cadm3
			Mecp2								Gm10097							Gm7247
			Cerk								Prdx2							BC089491
			Phf8								Fam129a							Tada1
			2610008E11Rik								Hp1bp3							Brf2
			Abca3								Scrib							Armc2
			Cggbp1								Prpf31							2510003E04Rik
			Arl10								Kirrel3							Ippk
			Ckap4								Nucb1							Fkbp5
			Zc3h12d								Flt4							Ppp1r3d
			Plxnd1								Prr16							Gm10797
			Col10a1								Dlgap4							Xpo6
			Slc28a3								Ngfr							Gm6034
			Snx29								Snai1							Pou2af1
			Pnmal2								Ccnt1							Sept2
			Tor4a								Rhob							Gpr182
			B3gat2								Glrx3							A130010J15Rik
			Rsg1								Hist1h4m							Tmem86a
			Pla2g2c								Adck2							Tmod2
			Gpc1								Zc3hc1							Igdcc4
			Esrrg								Mthfsd							Ikbke
			Trim24								A830073O21Rik							Vav1
			Upk1b								Kansl1l							Prkca
			Fzd3								Mturn							Gle1
			Skint4								Glb1							Stk25
			Lrtm1								Ankef1							Ighmbp2

miR-199-3p	miR-29-3p	miR-486-5p	miR-361-3p	miR-122-5p	miR-425-5p	miR-136-5p	miR-96-5p	miR-142-5p	miR-19-3p	miR-141-3p	miR-770-3p	miR-335-5p	miR-200-3p	miR-194-5p	miR-183-5p	miR-341-3p	miR-182-5p	miR-370-3p
			Krt81								Begain							Nim1k
			Ccdc130								Tmod2							Man1a2
			Aak1								Exog							Dclre1c
			Larp4								Ears2							Erf
			Zfand2a								Slc25a12							Cep89
			Ap3m2								Smox							Gpr68
			Sap18								BC017158							Brca2
			Psap								Gltscr2							Scyl1
			Glul								Heatr2							Slc22a21
			Onecut3								Vwa5a							Naa40
			Pappa2								Mea1							Lox
			Vps25								Gm10447							Zfp706
			Snx30								Zfp799							Mr1
			Myo18a								Pigm							Ajap1
			Dyrk1b								Ttc30b							Qk
			Tle3								Ten1							Csf1
			Pgpep1								Nkap							Pla2g2f
			Srp72								Zfp618							Fam65b
			Herc4								Cldn19							Ttf1
			Rgs20								Dok4							Vgll3
			Kirrel								Zfp3							Acvrl1
			Stk4								Ncaph2							Pom121
			Pnpo								Ube4a							Zfp866
			Loxl3								Ikbkg							Usp31
			Thra								Krt35							Sntg1
			B3galt5								Gm10600							Jade1
			Gm12695								Oaz3							Ctla2b
			Rerg								1700029H14Rik							Cdr2
			Ptdss1								Cnot3							St6gal2
			Wnt5b								3110052M02Rik							H2-T22
			Adat3								Ssx2ip							Tnip1
			Shc4								Grik2							Snx33
			Srl								Ldlrad1							Tmem104
			Klhl42								Hist1h2be							Speer4a
			Farsa								Slc26a9							Elavl1
			Gm14221								Zfp839							Ntrk1
			Insm1								G6b							Srd5a1
			Kif3c								Adam19							Ppm1a
			B3gat1								Cnnm2							Slc25a23
			Fbxl16								Gm10775							Rab11fip2
			Gmps								Emc10							Mrpl13
			Scamp1								Gstm7							Apitd1
			Phb2								S1pr3							Gm20721
			Bmp8a								Faf2							Pear1
			Bcl6b								C4b							Cyp4f37
			Nt5c1a								Hoxb1							Gm10471
			Slc6a2								Padi3							Fem1a
			Mid1ip1								Tuft1							Ppt1
			Slc25a30								Serinc1							Mrc2
			Tex261								Anxa7							Lman1
			Slc25a27								Flad1							Maneal
			Gpr22								Bcas3							Tnip2
			Glp1r								Extl3							Arf2
			Gimap3								Ripk1							Fuk
			Map7d1								Prrx2							Ubash3b
			Nyx								Ano6							Mbnl3
			Arrb1								Tmem145							Etnk2
			Cdc25a								Pou3f3							Plod2
			Lysmd4								2810459M11Rik							H2-Bl
			Tfeb								Grin2a							Dusp9
			Ept1								Gm4980							Trib2
			Clgn								Gm10645							Bloc1s6
			Neu1								Nrg2							Frk
			Cyb5r3								St8sia5							Foxred2
			Csk								Kdm6b							Oxr1
			Mapk12								Slc4a9							Fam167a
			Hoxb8								Abcg1							Pcdh12
			B4galt6								Papd7							Prps1l3
			Nudt21								Samd10							Fam107a
			3110082I17Rik								Htr6							Ppm1k
			Fstl4								Katnb1							Arhgef6
			Lrp3								Trpv1							P4ha1
			Gse1								Gtf3c4							Mfsd6l
			Syndig1l								Gm11444							Gm7276
			Capn5								Tmf1							Gm614
			Cdc42ep4								Tuba4a							Mfrp
			Ndrg1								Sema3g							Bbs1
			Slc33a1								Ppfia3							Fam63b

miR-199-3p	miR-29-3p	miR-486-5p	miR-361-3p	miR-122-5p	miR-425-5p	miR-136-5p	miR-96-5p	miR-142-5p	miR-19-3p	miR-141-3p	miR-770-3p	miR-335-5p	miR-200-3p	miR-194-5p	miR-183-5p	miR-341-3p	miR-182-5p	miR-370-3p
			BC005624								Lynx1							Pdgfb
			Gprc6a								Usp12							Myom3
			Klhl13								Asb10							Clmn
			Srsf10								Mocs1							Ocel1
			Bcorl1								Fbln5							Cdc34-ps
			Pggt1b								Scp2							Vmn1r55
			Nags								Med19							Trmt61a
			Homer2								Spata3							Lysmd4
			Snx9								Dynll1							Caps2
			Soat1								Krt33b							Rassf4
			Adcyap1								Adcy5							Pcyt1a
			Krt80								Cdx1							Tmem178
			Kpna4								Dnajc30							Lmtk3
			Hmgn1								Ogn							Slc12a2
			Ap3m1								Foxred1							Rlim
			St3gal2								Dbndd2							Pcdhga12
			Itpkb								Ccdc64							Map2k6
			Cntnap2								Pgm3							Lrrc66
			Zmynd19								Pikfyve							Cd99l2
			Col25a1								Uggt1							C730034F03Rik
			Gm4952								Hoxd11							Hist2h2bb
			Zfp148								Pnpo							Faah
			D3Ertd254e								Pde6h							Cd83
			Patz1								Pex14							Krt84
			Cyp4f37								Slc25a38							Ptk7
			Arhgef9								Fosl2							Ifnar1
			Khdc1c								Chst2							Fam20a
			Rnft2								Zbtb3							Setd5
			Nus1								Ulk4							Ttn
			Kcnk3								Slc39a8							Ugt2b34
			Gosr1								3110082I17Rik							Asxl2
			Id1								Acsf2							Carkd
			Cdc14a								Oxct1							Gm17019
			Epm2aip1								Cacfd1							Lypd6
			Tbx20								Gcnt7							Pank3
			Fam84b								Bace1							Vwc2l
			Zfp933								Hmgcs2							Bnc1
			3110039M20Rik								Csnk2a1							Bhlha15
			Gmeb1								Naif1							Slc24a3
			Flrt1								Clptm1							Pik3r3
			Rbm27								Napa							Slc8a1
			Calcrl								Zglp1							Dcx
			Inhba								Cxcl15							Thada
			March8								Dlgap2							Cdca2
			Trim33								Itih4							Fam222b
			Pde10a								Krt42							Phlpp1
			Ablim1								Ydjc							Dynll2
			Slc16a6								Foxred2							Zfp691
			Mmp17								Slc9a4							Tpmt
			Pex26								1700102P08Rik							Rcn2
			Ppm1l								Tmem199							Crk
			4931440F15Rik								Dda1							Kifc2
			8430419L09Rik								E130012A19Rik							Fscn1
			Asah2								Arid5a							Zfyve19
			Smad2								Aph1a							Cenpb
			Nudt12								Nkain1							Atp5g1
			Snupn								Rbfox3							E130308A19Rik
			Foxp1								Parp16							Ddx46
			D130040H23Rik								A530084C06Rik							Siglec1
			Rpa1								Tada2a							Hus1
			Kcng1								Hhatl							Zbtb25
			Atp2b3								Akap11							Fbxl17
			Kcnj3								Plod1							Trpm2
			Cd180								Scarb1							Vps13a
			Npcd								Hcar1							Dnajc30
			Igsf11								Nos1ap							Btg1
			Pea15a								Rab5b							Socs5
			Gimap5								Mertk							Prr33
			Ikzf3								Inpp1							Zfp874a
			Mmab								Mettl7a2							Slc38a11
			Chd9								Mettl7a1							Chst10
			Ksr1								Gm10676							Slc7a8
			Mon1b								Dedd2							Eng
			Cbx7								Mapk13							Gm16223
			Hist2h4								3110070M22Rik							Gcom1
			Kank1								Serf1							Art1
			Sp140								Met							Speer4f
			B3gnt6								Scara3							Trmt6

miR-199-3p	miR-29-3p	miR-486-5p	miR-361-3p	miR-122-5p	miR-425-5p	miR-136-5p	miR-96-5p	miR-142-5p	miR-19-3p	miR-141-3p	miR-770-3p	miR-335-5p	miR-200-3p	miR-194-5p	miR-183-5p	miR-341-3p	miR-182-5p	miR-370-3p
			Tbc1d2								Npcd							Arhgef7
			Pdp2								Ybey							Chrna4
			Fam46c								Ascc3							Cables2
			Exoc3l2								Xrcc3							Lmod1
			Kcnq5								Caln1							Phf21b
			Zyx								Mgat4a							Aph1a
			Med9								Abca6							Glud1
			Serf2								Rhov							Cd93
			Adam12								Gm12695							Efna2
			Bmpr1a								Atf2							Zfp39
			Ppp1r16b								Diras1							Ybx3
			Chst3								Elovl7							Ankrd54
			Arhgap35								Pura							Grin2d
			Lemd1								Gsc							Tbccd1
			Abcb10								Aip							Mertk
			Lrrc14b								Ifih1							Speer4b
			Mink1								Iglon5							Dusp16
			Cutal								Socs2							Mgat3
			Ffar3								Cd46							Lphn2
			Hhat								Rnf41							Lats1
			Hecw1								Tdh							Trim24
			Gal3st2								Mgat5							Uhrf1bp1
			Nrip3								2310039H08Rik							Abtb2
			Snx19								2210407C18Rik							Ppif
			Dnm3								Mad2l1							Dusp28
			Xylt1								Ppp1r14a							Gm9758
			Frs2								Ccdc89							Rnf114
			Pan3								Map3k5							H2bfm
			Ankrd12								Mrps23							Gbas
			Glra2								Lep							Cd46
			Sv2c								Hdhd2							Slain2
			Mc5r								Ercc2							Inf2
			Ptgfrn								Ttll6							Ammecr1l
			Clcn6								Csk							Slc38a10
			Snrpe								Lyzl6							Gabarap
			Ttll8								Serpinb9							Vmn1r56
			Frmd4b								Dhh							Gjb4
			Cntnap1								Eddm3b							Smco3
			Gbp6								Brd7							Prr15l
			Sned1								Sik1							Sel1l
			Ksr2								Llgl2							Zfp703
			Zfp382								Tmem234							Pomt1
			Zfp599								Card11							Pramel1
			Rps7								Ndufa4							Champ1
			AI661453								Tgm6							Ptchd1
			Socs4								Cyb5rl							Inpp4a
			Matn3								Elane							Os9
			Mfsd5								B4galnt2							Zdhhc23
			4930539E08Rik								Pabpn1							Prok1
			Rfx3								Ppcdc							Mtrr
			4933408J17Rik								Mrpl55							Zfp120
			Poc1a								Amz2							Fmnl2
			Usp46								Xrn1							Il5ra
			Zbtb25								Sept11							Ppp2r5a
			Pyroxd2								Iqcg							Sh3bgrl2
			Myrip								Tmem194b							Tnfrsf13c
			Nlrp1b								Agap1							Zfp488
			Cdc7								Dpp6							Syt12
			Oxsr1								Slmo1							Prpf19
			Iqcg								R3hcc1l							Casp9
			Nupr1l								Pag1							Pphln1
			Il10rb								Klf13							Dcdc2a
			AI593442								Ttll9							Ddr2
			Bsn								Nrsn2							Ccdc114
			Fbxo31								Psg16							E130208F15Rik
			Thap3								Nsfl1c							Heph
			Ticrr								Bcl6b							Cox10
			Cdc26								Map1b							Syngr4
			Stxbp5								Asb2							Tor1b
			4931440P22Rik								Bhlhe41							Tmem63a
			Mfrp								Clstn2							Atf2
			Sorcs3								Limd1							Klhl25
			Cblb								Slc38a10							Sv2a
			Gpr110								Oxsr1							Sh3bp5l
			Adipor2								B430305J03Rik							Golga7b
			Mcidas								Zfp397							Zcchc24
			2510039O18Rik								Lhcgr							Nos1ap
			1110004F10Rik								Irf1							Speer4e

miR-199-3p	miR-29-3p	miR-486-5p	miR-361-3p	miR-122-5p	miR-425-5p	miR-136-5p	miR-96-5p	miR-142-5p	miR-19-3p	miR-141-3p	miR-770-3p	miR-335-5p	miR-200-3p	miR-194-5p	miR-183-5p	miR-341-3p	miR-182-5p	miR-370-3p
			Rims3								P2rx4							Traf3ip1
			Thoc2								Shc1							Mapk1
			Eid2								Wtap							Aagab
			Sbno1								Hhip							Fancc
			Lilra5								Igf2bp1							Gdf2
			Apoo								Mrpl48							Cog6
			Tmem19								1700017B05Rik							8430419L09Rik
			Siglec15								Snai3							Tmem87b
			Fat3								Trim63							Zbtb21
			Il1rapl1								Tmprss6							Nisch
			Gpsm1								Ccdc25							Col9a3
			Snx24								Txlnb							Ung
			Bmp7								Mmrn2							Zcchc17
			Tom1l2								Tm4sf4							Iqce
			Dnajb5								Gm9913							Gab3
			Atrn								Pa2g4							Adamts20
			Akap5								Med16							1810026J23Rik
			Slc23a2								Hmgn2							Mapre2
			Lgr4								Aldh3a2							Sept9
			Prkar2a								Arhgap24							Ctbp2
			Plin1								Slc3a2							Cc2d1b
			Cd209c								Dppa3							Lnpep
			Ino80d								Fam193b							Tgfbrap1
			Actrt3								B3gnt9							Mllt3
			Gm9994								Lrch2							Mesdc2
			Cdk5r1								Smpd3							Ylpm1
			Shc2								Gopc							Rarres2
			Fam43b								Mxd1							Il10ra
			Tmem25								Stk25							2410131K14Rik
			Pla2g5								Fam124b							Atmin
			Lonrf2								Tgfb1							Cap1
			Tcaim								Noxred1							Gm9938
			Plekho1								Hdgfrp3							Pex11a
			G3bp1								Oxnad1							Bmp1
			Irgq								Dnase1l2							Krtap7-1
			Fgfr1								Abcg8							Dnali1
			Mllt3								Astn2							Tcp11
			Fbxl20								Sectm1b							Gm1979
			Surf6								Ing3							Kcnt1
			Zdhhc15								Dnajc1							Dnajc1
			Grin2d								Atp6v0a2							Jade2
			Arhgef3								Cdk6							Eif5b
			Ppapdc3								Hmces							Zfp597
			Gata4								Zbtb45							Aanat
			L3mbtl2								Qrfp							Fam207a
			1700012B07Rik								Iyd							Cdca8
			Dpep3								4930529M08Rik							Myoz3
			Setd1a								Psmd1							Acp1
			Zfp442								Tmem5							Map3k9
			Metap1								H2-T22							Shroom3
			Acacb								Rala							Fasn
			Arrdc3								Zfp113							Tpbpa
			Selt								5830473C10Rik							Gdi2
			Adora3								Ugt3a2							Tmub2
			Rab39								Gfra1							1110032A03Rik
			Nphp3								Arhgdia							Srp54b
			Taf1d								Fam101b							Mrpl47
			Fryl								Lime1							Eri1
			Zbtb6								Ddx54							Btbd2
			Dkk2								Sp110							Cops7b
			Gm13225								Fscn1							Dpy19l3
			Tmem181a								Fam136b-ps							H2-M3
			Zfp553								2610524H06Rik							Noxo1
			Lipg								Polr3h							Prmt7
			4933427D14Rik								Zc3h10							Cacnb2
			Tppp								Prr15l							Sdc4
			Lfng								Armc5							4933427D06Rik
			Gba								6030445D17Rik							A930004D18Rik
			Itga9								Pcsk1							Fzd2
			Eif5a2								Zfp26							Impg2
			Vash2								Csrnp1							Tmem56
			Sptbn1								Fmo5							Nek2
			Lrp6								Aco1							Mau2
			Hjurp								Slc26a4							Cdc34
			Arhgef39								Hoxb13							Plcxd1
			Sarm1								Arih2							Gm21244
			Pdlim5								Sptlc2							Mroh3
			Ubn1								Mllt3							Cnot8

miR-199-3p	miR-29-3p	miR-486-5p	miR-361-3p	miR-122-5p	miR-425-5p	miR-136-5p	miR-96-5p	miR-142-5p	miR-19-3p	miR-141-3p	miR-770-3p	miR-335-5p	miR-200-3p	miR-194-5p	miR-183-5p	miR-341-3p	miR-182-5p	miR-370-3p
			Camkv								Cenpq							Gm9887
			Mamstr								Psme3							Dbnl
			Ikbkg								C3ar1							Mob3a
			Fbxw8								Eps8l1							Ets1
			Rlim								Blm							Runx1
			Slc5a4b								Maoa							Cenpl
			Fgl2								Gm906							Gm9949
			Mkrn2								Tbx2							Alpk3
			Wbscr17								Ptpn11							Ndrg1
			Adam28								Gm15440							Fer1l6
			Lrrc71								Epha5							Smad9
			Paqr9								Hist1h3d							Ltbp2
			Gm14124								Slc2a2							Cpsf3l
			Akap13								Aadat							Wbscr27
			Clec9a								Mpi							Dnajb6
			Klf9								Gm20604							Vstm2l
			C130074G19Rik								Timm22							Stc2
			Ipcef1								Sec14l3							Dda1
			Hmx1								Wdr20							Cd82
			Vapb								Prss57							Kcnj11
			Neurl3								Sppl2b							Vat1
			Gabbr1								Brip1							Gm9979
			Mlph								Cul4a							Zfp697
			Abcd1								Narg2							Abat
			Pbx1								Fam26e							Zfand4
			Gm28040								Alkbh8							Tacr2
			Ablim2								Acpp							2610034B18Rik
			AU022252								Tmem86b							Gnptab
			Artn								Ugt8a							E2f3
			Fbln7								Ftsj2							Heatr2
			Slc8a3								Dusp13							Prdm2
			1700013F07Rik								Amfr							Kcnj10
			Cd300a								Prss54							Lin52
			Tram2								Tcp1							Gm10647
			Flt1								Prr5							3830406C13Rik
			Hist1h2ah								Cd109							Acox1
			Gck								Ggt7							Tha1
			Prpf4b								Ctu2							Txlna
			Clstn1								Pax8							Il17ra
			Pik3ip1								Col6a1							Slc10a4
			Srd5a1								Adamts7							Atp8a1
			Arih1								Stat1							Nln
			Sh2d1b2								Dhx15							Slc39a8
			Bnc2								Serpina3c							Ankrd44
			4833424O15Rik								Arl5c							Pgap1
			Mast4								1700067K01Rik							Myo18a
			Ppp3r1								Syt6							Gm5938
			Gm5627								Lmbrd1							Tpbpb
			Tmem9b								Ankrd9							Sptlc1
			Sytl5								Gatad2b							Gm5641
			Rasal2								Il16							Cdc42bpg
			Impg2								Syn2							Cyp4f17
			Ccdc157								Emc3							Adamtsl5
			Il12rb1								Nkiras1							Thap2
			Galnt4								Cant1							Gpm6b
			Aplp1								A430033K04Rik							Fbxw8
			Cir1								3110062M04Rik							Islr2
			Wnt8b								Vcpip1							Mb21d2
			Cbx2								Tacc2							Mok
			Unc5d								Fam193a							Tshz2
			P2rx7								Nkd1							Pex14
			Kpna1								Apoo							Enpp1
			Prdm1								Hspb7							Psd4
			Shank3								Ube2u							Ulk4
			Gemin2								Lbp							Hecw2
			Zkscan7								Apol6							Rpl12
			Elovl6								Bace2							Cbx1
			Cldn19								2310014L17Rik							Haus8
			D16Ertd472e								Pafah1b3							Zwint
			Tspan14								Bcl2l13							Zfp60
			Pip4k2a								Acsl6							Banf1
			Mief1								Zfp607							Ldlr
			Rere								Catsper4							Mettl2
			Plekha8								Pdap1							Ankrd34a
			Sox6								Arfgap2							Dhx33
			Smad7								Dgcr2							Fktn
			Med1								Wars2							Src
			Gm17349								Sh2d4a							Gabrb2

miR-199-3p	miR-29-3p	miR-486-5p	miR-361-3p	miR-122-5p	miR-425-5p	miR-136-5p	miR-96-5p	miR-142-5p	miR-19-3p	miR-141-3p	miR-770-3p	miR-335-5p	miR-200-3p	miR-194-5p	miR-183-5p	miR-341-3p	miR-182-5p	miR-370-3p
			Hmgn2								Inca1							Icosl
			Igfbpl1								Slc10a6							Leprel1
			Ppp6r3								Scn2b							Mtap7d3
			Ttc23								Map2k3							Keap1
			Sema6d								Stox1							Xaf1
			Zfp871								Amdhd2							Pcyt2
			Set								Chrna1							Bend7
			Snx18								Plekho2							Magt1
			Napepld								Tbx15							Ccdc67
			Sftpd								Mrgprh							Entpd7
			Gucy1a2								Fbxo25							Efna5
			9830147E19Rik								Eef1a2							Mark4
			Csrnp3								Kcnj9							Zfp810
			Nup210								Medag							Gm17349
			Psd2								Cdc34							Gm5616
			Map2k7								Shq1							Nhp2l1
			Fam19a2								Cript							Baiap2l2
			Meis1								Tcp11l2							R3hdm4
			Spib								Aplf							Ctsf
			D030025P21Rik								Emc7							Mef2a
			Pigl								Clec1a							Actr2
			Pde7a								Ipo9							Slc27a6
			Fads2								Grik4							Zfp592
			Socs7								Erp29							Atp8a2
			Chac1								Xpo7							Sco1
			BC024978								Cyp2b13							Slc35e3
			Tbc1d8b								Mdm4							Ptprs
			Cyp26b1								B4galt1							Cbln1
			Zbtb4								Large							Chst2
			Zic4								Gm20521							Heatr5b
			Tlr5								Fbxl22							Gpr132
			Gpr26								Gprin2							Il16
			Nfib								Dnm1l							Hcar1
			Scara3								Cox6b2							Grsf1
			Igf2bp1								3110040N11Rik							Slc27a4
			Klb								Ap3s2							Rab39
			Acvr1b								Slc30a3							Sh2d2a
			Ttpal								AI182371							2610044O15Rik
			Slc38a6								Cers4							C2cd5
			Lpp								Vezt							Pla2g15
			Padi2								Eif4g2							Nyap1
			Syn3								Kdelc1							Nsun3
			Sstr5								Ern1							Slc4a1
			Epha8								Gmfg							St6galnac5
			Sema5a								Kcnt1							Vwa5b1
			Rhog								Comtd1							Edaradd
			Olfr464								Plekhg3							Epsti1
			Acp2								Aco2							Tmem120a
			Myo7a								Arhgap8							Polr2e
			Rab6b								Arhgef2							Map3k7cl
			Luzp1								Ctbp1							Il25
			Traf4								Adora1							Ttll11
			Eif1ax								Strada							Kpna1
			Gm3739								Gm7932							Slc31a1
			Wdr7								1700029I15Rik							D19Bwg1357e
			Zfp345								Atg16l2							Slc8a2
			Rdx								Npepl1							Snx6
			Cux1								Cyp2b9							Osbpl2
			Tifab								Bok							Daam2
			Zfp418								Chmp4b							Sufu
			Zfp276								Pirt							Scn4b
			Ubald2								Plcl2							Add2
			Tns1								Gm21743							Zfp382
			Tsn								Sycp2							Lonrf3
			Ago4								Alpk1							Cyld
			Slc22a15								Dock6							Aff3
			Map3k1								Wdr36							Mfsd10
			Edem3								Dpt							Esp1
			Pxylp1								BC049352							Sgol1
			Adora1								Srpx2							Rbck1
			Tmem245								Mtrf1							Slc25a26
			Thap2								Akr1e1							Atf3
			Stxbp1								Lrwd1							Rgs17
			Elac1								Trim24							Dpm2
			Gprc5b								Fam89b							Gm9875
			Ago2								Minpp1							G3bp1
			Gm12216								1810009A15Rik							Nipal4
			Tmem132e								Cyp2a12							Acap2

miR-199-3p	miR-29-3p	miR-486-5p	miR-361-3p	miR-122-5p	miR-425-5p	miR-136-5p	miR-96-5p	miR-142-5p	miR-19-3p	miR-141-3p	miR-770-3p	miR-335-5p	miR-200-3p	miR-194-5p	miR-183-5p	miR-341-3p	miR-182-5p	miR-370-3p
			Fzd10								Prkdc							Rnf2
			Map2								Srek1ip1							Lgi4
			Tmem63c								Wsb2							Ttc14
			Cybrd1								Nptxr							Nkapl
			Slitrk5								Stx6							Sde2
			Svop								Zfand5							Tmem150c
			Syt9								Nbl1							Lpin1
			Fam160b2								Fam46c							Mturn
			Pcdhga12								Zc2hc1c							Smad3
			Tmem132c								Zfpm1							F13a1
			Crlf2								Spop							Fam188b
			Pbxip1								Gldc							Cttnbp2nl
			Rnf165								Pgm5							Gm11444
			Nacc1								Rhbdd2							Morc2a
			Lin7a								Ttyh1							Wfdc13
			Zfp397								Akr1c12							Zfand3
			5730409E04Rik								Cir1							H2afy
			Gad2								Spc25							2310002L09Rik
			Aldh5a1								Mtmr6							Dpep1
			Trim8								Tmem119							Rrp1
			C1qtnf9								Clip1							Rnf141
			Slc7a1								Lrrc14							Men1
			Ccdc93								B9d1							Slc44a2
			Slc39a9								Abcb11							Wdr11
			Aifm2								2410015M20Rik							Athl1
			BC052040								Hnrnpul2							Cacna1g
			Gatad1								Phb2							Guf1
			Kcnh2								Mfng							Cbx8
			Nol6								Eif2ak1							Snrnp200
			Tspan9								Smg7							Gdnf
			Pdzd4								Nfia							Kcnh5
			Gm10638								Vapb							Espl1
			Hist1h3f								BC021785							Tspear
			Hnrnpa1								4632415L05Rik							Map2k2
			Lmnb1								Btrc							Arid5a
			Rrn3								Smim13							Thsd7a
			Btg2								Hoga1							Sept11
			2210407C18Rik								Oscp1							Prrg3
			Tmem11								Ikbke							Ehd4
			Trove2								Rnf208							Serac1
			Tox4								Dpf3							Pik3cg
			Slc5a9								Dyrk2							Pet112
			Hus1								Acot13							Pfkp
			Anks4b								Sys1							Scyl2
			Prkrip1								Pkig							Gnrhr
			Lbh								Npsr1							Gm10354
			Mmp19								Nnmt							Tmem74b
			Dcp2								Ucp2							Mmp28
			Zfp36l3								Eef2k							D10Wsu102e
			Slc6a17								Afap1l1							3110082I17Rik
			Cacnb1								Rbm15							B3galt1
			Anks1								Cebpa							Cox15
			Slc12a6								Tspan9							St8sia1
			Pkdrej								Ormdl1							Pacs1
			Ttc9c								Tbcc							Parvb
			Nmt1								Cldn25							Cacng8
			Ric8b								Efnb1							Cacna2d4
			Aasdh								Rnase13							Wdtc1
			Rims4								Mlycd							Mroh8
			Slc9a8								Scimp							Wnt3
			Slc26a2								Magix							Mbtd1
			Itih5								Hip1r							Fn1
			Sufu								Preb							Rassf10
			Fam171b								Abcd4							Kif11
			Sla								Itga1							Bahd1
			Plp1								Rad51d							Mme
			Ube2h								Bnip2							Spidr
			Stk24								Lnp							Mtss1l
			Cacng3								Il15ra							Gpr107
			Adamts4								Wbscr28							Tspan14
			Gcnt4								Lepre1							Ccl6
			Mmp24								Epb4.2							Wnt5a
			She								Tbc1d30							Ppp1r3b
			Mag								Pth1r							Zscan25
			Cdc25b								Grk5							Lgi1
			Upk3b								Zfp385c							Agtrap
			Nkx6-2								Cdo1							Prkab1
			Cln5								Mipep							B230359F08Rik

miR-199-3p	miR-29-3p	miR-486-5p	miR-361-3p	miR-122-5p	miR-425-5p	miR-136-5p	miR-96-5p	miR-142-5p	miR-19-3p	miR-141-3p	miR-770-3p	miR-335-5p	miR-200-3p	miR-194-5p	miR-183-5p	miR-341-3p	miR-182-5p	miR-370-3p
			Lman2								Tnk1							Barhl1
			Zfp109								Trim8							Blnk
			Dnajc30								Rell2							Shroom1
			Ovca2								Scgb1b27							Mfi2
			Myh14								Pbx2							Slc10a2
			Ttc22								Fsd2							Casc3
			Slc12a8								Rad54l2							Arel1
			Zcchc14								Mpdz							Fat1
			Ssr2								Mtpap							Mep1b
			Wscd2								Ehd1							Gm21976
			Col23a1								Fhl3							Gapt
			Lyrm9								Gm10399							Gck
			Glra3								Coq5							Pls1
			Pald1								Tmem126b							Ccnd2
			Polr3gl								Six5							Wsb2
			Zfand3								Fam69b							Gns
			Tulp4								Orc3							Ccdc97
			Cemip								Abcb8							6030419C18Rik
			Wipf2								Tmem106a							Prpf38b
			Msx3								Gnat2							Cdan1
			Naalad2								Cltb							Tars
			Chd4								H2-T10							Cnih3
			Rtn4rl1								Zfp85							Ccna1
			Ccser2								Trmt44							Hes5
			Igf1r								Klhdc8a							Ubl4b
			Myo9a								Dvl2							Aim2
			Timp2								Rhod							Cyp4f16
			Gltpd2								Vps26a							Rtfdc1
			Tmem198								Ank							Nbr1
			Sgsm2								Rcl1							Tor4a
			Draxin								Sema3f							Zfp612
			Rnd3								Impa2							Syt13
			A930033H14Rik								Cyp7b1							Lig3
			Fat2								Rnf217							Rgs20
			Cep41								Upk1a							Vangl2
			Tubb2b								Micu1							C130074G19Rik
			Fzd4								Zfp169							Lif
			Rybp								Proser2							Cmtr1
			Tor3a								Slc25a35							6330403A02Rik
			Sox11								Rnf122							Ier3ip1
			Rundc3a								Avpr1a							Ovol1
			Bmf								Tle1							Hbegf
			Idh3a								Anxa13							1700012B07Rik
			Ptprd								Gpr4							Naf1
			Ubqln1								Tspan13							Lsm11
			Amotl2								Arhgef7							Slc9a7
			Depdc1a								Tssk3							Mysm1
			G3bp2								Abcg3							Rufy2
			Ptx4								St6galnac4							Pten
			Leprot								Slc25a1							Rrp7a
			Rufy3								Taf10							Cdkn2aipnl
			Mkl1								Ccl25							Pxdc1
			Litaf								Gak							Pigu
			Rassf2								Map3k3							2810006K23Rik
			Casp16								Nmrk1							Fam169a
			Nufip2								Acbd4							Otud7b
			Tmem194								Sgcg							Tspan2
			Disc1								March9							Pomt2
			Ccdc184								Armc1							Arhgap10
			Ivd								Ushbp1							Cyp1a1
			Ptger1								P4htm							Rexo4
			Rgl3								Pced1a							Nr5a1
			Mars2								Il17ra							Tectb
			Gm6483								Dmxl1							Slc9a9
			Ggta1								Msmb							Dolpp1
			Mettl8								Nmral1							Zfp322a
			Tnpo2								Strbp							Olfr701
			Pex5								Rcc2							Krt80
			Phf6								Ift22							Poll
			Aplnr								Neil2							Nrg4
			Gif								Tsc22d4							D3Ertd254e
			R3hdm4								Sesn2							Dcp2
			Gm13011								Tgfb2							Intu
			Sort1								Amigo1							Tanc1
			Adcy6								Fam89a							Crb3
			Btbd19								Sugt1							Kxd1
			AW011738								Thada							Nkain3
			Nptn								Clcn3							Clec2i

miR-199-3p	miR-29-3p	miR-486-5p	miR-361-3p	miR-122-5p	miR-425-5p	miR-136-5p	miR-96-5p	miR-142-5p	miR-19-3p	miR-141-3p	miR-770-3p	miR-335-5p	miR-200-3p	miR-194-5p	miR-183-5p	miR-341-3p	miR-182-5p	miR-370-3p
			Camsap2								Tppp3							Cckar
			St6gal2								Tmem240							Oprd1
			4931406C07Rik								BC089597							Brd3
			Rmdn2								Zfp456							Plekhd1
			C4b								Ms4a8a							Calcoco1
			Ctdspl								Dtnb							Zfx
			Slc6a20b								F830016B08Rik							Fgf22
			Ppp1r18								Gng3							Ptprq
			Scn1b								Slc35e4							Obfc1
			Bhlha9								Tyms							Dtl
			Sec31b								Slc25a43							Nr1i3
			Elfn1								Nop9							Gm5447
			Dbnl								Zfp940							Fam109b
			Srrm4								D1Ertd622e							Tec
			Psd4								Bckdhb							4933440M02Rik
			Rfwd3								Hes2							Gpr110
			Yae1d1								Timm13							Dcbld1
			Camk4								Raf1							Setdb2
			Ppm1h								Ackr3							Cpt1a
			Lzts1								Tubb3							Smad7
			Hoxd4								Thap3							Atp6v0a2
			Ldlrad1								Acot8							Mkln1
			Klhl23								Fam84b							Galnt15
			Kat6b								Aasdhppt							Mrgpre
			Fam132b								Pparg							Map1lc3b
			Scrn1								Mmp15							Heatr6
			Mpped2								Tysnd1							Abcc6
			Dnajc1								Agtpbp1							Hjurp
			Paox								Il34							Fkbp15
			Dnmt3a								Plekhj1							Acbd3
			Tpcn2								Zfp629							Mrto4
			Il9r								Rnf126							Tnfrsf13b
			Plekhh2								Eno4							Atp6v1b1
			Ccdc116								Fam114a1							Serpina9
			Notch2								Ephb4							Atxn3
			E130012A19Rik								Senp6							Loxl3
			Krt222								Ccdc23							Cmtm3
			Dph1								Tpcn1							Apbb1
			Zadh2								Alad							Klhl21
			Rag1								Tlcd1							Gabrq
			Fam47e								Chtop							Wnt4
			Atp1b2								Mme							Samd10
			Acbd4								Pole4							Ppp4r2
			Pomt2								Prss23							Erich5
			Btnl9								Mtdh							Rplp0
			Ttll6								Gdf10							Col26a1
			Ccdc3								Bcat2							Hdac6
			Cdc73								Fga							Sept7
			Ftcd								Cct6b							Rnf126
			Tom1								8430408G22Rik							Fam71f2
			Zfp873								Fam210a							Srpx2
			Camkk2								Banp							Tspyl3
			Endov								Me2							Scml4
			Mxd1								Tbc1d2b							Cnga3
			Hells								Il20rb							Wdr78
			Scml4								Cd1d1							Mmp24
			Slc7a8								Ece2							Hfm1
			Ezh1								Fam228a							Rprd2
			Ptpn3								Plekho1							Wnt9b
			Ankrd22								Stx8							Scaf8
			Foxi3								Smim5							Bmp3
			P2rx5								Slc22a12							Dmrtb1
			Asxl3								Ncbp1							Nnt
			Gm14139								Gadd45gip1							Josd1
			Gpr165								Inpp5k							Azi2
			Tmem151a								1700019N19Rik							Naa60
			Musk								Lrrc24							Mfap1b
			Pafah2								Metap2							Cyp4f14
			Caln1								Pcdhb22							Gm5862
			Iqce								Cln8							Irf1
			Htr1a								Ppp1cc							Tal1
			Cacng5								Cpxm2							Dhdh
			Gm4944								Ntf5							Dmp1
			Mzf1								Pcmt1							Dgcr2
			Kcnt1								Txnl4a							Vmn1r59
			Zfp710								Itgb5							Tm7sf2
			Cdon								Ndufb9							Tmem240
			Scarb2								Chst13							Gm10638

miR-199-3p	miR-29-3p	miR-486-5p	miR-361-3p	miR-122-5p	miR-425-5p	miR-136-5p	miR-96-5p	miR-142-5p	miR-19-3p	miR-141-3p	miR-770-3p	miR-335-5p	miR-200-3p	miR-194-5p	miR-183-5p	miR-341-3p	miR-182-5p	miR-370-3p
			Plcb2								Pigp							Atxn7l1
			Ltn1								Lhx9							Rabgap1l
			Jup								Mbl1							Tmem67
			Jdp2								Gm4944							Rbms2
			Tbx5								Mrs2							Dcun1d2
			Tial1								Plcl1							Pygo2
			Akap2								Rgs5							Eps8l1
			Sertad2								Rpf2							Wipi2
			Rab18								BC005624							Dennd1b
			Mtss1								1700013F07Rik							Zfp869
			Neurl1a								Arap1							Stil
			Pde1b								Dcaf12							Pnpla8
			Kmt2a								Pex11a							Ict1
			Hepacam								Cidec							Slmap
			Eif2b5								H2-Bl							Arl2bp
			Leng1								Ism2							Galnt14
			6430571L13Rik								Srgn							Ovca2
			Mmachc								Galnt14							Sec14l4
			Igfbp4								Tsn							Unc5b
			Ar								Tomm40l							Fam109a
			Art4								Naa30							Rasgef1c
			Pcif1								Mef2d							Zkscan2
			Tpi1								Fam188b							Ptpn6
			Dst								Nrg1							Hspa4l
			Slit1								Aqp7							B3gnt4
			Pkd1								Zfp119a							Cidea
			Anapc16								Prkca							Klhdc8a
			Slc45a4								Btnl9							Tma16
			Mesdc1								Eif4ebp1							Rbm41
			Bcap29								Sgk2							Esyt2
			S100a7a								Cacna1d							Zscan30
			Asl								Ffar1							Ccne1
			Zfp654								Kcna4							Uroc1
			Zdhhc8								2810004N23Rik							Tspan9
			Trim41								Nt5c2							Srcin1
			Syne1								Cnp							Gprc5b
			Arhgap4								Siglece							Spocd1
			Crebbp								Arrdc1							Olfr70
			Sema3b								Lrrc58							Zfp41
			Ube2j1								Sgol1							Mib1
			Ap5z1								Zfand3							Ap2b1
			Bet1								Tsen2							Epb4.1l1
			Pramef8								B4galt4							Lbp
			Taf10								Ly6a							Rpl11
			Ehd2								Nudcd3							Bbx
			Ptcd3								Tmem255b							Prss57
			Mettl10								Pdlim1							Spata24
			Adipoq								C2cd2l							Gmeb1
			Nsmf								Gna15							Mepe
			Ncald								Amd1							Rpl38
			Zfyve28								Cyp2j9							Cdk18
			Rab22a								Traf5							Zfp72
			4930402H24Rik								Adprh							Fzd9
			Tyw3								Arhgef16							Slc25a32
			1810043G02Rik								Rad18							Kcne4
			Setd1b								Glo1							Pip4k2b
			6430548M08Rik								Nmd3							AU022252
			Cd244								Acn9							Diap1
			Klhdc10								Acyp2							Zak
			Tnik								Serpina3k							Rnf39
			Chrnb2								Cdc14b							Micu2
			5330417C22Rik								2310045N01Rik							Frmpd3
			Me3								Mterfd2							Fgf7
			Rgs1								Itih1							Lrp10
			Aldoc								Cask							Lpar1
			Ankrd50								Cnnm3							Slc18a2
			Prr12								Ubxn6							Sass6
			Kcnj12								Fcgr1							3632451O06Rik
			Adcy1								Armc3							Pld5
			5730455P16Rik								Gm17641							Cecr2
			Prokr1								Dynll2							Il4ra
			Sh3bp5l								Pnpla8							Ptprk
			Pcmtd1								Zbtb7b							Xrra1
			Cap1								Mvk							Gm6793
			Vcan								Serpinh1							Fam180a
			Emc8								Ndfip1							Hnrnpdl
			Usp50								Plod3							Plaur
			H2bfm								Cyp39a1							Ndufaf4

miR-199-3p	miR-29-3p	miR-486-5p	miR-361-3p	miR-122-5p	miR-425-5p	miR-136-5p	miR-96-5p	miR-142-5p	miR-19-3p	miR-141-3p	miR-770-3p	miR-335-5p	miR-200-3p	miR-194-5p	miR-183-5p	miR-341-3p	miR-182-5p	miR-370-3p
			Hoxb9								Zfp937							H2-T10
			Ubl7								Nfkb2							Ssrp1
			Kbtbd11								Inpp5e							Agpat2
			Vwa5b1								Tkt							Fam134a
			Znrf4								Cdca7l							5830462I19Rik
			Tiam1								Kynu							Eda2r
			F2rl3								Cd226							Ddx19b
			Antxr2								Tinag							Gpr174
			Gpm6a								Tceb1							Ror2
			Timm22								Pcbp2							Faim2
			Suds3								Ms4a4b							Dnm2
			Cd109								Fam136a							Angptl2
			Stk35								Fuom							Kalrn
			Slit3								Ssbp2							Mdga2
			Acnat2								Cr1l							Zfp940
			Syna								Trim39							Ccdc80
			Cyb5rl								Pi16							Iscu
			Slc2a10								Fxyd3							2010107G23Rik
			Ipmk								Slc44a1							Rnf11
			Car5a								Cyp4b1							Mlycd
			Il31ra								Mfsd7c							Lamtor3
			Tnr								Slc25a46							Ptplad2
			Rab3il1								Polr2e							Cd3eap
			Zzef1								A530053G22Rik							Kcnh4
			Arl15								Slc5a11							Hist1h3f
			Cmtm4								Krit1							Chpf2
			Chtop								Chmp7							Kat2a
			Slc25a15								Cntnap5b							March9
			Mef2a								Gramd3							Pcdh1
			Zfand4								Ephx2							Stam2
			Klf17								Cntnap2							Pak7
			Hipk2								Mxra7							2410141K09Rik
			Map4k2								Hddc3							Kcnj9
			Cx3cr1								Smc3							Ghsr
			Braf								Cd300a							Smc3
			Kif3b								Bri3bp							Rif1
			Proc								F10							Gm6792
			Pip5k1c								Sf1							1700048O20Rik
			Sorl1								Serpina3m							Zc3hc1
			Flt4								Stx19							Tmem246
			Swap70								BC021614							Crhr1
			Sstr1								Arv1							Gng12
			Gfra3								Chfr							Mgat1
			Pcdhb19								N4bp3							Urgcp
			Gpr171								Ybx3							Cx3cl1
			Myo10								Neurl1a							Synpo2l
			Nlgn1								Myl3							Ak4
			Hand2								Rps3a1							Igsf11
			Cdkn2b								Spa17							Klhl31
			Slc4a1ap								Spsb2							Phactr1
			Ank2								Gfod2							Trove2
			AI317395								Mapk8							Taf4b
			Sh2b1								Btc							Nup160
			Celf1								Nhej1							Phkg1
			Ercc4								Slc30a10							Hivep1
			Avl9								Naa16							Atg4c
			Sap30l								Slc4a3							Styk1
			Tfb1m								Mthfsl							Clec2g
			Zfp26								Dcp1b							Snai1
			Nr1i3								Oprd1							Slc38a6
			Os9								Cml2							Lynx1
			Pycr1								Mrps25							Kcna7
			Slc39a3								Gpi1							Crem
			Il17d								Cnrip1							Selk
			Irx1								Colec12							Dock6
			Il22ra1								Mccc1							Nkain1
			Ids								Rars2							Cdc16
			Ushbp1								Ms4a6b							Papl
			Etv3								Fh1							Ercc4
			Tulp1								Chchd5							Rab11fip5
			Tyms								Shisa4							Dnajb14
			Tmem110								Rrp9							Ptplb
			Atrx								Dram2							Gm4944
			Vps33a								Sh3rf1							Plcb4
			Paqr4								Commd6							Bcl2
			Prrc2b								Lyrm9							Nmt1
			Themis								Mterf1b							Itgb1bp2
			Uck1								Gch1							Rwdd4a

miR-199-3p	miR-29-3p	miR-486-5p	miR-361-3p	miR-122-5p	miR-425-5p	miR-136-5p	miR-96-5p	miR-142-5p	miR-19-3p	miR-141-3p	miR-770-3p	miR-335-5p	miR-200-3p	miR-194-5p	miR-183-5p	miR-341-3p	miR-182-5p	miR-370-3p
			Actr1a								Ulk3							Pdcl
			Taf12								Ccs							Pik3r2
			Runx1t1								Dusp7							Kcnrg
			Esyt3								Top1mt							Psme3
			Dlgap3								Wdr12							Kcnmb4
			Ppp2r5c								Adck3							B3gat1
			Lhx4								Hnrnpu							Rdx
			Mecr								Dhrs7b							Slc25a10
			Tbxa2r								Asb3							Rnf187
			Tab3								Loh12cr1							Adnp
			Col6a5								Exoc7							Slc12a5
			Fam217b								Vta1							D630045J12Rik
			Eme2								Cdh8							Ttc37
			Mok								Hint1							1700019N19Rik
			Esr2								Exoc5							Acot12
			Nek9								Mgat2							Cdyl2
			Arl5a								Pomt2							Lrrc9
			Tmod2								Atg4b							Ranbp2
			Vrtn								Set							Enah
			Naa11								Slc35g2							Spsb3
			Magi2								Trim55							M1ap
			Slc25a19								Naa35							Zfand2b
			Fli1								Slc6a18							Cd200
			Rhobtb1								Fam135b							Mfap4
			Ebna1bp2								Zfr							Fibin
			Palm2Akap2								Moap1							Hmga1
			Utp15								Fabp12							Ubac2
			Rbm34								Rpp38							Sema4g
			Ptplad2								Fam103a1							Ccar2
			Gm13151								Stxbp3a							Piwil2
			Atf2								Hmgn5							Rmnd1
			Adrb3								Fam187b							Whsc1l1
			Acsm2								Scube2							Pla2r1
			Gabpb2								Prim1							BC094916
			Tmcc3								Dock11							Micu1
			St14								Mta1							Cnot10
			Iqsec2								Aspa							Cirbp
			Tead3								Erlec1							Wdr41
			Arpc5l								Paqr5							Cask
			Rgag4								Ces1d							Cep72
			Ifnar1								Hao1							Sostdc1
			Armc2								Slamf1							Ero1l
			Snx33								Sptlc1							Cyb5rl
			Ero1lb								Ces2a							Tifab
			Tbl2								M5C1000I18Rik							Poc1a
			Rnpepl1								Arl8b							Ric8b
			Ralgps2								9430038I01Rik							Oasl1
			Kif16b								Zfp719							Gdap1
			Fam3c								Sapcd2							Ppp1r12c
			Glipr2								Pmf1							Prickle1
			Dctn4								Pigk							Fam124b
			Commd5								Angpt2							Ccdc108
			Trim30c								Pin1							Txlnb
			Fkbp2								Cd320							Dpp6
			Zfyve9								Rad51							Pgpep1
			Mark2								Rsl1							Bst1
			Rpl38								1700061G19Rik							1700029H14Rik
			Senp1								Rab29							Slc31a2
			9930111J21Rik1								Rac2							Spag16
			Fam195a								Srd5a3							Ano1
			Smarcad1								Pex16							Zfp319
			Zfp507								Apol10b							Zfp523
			Fam26e								Gm13889							Mmp16
			Olfr39								Pdxk							Agrn
			1600012H06Rik								Cnih3							Plekhh2
			Snx11								Mmd							Slc25a14
			U2af1								Abtb2							Arf1
			P2rx4								Acaca							Srsf11
			Lonrf3								Suco							L3mbtl4
			Mpv17								Gmeb1							Agpat3
			Zkscan1								Commd8							Pfpl
			Poldip3								Atp1a2							Caskin2
			Chrna1								Hist2h3c2							2310022A10Rik
			Tnks								Mknk1							B930041F14Rik
			Snhg11								Zmynd8							Adar
			0610031J06Rik								Ddx10							Trp53bp1
			Hist4h4								Cpt1b							Tnfaip3
			Zhx3								Alox5ap							Gstcd

miR-199-3p	miR-29-3p	miR-486-5p	miR-361-3p	miR-122-5p	miR-425-5p	miR-136-5p	miR-96-5p	miR-142-5p	miR-19-3p	miR-141-3p	miR-770-3p	miR-335-5p	miR-200-3p	miR-194-5p	miR-183-5p	miR-341-3p	miR-182-5p	miR-370-3p
			Zbtb7c								0610037L13Rik							Cog2
			Gmeb2								Nhp2l1							Plin5
			Znrf2								Mrpl3							Inhbe
			Slc10a7								Vars2							Lhfpl2
			Ttc33								Aldh1a7							Slc24a4
			Nbeal1								Dnase2b							Tlk1
			C77370								F13b							Ppm1h
			Btbd11								App							Ccdc116
			Smim19								Tubgcp5							Zfp213
			Shc3								Bax							Zc3h12b
			Slc25a35								Glod4							Dapp1
			P2ry2								Cyp2d40							Bcan
			Cbx5								Wnt11							Snx32
			Dnal4								Acat1							Ube2g2
			Gpr50								Gpr108							Gltpd2
			Myocd								Rhou							Rab3d
			Tspan2								Nab1							Lrp12
			Cd72								Pcna							Ywhaz
			Arhgef12								Cyp2j13							Fam120a
			Dpysl3								Crcp							Trp53inp2
			Glis3								Tbp							Timp3
			Trim11								Actn1							Zfp217
			Laptm5								Prep							Glrx3
			Ptpn6								Nfrkb							Gm13011
			Emc10								Csad							Fam160b1
			Grb2								Vash2							Ccdc127
			Arnt2								Cops7b							Trappc12
			2810006K23Rik								Rgma							Cdc42se1
			Coro7								Cst8							Dhx40
			Tmem178								Tmem258							Eif4e
			Ubfd1								C1d							Tert
			Ccrl2								Bet1							Ap1ar
			2310005G13Rik								Grtp1							Mon1a
			Fam71f2								2210016F16Rik							Eif2ak2
			Twf1								Ube2w							Fam163b
			Slc9a1								Serbp1							1300002K09Rik
			Afap1								Zmym1							Pkn2
			Gm10382								Uck2							Crlf3
			Rgs5								Dtd2							St7l
			Fbxo46								Trim72							Fam222a
			Appl1								Upb1							Cdc37l1
			Hs3st1								Rpp21							Ptprf
			Tnfrsf10b								Klhl5							2810004N23Rik
			Hist1h2bk								Rrp15							Gm9905
			Egfr								D830039M14Rik							Nabp1
			Dennd4b								Upk1b							Gpr75
			Hist1h4j								P2ry2							Ano10
			Fam78b								Eif1ax							Colgalt1
			Tnfaip3								Fkbp9							Slc35g1
			Tbc1d24								Cd36							Mfn1
			Repin1								Hrsp12							1110038F14Rik
			Apol6								Eid2b							Epb4.1
			Kpna3								Tax1bp1							Extl1
			Fbxo16								Med6							Nkg7
			Zbtb9								Otc							Rnh1
			Il18r1								Rnf146							Cst6
			Nsun4								1200014J11Rik							Sh2b2
			Kirrel3								Tmem261							Gpr6
			Slc32a1								Dnaaf2							Snx31
			Pde11a								Mdfic							Ajuba
			Zfp648								Lonp2							Arhgef16
			D630045J12Rik								Mpv17l2							Tdrp
			Tenm4								Spon2							Rtkn2
			Pex11b								Tmem98							Fundc1
			Mmp25								Limk2							Mapre3
			Nefl								Ifrd2							H2-Eb2
			1110002E22Rik								Tmem220							Optn
			Sftpa1								Mrpl13							Sec23ip
			Imp4								Zfp873							1700102P08Rik
			3830408C21Rik								Fgb							Suv420h2
			Kif1b								Gabrb1							Lefty2
			Tns3								Hadh							Rps6ka3
			Foxn2								AU022252							Btla
			Irf5								Nlgn1							Ubxn7
			Vezf1								Mettl21a							Tnxb
			Scn3b								Slc5a12							Gm7325
			Scfd2								1810037I17Rik							Ldha
			Nolc1								Elp4							Neu3

miR-199-3p	miR-29-3p	miR-486-5p	miR-361-3p	miR-122-5p	miR-425-5p	miR-136-5p	miR-96-5p	miR-142-5p	miR-19-3p	miR-141-3p	miR-770-3p	miR-335-5p	miR-200-3p	miR-194-5p	miR-183-5p	miR-341-3p	miR-182-5p	miR-370-3p
			Tnfrsf25								Mrpl57							Adnp2
			Ccbl2								Prpf38b							BC022687
			Tomm7								Rab3gap1							Ace
			Rdh10								Sepw1							Slc6a1
			Ntrk3								Gnl1							Dnmt3b
			Ttbk2								Gdf2							Chst13
			Igfn1								Kif18a							Cdc40
			Dhodh								Pgam5							Wwp2
			Hoxb2								Mrpl1							Trub2
			Lyn								Bbs12							Rap2c
			Wnt2								Poc1a							Map3k19
			Gng13								Zfp27							Gimap4
			Car5b								Emx2							Rdh8
			Mtmr14								Nagpa							Dennd2d
			Fga								Osr1							Pmp22
			Ago3								Cdkn2b							Oasl2
			Spata25								Fundc2							Smox
			Lin28b								Nme6							C330007P06Rik
			Maoa								Ccdc109b							Spsb4
			Serinc5								Rplp2							Arl10
			Sp2								Ttc32							Gprc5a
			Extl1								Tpk1							Tyw1
			1700012B09Rik								Agpat6							Rbm18
			AA986860								Ncl							Fads3
			Khdc1a								Klri1							Rims2
			Eapp								Fbxw5							Abca2
			Htr6								Cdk5rap3							1200014J11Rik
			Ubqln4								Atp5o							Dvl2
			Qk								Efcab9							Narfl
			Anxa5								Trim61							Tiam1
			Cenpm								Plet1							Dnajb12
			Adc								Arl3							Med28
			Dnaaf1								Fbxw2							Vdac2
			Ubb								Frk							Tmem202
			Abcb1a								4933434E20Rik							Baiap2l1
			Adtrp								Mapkapk5							Crtac1
			Pdgfra								Nr1h4							Prr5l
			Ccdc158								Idnk							Rhbdd1
			Tmem38a								Fbxw8							Grb2
			Tmem72								Npc2							Tnfaip2
			Greb1l								Nubp1							Mlst8
			Nhsl2								Phb							Slc39a3
			Scn4a								Erich1							Crkl
			Rcor1								Rnf130							Abcg2
			Ikbkb								Itgb3bp							Kansl1
			Phf21b								Sike1							Cyfip2
			Stoml1								Myo1b							Vac14
			Sprtn								Ier2							D930015E06Rik
			Lnx2								2310036O22Rik							1700021K19Rik
			Phldb1								Ppp4r1							Tbp
			Rnf24								Penk							Gpr12
			Clec3b								Ttc1							Kdm4c
			Prex2								Avil							Gcnt3
			Shc1								Pfkfb2							Cdv3
			Ablim3								Rps15a							Skida1
			Kctd2								Trp53rk							Capza1
			BC035044								Rpap3							Dnd1
			Zbtb8b								Aurkaip1							Dgcr6
			Rbms2								Commd1							U2af2
			Aff1								Csnk1d							Mllt1
			Mtfmt								Arrdc3							Nt5c2
			Mcf2l								Iars							Wdr20
			Nup155								Ppif							Lmnb2
			Ptprg								Ooep							9930021J03Rik
			Zbtb20								Pyurf							Aldh1a1
			Fam3a								Timm17b							Dkk3
			Dhh								Rab2b							Fam71f1
			Ces2g								Sec61g							Rem1
			Ildr2								Paip1							AA474408
			Sgip1								Ly6e							Ska2
			Prdm16								Lrrc3b							Klf7
			Atp8a1								Ier5							Ccar1
			Hbp1								Lect2							4930578G10Rik
			Hist1h2bh								Coq10b							1500009C09Rik
			Tmem220								Psmb1							Lrrc1
			Gm11444								Shcbp1							Fgf12
			Neurl1b								Kti12							Commd7
			2410131K14Rik								Cdh10							Slc1a1

miR-199-3p	miR-29-3p	miR-486-5p	miR-361-3p	miR-122-5p	miR-425-5p	miR-136-5p	miR-96-5p	miR-142-5p	miR-19-3p	miR-141-3p	miR-770-3p	miR-335-5p	miR-200-3p	miR-194-5p	miR-183-5p	miR-341-3p	miR-182-5p	miR-370-3p
			Dido1								Trmt2a							Cnnm2
			Churc1								Aass							Cabp2
			Zfp937								Bclaf1							Strbp
			Ten1								Prr15							Npcd
			Orai1								Nhp2							Zmym4
			Atp7b								Tnnc2							Fam171a2
			Fnbp1l								Tmem140							Tubb1
			Fundc1								Zscan21							Map7d1
			Emx1								Polr2l							Klc1
			Ncor2								Rps8							Csdc2
			Pla2r1								Rplp1							Eogt
			Vapa								Them7							Lzic
			Arhgap32								Gm2026							Hcn3
			Gltscr2								Ubl5							Mccc1
			Smim1								Tmem55b							Map3k5
			Fbxo21								Fam192a							Tbc1d10b
			Wnt11								Ankrd17							Mmachc
			Apc2								Rbfa							Slc26a9
			Dzip1l								Tspan3							Ppp1r3c
			Sfmbt1								Gm14399							Slamf6
			Gtf2e2								Cebpb							Arl8a
			Slc4a9								Bcat1							Map3k8
			Scube3								Fdft1							Dhh
			Gm17641								2610528J11Rik							Klf14
			Etnk2								Cfdp1							Lrrc48
			Fam198b								Snap47							Pcif1
			Tpte								Fuca1							Unc93b1
			Clasp1								Mrps31							Ptgfrn
			Pknox1								Pgap2							Ttc38
			Rapgef3								Gpr161							Tfcp2
			Sec63								Sec62							Hdhd2
			Ptgs1								Pet100							Rhou
			Ttll11								Sult1a1							Lrrc32
			Pik3ap1								Mrpl41							Cacna1b
			Cttnbp2nl								Tmem205							Fndc7
			Sh2d7								Phf10							Scaf11
			Ado								Chek1							Mars2
			Dedd2								Rap1a							Abt1
			Cox19								Myl7							Gm6526
			1700021K19Rik								Psmg4							Mrps2
			Pon2								Dnajc19							Ccng1
			Ccnt2								Deb1							Mpp2
			Dhrs9								Ggct							Zmynd11
			Tma16								Eva1b							Slc35f3
			Lrrc8d								Gpr12							Cramp1l
			Ost4								Ptms							Lphn1
			Dhcr24								Pfdn4							Treml2
			Cacng8								Sla							Lrrc58
			Bcl7a								Mrpl14							Dicer1
			Prr14l								0610009L18Rik							Ctdsp1
			Fkbp10								Tmem242							Kbtbd2
			Zfp189								Atg12							Tapt1
			Zfp658								Trappc2l							Krt7
			Rbm4								Cyp2a22							Phxr4
			Ankef1								Ppwd1							Sult4a1
			Tenm3								Mrps16							Lyve1
			Heyl								Fblim1							Mgam
			A330021E22Rik								Gm14440							Guca1b
			Ebp								Thumpd3							Crls1
			Tmtc1								Emc6							Zfp773
			Ece2								Nags							Anapc7
			Sgcb								Alg5							Frmd3
			Trib1								Ift81							Ppp1cc
			Zbtb7b								Cyp2j5							Cxadr
			Shroom1								Gtf2b							Vamp3
			Klf12								H2-Q10							Zfp354b
			Ciita								Kcnv1							Clmp
			Mrpl16								Dcn							Mlh3
			Dhx8								Ugt2b36							Zmynd8
			Asb2								Qprt							Aspa
			Actr1b								Yaf2							Gmppb
			Tmem126b								Galntl6							Fam86
			Trp73								Spr							Foxc1
			Qars								Rce1							Cmtm6
			Znf512b								Pik3ip1							Gja4
			Tmem173								Ssr3							Eif2b2
			Aqp7								Mrps18a							Vwa7
			Cacng1								Mrpl12							Prkg1

miR-199-3p	miR-29-3p	miR-486-5p	miR-361-3p	miR-122-5p	miR-425-5p	miR-136-5p	miR-96-5p	miR-142-5p	miR-19-3p	miR-141-3p	miR-770-3p	miR-335-5p	miR-200-3p	miR-194-5p	miR-183-5p	miR-341-3p	miR-182-5p	miR-370-3p
			Nwd1								Mrps9							Upf1
			Ppp1r7								Rpl7l1							1810049J17Rik
			Wdr18								Sergef							Neurl3
			Rasgrp4								Rpl26							Slc9a5
			Hint3								Tmem9							Carm1
			Vgll3								Ctsl							Psmd11
			Dab2								Atp1b2							Tada2b
			Rsf1								Hfe2							Fam192a
			Abhd6								Kdelr1							Zfp418
			Fbxl17								Sav1							Cks1b
			Map3k14								Ppip5k2							Prpf39
			Opcml								Timm8b							Dennd5a
			Adamts14								Glrx5							Efcab14
			Sgsm1								Cyb5r4							Rmi2
			Hcfc1								Tmem176a							Nudt19
			Ccsap								Pvalb							Fadd
			Mrps21								Abhd13							Papd7
			Runx3								Ckm							Kcnk12
			Parp14								Lyrm2							Zfp266
			Pot1b								Gyk							Smarcal1
			Brd3								Atp2a2							Fads6
			Cys1								Ifitm2							Acss1
			Rffl								Adat2							Tceb3
			Cep131								Letmd1							Akr1d1
			Ubxn8								Tmem74							Rfc5
			Strbp								Prr3							Thap3
			Btrc								Psme2b							Rnf20
			Dpy19l1								Slc1a1							Stx1a
			Pgm5								Abhd6							Mxra7
			Spata18								Lamp2							Mdfic
			1810032O08Rik								Fxn							Nebl
			Dhx37								Cdk1							Lrrc2
			Slc24a4								Fxr1							Rxrb
			Pi4k2a								Lair1							Wnt2
			Accs								Slc25a3							Pggt1b
			Tanc2								Cox7a2							Aqp4
			Nkx2-5								Fopnl							4931428F04Rik
			Prr19								Sod2							Smpd3
			Wdr52								Adipoq							Lnp
			Fip1l1								Pot1a							Slc43a1
			Ndrg4								Phyh							Acbd4
			Uxs1								Kbtbd3							Ints1
			Cplx3								Arhgap11a							Erich1
			4933426M11Rik								Sec11c							Aspscr1
			Lsg1															Ssr1
			Fndc3b															Rhob
			Mkln1															Pkib
			Kcnk6															Slc26a11
			Zfp780b															Pate2
			Hoxa3															Zfp202
			1700021F05Rik															Derl1
			Serpina11															Tmem8b
			Nvl															Gcsam
			Fam83g															Odf3l1
			Magi1															Actr5
			Elavl1															Zfp386
			Cd8a															Zfp709
			Tarm1															Stom
			Mmd															Neil2
			Sult4a1															Tctn3
			Mdm4															Cep41
			Kcnc1															Mre11a
			Def8															Rhot1
			Cacnb4															1600012H06Rik
			Gpx4															B430305J03Rik
			Cdk7															Tbc1d9b
			9630033F20Rik															Gna12
			Gucy2g															Eif3c
			Eif4g1															N4bp3
			1700030J22Rik															Unc119b
			Klf15															Ski
			Krba1															Frat2
			Rpl21															Fbxl6
			Cyld															Zdhhc24
			Cyp2j13															Dusp15
			Sgms2															Srek1
			Atl3															2310067B10Rik
			Prkca															Samd8

miR-199-3p	miR-29-3p	miR-486-5p	miR-361-3p	miR-122-5p	miR-425-5p	miR-136-5p	miR-96-5p	miR-142-5p	miR-19-3p	miR-141-3p	miR-770-3p	miR-335-5p	miR-200-3p	miR-194-5p	miR-183-5p	miR-341-3p	miR-182-5p	miR-370-3p
			Nub1															Evi2a
			Foxp4															Crp
			Cpeb3															Myo1b
			Numbl															Prtn3
			Chordc1															Tmie
			Fem1a															Gga3
			Galc															Zfp28
			Rrp7a															Mapre1
			Mmp15															Ptprd
			Gm3448															Btnl2
			Cdh6															Cep135
			Cmc2															Spata18
			Memo1															Ap3s2
			Smarce1															Dedd2
			Gstm4															Ermp1
			Fam20a															Large
			Diablo															2610015P09Rik
			CTNND1															Ush1c
			BC049352															Rfng
			Cd3d															Uggt2
			Plb1															Plekhg2
			Cst8															Hgs
			Crispld1															Nucb1
			Fam189a1															Trpm3
			Zfp385c															Ddx28
			Uggt2															Esp31
			Zfp563															Klhl8
			Csrp2															Htr1b
			Slc25a32															Lrrn2
			Cdr2l															Hoxa11
			Acvr1															Gm10645
			Zfp329															Fam174b
			Pigp															Car10
			Kbtbd2															Tmem176a
			Itk															Cyp27b1
			Klhl26															Parpbp
			Sh3pxd2b															Acr
			Pcdhb16															Gosr2
			Fam78a															Slc41a2
			2310022A10Rik															Tceanc2
			Tmem201															Rngtt
			BC035947															Rnf125
			Muc6															Rab3gap2
			Adcy3															Tsku
			Git1															Cltc
			Skp2															2610002J02Rik
			Tsc22d3															5830454E08Rik
			Dpp9															Hsd11b2
			Kcnq2															Mrpl36
			Sox12															Sgms1
			Trav7n-6															Igfbpl1
			Plekhm2															Rnase9
			Atp1a3															Nlrp4e
			Mcc															Tmem18
			Flt3															Fbxo28
			Dip2a															Tmx4
			Scrt1															Jam3
			Gm5595															Kcp
			Prkar1b															Exd2
			Baiap3															Sumo3
			M1ap															Kidins220
			Wfdc8															Dlst
			Slc7a14															Gzf1
			Smad6															Hmgn1
			Itgb3															Ifng
			Fa2h															Chordc1
			Kcnb2															Mfsd2b
			Epha2															Spns3
			Kri1															Avpr1a
			Setd5															1700026D08Rik
			4933427D06Rik															Slitrk5
			Gm8113															Smim13
			Mdn1															Scarf2
			Ctnnd1															Tnks
			Slc35e1															AI661453
			Sept9															Mtnr1a
			Eid2b															Cycs
			D430042O09Rik															Atg7

miR-199-3p	miR-29-3p	miR-486-5p	miR-361-3p	miR-122-5p	miR-425-5p	miR-136-5p	miR-96-5p	miR-142-5p	miR-19-3p	miR-141-3p	miR-770-3p	miR-335-5p	miR-200-3p	miR-194-5p	miR-183-5p	miR-341-3p	miR-182-5p	miR-370-3p
			Lamp1															Stt3b
			Pcbp4															Epha5
			Trp53inp2															Ctu2
			Atp6v0a1															Lag3
			Mlst8															Timm44
			1700019N19Rik															Chil1
			Ddi2															Ghdc
			Enpp1															Rnase6
			Gna12															Gm6970
			Notch1															Selt
			Mkl2															Msi1
			Ggt5															Arl4c
			Sec14l5															Siah1a
			Ercc2															Gja1
			Slco2a1															Mef2d
			Coro2b															Ptchd4
			Sumo2															Dennd6b
			Alkbh5															Zbtb49
			Ldhd															Atp8b2
			Milr1															Glt6d1
			Ptpdc1															Arl11
			Map3k5															Cks1brt
			Hnf1a															Smco4
			Plekhd1															4930538K18Rik
			Prkg1															Nkx6-2
			Nkx2-1															4930481A15Rik
			Gpc4															Aplf
			Cyyr1															St3gal1
			Scn5a															Mcam
			Gas7															Asic4
			Lipo1															Pde6h
			Btaf1															Arfgef2
			Cdkal1															Robo4
			Lynx1															Ift20
			Pes1															Layn
			Mypn															Tmed3
			Ufm1															Ppm1d
			Hmgxb3															Cd300a
			Ecm2															Nlrp4c
			Taco1															Dll4
			Bola3															Crebl2
			Cyb561d1															Impact
			Nol4l															Asb16
			Alpk1															Tmem143
			Exph5															Lrp8
			Susd1															Pcdh17
			Gm9776															Mvk
			Frk															Stmn4
			Iyd															Nipsnap1
			St3gal1															Smek1
			Xpo4															Sar1a
			Lime1															I0C0044D17Rik
			Zfp704															Jam2
			Dll4															Tgm6
			Mprip															Rcbtb1
			Tpgs2															Fam19a5
			Bach2															Aldh1a3
			Uhrf1bp1															Ptges
			Lpar2															Hnrnpul2
			Csnk2a1															Zfp668
			Osbpl3															B4galnt4
			Gsk3b															Mycl
			Sirpa															Csnk1a1
			Zdhhc17															Fam206a
			Ube2k															Dcun1d1
			Mfn1															Rab9
			Uggt1															Top1mt
			3110021N24Rik															Ift52
			Stxbp5l															Tpcn2
			Tmem212															Dvl3
			Ccdc115															Insig1
			Retn															Apoa5
			Avpr1a															Ccdc47
			Kdm5b															Tmem161b
			Asns															Spata22
			Lnpep															Mitd1
			Prdm5															Rab3il1
			Skil															Irf8

miR-199-3p	miR-29-3p	miR-486-5p	miR-361-3p	miR-122-5p	miR-425-5p	miR-136-5p	miR-96-5p	miR-142-5p	miR-19-3p	miR-141-3p	miR-770-3p	miR-335-5p	miR-200-3p	miR-194-5p	miR-183-5p	miR-341-3p	miR-182-5p	miR-370-3p
			Zcchc6															Nsmf
			Rmnd5a															Ampd3
			Gramd2															Yae1d1
			Brsk2															Sec22a
			Sel1l															Csnk1g3
			Hspa4l															Rnf123
			Traf3ip1															Dhrs3
			Agpat6															Dgkg
			Lmod1															Cndp1
			Efcab2															Cyp21a1
			Chmp4b															Pnoc
			Zfp940															Gm5900
			Plxdc1															Neto2
			Mapkapk2															Taf12
			Slc26a3															Prox2
			Tbc1d25															Nlgn2
			Trmt10c															Fastkd1
			Tomm40l															Anks1b
			Slc52a2															Armc6
			Zeb2															Zfp563
			Tpd52															Lrit1
			Lrrc9															Plcb2
			Slc17a3															Purb
			Agtrap															Acsm2
			Ccdc19															Steap3
			Gm5084															Wdr45b
			Tns4															Ncapd2
			Cdh15															Mrap
			Sh2d2a															Rassf7
			Sh3kbp1															Ercc8
			Slc25a38															Kcnj13
			D1Ertd622e															Il17rb
			Ido2															Zfp655
			Ankrd9															Ywhae
			Ctnna3															Igdcc3
			Lmo4															Crlf2
			Gpr89															Pstpip2
			Vrk1															Smtnl2
			Heatr2															Evx1
			Mgmt															Scara3
			Aebp2															Exoc7
			Setd7															Cops3
			Il34															Csrp1
			Pigc															Olfml1
			Pitpnm2															Emx2
			Cacna1d															6430573F11Rik
			Cant1															Mea1
			Slc30a7															Actr1b
			Slc30a10															Hnrnpr
			Ly96															Gm10010
			Zfp292															Fbxo46
			Slc38a3															Prkd3
			Rnf220															Clasp1
			Ptk2															Mipep
			Pik3r6															Ipo5
			Casc3															Zbtb24
			Mau2															Cep57
			Usp31															Gm826
			Garem															Snapc2
			Aif1															Mfsd1
			Bod1															Terf2
			Srgap3															Itgad
			Ism2															Sike1
			Pcsk2															Kcnk2
			Zkscan2															Dnajc11
			Mpg															Esco1
			Zc2hc1c															Tcirg1
			Foxf2															1700067K01Rik
			Jrk															Slc11a1
			Thumpd2															Hpdl
			Myl6															Fastkd3
			Csmd2															Ddx24
			Nasp															Sirt7
			Xirp2															Slc51a
			Pom121															Suds3
			Gpam															Cnnm1
			Rnf144b															Chst4
			9130019O22Rik															Timd4

miR-199-3p	miR-29-3p	miR-486-5p	miR-361-3p	miR-122-5p	miR-425-5p	miR-136-5p	miR-96-5p	miR-142-5p	miR-19-3p	miR-141-3p	miR-770-3p	miR-335-5p	miR-200-3p	miR-194-5p	miR-183-5p	miR-341-3p	miR-182-5p	miR-370-3p
			Lpar5															Mat2a
			Trub2															Trim35
			Luc7l2															Ica1l
			Rps4l															Hrct1
			Zeb1															Zfp329
			Cecr2															Ssx2ip
			Spocd1															Ube2d2a
			Samd8															Slc35b2
			Cpxm2															Filip1l
			Hapln4															Ikbkap
			Spred1															Rasgrp3
			Ccin															Nab1
			Hs2st1															Wfdc18
			Ppp1cc															G430049J08Rik
			Spast															Coq10a
			Ndufa9															Atf5
			Abhd15															Mzf1
			Galk1															Lingo2
			Epb4.1															Itm2c
			Otud6b															Rrp12
			Slc6a1															Foxa1
			Fscn1															Lmx1a
			Cers6															1700030J22Rik
			Zfp11															Tpte
			Pdlim1															Slc35f6
			Bace2															Gsr
			1700001O22Rik															Cyp26b1
			Prdm2															Mapk8
			Ankrd39															Uap1
			Col22a1															E2f2
			Gnb1l															Uba6
			Ankrd28															Eef2k
			Rps12															A430033K04Rik
			Hpse															Thnsl1
			Plekhj1															Fhod3
			Mllt1															Mlph
			Cpt1c															Ppme1
			1700067K01Rik															4921517D22Rik
			Ube2z															Tmem109
			Gnrh1															Fam89a
			Diexf															Mlip
			Tmem86b															Rundc3b
			2510009E07Rik															Zfp760
			Zmym5															Smarca5
			Rnls															Cdh13
			Nup62															Cygb
			Slc43a2															Arhgap27
			Sphk2															Irf6
			Ucp2															Zfp955b
			Acr															Tmem110
			Fam86															2410127L17Rik
			Gch1															Ston1
			Ppme1															Uhrf1bp1l
			Scimp															Pex19
			Foxj3															Agt
			Fcho1															Elfn2
			Tril															Fgf1
			Ralgapb															Psma8
			Cln8															Polr2m
			Hal															Phxr2
			Ccdc127															Ctsd
			Reep3															Sord
			Pdhx															Gm15698
			Mterf1b															Rqcd1
			Hmgcr															Abra
			D430041D05Rik															Pik3ca
			Hnrnpab															Mxd1
			Asb10															Gdf6
			Glyat															Mmaa
			Ttc14															Gemin4
			Klhl38															Fcrl1
			Map3k9															Rnf40
			Ddx10															Hk2
			Nsd1															Wash
			Prss23															Ska1
			BC067074															Vstm2b
			Amotl1															Slx1b
			Mon2															Cyb5r4

miR-199-3p	miR-29-3p	miR-486-5p	miR-361-3p	miR-122-5p	miR-425-5p	miR-136-5p	miR-96-5p	miR-142-5p	miR-19-3p	miR-141-3p	miR-770-3p	miR-335-5p	miR-200-3p	miR-194-5p	miR-183-5p	miR-341-3p	miR-182-5p	miR-370-3p
			Zfp316															Tmem11
			Itgb1bp1															Tox2
			Sptb															Mapk4
			Slc11a1															Glt1d1
			Hrct1															Alad
			Cenpb															Mob3c
			Plxna4															Naif1
			Mboat2															Panx1
			Aptx															Aldh1l2
			Tm2d1															Dstyk
			Nat10															Zbtb9
			Il20rb															Ndor1
			Prr5l															Arpc4
			Nos1															Ociad1
			Slc39a5															Ank1
			Nxf1															Hadha
			Emc3															Oard1
			Ces2a															Ppm1m
			Arl3															Gfm2
			Ubald1															Vta1
			Gpr83															Mief1
			Ddx25															Ankef1
			Rab23															Otub2
			Dact3															Leprot
			2810459M11Rik															Mbd3l2
			Mcm2															Cyb561d1
			Homer1															Fbxl2
			Kcng4															Bbs12
			Depdc5															Csnk2a1
			Plekha6															Trub1
			Il6st															Ccl21a
			Lyl1															Gm10591
			Usp3															Gm21541
			Med15															Ccl21b
			Slc43a1															Gm13304
			Heatr6															Adm2
			Plin4															Prdx6
			Galnt6															F10
			Epb4.1l5															Gabrg2
			Fzd2															Cabyr
			Dnajb14															1810009A15Rik
			Ctbp2															Dkk2
			Myct1															Mul1
			Nsfl1c															Smim1
			Cdk6															Gm21743
			Inha															Syt3
			Slco3a1															B4galt7
			Vav1															Ubr1
			Sgk2															Catsper2
			Gm15557															Rims4
			Maf															Npr3
			Cysltr2															Tet1
			Rrp8															Cntnap2
			Commd6															Ndufa4
			Lrrc10															Ftsj2
			Tspan18															Dnpep
			Fam65b															Ube2n
			Ing2															Twf1
			Zfp84															Cant1
			Pmf1															Yy1
			Flywch2															Morc4
			Zfp2															Lrrc75a
			Six1															Galnt16
			Slc25a21															Chd1
			Gm14419															Begain
			Tbc1d14															Rpl9
			Ccnjl															Ttc29
			Fxyd5															Ppp2r4
			Fkbp5															Orai3
			Auts2															1700013F07Rik
			Trpc3															Prss33
			Per1															C130026I21Rik
			Fktn															Dennd6a
			Nod2															Hal
			Trim66															Ufm1
			Kif5a															Rhbdd2
			Rph3a															Xkr9
			Foxa1															Map4k5

miR-199-3p	miR-29-3p	miR-486-5p	miR-361-3p	miR-122-5p	miR-425-5p	miR-136-5p	miR-96-5p	miR-142-5p	miR-19-3p	miR-141-3p	miR-770-3p	miR-335-5p	miR-200-3p	miR-194-5p	miR-183-5p	miR-341-3p	miR-182-5p	miR-370-3p
			Nwd2															Umps
			Kif13b															Ppig
			Hspg2															4930562C15Rik
			Fbxo41															Six6
			Slc5a3															Ept1
			Pde2a															Sh3kbp1
			Serpinb5															Dclre1b
			Ddhd1															Sbk2
			Rrp12															9430007A20Rik
			Asgr1															Fkbp8
			Znrd1as															Ahcy
			Ppp1r14a															Cdk16
			Bdh1															Tc2n
			Polr3a															Gpc4
			Pdcl3															Gfer
			Bdnf															Ankib1
			Msi2															Slc52a2
			Chid1															Ptpn11
			Zfp85															Enc1
			Ppp1r16a															Vti1a
			Ubxn7															Abca3
			Ddx4															Zfyve9
			Ano5															Fam217b
			Tinag															Cdca5
			Hddc3															1810013L24Rik
			Dnajc12															Il7
			Srgn															Gimap3
			Trip4															D17Wsu104e
			Prkaa2															Dnajc5
			Pgm2l1															Clrn1
			2010107G23Rik															Rims3
			Tmc6															Depdc1b
			Zfp827															Osbpl3
			Rpp14															Arhgap1
			P4htm															Erich2
			Dok2															Lama4
			Pik3cb															Rnpepl1
			Cacna1c															Ssfa2
			Pin1															Afm
			Atp2b1															Rrm1
			Rem1															App
			Prrc2a															Trpc5
			Dnaja4															Itln1
			Cacna1a															Pcbd1
			Mpv17l															Gm6133
			Nrip1															Mcoln3
			Cnot6															Klf6
			Lrrn4															Pcdhb4
			Atg2b															Tmem221
			Acot12															Zfp235
			Lifr															Ube2f
			Acbd3															A930018M24Rik
			Sbk3															Plekho2
			Zbtb44															Ofd1
			Ms4a8a															Gstm7
			Zfp334															Rnf43
			Sh3pxd2a															Fdft1
			Gca															Fam46c
			Necab3															Jmjd4
			Impa2															Ubtf
			Rora															Map3k12
			Isy1															Maz
			Grik4															Gtpbp6
			Umps															Nupr1
			Vwa5a															Fut7
			Naa15															Serf1
			Ccdc86															Herpud1
			Coq10a															Mrgprb1
			Stk3															BC018242
			Arhgap8															Gm5930
			Aqp2															Mknk1
			Med12l															Gm3646
			Grk6															Slc7a5
			Cstf1															Paxbp1
			Hnrnpr															Vapb
			Ppp1r3f															Gria4
			Rftn1															Suclg2
			Zfp866															Tfe3

miR-199-3p	miR-29-3p	miR-486-5p	miR-361-3p	miR-122-5p	miR-425-5p	miR-136-5p	miR-96-5p	miR-142-5p	miR-19-3p	miR-141-3p	miR-770-3p	miR-335-5p	miR-200-3p	miR-194-5p	miR-183-5p	miR-341-3p	miR-182-5p	miR-370-3p
			Dlgap4															Luzp2
			Tceb1															Tmem168
			Fkbp4															Ttc33
			Mfng															Stab2
			Wee1															Zfp326
			Nucks1															Epn2
			Vstm4															B4galt4
			Upf3b															Il13ra1
			Bbox1															Tlk2
			Dock5															Gm454
			Tln1															Traf4
			Sowahb															Rit1
			Vwce															Mettl10
			Popdc3															Kcnc2
			Dnajb6															Col8a2
			Adamts3															Galk1
			2810004N23Rik															Ccr6
			Mtdh															Tcea1
			Sox1															Clec4e
			Kcnh5															Stk4
			Mul1															Bclaf1
			Gprin3															Klhl38
			Asxl2															Elf4
			Marco															Rtf1
			Nbn															Fem1c
			Hectd3															Rpa1
			Mettl16															Sap30bp
			Vkorc1															Cdk6
			Exd1															Hoxb8
			Esyt1															Magi2
			Dnajc13															Papss2
			Rabepk															Gap43
			Plekhg2															Krtap5-3
			Anks1b															Tuft1
			Gnptg															Cacng1
			Uap1l1															BC025446
			BC004004															Fam60a
			Urgcp															Cggbp1
			Ackr2															Sp100
			Mapk13															Des
			Slc52a3															Hoxd4
			Klrg2															Abcd2
			Usp15															Lad1
			Gtf2a2															Ehd1
			Kcnn3															Pank2
			Gspt2															Slc39a13
			Ogfrl1															Smad4
			St3gal6															Dhrs9
			Mmaa															Esrp2
			Fbxl6															Cnksr3
			Pank1															Tmem161a
			Zfp141															Tmem41a
			Inip															Kank3
			Actn1															Fam3a
			Ntn3															Gramd3
			Atp6v1b1															Slc16a10
			Krtap6-5															Fads2
			Ufl1															Stx2
			Hinfp															Mmab
			Gatad2a															Abcg1
			Pdxk															Relt
			Pask															Etl4
			Cnpy1															Heatr1
			Prr15															Aptx
			Rbck1															Adamts5
			Timp4															Strn3
			Zfp870															Ptgis
			Adcy4															R3hcc1
			Serpina3c															Rft1
			Anapc4															Tmem70
			Mmp11															Ndn
			Chchd4															Zfp707
			Lipt1															Prcc
			Eif5b															Mdh2
			Fbxo3															Rbm34
			Itga4															Ing1
			Lrpap1															Rela
			Tsku															Wscd2

miR-199-3p	miR-29-3p	miR-486-5p	miR-361-3p	miR-122-5p	miR-425-5p	miR-136-5p	miR-96-5p	miR-142-5p	miR-19-3p	miR-141-3p	miR-770-3p	miR-335-5p	miR-200-3p	miR-194-5p	miR-183-5p	miR-341-3p	miR-182-5p	miR-370-3p
			Rplp2															Ticam2
			Bid															Acaa1a
			Ubap2															Qsox1
			Ifrd2															Nt5c3
			Fsd2															Tssk1
			Dhrs7b															AA386476
			Fkbp9															Ascc1
			Grk5															Zfp446
			Slc9a4															Klhl20
			Nnmt															Elf1
			Gm7008															Il7r
			Ccdc13															Scube2
			Ugt3a2															Sis
			Noc3l															Cd5l
			Nxt1															Adprhl2
			Exoc7															Phf20
			Rbm28															Zbtb45
			C87436															Taf5l
			Traf5															Tmem173
			Frmd7															Adamts7
			Nfe2															Prr12
			Atf3															Rbm19
			Narfl															Dcaf8
			Lztfl1															Crip1
			Cyp2j9															AI848285
			Xrcc2															Mecr
			Eif4e															Hhex
			Tspan32															Rab8a
			Tmf1															F8
			Zfp668															Zic1
			Arhgef26															Tom1
			Nrcam															Tpd52l2
			3632451O06Rik															Zfand2a
			Zfp3															Mdm4
			Clec4d															Erbb2
			Pet100															Msantd2
			Mpi															Wars2
			Gng5															Plb1
			AU021092															Arfgap2
			Hddc2															Spp1
			Tuba4a															Hoxc13
			Pfn4															Slc48a1
			Slc17a4															Oas1b
			Cd8b1															Tmem45b
			Col4a4															Anapc2
			Arrdc1															Lgals3bp
			Arid5a															Kcnab1
			Alad															Sqstm1
			Plcb3															Olfr519
			Ccdc141															Tmem169
			Hsd3b2															Frmd6
			9530003J23Rik															Slc16a6
			Zfp239															Slc26a3
			Pink1															Slc38a3
			Cnot11															Fut9
			Cutc															Xpo7
			Ddx19a															0610009O20Rik
			Emc4															Trmt5
			Nrn1															Scgb1b17
			Mtr															Scgb1b15
			Rimklb															Rbpjl
			Cnst															Klhl17
			Chsy3															Npy4r
			H13															Sppl3
			Dpp6															6030445D17Rik
			Pikfyve															Atg12
			Ints12															Rtn4rl1
			2310057M21Rik															Rab11b
			Trpm6															Trpc2
			Slc17a2															Olfr920
			Efcab9															Ndufab1
			E130308A19Rik															Suco
			Ghitm															Gm21885
			Stam2															Timp2
			Dennd2a															Smc2
			Lst1															Ercc3
			Clasrp															Cenpm
			Hmmr															Epn3

miR-199-3p	miR-29-3p	miR-486-5p	miR-361-3p	miR-122-5p	miR-425-5p	miR-136-5p	miR-96-5p	miR-142-5p	miR-19-3p	miR-141-3p	miR-770-3p	miR-335-5p	miR-200-3p	miR-194-5p	miR-183-5p	miR-341-3p	miR-182-5p	miR-370-3p
			Aip															Ephb4
			Crat															Wdr38
			Phf21a															Dck
			Rcc2															F9
			N28178															Dnaja3
			Smyd3															Tex9
			Lrrc28															Bace2
			Prss41															Hid1
			Arhgap1															Ism1
			Spice1															Slc8b1
			Akirin1															Slc13a3
			Tubgcp2															Tmc2
			Six5															Cyp4f39
			Gm12184															Ccrl2
			Igf2bp2															Trim11
			Arcn1															Akap4
			A530053G22Rik															Coa7
			Paqr5															Stau1
			Rufy2															Lepre1
			Abcc6															Sec61a2
			Anks6															Npepl1
			Armc1															Cd27
			Dlg5															Acmsd
			Pacs1															Fxyd6
			Cnot10															Enox1
			Tnfaip8l1															Pde8b
			Zfp639															Phf19
			Znrf3															Erp29
			Efcab11															Cdca7l
			Rnaseh2c															Srd5a3
			Fyn															2210016L21Rik
			Pla2g16															C9
			Gm13889															Arfip1
			Wbp4															Srrm1
			Pnkp															9330182O14Rik
			Trim6															Triqk
			Sdf4															Ensa
			Colec12															Slc25a36
			Dock6															Mstn
			Timm17a															Atg13
			Zfp318															Nat10
			Gcdh															Zfp219
			Fam111a															Nfe2l1
			Ntf5															Nelfb
			Gpat2															Wdr1
			Wdr45b															Camk2b
			Rps17															Mrrf
			Sike1															Gcgr
			Kcnq1															E030019B06Rik
			Layn															Nfx1
			Trip6															Vwce
			Aldh1l2															Hsf2
			Uqcr10															Ugt2b35
			Abcd2															Rbm15
			Dsel															Supv3l1
			Gcgr															Mtmr6
			Pld5															Pfkm
			Rad18															Klra2
			Hdlbp															Myadm
			Ak5															Htr7
			Pigk															Grk5
			Cacna2d4															Wdr4
			Hsf2bp															Desi1
			Zfp593															Rho
			Pcdhb22															Zdhhc1
			Serpina3m															Cdkl1
			1810011H11Rik															Nme6
			Lmf1															Acsm1
			Metrn															Kcnk10
			Arl4a															Hps5
			Prr16															Rspo3
			Agpat5															Slco2b1
			Olfml3															Zfp64
			Rrnad1															Dgki
			Rpl7l1															Ckap5
			Ccdc62															Ly75
			Stoml2															Spop
			Taf3															Sccpdh

miR-199-3p	miR-29-3p	miR-486-5p	miR-361-3p	miR-122-5p	miR-425-5p	miR-136-5p	miR-96-5p	miR-142-5p	miR-19-3p	miR-141-3p	miR-770-3p	miR-335-5p	miR-200-3p	miR-194-5p	miR-183-5p	miR-341-3p	miR-182-5p	miR-370-3p
			Dpp4															Loxl1
			Rprd1b															Ccdc85b
			Apobec3															C3ar1
			Nme2															Trpm4
			Pmm2															Otud1
			Ephb2															Zcchc4
			Sh3rf3															Taf1c
			Fign															Mog
			Ttc12															Slc44a1
			Aox3															Eno1
			9430016H08Rik															Vwa5a
			Glod5															Srek1ip1
			Ugdh															Dock1
			Khnyn															Il1f9
			Scg3															Lrrc52
			Tmem60															Mas1
			2610524H06Rik															Rmdn2
			Tmem258															E130309D02Rik
			AA415398															Epb4.1l5
			Zfp324															Hmg20b
			Surf2															Btrc
			Tgm1															Sdhaf2
			Sim2															Stk16
			Tmem174															Sctr
			Mospd2															Lrrc51
			Mnat1															Gm16401
			Arfgef2															AI317395
			Cyp27a1															Mrpl48
			Rpap3															Gins1
			Agxt2															Foxp3
			Tshz2															Suv39h2
			Tbp															P2rx5
			Kcnj13															Pyroxd2
			Yaf2															Dlgap2
			Gm20390															E030018B13Rik
			Dctn1															Tomm34
			Wnt4															Fam104a
			Cdk2ap1															Nxn
			Lrrc4c															Tspan3
			Zfp282															TRPC2
			Cblc															St3gal4
			Zrsr1															Pm20d1
			Samm50															Duox1
			Usp24															Kcnc4
			Kansl1															Prkch
			Slc24a1															Arsb
			Tm4sf1															Itch
			Ush1c															Eif2d
			Ktn1															Siglece
			Lrrc25															Dpep3
			Calb1															Fam83b
			Oscp1															1110025L11Rik
			Blzf1															Apex2
			Idnk															Rnf217
			Trim26															Col3a1
			Hcar1															Pex16
			Pvalb															Vasp
			Dlg2															Gm3086
			Rac2															Rdh19
			Cyth2															Fam178b
			Ptprr															Cxcl16
			Prpf38a															Hist1h2bm
			Ugt8a															Ppfia3
			Mef2d															Ehd3
			Wdr20															Elovl1
			Abhd11															Pdhb
			Akr1e1															Cars
			Star															Pcgf5
			Bpgm															Acat1
			Atp6v1d															Wipf3
			Qpctl															Popdc3
			Tomm6															Lmod3
			Nhej1															Lgr5
			S1pr2															Ptpra
			Rnf40															Synj2bp
			Ube4a															Dtnb
			Zc3h15															Arhgap8
			Agr3															Mapk9

miR-199-3p	miR-29-3p	miR-486-5p	miR-361-3p	miR-122-5p	miR-425-5p	miR-136-5p	miR-96-5p	miR-142-5p	miR-19-3p	miR-141-3p	miR-770-3p	miR-335-5p	miR-200-3p	miR-194-5p	miR-183-5p	miR-341-3p	miR-182-5p	miR-370-3p
			Edc3															Tfr2
			Rock1															Mfsd8
			Zfp719															Diablo
			Kidins220															Upb1
			Lyrm5															Esm1
			Ddx54															Adam15
			Oxnad1															Mcm5
			Gtf2h3															Gm765
			Fam65a															Shisa4
			Slc8a1															Tor3a
			Zfp27															A1cf
			Katnb1															Tgfb2
			Colgalt1															Pi4k2a
			Slc2a8															Irf5
			Ank1															Asic1
			Zfp612															Zfp113
			Hs3st6															Rbfox3
			Rps19															Exog
			Lsm3															Med16
			Esyt2															Cryz
			Trp53rk															Dcaf12
			Ldhb															Usp47
			Agl															Itga1
			Ndufa10															March2
			Cdk8															Cpeb3
			Ier2															Sdc1
			Ifngr2															Rd3
			Sdhaf2															Gm933
			Cd300lg															Hoxb6
			Syn2															Zfp263
			Them4															Tmem25
			Cnksr3															Fbxo8
			Pdlim2															Cyb5d2
			Ocel1															Ascl1
			Psmf1															Sapcd2
			Upp1															Mfsd7b
			Polh															Mon2
			Nr1h4															Runx3
			Cebpb															Rbfa
			Rrp15															Aars
			Efhb															St13
			Ttbk1															Ces1g
			Trp53bp1															Egflam
			Kng2															Arf6
			Cluap1															Zfp81
			Nsdhl															Pdk3
			Extl2															Abcg3
			Pcid2															Rpap1
			Abcc1															Kiz
			Nploc4															Cdpf1
			Zfp623															Gabarapl1
			Caskin2															Serpini1
			Mthfsl															Rgs19
			Aste1															Cspg5
			Polr3k															Emp1
			Crybg3															Antxr2
			Snx12															Serp1
			Me2															5730455P16Rik
			Ctc1															Arl5a
			Lctl															Nkx2-5
			Fbrs															Cdc14b
			Tbck															Apol10a
			Ces1f															Gria2
			Lmtk3															Pafah1b2
			Zcchc8															Ctps2
			Adat1															Susd1
			Gm21992															Pla1a
			Rcl1															Gtf2h3
			Calcoco1															Zfp27
			Loxl4															Gpr149
			Mccc1															Casc5
			BC021785															Snx16
			Atp9a															Xpo1
			Ctns															Top2b
			Ank3															Krt24
			Slc9a3r1															Ccny
			Ift22															9330182L06Rik
			Eif4ebp1															Rprml

miR-199-3p	miR-29-3p	miR-486-5p	miR-361-3p	miR-122-5p	miR-425-5p	miR-136-5p	miR-96-5p	miR-142-5p	miR-19-3p	miR-141-3p	miR-770-3p	miR-335-5p	miR-200-3p	miR-194-5p	miR-183-5p	miR-341-3p	miR-182-5p	miR-370-3p
			Shisa4															Kitl
			Lrp10															Gfra3
			Vps4b															Tlr4
			Ugt2b37															Ldhb
			Hoxc10															Pgap2
			Cables1															Lrrc8d
			Tmed9															Lingo1
			Actr8															Cdh1
			Mrs2															Nat6
			Aass															Ccdc69
			Cyp4f15															1110032F04Rik
			Eif4g2															4933415A04Rik
			Ubiad1															Pgam5
			Tmem126a															Ces3a
			Gmcl1															Fsbp
			Lurap1l															Snx11
			Lgals3bp															Rab23
			Atp5b															2310057M21Rik
			Tcea2															Six5
			Abi3															Trhde
			Anln															Slc36a2
			Rtn4ip1															Plvap
			Anapc10															Tmsb4x
			Pklr															Sigirr
			Adam22															Riok3
			2210016L21Rik															Lyg2
			Fam35a															Rad54b
			Thap4															Dedd
			Ptgis															Asgr2
			Ift88															Cd40lg
			Mmp16															2610002M06Rik
			Orc3															Abi3
			Smc3															Hddc2
			Pparg															Alyref
			Zfp142															Plin2
			Myo5a															Ptgr1
			Syne4															Gorasp1
			Sart3															Lmnb1
			Rgmb															Dph6
			Nod1															Usp38
			Rnmtl1															Rilpl2
			Hscb															Epor
			Rbm33															Gm128
			Tpp2															Tradd
			Slc25a25															Dao
			2610528J11Rik															Pax5
			Cbx4															Pianp
			Dnajc15															Adap1
			Tmem53															Hnrnpab
			Dtnb															Reep4
			Dram2															Gm5884
			Exo1															Ctsk
			Nphs2															Kmo
			Cisd1															F930015N05Rik
			Rps15a															Echdc3
			Hadhb															Wnt3a
			Gfm1															Zyx
			4921524J17Rik															Mtfr2
			Dusp13															Rsrc2
			B4galt7															Csnk1d
			Nol12															AW822073
			Tnfsf13b															Apobec2
			Eno4															Svip
			Acsm1															Pax3
			Chst1															St3gal3
			Trat1															Fzr1
			Cyp4f16															Gabarapl2
			Adamts7															BC024139
			Ces1d															Cbx6
			BC022687															Dock2
			Pgam5															Msr1
			Metrnl															Ifngr2
			Ldlrap1															Colec12
			Hif1a															Snx12
			Man2c1															Prelp
			Rab11b															Mctp2
			Ap5b1															Mff
			Ctnnal1															Abcd1

miR-199-3p	miR-29-3p	miR-486-5p	miR-361-3p	miR-122-5p	miR-425-5p	miR-136-5p	miR-96-5p	miR-142-5p	miR-19-3p	miR-141-3p	miR-770-3p	miR-335-5p	miR-200-3p	miR-194-5p	miR-183-5p	miR-341-3p	miR-182-5p	miR-370-3p
			Fam193a															Slc25a40
			Atg7															Rxfp2
			Mms19															D16Ertd472e
			Robo4															Il1a
			Ubac1															Fbxw5
			Tnfaip8															Prcp
			Zfp532															Tnfrsf10b
			Gpn1															Slu7
			Apon															Hpse
			Dysf															Tkt
			Top1mt															Ube2u
			Chchd5															Fam58b
			Pole4															Cby1
			F13b															Rcl1
			Ccdc71l															Sycp3
			Hgfac															Chsy3
			Hacl1															Mycbp
			Plekhg5															Psma3
			Srek1ip1															Gm9956
			Rhod															Grhl1
			Aco1															Tmem220
			Polr2m															Psmb5
			Surf4															Amd1
			Cpn1															Hcfc2
			Txlnb															Rhpn1
			Rsl1															Nek11
			Mta1															Itga6
			Ddx31															Lrriq4
			Gcom1															Nrsn1
			Gprasp2															Mpv17l
			Slc38a10															Nadk
			Ugt2a3															Tmprss11f
			Adamtsl5															Prkaca
			Dld															Gda
			Myl9															Emc8
			Sugt1															Fam71e2
			Phlpp1															Zfp352
			D6Wsu163e															Tomm20
			Osbpl7															Vsx1
			Rnf208															Nusap1
			Kti12															Rnf220
			Smpd4															Bhmt
			Gyg															Pcsk1
			Comtd1															Nckap1
			Ict1															Gsn
			Ttc21b															Tcte2
			Rab4a															Smyd3
			Herpud1															Chic2
			Dgkg															Rps9
			Usf2															Hs6st2
			Ppp4r1															Efhb
			Nr1d1															Smim20
			Slc22a1															Ccdc158
			Rrp9															Mob4
			Flt3l															Gpr108
			Cyfip1															Cfh
			Hyi															Fam149a
			Cyp2d9															Isy1
			Sgcg															Arf4
			Tyw1															Afap1l1
			Naa25															Pfkfb2
			Tmem218															9130401M01Rik
			Taf1															Fam114a1
			Pcyt1a															Brd9
			Mrpl41															Nrg3
			Lsm4															Stat1
			Pex14															Chmp4b
			Cdc42ep3															Ifitm7
			Gc															Cript
			Adprhl1															Crcp
			Rpp25l															Rhoj
			Scarf2															Rab1
			Immt															Trim32
			Dut															Tyw3
			Casp12															Tyrp1
			Parp1															Usp12
			Hist2h3c2															Nprl3
			Plec															Gcnt1

miR-199-3p	miR-29-3p	miR-486-5p	miR-361-3p	miR-122-5p	miR-425-5p	miR-136-5p	miR-96-5p	miR-142-5p	miR-19-3p	miR-141-3p	miR-770-3p	miR-335-5p	miR-200-3p	miR-194-5p	miR-183-5p	miR-341-3p	miR-182-5p	miR-370-3p
			Ttc30b															Ror1
			Tbc1d1															Ppp2r2c
			Kifap3															Nepn
			Abl1															Acadsb
			Ift52															Plin3
			Ccdc108															Mavs
			Glrx2															Wdr82
			Kdelc2															Mpp3
			Serpinb8															Trem3
			Filip1l															Krtap4-1
			Cnnm3															Nxph3
			Nelfa															Gm10874
			Polr2f															Pou3f1
			Marc2															Sec61g
			Stk25															Ctc1
			Notum															Lemd1
			Cyba															Lacc1
			Otc															Rwdd3
			Ppp1r1c															Rbm4
			Ankrd42															Abcc5
			Fastkd2															Tomm40l
			Mrpl15															Aco1
			Slc22a12															Cyp2b9
			Hist1h4m															Cd247
			Serpini1															Tnnt2
			Nop10															Gm10784
			Polr3f															Grip1
			Zfp106															Stx12
			Gdf10															Cdo1
			Pspc1															Nubpl
			Tcp11															Dusp19
			Galntl6															Lyrm9
			Ift57															Enox2
			Tpgs1															Zfp286
			Tspan3															Tubb5
			Bfar															Nfatc1
			Glod4															Sell
			AI429214															Snrnp70
			Smg7															Ostn
			Tnnc2															Sdccag8
			Ikbke															Slc17a4
			Sf3a1															Akna
			Zfp160															Dusp23
			Usp5															Tax1bp1
			Ift46															Traf5
			Col5a3															Ywhaq
			Tmem176b															Heatr3
			Csf1r															Tmem140
			Zbtb49															Calm3
			Slc25a11															Rsl1d1
			Shpk															Tpk1
			Fdx1															Nkiras1
			Skp1a															Wscd1
			Tmem167															Sec24a
			Idh2															Magix
			Ttll12															Ms4a6c
			Ube2a															Snai3
			Ssfa2															Dlgap4
			Ifi27l2a															Slc25a1
			Rcsd1															Sema3b
			Mdp1															Arfgap1
			Rundc1															Zfp385c
			Jtb															Rnf222
			B2m															Tiprl
			Myot															Galr2
			Hpd															Gchfr
			S100a8															Lims2
			Nfu1															Dsel
			Tfdp2															Cyb5r3
			Ndufc1															Cnr2
			Rpl27a															Ldlrad4
			Pglyrp2															Alx1
			Trpm4															Gpr4
			Ints1															Vmac
			Fam135b															Hddc3
			Klhdc2															Glyr1
			Orc2															Iqcg
			Inpp5k															C2cd4c

miR-199-3p	miR-29-3p	miR-486-5p	miR-361-3p	miR-122-5p	miR-425-5p	miR-136-5p	miR-96-5p	miR-142-5p	miR-19-3p	miR-141-3p	miR-770-3p	miR-335-5p	miR-200-3p	miR-194-5p	miR-183-5p	miR-341-3p	miR-182-5p	miR-370-3p
			Sdpr															Cdc37
			Nrd1															Naca
			Wash															Ckap4
			Dtd2															Abhd17c
			Snx4															Man2c1
			Dock1															Letmd1
			Mta3															Zkscan4
			Ostf1															Pin1
			Ipo9															Synpo2
			Ttc32															Esp6
			Erlec1															Trim31
			Saal1															Tspan32
			Dnajb1															Dnase1l2
			Ugp2															Atp6v0d2
			Gnl1															Plxdc1
			Miox															Rcan1
			Ndufb2															Ccnf
			Sap30bp															6330419J24Rik
			Usp45															Uxs1
			Xpa															Rasgrp4
			Cul5															Zfp617
			Prox2															Dnaja4
			Ifnar2															Erlec1
			Terf1															Dmrt2
			Acy3															Nagk
			Med12															Dnmt1
			Tceb3															Slc4a3
			Arpc3															Ift74
			Dcps															Scgb1b10
			Wdr59															Zfp930
			Sec11a															Tmem204
			Eef1b2															Hsd3b7
			Fopnl															Gpr50
			Adra1d															BC024978
			Ppp1r36															Ly96
			Psmd11															Tmem163
			Zmym4															Hsf5
			AW112010															Gldc
			Apopt1															Pcsk9
			Ap1ar															Car5b
			Chd1l															Camk2d
			Chd7															Kiss1r
			Ccr8															Egf
			Cnih3															Txndc8
			Cp															Tob2
			Cldn10															Cdk10
			Fam162a															Trim9
			Ube2b															9130019O22Rik
			Itga3															Ptgr2
			Nuf2															Cln8
			Slmap															Tmem8
			Snrnp27															Scimp
			Pmpca															Frzb
			Trappc6b															Abcc3
			Aamdc															Khdrbs1
			Usp7															Ap1b1
			Rps24															Shfm1
			Cnbp															Ms4a8a
			Ces3b															Pdrg1
			Vps26a															BC030336
			Bptf															Olfml2b
			Ergic2															Atg4b
			Plaur															Best1
			Ffar1															Cyp2d10
			Them7															Lins
			Gm5819															Ube2e3
			Adck3															Slc38a9
			Lrrk1															Nkd1
			Gtpbp3															Phka2
			Foxl2															Actn2
			Naca															Tbrg4
			Cox6a1															Gm21685
			Pigx															Cers4
			Ptges2															Thap11
			Cep68															Sftpc
			Nudt13															Mblac1
			Zfp64															Fam120b
			Gabrb1															Slc5a8

miR-199-3p	miR-29-3p	miR-486-5p	miR-361-3p	miR-122-5p	miR-425-5p	miR-136-5p	miR-96-5p	miR-142-5p	miR-19-3p	miR-141-3p	miR-770-3p	miR-335-5p	miR-200-3p	miR-194-5p	miR-183-5p	miR-341-3p	miR-182-5p	miR-370-3p
			Rpp21															Limk2
			Trpc7															Pgm3
			Dlk1															Scrt1
			Tnfrsf21															Pabpn1l
			Slc25a4															Kctd15
			Lancl1															Serpina1f
			Nnt															Dmtn
			Acp6															Diexf
			Angptl3															Itga8
			Zfp94															Rad51d
			Mt1															Il15ra
			Siah3															Slc46a3
			Rnf169															Fkbp14
			Dgki															Polr1e
			Dera															Alg6
			D10Jhu81e															Flad1
			Myl7															Usp18
			Tagln2															Anapc5
			Lsm1															2310002J15Rik
			Tmem38b															Nmrk2
			Vps53															Hoxc4
			Tmem147															Art5
			Tmem242															Gm4794
			Dhrs3															Pomgnt1
			Oasl2															Gm4737
			Gja8															Rnf146
			Mrpl12															Lmo3
			Rpf2															Kcnt2
			Gimap1															Gmpr
			Ube2r2															Ttll1
			Errfi1															Unc119
			S100a6															B430306N03Rik
			Clcn3															Wisp3
			Twist1															Tmem19
			F10															Rassf9
			Aldob															Bhlhe41
			Serpina12															Pilra
			Krt7															Irak2
			Ppp1r9b															Gnrh1
			Cdh18															Zcchc9
			Clta															Ccl25
			Amfr															Lime1
			Ap5m1															Ces1f
			Dmgdh															Katnb1
			Hgd															Hmgcr
			Slamf7															Whamm
			Obfc1															Rgmb
			Kcnv1															Ergic1
			Scrib															Ces1b
			Rhou															Pou3f3
			Snapc5															Spsb2
			Rbm47															Txn2
			Zscan26															Baalc
			Sepw1															Utp23
			Sod1															Cacfd1
			Phc2															Phyhipl
			Abtb2															Rbm12b1
			Yeats2															Igsf23
			Pex1															Ophn1
			Rad51															Cln5
			Mrps31															Cnot6
			Serbp1															Mfng
			Fnta															Nat9
			Trim54															Gnb1l
			Ddx1															Proser2
			Itih1															Ocln
			Cyb5r4															Gstz1
			Tax1bp1															Cd248
			Ptms															Pdia4
			Lamp2															Slc25a46
			Tceal8															Rcvrn
			B4galt4															0610007P14Rik
			Dlgap2															Amigo2
			Ogdh															3100002H09Rik
			Trpc5															Gm17484
			Hps5															Vrk1
			Ppp2r2c															Hs2st1
			Hoxd11															Sbds

miR-199-3p	miR-29-3p	miR-486-5p	miR-361-3p	miR-122-5p	miR-425-5p	miR-136-5p	miR-96-5p	miR-142-5p	miR-19-3p	miR-141-3p	miR-770-3p	miR-335-5p	miR-200-3p	miR-194-5p	miR-183-5p	miR-341-3p	miR-182-5p	miR-370-3p
			Dcun1d5															Tmem218
			Dnajc25															Synj2
			Cox15															Pik3r6
			Aldh4a1															Lipt1
			Rpl19															Mbd3
			Ly6c1															Ngf
			Suox															Pla2g12a
			Rbp1															Slc22a3
			Bckdhb															Gfra4
			Gm5113															Bpi
			Dao															1700084C01Rik
			Fkbp15															Gapdh
			Fam192a															Gpr21
			Ubr4															Tgs1
			Apoa1															Fut4
			Rif1															Ino80c
			Eva1b															Mfsd2a
			Adck1															Cyp39a1
			Imp3															Pcdhb7
			Fuca2															Metrnl
			Cyp2s1															Cyp2c40
			Scrg1															Panx2
			Eif4e1b															Zbtb17
			Ndufb3															Llgl2
			Afg3l2															Gria1
			Eif4e2															Prkcsh
			Ascc3															Nipa1
			Lama3															Prpf38a
			Ptpn13															Trit1
			AW209491															Tspan11
			Dnm1l															Pigv
			Psmb4															Taz
			Aplp2															Dpt
			Qsox1															Vps53
			Sf1															Prkag3
			Ccdc174															Tcp11l2
			Gcat															Cd9
			Gtf3c2															Dupd1
			Prss16															Mefv
			Slco2b1															Tmem200b
			Rbm19															Ifltd1
			Ephb4															Dpf3
			Slfn8															Cdc73
			Egfl7															Odc1
			Plod3															Ssbp2
			Matr3															Zfp873
			Ddx56															Mtmr12
			Unc45b															Dysf
			Zfp706															Lnx2
			Yipf3															Ftcd
			Cnot8															Tspan31
			Ociad1															Rab19
			Tmco1															Ripply1
			Gys1															Cdh10
			Smarcc1															Exosc2
			Llgl2															Bcat1
			Mybpc2															Olfr20
			Mtcl1															Bgn
			Hace1															Hells
			Ddx60															Cldn22
			Pgap2															Wdr72
			Paf1															Rab10
			Top2b															Amdhd2
			Pam															Tnfrsf25
			Themis2															Ggh
			Cdk5rap3															Zfp444
			Commd8															Rpap3
			Psma6															Kifap3
			1700061G19Rik															Ranbp9
			Cap2															Ift88
			Rgs9															Fkbp4
			Zfp655															Bmp2
			Timm8b															Yaf2
			Hypk															Tbx2
			Cyp2b9															Immt
			Tmem248															Tfec
			Supv3l1															Asphd2
			Tatdn2															4932411N23Rik

miR-199-3p	miR-29-3p	miR-486-5p	miR-361-3p	miR-122-5p	miR-425-5p	miR-136-5p	miR-96-5p	miR-142-5p	miR-19-3p	miR-141-3p	miR-770-3p	miR-335-5p	miR-200-3p	miR-194-5p	miR-183-5p	miR-341-3p	miR-182-5p	miR-370-3p
			Fgf13															Letm1
			Sqrdl															Hoxa3
			Plscr2															Nfyb
			Kif13a															Ndufaf6
			Slco1a1															Stx11
			Cd38															Cyp2j5
			Smarca4															Kdelc1
			Zfp644															Impa1
			Uba3															Dnajb13
			Srgap2															Lhfp
			Senp2															Cib2
			Fam188b															Htr2b
			Sugp2															Ms4a15
			Oxld1															Ihh
			Csnk1a1															Mmp19
			Pik3c3															Dnajc9
			Tcp11l2															Eltd1
			Krtcap2															Fbxo25
			Tusc3															Pitpna
			Pkp2															Prss23
			Cep170															Slco3a1
			Trpc4															Aox3
			Ccdc154															Dnajc10
			Jund															Zfp474
			Sfpq															Gm16039
			Cyp2b13															Prune
			Optn															Frs3
			Itih4															Anxa7
			Pcnt															Lactb2
			Mrc2															Ubald2
			Mcm3															Ubb
			Yars															Tmem151a
			Slamf1															Cables1
			Mrpl20															Mapkapk2
			Ppp1cb															Banp
			Rint1															Armc10
			Coq10b															Trim62
			Hao2															Ppil1
			Golga3															Tnfsf4
			Acaca															Uox
			Scaf4															Trpc7
			Adnp															Tymp
			Cbs															Ptger3
			Ogn															Ccnk
			Rpl26															Anp32e
			Shmt2															Vtcn1
			Crip2															Fam92a
			Csnk1d															Sfn
			Trappc2l															Shisa7
			Brd9															Ppp1r7
			Fundc2															Necab3
			Papss2															1810037I17Rik
			Afg3l1															Aldh3a2
			Sftpb															Igfbp4
			Il21															Il21r
			Smdt1															Hspa5
			Gdf2															Ift57
			Myh6															Aifm1
			Ces1b															Imp3
			2310039H08Rik															Hsh2d
			Slc39a8															BC049352
			Aasdhppt															Slc25a38
			Psmd12															Ism2
			Mgat1															Fbln1
			Herc1															Lgi3
			Atp10a															Cd34
			Adat2															Ptdss1
			Tmem2															Slco1a1
			Wars2															Plk2
			Klhl31															B3gat3
			Gadd45g															Zbtb2
			Mios															Gabpb1
			Dnajc22															Hoxd11
			Zfp839															Kras
			Atp5o															Cybrd1
			Btla															Ankrd33b
			Cdpf1															Nmral1
			Fasn															Nudt7

miR-199-3p	miR-29-3p	miR-486-5p	miR-361-3p	miR-122-5p	miR-425-5p	miR-136-5p	miR-96-5p	miR-142-5p	miR-19-3p	miR-141-3p	miR-770-3p	miR-335-5p	miR-200-3p	miR-194-5p	miR-183-5p	miR-341-3p	miR-182-5p	miR-370-3p
			Dennd6a															Sptlc2
			Kctd3															Rrp15
			Plcxd1															Lrrn4
			Colq															Mnat1
			Mylk2															Dgkh
			Cltc															Idh2
			Commd2															Odf2l
			Pdzd2															Polr3d
			Apoa2															Eif4a3
			Degs1															Msn
			Otud7a															Hsph1
			Mbd5															Kera
			Hsph1															Prr18
			Rad52															Spata25
			Vps35															Fgf23
			Zap70															Snrnp35
			Dcc															Zc3h10
			Ecd															Cmtr2
			Tmem184c															Slc9a6
			Pcdhb1															Cstf1
			C1qb															Tmem184c
			Tiprl															Zfp846
			Pot1a															Ccdc42
			Grb7															Tbc1d25
			Tat															S1pr2
			Fgb															Gc
			Aldh1l1															Asb2
			Wdr75															Fbxl14
			Eltd1															Ubiad1
			Slc25a54															Gm20878
			Tgfb2															Slc25a11
			Pcm1															Rab21
			Ndrg2															Rangap1
			Sh2d4b															Uba5
			Snrpa1															AW549877
			Hilpda															2010315B03Rik
			Tada2a															Bves
			Abcg5															Tmem182
			Nphs1															Sidt2
			Zfp804a															Zfp14
			Cdk9															Polr3h
			Gm14399															Zfp318
			Mob2															Polr3g
			Bub1															Zfp191
			Plxnb3															Rab3b
			Synrg															Ttc30b
			Ptp4a3															Cd109
			Trip12															Repin1
			Top1															Fam179a
			Sergef															Rarg
			Snrnp70															Pcolce2
			Mfhas1															Sp110
			Kbtbd3															Atp2a2
			Galnt14															Eif2s1
			Lyz2															2510049J12Rik
			Pqlc3															Slc18a1
			Rnf11															Map2k4
			Rnf8															Dbndd2
			Mtmr9															Adal
			Slc25a46															Mrgprh
			Cyp2b10															Ak5
			Anxa3															Jup
			Zfand5															Emc10
			Slc8a2															Acnat2
			Mn1															Atp5b
			Mrpl3															Denr
			Arhgap5															Apoa2
			Hfe2															A930009A15Rik
			Herc2															Hist1h4c
			A230046K03Rik															Slc13a2
			Rpusd2															Myl6
			Vmp1															Gm12216
			Rbp4															Fosb
			Rfx7															Arl6ip6
			2900011O08Rik															Nqo2
			Luzp2															Ccdc92
			Spink3															Dram1
			Ptchd2															Cd72

miR-199-3p	miR-29-3p	miR-486-5p	miR-361-3p	miR-122-5p	miR-425-5p	miR-136-5p	miR-96-5p	miR-142-5p	miR-19-3p	miR-141-3p	miR-770-3p	miR-335-5p	miR-200-3p	miR-194-5p	miR-183-5p	miR-341-3p	miR-182-5p	miR-370-3p
			Snap47															Caln1
			Lyrm7															Zkscan16
			Gm9970															St8sia5
			Traf6															Fchsd2
			Acss3															Ip6k3
			Ighmbp2															AI597479
			Ppdpf															Loh12cr1
			Cox7a2															Elp4
			Bloc1s2															Ift46
			Zyg11b															Pmf1
			Slc25a3															Adat1
			Thoc3															Slc2a8
			Rnf34															Myoz1
			Eef1a2															Itih1
			Plxna3															Gm5113
			Emc6															Lancl1
			Aven															Pcbd2
			Fam134b															Ppip5k2
			Gpx3															4930503E14Rik
			Bcat2															Cdipt
			Neu3															Tial1
																		Tmem241
																		Unc45a
																		Zdhhc9
																		Gltscr2
																		Tyms
																		Rala
																		Ube2v2
																		Slc16a2
																		Wbp2nl
																		Klrb1b
																		Ephb2
																		Dync1i1
																		Scgb1b3
																		Aldh4a1
																		Pla2g12b
																		Bcap31
																		Gtpbp10
																		Msantd1
																		9130023H24Rik
																		Muc20
																		9130213A22Rik
																		Cln6
																		Il34
																		Ddhd1
																		Usp15
																		Fgfr2
																		Cyp51
																		Rab7
																		Afg3l2
																		Cobll1
																		Serpinh1
																		Ppp4r1
																		Zap70
																		Gp1bb
																		Sypl
																		Nsa2
																		Npm1
																		Tmem86b
																		Ten1
																		Gm906
																		Fbxw17
																		Hnrnpd
																		Mapkapk5
																		Lyn
																		Nckipsd
																		Acss2
																		P4hb
																		Clec14a
																		Ppdpf
																		Sptssb
																		4930550C14Rik
																		Smim19
																		Ndufaf7
																		Gm7932
																		Mrps26
																		Hars2
																		Cutc

miR-199-3p	miR-29-3p	miR-486-5p	miR-361-3p	miR-122-5p	miR-425-5p	miR-136-5p	miR-96-5p	miR-142-5p	miR-19-3p	miR-141-3p	miR-770-3p	miR-335-5p	miR-200-3p	miR-194-5p	miR-183-5p	miR-341-3p	miR-182-5p	miR-370-3p
																		Iars2
																		Pde12
																		Mrps18c
																		Tubd1
																		Prss44
																		Slc17a3
																		1810030O07Rik
																		Fgr
																		Znrf4
																		Hemgn
																		Cltb
																		Srgn
																		Cmc2
																		Sap30l
																		Nucb2
																		Gm16286
																		Chmp7
																		Bcl6
																		Shcbp1
																		Eif4g2
																		Them4
																		1700017B05Rik
																		Gm27029
																		Tmem38a
																		Hnrnpl
																		Lurap1l
																		Hist4h4
																		Bud13
																		Pcdhb8
																		Fbxl22
																		Sgms2
																		Dhfr
																		Kcnj3
																		Gusb
																		Acn9
																		Stoml1
																		Upp2
																		Gnb4
																		Cyp7b1
																		Zfp937
																		Ythdf2
																		Slc6a18
																		Gfm1
																		Gnl1
																		Slfn8
																		Plcd4
																		Plg
																		Cfl1
																		Tshr
																		Stx8
																		Slc39a5
																		Ctsb
																		Mlh1
																		Acox2
																		Tspan10
																		Mpl
																		Mtx2
																		Pyurf
																		Uba3
																		Eif5
																		Larp1b
																		BC037034
																		St6galnac2
																		Ampd2
																		Tssk3
																		Mbl1
																		Emc7
																		Ptgs1
																		Ubxn6
																		Emcn
																		Top3b
																		Dock11
																		Lfng
																		Cope
																		Lrrc4c
																		Polr1d
																		Utp11l
																		Emc2

miR-199-3p	miR-29-3p	miR-486-5p	miR-361-3p	miR-122-5p	miR-425-5p	miR-136-5p	miR-96-5p	miR-142-5p	miR-19-3p	miR-141-3p	miR-770-3p	miR-335-5p	miR-200-3p	miR-194-5p	miR-183-5p	miR-341-3p	miR-182-5p	miR-370-3p
																		Trmu
																		Arv1
																		Gpihbp1
																		Itgb7
																		Zscan12
																		Copg1
																		Pax8
																		Dhodh
																		Hgfac
																		Marc2
																		Twist1
																		Hcrtr1
																		Gm12184
																		Mrpl19
																		Cntfr
																		Tmem126b
																		Nars
																		Sppl2a
																		Trp53rk
																		Smndc1
																		Atg2a
																		Serpina3k
																		Sh3rf1
																		Dlg1
																		Prkar1a
																		Lamtor1
																		Cnpy3
																		Slc2a5
																		Klk13
																		Ces2b
																		Sumo2
																		Rnmtl1
																		Mdga1
																		Agk
																		Ubl7
																		Lcmt2
																		Efnb1
																		1810043H04Rik
																		Pdxk
																		Rnls
																		Tmem177
																		Frg1
																		Adssl1
																		Osgep
																		Commd2
																		Cmpk2
																		Ankrd2
																		Pln
																		Fbxo39
																		Susd3
																		Lmo4
																		Lenep
																		Me2
																		Rrp8
																		Mapk13
																		Ddx56
																		Rpusd2
																		Nell2
																		Eif2b5
																		Lair1
																		Me1
																		Cd209c
																		Acsl5
																		Gm10037
																		Tbx1
																		Pacrg
																		Hras
																		Fgfr1
																		Zfp623
																		Hnrnpll
																		Ing2
																		9030617O03Rik
																		Mrs2
																		Slc13a1
																		Fhl3
																		Tmem252
																		B9d1
																		Aldoc

miR-199-3p	miR-29-3p	miR-486-5p	miR-361-3p	miR-122-5p	miR-425-5p	miR-136-5p	miR-96-5p	miR-142-5p	miR-19-3p	miR-141-3p	miR-770-3p	miR-335-5p	miR-200-3p	miR-194-5p	miR-183-5p	miR-341-3p	miR-182-5p	miR-370-3p
																		Dok2
																		Rccd1
																		Cyp4v3
																		Nfu1
																		Sh3bp5
																		Kdelr1
																		Asb13
																		Cyp2d9
																		S1pr1
																		Gm5493
																		Mrpl51
																		Sgcb
																		Ciapin1
																		Chid1
																		Ccdc104
																		Nif3l1
																		Limd2
																		Prmt3
																		Fam221a
																		1700011H14Rik
																		Adhfe1
																		Hgsnat
																		Aarsd1
																		Hist1h3b
																		Zscan21
																		Tmem88
																		1190007I07Rik
																		Pigb
																		Zmym5
																		Zfp3
																		Krr1
																		Sdf4
																		Abcb8
																		Txndc9
																		Aldh16a1
																		Gm20390
																		Cept1
																		Clic4
																		Exo1
																		Ccdc135
																		Tubgcp5
																		Amy2a2
																		Rsu1
																		Arfrp1
																		Amy2a3
																		Amy2a4
																		Tomm70a
																		Tpm2
																		Zfp319
																		Ccdc17
																		Mrpl49
																		Lrrc57
																		Evx2
																		Dstn
																		Slc16a3
																		Spata6
																		Lst1
																		Mif4gd
																		Ctbp1
																		Mark1
																		Ncl
																		Zfp30
																		Mfge8
																		Zfp787
																		Mark3
																		Ccdc107
																		Ptbp2
																		Atp5a1
																		Dcun1d5
																		Gm14221
																		Styxl1
																		Sftpb
																		Tesk1
																		Dph3
																		Inip
																		Shq1
																		Fam210a
																		Cldn19

miR-199-3p	miR-29-3p	miR-486-5p	miR-361-3p	miR-122-5p	miR-425-5p	miR-136-5p	miR-96-5p	miR-142-5p	miR-19-3p	miR-141-3p	miR-770-3p	miR-335-5p	miR-200-3p	miR-194-5p	miR-183-5p	miR-341-3p	miR-182-5p	miR-370-3p
																		Cpt2
																		Ddrgk1
																		Stau2
																		Aqp2
																		Ptrhd1
																		Ttc39a
																		Masp1
																		Sqrdl
																		Mcm3
																		Wnt11
																		Clec2h
																		Prorsd1
																		Gm14124
																		Bdnf
																		Zfp566
																		Zfp68
																		Acnat1
																		Tspan1
																		Yipf7
																		Sart1
																		Faf1
																		Fkbp9
																		Tlr6
																		H1f0
																		Foxd2
																		Enkur
																		Pam16
																		Tex12
																		Plekhf1
																		Chtop
																		Spa17
																		Napsa
																		Cited2
																		Serpinc1
																		Tmem119
																		Acadl
																		Nop58
																		Ap1g2
																		Pfdn5
																		Cyp2j6
																		Tpst1
																		Lsm6
																		Ube2w
																		Rdh7
																		Ucp2
																		Rpp40
																		Marveld3
																		Dusp13
																		Zfp384
																		Mrpl39
																		Hsf4
																		Adprh
																		Pdcd1lg2
																		Grhpr
																		Cyp4a14
																		Ifi35
																		Ier2
																		Pik3c3
																		Chfr
																		Tnfaip8
																		Hace1
																		Rnf8
																		En2
																		Mrpl54
																		Ppp1r14a
																		Tarm1
																		Dhrs7b
																		Zbtb7b
																		Tmem248
																		Zfp94
																		Hspa2
																		Gclm
																		Nxt1
																		Gars
																		Pla2g5
																		Pspc1
																		Slc25a12
																		Pgd

miR-199-3p	miR-29-3p	miR-486-5p	miR-361-3p	miR-122-5p	miR-425-5p	miR-136-5p	miR-96-5p	miR-142-5p	miR-19-3p	miR-141-3p	miR-770-3p	miR-335-5p	miR-200-3p	miR-194-5p	miR-183-5p	miR-341-3p	miR-182-5p	miR-370-3p
																		Scly
																		Sorbs3
																		Spryd7
																		Mrps30
																		Tfam
																		Bhlha9
																		Mpi
																		Timm23
																		Spata5
																		Ncstn
																		Anapc11
																		Nr0b2
																		Ankrd39
																		Ccdc86
																		Tmem212
																		Rnf214
																		Nme2
																		Zfp850
																		Cdk7
																		Upk1b
																		Cyth2
																		Rtn4
																		Rtp4
																		Zfp639
																		Zfp933
																		Cdca4
																		D1Ertd622e
																		Ndufb11
																		Nelfa
																		Zfp689
																		Ppp2r5c
																		Prr3
																		Cct5
																		Tcn2
																		Timm10b
																		Tgm3
																		Txnl1
																		Yipf3
																		Sectm1b
																		Commd6
																		Cisd2
																		Eif3f
																		Ppp1r36
																		Fam187b
																		Ddx31
																		Dusp7
																		Ebpl
																		Ankrd42
																		Med8
																		Lsm4
																		Aldh1l1
																		Hao1
																		Lztr1
																		Cntnap5b
																		Agtpbp1
																		Umod
																		Cwh43
																		Efna4
																		BC089597
																		Mrpl11
																		Ticam1
																		Leprotl1
																		Pdzd11
																		Atg4d
																		Ndufa9
																		Mterfd2
																		Foxf2
																		Pddc1
																		Nhej1
																		Tpgs1
																		Ly6c1
																		Myl7
																		F2
																		Eif3e
																		Cyp8b1
																		Decr1
																		Timm8b
																		Nags

miR-199-3p	miR-29-3p	miR-486-5p	miR-361-3p	miR-122-5p	miR-425-5p	miR-136-5p	miR-96-5p	miR-142-5p	miR-19-3p	miR-141-3p	miR-770-3p	miR-335-5p	miR-200-3p	miR-194-5p	miR-183-5p	miR-341-3p	miR-182-5p	miR-370-3p
																		Tmem52
																		Hist1h2be
																		Timm17a
																		Ankrd9
																		Tmem209
																		Sub1
																		Dera
																		Cdk1
																		Plet1
																		Snap47
																		Zrsr1
																		Rsl24d1
																		Acsf3
																		Axl
																		Pea15a
																		Cyp2j13
																		Gng2
																		Hist1h4j
																		Cldn25
																		Atp9a
																		Cd226
																		Gm6871
																		Cyp2b10
																		Ncf2
																		Chrna9
																		Aven
																		Psmd1
																		Ugdh
																		Slc27a2
																		Nmd3
																		Obox5
																		Ackr3
																		Matn1
																		Tusc2
																		Cnpy1
																		Rps17
																		Rab29
																		Pck2
																		Oscp1
																		Asic5
																		Rnf208
																		Tbc1d15
																		Actr8
																		Pink1
																		Eef1a2
																		Iars
																		Cfdp1
																		Dbf4
																		Rps6ka6
																		Fgb
																		Anxa3
																		Fbxo36
																		Pet100
																		Cldn16
																		Wdr12
																		Fbxo7
																		Bloc1s4
																		Lmf1
																		Zfp78
																		Hint3
																		Ahsa1
																		Pfdn1
																		Fam47e
																		Srp19
																		Scp2
																		Slc7a13
																		Pglyrp2
																		Wdr18
																		Psma6
																		Ctdspl
																		Trem2
																		Eif1ax
																		Cxcl15
																		Zdhhc6
																		Oprm1
																		Trmt12
																		Pigc
																		Taf1b

miR-199-3p	miR-29-3p	miR-486-5p	miR-361-3p	miR-122-5p	miR-425-5p	miR-136-5p	miR-96-5p	miR-142-5p	miR-19-3p	miR-141-3p	miR-770-3p	miR-335-5p	miR-200-3p	miR-194-5p	miR-183-5p	miR-341-3p	miR-182-5p	miR-370-3p
																		Gdap2
																		Srpx
																		Zbtb3
																		Idnk
																		Csf1r
																		Gtf2b
																		Lsm12
																		Cdk9
																		Thpo
																		Gip
																		Cda
																		Med29
																		9430016H08Rik
																		Wdr5
																		Azgp1
																		Mterfd3
																		Gdf10
																		D830039M14Rik
																		Tmem115
																		Ntf5
																		Acsm5
																		Mrps9
																		Hfe2
																		Bnip3
																		Vmp1
																		Mrps17
																		Gng13
																		Snrpg
																		Ccbl2
																		Nr1h4
																		Csk
																		Trappc6b
																		Gm21992
																		Nostrin
																		Pdhx
																		Rpl21
																		Ephx2
																		2900011O08Rik
																		Zfp788
																		Mustn1
																		Ifrd1
																		Sf3b5
																		Il15
																		Acyp2
																		Lect2
																		Alkbh3
																		Mettl21c
																		Tnnc1
																		Eci1
																		Pigk
																		Urah
																		Clpp
																		Cmtm7
																		Rps26-ps1
																		Zbtb8os
																		Fus
																		Zmat2
																		Fam213b
																		Bri3bp
																		Samm50
																		Rplp2
																		Otulin
																		Gemin6
																		Edf1
																		Cdc42ep2
																		Cfhr2
																		Tmco1
																		Acot9
																		Alox5ap
																		Tmem242
																		Fah
																		Chpt1
																		Timp4
																		Pacrgl
																		Stk24
																		Kynu
																		Zfp791
																		Hoxc10

miR-199-3p	miR-29-3p	miR-486-5p	miR-361-3p	miR-122-5p	miR-425-5p	miR-136-5p	miR-96-5p	miR-142-5p	miR-19-3p	miR-141-3p	miR-770-3p	miR-335-5p	miR-200-3p	miR-194-5p	miR-183-5p	miR-341-3p	miR-182-5p	miR-370-3p
																		Triap1
																		Eva1b
																		Kti12
																		Oaf
																		Fbp1
																		Abhd6
																		Serpinb1a
																		Tmem52b
																		Stradb
																		Mgmt
																		Mrpl32
																		Mrpl1
																		Ly6a
																		Gsta3
																		Polr3k
																		Commd1
																		Cenpa
																		Tmem165
																		Lsmem1
																		Hfe
																		1700029I15Rik
																		Mettl21a
																		Utp3
																		Il18r1
																		Kcng2
																		Dnase2b
																		Mthfsl
																		Pcmt1
																		Psma2
																		Rbm12b2
																		Cyp4f13
																		Plod3
																		Cyb5r1
																		Cul4a
																		Cd8b1
																		Hgd
																		Tmem233
																		Ckm
																		Pigp
																		Gipc2
																		Ift22
																		Fuom
																		Gm5084
																		Mdp1
																		Camk2n1
																		AI467606
																		Oit3
																		Irx2
																		Acy3
																		Tssk6
																		F830016B08Rik
																		Fech
																		Ttc9c
																		Cox5a
																		Rad18
																		Pot1a
																		Bbip1
																		Aga
																		Ypel1
																		Adtrp
																		Ctf1
																		Zfp758
																		Fam35a
																		Gm2026
																		D3Ertd751e
																		Mrpl43
																		Nsun6
																		Bckdhb
																		Pigx
																		Lilra5
																		Snrpa1
																		Ccdc59
																		Mrpl30
																		Mfsd3
																		Gm5141
																		Anapc10
																		Dnajc15
																		Serpina1d

miR-199-3p	miR-29-3p	miR-486-5p	miR-361-3p	miR-122-5p	miR-425-5p	miR-136-5p	miR-96-5p	miR-142-5p	miR-19-3p	miR-141-3p	miR-770-3p	miR-335-5p	miR-200-3p	miR-194-5p	miR-183-5p	miR-341-3p	miR-182-5p	miR-370-3p
																		Cdk17
																		Eif4ebp1
																		AI462493
																		Gm14399
																		Hs3st6
																		Kcnv1
																		Rbp1
																		Nop16
																		Dpcd
																		Sod2
																		Rps6
																		Rps15a
																		Rps12
																		Mocs2
																		Rbm7
																		Cpn1
																		Ankrd13c
																		H2afj
																		Gtpbp4
																		Jdp2
																		Pecr
																		Aadac
																		Rsl1
																		Pycr2
																		Tysnd1
																		Serpina1c
																		Slc25a4
																		Bcl2l12
																		Memo1
																		2010107E04Rik
																		Fdx1
																		Cdk2ap1
																		Sertad1
																		Plscr3
																		Hadhb
																		Otc
																		Rpp21
																		0610009L18Rik
																		Tmem53
																		Aasdhppt
																		Tuba4a
																		Mbip
																		Zfp593
																		Smim5
																		Hax1
																		Tmem176b
																		Flt3l
																		H3f3b
																		Fcf1
																		Emb
																		Ccl9
																		Ak1
																		2310039H08Rik
																		Smpx
																		Mrpl53
																		Cyp2r1
																		Grpel1
																		Metrn
																		Elovl7
																		Adprhl1
																		Adk
																		Dap3
																		Lsm3
																		Cap2
																		H2-DMb1
																		Glod4
																		AU041133
																		Ccdc174
																		Rps3
																		3110040N11Rik
																		S100a11
																		Zfp467
																		Rpp25l
																		Mrps16
																		Polr2f
																		Mt1
																		Glrx
																		Smdt1

miR-199-3p	miR-29-3p	miR-486-5p	miR-361-3p	miR-122-5p	miR-425-5p	miR-136-5p	miR-96-5p	miR-142-5p	miR-19-3p	miR-141-3p	miR-770-3p	miR-335-5p	miR-200-3p	miR-194-5p	miR-183-5p	miR-341-3p	miR-182-5p	miR-370-3p
																		Snrpd3
																		Tmem205
																		Dpm1
																		Siah3
																		Ndufb2
																		Rer1
																		Tmem45a
																		Cisd1
																		Ifnar2
																		Ndufc1
																		Tmem192
																		Nol12
																		Cox14
																		Trappc2l
																		Cdc42ep3
																		Mrpl15
																		Ormdl1
																		Hibch
																		Upk1a
																		Ppp2ca
																		Cetn3
																		Cebpb
																		Tpd52
																		Rpf2
																		Hist1h4m
																		Fgf10
																		Klf2
																		Tusc3
																		Ugt2b36
																		Dynlt1a
																		AW112010
																		Cyba
																		Prim1
																		Igj
																		Ubl5
																		Sec11c
																		Myl10
																		Atp5o
																		Myl9
																		Malsu1
																		1700047I17Rik2
																		Mob2
																		Rpl37a
																		B2m
																		Ffar1
																		Crip2
																		Rgn
																		Vegfb
																		Gm15446
																		Apoc1
																		2610001J05Rik
																		Cox7a2
																		Sepw1
																		Tram1
																		Myl12a
																		Pepd
																		Ifitm2
																		Slc25a3
																		Fuca1
																		Pvalb
																		Kbtbd3
																		Zfp932
																		Phospho2
																		Nudt2
																		Adig
																		Alg14
																		Hspb11
																		Itm2b
																		Pnpo
																		9230110C19Rik
																		Slc22a1
																		Ndrg2
																		Egr4
																		Adck1
																		C1qb
																		Deb1
																		Nxph1
																		Gstm1

miR-199-3p	miR-29-3p	miR-486-5p	miR-361-3p	miR-122-5p	miR-425-5p	miR-136-5p	miR-96-5p	miR-142-5p	miR-19-3p	miR-141-3p	miR-770-3p	miR-335-5p	miR-200-3p	miR-194-5p	miR-183-5p	miR-341-3p	miR-182-5p	miR-370-3p
																		Ugt2a3
																		Gcat
																		Emc6
																		Vasn
																		Nxt2
																		6720489N17Rik
																		Slc7a6
