# Supplementary material for: A Panel of miRNA Biomarkers Common to Serum and Brain-Derived Extracellular Vesicles Identified in Mouse Model of Amyotrophic Lateral Sclerosis
Source: Mol Neurobiol. 2024 Jan 22;61(8):5901–15. doi: 10.1007/s12035-023-03857-z (PMC11249427; doi:10.1007/s12035-023-03857-z)
Supplement: Supplementary file 8 — Supplementary file8 (PDF 713 KB) [file 12035_2023_3857_MOESM8_ESM.pdf]

miR-5119	miR-671-5p	miR-486-5p	miR-451	miR-122-5p	miR-21-5p
Krtap26-1	Zfp704	Zc3hav1	Mex3c	Cux1	Gpr64
Esp24	Pdpk1	Map9	Osr1	Nol4l	Cpeb3
Lce1m	Tmem245	Gucy1a2	2210018M11Rik	Grhl2	Srl
Tnp2	Rgs8	Gabrb3	Cep55	Slc25a34	Klhl15
Ly6g	Tom1l2	Srsf3	Cox10	Slco5a1	Fam46a
Dcdc2c	Aak1	Gm12355	Trim66	Aldoa	Purg
Tmem161b	Gan	Mex3a	Psmb8	Vamp3	Prdm11
Ly6f	Prtg	Nalcn	Pak4	Slc12a6	Caskin1
Tmem218	Pard3b	Col6a6	Zfp644	Slc7a1	Plag1
4930430D24Rik	Rorb	Zfp281	Matr3	Nlgn3	Yod1
1700017N19Rik	Cplx2	Ccdc117	Gyk	Sh2d1a	Armcx1
Vmn1r45	Eif4ebp2	Nr2c2	Cir1	P4ha1	Epha4
Aknad1	Grin2b	Prrc2c	Psmd11	Gpm6b	Peli1
Dhrs13	Mllt1	Pik3r1	Cab39	Lnpep	Ppp1r3b
Zfp52	Prkcb	Wipf3	Gatad2b	Grem2	Mfap3l
Sptssb	Slx4ip	Pdpk1	Cdkn2d	Gm608	Dusp8
Zfp616	Uhrf1bp1	Cdh7	Fign	Slc41a1	Epm2aip1
F7	CDR1as	Ar	Vapa	Slc25a51	Sox5
Tmem167	Slc45a4	Gabra1	Tbx1	Ralgapa2	Nfib
Smco3	Gpd2	Armc8	Ywhaz	Ralgapa1	Armcx5
Mzt1	Zfp382	Arhgap5	Fbxo33	Ciita	Csrnp3
Car8	Zbtb4	Asb4	Ythdf2	Gm20489	Lcor
Gm9978	Hipk2	Celf2	Rer1	Ppip5k1	Zfp704
Epyc	Bend4	Lmo3	Dars2	Zfp282	Esyt2
Olfr1133	Ppargc1b	Dock3		Spef1	Rasgrp1
Dner	Bcl2l11	Gab2		Pdpr	Pbrm1
Six6	Alx4	Grhl2		Phf19	Tnrc6b
Gipc2	Cttn	8430419L09Rik		Bach2	Kdm7a
Tpbpa	Cnn2	Dlx3		Zfp827	Il6ra
Nyap2	Slc5a3	Tob1		Slc2a3	Thrb
Serpinb3c	Pappa	Ankrd13a		Kcnq5	Ppp1r3a
Timm9	Kctd12	Piga		Foxo3	Elf2
Msx3	4933426M11Rik	Agbl5		Arfip2	Osr1
Rnaseh2c	Gm6086	Crebrf		Gm6169	Ganc
A930004D18Rik	Csmd2	Bivm		Zscan29	Crebrf
Olfr67	Btbd9	Tsc22d2		Nfat5	Glis2
BC025920	Sort1	Aff3		Adamts2	Notch2
Wnt7a	Sytl5	Pdzrn3		Micu3	Ube2d3
Vimp	Mbtd1	Scn3b		Slc1a5	Hrk
Kcna4	Nt5c1a	Epha3		Ror1	Reck
Pate4	Creg2	Phf20l1		Csnk1g1	Tgfbi
Acnat1	Plekho2	Copg2		Ankrd13c	Ccl22
Mmrn1	Ssh1	Pten		Acap2	Gatad2b
Poglut1	Sec31b	Tenm2		Ptprb	Ski
Actr3	Arrb1	Ccdc66		Khdrbs1	Dkk2
Efcab12	Bmpr2	Gxylt1		Nhsl2	Kpna4

miR-5119	miR-671-5p	miR-486-5p	miR-451	miR-122-5p	miR-21-5p
Oxgr1	Cd33	Sp5		Atp11a	Kbtbd7
Smim9	Wnt9b	Slc10a7		Ptchd4	Nfia
Rdx	Ccl28	Tet3		Cdh6	Gid4
Acbd5	Zbtb20	4930533K18Rik		Ago1	Chic1
Rcn2	Rapgef1	Mamdc2		Gys1	1110002E22Rik
Ttc30a1	D130040H23Rik	Mark1		Galc	Fmo2
Fgfr1op2	Dlc1	Aak1		Meis2	Map3k1
Snx10	Mapt	Rhoq		5730596B20Rik	Bcl7a
Ccr9	Wdfy3	Snrpd1		Tbc1d10a	Bmpr2
Gm6483	Hic2	Pitx3		Zyx	Zfp367
Eprs	Atxn1l	Map3k7		Nt5c1a	Spg20
Tmed5	Slc1a2	Skiv2l2		Npas3	Edrf1
Smoc2	Rnf24	Slain2		Pkm	Mblac2
Lmx1a	C1qtnf1	Arl1		Akap10	Ptpn14
Iqcj	Urm1	Slc12a5		Tmem28	Tmem170
Figla	Nudt15	Phc3		Obfc1	Fasl
P2ry10	Ackr2	Nfat5		Tmcc3	Ppara
Serpinb6b	Gm17567	Abhd17b		Sox11	Mapk10
Hs3st5	Pou2af1	Plagl2		Ctdnep1	Tnpo1
Arfip2	Ptpn14	Hpse2		Evi5l	Plekha1
Tmem161a	Gabbr2	Chst3		Pmepa1	Klf3
Kif3b	Fmn1	Unc5c		Atp1b1	Fnip1
Cep57	Trim2	Arhgap44		Zbtb41	Klf6
Klf5	Tbc1d5	Nr3c1		Brpf1	Fmn1
4931400O07Rik	Plcxd2	Bahcc1		Tbc1d10b	Ntf3
Mtap	Ankrd52	Elavl2		Cpeb1	Stag2
Chrdl1	Kcnh7	B3gat2		Foxp2	Mprip
Samd5	FMN1	Timm23		Exoc6b	FMN1
Gm9938	Atf7	Cops7b		Snn	Pan3
Olfr968	Rnf150	Smad2		Map3k2	Zbtb41
Gpr12	Zfp871	Cnksr2		Gnpda2	Tsga10
Pus7l	Ubr4	Cabp2		Fundc2	Alx4
Gatm	Lypd1	Btaf1		Ocln	Rbms3
Mgat4c	Cdk12	Dcc		Inhba	Eif4ebp2
Snhg11	Ncdn	Glis1		Gm614	Il12a
Rpl15	Gm5423	Maml2		Aldh18a1	Jph1
Lilrb4	Snph	Kdm5b		Clic4	Acvr1c
Acnat2	Mapkbp1	Slc8a1		Fam117b	Rpa2
Pcmtd2	Ing5	Gpr153		Mllt1	Rnf24
BC052040	Ccbe1	Ppp3cb		Zfp46	Unkl
Psg22	Capn5	Cadm1		Rabl6	Fgf18
Gm5724	Satb2	Srsf1		Igf1r	Cux1
Trdn	Rab18	Dll4		Dennd2c	Spry1
Eltd1	Cdcp1	Gzf1		Hif3a	Gm8773
A330050F15Rik	A4gnt	Naa15		Cxcr3	Otud6b
Gm21092	Zfp618	Stk4		Kif2a	Pcsk6

miR-5119	miR-671-5p	miR-486-5p	miR-451	miR-122-5p	miR-21-5p
Lcp2	Lpp	Sypl		Cadm2	Xpo4
Lcn9	Slc6a5	Itgb3bp		Lrch3	Pitx2
Bri3	Xirp1	Stk35		Ppm1e	Smad7
4930524B15Rik	Wasf2	Pawr		Prickle2	Rmnd5a
Lgi4	Tmtc2	Ccdc47		Cs	Scml2
Akt3	Hs6st3	Siah1a		Acvr1	Cntfr
Slc7a15	Prdm11	Ipo7		Bhlhe41	Xkr6
Gbp4	Lgr6	Zfp827		Spry2	Ubr3
Tbc1d22a	Gcnt3	Pim1		Tmem150c	Suz12
Nox1	Nhsl2	Maml3		Maf1	Slc6a1
Rgr	RP23-180L12.5	Golga3		Sort1	Satb1
P2rx5	Celf1	Mettl1		G6pc3	Fcho2
Rs1	St8sia6	March4		Ccng1	Gramd3
Col9a3	Sfrp1	Atxn7l3		Mbnl1	Pvrl3
BC030500	E2f2	Dnajc21		Ccrn4l	Pik3r1
Ispd	Rbm41	Rassf3		Usp46	Stk40
Ms4a13	Ptprt	Tanc1		Idh3a	Pcdhb1
St8sia6	Prex2	Fgf9		Npepps	Arhgap32
Gabrg1	Atf6	Zfp507		Tns4	Ago2
Zfp874a	Ntrk3	Smoc1		Hnrnpu	Pcbp1
Skint3	Sec16a	Kctd1		Mars2	Dlgap1
Tm4sf1	Csnk1g1	H3f3b		Nkx1-2	Cdc25a
4930519P11Rik	Fev	Zfp644		Slc52a2	Mtap
Tmem72	Fam189a1	Irx5		Bai2	Dnajc16
Fam19a4	Gpam	Map7d1		Mmgt1	Trim33
BC007180	Nos1ap	D1Ertd622e		Trps1	Rell1
Nlrp4c	Kcna2	Fgf13		Adss	Rtkn2
Krtap4-13	Rs1	Bcorl1		Cdc42bpb	Purb
Zkscan2	Dvl3	Ccnt2		Slc4a3	Hipk3
Pramel1	Kif21b	Fam83g		Brwd1	Pag1
Ccnc	Gria1	Sox12		Rbm47	Zswim6
Scp2	Mga	Stau1		Nacc1	Myt1l
4930544L04Rik	Exph5	Dkk2		Negr1	Rnf103
Cckbr	Fam210b	Hnrnpa2b1		Nod2	Acat1
Gm11554	Lyve1	1810022K09Rik		Slc25a35	Pfkm
4933400A11Rik	Ppp2r5c	Nkx2-3		Cept1	Sox2
Gm11569	Fut4	Tmem115		Sgsh	Fbxo11
Nudt10	Crebrf	A230050P20Rik		Dlg2	Pura
Luc7l2	Pvrl1	Ptpn12		Foxk2	Ccl1
Dusp18	Fam101b	Psmb5		Hhip	Ror1
Gm21119	6030445D17Rik	Ctu2		Sox6	Cadm2
Gm15319	Adcy1	Tbx2		Tgif1	Sntb2
Dlgap2	E330009J07Rik	Dynll1		Olfml1	Jag1
Atg12	Hgd	3222401L13Rik		Stim2	Glcci1
Eva1a	Adam9	Mpv17l2		Sp2	Pcbp2
Rab11fip2	Zfp607	Elfn2		Pcdh7	Spry2

miR-5119	miR-671-5p	miR-486-5p	miR-451	miR-122-5p	miR-21-5p
Lep	Ago1	Rnf34		Tbr1	Arhgap24
Pde1a	Hes2	Idh2		Git1	Arhgef12
Efhd2	Chml	Rufy2		Hp1bp3	Asf1a
Paf1	Dusp8	Tmub2		Sesn2	Sox6
1700066M21Rik	Dennd1b	Surf2		Serp1	2700081O15Rik
Fam216b	Kctd16	Chd1		Adam10	Tnks
4930467E23Rik	Slc7a1	Aldh1a1		Cmtr1	Slmap
H2-T24	Mrgprg	Rpl37a		Gramd3	Npas3
Cep135	Cacna1h			Pitx2	Timp3
Alg2	Tbc1d4			Stx6	Chd7
Nxf3	Tmem136			Papola	Fubp1
Brms1l	Slc7a14			Gata4	Rspo2
Sptssa	Xrn1			Ccdc97	Yap1
Olfr173	Plekhm1			Calm3	Fam63b
Cysltr1	N4bp1			Slc15a1	Slc30a10
Mettl14	Samd7			Dand5	Cnot6
Zfp667	Tmem201			Syt7	Klhl42
2810403A07Rik	Mfsd10			Braf	Lrrc57
S100a10	Clec16a			Mipol1	Rffl
Nps	Vps52			Plcb3	Sfmbt1
5830403L16Rik	Trim66			Mtf2	4932411E22Rik
Cpne9	Tmem127			Map3k12	Aim1l
Trmt1l	Adamts20			Mapre1	Ube2d2a
2310034C09Rik	Sarm1			Pigv	Rbpj
Bag3	Col8a2			Hist1h3h	Rps6ka3
Impad1	Asxl3			Amigo1	Pdcd4
Wdr45b	Oas1a			Rad21	Mapre1
Apln	Hecw2			Ank1	Tiam1
Gxylt1	Prrg3			Morc4	Trpm7
Gm13247	Gpr152			Msn	Nfat5
Evi2a	Uhmk1			Vsig10l	Tagap
4931406C07Rik	Capn6			Fam219a	Srsf3
Leprel4	Esr1			Tfdp2	Kcna1
Fam111a	Zfhx2			G3bp2	Strn
Trpc5	E030010N08Rik			Slc9a1	Lats1
Psmc6	Cnnm4			Iqgap1	Paip2b
Cmtr2	Ano1			Tmem87a	Tagap1
Srp14	C130074G19Rik			Kif5b	Zfp36l2
Edil3	Nkx3-1			Galnt1	Dnajb14
Lyrm5	Fat4			Fkbp5	Mbnl1
Pnp	C2cd2			Lrp10	Pspc1
Tex12	Il17rd			Ms4a6c	Ppp3ca
Zc2hc1a	Zyg11b			Dlat	Tns1
Gjd2	Sv2c			Senp1	Foxp1
Gpr21	Frrs1l			Large	Thbd
Styk1	1700066B19Rik			Gtf3c2	Sgms2

miR-5119	miR-671-5p	miR-486-5p	miR-451	miR-122-5p	miR-21-5p
Gm9989	Pacsin1			Epo	Eml6
Atp1b4	Sacs			Slc39a8	Ap3m1
Dnah14	Anks1			Ccdc6	Mycl
Hsf3	Zdhhc21			Clic5	Gm10282
Cyb5d2	Fzd1			Atp6v1h	Cd44
Vmn1r59	Dhcr24			Dicer1	Mef2c
Lilra5	Cacul1			Mrrf	Foxp2
Fam46c	Mecp2			Bdnf	Slit2
Cenpw	Lrch3			Hdhd2	Matn2
Mpp7	Rab6b			Ihh	BC030336
Gpbp1	Tcf23			Mef2d	Stat3
Gm9913	Esrrg			Ptbp2	Alx1
Frs2	Wscd2			Csk	Megf9
Tex28	Pde11a			H1f0	Samd8
Islr	Cacng1			Plch1	Acvr2a
Dicer1	Gtpbp2			Stk24	Map2k3
Zim1	Tifa			Oprd1	Ralgps2
Erich4	Ppm1l			Dlg4	Bcl2
Gp49a	Tirap			Cbl	Rbm25
Manf	Lair1			Dhfr	Olr1
C2cd5	Lhx4			Ms4a6b	Bcl11b
Arhgap18	Fat3			Gatad2b	Nbea
Thoc6	Padi2			Gas2l2	Setd1b
Olfr976	Samd12			Daglb	Wwp1
Rbmx2	Edaradd			Ric3	Hgf
Sval1	Gucy2e			Gabrb1	Rdx
Zfp653	Sufu			Tmem126a	AI593442
Gstcd	Hsf4			Haao	Fbxo28
Yipf5	Mbp			Zfp689	Ssfa2
Ap1s3	AI597479			Shisa4	Gata2
Ppt1	Chrm1			Emx2	Tet1
Rpl13a	Siglec15				Gm12355
Fam154b	Vangl1				Lemd3
Elmod2	Stc1				Rhob
Mtcp1	Ube2z				Fam126b
Rgs10	D130052B06Rik				Adnp
Cnga2	Vwa5b1				Pom121
Mob4	Tns3				Bahd1
Fstl5	Dtx4				Hs3st1
4930500M09Rik	Padi1				Tnfaip3
Tmem177	Camk1d				Rsbn1
Rhox8	Prx				Cdk20
Abcc12	Cebpa				Cadm1
Slc23a3	Strip2				Snx29
Gm5585	Lgi2				Serpina7
Dnah3	Cybrd1				Sez6l

miR-5119	miR-671-5p	miR-486-5p	miR-451	miR-122-5p	miR-21-5p
Pdc	Zfp46				Zcchc3
Ube2d2b	Jade2				Itga8
Psg17	Arhgap26				Gpr125
AA792892	Sh3pxd2a				Rasa1
Tdrd7	Ntn1				Dcun1d3
Gm9830	Tnrc6b				Ppp1cc
Sept2	St6galnac3				Pdzd2
Rpl38	Lmx1b				mt-Nd2
Rnf146	Liph				Lgr4
Ntn4	Phactr1				Crebl2
Sln	Gpr182				Iffo2
1110037F02Rik	Fam102b				Mdga2
Hddc2	Tmem229b				Sox7
Mfap3l	Paqr4				Fbxl17
4632415L05Rik	Magi3				Btg2
Slc5a3	Elfn2				Itga1
Slc30a8	Dpp10				Ap1ar
Glo1	Tenm1				Bnc2
Fut4	Rassf4				Frs2
Ccdc147	Zfp114				Pgrmc2
Fam86	Trim67				Tbx2
Gm9799	Crkl				Pikfyve
B3gnt5	Fads1				Ehd1
Ercc8	Gid4				Tanc1
Hist1h2bj	Mturn				Matr3
Nlrp4f	Ildr2				Tgfbr2
Dbr1	Slc25a44				Gprasp2
Clec2d	Cd34				Sema3a
Nap1l2	Rad51b				Ap5m1
Ptma	Decr2				Etv1
Acadm	Gucy2f				Rab22a
Rnf39	Fto				Cbx4
Klrc2	Spock1				Hmgn2
Sox2	Lrrc32				Sec63
Med7	Ankrd28				Prpf4b
Ppp1r1c	Acaca				Kdm2b
Gm9956	Syngap1				Crim1
Cacng6	Prickle2				9930021J03Rik
Spock2	Spry4				Otud3
Lin28b	1700052N19Rik				Cxcl10
Izumo4	Vsig10l				Gng12
Ccdc62	Cbx7				Krit1
Nfatc1	Olfr1512				Arhgap5
Steap2	Fam78a				Per2
Ugt1a5	Txlna				Akirin1
Ugt1a9	Rell1				Adtrp

miR-5119	miR-671-5p	miR-486-5p	miR-451	miR-122-5p	miR-21-5p
D130052B06Rik	Sp7				Zadh2
Ugt1a6b	Slc39a14				Dnaja2
Ugt1a10	Atxn7l1				Tinag
Ugt1a2	Ttc7				Msx1
Ugt1a6a	Slc24a2				Rimbp2
Ugt1a1	Dnmt3a				Rqcd1
Ugt1a7c	Stx1b				Eif1ax
Ugt1a8	Rab17				Cdk6
9230109A22Rik	Tapt1				Rab11a
Snap25	Smg1				Kcnt2
9430020K01Rik	Fsd1l				Pja2
Paip2b	Sorcs1				Rasa2
Twistnb	Cdk2ap2				Emc6
Fam126a	Mcu				3632451O06Rik
Gm10093	Dcp1a				Rnf111
C730034F03Rik	Mapk8ip3				Nrg3
Kera	Lemd2				Psd4
9430038I01Rik	Fam163a				Mrpl49
Api5	Arl14epl				Hnrnpu
Gm5148	BC094916				Mfap1b
Phf7	Pcnx				Slc6a20b
Tyrobp	Vkorc1l1				mt-Nd1
Fam19a1	Rag1				Cyp51
Ppp2r2b	Scn4b				Sod3
3632451O06Rik	Src				Vash2
Plaur	Mroh6				Il21
Polr3gl	Scn2b				Zfp385c
Cdh8	Fbxl16				
Car2	Rere				
Rabif	Golim4				
Dennd6a	Ankdd1a				
1700028P14Rik	Ttc26				
Nicn1	Nme6				
Slamf8	Katnal1				
Cd200r3	Tmem135				
Lamp2	Prune2				
Nup35	Galc				
Mad2l1bp	Prelp				
Fam13a	Sh2b3				
Atp6v1g1	Nptx1				
Klrc3	Tbl2				
Scd1	Ighg2c				
Oas1d	Clip2				
Lyve1	Dagla				
Cdkl1	Bahcc1				
Hmgb2	Slc36a1				

miR-5119	miR-671-5p	miR-486-5p	miR-451	miR-122-5p	miR-21-5p
Nbn	Igf1r				
Gm11595	Abcc10				
Gm16223	Itga4				
Saa3	Cntn5				
Vmn2r42	Wnt2				
Tmprss11a	Slit3				
Sparcl1	Lbh				
Gm5128	Slc12a7				
Cdkl2	Plek				
Olfr519	Limd1				
BC022687	Gm9881				
4933440M02Rik	Csmd1				
Mrpl43	Rnf38				
Palm2	Ar				
1700013H16Rik	Zcchc2				
Herc6	Klf13				
Lgr5	Gm608				
Plk3	Mesdc2				
2010315B03Rik	Itpkc				
Wdr34	Sntb1				
Ranbp10	Nfatc2ip				
Khsrp	Fam129c				
Phf1	Timmdc1				
Ociad2	Abce1				
Nat3	Map3k13				
Tssc1	Mov10l1				
Fam163a	4833424O15Rik				
Pot1a	Slc25a10				
Nphs1	Prlr				
Zfp607	Gbp11				
Nrsn1	Slc39a12				
Xkr8	Cnot4				
Oat	Ugcg				
Ankrd26	Erbb4				
Armcx1	Scd1				
C330011M18Rik	Cxcl13				
Ccl28	Bmp10				
C1qc	Map3k9				
Slco1b2	Ap1s3				
Carf	Nebl				
Gm11562	Hpcal4				
F3	Camkk2				
Slc4a10	Mtus2				
Ostf1	Kirrel				
Scn7a	Unc5d				
Flrt2	Rnf144a				

miR-5119	miR-671-5p	miR-486-5p	miR-451	miR-122-5p	miR-21-5p
Zfp955a	Tspan7				
Htr2a	Coro2b				
Trim68	Maml3				
Slc4a4	Lrrk1				
Sigirr	Nav2				
Esm1	Vgll4				
Gramd1c	Snx10				
Dnajc1	Grk1				
Tbc1d15	Ksr2				
Tnfaip2	Hnrnpul1				
Slc31a1	Klhl18				
Chmp5	Ctif				
Nipsnap3a	Ammecr1l				
Nubp2	Znrf3				
Ist1	Zscan22				
Ppip5k2	Fam83f				
Ttc19	Gm16485				
Ccdc51	Tbc1d24				
Sntg1	Foxo3				
Mrpl18	Bace1				
Mtmr4	Acadsb				
Gm13088	Pdgfra				
Clec1a	Uvssa				
Tmem50b	Lyg2				
Tnfsf14	Entpd7				
Chac1	Gatad2a				
Rad54b	Syt15				
Pcdhb12	Cpne2				
Gm10874	Jmjd8				
Nfkbie	Myo18a				
Oog1	Asb7				
Rhot1	Rab42				
Proser1	Sh3bgrl2				
Rpl27a	Nhlrc3				
Lysmd2	Slc35d1				
Tax1bp1	Chtf8				
Gm13103	Fbxl18				
Tmem52b	Fam160b2				
Galnt12	Megf8				
Gm17174	Lypd6				
Mlycd	POU2F1				
Maob	Tgif2				
Pip4k2a	Rora				
Ak9	Otud5				
Hist1h2bp	Plekha5				
Hdac1	1110002E22Rik				

miR-5119	miR-671-5p	miR-486-5p	miR-451	miR-122-5p	miR-21-5p
Hps4	Tfap2b				
Ccl9	Polr3e				
Tnfaip8l3	Nlrc3				
Arxes2	Tex2				
Bhlhb9	Klf7				
Zic5	Helz2				
Lelp1	4632428N05Rik				
Fscb	Abhd2				
Fzd4	Bnc2				
Armc10	Rps6ka3				
Adarb1	4833403I15Rik				
Dnmt3a	Zcchc14				
Npy4r	Cdh6				
Nkain2	Slc6a1				
Prpf4b	Stox2				
Gadd45a	Fam217b				
Gm4861	Fndc7				
Hsph1	Nkx2-1				
4932442L08Rik	Ptpn1				
Meox1	Slc17a5				
Neurod2	Scai				
Fam133b	Ppp1r12b				
Gipc1	Tnks				
AW112010	Znrf1				
Myo1b	Dlx2				
Psd2	Fnip2				
Rex2	Pabpc4l				
Ppp3cb	Atp6v1a				
Ephx2	Pfkfb4				
Dera	Rfng				
Sord	Sstr2				
Oog3	Gys1				
Olfr482	Wfikkn2				
3830408C21Rik	Cry2				
Ttbk2	Eda				
4933434E20Rik	1810013L24Rik				
Gm17590	Fgf4				
Fgd6	Atp2b2				
Sh3bgrl2	Cbfb				
Gm10436	Kcna6				
Fadd	Mroh7				
Elfn2	Ptpdc1				
Adprh	Slc22a23				
Ppef1	Hs1bp3				
Vmn2r37	Stk36				
Pgr	Fam219a				

miR-5119	miR-671-5p	miR-486-5p	miR-451	miR-122-5p	miR-21-5p
Tcerg1l	Rbbp5				
Fam228b	Rap1gap2				
Csn1s2a	Ciita				
Stard4	4732440D04Rik				
Dkk2	Add2				
Il18bp	Jade1				
Jmjd8	Grip2				
Mob1a	Tmppe				
Uspl1	Zc3h12c				
Cpeb4	Kdm5a				
Aqp9	Sephs1				
Itch	Fras1				
Lats2	Mrgprb1				
Gm13242	Arid5b				
Ube2v2	Ppp1r18				
Zfp955b	Ociad2				
Ccdc92	Taf8				
Iscu	Zeb2				
Syt9	Fkbp14				
Btbd3	4930538K18Rik				
Dcun1d3	Rab11fip4				
Ctnna1	Sypl2				
Morn4	Mtor				
Tph2	Tsc1				
Svopl	Dhrs9				
Gsdmc	Fam161b				
1700018C11Rik	Xpo4				
Amdhd1	Zfp281				
Rel	Samd5				
Def6	Rimkla				
Ccdc59	Cyth3				
Yipf7	Foxj2				
Cers6	Grid2ip				
Gm17484	Tnpo2				
Hist3h2ba	Trnp1				
Zscan29	Gm1968				
Gm10767	Tmem216				
Ptbp3	Fbxo28				
Chrna3	Ypel2				
Plekha1	Sh3pxd2b				
Rmi1	4933433C11Rik				
Zfp128	Hunk				
Cnga1	Papln				
Mpv17l	Zfp667				
Grwd1	Syn3				
Gm6625	P2ry10				

miR-5119	miR-671-5p	miR-486-5p	miR-451	miR-122-5p	miR-21-5p
Irgm2	Zak				
6030445D17Rik	Sav1				
Camk4	Ak4				
Atf7ip2	Arid3a				
Mef2d	Ccdc15				
Casp16	Abhd4				
Cbln3	Speer4a				
Yy2	Tmem80				
Gm10000	Shisa7				
AA474331	Surf6				
Agbl2	Fndc5				
Arl6ip6	Kcng4				
Zdhhc24	Mrpl45				
Galnt1	Plcb2				
Fam63b	Pcdh11x				
Tmem41a	Mafg				
Insm2	Phf19				
Asf1b	Draxin				
Hbq1b	Crtc3				
Gm5464	B4galnt1				
Ermap	Neu2				
Gm10742	Setd7				
Shisa3	Fhl2				
Zfp600	Hdx				
Lats1	Tet3				
Topbp1	Gmeb1				
Sesn1	Garem				
Sypl	Ado				
Egf	Phip				
Cdh6	Klhl25				
Gm9825	Sept11				
Akr1b7	H13				
Coprs	Sepsecs				
Gtf3c4	Kbtbd11				
Mdm4	Gm9845				
Ppp1r14b	Rad51				
Swsap1	Foxp2				
BC049635	Ppm1f				
Olfr1033	Mab21l3				
Itfg1	C9				
Lypd1	Mbtps2				
Spsb2	Ltbp2				
Gm13023	Snhg11				
2810417H13Rik	Nipal1				
Hspa2	Desi2				
Tmem229a	Selplg				

miR-5119	miR-671-5p	miR-486-5p	miR-451	miR-122-5p	miR-21-5p
Naa30	C8b				
Cnot4	Arih1				
Bmp15	Fbxl20				
Gm5549	Gpr146				
Acadl	Tmem151b				
Ttc9	Srl				
Ccdc66	Col4a6				
Hccs	Rbm20				
Dmbt1	Mrgprx2				
Trim43b	Ap2m1				
Cpsf4l	Mzf1				
Pck1	Cox10				
Lect2	Arnt2				
Tmem17	Ankib1				
St6gal2	Nfam1				
Rnaseh1	Osbpl6				
Klri2	Aim1				
Slc7a11	Tnfrsf1b				
4930453N24Rik	2900026A02Rik				
mt-Nd6	Cacna1e				
Tmed7	Socs2				
Sema6d	Dcx				
Elavl2	Smim17				
Ripply3	Fignl2				
Pja2	Ryr2				
Tmem30a	Htr5b				
Dnajc28	Ncald				
Pkig	Arid3b				
Cyb5b	Ambra1				
Frem1	Mcat				
Olig3	Cd79b				
Ankrd66	Ppp1r10				
Tbx15	Pyhin1				
Miip	Tmem56				
C1qtnf3	Cramp1l				
Npy2r	Iqsec3				
9930022D16Rik	Mib1				
Abcc9	Prok1				
Epsti1	Hsdl1				
Arfgap1	Map3k2				
Efr3a	Ndst3				
Psg25	Ttc34				
Fam210a	Asxl2				
Slc25a44	C1qtnf9				
Tram1	Gm10134				
Tmem8b	Ptpn7				

miR-5119	miR-671-5p	miR-486-5p	miR-451	miR-122-5p	miR-21-5p
Rrbp1	mt-Nd6				
Tatdn2	Fam129a				
Dexi	C1qtnf6				
Got1	Prss57				
Hfe2	Bsn				
Gbe1	Lgalsl				
Zyg11b	Pex5l				
Fam180a	Ccser2				
Itga7	Osbpl2				
Pcmtd1	Hrnr				
Wdr43	Slc12a8				
AI593442	Hif3a				
Thap1	Ctnnd1				
Etnk1	Rtkn2				
Mipol1	Creb3l2				
Rnps1	Marveld1				
Cilp2	Xkr5				
Sp9	Rnf182				
Klrc1	CTNND1				
Rbpms2	Gprc5b				
Slc25a40	Amer2				
Stxbp4	Shisa9				
Slc38a11	Svop				
Vmn1r43	Tdrp				
Zfp709	Gm16515				
Ecscr	Armc9				
Sash3	Atp2b3				
Zfp367	Dock3				
Rabgap1l	Vps26b				
Arf4	Frmd4b				
Mrps21	Mkl2				
Ipo11	Adcy9				
Vmn2r40	Srgap2				
Il7	Nat6				
Ccdc50	Irf4				
Efcab2	Ptk6				
Gm10097	Hus1				
Abhd5	Zfp185				
2610028H24Rik	Sgcd				
Nip7	Ptprb				
Zfp260	Prkcz				
Snx12	Mtfr2				
Pttg1	Tef				
Hormad1	B3gat1				
Oas1f	Sbno1				
Slc35g3	Zfp287				

miR-5119	miR-671-5p	miR-486-5p	miR-451	miR-122-5p	miR-21-5p
Fmo9	Fv1				
Gpr171	Pygb				
Zadh2	Nlrp1b				
Cdh9	Col25a1				
Zfp395	Sec24c				
Dcun1d1	Ppp1r3d				
Rnf170	Nacc1				
Lmln	Ino80d				
Cyp4x1	Btbd11				
Gpr85	Baz1b				
Rnaset2b	Tnik				
Dzip1l	Anks6				
Cyfip1	Glg1				
BC051628	Ttll4				
Zfp712	Elovl6				
Rgs16	Zfp652				
Paqr6	Otx1				
Vmn2r34	Kidins220				
Tmem236	Bhlha15				
Parp12	Osbpl3				
Kdm1a	Zfp655				
Nipal4	Kcnk6				
Pcbp2	Syne3				
Fam19a3	Vsig4				
Vmn2r45	Sept6				
Gm10302	Idua				
Tstd3	Ppara				
Slc19a2	Atp8b2				
Tnfrsf13c	Cers1				
Esr1	Phf20				
Prdm4	Grin1				
Ercc6l2	Dnajc27				
Prex2	Dnajb5				
Spag1	Slc6a11				
Hey2	Zfp366				
Uxs1	Ccnt2				
Lrrc3	Map3k3				
Invs	Klb				
Xaf1	Greb1				
Mrpl49	Gcnt4				
Asb13	Lifr				
Foxi3	Bmp8a				
Ascl1	Armc8				
D130040H23Rik	Ormdl3				
Pi4k2b	Lpgat1				
Nlrp4e	Cdc7				

miR-5119	miR-671-5p	miR-486-5p	miR-451	miR-122-5p	miR-21-5p
Vmn1r58	4932414N04Rik				
RP24-392D11.2	Smcr8				
C5ar2	Guca1b				
Ythdc2	Fbxl14				
Msl3	Taf13				
Slc24a3	Serinc5				
Trpd52l3	Scube3				
Serpinb2	Slc38a6				
Cylc2	Dgke				
Zbtb2	Prrt2				
Lrpap1	Csta1				
Tmem263	Ctnna3				
Calu	Erc2				
Chd9	Zbtb46				
Ms4a2	Rasl12				
Cxcl5	Cdon				
Magel2	Rhot2				
Ccar1	Brwd3				
Ccdc132	Pcyt1a				
Themis	Rph3al				
Fbxl3	Suclg2				
1810011H11Rik	Klf6				
Gpcpd1	Zfp651				
Cbx3	Tgfbr3				
Uvrag	Cuta				
Atad2b	Melk				
Cyp4b1	Adamts2				
Trip10	Lgi4				
Hnrnpa2b1	Smarcc1				
Scamp5	Trak1				
Dennd2c	Gsk3a				
Gm10309	Gad2				
Sv2b	Wipf2				
Slc30a5	Nfatc4				
Jade1	Kcnk5				
Gabra4	Gm15800				
Abhd12b	Tk2				
Lrp3	Kcnh1				
Oprd1	Ralgapa2				
Zdhhc13	Tmem86b				
Gm10330	Celsr3				
Olfr354	Apcdd1				
Hoxb13	Bend3				
Gja1	Arl5b				
Arcn1	Lig3				
Colec10	Gm4767				

miR-5119	miR-671-5p	miR-486-5p	miR-451	miR-122-5p	miR-21-5p
Syt4	Gm10778				
Socs2	Lactbl1				
Mpz	Krtap5-2				
Eda	Gm5784				
Ssfa2	Pcdh20				
Gm10010	Itga10				
Ndrg3	Pip4k2b				
Pdyn	Fcrls				
Birc5	Galnt15				
Ctnna3	Otud6b				
Efnb2	Mfap1a				
Zbtb6	Sema4f				
Ccne2	D17Wsu92e				
Gm5868	Tle6				
Ppp2r2a	Cast				
Zfp658	Tab1				
Sumo3	Zc3h12b				
Gpr173	Itpk1				
Tnfsf10	Fgf1				
Adsl	Gm10654				
Lypla1	Rpa1				
Zscan30	Mid1				
Armcx4	A530084C06Rik				
Mctp2	Epgn				
Gm3327	Poldip3				
Zfp764	A430105I19Rik				
C9	Oas2				
Hsd17b11	Dlgap1				
Hspa13	4933402N03Rik				
Hap1	Enpp4				
Thap2	Parvb				
Ccdc43	Dpy19l1				
Ccr2	Fzd5				
Ttc32	Ablim2				
Gm17509	Tshz2				
Gria4	Zfand4				
Tmem233	Pfpl				
Gm10300	Mcc				
Grm6	Tnfrsf23				
Stx12	Prrc2a				
Sdpr	Pcdh17				
Lrp8	Col11a2				
Rbm41	Cyfip2				
Mmab	Dnah10				
Hp1bp3	Zfp41				
Siglech	Syvn1				

miR-5119	miR-671-5p	miR-486-5p	miR-451	miR-122-5p	miR-21-5p
Nebl	Rbfox3				
Gpt2	Smg5				
Zfp191	Hmgxb3				
Cggbp1	Ttc28				
Rhoq	C2cd4c				
Gm4894	Gabrg3				
Dpp10	Plxna1				
Gabpb2	Slc7a11				
Ect2	Pde1b				
Spcs3	Sertad2				
Tpcn2	Chp1				
Clspn	Mfap1b				
Soat1	Slc25a51				
Zfr2	Gata2				
Pilrb2	Dao				
Thbs2	Cyp26b1				
Ugt8a	Nfya				
Clec2g	Pcnxl2				
S100b	Cldn15				
Cd33	Rnf144b				
Krtap21-1	Epdr1				
Adat1	Cnot7				
Vegfa	Mapre3				
Ccdc117	Ykt6				
Tgfbr2	Mrgpre				
Gtdc1	Ubr7				
Olfr456	Npas3				
Lamc2	Nlrx1				
Gm14214	Npffr1				
Usp33	Atxn7				
Wdhd1	Myo7a				
4921522P10Rik	Smarcd1				
D17Wsu92e	Gpr21				
Tas1r1	Cerk				
Tbc1d10c	Arhgap32				
Urm1	Dmbx1				
Cdc73	Phkb				
Hltf	Phf11c				
Dnajc11	Cdc25b				
Gldn	6330419J24Rik				
Adamts19	Ddr1				
Prss38	C030039L03Rik				
Cpox	5330438I03Rik				
Dpy19l1	Carhsp1				
Dgkb	Wnt8b				
Acp1	Kif3c				

miR-5119	miR-671-5p	miR-486-5p	miR-451	miR-122-5p	miR-21-5p
Tmem215	Ccr6				
Zfr	Med13l				
RP23-230O9.7	Wdr48				
Tenm3	Rasal2				
Kcnh7	3110039M20Rik				
Nr6a1	Nsd1				
AA386476	Taok1				
Fam83a	Stat5b				
Sumo2	Efr3b				
Lgalsl	Lrrc8c				
Fgf10	Frmpd4				
Vmn2r30	Galnt10				
Tada2a	Ankrd46				
Ubr4	Klhl3				
Gm12830	Chit1				
Adora2a	Gpr133				
Nsun3	Fbxo32				
Maf	Slc35c1				
Sfrp2	Kctd21				
Dgat2	Syngr3				
Slc31a2	Thumpd2				
Tmem170b	Scn3a				
Sav1	Timp2				
Rbfox1	Gas7				
Eml4	Fbxw11				
Capn2	Magi2				
Gtf2e1	Jmy				
Gtf2ird1	Adipor2				
Pcdh19	Vipr1				
Rps4x	Dag1				
Taf7l	Usp46				
Nudt13	Fbxl7				
Kcnj14	Sox6				
Opa3	Stxbp5l				
Agl	Tnks1bp1				
Gm2042	Lonrf2				
Dach1	Ano6				
Pet2	Sepp1				
Arl5b	Slc4a5				
Spry1	Cux2				
Rtn4	Prom2				
Olfr1034	Pnpo				
Letm2	Ick				
Gm7954	Srpk1				
Farp1	Myrip				
Srpr	Mon1b				

miR-5119	miR-671-5p	miR-486-5p	miR-451	miR-122-5p	miR-21-5p
Ildr2	Ltbp4				
B430203G13Rik	Htt				
Hs6st3	Gm21786				
Gadl1	Sult1d1				
Ino80	Sorcs2				
Rfc2	Zbtb44				
N4bp2l1	Ly6e				
Zfp563	Mgat5b				
Xkr9	Akr1b8				
Cep57l1	Dhx8				
Casc4	Gm10935				
Gm8180	Slc39a6				
Gm28049	Lrrtm2				
Hnf4g	Vat1				
Ubxn7	Pla2g4d				
Sh2d4a	Ccdc85c				
Retsat	Ptprn2				
AF529169	Clcf1				
Zfp740	Il1r1				
Xkr7	Cdc42bpg				
Rp2h	Thsd4				
Tomt	Slit1				
Atp5s	BC021891				
Vash2	Mtf1				
Pnma5	Rragd				
Nudt4	Tyrp1				
Mid2	Prkg1				
Rps6ka2	Tns1				
Rasl11b	Pou2f1				
1500002C15Rik	Fam65a				
Spic	Gbp9				
Gtf2i	Ehd3				
Epb4.1l4b	Btc				
Cldn1	Cckbr				
Usp49	Cux1				
Rint1	Htr1b				
Gm9804	Rab22a				
Atp1a4	Capn8				
Mdga2	1110059G10Rik				
Gm8082	Paics				
Aqp11	Neurl1b				
Bicd1	Gtf2i				
Leprel2	Chmp1a				
Wnt6	Med20				
Ttc9c	Pctp				
Fam177a	Srd5a1				

miR-5119	miR-671-5p	miR-486-5p	miR-451	miR-122-5p	miR-21-5p
Gm8020	Ina				
Skint6	Syt7				
Gm7903	Tbc1d13				
Slc16a5	Fam122b				
Prlr	Dtx1				
St3gal5	St3gal1				
Neurl3	Smurf2				
Mrps10	Myo3b				
Arrdc2	Map3k14				
Sgsh	Gm10382				
Zfp735	6030458C11Rik				
Gm7945	N28178				
Mier1	Fan1				
Btnl9	Zfp81				
Ccdc122	Ube2f				
Nudt21	Hibadh				
Pde1c	B4galt2				
Tbcel	Mob1a				
Slc39a1	Vamp2				
Lbr	Foxp1				
Hipk1	Traf3				
Mavs	Gpr35				
Rad21	Stxbp4				
Galc	Ankfy1				
Gcnt1	Ephb6				
Olfr536	Rgs3				
Zfp605	Tm4sf19				
6430548M08Rik	Dpp6				
Alg10b	Tspyl5				
Syap1	Esyt2				
Pde8b	Traf6				
Flrt3	Tln1				
Wsb1	Wdr7				
Apol7a	Ncs1				
Kcnb2	S1pr2				
Kdm6b	H2-DMb2				
Ubash3b	Mtmr9				
Apobr	Senp1				
Gatad1	Ttc38				
Ssx2ip	Elmod2				
Wipf3	Glis3				
Ccdc25	Sbf1				
Nsun2	Atxn1				
Aco1	Slc26a2				
Evx2	Zfp398				
Gm16506	Evl				

miR-5119	miR-671-5p	miR-486-5p	miR-451	miR-122-5p	miR-21-5p
Rnf182	Acvr2a				
Parp11	Rgn				
Tmem130	Tgm3				
Lgals8	Dcaf8				
Mxd4	Parp1				
Rbm15b	4632415L05Rik				
Dnajb13	Zfp799				
Exph5	Zmat3				
Zfp488	Map1b				
Vmn2r41	Epha3				
Msl3l2	Col17a1				
Gdpd4	Psen1				
Catsperg2	Cep85l				
Taf3	Wipf3				
Osbpl1a	Scyl1				
Zfp811	Tmem236				
St8sia4	Plekhh1				
Gm15821	Ddi2				
Gm17027	Rabgef1				
Gid4	Acot11				
Tnip2	Grm6				
Klf12	4931440P22Rik				
Gpr157	Atf5				
Klhl32	Dpysl3				
Zfp641	Slc6a7				
Snx19	Sppl2c				
Slc20a2	Tmprss13				
Zwint	Otof				
Tnfsf8	BC027231				
Gab2	Pgam5				
Slitrk4	Aqp4				
Galnt4	Mmp24				
C87977	Cwc25				
Fam103a1	Rph3a				
Rps6ka4	Slc25a34				
Pid1	Synpo				
Zfp282	2010111I01Rik				
Ikzf3	Pgm5				
Sema4d	Tmtc1				
Zfp719	Map2				
Nrxn3	Gm6483				
Dmbx1	Steap4				
Cald1	4932411E22Rik				
Fam207a	Lrig2				
Tktl2	Ccdc177				
Zfp36	Phka1				

miR-5119	miR-671-5p	miR-486-5p	miR-451	miR-122-5p	miR-21-5p
Sgk2	Maff				
Atp1b2	Ago2				
Apold1	Lpar5				
BC016579	Slc25a15				
Plau	Kif3b				
Fam89a	1700012A03Rik				
Lamp3	Frmd4a				
Vsx1	Tarbp2				
Creb1	Bdp1				
Zer1	Tmem184a				
Mchr1	Psg20				
Vhl	Nf2				
Scrn1	Zbtb6				
Tsg101	Lmbrd1				
Dsg1a	Lep				
Mrgprb2	Ccdc13				
Rit2	Sec14l1				
Gcm2	Gm12216				
Suv39h1	Arrdc2				
Tubb2b	Capns1				
Mpeg1	Zfp781				
Rlim	Tspan32				
Ticrr	Cgnl1				
Fam107b	Gm3055				
Rab42	Rnf185				
A130010J15Rik	Gpm6b				
Dnaja1	Pmp2				
Fam20c	Podxl				
Nqo2	Slc24a4				
Rybp	Homer2				
Ccdc8	Vldlr				
Map4k3	Tecpr2				
Ogn	Plxna4				
Szt2	Tmem159				
Tmem88b	Ikzf1				
Lhpp	Eno2				
Depdc1a	Far2				
Zfp763	Zfp446				
Ces2g	Asap2				
Sms	Creb3l4				
Osbpl11	Wfs1				
Tacr2	Arhgap12				
Gjb1	Rbms3				
Hist1h2bg	Zscan30				
Fam124b	Ccdc36				
Rbm27	Arhgap19				

miR-5119	miR-671-5p	miR-486-5p	miR-451	miR-122-5p	miR-21-5p
Hdac11	Lemd3				
4930505A04Rik	Wdfy2				
1500011B03Rik	Ap3d1				
Slc27a1	Myrfl				
Uprt	Slc27a1				
Stk36	Syt14				
Parva	Msrb3				
Oip5	Tmem39b				
Dhx40	Rnpep				
AU040320	Zc3h12d				
Notch4	Sash3				
Adamtsl2	Aar2				
Aldh1a3	Adap1				
Klhl40	5430435G22Rik				
Fktn	Tollip				
Zzz3	Mc5r				
Hdhd2	Pxmp4				
Dcp1a	Osbp				
Paqr7	Mip				
Agr3	Ccdc141				
Scyl2	C920021L13Rik				
Tmx4	Gbf1				
Fuom	Oas1d				
Gm10676	Thbs1				
Gm15737	Smad9				
Gm17093	Usp31				
Glrb	Ate1				
Tab2	Plekhg2				
Pfkfb3	Snap23				
Ube2k	Mink1				
Smyd1	Trim17				
Rbm33	Ldoc1l				
Lrat	Rassf6				
Taf1d	Slc22a15				
Pygo1	Trpm3				
Cbwd1	Ptpn6				
Tigd2	Mmp2				
Gpr88	1200014J11Rik				
Dspp	Olfr619				
6030458C11Rik	Hmga1-rs1				
Tmem55a	Fosl2				
Lmcd1	Ly6g6f				
Ahsa2	Large				
Murc	Sstr4				
Itgb6	Cxxc1				
Gm20499	Apba1				

miR-5119	miR-671-5p	miR-486-5p	miR-451	miR-122-5p	miR-21-5p
Erbb2	Olfml2a				
Pik3c2a	Col23a1				
Zfp182	Cd164				
Gm8165	Tpo				
Cntn4	Nova2				
Gm17079	Eps8l1				
Apol10b	Fam204a				
Dmp1	Krtap11-1				
Acvr1c	Ammecr1				
Klf10	Cdkl1				
Tgfbr1	Ranbp6				
Gm17654	Avpr2				
Prps1l3	Zfp334				
Ptgfr	Tenm4				
Hist4h4	Tspan18				
Tnfaip3	Camk2a				
Gm8212	Slc34a2				
Rnf168	Espnl				
RP23-408P8.1	Fam102a				
Apol7c	Stil				
Gm8024	Kdm4b				
Gm17078	Cma1				
Tmprss2	Atg9a				
Mfap1a	Slco1a1				
Crim1	Tex35				
Tbc1d20	Rab28				
Wrn	Eya3				
Stk17b	Rhd				
Gatad2b	Ncor1				
Sgol2	Atp6v1e1				
8430419L09Rik	Cox19				
Ces2a	Cecr2				
Ikzf5	Npr3				
Tmprss11g	Bcas3				
Slc35e1	Sqstm1				
9130019O22Rik	Sema3c				
Chrna2	Crb2				
RP23-180L12.5	Akap8				
Susd1	Wrn				
Wasl	Qprt				
Rab30	Baz2a				
Epn2	Apol6				
Eef2k	Acap2				
Zfp930	Nudt2				
Glra3	Syt9				
Pax9	Runx2				

miR-5119	miR-671-5p	miR-486-5p	miR-451	miR-122-5p	miR-21-5p
Txlna	Bcr				
Dcx	B4galt7				
Gpr89	Ssrp1				
Ubxn2a	Gal3st1				
Bcl9	Mprip				
Spred2	Zfp825				
Lmo3	D030025P21Rik				
Mr1	Pou3f4				
Cux2	Oprm1				
Dtx4	Sugp2				
Irgq	Tmem183a				
Erp29	Glp2r				
Slit2	Lclat1				
Skint4	Lrrc3				
Ctcfl	Prkch				
Chn2	Reg4				
Trabd2b	Usp30				
Ptgs2	Vopp1				
Rassf6	Lrrc59				
Gnptab	Gapvd1				
Rnf149	Gcsam				
Samd12	Dpp9				
Spag17	Gab2				
Cyp2d22	Tlk1				
5730559C18Rik	Mn1				
Gm14434	Acp2				
Pdzd3	Whsc1				
Gm12695	Vip				
Smc3	Sp140				
Sec61a1	Ttyh3				
B230217C12Rik	Sh2d4b				
Yars	Prr5l				
Mgat4a	Kbtbd12				
Rrm2b	Fam134a				
Cabp2	Aldh3b2				
Ptchd4	Asb18				
Prpf38b	Mlxipl				
Morf4l2	H2-M5				
Naip5	Htr4				
Asb17	Fyn				
Rassf8	Vezf1				
Desi2	Kcnk3				
2510003E04Rik	Plch2				
Pclo	Mcemp1				
Gpc5	Casr				
Fam196b	Gemin5				

miR-5119	miR-671-5p	miR-486-5p	miR-451	miR-122-5p	miR-21-5p
Gm4724	Ttc23				
Psmb11	Lims2				
Gm14305	Rassf2				
Nim1k	Hist2h4				
Gm11007	Lrsam1				
Gm2007	Adarb1				
Slco2b1	Ap1s1				
Gabrg3	Gm21698				
Ccdc36	5031410I06Rik				
Orc2	Gm10000				
Eny2	Hoxa6				
Usp43	Gm10471				
Hcls1	Ttll10				
Cenpl	Lzic				
D130043K22Rik	Slc38a2				
Nlgn1	Sh2d5				
Nlrp2	0610030E20Rik				
Enam	Ubp1				
Nabp1	Slc6a9				
Zfp382	Slc25a40				
Tmem126b	Fbn1				
Nck1	Gstm6				
Rnf24	Pear1				
Slc26a9	Gbx1				
Aifm2	1700011L22Rik				
Brcc3	Alkbh5				
Prrg1	Emx2				
Hhat	Npm3				
Gm8232	Tspyl1				
Phf11c	AW549877				
Erg	Scn5a				
Gm6710	Rlf				
Malt1	Sec61a2				
Slc6a1	Wdr43				
Crispld2	Wdr52				
Rragc	Snx29				
Ptp4a2	Sh2d2a				
Mcph1	Galnt4				
Gm3072	Gdf2				
Tceb1	Nos1				
Cyld	Gm7347				
Acsm5	Baalc				
Slc26a7	Hcn1				
Mllt10	Pank3				
Stx16	1700019G17Rik				
Vkorc1l1	Scrt2				

miR-5119	miR-671-5p	miR-486-5p	miR-451	miR-122-5p	miR-21-5p
Cdkn1b	Cst11				
Capza1	Tanc2				
Prss35	Thsd7a				
Slc35b4	Ide				
Tmtc1	Mme				
Lztfl1	Gm10735				
Nr2c1	Fmo9				
Klhl33	Krt15				
Gm6658	St6gal1				
Hecw1	Slc2a2				
Prkx	Sel1l3				
Trim6	Hinfp				
Hnf4a	5031439G07Rik				
Rpgrip1l	Ube2i				
Becn1	Relt				
Pum2	Mylip				
Csdc2	Map2k7				
Snrpd1	Acvrl1				
Mroh6	Fbrs				
Gad1	Mfap3				
Slfn5	Med15				
Tnfsf4	Stag1				
Nhlrc4	Tspan14				
Dmxl2	Gltscr1				
Zfp385b	Hist1h1c				
Slc14a2	Ramp2				
Tmod2	Nckap5l				
Zfp655	Fam135a				
Pask	Llgl1				
Erbb4	Cntn2				
Serf1	Tmem132e				
St6galnac3	Gapdhs				
2410141K09Rik	Zfp182				
Mfap3	Usp53				
Vdr	Car7				
Trnt1	Dlx3				
Mterfd3	Jag1				
Vmn2r57	Pde9a				
Rbx1	Klk14				
Gm7970	Rap2b				
Nhsl2	Zfp275				
Gm7980	Tnrc6c				
Pex5l	Atg13				
Rbm24	Tmem35				
Fkbp9	Tmem72				
Jak2	Oas1e				

miR-5119	miR-671-5p	miR-486-5p	miR-451	miR-122-5p	miR-21-5p
Pdap1	Ptchd4				
Lingo1	Nrp1				
Gabra1	Fam117a				
Dpcr1	Plekha6				
Prkch	Slc16a2				
Spred3	P4htm				
Asb4	Zglp1				
Dcaf12l1	Hpcal1				
Fkrp	Ctdspl				
Setd8	Tmc7				
Chrna4	Dyrk1b				
Trim24	Twf1				
Gm10032	Nefh				
Chid1	Parm1				
Ccny	Secisbp2l				
Tmem260	Cyp21a1				
Rcan3	Srd5a2				
Pex11g	Fam199x				
St18	Chrna3				
Hdgf	Apold1				
Cdk9	D15Ertd621e				
Otog	Pik3c2b				
Slc6a3	Crp				
Uqcc1	Mmp15				
Ism1	Lmbrd2				
Hebp2	Inpp5b				
Tomm40l	Tmem178b				
Sco1	Unc119b				
Pten	Gprin1				
Fgf3	Ubap2				
Eif6	Aacs				
Tgs1	Tomm20				
Pcdh15	Tmem125				
Dsc3	B430305J03Rik				
Xk	Marveld2				
Rpl7	Rhoq				
Car12	Rusc2				
Bace1	Nudt4				
Eif4a2	Plcxd3				
Polr3f	Filip1				
Apon	Ddx39				
A930033H14Rik	Nfrkb				
Atp8b2	Aif1l				
Ghsr	Pitpnm2				
Hrsp12	Zfp212				
Cct6b	Fam132b				

miR-5119	miR-671-5p	miR-486-5p	miR-451	miR-122-5p	miR-21-5p
Hgd	Gm5447				
Lrrc8b	Npy4r				
Ccdc141	Zfp623				
Cdh5	Fut10				
6330419J24Rik	Krt1				
Gm17296	Fbxo21				
Rab14	Tmem150a				
Alcam	Qsox1				
Cntnap5c	Zbtb39				
Syndig1	Rab44				
Gpr174	Clec2l				
Mpp5	P2rx5				
Arhgap6	Bard1				
Cflar	Igsf21				
Coa5	Hist1h4j				
Brd7	Dctn4				
Coro1c	Fam168b				
Gm10615	2310050C09Rik				
Gm17026	B4galt6				
Fjx1	Atp9a				
4930539E08Rik	P2rx6				
Diras1	Stpg1				
Gm17124	Olfr519				
Rnf141	Gzf1				
Rab27b	Vsnl1				
Vcpip1	Usp13				
Inhbe	Vamp1				
Dpt	Pdzk1				
Trim12c	Trpc1				
Lrrc75a	Agps				
Ncoa2	RP23-29K12.9				
BC026585	Cdk5r1				
Carm1	Hcar2				
Tmem125	Crtam				
Syndig1l	Pacsin2				
Dcaf6	Elmo1				
Coq10b	Slx1b				
Lonrf3	Klhdc7a				
Slc24a4	Upk1a				
Tmem106b	Zfp111				
Rho	Mettl7a3				
Fam184b	Mettl7a2				
Clec2e	Mras				
Zfp697	Fancd2os				
Glp2r	Cdkl4				
Nrk	Gm28049				

miR-5119	miR-671-5p	miR-486-5p	miR-451	miR-122-5p	miR-21-5p
Cadps	Nupr1l				
Mef2c	Sec61a1				
Tenm4	Gm10220				
Gdap1	Wdr78				
Epm2aip1	Ifitm10				
Ago3	Uba5				
Rpusd2	Gm1979				
RP23-133G16.2	Klrk1				
Gm6482	Cep170b				
Adamts18	Ppp1r16b				
Six3	Rac2				
Nkx1-2	Trak2				
Cmc1	Scn7a				
Gm7951	Gm9925				
Zdhhc20	C1galt1				
Trip12	Rasl10b				
Gramd1b	Exoc3l4				
Hist1h2bh	Adam23				
Nkapl	Ldlrap1				
Akirin2	Cdk5rap2				
Gpr183	Fech				
Slc25a51	Nxpe2				
Gtf2f2	Scara5				
Gm8229	Tlk2				
Jarid2	Zfp236				
Gpr50	Gkn3				
Rcor3	Dnajb13				
Tnip3	Kcnq2				
Erich1	Arhgef7				
Alkbh8	Crat				
Ttc4	Tmem132c				
Cxx1b	Sfxn5				
Rbm8a	Cachd1				
Piezo2	Adam11				
Mxi1	Sestd1				
Med13l	Chdh				
Anapc16	Ptch1				
Gm17349	Dpp8				
Mkln1	Iqsec1				
Me1	Tmem97				
Tead1	Slamf1				
Abhd2	Hnrnpa1				
Gcsam	Ybey				
Mtif2	Chrnb3				
Lnpep	Adal				
Helb	Necab2				

miR-5119	miR-671-5p	miR-486-5p	miR-451	miR-122-5p	miR-21-5p
Pcf11	Prss8				
Yod1	Ago3				
Ahr	Cnr1				
Pou3f1	Ucn2				
Cxcr2	Exoc3l2				
Frk	Atp6v0a1				
Egr3	Faxc				
Rin2	Lct				
Hist1h2bk	Gfi1				
Ythdf3	Tmem8b				
Rwdd4a	Hmga1				
4932411E22Rik	Gm10681				
Tma16	Hsd3b4				
Itgb3bp	Rgl1				
Zfp442	Tbx15				
Enpp4	Col6a4				
Prdm9	Fbxo40				
Msl2	Gm8923				
Ghdc	Csnk2a2				
Pak7	Npbwr1				
B3galt1	Mdm4				
Plk2	Ptprg				
Nhlh2	Dnajb12				
Fgf6	Pcdhb2				
Anp32e	Zcchc3				
Rab43	Adcy7				
Pigr	Clns1a				
Zfp91	Nckap1				
Alkbh5	Gm5531				
Klrb1f	Pcgf3				
Acp2	Olfr157				
Ggps1	Dnajb14				
Atl3	Krt71				
Slc39a14	Gpr107				
Cdon	Usp28				
Ptpn2	Gm7361				
Ppp4r2	Zfp740				
Lyrm7	Sgpl1				
Zkscan4	Pttg1ip				
Gm9949	Kif5a				
Scyl3	Styx				
Rnf150	Tlr5				
Atp13a3	Alk				
Pdpr	Vwa2				
Gm5127	Gm4450				
Scn9a	Gm14698				

miR-5119	miR-671-5p	miR-486-5p	miR-451	miR-122-5p	miR-21-5p
Pkd1l1	Chil3				
Dio2	Fam185a				
Ddx3x	Fam120c				
Magt1	Ahrr				
Fads2	Tnfaip3				
1810049J17Rik	6430571L13Rik				
Bicc1	Hoxc10				
Papolg	Itih5				
Sypl2	Celf5				
Ltn1	Kcnb2				
Epb4.2	Wwp1				
Csgalnact2	Stxbp2				
Homer1	Abcg2				
Pgr15l	Siglech				
Oas1h	Gstcd				
Scimp	Rbm33				
Rsph3a	Tppp				
Tmem26	Chrnb2				
Hdac4	Chd6				
Ank2	Mepce				
Csgalnact1	Sbk1				
Rps6kb1	Glp1r				
Klf13	P2rx7				
Tmem74b	Il34				
Prox1	Fam180a				
Cttnbp2nl	Fes				
Prc1	Sema3g				
Zbtb20	Ptplad2				
Rdh16	Prss39				
Ptprt	Peg10				
S100pbp	Mup5				
Ngfr	Sptlc1				
Icmt	Peli3				
Serpina3c	Neurod2				
Snapc3	Ern1				
Fzd7	Krt6a				
Rapgef3	Arfgef1				
Cntln	Slc9a3				
Gpr160	Mettl14				
Atrx	Tnpo1				
Ddr2	Sptb				
Osbpl8	Mospd2				
Zfp964	Shroom3				
Otud7b	Rpusd1				
Fgf12	AB041806				
Fam161b	Ubn2				

miR-5119	miR-671-5p	miR-486-5p	miR-451	miR-122-5p	miR-21-5p
Ak4	Pkp3				
Tmem116	D10Bwg1379e				
Lhx4	Taf4b				
Fkbp15	Cacng5				
Rfwd3	Trpv6				
Tnfsf18	Fhit				
Csmd3	Bmf				
Myo5b	Pik3ip1				
Dnajc24	Calb2				
Ubn1	Tmem233				
Gm12216	Sacm1l				
Fcamr	Rab11fip3				
Gng2	Slc35a4				
Vwa5a	Tmem207				
Adipor1	Ppp1r16a				
Eid2b	Sstr3				
Ttpa	Bmp2k				
Trim44	1700012B07Rik				
Mfsd12	Ppm1e				
Nek2	Gm6878				
Pou3f4	Prrc1				
Zfp346	Msi1				
Sptbn1	Gpr157				
Pgpep1	Il33				
Zfml	Ppp1r3f				
Gbp9	Gucd1				
Ago4	Gfpt2				
Ptgfrn	Sdc3				
Socs6	Slc6a2				
Dnajc3	Smim6				
Gm765	Ap1ar				
Guf1	Rbm27				
0610030E20Rik	Lrrn4cl				
Tsr2	Minpp1				
3830406C13Rik	Bmpr1b				
Hykk	Inpp4b				
Tmem231	Scrt1				
Lpar3	Gpr124				
Rtel1	Pde10a				
Pnpla3	Tmie				
Lpar2	Gm9996				
Dsn1	Pim1				
Klf16	Fitm2				
Agtr2	Tceal8				
Npy1r	D10Wsu102e				
9230019H11Rik	Gnb3				

miR-5119	miR-671-5p	miR-486-5p	miR-451	miR-122-5p	miR-21-5p
Mtmr9	Mettl7a1				
Prpf40a	Sez6l				
Hmgb1	Rapgef5				
Pyroxd1	Alg10b				
Selt	Foxd4				
Maml2	Mapk1ip1l				
4933415A04Rik	Pqlc1				
A830018L16Rik	Pxk				
Limk2	Sdf4				
Fam117a	Hcar1				
Foxo3	Gnpda1				
Rbl1	Ikzf5				
C2cd4a	Gng12				
Itga8	Lrtm2				
Actrt3	Tubgcp6				
Rrp36	Chst3				
Gabrb3	Kcnj15				
Gzf1	Fmnl3				
Ucp3	Atxn3				
C77370	Map6d1				
Pcdhb19	2310022A10Rik				
Cxxc4	Cd46				
Aoc3	Sppl3				
Smek2	Sytl2				
Hsf5	Slc7a8				
Wapal	Tmem184b				
Mtfmt	Slco1c1				
Lrrc8c	Xkr7				
Olfr701	Mcidas				
Peg12	Clmp				
Ddx60	Lman2l				
Park2	Nphs2				
Bcap29	Ncmap				
Tnpo1	Chodl				
Pex11a	Slc48a1				
Exoc8	Grp				
Gnai3	Gm12166				
Serinc5	4930453N24Rik				
Sfxn1	Dnah3				
Hepacam	Polr1a				
Prickle2	Lphn1				
FMN1	Sidt1				
Camk1d	Ccdc71l				
Spg7	Zc3h4				
Rorb	Asb11				
Gfi1	Arhgef12				

miR-5119	miR-671-5p	miR-486-5p	miR-451	miR-122-5p	miR-21-5p
Kcnj10	Pard3				
Pitpnm3	Arfip1				
Zufsp	Arhgef37				
Synpr	Ctu1				
Txlng	Gna15				
Lrrn4	Chil4				
Zfp652	Trim46				
Aak1	Fbxl21				
Paox	Gabbr1				
Olfr920	Ctdp1				
A830073O21Rik	Il1rapl1				
E030037K01Rik	Sft2d1				
Gm9905	Ppib				
RP23-113N18.1	Aimp2				
Neu3	Ispd				
Slc23a1	Crocc				
Efcab1	Htra3				
Parpbp	Moxd1				
Capza2	Foxe1				
Nlgn3	Fbxl5				
Hist1h1a	Zcchc8				
Cwf19l1	Snx11				
D930020B18Rik	Sowaha				
Kidins220	Gabrg1				
Antxr2	Micall1				
Swap70	Nde1				
Srr	Gm10742				
Zfp324	Zfp956				
Runx1	Hmbs				
Has3	Otud3				
Pcgf3	F2r				
B3gnt6	Gm17093				
Il1rapl1	Chrng				
Lancl3	Hs2st1				
Gucy1a2	Sipa1l1				
Cacna1e	Ywhaz				
Slc7a14	Hoxb5				
Casp2	Sptan1				
Stom	Mdn1				
Gm14419	Alg9				
Csrnp3	Vmac				
Itpripl2	Mcm8				
Ccdc82	E130309D14Rik				
Gad1-ps	Fbxo30				
Fcrls	9030624G23Rik				
Wnt9b	Trim16				

miR-5119	miR-671-5p	miR-486-5p	miR-451	miR-122-5p	miR-21-5p
Pkd2	Cxcr5				
Dhh	Prss32				
Cadm2	Srgap3				
Ptplad2	Tubb1				
Zfp322a	Kdr				
Zc3h4	Cntln				
Sertad2	1700021K19Rik				
Tmed2	Dok4				
Gm10033	Ppp2r3c				
Cbx5	Rnf14				
Tmed4	Diras2				
Cpd	Tnfaip8l3				
Pura	Dgkk				
Zfand5	Cdk19				
Rbm18	Atp6ap1				
Mmp11	4931406P16Rik				
Zfp825	Gprin3				
Nr3c1	Amotl1				
2610002J02Rik	Tmem239				
Fitm2	Sf1				
Col8a1	Gbp7				
Stard8	Prrx2				
Ubr3	Dctn5				
Iws1	1700123L14Rik				
Cd97	Ddx54				
Prkaa2	Fzd3				
Sft2d2	Rpp25				
Arf6	Rtn4				
Nhs	Tada2b				
Ptprc	Rasgrp1				
Cldn15	Epn2				
Olfr1442	Hic1				
Vldlr	Kat2b				
Calm1	Negr1				
Zfp125	Olfr354				
AW011738	Rrp7a				
Ptprf	Piga				
Gprc5b	Sowahb				
Smim17	Gramd1b				
Rfx2	Paqr9				
Plch2	Upk1b				
Ostc	Nkd2				
Rassf4	A830018L16Rik				
Usp6nl	Rnf149				
Them4	BC051076				
Asb18	Slco2b1				

miR-5119	miR-671-5p	miR-486-5p	miR-451	miR-122-5p	miR-21-5p
Synj2bp	Erc1				
Tceanc2	Nutf2				
Myzap	Fmod				
Fzd1	Abi1				
Pi15	Gen1				
Zfp398	Spock2				
Mcemp1	Gfra4				
Pbx1	Foxm1				
Ntrk3	Iqgap3				
Fmn1	Scarb2				
Cd93	Jph3				
9430015G10Rik	Cnppd1				
Tirap	Yipf6				
Tnfrsf8	Sec14l2				
Hcn1	Gulp1				
2610018G03Rik	Srebf2				
Rasgef1a	Gne				
Gclm	1700016D06Rik				
C5ar1	Fshb				
Sgta	Dnpep				
Aim2	Abhd10				
Tanc2	Dbt				
Rufy2	Ppp4c				
Dusp4	Pgbd5				
Ccr3	Adora1				
Vps35	Itpripl2				
Acpp	Fbxo31				
Zfp882	Scamp2				
Twsg1	Dnaic2				
Camk2d	Ttyh1				
Robo1	G2e3				
Npsr1	Pla2g2e				
Spock1	Tnr				
Cybb	Tmem26				
Zmym2	Dlx5				
Ddx19a	Trmt44				
Mrpl57	S100a14				
Lrrfip2	Syndig1				
Commd6	Itpkb				
Dclre1c	Igf2				
Mbp	Golga7				
Mblac2	Fgd3				
Rab11fip1	Ccdc134				
Ssbp3	Nup210				
Pdgfd	Olfr1414				
Mturn	Gabpb2				

miR-5119	miR-671-5p	miR-486-5p	miR-451	miR-122-5p	miR-21-5p
Cdnf	Cyp2d37-ps				
Sesn3	Rnf125				
Bmp3	Fam207a				
Mtfr1l	Esp3				
Gucy2e	Arhgap35				
Gm16485	Foxo1				
Srp54a	Ogt				
Als2cl	Pcyt1b				
Ptar1	Srrm4				
Ralgps2	Epn1				
Gbgt1	Yars				
Flrt1	Det1				
Bmpr1a	Axl				
Zfand4	Btf3l4				
Emb	Tbccd1				
Jam2	Mpeg1				
Cers4	Sbk3				
Pex1	Wdhd1				
Slc6a11	Pofut1				
Wbp4	Ubn1				
Musk	Prkcsh				
Kif1b	Tm7sf2				
Phactr2	Ssr1				
Zc3h15	Thbs2				
Ace2	Myh14				
Ccna2	Chst12				
Smg7	Ogdh				
Rhof	Arl4c				
Dixdc1	Ppfia1				
Hsbp1	Zfp300				
Srek1ip1	Rnf220				
Parp8	Cbx6				
Txnl4a	Tal1				
Prkce	Ino80c				
4921528I07Rik	Xlr4c				
Triqk	Vwa5a				
Slc1a7	Atf3				
Vopp1	Stard13				
2610002M06Rik	Dock5				
Ptafr	E430018J23Rik				
Insr	Trim33				
Dll1	Fstl4				
Tapt1	Zcchc6				
Slc7a5	Yap1				
Saa4	Slc4a8				
Slmap	Fblim1				

miR-5119	miR-671-5p	miR-486-5p	miR-451	miR-122-5p	miR-21-5p
Xndc1	Syngr1				
Impa2	Zfp386				
Iyd	Ttc19				
Nacc1	Slc22a26				
Fam149b	Lyplal1				
Prrx2	Ptchd2				
Dppa2	Ppme1				
Tmem87b	Etv5				
Tulp4	Runx1				
Tll1	Wfdc5				
Trim56	Arhgef17				
Gm5431	Tiparp				
Stard5	Ptpro				
Akr1e1	Nek7				
Paqr8	Zswim5				
Gda	Slc5a7				
Zfp84	Otud4				
C530008M17Rik	Nfic				
Ubxn8	Dcakd				
Adam12	Traf3ip1				
Btbd11	Slc5a5				
Lins	Arap2				
Nras	1500009L16Rik				
Cd8a	Nox1				
Eif4ebp2	Cdip1				
Fbrs	Cyp4f17				
Ankrd17	C1s2				
Zfp111	Gm20431				
Zdhhc17	Megf9				
Tank	Traf3ip2				
Gm13011	Pank1				
Whsc1l1	Gm21743				
Dmrta1	Suv420h2				
Spty2d1	Calcrl				
Glra2	Specc1l				
Arhgap5	Plekha8				
Asun	E130309F12Rik				
Cdh1	Aldh5a1				
Btbd1	Dsc3				
Slc17a6	Rcl1				
Wwc2	Dstn				
Prr5l	Mansc1				
Pde7b	Strip1				
Plxna2	Spata2				
Vgll3	Slc37a2				
Hn1l	Pcnt				

miR-5119	miR-671-5p	miR-486-5p	miR-451	miR-122-5p	miR-21-5p
Acot6	Nav1				
Mrpl11	Lin9				
Fign	Myadm				
Eef1a1	Tcta				
Epb4.1	Pqlc2				
Mxd3	Avil				
Vstm2b	Mbl1				
Mbd5	Cabp2				
Wnt2	Rft1				
Pde11a	Ccr9				
Actr10	Phactr4				
Nek10	Nedd4				
Rxfp1	Pramel7				
Tmppe	Nradd				
Nhlrc2	Gm12888				
Astn1	Per1				
Lrrc15	Masp1				
Grm5	Lrp3				
Fv1	Pdk3				
Adarb2	Tvp23a				
Plcd3	Ggt7				
Slc15a4	Cyb5r2				
Asxl3	Piwil2				
Greb1l	H2bfm				
Grhl2	Ubxn2a				
Zfp516	Plekhh2				
Stx6	Cngb3				
Zc3h7b	Prss29				
Lpp	Cnnm3				
AW554918	Tmed8				
Hmgcll1	Arrdc4				
Ckap4	Speer4b				
Gm15800	Olfr70				
Btbd9	Usp24				
Dlgap5	Klhl36				
Stau2	Naa40				
Rsg1	BC053393				
Camta1	Clec9a				
Prrg3	Rab40c				
Slc1a2	C1s1				
Unc13a	Rfwd3				
Ccser2	Anks4b				
Rnf152	Zfp935				
Rtp4	Rela				
Stox2	Zkscan3				
Xbp1	Khnyn				

miR-5119	miR-671-5p	miR-486-5p	miR-451	miR-122-5p	miR-21-5p
Pabpn1l	Syt6				
Mfsd11	Leprotl1				
Nr2c2	Egr3				
Deptor	Arid2				
Hook3	Defb1				
Ptk6	Arc				
Dgcr8	Vcl				
Appl1	Prss55				
Ccdc85c	Prc1				
Kcnd1	L1cam				
5830411N06Rik	Il13ra1				
Mxd1	Psme3				
Cblb	Slc4a9				
Tet2	Git2				
Cpne5	Akap5				
Aldh4a1	Il1rap				
Tardbp	Scamp5				
Fbxo32	Pirt				
Sepsecs	Sema7a				
Midn	Zfp518b				
Alox12	Hars2				
9130008F23Rik	Slc30a1				
Fbln7	Upf2				
Rbm4	6430573F11Rik				
Fam173b	Tmem144				
Acadsb	Mapre2				
Polr3g	Ednra				
Sox6	Gm10300				
Pus7	Pbx1				
Fam208a	Dusp4				
Slc26a2	Lpcat2b				
Plekhh2	Ddx24				
Abra	Lpar1				
Lcorl	Mex3d				
Rfx3	Mbd5				
Zfp869	Nek2				
Gmeb1	Clec11a				
Tmc6	Plxdc2				
Chl1	Dstyk				
Fam129a	Apba2				
Ttll5	Sgpp2				
Atp8b4	Btbd16				
Fam199x	Slc25a11				
Adamts15	Chst4				
Exoc6b	Il10ra				
Hrk	Adra2b				

miR-5119	miR-671-5p	miR-486-5p	miR-451	miR-122-5p	miR-21-5p
Gpkow	Tmc2				
Armc2	Gata5				
Zbtb44	Ube2q1				
4933426M11Rik	Casc4				
Ppargc1b	Ddhd1				
S100a7a	Kctd4				
Kdm7a	Abr				
Cep104	Hif1an				
Ei24	Lrrc20				
Tmem132b	Hecw1				
Tmem184b	Tsen2				
Robo4	Gm9938				
Fhl5	Ptpn2				
Plcxd3	Gm5862				
Haus6	Gtf2h3				
Pou4f1	Abcg1				
Dcaf7	Dapk3				
Plat	Hoxb9				
Pkp4	Zfp169				
Ccr10	Rrn3				
Fam115c	Acsl4				
B230219D22Rik	Hey2				
Wac	Lrrc39				
Rnf11	Mylk2				
Ppp4r4	Tnfrsf22				
Prkg1	Dbn1				
Fndc3b	Hdac4				
Arl5a	Trex2				
Mc5r	Clcn1				
Lnp	Ccdc85a				
BC106179	1810009A15Rik				
4931428F04Rik	Zfp459				
Dhx36	Wbp2				
Slit3	Pde4d				
Ndufab1	Gltscr1l				
Wdr13	P2rx1				
Gm7461	Gpr45				
Sytl5	Pnkd				
U2af1	Gbp6				
Stk32a	Ppm1h				
Zfp148	Drp2				
Gm5434	Lrig3				
Kcnc1	Mmp20				
Cs	Tmem119				
Hdac9	Dpf2				
Syt1	Serpina3n				

miR-5119	miR-671-5p	miR-486-5p	miR-451	miR-122-5p	miR-21-5p
Fut11	B3gat2				
Dock2	Smek2				
Xirp1	Gpt2				
Fam122b	Ercc4				
C3ar1	Cog8				
Chordc1	Myh9				
Snapc1	3110062M04Rik				
Pycard	Psg21				
Antxr1	Myo1c				
Cfhr2	Trim65				
Zfp958	Cxcl11				
Tmem220	Hbs1l				
St8sia3	Exoc4				
D10Bwg1379e	Lrp6				
Rfx7	Tesk1				
Muc19	Pi4kb				
Zswim1	Il18rap				
Ptgdr	Fa2h				
Med6	Diexf				
Prkg2	Slco2a1				
Itga10	Cdc42ep4				
Slc20a1	Setd8				
Tcp11l1	Relb				
Gpd2	Iqcd				
Sc5d	Inhbe				
Gripap1	Zfp595				
C1qtnf7	Tbck				
Lcor	Slc44a1				
Krtap6-5	Slc46a1				
Tnr	Glipr2				
Ptprg	1810065E05Rik				
Tomm20	Gipr				
Mrgprx2	Zfp180				
Mfsd5	Fam71f2				
Slc4a9	6330409D20Rik				
Prickle1	Ckap4				
Sec14l3	Smim14				
Extl1	Mob3b				
Drp2	Kctd10				
Klrk1	Panx1				
Ascc2	Ogfod1				
Eif2ak2	Tarsl2				
1700048O20Rik	Myo9a				
Mib1	Pid1				
Zfp53	Txndc5				
Rasgrp3	Pcid2				

miR-5119	miR-671-5p	miR-486-5p	miR-451	miR-122-5p	miR-21-5p
Zbtb25	Galnt13				
Parp14	Wars2				
Bmper	Slc38a9				
Fcrl1	Efcab6				
Thnsl1	Prkaa2				
Lamtor3	Atox1				
Cbln1	2610002M06Rik				
Pds5b	Gm9804				
Pycr1	Apobec2				
Gm14569	Man2a2				
BC027072	Flrt2				
Adra1a	Numbl				
Slc9a7	Gcnt2				
Mrvi1	Kcnj16				
Uba6	Wnt9a				
Fibin	Pvrl4				
Tmeff2	Onecut2				
Rnf144a	Hoxb1				
Adam23	Cdc37				
3425401B19Rik	Rasa4				
Hipk2	Flrt1				
Purb	Mars2				
Gm10447	Cnot6l				
Fam160a2	Mrvi1				
Ifi203	Gpr89				
Gltpd2	Ttc22				
Ptpro	Zfp592				
Tbx18	Zhx3				
Emp2	Nlrp4b				
Fbxl2	Rdh7				
Zkscan1	A830073O21Rik				
Slc23a2	Nrg1				
Cyp7b1	Ccnb1ip1				
Clcn5	Epha6				
Chtf8	Rnf217				
Olfml2a	Colgalt1				
Zbtb26	Got2				
Sv2c	Zfp28				
Usp12	Fmo5				
Gtpbp8	Pyroxd2				
Cdc37	Nat8l				
Cyp4f39	Lepr				
Kat6b	Oas1c				
Fndc1	Nrxn2				
Gm7008	AU018091				
D3Ertd254e	Cacnb1				

miR-5119	miR-671-5p	miR-486-5p	miR-451	miR-122-5p	miR-21-5p
Klhl8	G6pc				
Pde3a	Gspt1				
Zfx	Prpf4				
Fam92a	Acnat1				
Pank3	Bicd2				
Ksr2	Crip3				
Oprk1	Dact3				
Apol8	Rnft2				
Lyg2	1700055N04Rik				
Fut9	Extl1				
Foxi2	Nrsn2				
Bean1	Cenpo				
Cped1	Dgki				
Lman1	Arl10				
Mecp2	Prkce				
Bnc1	Fam58b				
Ano5	Cdc5l				
Fyttd1	Ncaph				
Setdb2	Kcnk12				
Bbox1	B020004J07Rik				
Taok1	Ube2v1				
Rpl24	Plxna2				
Golph3	D1Ertd622e				
Lpcat1	Gid8				
Arih1	Bcl9l				
Fam160b1	Wee2				
Ammecr1	AI837181				
Sh3rf2	Tfb2m				
Blnk	Adamtsl2				
Mettl10	Clp1				
Vsig2	Gm10032				
Gtf3c3	Aldh1a2				
Zdhhc9	Hykk				
Cr2	Serpina3i				
1110032F04Rik	Gm1818				
Nt5dc3	Fam110b				
Prokr1	Lats1				
Mroh5	Mypop				
Manba	Nppa				
Tfr2	Agfg2				
CTNND1	Zfp709				
Ctnnd1	Nucb1				
Gm14124	Cd99l2				
Ednra	Tra2a				
Impg2	Pla2g15				
Msantd3	Socs5				

miR-5119	miR-671-5p	miR-486-5p	miR-451	miR-122-5p	miR-21-5p
Esyt2	Gcc1				
Gpatch11	Arf5				
Sbk1	Cnnm2				
Rab3c	Adamts12				
Phf8	Cyp4a12b				
Snrnp48	Fam57b				
Trhde	Scn3b				
Anxa13	Nr4a3				
Irf2bp1	Faah				
Bsn	Med13				
Akr1c12	Igfbp2				
Syne3	Zfp882				
Kcnv1	Cblb				
Taf13	Man2c1				
Ypel1	C77080				
G6b	Stat6				
Mtpn	Brd7				
Rnf20	Fam107a				
Gm4631	Heatr3				
Ppfia2	Dpysl2				
Fam219b	Ntrk2				
Rap1gap2	Igf1				
Gpr133	Usp8				
Cxcr5	Ghitm				
Sh2d5	Sstr5				
Pcdh20	Heatr6				
Shisa7	Trim41				
Prkcg	Arhgap44				
Gm14322	Gypa				
Usp29	Mitf				
Gm16515	Ascl1				
Glcci1	Gm16314				
Sdk2	Cyp4a12a				
Ankrd52	Ryk				
Tbck	Brd4				
Pim2	Vmn1r180				
Gm14308	Gab3				
Rab27a	Cnga4				
Gm14326	Adamts3				
Tspan7	Foxi2				
Gpr101	F2rl3				
2210418O10Rik	Cap1				
Orai2	Fgd6				
Skil	Otop3				
Grip1	Tmem231				
Phf20	Tbc1d14				

miR-5119	miR-671-5p	miR-486-5p	miR-451	miR-122-5p	miR-21-5p
Dnm3	Vapb				
Rgs9bp	Fam84b				
Rbpj	Gm14214				
Frmpd4	Hspa4l				
Rhpn2	Fancl				
Zbtb11	Tnfaip2				
Gad2	Bcap31				
Cacng4	Agl				
Ttf1	Pikfyve				
Oas3	Fate1				
Irs4	Zfp354b				
Gm14410	Lrrc52				
Rimkla	Peg12				
Gpr179	Evx1				
Trim33	Zrsr1				
Il33	Zfp629				
Tacr3	Morc2a				
Alx4	Fam49a				
Scn2a1	Ucn3				
Gpr155	Mat1a				
Dnah5	Dmgdh				
Gucy2f	Enpp1				
Bend3	Gpr88				
Cntn3	Btbd19				
Kcnq2	Klhdc8a				
Ncoa1	Marc2				
Tbc1d8b	Zc3h14				
Fbxl18	Gpr137				
Sprr2a2	Cpa5				
Cemip	Dsg1c				
Enc1	C030017K20Rik				
Ppfibp1	Paox				
Ncoa3	Adh6b				
Foxn3	BC068157				
Gm14391	Cdyl2				
L3mbtl4	Edem3				
Fam189a1	Sash1				
Tenm1	Hip1				
Pnma2	Ctnnbip1				
Grin3a	Cbx2				
Gm14295	Rlim				
Ccdc170	Zswim1				
Unc5b	Zdhhc24				
Rasgef1c	Klhl13				
Sele	Chrna4				
Atp10b	Sri				

miR-5119	miR-671-5p	miR-486-5p	miR-451	miR-122-5p	miR-21-5p
Gm14296	Ptplad1				
Mlxip	Dtwd2				
Trub2	Ppp1r13b				
Trrap	Naa30				
Ppp1r3a	F13a1				
Sertm1	Fign				
Zfp651	Ccnd2				
Csf2rb	Slc29a3				
Zfp654	Onecut1				
Shank3	Tnip1				
Srpk3	Mical3				
Kcna6	Ambn				
Sprr2a1	Atg4b				
Pip4k2b	2510049J12Rik				
Cntnap5a	Myt1				
Mief1	Ist1				
Alk	Zfp869				
Htr4	Gpkow				
Gli3	Zkscan1				
Zmynd19	C330021F23Rik				
Cry2	BC107364				
5730507C01Rik	Mgrn1				
Cdc42bpb	Spry1				
Ppm1f	Fzd7				
Dazl	Rce1				
Specc1	H2-Q6				
Pgbd5	Dpf3				
Kcnk5	Zbtb42				
Cngb1	Trabd				
Tbc1d16	Dtd2				
Igfn1	Wrb				
Srsf12	Nlrp1a				
Speg	Aldh3a2				
Gm10083	Kcnj5				
Rag1	Psg17				
Gm14443	Mon1a				
1700024P16Rik	Itgb1bp1				
Pik3r6	Bmp3				
Amot	Cldn9				
Prdm11	Tmem130				
Adamts9	Cenpm				
Gm14418	Prdm13				
Gm14412	Gabarapl1				
Pxdn	Qpct				
AI606181	Ctla2a				
Trpv2	AK157302				

miR-5119	miR-671-5p	miR-486-5p	miR-451	miR-122-5p	miR-21-5p
Erbb3	Wdr91				
Dcaf17	Thoc3				
Adamts1	Senp2				
Ash1l	Shkbp1				
Pea15a	Mrgprf				
Slc25a16	Kctd18				
Rprd1b	Qser1				
Ptch1	Eif4ebp3				
Odf2	Gna14				
Mcu	Canx				
Fam73a	Tcp10c				
Decr1	Lgi1				
Numb	Sh3gl2				
Pxn	Cdc25a				
Arel1	Rasa2				
Arrb1	Gsk3b				
Slc6a6	Eef1a2				
Ermp1	Adk				
Kif5b	Senp5				
Aldh1l2	Plekha2				
Adat3	Hpse2				
Homer2	Trim50				
Lgr6	Rab30				
Vps39	Smg9				
Sdc3	Napepld				
Kitl	Sema4d				
Atf6	Mthfsd				
Mill2	Zfp947				
Trp53inp1	Dgcr2				
Cdyl2	Mrgprd				
Vps13c	Dlg1				
Unc119b	Rfx5				
Itga3	Vmn1r25				
Rcor1	Cacnb3				
Zkscan8	Pgk1				
Pogk	Plekhb1				
Ndst1	Asb8				
Tmem170	Il5				
Gas7	Odf3l1				
Ppara	Il20ra				
Tgfbr3	Pms2				
Uap1	Zdhhc16				
Man2a2	Actr2				
Ddx6	Lrrc49				
Ern1	Mrrf				
Adra2b	2210408I21Rik				

miR-5119	miR-671-5p	miR-486-5p	miR-451	miR-122-5p	miR-21-5p
Nploc4	Malt1				
Gid8	Fnbp1l				
Gtf2h5	Hp1bp3				
Mis12	Nras				
Abca6	Mlst8				
Necab1	Luc7l2				
Dip2a	Slc12a5				
Slc35d1	Ctsa				
Prkcb	Cnbp				
Cgnl1	Hrk				
Abcg2	Gdpd4				
Inpp4b	Sssca1				
Hhipl1	Lhfpl5				
Birc3	Hrct1				
Kras	Mc1r				
Lingo2	Thada				
Fam171b	Cx3cl1				
Ccin	Ptx4				
Eif4h	Nr6a1				
Pacs2	Fam84a				
Map2k4	Nr3c1				
Il6st	Mapk10				
Tmem185b	1700037H04Rik				
Peli2	Etnk1				
Ttl	Abca6				
Slc26a1	Zfp831				
Pofut1	BC021785				
Adal	Dync2li1				
Rpl22	Elac1				
Trove2	Eya2				
Zfp445	Yod1				
Zfp207	Ralgapb				
Pabpc4l	Dock9				
Cd99l2	Fam196b				
Pmpca	Cd84				
Nr4a2	Aanat				
Tmem251	Cul3				
Zkscan7	Cc2d1b				
Ncald	Sema3e				
Zfp446	Acsbg1				
Loxl2	Pramel1				
Lin54	Iqce				
Eea1	Hist1h3d				
Lactbl1	Mphosph9				
Rbfox3	Ap3m1				
Peg3	Htr1a				

miR-5119	miR-671-5p	miR-486-5p	miR-451	miR-122-5p	miR-21-5p
Mmp16	Chn2				
Epha3	Mab21l2				
Scube3	Cd28				
Lsamp	Klhl5				
Lactb2	Dpm2				
P2rx7	Kcnj12				
Piwil2	Klkb1				
Ppp1r26	Snx19				
Rps6	Il17rb				
Mga	Isca1				
Mmachc	Gpatch2				
Stc2	Pif1				
Adamts2	Rai1				
Fam126b	St6galnac4				
Rnls	Akt2				
Prrg4	Ero1lb				
Rc3h1	Pigv				
Tmem245	Col6a2				
Slc38a2	Mamstr				
Spopl	Adnp2				
Dpep2	Nfkbib				
Ano3	Smad3				
Braf	Ccdc129				
Pgap1	Runx3				
Hs3st1	Ap1m2				
Srsf3	Cxcl9				
F2rl3	Sgk2				
Igfbpl1	Brca1				
Hsf2bp	Fktn				
Zfp354a	Chst11				
Acn9	Sod3				
Spc25	Slamf6				
Tram2	Irak3				
Cd46	Fam122c				
Elmsan1	Ttbk1				
Rora	Glcci1				
Rap1a	Thoc7				
Tmx3	Gm5900				
Zbtb41	Rfx1				
Foxm1	Spag17				
Pign	Cog6				
Setd7	Pde5a				
Rcan2	Mrps18a				
Ino80d	Gpr125				
Gab3	Fkbp1a				
Rbm34	Gm12353				

miR-5119	miR-671-5p	miR-486-5p	miR-451	miR-122-5p	miR-21-5p
Fmo2	Eme2				
Zbtb1	Tmco3				
1810013L24Rik	Rasef				
4930579G24Rik	Mx1				
Dao	Knop1				
Stat1	Fahd1				
Cmpk2	Ctbp2				
Fkbp5	Gm4861				
Ascc1	Pom121				
Pak3	Prdm10				
Car13	Zfyve28				
Sox11	Tmem179				
Cyp51	Hoxa10				
Naa50	Rad54l2				
Ggcx	Aph1a				
Hddc3	A930016O22Rik				
Gm12185	Akap6				
Slc22a14	Ensa				
Dnajc18	Pdgfrl				
Dgke	Klf16				
Ttll1	Fbxo41				
Ears2	Pign				
Uox	Whamm				
Noxred1	Doc2b				
Sycp2	Rabep2				
Shmt1	Col26a1				
Tmem154	Slc23a2				
Vezf1	Engase				
Acacb	Phc2				
Ttc39b	St6galnac6				
Zranb1	Cyp4f16				
Prkrip1	Cebpzos				
Cog8	Lipn				
Btbd19	Dedd				
Zdhhc15	Stau1				
Chd3	Numb				
Sap30bp	Ccdc114				
1700102P08Rik	Chd1				
Cmtm6	Loh12cr1				
Ubox5	Nipa2				
Akap11	Srrm1				
Asxl1	Itgb2				
Akap17b	Cntnap5c				
Cycs	Iglon5				
Cacna1g	Cyp2u1				
Sh3glb1	Gast				

miR-5119	miR-671-5p	miR-486-5p	miR-451	miR-122-5p	miR-21-5p
Fads6	Ap3s2				
Sbno1	Fzd4				
Wdr59	1810041L15Rik				
Gatsl2	Meaf6				
Arhgap20	Slc19a2				
Myoz1	Def8				
Pcdhb2	Epx				
Chrna1	Samd11				
Ankrd13c	Ptprd				
Ndc1	Hs3st3b1				
Abi2	Kctd15				
Bcl9l	Tram2				
Grk5	Cpd				
Zc3hav1l	Rcan2				
Rragd	Erbb3				
Zfp292	Fndc1				
Gtf3c6	Trip10				
Doc2b	Plxdc1				
Gpr1	Brf1				
Slc25a43	Iqca				
Arf3	Fbxl12				
POU2F1	Dse				
Arhgap12	Meox2				
1110002E22Rik	Krt83				
Klhl5	Trp53i11				
1190002N15Rik	Osbpl1a				
Dram2	Ralgapa1				
Gca	Pcdh1				
2310005G13Rik	Serpina9				
Rreb1	Ndfip1				
Gm11437	Stat5a				
Nsa2	Ttc14				
Capn5	Nanos1				
B430306N03Rik	9330159N05Rik				
Cyp2j9	Igf2r				
Mtr	F930015N05Rik				
Tmem135	Xylt1				
Dennd5b	Ell				
Fbxl20	Art4				
Pcgf5	Kpna3				
Aebp2	Gm10309				
Dnajc13	Slc1a7				
Mrpl51	Lamtor3				
Jph4	Zfp964				
Zfp316	Bnip1				
Memo1	Lmo2				

miR-5119	miR-671-5p	miR-486-5p	miR-451	miR-122-5p	miR-21-5p
Twf1	Spryd4				
Senp5	1810026J23Rik				
Il15	BC026585				
Tspan8	Eno4				
Pigp	Hoxc8				
Crtap	Riok1				
Ston2	Mapk14				
Ccdc115	Xcr1				
Kcnmb4	Dusp28				
Zfp422	Lipe				
Sos1	Fgf3				
Atp6v1d	Eif2ak1				
Slc16a7	Zpld1				
Zfp106	Rad50				
Nadk2	Cdk20				
Adnp2	Fam13a				
Sec61g	G3bp1				
Aqp2	Tgfbr2				
Hdx	Mapk12				
Lims1	Spryd3				
Naa25	2010107G23Rik				
Kpna4	Crispld2				
Papln	Prdm16				
Foxp1	Krt4				
0610031J06Rik	Oca2				
Tmem178b	Arhgap17				
Mpp6	Fxyd6				
Mfhas1	Tfap2a				
Tmem178	Bub3				
Gm13151	Btn1a1				
Tert	Cnnm1				
Angptl4	Ears2				
Gdpgp1	Nsf				
Pik3r1	Pnpla1				
Bcl7c	Cdk2				
Nova1	Zfp787				
Apex2	Cbx4				
Slco1a1	Lrrn1				
Mme	Kremen1				
Mettl2	D3Ertd254e				
Dnajb14	Astn2				
Gprin3	Rpl22				
Cacul1	4930519P11Rik				
Csrnp2	Chst14				
Lrrc4c	Klrg1				
Zfp65	Shisa5				

miR-5119	miR-671-5p	miR-486-5p	miR-451	miR-122-5p	miR-21-5p
Sh3d19	Rnf224				
Gm3629	Tusc2				
2610001J05Rik	Dlgap4				
Errfi1	Sema4g				
Adam22	St14				
Sphkap	Mecom				
Tmem110	Usp42				
Trim5	Dock6				
Btg1	Limch1				
Sh2b1	Gm11437				
Cnot6	Ccl19				
Snrnp27	Prss23				
Alox5ap	Pcdh15				
Zfp462	Magee1				
Aptx	Gpr17				
Ap3s2	Ptgfr				
Hax1	Slc30a4				
Tfrc	Krba1				
Agap1	Ncf1				
Dhtkd1	Ptrf				
Yy1	Pbx2				
Rab8b	Rnf114				
Ppm1a	Adra1a				
Usp30	Grin2a				
Zfp597	Prrxl1				
Sp4	Pnpla3				
Ubald1	Galnt6				
Map3k9	Ncan				
Opa1	Disc1				
Ddx10	Lman2				
Map2k2	Nfatc1				
Ppp1r9a	Gns				
Gucy2g	4933427I04Rik				
Fam69a	Gm16286				
Nmnat2	Srxn1				
Stk35	Wt1				
Rnf217	Apobec3				
Pqlc2	Foxl1				
Chrm1	Endov				
Elovl7	Gm5111				
Zfp526	Atp5s				
Diap2	Ubash3b				
Arhgef12	Hist4h4				
Otud4	Aph1b				
Stxbp5	Slc13a3				
Coa4	Etv3				

miR-5119	miR-671-5p	miR-486-5p	miR-451	miR-122-5p	miR-21-5p
Clcn6	Ikbke				
Tango6	Socs7				
Slc17a3	Bloc1s1				
Rin3	Manea				
Zfp113	Itk				
Tmem44	6530409C15Rik				
Gm3739	Bche				
Eif2ak1	Txlng				
Hist1h2ac	Abt1				
Cacna1a	Gm10308				
Akap8	Slc9a9				
Cetn2	Pik3cb				
Lrrc38	Gm12887				
Kctd16	Htr5a				
Zfp81	Ank3				
Mctp1	Cdc73				
9530068E07Rik	Il20rb				
Ptger1	Gpalpp1				
BC067074	Ulbp1				
Hsd17b6	Il1rn				
Sh2d4b	Bahd1				
Sult1d1	Lmtk3				
Pigm	Eif6				
Tatdn3	Elmsan1				
Agpat6	Usp6nl				
Exoc3	Tbc1d2b				
Hist1h4k	Rbfox2				
A530054K11Rik	Fam89b				
Pcdh7	Cables2				
Bard1	Prkag3				
Cplx2	Cryba1				
Zscan25	Zdhhc7				
Vdac2	Exoc3				
Rapgefl1	Serpina3b				
Ppp1r12a	Fcgr2b				
Etf1	Cwf19l2				
Fzd9	Cobll1				
Prkd3	Sik2				
Pgk1	BC030500				
Zc2hc1c	St8sia2				
Ophn1	Gimap4				
Gm17641	Cd300lb				
Zfp39	Ankrd63				
Relb	Asphd2				
Slc30a6	Cep41				
Zfp85	AA986860				

miR-5119	miR-671-5p	miR-486-5p	miR-451	miR-122-5p	miR-21-5p
Thsd1	Prpf18				
Trim16	Slc30a7				
Pcdhb14	Pdik1l				
Pdk3	Plcl1				
Cpsf2	1810055G02Rik				
Ccdc88a	Rab36				
Snupn	Prrx1				
Ube2e3	Maob				
Strbp	Myf5				
Smim20	Kcnk2				
Mrgprh	Ankef1				
Usp15	Rac3				
Pou5f1	Gpr20				
Irak4	Dpysl4				
Arhgap32	Wnt4				
Srp54b	Trim60				
Atp8a1	Siglec1				
B3galt5	Cuedc2				
Ccdc177	Abhd16b				
Plxna1	Edem1				
Smug1	Barx2				
Peg10	Col4a4				
Wnt2b	A330070K13Rik				
Tbc1d14	Kcnq1				
Kctd15	Ophn1				
Cd74	Arel1				
Sp6	Slc35a3				
Scrt1	Rptor				
Adamts3	G6b				
Zdbf2	Casp16				
Tcam1	Adcy5				
Kif5a	Nckipsd				
Pcdh11x	Rab15				
Sfmbt2	Aass				
Chml	Tspan9				
Cramp1l	Htr2c				
Hlcs	Dnajc5				
Avpr1b	Rufy2				
4930402H24Rik	Ampd3				
Csnk1e	Padi4				
Gm6086	Tcp10b				
Zcchc2	Cd6				
Slain1	Smarcal1				
Dgkd	Ak1				
Onecut3	Syt1				
Tspyl5	Nhlrc2				

miR-5119	miR-671-5p	miR-486-5p	miR-451	miR-122-5p	miR-21-5p
Flywch1	Mnt				
Larp4	Slc6a8				
Lrrtm2	Dlg4				
Leprel1	Glrx3				
Thoc2	Kdsr				
Nr4a3	Stard5				
Ano6	Ifi44l				
Chrnb2	Fxyd4				
Kmt2a	Casc3				
Plxna4	Pitpnc1				
Tollip	Prpf38b				
Ldoc1l	Emp2				
Creb3l2	Mfap5				
Cnga3	Morn4				
Ccser1	Enpp6				
Gm6970	Hcn4				
Pcdha10	Aktip				
Numa1	Igf2bp1				
Thsd7a	Fam131b				
Podxl	Tcea1				
Rfng	Ptafr				
Rnf139	N4bp2				
Smarca5	Spink2				
Prkacb	Gpr1				
AU022252	Tmem38b				
Nfib	Usp26				
Dpysl5	Aatk				
Itgav	Swt1				
Zfyve20	Taf1d				
Plek	Xylt2				
Zak	Tmem74b				
Acyp2	Tusc5				
Ppp2r3c	Pitx3				
G2e3	Fem1c				
Kif5c	Cyp1b1				
Lmbrd2	Sh3bgrl				
Ahcyl2	Mkln1				
Ebf3	Mex3b				
Ylpm1	Hist2h3c1				
Glis3	Rad51ap1				
Kif1a	Gnb5				
Samd4	Loxl4				
Abcb7	B3galt1				
Rxra	Hk1				
Snrpb2	Heph				
Kifap3	Tsc22d2				

miR-5119	miR-671-5p	miR-486-5p	miR-451	miR-122-5p	miR-21-5p
Plekhh1	Rpl38				
Bbx	Ppp1r8				
Rbms1	Rab2b				
Socs7	Gmppa				
Rnf111	Hdac3				
Macrod2	Paqr8				
Vps26b	Foxa1				
Asap1	Arhgdia				
Kdm5a	Sit1				
Gla	Syngr4				
Zfp553	2410131K14Rik				
Olfr39	Rbm4				
Usp2	Xirp2				
Slc45a4	Trim35				
Ptpn4	Nlgn1				
Sf3a1	Nxf1				
Paqr5	Gtf3c3				
Alg14	5830473C10Rik				
Wdr26	Llgl2				
Pou2f1	Mllt11				
Igf1r	Npc2				
Klf7	Cd93				
Avl9	Kcnd3				
Smad9	Kcna7				
Phip	Fam188b				
AA415398	Nov				
Cp	Ddx51				
Erlin2	Rsf1				
Arhgap26	Lsm11				
Actr8	Morc4				
Dcxr	Hoxd3				
Trim2	Enpp5				
Ddx4	Lrrn4				
Acad12	Otud7b				
Tns1	4930481A15Rik				
Myct1	Dgat2				
Vps37a	4932431P20Rik				
Ipo8	Fgd5				
Brwd3	Mief1				
Sgpl1	Exosc10				
Smcr8	Chpf2				
Tgoln1	Pxylp1				
Tnrc6b	Snx1				
Gm17067	Mxd4				
Ergic1	Rassf8				
Glp1r	Tmem116				

miR-5119	miR-671-5p	miR-486-5p	miR-451	miR-122-5p	miR-21-5p
Igsf23	Phf21a				
Pde10a	Mmp16				
Nrg1	Cdh11				
Xpo4	Leprel1				
Pdzd2	Tcl1b1				
Unc5d	Chrnd				
Zfp932	Ifit1				
Hpse2	P2ry4				
Ttc21a	A130051J06Rik				
Kcnab1	Pola2				
Rnf157	St8sia1				
Mab21l2	Lrrc66				
Snap23	Capn2				
Kcnn3	Zdhhc18				
Tial1	Sfmbt1				
Ppp1r3b	Adamts4				
Cul5	A930011G23Rik				
Arhgef28	Plagl2				
Npas2	Fam160a2				
Casc5	Hbegf				
Nkap	Zfp418				
Slc12a7	Dusp10				
Fam184a	Fam109b				
Trmt44	Adprhl1				
Dusp19	Cyp4f13				
Drg1	Lnp				
Nmd3	Nrbf2				
Agtrap	Scgb1a1				
Cbl	Pdcd1				
A930018M24Rik	Agpat3				
Itgb8	Rab3gap2				
Slc30a1	Ubtd2				
Lss	Ms4a15				
Stil	Ptdss1				
Fgd4	Treml2				
AB124611	Fkbp8				
Ankrd12	Cdhr5				
Cacna1d	Zfp90				
Abhd13	Atp6v0d1				
Slx1b	Hgsnat				
Lrrc9	Cep76				
Slco3a1	Slain2				
Atp2b3	Fibin				
Dlgap1	Rdx				
Plxdc2	Mmp25				
Cdc6	Acot6				

miR-5119	miR-671-5p	miR-486-5p	miR-451	miR-122-5p	miR-21-5p
Ticam2	Stk4				
Klhl31	Hcn2				
Suv420h1	Defb23				
Zfhx3	Dhx35				
Trip11	Atp1b3				
Mfap1b	Cftr				
Kcnb1	Dtna				
Trip6	Herc1				
Ift22	Xxylt1				
Zfp618	Creb3l3				
Cyp4f14	Armc10				
Mars2	6330403A02Rik				
Sema5a	4933414I15Rik				
Gm15446	Tgfb3				
Zfp612	Pnpla5				
Nek9	Bin3				
Lphn3	Bcat2				
Scaf11	Lrtm1				
Tmem207	Bcorl1				
Chst2	Dyrk2				
Socs4	Dbnl				
Irs1	Map2k6				
Akr1b10	Vasn				
Pias2	Chga				
M5C1000I18Rik	Synj1				
Gabrb2	Dzip1l				
Dhrs9	Parpbp				
Ccdc71l	D630045J12Rik				
Frem2	Rbmx				
Tfdp2	Plp1				
5430435G22Rik	Col8a1				
Sardh	H2-Q4				
Entpd5	Myo5b				
Mbl1	Mgam				
Rab39	Bbs4				
A4gnt	Csdc2				
Fam57a	Appbp2				
Dcbld2	Arap1				
Alg5	Saa2				
Exoc5	Pa2g4				
Gopc	Rnf166				
Gm5084	Arid1b				
Eme2	Scaf11				
Twist2	Uroc1				
Sppl2a	Acot8				
Bmpr2	Lin7a				

miR-5119	miR-671-5p	miR-486-5p	miR-451	miR-122-5p	miR-21-5p
Ntrk2	Dap3				
Tpte	Gbp4				
Gne	Sema4b				
Chdh	Atg16l1				
Cep41	Ppp1r7				
Irak1	Colec12				
Mboat2	Ubap1				
Prkca	4933411K20Rik				
Cntn2	Slk				
Wdr20	Habp2				
Brip1	Mtch1				
Adcy1	Rprml				
Zfp704	Hspb7				
Mau2	Gm996				
Sntb2	Lrrc9				
Ccbe1	Cdc42bpb				
Anxa5	Pfn2				
Ppm1h	Nxnl1				
Zfp354b	Ccdc24				
Zbed6	Tfap2c				
Mapre1	2700081O15Rik				
Ccdc68	Slc8a1				
Cadm1	H2-T22				
Cmklr1	Mob1b				
Mrpl35	Dclk1				
Cul2	Bbs1				
D430041D05Rik	Plbd2				
Stxbp5l	Fam163b				
Aldh7a1	Sept9				
Gm6614	1810011H11Rik				
Usp46	Rab3d				
Heatr3	Nr3c2				
Il23r	Kcnn3				
Zyx	Chmp4c				
Slc4a8	Amigo3				
Nupr1l	Syt12				
Map1b	Cdca7l				
Ccdc96	Faf2				
Ptcd3	Ecm2				
Rhobtb2	Agfg1				
Gorasp1	Mlec				
Serpinb5	Ppp1r37				
Il4ra	Folr2				
Slc5a8	Mapk9				
Oas1a	Usb1				
Ccng1	Fkbp5				

miR-5119	miR-671-5p	miR-486-5p	miR-451	miR-122-5p	miR-21-5p
Alyref	Dysf				
Fbxl17	Depdc5				
9130230L23Rik	Fam71b				
Med17	Fam192a				
Ssbp2	Iqch				
Kcnj12	Plekhn1				
1700055N04Rik	Slc8a3				
Rrp7a	Crlf2				
Fam204a	Lins				
Nup133	Rhobtb3				
Pdlim1	Pphln1				
Trim7	Agpat5				
Ppp1r2	C8a				
Hsf1	Mbd3				
Smarcal1	Spib				
Fbxo45	Sh3glb2				
Pvrl3	Pced1a				
Trpm6	Pde12				
Bcl2l11	Max				
Fbxo39	Endou				
Scai	2310079G19Rik				
Gmcl1	Ttc21b				
Acr	1700014D04Rik				
Harbi1	Frmd5				
Kcnt2	Nampt				
Gimap3	Klhl8				
Crem	Abl1				
Uggt2	Gm53				
Asf1a	Picalm				
2310057M21Rik	Cyp2a5				
Kl	Katnbl1				
Scube1	Prpf40b				
Uevld	Pcsk7				
Arhgap1	Pth1r				
Cox15	Nhej1				
Anks4b	Gramd1a				
Kdm5c	Ptgis				
Hcfc2	Ccdc88c				
Kiss1r	Ganab				
Nipal3	Dclk2				
Itga4	Arhgap31				
Zbtb4	Gm2564				
Amer2	Slc10a6				
Atxn7l3b	Sec14l3				
Zfp768	Colq				
1700013F07Rik	Nlrp6				

miR-5119	miR-671-5p	miR-486-5p	miR-451	miR-122-5p	miR-21-5p
Sfmbt1	Shc2				
Enox2	Prkdc				
Ushbp1	Dcaf5				
Lrrc57	Cyp2b10				
Ocstamp	Golga2				
Nat1	Zbed3				
Igf2bp1	Tomm40l				
Gemin5	Mrps27				
Nfam1	Zdhhc3				
Rsbn1l	Gm765				
Otud6b	Nsun6				
Tmem261	Dedd2				
Cd244	Tnfaip8l1				
Zfp521	Srcin1				
Pcdhb9	Epha5				
Fdft1	Clip1				
Stx17	Mief2				
Clec4g	Dgcr8				
Dhrs7b	Tbc1d10b				
Cd200r1	Scube1				
Ndufaf4	Zfp507				
F830016B08Rik	Man2b1				
Cmtr1	Wwp2				
Rab3b	Extl2				
Dydc2	Chrm2				
Kctd1	Map2k3				
Cul3	Prdm1				
Golga3	Ap5z1				
Smim8	Brms1				
5830415F09Rik	Medag				
Med13	Srr				
Cdk7	Sarnp				
Bhlha9	Agr3				
Nov	Zdhhc17				
Lrig2	Sars				
Egfr	Tcte2				
A430033K04Rik	Brinp2				
Eef1e1	Crb1				
Zfp202	Ston2				
Dcdc2a	Rtf1				
Lsmem1	Ranbp3				
Pacrgl	Spns3				
Zbtb21	Acnat2				
Lrrc66	Hoxd9				
Epha7	Zfp397				
Slc25a38	Rap2a				

miR-5119	miR-671-5p	miR-486-5p	miR-451	miR-122-5p	miR-21-5p
Tmem45a	Prss38				
H6pd	Mex3a				
Unc5cl	Glce				
Vwa9	Fhdc1				
6030419C18Rik	Kcnj13				
Mettl16	Plcb4				
Nmrk1	Fuca1				
F930015N05Rik	Runx1t1				
Tmem184c	Gm6970				
Ubn2	Efhb				
Ppdpf	Trim54				
Ankrd33b	Gck				
Nlrc3	Ugt2b1				
Gm14403	Kcmf1				
Ap5m1	4933416C03Rik				
Kbtbd2	Gas2l2				
Kcnh5	Zbtb7a				
Cbx6	Dnajc1				
Pla2r1	Fads6				
Ggt7	Nlgn3				
Syt14	D430019H16Rik				
Ptgs1	Pole				
Pla2g12b	Rhoh				
Cyp2j5	Cbx8				
6330403A02Rik	Fbf1				
Rps7	Rras2				
9530003J23Rik	Commd10				
Trim61	Grhl1				
Nrd1	Caln1				
Chrd	Gcc2				
Fastkd1	Ascc3				
Hilpda	Entpd6				
Zfp738	Hrh4				
BC004004	Man1a2				
Tbc1d25	Wdr45				
Reep3	Cercam				
Calcoco1	Angptl2				
Hist1h4j	Pafah1b2				
Rdm1	Pura				
Srcin1	Dram1				
Steap3	Urgcp				
BC005624	Ccdc137				
Map3k19	Stra6				
Uxt	Ccdc3				
Gfod2	Scgb1b27				
Erbb2ip	4921511H03Rik				

miR-5119	miR-671-5p	miR-486-5p	miR-451	miR-122-5p	miR-21-5p
Dyrk1b	Slc18a1				
Cyp2j6	Dennd4b				
Slc35a5	D3Bwg0562e				
Il2ra	Mat2a				
Dlg2	Phyh				
Gas2l3	Zdhhc12				
Alpk1	Ctsk				
Rtca	Agpat9				
Srpx2	Smyd1				
Ints10	Tor4a				
Bbip1	Ticam2				
Cep76	Nckap1l				
Siae	Bcap29				
Nkx2-5	Sik3				
Slc16a12	Fbxl17				
Dcp2	E2f3				
Pld5	Pik3r6				
Ube2w	Ddx6				
Esco1	Tomt				
Fubp1	Msantd3				
Bag5	Ces4a				
Asxl2	Loxl1				
Rif1	Hal				
Zfp82	Wnt16				
Gjc1	Gemin4				
Ntn3	Arid5a				
Adamtsl5	Itih4				
Zfp623	Cep63				
BC024978	Zbtb34				
Ikbkg	Atg2a				
D1Ertd622e	Trpm2				
Zfp329	Aamdc				
Mroh2a	Ccdc178				
Rps6kl1	Epb4.1l2				
Anks1b	D930020B18Rik				
Pyroxd2	Sigirr				
Ybey	Acsbg2				
Plekhf1	Gosr1				
Mre11a	Zfp345				
Slc4a1ap	Sell				
Naf1	Nuf2				
Sp5	Sde2				
Atp8a2	Gm10615				
Tm9sf3	Trim62				
Alad	Tm7sf3				
Vps33a	Naa11				

miR-5119	miR-671-5p	miR-486-5p	miR-451	miR-122-5p	miR-21-5p
Hemgn	Lss				
Tmem246	Vps13d				
Ccbl2	Tram1l1				
Mapk1	Itgax				
Pvalb	Mios				
Dock4	Gemin8				
Srsf1	Ccdc103				
Sphk2	Dpp4				
Ebag9	Lingo2				
Ndufa4	Ufl1				
Synj2	Mboat2				
Ercc3	Cmpk1				
Rab3il1	Pi4k2a				
Chd7	Epb4.1				
Lurap1l	Nepn				
Pde7a	Scrib				
Efhb	Ptger1				
Nup98	Tie1				
Aars	Cd101				
AY358078	Synj2bp				
Umps	Atp2a3				
Ctnnd2	Phf21b				
Tbc1d12	Kif14				
Atp5j	Gigyf1				
Stam	Tuba1a				
Igf1	Cml2				
Aasdhppt	Tmem151a				
Sftpd	Os9				
Zeb2	Ttyh2				
Ccdc130	Madd				
Rab44	2610021A01Rik				
Slco2a1	Magea10				
Nfix	Haus6				
Dtwd2	Ly6d				
Srgn	Sardh				
Hist1h3h	Dhh				
Zc3h12a	Pip5k1c				
Muc20	Tulp3				
Tmem127	Cxcl14				
Acbd3	Cdc6				
Fbxo8	Angptl7				
Mapk10	Clec4f				
Fbxo30	Idh2				
Eefsec	Rhbdd2				
Lsm11	Podn				
Prr15	Gdpgp1				

miR-5119	miR-671-5p	miR-486-5p	miR-451	miR-122-5p	miR-21-5p
Kcnj8	Trpm4				
Lrrc39	Brat1				
Alkbh4	Nicn1				
Nat8l	Ucp2				
Rrp12	Ptbp1				
Ufm1	Nploc4				
Camk2b	Scoc				
Polr1e	Asic1				
Cdh11	Grip1				
Adat2	Nek5				
Eef1a2	Ric8				
Nrcam	Cmtr1				
1810037I17Rik	Folh1				
Smad4	Rsbn1l				
Igsf10	1600014C23Rik				
Dennd6b	Mixl1				
Hspa4l	Dnajc25				
Ly6a	Slc25a19				
Foxj3	Arhgap8				
Idnk	Sdcbp2				
Srrm4	Aoah				
Anapc10	Defb12				
Tsc22d2	Cdc42se2				
Fam172a	Clic4				
AI846148	Efna3				
Megf6	Ubald1				
Thrb	Lsamp				
Arhgef26	2210018M11Rik				
Tor4a	Fkbp15				
Oxtr	Vps13a				
Asb2	Csnk2a1				
Mark1	Ninj2				
Rnmtl1	Cdk9				
Zfp119a	Clmn				
Haus8	Polr3a				
Mphosph6	Map1s				
G0s2	Vps53				
Ska1	Ppat				
Asb3	9230104L09Rik				
Zcrb1	Paip2b				
Clec2h	Hapln3				
Kmo	Rmi1				
Nt5c2	Klhdc8b				
Pde4c	Fam149a				
A830010M20Rik	Grm2				
Clp1	Lrrc58				

miR-5119	miR-671-5p	miR-486-5p	miR-451	miR-122-5p	miR-21-5p
Ifnar1	Pnkp				
Ipo9	Zwint				
Ankrd42	Prokr2				
Zfp945	Sh2d6				
Fam32a	Mtrr				
Atp5b	Nrip3				
Adhfe1	Ism2				
Mta1	Cntnap5a				
Gramd1a	Fbln5				
Raly	Gm7030				
Nom1	Mipep				
Otulin	Lias				
Srek1	Sdr42e1				
Sertad1	Tnfrsf9				
1700019N19Rik	Prpf4b				
Sumf2	Rgs2				
Ufl1	Msr1				
Cys1	Coa5				
Gm21992	Smim20				
Dnhd1	Thrap3				
Polh	Ttl				
Cnppd1	Eogt				
Gphn	Cadps2				
Pifo	Chchd3				
Pacrg	Lix1l				
Pcdhb7	Rbm12b1				
Dhdds	Gm15821				
Rdh7	Fchsd2				
Rabggta	Leng9				
Klhdc8a	Ephb2				
Herc4	Cops7b				
Gm9776	Apex2				
Rad50	A630033H20Rik				
Kdm5d	Anks1b				
Txndc9	A830010M20Rik				
Prss41	2310002L09Rik				
Ptpn11	Tor2a				
Ctdsp1	Ralyl				
Tmigd1	Mtfmt				
Kcnrg	Appl1				
Pms2	Alpk1				
Gabpb1	Cdk8				
Mcm2	Slitrk4				
C4b	Rbbp4				
Helz	Shf				
Terf1	Sost				

miR-5119	miR-671-5p	miR-486-5p	miR-451	miR-122-5p	miR-21-5p
Susd3	Strbp				
Hnrnpr	Ms4a1				
Emc7	Cnih3				
Trpv1	Ccpg1				
Zfp30	Olfml3				
Trp53bp1	Cyp4b1				
G630090E17Rik	Nphp1				
Taco1	Lax1				
Nudt15	Tnip3				
P2ry2	Smad7				
Angpt2	Sh3gl1				
Fancf	Serhl				
Inha	Necab3				
Ddx19b	Emc7				
Map1a	Cd274				
Ugt3a2	Ythdf3				
Dars2	Zfp92				
Gpc4	Abcb11				
Ppp1r14a	Ndst2				
Tmem45b	Ccng2				
Rprd1a	Prdx3				
Fam171a1	Gm13251				
Mast2	Tmem47				
Tlk2	Tex264				
Cyp3a13	4930427A07Rik				
Prpf39	Naa50				
Hk2	Rhbdl3				
Rnf214	Dpep2				
Ubb	Limd2				
Kazn	Szrd1				
Ces2b	Cebpg				
Rtkn2	Lgals12				
Tubgcp5	Ctsz				
Rnf8	Pfn4				
Zcchc18	Tpgs2				
Zrsr2	Alpk2				
Tbc1d30	Cep72				
Gm4980	Hectd3				
Ccdc58	Plaur				
Cnn2	Mrgbp				
Lcn4	K230010J24Rik				
Me2	Camkv				
Mmaa	Tmem163				
Marc1	Rasgrp4				
Ppp1r36	Sel1l				
Ndufa12	Pot1b				

miR-5119	miR-671-5p	miR-486-5p	miR-451	miR-122-5p	miR-21-5p
Ddx51	Tcl1				
Tbp	Ankrd33b				
Cilp	Aco2				
Lman2l	Clic1				
Cr1l	Kif19a				
Rgs18	Ntn3				
6430571L13Rik	Scrn3				
Hist1h1d	7420426K07Rik				
Kif18a	Cnksr3				
Fcf1	Rgl2				
Rnft1	Siah2				
Srl	Rmi2				
Atpaf1	Rbpjl				
Sema3d	Mcoln2				
Nkx2-1	Rgs20				
Nucb2	Efcab1				
Knstrn	Slc38a3				
Lsm14a	Lif				
Nrxn2	Acpp				
Jmjd4	Snapc1				
3110062M04Rik	Exd2				
Erlec1	Cd82				
Ppp2ca	Zfp109				
Gm3448	Higd2a				
l7Rn6	Aph1c				
Hars2	Hmgcr				
Shoc2	Pdc				
Polr3k	Terf2ip				
Ces1f	Cemip				
Ppp1r7	Derl1				
Rac2	Tatdn2				
Sugt1	Lphn2				
Hhip	Fastk				
Lmbr1	Zfp410				
Necab3	Anln				
Ddx25	Sorbs2				
Gabbr1	Pigm				
Derl2	Cggbp1				
Ndst4	Adamts15				
Cyp3a59	St7l				
Lsm6	Dgkh				
Isy1	Pafah2				
Slc17a2	Arhgef10				
Zfp78	Kcnh5				
Mmp12	Micu1				
Caln1	Eml1				

miR-5119	miR-671-5p	miR-486-5p	miR-451	miR-122-5p	miR-21-5p
Crispld1	Wdr20				
Aadat	Bbox1				
Tspan32	Synb				
Pold4	Itgb5				
Ext1	Hspa12b				
Wdr12	Ndrg1				
H13	2810006K23Rik				
Dusp23	Ago4				
Scara3	Palld				
Megf9	Plscr4				
Ggt5	Alas2				
Mfap5	Gramd3				
Rhbdd2	Rcan1				
Jam3	Wtap				
Satb1	Jdp2				
Fam65a	1810049J17Rik				
Cacna1c	Wdr5				
Mplkip	Ier3ip1				
Ccs	2210016L21Rik				
Snx18	Atp8a2				
Zfp2	Ppcdc				
Lars2	Cd3d				
Glrx2	Dynlt3				
Nepn	Rufy3				
Ucp2	Ptger3				
Zfp46	Tuba4a				
Serpina3m	Nab1				
Aadac	2610034B18Rik				
Asic5	Mdga1				
Shq1	Stx7				
Ankrd9	Rsph4a				
Gata6	Rsl1d1				
Cd300lg	Tmem214				
Prkcq	Aen				
Gprasp2	Ulk4				
Rpp40	Prkg2				
Zfp956	Tmf1				
Dusp12	Cyth4				
Btla	Kcnn1				
Dedd2	Ids				
Tmem256	Il12rb2				
Srprb	Fndc3b				
Zfp318	Helz				
AU021092	Tbp				
Narf	4833439L19Rik				
Casp8	Tbl1xr1				

miR-5119	miR-671-5p	miR-486-5p	miR-451	miR-122-5p	miR-21-5p
Lgals3bp	Plcb3				
BC089597	Ttll9				
Ccdc86	Fkrp				
Rps15a	Cnrip1				
Flt3l	Cstf2				
2210018M11Rik	Agap1				
3110001I22Rik	Mpnd				
Acsm1	Gm826				
Gm10638	Exoc6b				
Plekhf2	Sc5d				
Oxnad1	Dcaf7				
Snrpn	Cd160				
4930562C15Rik	Stx19				
Rnf41	Atxn10				
Eif5b	Spred3				
Tpp1	Saal1				
Slc8a1	Rsc1a1				
Fam65b	Il17c				
Dcun1d5	1110058L19Rik				
Gpr137b	Rab21				
Arhgap24	Lamc3				
Rrp1b	Sptlc2				
1200014J11Rik	Pcsk2				
Csnk1a1	Magi1				
Ttc21b	Slc6a12				
Fbxw17	Fam131a				
Derl3	Dtnb				
Tmem173	A530016L24Rik				
St14	Tpcn2				
Timm17b	Wfdc12				
Zfp639	Psors1c2				
Hs6st2	Taf5l				
Nfe2l1	Gm27179				
Jup	Csnk1d				
Atf2	U2surp				
Ackr2	Gm9776				
Aspa	Ngef				
Mthfsl	Taf10				
Ablim1	Enox2				
Emp1	Rasd2				
Gnb4	Stradb				
Fga	Zbtb1				
Cenpa	Bysl				
Ism2	Dirc2				
Txnip	Metrnl				
Prpf38a	Tcf20				

miR-5119	miR-671-5p	miR-486-5p	miR-451	miR-122-5p	miR-21-5p
Slfn3	Reep5				
Rhbdl3	Sh3glb1				
Sirt7	Med25				
Trim34b	Obp2a				
Trim34a	Snip1				
Cyp3a25	Hnrnpc				
Tcea3	Gm26992				
Fam58b	Cinp				
Oscp1	Dpcd				
Mnat1	Slc38a4				
Siah2	Spef1				
Hjurp	Zbtb37				
Tra2b	Sln				
Prpf6	Acot3				
Cldn25	Gm9922				
Narg2	Homer1				
Rundc1	Cacna2d4				
Preb	1700018B08Rik				
Rpap1	Lsg1				
Cnrip1	Zfp563				
Uba5	Kat6b				
Odf2l	Atf7ip				
Mapk13	Pik3ca				
Plin3	5330417H12Rik				
Espl1	Spaca4				
Foxp4	Rab9				
Krit1	Gm14543				
Nipa2	Chmp7				
Acad9	Gmps				
Lpin1	Ift46				
Mtnr1a	Naif1				
Hadhb	Pon2				
Fli1	Zfp867				
Ccdc134	Phlpp1				
Dlg5	Ppp4r1				
Eif4g2	Lynx1				
Gpr149	Timm22				
Atp6v1e1	Pex10				
Cdkn3	Spsb1				
Dpm1	Sh3bgrl3				
Tiparp	Pnma3				
Ephb6	Dhrs1				
Traf5	Zscan25				
Trappc6b	Grk5				
Man2c1	Add1				
Stoml2	Bcl7c				

miR-5119	miR-671-5p	miR-486-5p	miR-451	miR-122-5p	miR-21-5p
Npc2	Zfp706				
Ret	Rabif				
Afg3l2	Mfi2				
Sorbs3	0610009O20Rik				
Casp12	Rpp14				
Agap3	Irf5				
Ubap2	Hnrnpu				
Gspt1	Lrrc2				
Pfdn2	Sap30bp				
BC005537	Gatc				
Lcat	Wfdc8				
Emc10	Atcay				
Fam49a	Fbxo8				
Ftsj1	Exog				
Frg1	Ccdc127				
Incenp	Mdga2				
Naalad2	Msn				
F11r	Zfp37				
Timm17a	Ext1				
Mpi	Shq1				
Nudt18	Fcrla				
Vil1	Sgol1				
Apcs	Smad2				
Chic2	Pdpn				
Dlst	Pitpna				
Adrb3	Aptx				
Bambi	Usp48				
Rb1cc1	Rhbdl2				
Timmdc1	Gins4				
Atg4d	Slc25a37				
Fam65c	Slc28a3				
Rps6kc1	Slc27a4				
Stk24	Cd8a				
Dppa1	Pou3f3				
Mettl21c	Tdo2				
Gfra1	Epb4.1l1				
Tcf12	Hdhd2				
Cript	Evc				
Oit3	Usp39				
Amz1	Inpp5k				
Litaf	Kcnrg				
Gm13889	Akr1e1				
Mfng	Clstn1				
Ero1l	Dsc2				
Trib1	Dnajc10				
Nedd4l	1700026D08Rik				

miR-5119	miR-671-5p	miR-486-5p	miR-451	miR-122-5p	miR-21-5p
Glyat	Nphp3				
Ptges3l	Dennd6b				
Slc37a2	Uqcr11				
Arhgap42	Prps1l1				
Slc13a1	Amotl2				
Lrrc3b	6330416G13Rik				
Iars	Qpctl				
Timm21	Cant1				
Ganab	Phospho2				
Atmin	4930523C07Rik				
Bckdk	Slc36a2				
Sqstm1	Kpna6				
Rab9	Lmf1				
Col6a2	Zzz3				
Pelp1	Cr1l				
Zbtb3	Dkk3				
Dock1	Myo9b				
Cdk2ap1	Celf2				
Ociad1	Wdr16				
Ttn	Brd2				
Map2k3	Tubb2b				
9930021J03Rik	4930595D18Rik				
Cyp4f15	Tfam				
Serpina3k	Med23				
Fmo5	1700017B05Rik				
4930523C07Rik	9130008F23Rik				
Mbd2	Acvr1c				
Gpi1	Adarb2				
Ythdf2	Kpna1				
Ube2a	Acot4				
Zfp846	Plekhm2				
Filip1l	Gda				
Sdhaf2	Mtap7d3				
Scg3	Tstd2				
Tom1	Ldb1				
Mastl	Svip				
Eif2d	Rbm43				
Krba1	Hmgn2				
Mmp15	Arfgef2				
Liph	Tgfb2				
Snf8	Cct3				
Hps5	Galk2				
Dhx38	Dusp9				
Klk13	Cnih4				
Pld3	Hsf2				
Pgam5	Adipor1				

miR-5119	miR-671-5p	miR-486-5p	miR-451	miR-122-5p	miR-21-5p
Rpl7l1	Spire1				
Metrnl	Krtcap2				
Eno3	Kcnf1				
Mtmr12	Tlr8				
Smpd4	Pdzrn3				
Rhoj	Rpgrip1l				
Stau1	Prdm15				
Sdccag8	Elp6				
Vapa	B3galt2				
Ppp1cb	Pdx1				
Akap10	Mtch2				
Slc25a36	Thnsl1				
Wiz	Prickle1				
Mgat2	Tbk1				
Cyp2a12	Bcl2l1				
Top1mt	Zfp53				
Rab11b	Elovl2				
Cep44	Tmc6				
Myl7	Sema3b				
2810459M11Rik	Gm8909				
Rsl1d1	Trim68				
Efnb1	Sft2d2				
Dgkh	Cox6b2				
Prox2	Dusp15				
Sbk2	Ddit4l				
Ldlrap1	Fsd2				
Ccdc108	Vamp5				
Sod3	Syn2				
Skap2	Kcna4				
Spice1	Sox11				
Ikbkap	Cand1				
Ak3	Rab23				
Dut	Eif5b				
Ubl7	Odc1				
Tyms	Lama4				
Ormdl1	Tmem238				
Hsf2	Phykpl				
Ugt2a3	Stk16				
Timm13	Alg11				
Fignl2	Chsy1				
Cntnap5b	Tspyl3				
Mynn	Dolpp1				
Ifi27	Sftpb				
Dync2h1	Aqp9				
Lrrk1	Rhoj				
Tnrc6a	Gng5				

miR-5119	miR-671-5p	miR-486-5p	miR-451	miR-122-5p	miR-21-5p
Atf1	Btla				
Nhsl1	Ccdc167				
Tsku	Tbx5				
Upb1	Aldob				
Psg18	Mknk1				
Csad	Ppfia3				
Nhej1	Acvr1				
Nfkbia	Tmem178				
BC021614	Pkd2				
Trpc1	Ubox5				
Obfc1	Ptpn11				
Polr3a	Terf2				
Plec	Eif4e				
Tacc2	Itgb3				
Med12	Myo1b				
Gm20425	Ugdh				
Rab2b	Klf11				
Rag2	Mapre1				
Faf1	Pik3ap1				
Rrm1	Prpf31				
Dnajb9	AI118078				
Psd4	Nubpl				
Vipas39	Acsm1				
Inhbc	Lpcat1				
Dtnb	Nup50				
Nrp1	Nr2c1				
Tmed9	Slc16a3				
Gm20815	Rbm19				
Chmp7	LHB				
Tbc1d10a	Tmem252				
Pelo	Trp53rk				
Mapk8	Cyld				
Cd248	H2-T10				
Ddrgk1	Cd226				
Agpat5	Plekho1				
Trpm4	Snrpe				
Tcf20	Tmem42				
Tbk1	Rexo1				
Actr5	Phtf2				
Pdhx	Trappc12				
Ergic2	Sass6				
Fsd2	Smc3				
Acot9	Clasp1				
Hoga1	Pnoc				
Rab5b	Rbm17				
Dnajc15	Entpd1				

miR-5119	miR-671-5p	miR-486-5p	miR-451	miR-122-5p	miR-21-5p
Mtf2	1700019N19Rik				
Spata6	Ccdc68				
Klri1	Rad21				
Rai1	Rfxank				
Nkrf	Tmem57				
Nptn	Gnptg				
Hdac3	Tpp1				
Mtmr7	Aldh16a1				
Palmd	Gja1				
Lrp10	1700012B09Rik				
Chmp2a	Ap3m2				
Agk	Ppfia4				
Ap5z1	Zfp128				
Ccdc163	Nrg4				
Rps19	AI317395				
Ranbp2	B3gnt5				
Tmem194b	Rab3il1				
Tle1	Smim1				
Ssr3	Hexim1				
Lpar6	Aifm2				
Rsrc2	Prep				
Ciapin1	Mblac1				
Gabrg2	Nub1				
Suz12	F830016B08Rik				
Ndufaf6	Kcnq5				
Rere	Stx4a				
Abcb11	Il23r				
Nufip1	Atg7				
Rpap3	Traip				
Abcf3	Zfp13				
Atg13	Ddx19b				
Csnk2a1	Slc7a10				
Pglyrp2	Btg1				
Trim62	Zfp868				
Zmynd8	Cir1				
Bcat1	N4bp3				
Pnpla8	Ptges				
Tcp11	Zfyve9				
Cul1	Reep3				
Eid2	Phldb2				
Myh11	Faf1				
Astn2	2810021J22Rik				
Rchy1	Fgfr1				
Mettl1	Rad51d				
Cyp4f13	Hnrnpa2b1				
Syde2	Tex26				

miR-5119	miR-671-5p	miR-486-5p	miR-451	miR-122-5p	miR-21-5p
Psma2	Calcoco1				
Stbd1	Leprot				
Zfp532	Pias2				
1700047I17Rik2	Fam105a				
Ssr2	Rer1				
Phtf1	Alyref				
Slc36a4	Zfp273				
Rrp15	Brd3				
Urb2	Amd1				
Pole	Zfp933				
Mios	Serpinb6b				
Rarg	Thrsp				
Pdia3	Ier2				
Ran	Proc				
Denr	Alad				
Nop10	Fem1a				
Dennd4b	Cnga3				
Wdr36	Chordc1				
Gemin6	Cdk6				
Shisa4	Tlr3				
Tpmt	Ubl3				
Foxp2	Rassf9				
Apbb2	Hes7				
Gm16286	Mrpl35				
Gja8	Clec1a				
Zfp12	Ip6k3				
Plce1	Dcun1d3				
Ube2f	Ercc6l				
Ugt2b35	Mpv17l				
Jsrp1	Hist1h4i				
Nr1h4	Trdmt1				
Tpp2	Rsl24d1				
Gpn1	Sfn				
Chfr	Nim1k				
Ppil6	Sugp1				
Rdh14	Tceanc2				
Cep170	St3gal2				
Fgfr2	Adcyap1				
Rbm12b2	Pnpla8				
Vmp1	Gpr3				
2510002D24Rik	Spata6				
Plscr2	Cttnbp2nl				
Cyp2b9	Ppil1				
Aass	Vcpkmt				
Slc3a2	Fam195b				
Rnf103	Emilin1				

miR-5119	miR-671-5p	miR-486-5p	miR-451	miR-122-5p	miR-21-5p
Mill1	Zbtb7b				
Rbm7	Dok2				
Oasl2	Ssr3				
Tmem53	BC017643				
Uaca	Hoxa3				
Nosip	Cenpb				
Loh12cr1	2310005G13Rik				
Ifnar2	Elavl1				
Lsm4	Zfp292				
Cisd1	Tdrd5				
Gltpd1	Elk3				
Ift74	Gabarapl2				
Dhx15	2810459M11Rik				
Ntf5	Slc11a1				
Sema6a	Lrrc10				
Atp2b4	Trabd2b				
Myadm	Brca2				
Cox6a1	Prim2				
Tmem214	Phlda2				
Ing2	Prr18				
H3f3b	Narf				
Pigx	Nadk				
Abhd11	Fastkd2				
Wrb	Smim13				
Abca8a	Tmem167				
Cetn3	Ints1				
Thoc3	Rab8a				
Cnot10	Gdf3				
Rbm28	Chfr				
Tubb6	Lgi3				
Hnrnpdl	F2				
Swt1	Pcdh18				
Nop9	Siglece				
Dnmt1	Btrc				
Kbtbd3	Il6ra				
Ngly1	Fam135b				
Nme1	Calm1				
Pdia4	Zdhhc15				
Fgfr1	Krt7				
Tor1b	Slc51a				
Crot	Mul1				
Txndc17	Vars2				
Sp110	Mpdz				
Def8	Ddx11				
Kti12	Gtpbp8				
Gm2026	Slc25a38				

miR-5119	miR-671-5p	miR-486-5p	miR-451	miR-122-5p	miR-21-5p
Dhx35	Frk				
Hnrnpu	Vasp				
Myl4	Hoxd4				
Il6ra	Exosc2				
Crp	Wnt8a				
Slc25a45	Sdr39u1				
Ptchd2	Tmem51				
Gm21685	Abcg3				
Irx2	Tacc2				
Gm4788	Lyrm9				
Homez	Ube2g1				
Dnajc10	Csnk1g3				
Tspan13	Tspan11				
Nol11	Zkscan4				
Ppif	B3galt6				
Strn3	Egf				
Fam135b	Rin3				
Ugdh	St6galnac2				
Mfsd2b	Crem				
Ncl	Atp5g1				
Il22ra1	Cdh3				
Skp1a	Zbtb9				
2310036O22Rik	Trp73				
Itk	Rif1				
Gimap1	Nnmt				
Fbxw2	Tshr				
Dstyk	Zfp85				
Mdp1	Nrg2				
Trpc4	Ttc33				
Ccl25	Srsf10				
Cyp2b13	Top2b				
Luc7l3	Pnck				
Mrps16	Rab11fip1				
Zfp868	Impa1				
Grin2a	Ift57				
Ift46	Pck2				
Gabrb1	Pcif1				
Eif2b5	2410015M20Rik				
Lancl1	Mtf2				
Lyn	Slc19a1				
Rnf130	Ccdc47				
Lgmn	Slit2				
Fblim1	Ppp1r14a				
Phax	Tkt				
Tmem205	Btg2				
Uap1l1	U2af1				

miR-5119	miR-671-5p	miR-486-5p	miR-451	miR-122-5p	miR-21-5p
Rpl19	Vstm2b				
Dnajb12	Kif1c				
Spata5	Dcaf6				
Ikbkb	Ppp1cc				
Gm20498	Pcsk1				
Pecr	Trim12c				
Fahd1	Rpl6				
Ehd1	Dpt				
C1ql2	Wwtr1				
Rpl26	Egr4				
Dync1li2	Rrp36				
Ndufb2	Nefl				
Lgi1	Tmem176b				
0610040J01Rik	Gabrb1				
Rpp14	Gabrb2				
Clcn3	Alpk3				
Dock9	Prkrip1				
Kctd4	Clstn2				
Limd1	Adamts7				
Glrx	Exo1				
Arid1b	Vmn1r30				
Slitrk5	Tmem86a				
Ptk2	Gnao1				
Fasn	D19Bwg1357e				
Nxph1	Scgn				
Slamf7	Tmem44				
Slc22a12	Timm50				
Cdh10	Ccdc158				
Lamb1	Gm21685				
C2cd2l	Mctp2				
Tusc3	Tspan13				
Rcn1	H2-Q10				
Itfg3	Spats2l				
Cox16	Trafd1				
Ugt2b36	Cdh15				
Gpr108	Ckm				
Polr1a	Gmppb				
Mycbp2	Nat10				
Apip	Mpzl2				
Grb14	Csf1r				
Sh3tc2	Trim72				
Ddx56	Gm10399				
Dis3	Zfp106				
Snap47	Lrrc4c				
Ptpra	Fer				
Fhod3	Plekha1				

miR-5119	miR-671-5p	miR-486-5p	miR-451	miR-122-5p	miR-21-5p
Trappc2l	Poc1a				
Nfatc4	Car8				
Snrpa1	Clec7a				
Cul4b	Fip1l1				
Tmem248	Six1				
Vwa1	Arhgap11a				
Mrpl36	Usp15				
Prdx6	Nudt3				
Nell2	Dsn1				
Wdr92	Aspscr1				
Gm14399	B4galt4				
Med20	Arhgap29				
Tsn	3110040N11Rik				
Il18r1	Ldlrad4				
Slc7a13	Nudt13				
Slc4a3	Yipf1				
Ces1g	Kdm6b				
Pgd	Anapc11				
Rbp1	Tmem59l				
Syt10	Ppp4r4				
Pepd	Scyl2				
Erich5	Akip1				
Dkk3	Pds5a				
2610528J11Rik	Itgb3bp				
Luzp2	Mcm2				
Thg1l	Stt3a				
Prep	Aste1				
Imp3	Mavs				
Bcas2	Evi2a				
Mtpap	Pot1a				
Ier2	Gars				
Hoxd11	Cilp				
AI429214	Racgap1				
Rad51	Ccr5				
Tbc1d2b	Hs3st1				
Dmgdh	Cela1				
Slitrk3	Birc3				
Cyb5r4	Ap5b1				
Ccr8	Trappc10				
Zfp706	Sh3d19				
Azgp1	Dcc				
Krt23	Mill1				
Acvr1	Snx31				
Col6a1	Colec10				
Ffar1	2310045N01Rik				
Epha6	Chic2				

miR-5119	miR-671-5p	miR-486-5p	miR-451	miR-122-5p	miR-21-5p
Prpsap1	Csnk1g2				
1110038F14Rik	Cnpy3				
Syncrip	Hmmr				
Afap1l1	Htr2a				
Ift57	Kcnma1				
Anapc4	Chchd4				
Phospho2	Lacc1				
Ccdc80	Hnrnpr				
Mgst3	Pcsk5				
Ippk	Slc30a2				
Gbp11	Gm2a				
Vbp1	Cyp2d9				
Ndufa11	Mbd2				
Fscn1	Twistnb				
Kap	Cbx5				
Calml4	Sucnr1				
Tro	Cep170				
Hip1	Exoc7				
Fem1c	Timp1				
Zfp26	Ccl24				
Ftcd	Rdh5				
Grhpr	Gnai1				
Hist1h1c	Hlf				
Aldh1l1	Ipo7				
Pcyox1	Gimap3				
Txn2	Zfp846				
Actn1	Lsm10				
Hivep1	Smim7				
Ap5b1	St3gal6				
Acsl5	Stra8				
Lpar1	Aadac				
Acot12	Eif4a3				
Oxld1	Rbck1				
Amy2a2	Rps9				
D230025D16Rik	Itga1				
Ces3a	Psmd5				
Pml	Rbm47				
Acvr1b	Lmf2				
Amy2a3	Slc5a8				
Mrpl37	Rcn1				
Pik3c3	Nkx2-5				
Slc38a4	Cep131				
Rpp21	Atp6v0a2				
Ctsl	Ncstn				
Scaf8	0610007P14Rik				
Usp39	Usp20				

miR-5119	miR-671-5p	miR-486-5p	miR-451	miR-122-5p	miR-21-5p
0610009L18Rik	Klhl7				
Carhsp1	1700003E16Rik				
Atp5a1	Igfbp4				
Atp5o	Slu7				
Ankrd23	Dcun1d1				
Eif4e	Ankrd9				
Slc39a8	Timm17b				
Adipoq	Gtf3c4				
Trim23	Rpp25l				
Slfn8	Kcnj4				
Amigo1	Eefsec				
Fam134b	Trmt5				
Cyp2b10	Ecsit				
Adk	Arpp19				
Slc25a46	Cda				
Alpk3	Tln2				
Copa	Pwp1				
Slc39a6	Hsd3b2				
Sesn2	Myo5a				
Pcolce2	Slc7a6				
Lfng	Fam222a				
Dgki	Abcc6				
Syt15	Gpx3				
Myl9	Mxra7				
Gpm6a	Cyba				
Paxip1	Lrp10				
Mesdc1	Sart1				
Xpo7	0610037L13Rik				
Tuba4a	Oraov1				
Ndrg1	Trim45				
Galnt14	Bst1				
Acox1	Rasgrf2				
Fkbp4	Nsmf				
Emc6	Anp32a				
Acot13	Knstrn				
Adprhl1	Nom1				
Sparc	Zmym2				
Tbc1d1	Cfdp1				
Zmynd11	Oprd1				
Magi1	Aasdhppt				
Fam193a	Neurl2				
Fam84a	Rdh9				
Plcxd1	Gm28046				
Map3k5	Epn3				
1110001J03Rik	Steap2				
Slc25a3	Rhou				

miR-5119	miR-671-5p	miR-486-5p	miR-451	miR-122-5p	miR-21-5p
Gtf2a1	1190007I07Rik				
Sepw1	Syngr2				
Ppp6r3	Fam101a				
Fbxo21	Lepre1				
Hs2st1	Shisa4				
Agpat3	Npy2r				
Sdr9c7	Mfng				
Pck2	Cnot6				
Zap70	Fam179a				
Clta	Smarca4				
Ankrd50	BC049352				
Pin1	Dnaja1				
Etnk2	Tll1				
Letm1	Hivep1				
Grap2	Fga				
Zmym5	Epo				
Ipcef1	Pole4				
Ogdh	Dcxr				
Ncr1	Larp7				
Mdn1	Cep162				
Tcerg1	Agxt				
Myh4	Tmem110				
Plod3	Dusp11				
Kcnk3	Ankrd13a				
0610009O20Rik	Sap30l				
Cinp	Dvl2				
Otc	Zfp101				
Itgb1bp2	Rpl19				
Cand1	Mmadhc				
Cx3cl1	Kmo				
Inpp5e	Oxtr				
Pnpla6	Vash2				
Egr4	Sp4				
Cmtm7	Bckdhb				
Hspb11	Pelo				
Tmco1	Dis3				
Brk1	Glra3				
Zfp873	Atp6v1d				
Nap1l1	Xlr3b				
Cdk17	1110038F14Rik				
Mtcl1	Cyb5r4				
Gpr161	Gpm6a				
Fundc2	Orai2				
Tmem74	Smpd4				
Nwd1	Guf1				
Otud7a	Fbxo38				

miR-5119	miR-671-5p	miR-486-5p	miR-451	miR-122-5p	miR-21-5p
Kdm4b	Abi3				
Nnt	Gm3448				
Psmd2	Gcat				
Prr16	Hfe2				
Dcps	Hsp90ab1				
Trpm3	Klhl26				
Hcar1	Ciapin1				
Slain2	Slc38a10				
Sh3bp5l	Fam210a				
Osbpl2	Akirin1				
Syt13	C87436				
Mark4	Cdca5				
Haus3	March5				
Mum1	Palm2				
Dap3	Zfp94				
Deb1	Sox17				
Sec13	Acaa1a				
Itih4	Cib4				
ZBED6	Traf1				
Usp9x	Stat3				
Adamts4	Pon1				
Grik2	Nudcd3				
Rock2	Rpl7l1				
Tex9	Pcca				
Bnip3	Sntg2				
Prss16	Nipbl				
Gabarapl1	Arl5a				
Ddx11	Supv3l1				
Ighmbp2	Nxn				
Usp34	Ppp2r2c				
Msn	Lipt2				
Wwp2	Mok				
Ppil4	Papd7				
Cog2	Gas1				
Fxyd2	Gypc				
Zbtb10	Ifltd1				
Sf1	Dhrs7c				
Nsmf	Aplp2				
Amy2a4	Drg1				
Sfpq	A930004D18Rik				
Hnrnpk	Umps				
Fbxo28	Tpt1				
Sec62	Mrap2				
Sec24c	1700024G13Rik				
Hadh	Gng2				
AI462493	Pla2g12b				

miR-5119	miR-671-5p	miR-486-5p	miR-451	miR-122-5p	miR-21-5p
Inpp5f	Usp7				
Lyrm2	Ctsc				
Mob2	Tmem209				
Aar2	Galnt16				
Rpl37a	Pitrm1				
Eif4g3	Hmgb1				
Sod1	Lrrc14b				
Bckdhb	Tor3a				
Dpy30	Nucks1				
Ppp2r2c	Slc46a3				
Slc25a12	Bhlhe40				
Fnta	Emc8				
Srsf5	Hnrnpd				
Oaf	Slc52a2				
	Enkur				
	Ube2e3				
	Ufm1				
	Henmt1				
	M5C1000I18Rik				
	Ffar1				
	Fbxo34				
	Hrsp12				
	Mmd				
	Mas1				
	Tmem45b				
	Chst7				
	Tm2d1				
	Mrps25				
	Dbndd2				
	Csrp2				
	Nudt18				
	Agt				
	Il2ra				
	Tmem53				
	Mettl16				
	Coa4				
	Ppil2				
	Mrpl16				
	Ces2a				
	Limk2				
	Traf7				
	Lpin1				
	Luzp2				
	Atp2a2				
	Commd6				
	Kcng2				

miR-5119	miR-671-5p	miR-486-5p	miR-451	miR-122-5p	miR-21-5p
	Pts				
	Serpina12				
	Kcnj11				
	Lzts1				
	Ube2r2				
	Wdr45b				
	Mrpl54				
	Uap1				
	Ptprr				
	Mtmr12				
	Pald1				
	Otud7a				
	Gm16401				
	Gm10221				
	Wdr86				
	Il17ra				
	Ddrgk1				
	Casc5				
	Kifc3				
	Gnrh1				
	Eif2d				
	Slmap				
	Fxyd3				
	Dnajc14				
	Slc7a4				
	Dnaaf2				
	Inha				
	Gpx4				
	Herc6				
	Rspo4				
	Sp2				
	Mrpl51				
	Myoc				
	Pdlim1				
	Fam221a				
	Klhl41				
	Metrn				
	Fgf12				
	Nars				
	Aqp2				
	Sdhc				
	Asb10				
	Cdc23				
	Glrx				
	AW822073				
	Rnf183				

miR-5119	miR-671-5p	miR-486-5p	miR-451	miR-122-5p	miR-21-5p
	Gm10521				
	Trub1				
	Dlgap2				
	Mtrf1				
	Rbms2				
	Cln8				
	Ttll12				
	Dll4				
	Bcl2l2				
	Egln1				
	Rfc1				
	Nup214				
	Tmem261				
	Ptpn21				
	Tert				
	Rab11a				
	Cat				
	Zfp768				
	Dhx15				
	Smim19				
	Sugt1				
	Golga3				
	Sgcg				
	Mphosph6				
	Odf2l				
	Cenpu				
	Psrc1				
	Sult6b1				
	Hax1				
	Tbc1d30				
	Mtif3				
	Fah				
	Ftcd				
	Tsn				
	Nfx1				
	Sae1				
	Dpagt1				
	Cblc				
	Nfe2				
	Xdh				
	Bri3bp				
	Siah3				
	Sesn2				
	Bcl6				
	Gm7008				
	Trub2				

miR-5119	miR-671-5p	miR-486-5p	miR-451	miR-122-5p	miR-21-5p
	9230110C19Rik				
	Pla1a				
	Erp44				
	Fgfr1op				
	Decr1				
	Rabepk				
	Flywch2				
	Fbxw2				
	Pfkm				
	Cdc14b				
	Jup				
	Traf5				
	Pex14				
	Amph				
	Dach2				
	Gmpr				
	Tmem173				
	Rab24				
	Gpr108				
	Rnls				
	Ints10				
	Ctsb				
	Slc39a8				
	Hnrnpll				
	Tpd52l2				
	Mettl24				
	Fam174a				
	Pddc1				
	Eif4g2				
	Chtop				
	E130308A19Rik				
	Myl12a				
	Cul1				
	Abcf2				
	Zrsr2				
	Tmem5				
	Fut8				
	Eef1e1				
	Amz1				
	Aco1				
	Itga5				
	Snai3				
	Myoz1				
	Gm17641				
	Tmem126b				
	Snap47				

miR-5119	miR-671-5p	miR-486-5p	miR-451	miR-122-5p	miR-21-5p
	Ptp4a3				
	Bud13				
	Sqrdl				
	Nxt1				
	Ifitm2				
	Ptar1				
	Zfp39				
	Itm2b				
	Txnl1				
	Chst13				
	Me2				
	Nt5m				
	Kiz				
	Eno3				
	D10Jhu81e				
	Mastl				
	Yae1d1				
	Mrpl13				
	Adat2				
	Klf8				
	Rnf34				
	Prr13				
	Zfp467				
	Gp6				
	Rnf207				
	Ap5s1				
	Cdc14a				
	Kdelr1				
	Tmem106a				
	Cops2				
	Ugt8a				
	Tgm2				
	Gc				
	Scp2				
	Zscan12				
	Trmu				
	Erlec1				
	Mrs2				
	Sin3a				
	Gpihbp1				
	Banf1				
	Gcfc2				
	Fam92a				
	Dmrt2				
	Fam219b				
	Foxf2				

miR-5119	miR-671-5p	miR-486-5p	miR-451	miR-122-5p	miR-21-5p
	Dcp1b				
	Tpm3				
	Ccl9				
	Akr1b10				
	Comtd1				
	Chchd5				
	Taco1				
	Rnd1				
	Slco3a1				
	B9d1				
	Eepd1				
	Cdk13				
	Chrd				
	Mb21d2				
	Commd2				
	Ugt2b34				
	Tagln2				
	Pnpla6				
	Dimt1				
	Echdc3				
	Arhgap24				
	Polr1e				
	Fam65b				
	Pdzk1ip1				
	Anapc15				
	Timd2				
	Secisbp2				
	Dnase1l3				
	Igfals				
	Adrb2				
	Wdr72				
	Filip1l				
	Myo6				
	Mrps7				
	Tgm1				
	Dusp23				
	Irx4				
	Znrf4				
	Ebag9				
	Adamtsl5				
	Scg3				
	Paqr5				
	Pdia4				
	Spo11				
	Gm6614				
	Zfp239				

miR-5119	miR-671-5p	miR-486-5p	miR-451	miR-122-5p	miR-21-5p
	Il15ra				
	Serpina1c				
	Cask				
	Tssc1				
	Rrp15				
	Tmem177				
	Snapin				
	Prdx6				
	Nup35				
	Sdhaf2				
	Nln				
	Slc8a2				
	Smim5				
	Cyp2j6				
	Timm8a1				
	Zfand2a				
	Lztfl1				
	Peli2				
	Ercc8				
	Cfl2				
	Dph3				
	Hnrnpa0				
	Rhpn1				
	Mpi				
	Pdp2				
	Desi1				
	Rgl3				
	Erp29				
	Narg2				
	Mob2				
	Zfp644				
	Dusp12				
	Rccd1				
	Sec22a				
	Rps7				
	Brix1				
	Cldn10				
	C1qc				
	Nucb2				
	Havcr1				
	Tex12				
	Cutc				
	Psmd9				
	Actn2				
	Srd5a3				
	Imp3				

miR-5119	miR-671-5p	miR-486-5p	miR-451	miR-122-5p	miR-21-5p
	Efnb1				
	Rbm7				
	Prdm5				
	Mpv17				
	Lsm6				
	Ccdc125				
	Cebpb				
	Adipoq				
	Gnpat				
	Efcab2				
	Bola2				
	Cdh2				
	Olfr920				
	Cdh8				
	mt-Nd4l				
	Vwa9				
	Naa25				
	Casp8				
	Nmd3				
	Sec62				
	Tfdp2				
	March7				
	Uxs1				
	Klf2				
	Trmt10c				
	Mcm4				
	Pmf1				
	Slc13a1				
	Sept7				
	Lyn				
	Hddc2				
	Csk				
	Acad9				
	Sub1				
	Phactr3				
	Ly6a				
	Ubxn8				
	Acox1				
	Psmc2				
	Gnb1l				
	Mtss1l				
	Eps8				
	Nell2				
	Cdc42ep3				
	Fam149b				
	Brms1l				

miR-5119	miR-671-5p	miR-486-5p	miR-451	miR-122-5p	miR-21-5p
	Bambi				
	Yars2				
	Gstz1				
	Snrnp70				
	Ces1d				
	Nupr1				
	Adhfe1				
	Skida1				
	Lfng				
	Rnmtl1				
	Rcc2				
	Plac9b				
	Zfp788				
	Irf7				
	Snap29				
	Mapkapk2				
	Spin1				
	Ppil6				
	Tsc22d4				
	Tinag				
	Obfc1				
	Mettl21a				
	Mfge8				
	Atp5b				
	Ndufb3				
	Zmynd8				
	Arpc4				
	Lrrc40				
	Slc4a1ap				
	Cltc				
	Fam213b				
	Mrpl4				
	Hadh				
	Klhl11				
	Med7				
	Fancf				
	Stt3b				
	Rasgef1b				
	Ring1				
	Amdhd2				
	Commd8				
	Rnf8				
	Traf4				
	Bet1l				
	Ppp3cc				
	Zfp26				

miR-5119	miR-671-5p	miR-486-5p	miR-451	miR-122-5p	miR-21-5p
	Zfp940				
	Pde6d				
	Mak16				
	Myoz2				
	Tmx1				
	Cradd				
	Elf2				
	Zbtb8os				
	Ict1				
	Rrm1				
	Adck2				
	Ccdc17				
	Dupd1				
	Mrpl57				
	Pold4				
	Mrpl17				
	Top1mt				
	Osgep				
	Isy1				
	Serpina1d				
	Ccar1				
	Memo1				
	1700025G04Rik				
	Med6				
	Fli1				
	Iars				
	Ptprf				
	Nrd1				
	Cldn16				
	Wdr12				
	Lactb2				
	Otc				
	Taf12				
	4933411K16Rik				
	Inip				
	Mccc1				
	Dhx38				
	Txndc9				
	Sept10				
	Fuom				
	Lrpap1				
	Chuk				
	Rpap3				
	Ngf				
	Rad23b				
	Ctbp1				

miR-5119	miR-671-5p	miR-486-5p	miR-451	miR-122-5p	miR-21-5p
	Ttc32				
	Psmb1				
	Asns				
	Cyp2b13				
	Pvalb				
	Car5b				
	Dgcr14				
	Stk24				
	Josd2				
	Anapc10				
	Saa4				
	Ifnar2				
	Tbc1d10a				
	Tmem258				
	Ube2d2a				
	Ing2				
	Gm20498				
	Gipc1				
	Lect2				
	Cox5b				
	Nol10				
	Adrb3				
	Slc25a46				
	Fam122a				
	Tcea3				
	Ntf5				
	Dnajc15				
	Cse1l				
	Ppp1r35				
	App				
	Slc22a1				
	Gmpr2				
	Rit1				
	Ptrhd1				
	Sfr1				
	Zmat2				
	Tor1a				
	Eif3e				
	Slc25a33				
	Cidec				
	Mest				
	2200002D01Rik				
	Dhfr				
	Bclaf1				
	Cd8b1				
	Glrx2				

miR-5119	miR-671-5p	miR-486-5p	miR-451	miR-122-5p	miR-21-5p
	Ms4a8a				
	Ttc30b				
	Hspa2				
	Pet100				
	Nt5c2				
	Grtp1				
	Impa2				
	Sntb2				
	Clpx				
	Kctd3				
	Sergef				
	Gm13889				
	Atg12				
	Skp1a				
	Erich2				
	Pigc				
	Scaf8				
	Myl9				
	Amacr				
	Cox16				
	Syne4				
	Nop58				
	C1qb				
	Sdpr				
	Bcat1				
	Zfp639				
	Nap1l1				
	Mcm5				
	Rtn4rl1				
	Apod				
	Yipf7				
	Zbtb24				
	Afm				
	Fam114a1				
	l7Rn6				
	Rsl1				
	Pcmt1				
	Cops6				
	Emc10				
	Upp1				
	Nostrin				
	Ipo5				
	Tgfb1				
	Amigo1				
	4933434E20Rik				
	Lrrc25				

miR-5119	miR-671-5p	miR-486-5p	miR-451	miR-122-5p	miR-21-5p
	Tmem218				
	Cst3				
	Glyat				
	Zfand5				
	Uaca				
	Atp5j				
	Rab4a				
	Upb1				
	Chac2				
	S100a6				
	Acss1				
	Ormdl2				
	2610528J11Rik				
	Ldhb				
	Lilra5				
	Ccdc59				
	Trappc2l				
	Gja8				
	Emc6				
	Terf1				
	9430038I01Rik				
	Tspan3				
	Ccdc174				
	Slc3a2				
	Micu3				
	Aldh1a1				
	Fdft1				
	Glrx5				
	Crispld1				
	Epb4.1l4a				
	Ccdc85b				
	Mrpl55				
	Dbf4				
	Ppwd1				
	Tbc1d1				
	Il18r1				
	Kcnk10				
	Gpcpd1				
	Mrpl32				
	Actr10				
	Sec11c				
	Acot9				
	Aars				
	Dcun1d5				
	Slc16a4				
	Kbtbd3				

miR-5119	miR-671-5p	miR-486-5p	miR-451	miR-122-5p	miR-21-5p
	Grpel1				
	Phb2				
	Pex16				
	Cul5				
	F10				
	Ebpl				
	Prmt1				
	Cnot10				
	Slc25a3				
	Slirp				
	Oaf				
	Hao2				
	Luc7l3				
	Polr3c				
	Coa3				
	Arpc3				
	Gm20878				
	1810043H04Rik				
	Hist1h4m				
	Samm50				
	Yaf2				
	H2-Ab1				
	Adck1				
	Lcat				
	Pfdn4				
	AW112010				
	Ddah2				
	Polr2f				
	Ppp1cb				
	Amy2a2				
	Prkag1				
	Amy2a4				
	Amy2a3				
	Rhob				
	Utp3				
	Aspa				
	Usp18				
	Apoc3				
	Lrrc56				
	AI462493				
	Ndufa11				
	Mt1				
	9130401M01Rik				
	Sp110				
	Apip				
	Stx12				

miR-5119	miR-671-5p	miR-486-5p	miR-451	miR-122-5p	miR-21-5p
	Gm14306				
	Timm23				
	Pawr				
	Anapc16				
	Chmp4b				
	Gm2026				
	Mrpl20				
	Wdr1				
	Zfp930				
	BC005624				
	Fth1				
	Scrg1				
	Psmg4				
	Srp19				
	Cct5				
	Sumo3				
	S100a11				
	Fnta				
	Polr2e				
	Hspb11				
	Pdcd1lg2				
	0610009L18Rik				
	Mdp1				
	Grik2				
	Gm14399				
	Ptms				
	Smlr1				
	Snrpa1				
	Fbp1				
	Hscb				
	Nt5c3				
	Mrps33				
	Mrps9				
	Lyz2				
	Spink3				
	Vmp1				
	Mgst1				
	Hilpda				
	Sec13				
	Havcr2				
	Rpl37a				
	Srsf5				
	Ccne1				
	Rpl8				
	Atad1				
	Acot13				

miR-5119	miR-671-5p	miR-486-5p	miR-451	miR-122-5p	miR-21-5p
	Fxyd2				
	2310002J15Rik				
	Gm10118				
